# Advancing molecular modeling and reverse vaccinology in broad-spectrum yellow fever virus vaccine development

**DOI:** 10.1038/s41598-024-60680-9

**Published:** 2024-05-12

**Authors:** Ohana Leticia Tavares da Silva, Maria Karolaynne da Silva, Joao Firmino Rodrigues-Neto, Joao Paulo Matos Santos Lima, Vinicius Manzoni, Shopnil Akash, Umberto Laino Fulco, Mohammed Bourhia, Turki M. Dawoud, Hiba-Allah Nafidi, Baye Sitotaw, Shahina Akter, Jonas Ivan Nobre Oliveira

**Affiliations:** 1 Department of Biophysics and Pharmacology, Bioscience Center, Federal University of Rio Grande Do Norte, Natal, RN 59064-741 Brazil; 2 Multicampi School of Medical Sciences, Federal University of Rio Grande do Norte, Caicó, RN 59300-000 Brazil; 3 Department of Biochemistry, Bioscience Center, Federal University of Rio Grande do Norte, Natal, RN 59064-741 Brazil; 4 Physics Institute, Federal University of Alagoas, Maceio, AL 57072-970 Brazil; 5 Department of Pharmacy, Daffodil International University, Sukrabad, Dhaka, 1207 Bangladesh; 6 Department of Chemistry and Biochemistry, Faculty of Medicine and Pharmacy, Ibn Zohr University, 70000 Laayoune, Morocco; 7 Department of Botany and Microbiology, College of Science, King Saud University, P.O. Box 2455, 11451 Riyadh, Saudi Arabia; 8 Department of Food Science, Faculty of Agricultural and Food Sciences, Laval University, 2325, Quebec City, QC G1V 0A6 Canada; 9 Department of Biology, Bahir Dar University, P.O. Box 79, Bahir Dar, Ethiopia; 10 Bangladesh Council of Scientific and Industrial Research (BCSIR), Dhaka, 1205 Bangladesh

**Keywords:** Yellow fever, Multi-epitope vaccine, Immunoinformatic, B-lymphocyte, Cytotoxic T-lymphocyte, Helper T-lymphocyte, Drug discovery, Molecular medicine

## Abstract

Yellow fever outbreaks are prevalent, particularly in endemic regions. Given the lack of an established treatment for this disease, significant attention has been directed toward managing this arbovirus. In response, we developed a multiepitope vaccine designed to elicit an immune response, utilizing advanced immunoinformatic and molecular modeling techniques. To achieve this, we predicted B- and T-cell epitopes using the sequences from all structural (E, prM, and C) and nonstructural proteins of 196 YFV strains. Through comprehensive analysis, we identified 10 cytotoxic T-lymphocyte (CTL) and 5T-helper (Th) epitopes that exhibited overlap with B-lymphocyte epitopes. These epitopes were further evaluated for their affinity to a wide range of human leukocyte antigen system alleles and were rigorously tested for antigenicity, immunogenicity, allergenicity, toxicity, and conservation. These epitopes were linked to an adjuvant ($\beta$-defensin) and to each other using ligands, resulting in a vaccine sequence with appropriate physicochemical properties. The 3D structure of this sequence was created, improved, and quality checked; then it was anchored to the Toll-like receptor. Molecular Dynamics and Quantum Mechanics/Molecular Mechanics simulations were employed to enhance the accuracy of docking calculations, with the QM portion of the simulations carried out utilizing the density functional theory formalism. Moreover, the inoculation model was able to provide an optimal codon sequence that was inserted into the pET-28a( +) vector for in silico cloning and could even stimulate highly relevant humoral and cellular immunological responses. Overall, these results suggest that the designed multi-epitope vaccine can serve as prophylaxis against the yellow fever virus.

## Introduction

The Yellow fever virus belongs to the Flavivirus genus, a part of the Flaviviridae family^1^. This arbovirus, also known as an arthropod-borne virus, features a single-stranded, unsegmented RNA genome with positive polarity. This genome encompasses a single reading frame, spanning a total of 10,233 nucleotides. Within this genetic sequence, it encodes three structural proteins (E, prM, and C) and seven nonstructural proteins (NS1, NS2A, NS2B, NS3, NS4A, NS4B, and NS5). These protein-coding regions are separated by a short noncoding segment^2^. The structural proteins contribute to the virus's basic structure, playing a essential function in the human immune activities, while the non-structural proteins are responsible for regulatory activities and virus expression^3^.

The discovery of the origin of this virus was possible only through phylogenetic analysis studies showing that the noncoding regions of the virus strains originating in Africa are much more conserved than those of the strains originating in the Americas^1,2^. This indicates that the virus originated on the African continent and spread to the Americas. The places most affected by the virus are the tropical regions of South Africa and the Americas, where its vectors (*Aedes* spp and *Haemagogus* spp) are endemic. From 2019 to 2021, the World Health Organization (WHO) documented yellow fever outbreaks in sixteen African countries (including Chad, Cameroon, Central African Republic, Côte d’Ivoire, Democratic Republic of Congo, Ghana, Niger, Nigeria, Republic of Congo, Senegal, Guinea, Gabon, Togo, Ethiopia, South Sudan, and Uganda) and three countries in the Americas (Venezuela, French Guiana, and Brazil). These occurrences raised significant concerns about disease control^7^.

Transmission of the virus occurs exclusively through the bite of the transmitting mosquito, with no direct human-to-human transmission. There are two transmission cycles of the virus: the urban cycle, in which the *Aedes aegypti* mosquito is responsible for spreading the disease, and the sylvatic cycle, in which several species are involved in transmission, the *Aedes* mosquitoes in Africa and the *Haemagogus* and *Sabethes* mosquitoes in the Americas^3^.

The incubation period of the virus is short, usually 3–6 days, but may be as long as 10 days. Yellow fever occurs in asymptomatic, mild and moderate forms with a nonspecific fever pattern that may or may not be accompanied by jaundice. However, it can also manifest in the severe form, which has a high mortality rate, with affected individuals exhibiting jaundice, organ dysfunction, and even hemorrhage^4^. There is no particular therapy for yellow fever at now, and in milder cases, treatment is supportive of symptoms. Only in severe cases are patients admitted to hospitals for more specific treatment, which is only available in intensive care units^5^. For this reason, the forms of prevention are crucially important in combating this disease, the most important being the vaccine.

Currently available immunizations against yellow fever are attenuated viral vaccines (17DD and 17D-204), which have been shown to be very effective in forming an immune memory. Approximately 90% of vaccinated individuals develop antibodies to yellow fever in less than 1 month^6^. Adverse effects associated with this vaccine are rare in healthy individuals, but are common in children, the elderly over 60 years of age and in individuals with weakened immune systems or hypersensitivity to vaccine components. In these cases, vaccination is not recommended and can lead to local, neurological and even systemic adverse effects^7–12^. While the 17D-204 vaccine has maintained an outstanding safety track record, it's crucial to acknowledge that rare instances (approximately 1 in 250,000 cases) of severe adverse effects have been reported, and, regrettably, these cases can be fatal. These adverse outcomes, which result from the neuroinvasion of 17D-204, are categorized as vaccine-associated neurotropic diseases. They encompass conditions such as post-vaccinal encephalitis, Guillain-Barré syndrome, and autoimmune disorders affecting either the central or peripheral nervous system^13^.

Although the 17DD vaccine has traditionally been associated with a protective humoral immune response against virulent YFV strains^11^, it remains unclear whether the T-cell immune response is also significant. Furthermore, instances of vaccine-induced multisystemic illness have been reported in Brazil, the United States, Argentina, and Australia, with fatal outcomes in most cases^6,14–16^. Furthermore, it's worth noting that vaccine viscerotropic diseases have been documented, which are distinguished by systemic infections affecting multiple organ systems, including liver damage that closely resembles the effects of wild-type infections^17–19^. Considering the recurrence of the diseases, the lack of drugs to treat it, and the side effects that the attenuated virus vaccine causes in certain groups, the development of a subunit vaccine that contains only a portion of the infectious agent is of paramount importance to minimize harm.

In light of this, the present work proposed to use immunoinformatic in order to predict parts of yellow fever virus that are compatible with an acceptable number of HLA alleles, antigenic, non-allergenic, immunogenic, non-toxic, conserved, and have good population coverage for cytotoxic T lymphocytes (CTL), helper T lymphocytes (HTL), and B cells.

From these epitopes, a vaccine template was generated and tested for its receptor binding affinity (TLR-2) and ability to elicit an immune response in the body. Additionally, the study included an assessment of gene expression related to the vaccine in *E. coli*. Furthermore, in silico gene cloning was conducted using the pET-28a( +) vector. This is a consolidated approach that has been used in several studies with pathogenic microorganisms such as SARS-CoV-2^20^, *Streptococcus pneumoniae*^21^, and Alkhurma hemorrhagic fever virus ^22^ as well as with other arboviruses such as dengue^23^, chikungunya^24^, and Mayaro virus^25,26^.

Building upon this foundation, the objective of this research was to develop a multi-epitope vaccine targeting the yellow fever virus. This vaccine was designed to incorporate epitopes characterized by high levels of antigenicity, immunogenicity, conservation, non-allergenicity, non-toxicity, and exceptional population coverage, all with the capacity to induce a robust immune response within the human body. To achieve this goal, an exhaustive examination of the structural (E, prM, and C) and nonstructural proteins (NS1, NS2A, NS2B, NS3, NS4A, NS4B, and NS5) across 196 sequenced viral strains was conducted, coupled with the application of a rigorous molecular modeling approach.

## Methods

**Figure 1:**
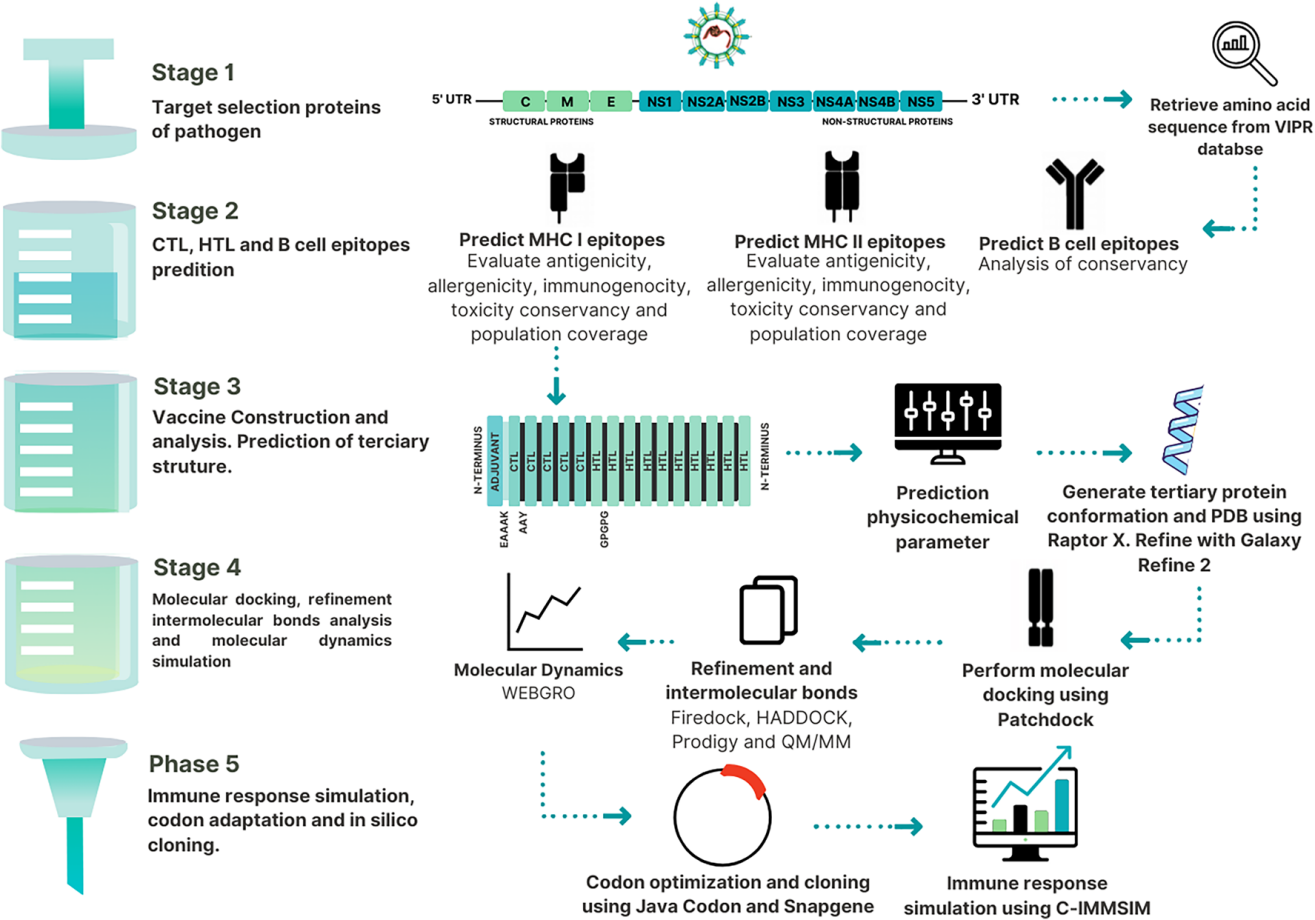
A comprehensive schematic portrayal of the workflow employed in the present study.

The flow chart of the methodology used in this study is shown graphically in Fig. 1. Recently, our research group validated similar immunoinformatics and molecular modeling approaches in the construction of a multiepitope vaccine against Mayaro virus and SARS-CoV-2^25–27^.

### Development/obtaining viral protein sequences

#### Prediction of T‑cell epitopes

Firstly, the primary sequences of the structural (C, M, and E) and nonstructural (NS1, NS2A, NS2B, NS3, NS4A, NS4B, and NS5) proteins of the yellow fever virus (YFV) were obtained from the Virus Pathogen Resource (ViPR) database (https://www.viprbrc.org/brc/home.spg?decorator=vipr)^28^. The data were meticulously filtered according to specific criteria, including Family Flaviviridae, Genus Flavivirus, Species Yellow fever virus, and Global geographic group, leading to a preliminary selection of 385 sequences from the ViPR database; this was further refined to 196 sequences for detailed investigation based on additional criteria of ‘genome complete’ and ‘human host group’. This comprehensive analysis encompassed sequences from various geographical regions, including Africa (27), Asia (9), Europe (28), North America (7), and South America (125), thus ensuring a globally representative and diverse dataset for our yellow fever virus research. To achieve our research objectives, the proteomes of these 196 viral strains were meticulously compiled, categorized by protein type, and systematically aligned to derive consensus sequences.

We conducted MHC class I restricted (CTL) epitope prediction using two online servers: ProPred I (http://crdd.osdd.net/raghava/propred1) and NetCTL (http://www.cbs.dtu.dk/services/NetCTL/). ProPred I identifies promiscuous regions in protein sequences by employing matrices for 47 MHC-I alleles and models for proteasomal and immunoproteasome processing^33^. The analysis was carried out with specific parameters, including a 4% threshold, and the activation of proteasome and immunoproteasome filters, each set at a threshold of 5%, as referenced in^34,35^.

For validation of the identified epitopes in ProPred, we utilized NetCTL. NetCTL not only predicts potential epitopes within protein sequences for cytotoxic T lymphocytes (CTL) but also provides information regarding proteasomal C-terminal cleavage through artificial neural networks and TAP transport efficiency through weight matrix calculations^36^. The thresholds for CTL epitope identification, C-terminal cleavage, and TAP transport efficiency were set at 0.75, 0.15, and 0.05, respectively.

Similarly, two distinct methods were employed to assess the HTL epitopes capable of binding to HLA-DQ, HLA-DP, and HLA-DR alleles, employing artificial neural networks. The IEDB tool (http://tools.iedb.org/mhcii/) utilizes a big dataset including over 10,000 unpublished MHC-peptide binding affinities, 29 peptide/MHC crystal structures, and 664 peptides that have been experimentally tested^29^. To validate the forecasts generated by IEDB, the protein sequences have been uploaded to the NetMHCIIpan database., which is accessible at http://www.cbs.dtu.dk/services/NetMHCIIpan/. This server utilizes a vast dataset that includes more than 100,000 quantitative measurements of peptide binding sourced from IEDB. It covers a wide range of molecules, including HLA-DR, HLA-DQ, HLA-DP and even mouse MHC-II molecules, namely 36 HLA-DR, 27 HLA-DQ, 9 HLA-DP and 8 mouse MHC-II molecules^38^.

#### Prediction of B‑cell epitopes

The “Bepipred Linear Epitope Prediction 2.0” method, available through the IEDB tool at http://tools.iedb.org/bcell/, was employed to enhance the accuracy of predicting B lymphocyte (BL) epitopes within protein sequences. "Prediction 2.0" was specifically developed to address limitations observed in other epitope prediction tools, which predict continuous epitopes through a random forest algorithm trained on epitopes annotated from antibody antigen protein structures^30^. The standard threshold of 0.5 for predictions was maintained.

#### Antigenicity prediction

The theoretical epitopes underwent an antigenicity evaluation step in which they were individually entered into VaxiJen 2.0 (http://www.ddg-pharmfac.net/vaxijen/VaxiJen/VaxiJen.html). This software assesses antigenicity, taking into account the selection of a target organism, such as a virus, bacterium, tumor, parasite, or fungus. In this analysis, a threshold of 0.5 was adopted, as this value aligns with the peak accuracy for most of the models used^31^.

#### Allergenicity prediction

The allergic or nonallergic nature of possible epitopes was predicted using the AllerTop 2.0 server (http://www.ddg-pharmfac.net/AllerTOP/). This online server evaluates the similarities between the peptide sequence under study and the sequences in its database. The epitopes are individually assessed, and the outcome, indicating whether the sequence is likely allergenic or non-allergenic, is furnished. Additionally, a link to the protein with a similar sequence is provided.

#### Immunogenicity prediction

Immunogenicity scores of CTL epitopes were calculated using IEDB immunogenicity (http://tools.iedb.org/ immunogenicity/). This tool collects the most important variables affecting immunogenicity, such as the P4-6 position of a peptide and amino acids with large and aromatic side chains^32^. The masking option used was the default (1, 2, and c-terminal) and the cutoff was set to zero^33^.

#### Toxicity prediction

To guarantee that the chosen epitopes were not toxic, the ToxinPred web server (http://www.imtech.res.in/raghava/toxinpred/) was used. To predict the toxicity of the epitopes, the server is based on the physicochemical characteristics of the peptides using machine learning and a quantitative matrix. The database of this method includes 1,805 toxic and 3,593 non-toxic peptides^34^.

#### Conservation analysis

To gauge the extent of epitope conservation within the acquired protein sequences at varying levels of identity, the IEDB conservation tool, accessible at http://tools.iedb.org/conservancy^44^, was utilized. The extent of conservation is measured by the percentage of protein sequences with which the epitope is identical at a given level of similarity. This approach allows for the selection of broadly protective epitopes.

### Population coverage analysis

Population coverage provides a direct indication of vaccine efficacy in different geographic regions by examining the prevalence of human leukocyte antigen (HLA) alleles associated with the epitope of interest. For this study, the selected epitopes, along with their respective HLA-binding alleles, were submitted to the IEDB population coverage tool (http://tools.iedb.org/conservancy)^35^. This tool was programmed for the principal endemic regions of yellow fever—South America and Africa.

### Multi–epitope vaccine construction

The vaccine sequence was constructed using LCTL and HTL epitopes with sequences overlapping with the BL epitopes that passed all immunoinformatic analyses. These sequences were connected using AAY and GPGPG linkers, respectively^36^. The AAY peptide linker helps the epitopes generate suitable sites for binding to the TAP transporter and enhances epitope presentation, whereas the GPGPG linker stimulates TCD4 + responses and preserves the conformational immunogenicity of the helpers as well as the antibody epitopes^37^.

A $\beta$-defensin adjuvant sequence was added to the N-terminus of the multi-epitope vaccine using the linker EAAAK, thus enhancing its immunogenicity. The $\beta$-defensin induces recruitment of naive T cells and immature dendritic cells by contacting TLR and CCR 6 (chemokine receptor 6) receptors at the site of infection^38^, and the linker EAAAK reduces association with other protein domains with efficient detachment and increases stability^39^.

### Assessment physicochemical properties of the vaccine prototype

To analyze the physicochemical characteristics of the vaccine model, such as: To obtain information on molecular weight, pI (isoelectric point), half-life, instability index, aliphatic index, and GRAVY (Grand Average Hydropathy) of the vaccine sequence, it was subjected to analysis using the ExPASy-ProtParam tool, available at http://web.expasy.org/protparam/. These analyses leveraged a database of proteins with well-established properties, which were used as reference parameters for the provided protein sequence^40^.

### Design, refinement and validation of the tertiary structure of the vaccine prototype

The Raptor-X server, accessible at http://raptorx.uchicago.edu/, was used to predict the three-dimensional structure of the vaccine sequence. This procedure consists of taking an input sequence in FASTA format and implementing three techniques: single and multiple template threading, along with prediction of alignment quality^7^. To gauge the precision of the projected 3D structure, the online website is the most trusted sources, which encompass the *P*-value for relative global quality, as well as GDT (Global Distance Test) and uGDT (un-normalized GDT) for assessing the overall structural integrity^52^.

The refinement of the tertiary structure was carried out through the GalaxyRefine 2 server, which is available at http://galaxy.seoklab.org/cgi-bin/submit.cgi?type=REFINE2. This web server employs a specialized approach for refining 3D structures by implementing short Molecular Dynamics simulations after repeated perturbations involving side-chain repacking at both global and local levels. This method enables more extensive structural adjustments^53^. It incorporates multiple local and global move sets and iteratively accumulates conformational changes, facilitating larger-scale modifications. The local and global move sets use an estimated structure error to concentrate refinement efforts in regions with greater inaccuracies.

Finally, the quality and potential errors in the 3D model were checked using MolProbity, Swiss Model, ProSA-web, ERRAT, and Verify3D. For structural validation, the Swiss Model's Structure Assessment Tool, available at https://swissmodel.expasy.org/assess, was employed to obtain information on both the global and local aspects of the structure. This tool utilizes its own methods, including QMEAN and Ramachandran plot analysis, and can also run additional software tools like MolProbity. In addition, the ProSA web server, accessible at https://prosa.services.come.sbg.ac.at/prosa.php, was included in the analysis to validate the structural quality, and a quality score (Z-score) for the input structure was calculated. Score values that fall outside a typical range for native proteins suggest that there are likely errors in the structure^41^. Another validation server, known as ERRAT (http://services.mbi.ucla.edu/ERRAT/)^42^, assessed disjoint interactions within the framework. The accuracy of the 3D model's design was assessed using Verify3D, available at https://servicesn.mbi.ucla.edu/Verify3D/. This tool gauges the compatibility of the model with its corresponding amino acid sequence, providing insights into the quality and reliability of the structural model^43^.

### Molecular docking simulations and refinement

Binding of the vaccine to the appropriate immunological recipient is paramount to elicit an appropriate immune response^44,45^. Toll-like receptors (TLR) are members of a family of pattern recognition receptors that recognize products of various microorganisms^46^. In the recognition of YFV by the host immune system, TLR-2 along with three other Toll-like receptors (7, 8, and 9) are described to be crucial for the interactions between 17D vaccine and human cells stimulating a mixed Th2 and Th1 cell profile^47^.

Therefore, the vaccinal model was linked to the TLR-2 receptor (PDB ID: 2z7x) using PatchDock server (http://bioinfo3d.cs.tau.ac.il/PatchDock/). PatchDock^48^ is a geometry-based molecular docking algorithm. This program, upon inputting two molecules, segments them into patches based on their surface shape, effectively dividing them into patterns akin to visually distinguishable puzzle pieces. The algorithm entails several key steps: (a) the representation of molecular shape, (b) matching surface patches, (c) filtering and evaluation. These patches can be overlaid using shape matching algorithms to facilitate the comparison and analysis of the two molecular structures^49^.

The FireDock web tool (http://bioinfo3d.cs.tau.ac.il/FireDock/)^50^ was used to optimize and re-evaluate the rigid-body molecular docking solutions. The final 10 models are categorized based on a general energy that includes atomic contact energy as well as van der Waals interaction, partial electrostatics, and binding energy estimates. The most promising Firedock model underwent further refinement within the HADDOCK interface, which can be accessed at https://bianca.science.uu.nl/haddock2.4/refinement/1. This server offers a list of clusters ranked by score and provides comprehensive statistics, including the average score for the top four structures within each cluster. This step allows for a more in-depth analysis and selection of refined structures based on their quality and score^51^.

Molecular dynamics simulation (MD) is an important technique for analyzing the strength of the receptor-ligand complex. It was used with the WEBGRO for Macromolecular Simulations (https://simlab.uams.edu/) to investigate the binding stability of the final complex^52^. In the simulation of the TLR2-vaccine complex, a 50 ns timeframe was employed. The GROMOS96 43a1 force field parameters were used for the simulation. The entire system was solvated in water, neutralized to balance the charges, and supplemented with 0.15 M NaCl salt to mimic physiological conditions. Key simulation parameters monitored during this process included the Root Mean Square Deviation (RMSD) and the Root Mean Square Fluctuation (RMSF). These parameters are fundamental for assessing the stability and dynamics of the system throughout the simulation.

To identify the most pertinent vaccine-TLR2 complex resulting from the docking calculations, we employed the combined quantum mechanics/molecular mechanics technique (QM/MM). This approach combines quantum mechanics for the ligand-receptor interactions with molecular mechanics for the surrounding environment, allowing for a more accurate representation of the system's behavior. QM/MM methods have solidified their position as advanced computational tools for studying biomolecular systems, as evidenced by the increasing number of publications utilizing these methods^20,26,53,54^. this procedure consists of taking an input sequence in FASTA format and implementing three techniques: single and multiple template threading, along with prediction of alignment quality^7^. To gauge the precision of the projected 3D structure, the server offers confidence scores, which encompass the *P*-value for relative global quality, as well as GDT (Global Distance Test) and uGDT (un-normalized GDT) for assessing the overall structural integrity^52^. The ONIOM multilayer technique, a unified strategy accessible in the Gaussian code, was used to carry out the QM/MM optimization. Ab initio calculations of the total energy of large complexes, such as biological systems, are possible and accurate using this approach when the systems have been divided into two or three layers. The TLR-2 receptor was placed in the MM layer, while the vaccine's main residues of amino acids were assigned to the QM layer.

To enlarge the electronic orbitals in the QM layer, we used the well-known B3LYP hybrid functional (Becke, three parameters, Lee–Yang–Parr) for exchange–correlation in conjunction with the 6-311G (d, p) basis set. Notably, during the geometry optimization process, all amino acid residues within a 6.0 Å radius from the ligand's centroid were allowed to adjust their positions^55–57^. This approach facilitates the accurate exploration of the ligand-receptor interactions and structural changes within the specified region of the complex ^58^.

The best PatchDock + FireDock + HADDOCK (PFH) and PatchDock + FireDock + HADDOCK + MD + QM/MM (PFHMQM) models were operated in the PRODIGY prediction for a comparative analysis of binding energies. The PRODIGY forecast protein–protein binding affinity (or binding free energy) on the basis of the biological system's structure and function, i.e., the interfacial contact network^59^. RMSD analysis in Discovery studio compared structures to original PatchDock complexes, revealing structural disparities^60^.

**Figure 2:**
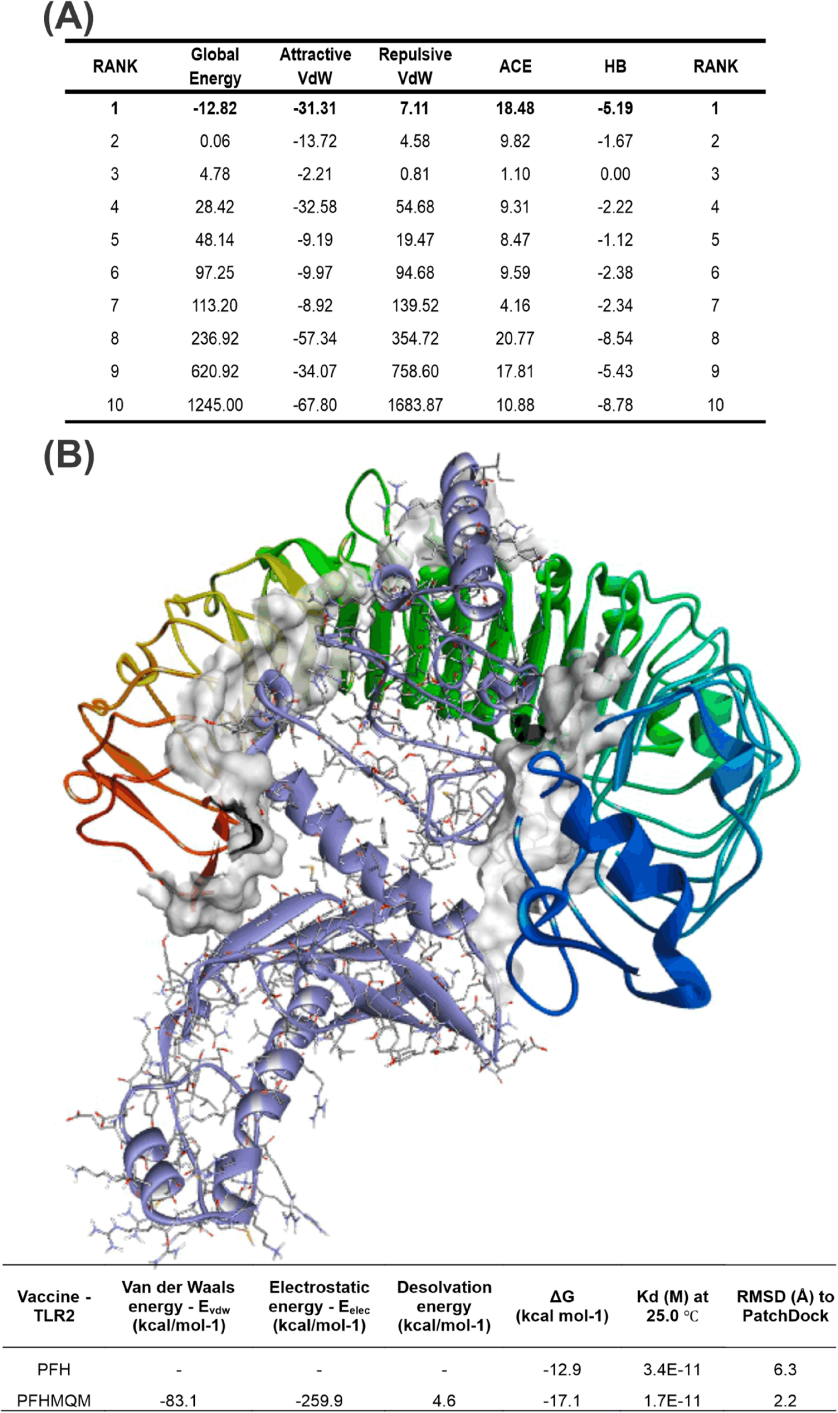
Molecular docking and refinements. (**A**) Energies values obtained after refinement by FireDock. (**B**) Molecular Docking and Refinements. (**A**) Energy values obtained after refinement by FireDock. (**B**) Best 3D docking model acquired after docking in PatchDock and subsequent refinements in FireDock, HADDOCK, MD, and QM/MM (structure PFHMQM). At the bottom of the image, energetic scores of the structures following sequential stages of geometric refinement.

Finally, Discovery Studio Visualizer, LigPlot + (https://www.ebi.ac.uk/thornton-srv/software/LigPlus/), and Pose View (https://proteins.Plus/) were implemented to evaluate binding postures and the existence of intermolecular interactions, in particular intermolecular hydrogen bonds (Fig. 2a) (carbon, conventional, and pi-donor H-bonds), electrostatic (Fig. 2b) (salt bridge, attractive charges, pi-cation, pi-anion), hydrophobic (Fig. 2c) (pi-pi stacked, pi-pi stacked, alkyl, pi-sigma, pi-alkyl), halogens (Cl, fluorine, Br, and I), miscellaneous (charge repulsion, steric unevenness, acceptor-acceptor collision.

### Codon adaptation and in silico cloning

Codon adaptation according to the host microorganism to be used is a very important step for in silico cloning. For this purpose, we used the Java Codon Adaptation Tool (http://www.jcat.de/), which specializes in predicting an optimized coding sequence for each input sequence (DNA or protein). Its result output includes the optimized gene sequence along with its codon adaptive index (CAI) and the percentage of CG content^61^. In this step, *E. coli* k12 was considered, which is widely used as a host microorganism. In addition, three criteria were selected (a) avoidance of rho-independent transcription terminators, (b) avoidance of prokaryotic ribosome binding sites, (c) avoidance of restriction enzyme cleavage sites. This aligned sequence was inverted using the IUPAC convention (https://arep.med.harvard.edu/labgc/adnan/projects/Utilities/revcomp.html) to show complementarity with the replication cycle of its vector. The restriction sites *XhoI* and *BamHI* were added to the N-terminus and C-terminus of the optimized reverse cDNA sequence. This resulting sequence was inserted into the pET-28a( +) vector using SnapGene v4.2 software (http://www.snapgene.com)^62^ for subsequent in silico cloning.

### Immune response simulation

The immunogenicity and immune response of the vaccine construct were assessed using the C-ImmSim service (https://kraken.iac.rm.cnr.it/C-IMMSIM/), which combines molecular biology approaches with data-driven prediction methods to provide a comprehensive profile^63^. The program was adjusted so that the period between injection doses is approximately one month, which equates to (84 time steps), and the simulation steps were set to one thousand, while all other stimulation parameters were left at their default values.

## Results and discussion

Vaccines are the best strategy to prevent infectious diseases by generating protective immunity. Conventional vaccines are used worldwide and are considered the best method for treating various diseases. However, new vaccination tactics are required immediately to address the problems associated with live or attenuated vaccines (see our introductory section). For example, multiepitope-based vaccines produced by reverse vaccinology techniques are harmless, more stable, and easier to produce than attenuated viral vaccines. In addition, they are recommended primarily for their cost-effectiveness and higher efficacy^64,65^.

The importance of computational methods in the development of these vaccines is growing. Current approaches in molecular modeling, bioinformatics, and immunoinformatic have accelerated the production process and enabled screening of genomes to identify potential vaccine candidates and develop multiepitope vaccines with higher efficacy. This technology has evolved to identify viral proteome areas that are potentially capable of activating innate and adaptive immune responses to induce protective memory. Therefore, it has been used in several studies with pathogenic microorganisms such as SARS-CoV-2^20^ and other arboviruses such as dengue virus^44^, Burkholderia^64^, Chikungunya virus^24^, and Mayaro virus^25,26^. Recent studies in animal models have shown excellent results for multiepitope vaccines, suggesting that this platform is a promising and safe method compared with attenuated vaccines^66^. Interestingly, the first candidate vaccine against malaria to progress to phase III clinical trials is the MosquirixTM, which comprises contiguous epitopes derived from the circumsporozoite protein of *Plasmodium falciparum*^67^.

In the present research, we proposed a multi-epitope vaccine against Yellow fever virus based on a robust methodology. The results will be presented and discussed below.

### Acquisition of viral protein sequences

In our quest to explore antigenic epitopes for the development of an effective Yellow Fever Virus (YFV) vaccine, we conducted a thorough examination of validated data from the ViPR database. This investigation yielded a total of 5, 5, 3, 9, 10, 10, 6, 10, 7, 5, and 4 protein sequences for the proteins E, C, M, NS1, NS2A, NS2B, NS3, NS4A, NS4B, and NS5, respectively, within the YFV. All these proteins were taken from a total of 196 complete genomes, included sequences from different geographical regions such as Africa (27), Asia (9), Europe (28), North America (7) and South America (125). The consensus sequences of these proteins were used to predict the B and T cell epitopes for the design of the multi-epitope vaccine.

### CTL epitope selection

Combining Propred and NetCTL data, 110 epitopes with binding affinity to MHC class I were identified. However, only 10,$$({M}^{60-68}$$, $$NS2{A}^{105-113}$$,$$NS2{B}^{5-13}$$, $$NS2{B}^{8-16}$$, $$NS2{B}^{9-17}$$, $$NS2{B}^{79-87}$$, $$NS{3}^{123-131}$$, $$NS4{A}^{106-114}$$, $$NS4{B}^{199-207}$$ and $$NS{3}^{144-152})$$, had a binding affinity of $$\ge$$ 5 alleles, obtained relevant results in antigenicity, allergenicity, immunogenicity, non-toxicity, and had a maintenance rate of $$\ge$$ 90% (Tables 1, 2, 3, 4, 5, 6, 7, 8, 9, 10 and 11). One by one, we will present and analyze the results for each YFV protein.

**Table 1:** List of predicted MHC-I (CTL) epitopes on YVF proteins, with the sequence of each peptide and its allele number, antigenicity score, allergenicity, immunogenicity, toxicity, and conservation.

Protein	Peptide sequence	Propred I		NetCTL		VaxiJen	Allertop	IEDB	ToxinPred	IEDB
		Alleles	Supertypes	Binding affinity	Prediction score	Antigenicity	Allergenicity	Immunogenicity	Toxicity	Conservancy
M	60-RVVIALLVL	14	3	0.5920	13.513	0.6326	PROBABLE NON-ALLERGEN	0.18974	Non-Toxin	100.00% (3/3)
NS2A	105-TLGAAMVEI	7	1	0.5693	0.9863	11.467	PROBABLE NON-ALLERGEN	0.01651	Non-Toxin	90.00% (9/10)
NS2B	5-NEALAAAGL	7	1	0.5745	15.325	0.5348	PROBABLE NON-ALLERGEN	0.12929	Non-Toxin	100.00% (6/6)
	8-LAAAGLVGV	7	1	0.4588	0.7671	12.057	PROBABLE NON-ALLERGEN	0.12927	Non-Toxin	100.00% (6/6)
	9-AAAGLVGVL	16	1	0.3389	0.8325	0.6883	PROBABLE NON-ALLERGEN	0.12758	Non-Toxin	100.00% (6/6)
	79-SEQGEFKLL	9	1	0.6436	17.793	0.5125	PROBABLE NON-ALLERGEN	0.01572	Non-Toxin	100.00% (6/6)
NS3	123-GGEIGAVAL	8	1	0.1885	0.7702	16.896	PROBABLE NON-ALLERGEN	0.29395	Non-Toxin	100.00% (10/10)
	144-NRNGEVIGL	5	2	0.4367	13.586	0.9863	PROBABLE NON-ALLERGEN	0.30048	Non-Toxin	100.00% (10/10)
NS4A	106-YIMLIFFVL	17	4	0.7908	13.786	0.6225	PROBABLE NON-ALLERGEN	0.29456	Non-Toxin	100.00% (7/7)
NS4B	199-LASAALGPL	12	1	0.4779	10.447	10.003	PROBABLE NON-ALLERGEN	0.03545	Non-Toxin	100.00% (5/5)

**Table 2:** List of predicted MHC-I (CTL) epitopes on YVF E protein, with the sequence of each peptide and its allele number, antigenicity score, allergenicity, immunogenicity, toxicity, and conservation.

Protein	Peptide sequence	Propred I		NetCTL		VaxiJen	Allertop	IEDB	ToxinPred	IEDB
		Alleles	Supertypes	Binding affinity	Prediction score	Antigenicity	Allergenicity	Immunogenicity	Toxicity	Conservancy
E	239-AATIKVLAL	12	1	0.5194	1.1980	0.7571	PROBABLE NON-ALLERGEN			
	284-RVKLSALTL	7	3	0.5967	1.3614	1.1359	PROBABLE NON-ALLERGEN			
	234-FEPPHAATI	12	1	0.4399	1.2170	0.6489	PROBABLE ALLERGEN			
	33-VMAPDKPSL	12	3	0.6088	1.1104	0.1240				
	74-CPSTGEAHL	16	1	0.3824	0.9197	0.4244				
	250-QEGSLKTAL	8	1	0.5141	1.3861	− 0.2428				
	328-APCRIPVMV	14	1	0.4988	1.1071	0.0193				
	332-IPVMVADDL	20	1	0.5101	1.1539	0.3177				
	453-TKVIMGVVL	6	1	0.3761	1.2786	0.0979				
	40-SLDISLETV	2	2	0.1441	0.7609					
	265-TKDTNNSKL	4	1	0.3457	1.2941					
	237-PHAATIKVL	2	1	0.4719	1.6731					
	334-VMVADDLTA	4	1	0.5217	0.8061

**Table 3:** List of predicted MHC-I (CTL) epitopes on YVF M protein, with the sequence of each peptide and its allele number, antigenicity score, allergenicity, immunogenicity, toxicity, and conservation.

Protein	Peptide sequence	Propred I		NetCTL		VaxiJen	Allertop	IEDB	ToxinPred	IEDB
		Alleles	Supertypes	Binding affinity	Prediction score	Antigenicity	Allergenicity	Immunogenicity	Toxicity	Conservancy
M	60-RVVIALLVL	14	3	0.5920	13.513	0.6326	PROBABLE NON-ALLERGEN	0.18974	Non-Toxin	100.00% (3/3)
	39-NPFFAVTAL	23	1	0.6816	15.079	12.672	PROBABLE NON-ALLERGEN	0.28838	Non-Toxin	66.67% (2/3)
	41-FFAVTALAI	8	2	0.4708	10.398	12.651	PROBABLE NON-ALLERGEN	0.14237	Non-Toxin	33.33% (1/3)
	57-MTQRVVIAL	11	3	0.3956	15.239	0.5028	PROBABLE ALLERGEN

**Table 4:** List of predicted MHC-I (CTL) epitopes on YVF C protein, with the sequence of each peptide and its allele number, antigenicity score, allergenicity, immunogenicity, toxicity, and conservation.

Protein	Peptide sequence	Propred I		NetCTL		VaxiJen	Allertop	IEDB	ToxinPred	IEDB
		Alleles	Supertypes	Binding affinity	Prediction score	Antigenicity	Allergenicity	Immunogenicity	Toxicity	Conservancy
C	70-RMLDPRQGL	18	3	0.4448	0.8571	13.585	PROBABLE NON-ALLERGEN	− 0.02132		
	73-DPRQGLAVL	14	2	0.6231	13.682	0.4958				
	80-VLKKVKRVV	7	1	0.4249	15.860	0.3180				
	83-KVKRVVASL	9	3	0.4601	10.986	− 0.1043				
	3-GRKAQGKTL	2	1	0.3322	10.481					
	74-PRQGLAVLK	1	1	0.3414	0.9959					
	77-GLAVLKKVK	4	1	0.4007	0.7992

**Table 5:** List of predicted MHC-I (CTL) epitopes on YVF NS1 protein, with the sequence of each peptide and its allele number, antigenicity score, allergenicity, immunogenicity, toxicity, and conservation.

Protein	Peptide sequence	Propred I	NetCTL	VaxiJen	Allertop	IEDB	ToxinPred	IEDB	ToxinPred	IEDB
		Alleles	Supertypes	Binding affinity	Prediction score	Antigenicity	Allergenicity	Immunogenicity	Toxicity	Conservancy
NS1	34-YYPEDPVKL	11	2	0.6325	15.513	0.2597				
	176-TMDCDGSIL	10	2	0.1459	0.7827	− 0.6660

**Table 6:** List of predicted MHC-I (CTL) epitopes on YVF NS2A protein, with the sequence of each peptide and its allele number, antigenicity score, allergenicity, immunogenicity, toxicity, and conservation.

Protein	Peptide sequence	Propred I	NetCTL	VaxiJen	Allertop	IEDB	ToxinPred	IEDB	ToxinPred	IEDB
		Alleles	Supertypes	Binding affinity	Prediction score	Antigenicity	Allergenicity	Immunogenicity	Toxicity	Conservancy
NS2A	105-TLGAAMVEI	7	1	0.5693	0.9863	1.1467	PROBABLE NON-ALLERGEN	0.01651	Non-Toxin	90.00% (9/10)
	13-MMIAMEVVL	15	5	0.6326	1.1457	0.5970	PROBABLE NON-ALLERGEN	0.06478	Non-Toxin	80.00% (8/10)
	40-AMLVGQVTI	13	1	0.5446	0.9867	0.8320	PROBABLE NON-ALLERGEN	0.01942	Non-Toxin	60.00% (6/10)
	34-GMVLLGAML	16	1	0.3826	0.8618	0.5315	PROBABLE NON-ALLERGEN	− 0.04624		
	44-GQVTILDLL	18	1	0.3058	0.8864	1.0725	PROBABLE ALLERGEN			
	123-WKYLNAVSL	11	2	0.2390	0.8250	0.9377	PROBABLE ALLERGEN			
	195-VALTLTSYL	23	1	0.4149	0.8190	0.8042	PROBABLE ALLERGEN			
	23-KRQGPKQIL	6	2	0.4317	1.3486	− 0.6389				
	29-QILVGGMVL	9	3	0.1908	0.8475	0.4287				
	30-ILVGGMVLL	19	1	0.5679	1.0425	0.2574				
	41-MLVGQVTIL	18	1	0.5668	1.0532	0.4934				
	48-ILDLLKLTV	8	1	0.5170	0.9109	0.3765				
	94-TLWSPRERL	13	1	0.3809	0.7759	0.4337				
	118-MMGGLWKYL	9	1	0.3072	0.7510	− 0.5650				
	161-AEVRLATML	9	1	0.6177	1.6774	0.4177				
	98-PRERLVLTL	3	2	0.2218	0.7643					
	135- TINAVASRK	2	1	0.7592	1.5524					
	142-SRKASNVIL	2	2	0.3253	1.0692					
	144-ASNVILPLM	4	1	0.2935	0.7547

**Table 7:** List of predicted MHC-I (CTL) epitopes on YVF NS2B protein, with the sequence of each peptide and its allele number, antigenicity, score, allergenicity, immunogenicity, toxicity, and conservation.

Protein	Peptide sequence	Propred I		NetCTL		VaxiJen	Allertop	IEDB	ToxinPred	IEDB
		Alleles	Supertypes	Binding affinity	Prediction score	Antigenicity	Allergenicity	Immunogenicity	Toxicity	Conservancy
NS2B	5-NEALAAAGL	7	1	0.5745	15.325	0.5348	PROBABLE NON-ALLERGEN	0.12929	Non-Toxin	100.00% (6/6)
	8-LAAAGLVGV	7	1	0.4588	0.7671	12.057	PROBABLE NON-ALLERGEN	0.12927	Non-Toxin	100.00% (6/6)
	9-AAAGLVGVL	16	1	0.3389	0.8325	0.6883	PROBABLE NON-ALLERGEN	0.12758	Non-Toxin	100.00% (6/6)
	79-SEQGEFKLL	9	1	0.6436	17.793	0.5125	PROBABLE NON-ALLERGEN	0.01572	Non-Toxin	100.00% (6/6)
	30-GPVAVGGIL	21	1	0.6662	13.958	0.8093	PROBABLE NON-ALLERGEN	0.23123	Non-Toxin	83.33% (5/6)
	108-AIHPFALLL	11	1	0.4743	0.9208	0.6095	PROBABLE NON-ALLERGEN	0.13433	Non-Toxin	50.00% (3/6)
	106-GAAIHPFAL	7	3	0.3785	0.8832	0.6859	PROBABLE NON-ALLERGEN	0.28934	Non-Toxin	83.33% (5/6)
	110-HPFALLLVL	25	3	0.4841	11.140	0.9569	PROBABLE NON-ALLERGEN	0.07089	Non-Toxin	66.67% (4/6)
	96-QVVMTSLAL	7	2	0.4927	11.301	10.096	PROBABLE ALLERGEN			
	2-IPVNEALAA	10	1	0.6182	11.985	0.2934				
	12-GLVGVLAGL	19	1	0.6187	11.192	0.3833				
	43-SVAGRVDGL	11	1	0.2834	0.9631	− 0.2971				
	94-WDQVVMTSL	11	2	0.2828	11.017	0.4259				
	104 -LVGAAIHPF	5	2	0.3059	0.9402	0.4900				
	72-ARYDVALSE	2	1	0.3523	0.9317

**Table 8:** List of predicted MHC-I (CTL) epitopes on YVF NS3 protein, with the sequence of each peptide and its allele number, antigenicity score, allergenicity, immunogenicity, toxicity, and conservation.

Protein	Peptide sequence	Propred I		NetCTL		VaxiJen	Allertop	IEDB	ToxinPred	IEDB
		Alleles	Supertypes	Binding affinity	Prediction score	Antigenicity	Allergenicity	Immunogenicity	Toxicity	Conservancy
**NS3**	123-GGEIGAVAL	8	1	0.1885	0.7702	16.896	PROBABLE NON-ALLERGEN	0.29395	Non-Toxin	100.00% (10/10)
	144-NRNGEVIGL	5	2	0.4367	13.586	0.9863	PROBABLE NON-ALLERGEN	0.30048	Non-Toxin	100.00% (10/10)
	374-NVMAASLRK	5	1	0.5892	12.453	0.5397	PROBABLE NON-ALLERGEN	− 0.11438		
	430-CRTAFKPVL	8	1	0.2747	10.454	0.6957	PROBABLE NON-ALLERGEN	− 0.02227		
	225-VLAPTRVVL	12	5	0.3638	14.408	0.8747	PROBABLE ALLERGEN			
	403-KQKKPDFIL	11	2	0.4473	10.823	18.986	PROBABLE ALLERGEN			
	70-SWASVKEDL	5	1	0.3225	0.8020	0.0638				
	218-RRRLRTLVL	7	4	0.5573	21.275	0.1166				
	372-AANVMAASL	13	1	0.4466	10.607	0.2921				
	386-IIMDEAHFL	14	1	0.7348	12.834	0.4284				
	443-RKVAIKGPL	7	1	0.3917	12.283	− 0.1085				
	604-RVSSDQSAL	8	2	0.6800	14.505	0.0011				
	67-LVPSWASVK	3	1	0.5757	12.017

**Table 9:** List of predicted MHC-I (CTL) epitopes on YVF NS4A protein, with the sequence of each peptide and its allele number, antigenicity score, allergenicity, immunogenicity, toxicity, and conservation.

Protein	Peptide sequence	Propred I		NetCTL		VaxiJen	Allertop	IEDB	ToxinPred	IEDB
		Alleles	Supertypes	Binding affinity	Prediction score	Antigenicity	Allergenicity	Immunogenicity	Toxicity	Conservancy
**NS4A**	106-YIMLIFFVL	17	4	0.7908	13.786	0.6225	PROBABLE NON-ALLERGEN	0.29456	Non-Toxin	100.00% (7/7)
	4- EVLVVLSEL	13	1	0.2639	0.8703	0.5835	PROBABLE NON-ALLERGEN	− 0.01342		
	1-GAAEVLVVL	18	1	0.2574	10.122	0.2977				
	8-VLSELPDFL	16	1	0.6341	11.272	0.2109				
	23-AVDTISVFL	10	2	0.1579	0.8611	− 0.3616				
	51-MTTVMLFVL	8	2	0.3881	0.7702	0.1512				
	48-PEAMTTVML	4	1	0.4624	12.423

**Table 10:** List of predicted MHC-I (CTL) epitopes on YVF NS4B protein, with the sequence of each peptide and its allele number, antigenicity score, allergenicity, immunogenicity, toxicity, and conservation.

Protein	Peptide sequence	Propred I		NetCTL		VaxiJen	Allertop	IEDB	ToxinPred	IEDB
		Alleles	Supertypes	Binding affinity	Prediction score	Antigenicity	Allergenicity	Immunogenicity	Toxicity	Conservancy
**NS4B**	199-LASAALGPL	12	1	0.4779	10.447	10.003	PROBABLE NON-ALLERGEN	0.03545	Non-Toxin	100.00% (5/5)
	163-MPALYEKKL	14	2	0.5710	12.709	13.793	PROBABLE NON-ALLERGEN	− 0.21581		
	165-ALYEKKLAL	15	5	0.3219	12.984	0.9847	PROBABLE NON-ALLERGEN	− 0.29995		
	171-LALYLLLAL	24	3	0.2272	0.9431	0.6164	PROBABLE NON-ALLERGEN	− 0.01506		
	173-LYLLLALSL	10	1	0.6386	15.478	10.201	PROBABLE NON-ALLERGEN	− 0.09475		
	196-GIVLASAAL	6	1	0.3135	0.7804	0.5756	PROBABLE NON-ALLERGEN	− 0.05951		
	167-YEKKLALYL	12	3	0.6220	17.162	0.5543	PROBABLE ALLERGEN			
	169-KKLALYLLL	9	1	0.3684	11.658	0.2525				
	185-AMCRTPFSL	11	3	0.4768	0.9062	0.3537				
	191-FSLAEGIVL	16	2	0.3219	11.927	0.1835				
	21-PSSAAPWSW	1	1	0.7443	17.885

**Table 11:** List of predicted MHC-I (CTL) epitopes on YVF NS5 protein, with the sequence of each peptide and its allele number, antigenicity score, allergenicity, immunogenicity, toxicity, and conservation.

Protein	Peptide sequence	Propred I		NetCTL		VaxiJen	Allertop	IEDB	ToxinPred	IEDB
		Alleles	Supertypes	Binding affinity	Prediction score	Antigenicity	Allergenicity	Immunogenicity	Toxicity	Conservancy
NS5	176-VESFCVKVL	8	1	0.4388	12.323	0.6768	PROBABLE NON-ALLERGEN	− 0.10742		
	445-RRPTGKVTL	7	2	0.5379	16.478	0.6234	PROBABLE NON-ALLERGEN	− 0.07702		
	455-MMGKREKKL	8	1	0.2460	10.105	21.398	PROBABLE NON-ALLERGEN	− 0.36935		
	773-KRDMRLLSL	8	2	0.4580	14.159	16.470	PROBABLE NON-ALLERGEN	− 0.23556		
	477-YMWLGARYL	13	3	0.4869	11.708	0.5589	PROBABLE ALLERGEN			
	482-ARYLEFEAL	8	2	0.4440	16.326	14.394	PROBABLE ALLERGEN			
	672-RPIDDRFGL	20	1	0.6747	14.977	12.836	PROBABLE ALLERGEN			
	771-FHKRDMRLL	7	2	0.4739	16.465	12.584	PROBABLE ALLERGEN			
	65-FHERGYVKL	8	2	0.7907	27.094	0.1434				
	95-KEVSGVKGF	9	1	0.3795	12.017	0.1382				
	97-VSGVKGFTL	10	1	0.2935	0.8404	0.3110				
	187-YMPDVLEKL	23	3	0.8442	14.498	0.1060				
	265-SVETDKGPL	6	1	0.3011	0.7665	0.3211				
	524-AALEGGGFY	6	3	0.3778	13.049	0.0086				
	574-YKNKVVKVL	8	2	0.4485	16.253	− 0.8500				
	40-ARRHLAEGK	2	1	0.2767	0.7995					
	41-RRHLAEGKV	4	1	0.4182	12.559					
	674-IDDRFGLAL	4	1	0.3886	13.569					
	714-SHHFHELQL	4	1	0.3418	12.884					

Some studies concluded that the envelope protein is critical for eliciting a strong humoral and cellular adaptative immune response against YFV^68,69^. Analysis of most antigenic regions of this protein revealed a total of 13 epitopes with possible affinity for MHC-I, but epitopes ${E}^{40-48}$, ${E}^{237-245}$, ${E}^{265-273}$, and ${E}^{334-342}$ did not have binding affinity to at least 5 HLA alleles, so they were discarded from our analysis. Of the remaining 9 epitopes, 6 had no antigenicity values above 0.5, and ${E}^{234-242}$ was classified as a probable allergen. ${E}^{239-247}$ and ${E}^{284-292}$ had negative immunogenicity values and were also discarded (Table 2).

Melo et al. (2013)^70^ provided six epitopes of the YFV envelope protein that elicited both CD4 + and CD8 + T cells, namely $${E}^{57-71}$$, $${E}^{65-79}$$, $${E}^{72-87}$$, $${E}^{337-351}$$, $${E}^{345-359}$$, and $${E}^{361-375}$$^70^. Milton et al.^71^ showed that $${E}^{57-71}$$ and $${E}^{329-343}$$ ($${E}^{57-71}$$, $${E}^{61-75}$$ , $${E}^{129-145}$$ and $${E}^{135-147)}$$ generate the highest CD8 + (CD4 +) T cell responses in mice^71^. Recently, Hassan et al.^72^ found that $${E}^{471-479}$$, $${E}^{363-371}$$, and $${E}^{226-234}$$ ($${E}^{284-292}$$ and $${E}^{479-487}$$) interact only with MHC-I and MHC-II alleles with extensive population coverage^72^.

As we saw here, none of these peptides matched our predicted peptides. Using a more robust and sophisticated methodology, we excluded ${E}^{72-87}$^70,71^${E}^{329-343}$, and ${E}^{337-351}$^70^ epitopes because they had very low antigenicity values^31^. In addition, we discarded ${E}^{284-292}$^72^ because it had an immunogenicity score (i.e., a T-cell recognition score) of -0.19211, indicating that the peptide-MHC-I complex formed by this epitope is theoretically not immunogenic in humans^32^. The selection of such epitopes (and many others) for the construction of a vaccine would not confer immunogenic power to it, which could logically be confirmed only by experimental testing.

It is essential to stress that we configure and parameterize the tools we use for highly sensitive analysis. Although we found a lower number of epitopes than other authors, we guarantee high confidence in the regions of the YFV proteome selected for the composition of our vaccine prototype by avoiding false positives.

The promiscuous epitope ${M}^{57-65}$, which belonged to 11 HLA class I alleles, had an antigenicity of 0.5028 but was noted as a possible allergen in the allergenicity analysis. ${M}^{39-47}$, ${M}^{41-49}$ and ${M}^{60-68}$ had satisfactory results in all analyses. However, only ${M}^{60-68}$, which was predicted for 14 alleles, was conserved in all M protein sequences (Table 3).

As happened with protein E, the epitopes of protein C did not pass through the analyses to which they were subjected, ${C}^{3-11}$, ^74-82^ ${C}^{77-85}$, not associated with the number of alleles required. Epitopes ${C}^{73-81}$, ${C}^{80-88}$, ${C}^{83-91}$ obtained antigenicity values below 0.5, remaining only ${C}^{70-78}$, which presented as a possible allergen (Table 4).

Only 2 epitopes were identified in the NS1 protein, $NS{1}^{34-42}$ and $NS{1}^{176-184}$, which were associated with 11 and 12 alleles, respectively, but the antigenicity values were less than 0.5, suggesting nonantigenic sequences, so they were not considered for further testing (Table 5).

The NS2A protein had a total of 19 epitopes (Table 6). $NS2{A}^{98-106}$, $NS2{A}^{135-143}$, $NS2{A}^{142-151}$ and $NS2{A}^{144-143}$ did not have the required number of alleles. Epitopes $NS2{A}^{44-52}$, $NS2{A}^{123-132}$ and $NS2{A}^{195-203}$ had significant scores of 0.8864, 0.8250, and 0.819, respectively, but were classified as allergenic molecules. $NS2{A}^{34-42}$ had a score of 0.8618 and was not allergenic, but had an immunogenic score of -0.04624, indicating non-immunogenic sequences. Epitopes $NS2{A}^{13-21}$, $NS2{A}^{40-48}$ and $NS2{A}^{105-113}$, which were predicted to bind to 15, 13, and 7 alleles, respectively, performed well in terms of antigenicity, allergenicity, immunogenicity, and toxicity, but only $NS2{A}^{105-113}$ was conserved in 90% of the sequences (Table 6).

Of the 15 epitopes of the NS2B protein that demonstrated affinity for the MHC I molecule, epitope $NS2{B}^{72-80}$ failed to bind to $\ge$ 5 alleles, and subsequently $NS2{B}^{96-104}$ failed the allergenicity test. The remaining epitopes passed the other analyses, but only $NS2{B}^{5-13}$, $NS2{B}^{8-16}$, $NS2{B}^{9-17}$ and $NS2{B}^{79-87}$ had sequences conserved in all NS2B sequences used (Table 7).

The NS3 protein was able to preserve the $NS{3}^{123-131}$ and $NS{3}^{144-152}$ epitopes throughout the analysis. The promiscuous epitope $NS{3}^{67-75}$ was predicted to bind only 3 HLA. $NS{3}^{225-233}$ and $NS{3}^{403-411}$ had significant antigenicity values but were disregarded for further analysis because they were considered allergenic. The immunogenicity values of epitopes $NS{3}^{374-382}$ and $NS{3}^{430-438}$ were negative and therefore had to be discarded (Table 8).

Table 9 shows that the peptide sequence $NS4{A}^{48-56}$ was not considered for antigenicity analysis because it has binding affinity for only 4 HLA alleles. Only $NS4{A}^{4-12}$ and $NS4{A}^{106-114}$ were considered antigenic, but the immunogenicity value of the first peptide was significantly low. Therefore, only the $NS4{A}^{106-114}$ epitope of NS4A protein was further analyzed and allowed in all phases.

$NS4{B}^{21-29}$ binds to 1 HLA class I allele, and the others are linked to $\ge$ 5 alleles. $NS4{B}^{167-175}$ was determined to be antigenic and not-allergic, but had negative immunogenicity. Of the other 6 epitopes of NS4B protein that were classified as antigenic, non-allergic, only $NS4{B}^{199-207}$ was found to be immunogenic, non-toxic and 100% conserved. (Table 10).

Originally, many epitopes were predicted for the NS5 protein, but none of the epitopes that were able to bind to the required number of alleles passed all tests (Table 11).

The identification of epitopes that can be recognized by TCD8 + lymphocytes is very important in the cellular immune responses against intracellular microorganisms, such as viruses^46^. So, we hope that these sequences can activate this response and the infected cells can be destroyed.

### HTL epitope selection

Bringing together NetMHCII and NetMHCIIpan data, a total of 365 potential epitopes with possible binding affinity to the MHC class 2 were found. Nonetheless, only 9 ($NS{3}^{47-55}$, $NS{3}^{49-57}$, $NS{3}^{217-225}$, $NS{3}^{218-226}$, $NS{3}^{267-275}$, $NS{3}^{268-276}$, $NS{3}^{448-456}$, $NS{3}^{449-457}$, $NS{5}^{398-406}$) had binding affinity of $\ge$ 5 alleles, showed considerable antigenicity, allergenicity, immunogenicity, non-toxicity results and possessed a conservation $\ge$ 90% (Table 12).

**Table 12:** List of predicted MHC-II (HTL) epitopes in YVF proteins, with the sequence of each peptide and its allele number, antigenicity prediction score, allergenicity, toxicity and conservation.

Protein	NetMHCII/NetMHCIIpan/IEDB		VaxiJen	Allertop	ToxinPred	IEDB
	Peptide sequence	Alleles	Antigenicity	Allergenicity	Toxicity	Conservancy
NS3	47-KKPDFILATDIAEMG	5	0.5351	NON-ALLERGEN	Non-Toxin	100.00% (10/10)
	49-HTMWHVTRGAFLVRN	6	0.9489	NON-ALLERGEN	Non-Toxin	100.00% (10/10)
	217-ARRRLRTLVLAPTRV	5	0.6485	NON-ALLERGEN	Non-Toxin	90.00% (9/10)
	218-RRRLRTLVLAPTRVV	7	0.5590	NON-ALLERGEN	Non-Toxin	100.00% (10/10)
	267-HATLTYRMLEPTRVV	6	0.9803	NON-ALLERGEN	Non-Toxin	100.00% (10/10)
	268-ATLTYRMLEPTRVVN	6	0.9668	NON-ALLERGEN	Non-Toxin	100.00% (10/10)
	448-KGPLRISASSAAQRR	7	0.6972	NON-ALLERGEN	Non-Toxin	100.00% (10/10)
	449-GPLRISASSAAQRRG	7	0.8559	NON-ALLERGEN	Non-Toxin	100.00% (10/10)
NS5	398-EEFIAKVRSHAAIGA	5	0.6055	NON-ALLERGEN	Non-Toxin	100.00% (4/4)

### BL epitope selection

The adaptive humoral immune response is one of the most sought after effects when it comes to vaccines. T-helper lymphocytes are crucial in the differentiation of B-lymphocytes. For this reason, finding LB epitopes that overlap with HTL epitopes ensures protection for different cell types working together. Here, a total of 98 BL epitopes, of varying size, were identified initially using the IEDB server. Of these, only 11 had a conservation of $\ge$ 90% and were used for sequence overlap analysis with HTL epitopes, in which only 5 ($NS{3}^{277-281}$, $NS{3}^{443-458}$, $NS{3}^{458-471}$ and $NS{5}^{401-409}$) found matching sequences (Table 13).

**Table 13:** B-cell epitopes with overlapping HTL epitopes.

Protein	B-cell epitope	HTL epitope
NS3	277-**PTRVV**	267-HATLTYRMLE**PTRVV**
		268-ATLTYRMLE**PTRVV**N
	433-AFKPVLVDEGRKVAI**K**	448-**K**GPLRISASSAAQRR
	458-**AAQRRG**RIGRNPNR	449-GPLRISASS**AAQRRG**
NS5	401-**IAKVRSHAA**	398-EEF**IAKVRSHAA**IGA

Recently, Tosta et al.^73^ found 28 CTL (06 HTL) epitopes overlapping with B-cell epitopes, including three (one) from the envelope, two (one) from the capsid, two from prM, four (one) from NS1, two (one) from NS2A, five from NS3, one (two) from NS4B, nine from NS5^73^. As mentioned earlier, no epitope matches our results, as we are extremely careful to avoid false positives as much as possible.

In our study, significant progress was made in immunoinformatic analysis, highlighting crucial aspects in epitope selection for the development of vaccines against flaviviruses such as YFV and Zika virus. Through comprehensive immunoinformatic analyzes, we were able to identify important epitopes in the NS3 and NS5 proteins of these viruses. The identified epitopes “PTRVV” (277-PTRVV) and “YMWLGARYL” (477-YMWLGARYL) can be compared to the promiscuous T-cell and B-cell epitopes predicted for Zika virus by Dar et al.^74^. The identification of overlapping or similar epitopes in different flaviviruses such as Zika and YFV emphasizes the potential for cross-reactivity or shared immunological features between these viruses. However, “YMWLGARYL” shows intriguing complexity in epitope selection as it fails in allergenicity assays. This finding underscores the need to consider a range of factors, including allergenicity, when developing vaccines.

### Population coverage

As we have seen so far, one of the most important concerns in vaccine development is the efficacy of the vaccine considering regional populations in the case of endemic diseases. Our method aims to predict overlapping epitopes between CTL, HTL, and B cells that can be recognized by the most common HLA alleles in Africa and South American populations and thus can induce both humoral and cellular immune responses.

Population coverage analysis was performed on 10 CTL and 5 HTL epitopes that overlapped with LB epitopes, seeking to identify the most common alleles in Africa and North America. The epitopes ${M}^{60-68}$, $NS{3}^{267-275}$, $NS{3}^{268-276}$, $NS{3}^{448-456}$, $NS{3}^{449-457}$, $NS4{A}^{106-114}$ and $NS{5}^{398-406}$ obtained a population coverage $>$ 50%. $NS2{A}^{105-113}$, $NS2{B}^{5-13}$, $NS2{B}^{8-16}$, $NS2{B}^{9-17}$, $NS2{B}^{79-87}$ and $NS4{B}^{199-207}$ had a coverage percentage between 24.57% and 42.09%. Only the epitopes $NS{3}^{123-131}$ and $NS{3}^{144-152}$ had a coverage $<$ 20%. Which shows that most of the final epitopes have excellent population coverage in the regions of interest (Table 14).

**Table 14:** Population coverage of the final CTL and HTL epitopes for the principal endemic regions of yellow fever—Africa and South America.

Protein	Sequence	Africa (%)	South American (%)	Average (%)
M	60-RVVIALLV	64.93	62.63	63.78
NS2A	105-TLGAAMVEI	53.89	28.78	41.34
NS2B	5-NEALAAAGL	26.58	25.59	26.09
	8-LAAAGLVGV	55.09	28.29	41.69
	9-AAAGLVGVL	47.63	36.54	42.09
	79-SEQGEFKLL	23.39	25.75	24.57
NS3	123-GGEIGAVAL	15.72	7.32	11.52
	144-NRNGEVIGL	14.64	9.17	11.91
	267-HATLTYRMLEPTRVV	85.29	48.48	66.89
	268-ATLTYRMLEPTRVVN	85.29	48.48	66.89
	448-KGPLRISASSAAQRR	93.78	82.61	88.20
	449-GPLRISASSAAQRRG	93.78	82.61	88.20
NS4A	106-YIMLIFFVL	86.51	90.14	88.17
NS4B	199-LASAALGPL	40.66	17.07	28.86
NS5	398-EEFIAKVRSHAAIGA	70.32	74.34	72.33

### Vaccine sequence construction

Having identified the immunogenic, nonallergenic, nontoxic, conserved epitopes of the YFV proteome capable of binding to a substantial number of alleles of the HLA system, we propose the construction of a vaccine prototype using these epitopes. Precisely because we know that vaccines based on classical vaccine platforms can cause allergic reactions because they are formulated with multiple proteins of the target organism^75^, we are interested in developing a prototype with epitopes that have the most important properties of a vaccine candidate, including minimal allergic reactions, toxicity, and side effects. In addition, we used the linkers in our multi-epitope vaccine to reduce (improve) the likelihood of misfolding of the fusion epitopes and low yield in vaccine production (folding, stability, and flexibility or stiffness of the designed chimeric vaccine candidate)^76^. At the N-terminus of our vaccine sequence, the apeptide adjuvant $\beta$-defensin was added to ensure high antigenicity and enhance the immunological response^38^.

Thus, our prototype vaccine consists of 10 CTL and 5 HTL with overlapping LB epitopes that are bonded together with the adjuvant $beta$-defensin, yielding a subunit vaccine with a total of 264 amino acids (Fig. 1). In general, the development of a vaccine is quite tedious and takes a long time. However, the early stages of this work demonstrate how immunoinformatics helps to reduce the required research time by selecting only the best sequences from a substantial number of viral proteins that are able to activate humoral and cellular immune responses against YFV.

Because simply binding to the MHC complex does not guarantee that these sequences are epitopes^77^, only those that met the previously defined criteria were used to construct our prototype, specifically.

### Physicochemical parameter prediction

The chemical formula of the vaccine sequence was predicted to be C^1213^H^1970^N^348^O^336^S^10^. The molecular weight was 27,125.72 kDa, the theoretical isoelectric point (pI) was 9.87, the instability index was 27.87, the aliphatic index was 100.42, and GRAVY was 0.315. These results indicate the preparation of a basic, stable (cut-off point $<$ 40), thermostable, and hydrophobic sequence, which, with the exception of hydrophobicity, are favorable parameters for a vaccine.

### Design, refinement and validation of tertiary structure of the vaccine prototype

The amino acid sequence was uploaded into the Raptor X server, and the tertiary structure of the vaccine that is based on several epitopes was constructed as a consequence. Of the 264 residues, 264 (100%) were modeled, while 61 (23%) predicted positions were disordered. Absolute model quality is measured by total uGDT (GDT), which was predicted to be 94. For a protein with $>$ 100 residues, uGD $>$ 50 is a good indicator^78^. The relative quality of the model was also evaluated and a p-value of 1.559 × 10^−3^ was obtained. All these results indicate a good overall tertiary model of the subunit vaccine, as the maximum number of residuals was modeled.

Vaccine model refinement was performed using the GalaxyRefine2 server. The GalaxyRefine creates 5 refined models, and model 1 was selected as the final vaccine model for further analysis because it produced the best results, including GDT-HA (0.9725), RMSD (0.342), MolProbity (1.761), Clash score (9.5), Poor rotamers (0.6), and Rama favored (96.2) (Fig. 1). A higher GDT-HA value indicates superior overall model quality. The MolProbity score serves as a critical protein quality metric, amalgamating Clashscore, Rotamer, and Ramachandran scores into a normalized single score. RMSD provides insight into the average atom deviation between the refined and unrefined structure, and ideally, it should be minimal. A lower MolProbity value often correlates with the promotion of TH2 cytokines, fostering B-cell and antibody responses^47^.

**Figure 3:**
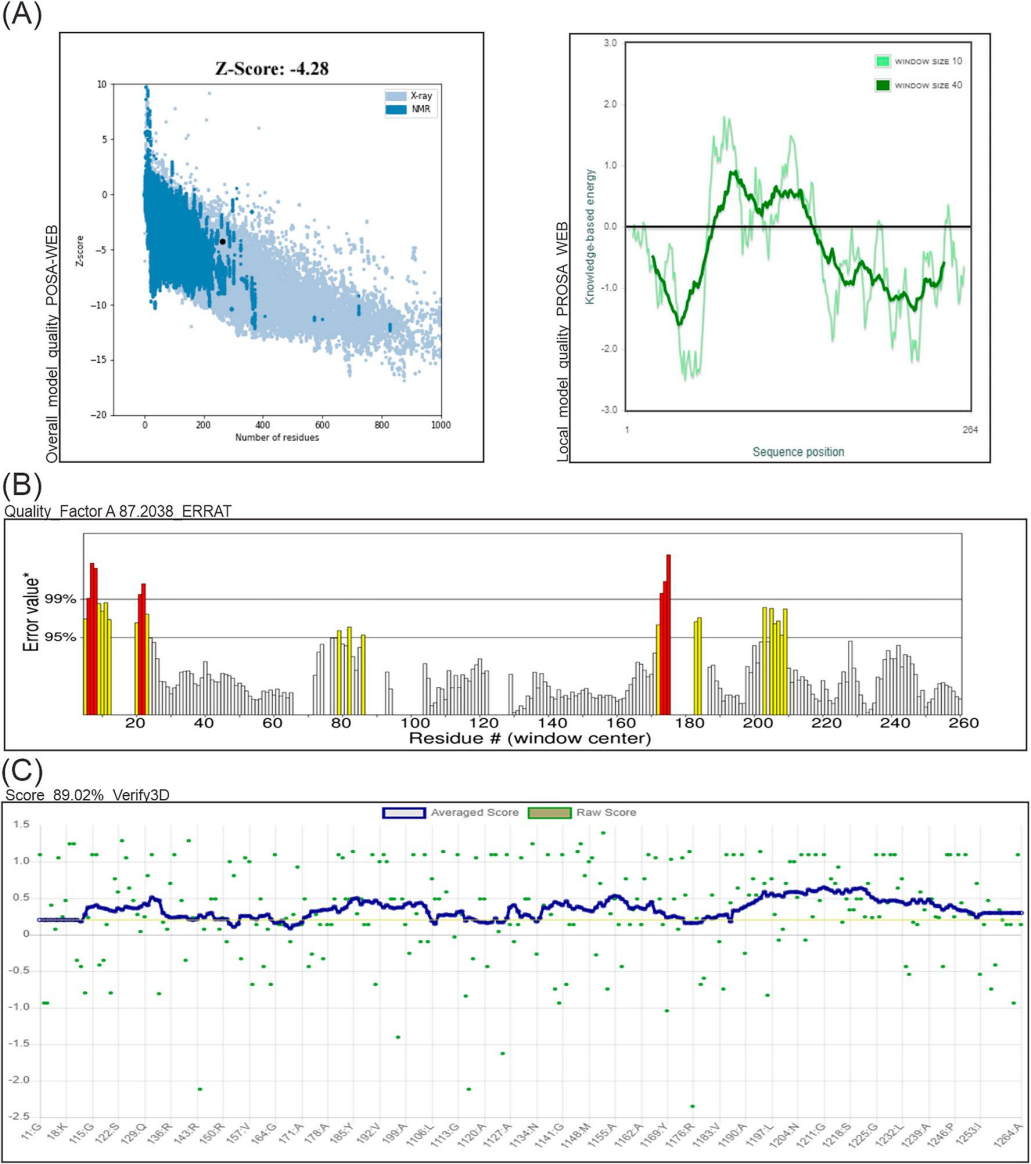
Validation of the final subunit vaccine model. (**A**) Vaccine 3D Structure Validation by ProSA-web illustrating Z-score; (**B**) Quality factor and quality score by ERRAT (**C**) Verify3D tools, respectively.

To assess the structural and stereochemical integrity of the refined models, comprehensive all-atom structure validation analyses were conducted. These analyses encompassed the use of the Ramachandran plot, Z-score, ERRAT, Verify3D, and PROCHECK tools. Ramachandran plot revealed that 95.83% of the residues were in the most favorable ranges, 5.17% were in allowed ranges. There is no percentage of disallowed ranges. The Z-score of the model was estimated to be − 4.26, which is within the range of scores normally found for native proteins of similar size (Fig. 3A**).** As per the ERRAT assessment, the protein model exhibited an overall quality factor of 87.20% when compared to highly refined structures. Furthermore, in Verify3D, 89.02% of the residues achieved a 3D-1D score of ≥ 2 (as depicted in Fig. 3B, C), indicating a high level of compatibility with the benchmark models. This validation process confirmed the tertiary structure model's integrity, with no critical errors detected.

**Figure 4:**
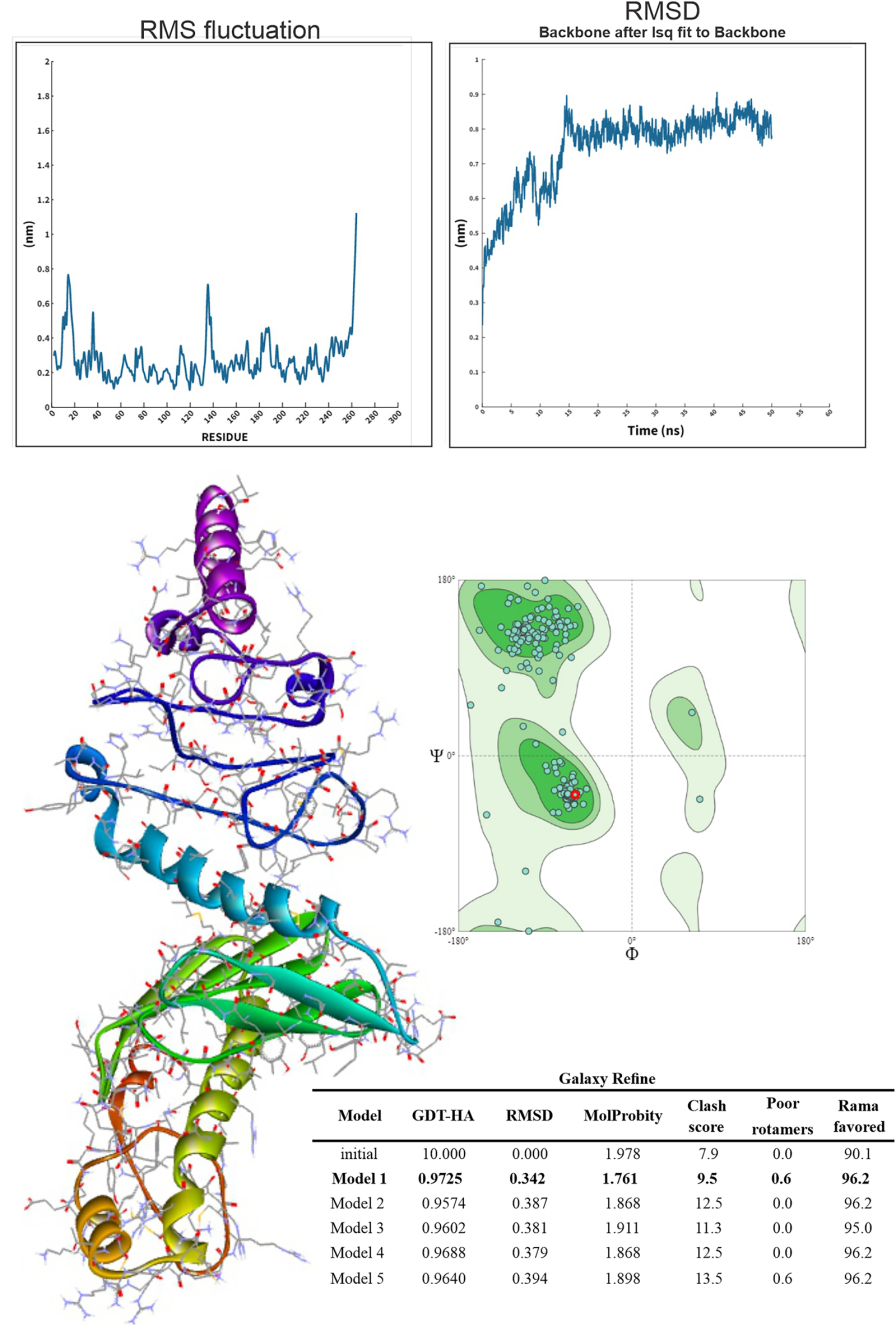
3D structural conformation of the multi-epitope subunit vaccine after homology modeling and refinement by SwissModel and Galaxy Refine servers.

The results of the Molecular Dynamics (MD) simulations, portraying the stability and flexibility of the vaccine, are illustrated in Fig. 4. The root mean square deviation (RMSD) plot initially showed very little variation up to 15 ns in the range of 0.4–0.9 nm, followed by a stable conformation up to 50 ns. This stability could be due to the higher number of stable bonds of the target protein. Then, the root mean square fluctuation (RMSF) value was calculated to investigate the structural flexibility of the backbone atoms of the protein. The results show that there are no large fluctuations and that the complex is flexible (RMSF $\le$ 0.8 nm).

MD simulations can be used prior to docking, as a set of “new” and broader protein conformations can be extracted from the processing of the resulting trajectory and used as targets for docking. These results suggest that the developed vaccines can strongly interact with immune receptors.

### Molecular docking, refinement and comparative analysis

In the recognition of YFV by the host immune system, Toll-Like Receptor 2 (TLR-2) has been identified along with three other Toll-Like receptors (7, 8, and 9) as critical in the interactions between the 17D vaccine and human cells. According to Pulendran^47^, TLR-2 has the ability to induce both Th1 and Th2 cells and can indirectly facilitate either antibody production or a cytotoxic cellular response. Hence, the interaction between TLR-2 and the vaccine prototype was evaluated through protein–protein docking and was validated through a comprehensive structural validation protocol.

The binding of the refined vaccine model performed by the PatchDock server with TLR-2 resulted in 20 models ranked by a geometric complementarity score. The top 10 models were refined based on binding energy using FireDock (Fig. 2a). In the HADDOCK refinement process, a solvent shell was constructed around the top complexes, followed by a series of brief Molecular Dynamics (MD) simulations governed by the subsequent parameters: all atoms, excluding side-chain atoms at the interface, were restrained to their original positions. Subsequently, 1250 MD steps were executed at 300 K with position restraints applied to heavy atoms not participating in the Protein–Protein Interaction (PPI) (specifically, residues outside of intermolecular contacts within 5 Å). The systems were gradually cooled down through 1000 MD steps at 300, 200, and 100 K, while maintaining position restraints on the backbone atoms of the protein complex, except for those at the interface. In the final step, the optimal model was refined by applying the methodology of quantum mechanics/molecular mechanics (QM/MM).

In our research, we conducted a comparative analysis between structures derived from basic geometric optimization using classical mechanics, resulting in the 'PatchDock + FireDock + HADDOCK' (PFH) structure, and those obtained from advanced computational methods such as molecular dynamics (MD) simulations and quantum mechanics/molecular mechanics (QM/MM) techniques, which led to the ‘PatchDock + FireDock + HADDOCK + MD + QM/MM’ (PFHMQM) structure. We utilized the PRODIGY tool to assess energy scores and binding affinity of both models, as shown in Fig. 2b. This comparison unveiled significant discrepancies, particularly in the RMSD and Gibbs free energy parameters. The RMSD for PFH (6.3 Å) was higher than that for PFHMQM (2.2 Å), and ΔG_PFHMQM (− 17.1 kcal/mol) was lower compared to ΔG_PFH (− 12.9 kcal/mol). These results emphasize the importance of sophisticated geometric optimization calculations that provide a more accurate and reliable depiction of the system's electronic cloud. Consequently, even subtle structural changes brought about by these calculations can significantly alter ligand binding to receptors.

**Figure 5:**
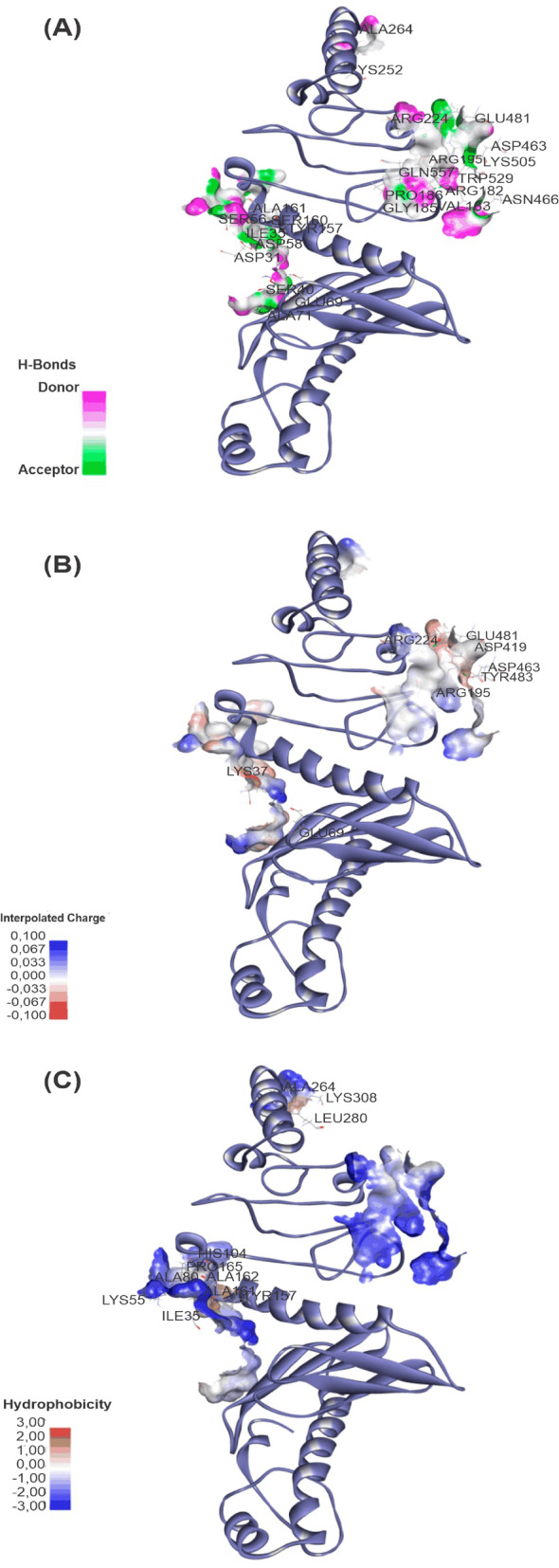
Intermolecular interactions between vaccine-TLR-2 evidenced by the contact surface. (**A**) Hydrogen bonds. (**B**) Electrostatic bonds. (**C**) Hydrophobic bonds.

Finally, Discovery Studio, LigPlot + and PoseView were used to extract the graphical image of the vaccine-receptor interaction profile of the QM /MM complex. Of the total 28 intermolecular interactions, 14 were of hydrogen bonding type (GLU69-SER40, ALA161-SER56, ALA264-LYS252, VAL183-LYS505, GLY185-TRP529, ALA71-SER40, TYR157-ASP31, SER160-ASP58, ARG182-ASN466 , SER160-SER56, ARG182-ASN466, PRO186-GLN557, ARG195-ASP463, ARG224-GLU481), 8 strongly electrostatic in nature (ARG195-ASP463, ARG224-GLU481, ARG224-GLU481, GLU69-LYS37, ARG195-ARGSP2419-GLU481, ARG195-TYR483) and 7 with a polar/hydrophobic properties (PRO165-LYS55, ALA162-ALA80, ALA161-ILE35, LA264-LEU280, ALA264-LYS308, ALA162-HIS104, TYR157-ILE35) (Fig. 5).

In summary, the docking analysis revealed robust interactions between the vaccine molecules and immune cells. However, it's important to note that this assessment was theoretical, and a real evaluation of binding potency within the host is still needed. To validate the docking results, various techniques, including molecular dynamics and QM/MM simulations, were employed. The MD + QM/MM analysis predicted substantial binding stability, which is vital for ensuring the lasting effectiveness and durability of the vaccine construct within the host.

#### Codon adaptation and in silico cloning

Validation of the candidate vaccine construct necessitates immunoreactivity screening via serological analysis^32^. This involves the expression of the recombinant protein in Escherichia coli expression systems, as these systems are well-suited for the production of recombinant proteins^79,80^. Codon optimization performed to achieve high-level expression of our vaccine prototype (801 nucleotides) in E. coli (strain K12) shows that the codon adaptability index (1.0) and GC content (55.42%) were favorable for a high-level expression in bacteria.

**Figure 6:**
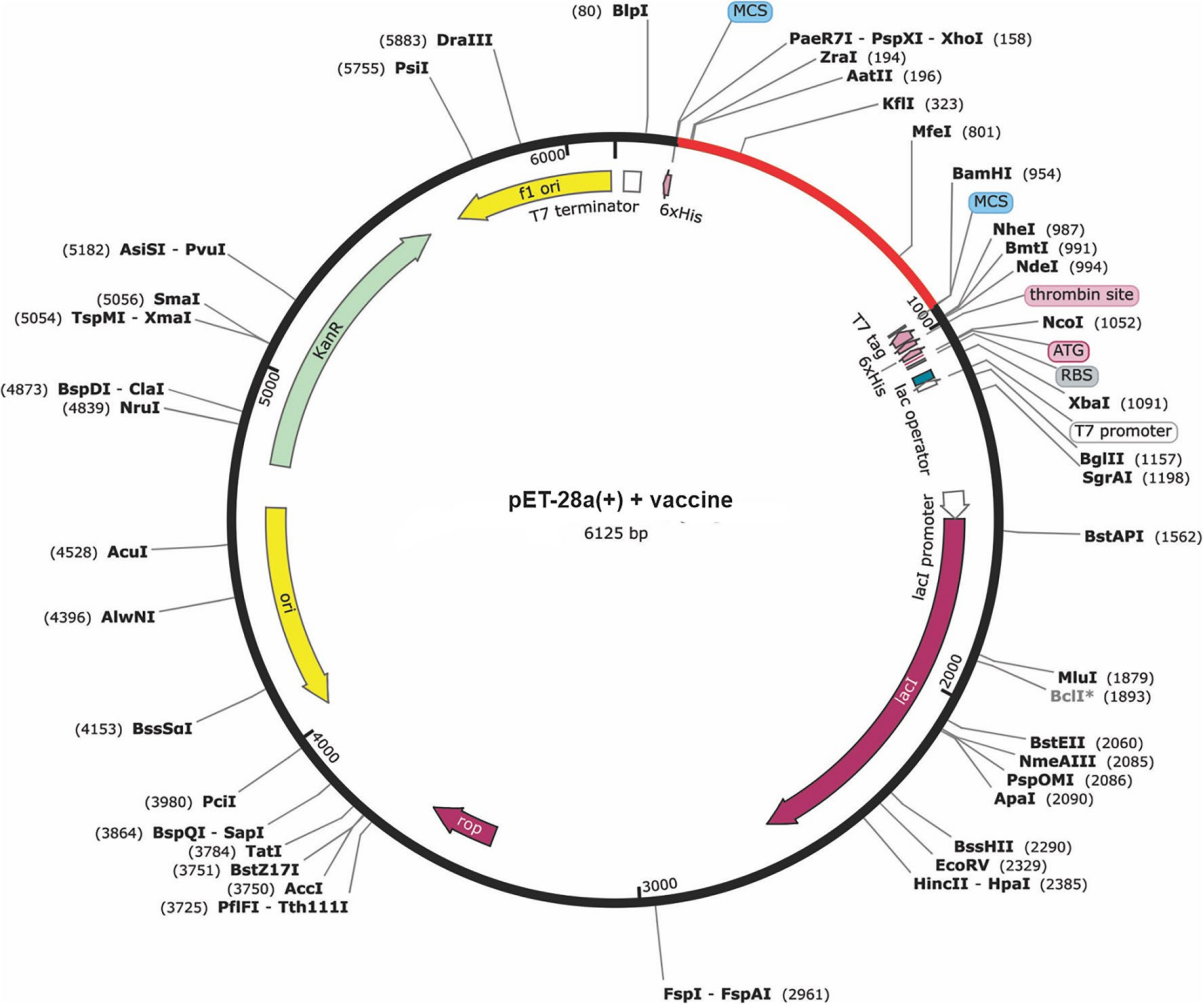
In silico cloning of the final vaccine construct into the pET28a ( +) expression vector.

For gene expression in an organism of interest, the ideal CAI value should be 1.00, but a value $>$ 0.8 is also considered good and the percent GC content should be in the range of 30–70%. These results suggest a good expression of the genetically engineered vaccine in the *E. coli* K-12 strain. After evaluating these parameters and once the sequence was free of commercially available restriction sites, two restriction sites XhoI and BamHI were added to the N- and C-terminal, respectively, of the optimized reverse codon sequence. Ultimately, the recombinant plasmid was generated by incorporating the reverse sequence of the adapted codon into the pET-28a( +) vector. The chosen restriction sites for this insertion were PspXI and BamHI, which served as the starting and ending cut points, respectively (Fig. 6).

#### Immune simulation of the multiepitope vaccine

**Figure 7:**
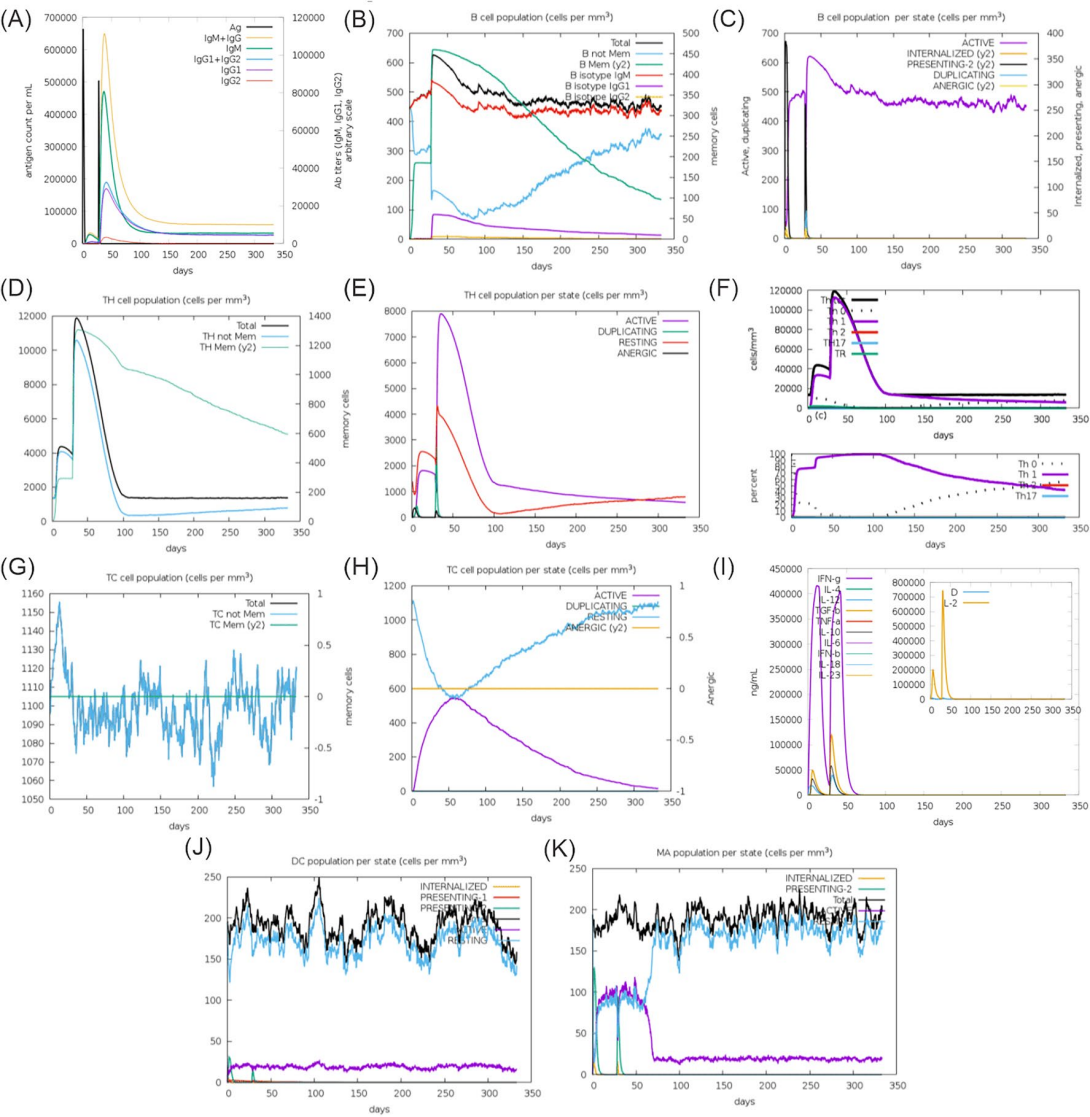
Graphs of the immune response elicited at the first and second dose of vaccine. (**A**) Antigens (Ag) and antibodies (Ac) Antibodies are divided by isotypes: IgM + IgG, IgM, IgG1 + IgG2, IgG1 and IgG2; (**B**) B lymphocyte (LB) population: total, memory cells, no memory (B non Mem) and IgM, IgG1 and IgG2 isotypes; (**C**) B lymphocyte (LB) population by state: active, internalized Ag (Internalized), presenting to MHC class II (Presenting-2), doubling and anergic; (**D**) T-helper lymphocyte (THL) population: total, memory cells (TH Mem) and no memory (TH non Mem); (**E**) T-helper lymphocyte (LTH) population by state: active, doubling, resting, and anergic; (**F**) T-helper lymphocyte profile; (**G**) T-cytotoxic lymphocyte (CTL) population: total, memory cells (TC Mem) and no memory (TC non-Mem), (**H**) T-cytotoxic lymphocyte population by state: active, doubling, resting and anergic; (**I**) Cytokine and interleukin concentration. D in the inserted graph is a danger signal; (**J**) Dendritic cells: internalized Ag, presenting to MHC class I and II (Presenting-1 and Presenting-2), total, active and resting, (**k**) Macrophages: internalized Ag, presenting to MHC class II, total, active and resting.

Although available immunoinformatic algorithms for in silico predictors of epitopes and potential vaccines demonstrate remarkable precision, one of the greatest hurdles in the field is to correctly stimulate the immune system^81^. Here, the immune response simulation results obtained using C-ImmSim revealed that the secondary immune response, overall, was significantly greater than the first response, coinciding with what occurs in vivo immune response (Fig. 7).

One of the key elements in the immune response to the Yellow Fever Virus (YFV) is the presence of neutralizing antibodies, primarily of the IgG type. These antibodies play a critical role in establishing a long-lasting immune defense against YFV and are considered a significant gauge of immunity^70^. Following the administration of a peptide vaccine booster, there was a notable increase in the antibody response, accompanied by a simultaneous decrease in antigen levels. This was evident from the narrower base of the antigenic spike in comparison to the previous dosage. During this period, a predominant humoral response was observed, characterized by higher IgM production. Despite the increased IgM production, the booster resulted in higher quantities of IgG compared to the initial dose, indicating a degree of seroconversion (see Fig. 7A).

Analysis of B-cell population per cell showed overall B-cell memory responses higher after boost dose and which remains in greater proportion for more than 200 days (Fig. 7B). In Fig. 7C, it is evident that the B-cell population remained higher and stable over the same time periods. As for the T cells, after the prime dose, there was a simultaneous increase in effector T-helper (Th) cells, while Th-memory cells showed a lower response, but after the boost, Th-memory respond faster and higher as expected for an efficient immune response to antigenic challenge (Fig. 7D). At the same period, there was a predominance of Th active cells (Fig. 7E). There was an increase of active effect cytotoxic T-cells after prime dose and following around day 30 (Fig. 7G, H), which corresponds to the period of application of the second dose, explaining that there was a previous recognition of these antigens.

After the initial prime dose, there was an initial surge in the IFN-γ response, which is associated with both CD8 + T-cell and CD4 + Th1 response. Additionally, there was a notable response in terms of IL-10 and TGF-β cytokines, which are associated with the T-regulatory (T-reg) phenotype. After boost, IFN-$\gamma$ also peaked and TGF-b had a higher production than prime dose (Fig. 7I). The cytokines IL-12 and IL-2 increased in the second phase of vaccination, this increase reinforces the modulation to a Th1 cell-mediated response^82^, moreover, IL-12 production correlates with Natural Killer (NK) cells. NK cells are innate lymphocytes and their role is well established in the immune response against viruses^83^. Its function is regulated, in part, by the action of cytokines such as IL-12, which act to activate these cells. The importance of NK cells in yellow fever disease has been suggested^84^.

There was observed increase in dendritic cells activities throughout the duration of the simulation (Fig. 7J). Active macrophages increase after the administration of vaccine doses, being probably responsible for the antigenic presentation of the vaccine peptide (Fig. 7K). This is an important event indicating that the vaccine construct showed the ability to stimulate the right immunological compartment to effective response. Moreover, the increase in the Th1 response observed after the first and second doses are important because they demonstrate that the construct vaccine is effective in mounting a specific response to eliminate the YF virus, considering that CD4 T cells are important to activate CD8 T cells, and also for the activation of B cells, favoring affinity maturation and immunoglobulin isotype switching (Fig. 7F).

Our results confirm the findings of de Melo et al.^70^, who were the first to demonstrate that TCD4 + lymphocyte-recognized epitopes play a crucial role in the immune response against YFV. Helping humoral immune response (B-lymphocyte differentiation) activated TCD4 + cells may differentiate into Th1, Th2 and Th17, based on their cytokine secretion profile. This type of mixed response has already been verified with the yellow fever vaccine 17DD, including IL-2, IFN-$\gamma$, TNF-$\alpha$, IL-12 (TH1), IL-4, IL-5, IL-10, and IL-13 (TH2). TH2 cytokines promote B-cell and antibody responses whereas TH1 T cells habitually promote CD8 + responses^47^.

The multi-epitope vaccine under consideration was intended to serve as an effective vaccine and could also function as a booster in case of mutations in the YFV. An evaluation of memory T-cell responses and neutralizing antibody levels in individuals who received primary vaccines ranging from 45 to 13 years after vaccination revealed a decline in memory responses after 10 years of YFV vaccination. This decline was observed in classical memory B-cells, CD4 + and CD8 + T-cells^85^. To enhance memory responses, booster shots may be necessary, which could include the multi-epitope vaccine as an adjuvant peptide to provide a targeted and directed immune response against YFV.

## Conclusion

In summary, we identified 15 epitopes for T and B cells in the structural and nonstructural proteins of 196 strains of YFV that have essential properties for eliciting an effective and population-wide immune response, namely high antigenicity and immunogenicity, extensive conservation among different strains, comprehensive population coverage, non-toxicity and non-allergenic in humans. On this basis, we have developed and optimized the spatial geometry of a prototype subunit vaccine with appropriate physicochemical properties, the ability to bind to the Toll-2 receptor, the potential to elicit an effective and durable immune response in two doses, and a coding region that can be successfully inserted into a cloning vector. Experimental studies (in vitro and in vivo) are required to validate the efficacy and safety of the proposed vaccine (Supplementary information 1).

### Supplementary Information

Supplementary Information.

## Data Availability

All data generated or analysed during this study are included in this published article.
