# Supplementary material for: Advancing molecular modeling and reverse vaccinology in broad-spectrum yellow fever virus vaccine development
Source: Sci Rep. 2024 May 12;14:10842. doi: 10.1038/s41598-024-60680-9 (PMC11089047; doi:10.1038/s41598-024-60680-9)
Supplement: Supplementary file 1 — Supplementary Information. [file 41598_2024_60680_MOESM1_ESM.zip › Yellow_Fever_data/Figures_pdf/Figure_2.pdf]

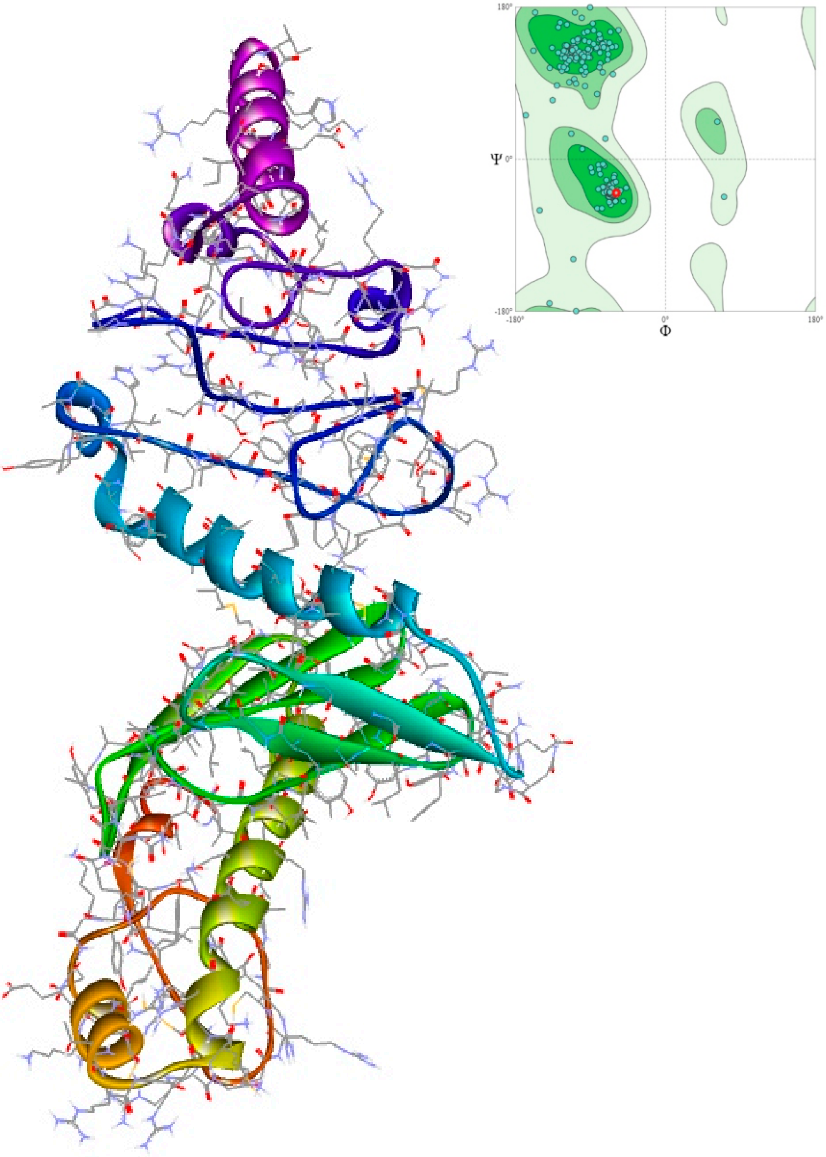

Galaxy Refine

	Model	GDT-HA	RMSD	MolProbity	Clash score	Poor rotamers	Rama favored
SwissModel	initial	10.000	0.000	1.978	7.9	0.0	90.1
	**Model 1**	**0.9725**	**0.342**	**1.761**	**9.5**	**0.6**	**96.2**
	Model 2	0.9574	0.387	1.868	12.5	0.0	96.2
	Model 3	0.9602	0.381	1.911	11.3	0.0	95.0
	Model 4	0.9688	0.379	1.868	12.5	0.0	96.2
	Model 5	0.9640	0.394	1.898	13.5	0.6	96.2
