# Supplementary material for: Advancing molecular modeling and reverse vaccinology in broad-spectrum yellow fever virus vaccine development
Source: Sci Rep. 2024 May 12;14:10842. doi: 10.1038/s41598-024-60680-9 (PMC11089047; doi:10.1038/s41598-024-60680-9)
Supplement: Supplementary file 1 — Supplementary Information. [file 41598_2024_60680_MOESM1_ESM.zip › Yellow_Fever_data/Figures_pdf/Figure_4.pdf]

(A)

RANK	Global Energy	Attractive VdW	Repulsive VdW	ACE	HB	RANK
**1**	**-12.82**	**-31.31**	**7.11**	**18.48**	**-5.19**	**1**
2	0.06	-13.72	4.58	9.82	-1.67	2
3	4.78	-2.21	0.81	1.10	0.00	3
4	28.42	-32.58	54.68	9.31	-2.22	4
5	48.14	-9.19	19.47	8.47	-1.12	5
6	97.25	-9.97	94.68	9.59	-2.38	6
7	113.20	-8.92	139.52	4.16	-2.34	7
8	236.92	-57.34	354.72	20.77	-8.54	8
9	620.92	-34.07	758.60	17.81	-5.43	9
10	1245.00	-67.80	1683.87	10.88	-8.78	10

(B)

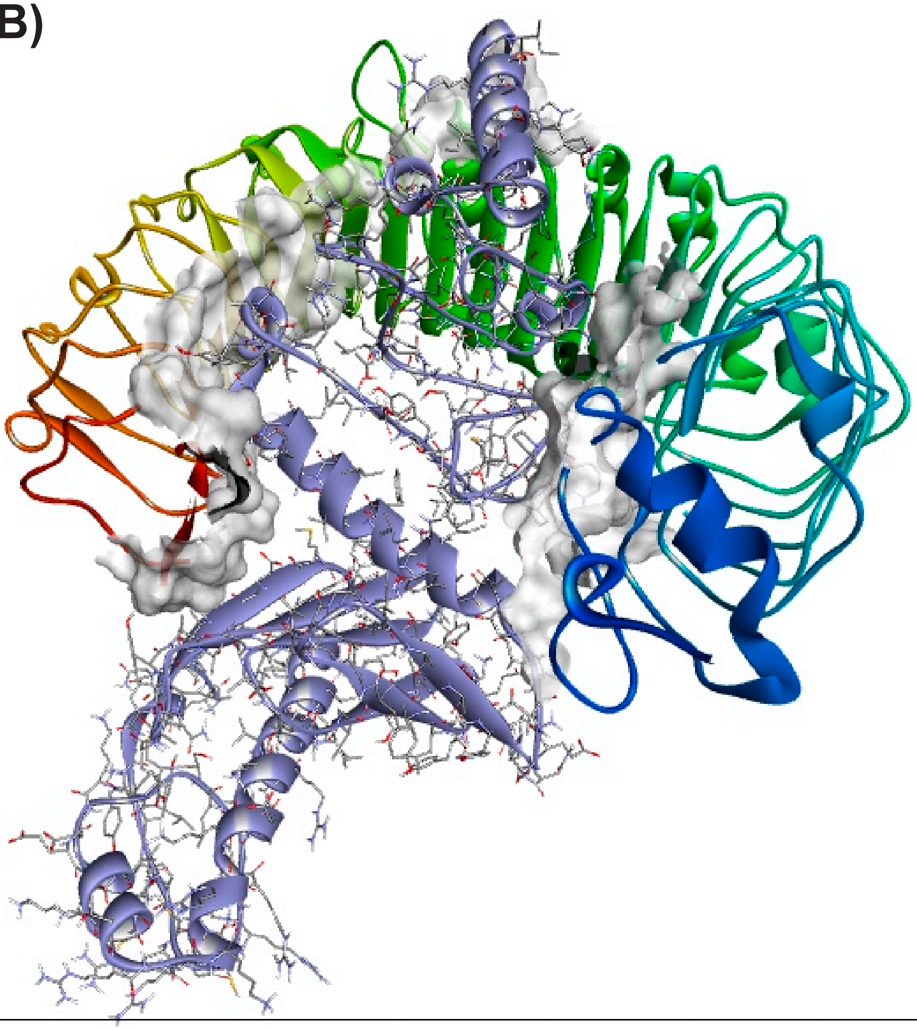

Vaccine - TLR2	Van der Waals energy - E_{vdw} (kcal/mol-1)	Electrostatic energy - E_{elec} (kcal/mol-1)	Desolvation energy (kcal/mol-1)	ΔG (kcal mol-1)	Kd (M) at 25.0 ℃	RMSD (Å) to PatchDock
PFH	-	-	-	-12.9	3.4E-11	6.3
PFHMQM	-83.1	-259.9	4.6	-17.1	1.7E-11	2.2
