# Supplementary material for: Advancing molecular modeling and reverse vaccinology in broad-spectrum yellow fever virus vaccine development
Source: Sci Rep. 2024 May 12;14:10842. doi: 10.1038/s41598-024-60680-9 (PMC11089047; doi:10.1038/s41598-024-60680-9)
Supplement: Supplementary file 1 — Supplementary Information. [file 41598_2024_60680_MOESM1_ESM.zip › Yellow_Fever_data/Figures_pdf/Figure_1.pdf]

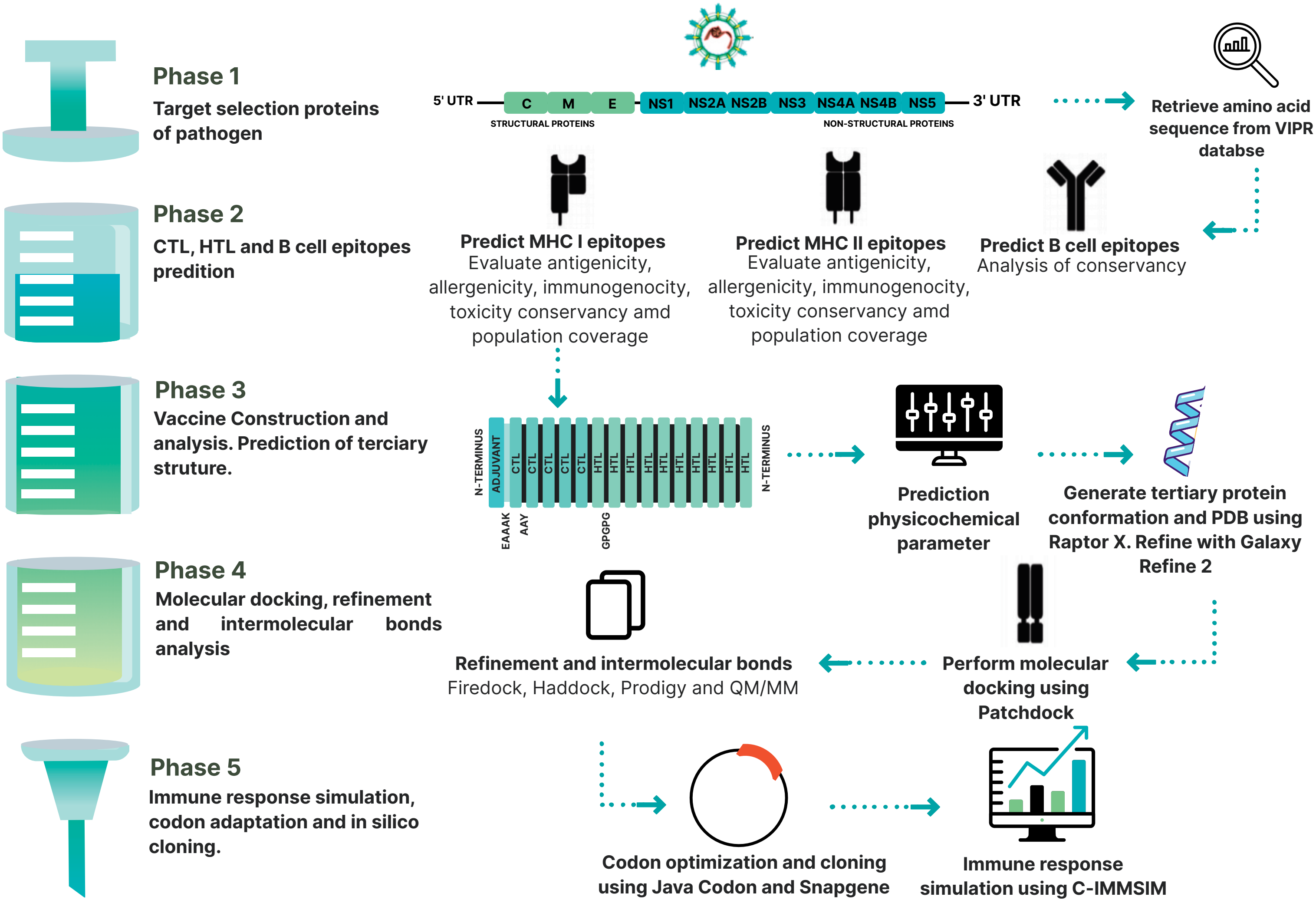
Phase 1
Target selection proteins of pathogen
5' UTR
C
M
E
NS1
NS2A
NS2B
NS3
NS4A
NS4B
NS5
3' UTR
STRUCTURAL PROTEINS
NON-STRUCTURAL PROTEINS
Retrieve amino acid sequence from VIPR databse
Phase 2
CTL, HTL and B cell epitopes predition
Predict MHC I epitopes
Evaluate antigenicity, allergenicity, immunogenocity, toxicity conservancy amd population coverage
Predict MHC II epitopes
Evaluate antigenicity, allergenicity, immunogenocity, toxicity conservancy amd population coverage
Predict B cell epitopes
Analysis of conservancy
Phase 3
Vaccine Construction and analysis. Prediction of terciary struture.
N-TERMINUS
ADJUVANT
CTL
CTL
CTL
CTL
CTL
HTL
HTL
HTL
HTL
HTL
HTL
HTL
HTL
HTL
HTL
N-TERMINUS
EAAAK
AAY
GPGPG
Prediction physicochemical parameter
Generate tertiary protein conformation and PDB using Raptor X. Refine with Galaxy Refine 2
Phase 4
Molecular docking, refinement and intermolecular bonds analysis
Refinement and intermolecular bonds
Firedock, Haddock, Prodigy and QM/MM
Perform molecular docking using Patchdock
Phase 5
Immune response simulation, codon adaptation and in silico cloning.
Codon optimization and cloning using Java Codon and Snapgene
Immune response simulation using C-IMMSIM
