# Supplementary material for: Advancing molecular modeling and reverse vaccinology in broad-spectrum yellow fever virus vaccine development
Source: Sci Rep. 2024 May 12;14:10842. doi: 10.1038/s41598-024-60680-9 (PMC11089047; doi:10.1038/s41598-024-60680-9)
Supplement: Supplementary file 1 — Supplementary Information. [file 41598_2024_60680_MOESM1_ESM.zip › Yellow_Fever_data/4_Vaccine_validation/RANK1.docx]

| **RANK** | **Global Energy** | **Attractive VdW** | **Repulsive VdW** | **ACE** | **HB** |
| --- | --- | --- | --- | --- | --- |
| **1** | **-27.32** | **-13.48** | **10.84** | **11.42** | **-3.78** |
| 2 | 30.17 | -9.57 | 4.40 | 10.91 | -2.57 |
| 3 | 40.35 | -9.39 | 0.20 | 10.84 | -1.51 |
| 4 | 49.14 | -43.34 | 46.51 | 21.10 | -5.51 |
| 5 | 61.74 | -18.80 | 77.98 | 10.90 | -2.29 |
| 6 | 62.67 | -26.35 | 108.46 | 7.33 | -4.34 |
| 7 | 132.65 | -5.75 | 145.20 | 1.82 | 0.00 |
| 8 | 406.56 | -21.79 | 523.48 | 10.23 | -2.08 |
| 9 | 644.91 | -56.58 | 855.18 | 21.58 | -9.48 |
| 10 | 1799.12 | -63.86 | 2361.91 | 10.75 | -14.39 |
