# Supplementary material for: Advancing molecular modeling and reverse vaccinology in broad-spectrum yellow fever virus vaccine development
Source: Sci Rep. 2024 May 12;14:10842. doi: 10.1038/s41598-024-60680-9 (PMC11089047; doi:10.1038/s41598-024-60680-9)
Supplement: Supplementary file 1 — Supplementary Information. [file 41598_2024_60680_MOESM1_ESM.zip › Yellow_Fever_data/1_Acquisition_proteins/Alignment_consensus_ViPR.docx]

**Proteina preM**

>case_#1|VIPR_ALG4_256274852_482_973/1-164

VTLVRKNRWLLLNVTSEDLGKTFSVGTGNCTTNILEAKYWCPDSMEYNCPNLSPREEPDDIDCWCYGVENVR

VAYGKCDSAGRSRRSRRAIDLPTHENHGLKTRQEKWMTGRMGERQLQKIERWFVRNPFFAVTALTIAYLVGS

NMTQRVVIALLVLAVGPAYS

>BeH622205|VIPR_ALG4_383464642_482_973/1-164

VTLVRRNRWLLLNVTSEDLGKTFSVGTGNCTTNILEAKYWCSDSMEYNCPNLSPREEPDDIDCWCYGVENVR

VAYGKCDSAGRSRRSRRAIDLPTHENHGLKTRQEKWMTGRMGERQLQKIERWLVRNPFFAATALAIAYLVGS

NMTQRVVIALLVLAVGPAYS

>BeH422973|VIPR_ALG4_383464632_482_973/1-164

VTLVRKNRWLLLNVTSEDLGKTFSVGTGNCTTNILEAKYWCPDSMEYNCPNLSPREEPDDIDCWCYGVENVR

VAYGKCDSAGRSRRSRRAIDLPTHENHGLKTRQEKWMTGRMGERQLQKIERWLVRNPFFAATALAIAYLVGS

NMTQRVVIALLVLAVGPAYS

>BeH413820|VIPR_ALG4_383464630_482_973/1-164

VTLVRKSRWLLLNVTSEDLGKTFSVGTGNCTTNILDAKNWCPDSMEYNCPNLSPREEPDDIDCWCYGVENVR

VAYGKCDSAGRSRRSRRAIDLPTHENHGLKTRQEKWMTGRMGERQLQKIERWLVRNPFFAVTALAIAYLVGS

NMTQRVVIALLVLAVGPAYS

>BeH394880|VIPR_ALG4_383464628_482_973/1-164

VTLVRRNRWLLLNVTSEDLGKTFSVGTGNCTTNILEAKYWCPDSMEYNCPNLSPREEPDDIDCWCYGVENVR

VAYGKCDSAGRSRRSRRAIDLPTHENHGLKTRQEKWMTGRMGERQLQKIERWLVRNPFFAATALAIAYLVGS

NMTQRVVIALLVLAVGPAYS

>BeH526722|VIPR_ALG4_383464640_482_973/1-164

VTLVRRSRWLLLNVTSEDLGKTFSVGTGNCTTNILEAKYWCPDSMEYNCPNLSPREEPDDIDCWCYGVENVR

VAYGKCDSAGRSRRSRRAIDLPTHENHGLKTRQEKWMTGRMGERQLQKIERWLVRNPFFAATALAIAYLVGS

NMTQRVVIALLVLAVGPAYS

>88/1999|VIPR_ALG4_586947712_482_973/1-164

VTLVRKSRWLLLNVTSEDLGKTFSVGTGNCTTNILEAKNWCPDSMEYNCPNLSPREEPDDIDCWCYGVENVR

VAYGKCDSAGRSRRSRRAIDLPTHENHGLKTRQEKWMTGRMGERQLQKIERWLVRNPFFAVTALAIAYLVGS

NMTQRVVIALLVLAVGPAYS

>Consensus/1-164 Percentage Identity Consensus

VTLVRKNRWLLLNVTSEDLGKTFSVGTGNCTTNILEAKYWCPDSMEYNCPNLSPREEPDDIDCWCYGVENVR

VAYGKCDSAGRSRRSRRAIDLPTHENHGLKTRQEKWMTGRMGERQLQKIERWLVRNPFFAATALAIAYLVGS

NMTQRVVIALLVLAVGPAYS

**Proteina M (proteína estrutural) 3 proteinas**

>case_#1|VIPR_ALG4_256274852_749_973

AIDLPTHENHGLKTRQEKWMTGRMGERQLQKIERWFVRNPFFAVTALTIAYLVGSNMTQRVVIALLVLAVGP

AYS

>BeH413820|VIPR_ALG4_383464630_749_973

AIDLPTHENHGLKTRQEKWMTGRMGERQLQKIERWLVRNPFFAVTALAIAYLVGSNMTQRVVIALLVLAVGP

AYS

>BeH394880|VIPR_ALG4_383464628_749_973

AIDLPTHENHGLKTRQEKWMTGRMGERQLQKIERWLVRNPFFAATALAIAYLVGSNMTQRVVIALLVLAVGP

AYS

**>Consenso M**

AIDLPTHENHGLKTRQEKWMTGRMGERQLQKIERWLVRNPFFAVTALAIAYLVGSNMTQRVVIALLVLAVGP

AYS

RNPFFAVTALAIAYL

**Proteina E (estrutural) 5 proteinas**

**>BeH423602|VIPR_ALG4_383464634_974_2452**

AHCIGITDRDFIEGVHGGTWVSATLEQDKCVTVMAPDKPSLDISLETVAIDGPAEARKVCYSAVLTNVKIND

KCPSTGEAHLEEENEGDNACKRTYSDRGWGNGCGLFGKGSIVACAKFTCAKSMSLFEVDQTKIQYVIRAQLH

VGAKQENWNTDIKTLKFDALSGSQEAEFTGYGKATLECQVQTAVDFSNSYIAEMEKESWIVDKQWAQDLTLP

WQSGSGGVWREMHHLVEFEPPHAATIKVLALGNQEGSLKTALTGAMRVTKDTNNSKLYKLHGGHVACRVKLS

ALTLKGTSYKMCTDKMSFVKNPTDTGHGTAVMQVKVPKGAPCRIPVMVADDLTAAVNKGILVTVNPIASTND

DEVLIEVNPPFGDSYIIVGTGDSRLTYQWHKEGSSIGKLFTQTMKGAERLAVMGDAAWDFGSAGGFFTSVGK

GIHTVFGSAFQGLFGGLSWITKVIMGVVLIWVGINTRNMTMSMSMILVGVIMMFLSLGVGA

**>BeH422973|VIPR_ALG4_383464632_974_2452**

AHCIGITDRDFIEGVHGGTWVSATLEQDKCVTVMAPDKPSLDISLETVAIDGPAEARKVCYSAVLTNVKIND

KCPSTGEAHLEEENEGDNACKRTYSDRGWGNGCGLFGKGSIVACAKFACAKSMSLFEVDQTKIQYVIRAQLH

VGAKQENWNTDIKTLKFDALSGSQEAEFTGYGRATLECQVQTAVDFSNSYIAEMEKESWIVDKQWAQDLTLP

WQSGSGGVWREMHHLVEFEPPHAATIKVLALGNQEGSLKTALTGAMRVTKDTNNSKLYKLHGGHVACRVKLS

ALTLKGTSYKMCTDKMSFVKNPTDTGHGTAVMQVKVPKGAPCRIPVMVADDLTAAVNKGILVTVNPIASTND

DEVLIEVNPPFGDSYIIVGTGDSRLTYQWHKEGSSIGKLFTQTMKGAERLAVMGDAAWDFGSAGGFFTSVGK

GIHTVFGSAFQGLFGGLSWITKVIMGVVLIWVGINTRNMTMSMSMILVGVIMMFLSLGVGA

**>BeH413820|VIPR_ALG4_383464630_974_2452**

AHCIGITDRDFIEGVHGGTWVSATLEQDKCVTVMAPDKPSLDISLETVAIDGPAEARKVCYSAVLTHVKIND

KCPSTGEAHLAEENEGDNACKRTYSDRGWGNGCGLFGKGSIVACAKFTCAKSMSLFEVDQTKIQYVIRAQLH

VGAKQENWNADIKTLKFDALSGSQEAEFTGYGKATLECQVQTAVDFSNSYIAEMEKESWIVDRQWAQDLTLP

WQSGSGGVWREMHHLVEFEPPHAATIKVLALGNQEGSLKTALTGAMRVTKDANGSNLYKLHGGHVSCRVKLS

ALTLKGTSYKMCTDKMSFVKNPTDTGHGTAVMQVKVPKGAPCRIPVMVADDLTAAVNKGILVTVNPIASTNE

DEVLIEVNPPFGDSYIIVGTGDSRLTYQWHKEGSSIGKLFTQTMKGAERLAVMGDAAWDFSSAGGFFTSVGK

GIHMVFGSAFQGLFGGLSWITKVIIGAVLIWVGINMRNMTMSMGMILVGVIMMFLSLGVGA

**>BeH394880|VIPR_ALG4_383464628_974_2452**

AHCIGITDRDFIEGVHGGTWVSATLEQDKCVTVMAPDKPSLDISLETVAIDGPAEARKVCYSAVLTNVKIND

KCPSTGEAHLEEENEGDNACKRTYSDRGWGNGCGLFGKGSIVACAKFTCAKSMSLFEVDQTKIQYVIRAQLH

VGAKQENWNTDIKTLKFDALSGSQEAEFTGYGKATLECQVQTAVDFSNSYIAEMEKESWIVDKQWAQDLTLP

WQSGSGGVWREMHHLVEFEPPHAATIKVLALGNQEGSLKTALTGAMRVTKDTNNSKLYKLHGGHVSCRVKLS

ALTLKGTSYKMCTDKMSFVKNPTDTGHGTAVMQVKVPKGAPCRIPVMVADDLTAAVNKGILVTVNPIASTND

DEVLIEVNPPFGDSYIIVGTGDSRLTYQWHKEGSSIGKLFTQTMKGAERLAVMGDAAWDFGSAGGFFTSVGK

GIHTVFGSAFQGLFGGLSWITKVIMGVVLIWVGINTRNMTMSMSMILVGVIMMFLSLGVGA

**>88/1999|VIPR_ALG4_586947712_974_2452**

AHCIGITDRDFIEGVHGGTWVSATLEQDKCVTVMAPDKPSLDISLETVAIDGPAEARKVCYSAVLTHVKIND

KCPSTGEAHLAEENEGDHACKRTYSDRGWGNGCGLFGKGSIVACAKFTCAKSMSLFEVDQTKIQYVIRAQLH

VGAKQENWNADIKTLKFDALSGSQEAEFTGYGKATLECQVQTAVDFSNSYIAEMEKESWIVDRQWAQDLTLP

WQSGSGGVWREMHHLVEFEPPHAATIKVLALGNQEGSLKTALTGAMRVTKDTNGSNLYKLHGGHVSCRVKLS

ALTLKGTSYKMCTDKMSFVKNPTDTGHGTAVMQVKVPKGAPCRIPVMVADDLTASVNKGILVTVNPIASTNE

DEVLIEVNPPFGDSYIIVGTGDSRLTYQWHKEGSSIGKLFTQTMKGAERLAVMGDAAWDFSSAGGFFTSVGK

GIHMVFGSAFQGLFGGLSWITKVIMGAVLIWVGINMRNMTMSMSMILVGVIMMFLSLGVGA

**>Consenso E**

AHCIGITDRDFIEGVHGGTWVSATLEQDKCVTVMAPDKPSLDISLETVAIDGPAEARKVCYSAVLTNVKINDKCPSTGEAHLEEENEGDNACKRTYSDRGWGNGCGLFGKGSIVACAKFTCAKSMSLFEVDQTKIQYVIRAQLHVGAKQENWNTDIKTLKFDALSGSQEAEFTGYGKATLECQVQTAVDFSNSYIAEMEKESWIVDKQWAQDLTLPWQSGSGGVWREMHHLVEFEPPHAATIKVLALGNQEGSLKTALTGAMRVTKDTNNSKLYKLHGGHVSCRVKLSALTLKGTSYKMCTDKMSFVKNPTDTGHGTAVMQVKVPKGAPCRIPVMVADDLTAAVNKGILVTVNPIASTNDDEVLIEVNPPFGDSYIIVGTGDSRLTYQWHKEGSSIGKLFTQTMKGAERLAVMGDAAWDFGSAGGFFTSVGKGIHTVFGSAFQGLFGGLSWITKVIMGVVLIWVGINTRNMTMSMSMILVGVIMMFLSLGVGA

**Proteina C (estrutural) 5 proteinas**

**>BeH422973|VIPR_ALG4_383464632_119_421**

MSGRKAQGKTLGVNMVRRGVRSLSSKIKQKTKQIGSRPGPSRGVQGFVFFFLFNVLTGKKITAHLKKLWRML

DPRQGLAVLKKVKRVVASLMRGLSSRKRR

**>BeH413820|VIPR_ALG4_383464630_119_421**

MSGRKAQGKTLGVNMVRQGVRSLSNKIKQKTKQIGNRPGPSRGVQGFIFFFLFNVLTGRKITAHLKRLWRML

DPRQGLAVLKKVKRVVASLMRGLSSRKRR

**>BeH394880|VIPR_ALG4_383464628_119_421**

MSGRKAQGKTLGVNMVRRGVRSLSSKIKQKTKQIGSRPGPSRGVQGFIFFFLFNVLTGKKITAHLKKLWRML

DPRQGLAVLKKVKRVVASLMRGLSSRKRR

>BeH526722|VIPR_ALG4_383464640_119_421

MSGRKAQGRTLGVNMVRRGVRSLSSKIKQKTKQIGSRPGPSRGVQGFVFFFLFNVLTGKKITAHLKKLWRML

DPRQGLAVLKKVKRVVASLMRGLSSRKRR

**>88/1999|VIPR_ALG4_586947712_119_421**

MSGRKAQGKTLGVNMVRQGVRSLSNKIKQKTKQIGNRPGPSRGVQGFIFFFLFNVLTGRKITAHLKKLWRML

DPRQGLAVLKKVKRVVASLMRGLSSRKRR

**>Consenso C**

MSGRKAQGKTLGVNMVRRGVRSLSSKIKQKTKQIGSRPGPSRGVQGFIFFFLFNVLTGKKITAHLKKLWRML

DPRQGLAVLKKVKRVVASLMRGLSSRKRR

**Proteina ns1 (não estrutural) 9 proteinas**

>case_#1|VIPR_ALG4_256274852_2453_3508

DQGCAINFGKRELKCGDGIFIFRDSDDWLNKYSYYPEDPVKLASIVKASFEEGKCGLNSVDSLEHEMWRSRA

DEINAIFEENEVDISVVVQDPKNVYQRGTHPFSRIRDGLQYGWKTWGKNLVFSPGRKNGSFIIDGKSRKECP

FSNRVWNSFQIEEFGTGVFTTRVYMDAVFEYTIDCDGSILGAAVNGKKSAHGSPTFWMGSHEVNGTWMIHTL

EALDYKECEWPLTHTIGTSVEESEMFMPRSIGGPVSSHNHIPGYKVQTNGPWMQVPLEVKREACPGTSVIID

GNCDGRGKSTRSTTDSGKVIPEWCCRSCTMPPVSFHGSDGCWYPMEIRPRKTHESHLVRSWVTA

>BeH622205|VIPR_ALG4_383464642_2453_3508

DQGCAINFGKRELKCGDGIFVFRDSDDWLNKYSYYPEDPVKLASIVKASFEEGKCGLNSVDSLEHEMWRSRA

DEINAILEENEVDISVVVQDSKNIYQRGTHPFSRIRDGLQYGWKTWGKNLVFSPGRKNGSFIIDGKSRKECP

FSNRVWNSFQIEEFGTGVFTTRVYMDAVFEYTMDCDGSILGAAVNGKKSAHGSPTFWMGSHEVNGTWMIHTL

ETLDYKECEWPLTHTIGTSVEESDMFMPRSIGGPVSSHNHIPGYKVQTNGPWMQVPLEVKREACPGTSVVVD

GGCDGRGKSTRSTTDSGKIIPEWCCRSCTMPPVSFHGSDGCWYSMEIRPKKTHDSHLVRSWVTA

>BeH423602|VIPR_ALG4_383464634_2453_3508

DQGCAINFGKRELKCGDGIFVFRDSDDWLSKYSYYPEDPVKLASIVKASFEEGKCGLNSVDSLEHEMWRSRA

DEINAILEENEVDISVVVQDSKNIYQRGTHPFSRIRDGLQYGWKTWGKNLVFSPGRKNGSFIIDGKSRKECP

FSNRVWNSFQIEEFGTGVFTTRVYMDAVFEYTMDCDGSILGAAVNGKKSAHGSPTFWMGSHEVNGTWMIHTL

ETLDYKECEWPLTHTIGTSVEESDMFMPRSIGGPVSSHNHIPGYKVQTNGPWMQVPLEVKREACPGTSVVVD

GGCDGRGKSTRSTTDSGKIIPEWCCRSCTMPPVSFHGSDGCWYPMEIRPKKTHDSHLVRSWVTA

>BeH422973|VIPR_ALG4_383464632_2453_3508

DQGCAINFGKRELKCGDGIFVFRDSDDWLNKYSYYPEDPVKLASIVKASFEEGKCGLNSVDSLEHEMWRSRA

DEINAILEENEVDISVVVQDSKNIYQRGTHPFSRIRDGLQYGWKTWGKNLVFSPGRKNGSFIIDGKSRKECP

FSNRVWNSFQIEEFGTGVFTTRVYMDAVFEYTMDCDGSILGAAVNGKKSAHGSPTFWMGSHEVNGTWMIHTL

ETLDYKECEWPLTHTIGTSVEESDMFMPRSIGGPVSSHNHIPGYKVQTNGPWMQVPLEVKREACPGTSVVVD

GGCDGRGKSTRSTTDSGKIIPEWCCRSCTMPPVSFHGSDGCWYPMEIRPKKTHDSHLVRSWVTA

>BeH413820|VIPR_ALG4_383464630_2453_3508

DQGCAINFGKRELKCGDGVFIFRDSDDWLNKYSYYPEDPVKLASIVKASFEEGKCGLNSVDSLEHEMWRSRA

DEINAILEENEVDISVVVQDPKNIYQRGTHPFSRIRDGLQYGWKTWGKNLVFSPGRKNGSFIIDGKSRKECP

FSNRVWNSFQIEEFGTGVFTTRVYMDAVFEYTMDCDGSILGAAVNGKKSAHGSPTFWMGSHEVNGTWMIHTL

ETLDYKECEWPLTHTIGTSVEESDMFMPRSIGGPVSSHNHIPGYKVQTNGPWMQVPLEVKREACPGTSVVVD

GGCDGRGKSTRSTTDSGKIIPEWCCRSCTMPPVSFHGSDGCWYPMEIRPRKTHDNHLVRSWVTA

>BeH463676|VIPR_ALG4_383464636_2453_3508

DQGCAINFGKRELKCGDGIFVFRDSDDWLSKYSYYPEDPVKLASIVKASFEEGKCGLNSVDSLEHEMWRSRA

DEINAILEENEVDISVVVQDSKNIYQRGTHSFSRIRDGLQYGWKTWGKNLVFSPGRKNGSFIIDGKSRKECP

FSNRVWNSFQIEEFGTGVFTTRVYMDAVFEYTMDCDGSILGAAVNGKKSAHGSPTFWMGSHEVNGTWMIHTL

ETLDYKECEWPLTHTIGTSVEESDMFMPRSIGGPVSSHNHIPGYKVQTNGPWMQVPLEVKREACPGTSVVVD

GGCDGRGKSTRSTTDSGKIIPEWCCRSCTMPPVSFHGSDGCWYPMEIRPKKTHDSHLVRSWVTA

>BeH394880|VIPR_ALG4_383464628_2453_3508

DQGCAINFGKRELKCGDGIFIFRDSDDWLNKYSYYPEDPVKLASIVKASFEEGKCGLNSVDSLEHEMWRSRA

DEINAILEENEVDISVVVQDSKNIYQRGTHPFSRIRDGLQYGWKTWGKNLVFSPGRKNGSFIIDGKSRKECP

FSNRVWNSFQIEEFGTGVFTTRVYMDAVFEYTMDCDGSILGAAVNGKKSAHGSPTFWMGSHEVNGTWMIHTL

ETLDYKECEWPLTHTIGTSVEESDMFMPRSIGGPVSSHNHIPGYKVQTNGPWMQVPLEVKREACPGTNVVVD

GGCDGRGKSTRSTTDSGKIIPEWCCRSCTMPPVSFHGSDGCWYPMEIRPKKTHDSHLVRSWVTA

>88/1999|VIPR_ALG4_586947712_2453_3508

DQGCAINFGRRELKCGDGVFIFRDSDDWLNKYSYYPEDPVKLASIVKASFEEGKCGLNSVDSLEHEMWRSRA

DEINAILEENEVDISVVVQDPKNIYQRGTHPFSRIRDGLQYGWKTWGKNLVFSPGRKNGSFIIDGKSRKECP

FSNRVWNSFQIEEFGTGVFTTRVYMDAVFEYTMDCDGSILGAAVNGKKSAHGSPTFWMGSHEVNGTWMIHTL

ETLDYKECEWPLTHTIGTSVEESDMFMPRSIGGPVSSHNHIPGYKVQTNGPWMQVPLEVKREACPGTSVVVD

GGCDGRGKSTRSTTDSGKIIPEWCCRSCTMPPVSFHGSDGCWYPMEIRPRKTHDNHLVRSWVTA

>H199|VIPR_ALG4_AVQ67777_1_2453_3508

DQGCAINFGKRELKCGDGIFVFRDSDDWLNKYSYYPEDPVKLASIVKSSFEEGKCGLNSVDSLEHEMWRSRA

DEINAILEENEVDISVVVQDSKNIYQRGTHPFSRIRDGLQYGWKTWGKNLVFSPGRKNGSFIIDGKSRKECP

FSNRVWNSFQIEEFGTGVFTTRVYMDAVFEYTMDCDGSILGAAVNGKKSAHGSPTFWMGSHEVNGTWMIHTL

ETLDYKECEWPLTHTIGTSVEESDMFMPRSIGGPVSSHNHIPGYKVQTNGPWMQVPLEVKREACPGTSVVVD

GGCDGRGKSTRSTTDSGKIIPEWCCRSCTMPPVSFHGSDGCWYPMEIRPKKTHDSHLVRSWVTA

**>Consenso NS1**

DQGCAINFGKRELKCGDGIFVFRDSDDWLNKYSYYPEDPVKLASIVKASFEEGKCGLNSVDSLEHEMWRSRADEINAILEENEVDISVVVQDSKNIYQRGTHPFSRIRDGLQYGWKTWGKNLVFSPGRKNGSFIIDGKSRKECPFSNRVWNSFQIEEFGTGVFTTRVYMDAVFEYTMDCDGSILGAAVNGKKSAHGSPTFWMGSHEVNGTWMIHTLETLDYKECEWPLTHTIGTSVEESDMFMPRSIGGPVSSHNHIPGYKVQTNGPWMQVPLEVKREACPGTSVVVDGGCDGRGKSTRSTTDSGKIIPEWCCRSCTMPPVSFHGSDGCWYPMEIRPKKTHDSHLVRSWVTA

**Proteina ns2a (não estrutural) 10 proteinas**

**>case_#1|VIPR_ALG4_256274852_3509_4180**

GEIHAVPFGLVSMMIAMEVVLRKRQGPKQMLVGGVVLLGAMLVGQVTLLDLLKLTVAVGLHFHEMNNGGDAMYMALIAAFSIRPGLLIGFGLRTLWSPRERLVLTLGAAMVEIALGGVMGGLWKYLNAVSLCILTINAVASRKASNTILPLMALLTPVTMAEVRLAAMLFCAVVIIGVLHQNFKDTSMQKTIPLVALTLTSYLGLTQPFLGLCAFLATRIFGRR

**>BeH622205|VIPR_ALG4_383464642_3509_4180**

GEIHAVPFGLVSMMIAMEVVLRKRQGPKQILVGGMVLLGAMLVGQVTILDLLKLTVAVGLHFHEMNNGGDAMYMALIAAFSIRPGLLIGFGLRTLWSPRERLVLTLGAAMVEIALGGMMGGLWKYLNAVSLCILTINAVASRKASNVILPLMALLTPVTMAEVRLATMLFCTVVIIGVLHQNSKDTSMQKTIPLVALTLTSYLGLTQPFLGLCAFMATRIFGRR

**>BeH423602|VIPR_ALG4_383464634_3509_4180**

GEIHAIPFGLVSMMIAMEVVLRKRQGPKQILVGGMVLLGAMLVGQVTILDLLKLTVAVGLHFHEMNNGGDAMYMALIAAFSIRPGLLIGFGLRTLWSPRERLVLTLGAAMVEIALGGMMGGLWKYLNAVSLCILTINAVASRKASNVILPLMALLTPVTMAEVRLATMLFCTVVIIGILHQNSKDTSMQKTIPLVALTLTSYLGLTQPFLGLCAFMATRIFGRR

**>BeH655417|VIPR_ALG4_383464648_3509_4180**

GEVHAIPFGLVSMMIAMEVVLRKRQGPKQILVGGMVLLGAMLVGQVTVLDLLKLTVAVGLHFHEMNNGGDAMYMALIAAFSIRPGLLIGFGLRTLWSPRERLVLTLGAAMVEIALGGMMGGLWKYLNAVSLCILTINAVASRKASNVILPLMALLTPVTMAEVRLATMLFCTVVIIGVLHQNSKDTSMQKTIPLVALTLTSYLGLTQPFLGLCAFMATRIFGRR

**>BeH422973|VIPR_ALG4_383464632_3509_4180**

GEIHAIPFGLVSMMIAMEVVLRKRQGPKQILVGGMVLLGAMLVGQVTILDLLKLTVAVGLHFHEMNNGGDAMYMALIAAFSIRPGLLIGFGLRTLWSPRERLVLTLGAAMVEIALGGMTGGLWKYLNAVSLCILTINAVASRKASNVILPLMALLTPVTMAEVRLATMLFCTVVIIGVLHQNSKDTSMQKTIPLVALTLTSYLGLTQPFLGLCAFMATRIFGRR

**>BeH413820|VIPR_ALG4_383464630_3509_4180**

GEVHAVPFGLVSMMIAMEVVLKKRQGPKQILVGGIVLLGAMLVGQVTLLDLLKLTVAVGLHFHEMNNGGDAMYMALIAAFSIRPGLLIGFGLRTLWSPRERLVLTLGAAMVEIALGGMMGGLWKYLNAVSLCILTINAVASRKASNAILPLMALLTPVTMAEVRLATMLFCTVVIIGVLHQNSKDTSMQKTIPLVALTLTSYLGLTQPFLGLCAFMATRIFGRR

**>BeH394880|VIPR_ALG4_383464628_3509_4180**

GEIHAIPFGLVSMMIAMEVVLRKRQGPKQILVGGMVLLGAMLVGQVTILDLLKLTVAVGLHFHEMNNGGDAMYMALITAFSIRPGLLIGFGLRTLWSPRERLVLTLGAAMVEIALGGMMGGLWKYLNAVSLCVLTINAVASRKASNVILPLMALLTPVTMAEVRLATMLFCTVVIIGVLHQNSKDTSMQKTIPLVALTLTSYLGLTQPFLGLCAFMATRIFGRR

**>BeH526722|VIPR_ALG4_383464640_3509_4180**

GEIHAIPFGLVSMMITMEVVLRKRQGPKQILVGGMVLLGAMLVGQVTILDLLKLTVAVGLHFHEMNSGGDAMYMALIAAFSIRPGLLIGFGLRTLWSPRERLVLTLGAAMVEIALGGMMGGLWKYLNAVSLCILTINAVASRKASNVILPLMALLTPVTMVEVRLATMLFCTVVIIGILHQNSKDTSMQKTIPLVALTLTSYLGLTQPFLGLCAFMATRIFGRR

**>88/1999|VIPR_ALG4_586947712_3509_4180**

GEVHAVPFGLVSMMIAMEVFLKRRQGPKQILVGGIVLLGAMLVGQVTLLDLLKLVVAVGLHFHEMNNGGDAMYMALIAAFSIRPGLLIGFGLRTLWSPRERLVLAFGAAMVEIALGGMMGGLWKYLNAVSLCILTINAVASRKASSAILPLMALLTPVTMAEVRLATMLFCTVVIIGVLHQNSKDTSMQKTMPLVALTLTSYLGLTQPFLGLCAFMATRIFGRR

**>H190|VIPR_ALG4_AVQ94369_1_3509_4180**

GEIHAIPFGLVSMMIAMEVVLRKRQGPKQILVGGMVLLGAMLVGQVTILDLLKLTVAVGLHFHEMNNGGDAMYMALIAAFSIRPGLLIGFGLRTLWSPRERLVLTLGAAMVEIALGGMMGGLWKYLNAVSLCILTINAVASRKASNVILPLMALLTPVTMAEVRLATMLFCTVVIIGVLHQNSKDTSMQKTIPLVALTLTSYLGLTQPFLGLCAFMATRIFGRR

**>Consenso NS2A**

GEIHAIPFGLVSMMIAMEVVLRKRQGPKQILVGGMVLLGAMLVGQVTILDLLKLTVAVGLHFHEMNNGGDAMYMALIAAFSIRPGLLIGFGLRTLWSPRERLVLTLGAAMVEIALGGMMGGLWKYLNAVSLCILTINAVASRKASNVILPLMALLTPVTMAEVRLATMLFCTVVIIGVLHQNSKDTSMQKTIPLVALTLTSYLGLTQPFLGLCAFMATRIFGRR

**Proteina ns2b (não estrutural) 6 proteinas**

>case_#1|VIPR_ALG4_256274852_4181_4570

SIPVNEALAAAGLVGVLAGLAFQEMENFLGPIAVGGLLMMLVSVAGRVDGLELKKLGEVSWEEEAEISGSSARYDVALSEQGEFKLLSEEKVPWDQVVMTSLALVGAALHPFALLLVLAGWLFHVRGARR

>BeH423602|VIPR_ALG4_383464634_4181_4570

SIPVNEALAAAGLVGVLAGLAFQEMENFLGPVAVGGILMMLVSVAGRVDGLELKKLGEVSWEEEAEISGSSARYDVTLSEQGEFKLLSEEKVPWDQVVMTSLALVGAAIHPFALLLVLAGWLLHVKGARR

>BeH413820|VIPR_ALG4_383464630_4181_4570

SIPVNEALAAAGLVGVLAGLAFQEMENFLGPVAVGGILMMLVSVAGRVDGLELRKLGEVSWEEEAEISGSSARYDVALSEQGEFKLLSEEKVPWDQIVMTSLALVGAAIHPFALMLVLAGWLLHVKGTRR

>BeH463676|VIPR_ALG4_383464636_4181_4570

SIPVNEALAAAGLVGVLAGLAFQEMENFLGPVAVGGILMMLVSVAGRVDGLELKKLGEVSWEEEAEISGSSARYDVTLSEQGEFKLLSEEKVPWDQVVMTSLTLVGAAIHPFALLLVLAGWLLHVKGARR

>BeH394880|VIPR_ALG4_383464628_4181_4570

SIPVNEALAAAGLVGVLAGLAFQEMENFLGPVAVGGILMMLVSVAGRVDGLELRKLGEVSWEEEAEISGSSARYDVTLSEQGEFKLLSEEKVPWDQVVMTSLALVGAAIHPFALLLVLAGWLFHVKGARR

>88/1999|VIPR_ALG4_586947712_4181_4570

SIPVNEALAAAGLVGVLAGLAFQEMENFLGPVAVGGILMMLISVAGRVDGLELRKLGEVSWEEEAEISGSSARYDVALSEQGEFKLLSEEKIPWDQIVMTSLALVGAAIHPFALMLVLAGWLLHVKGARR

**>Consenso NS2B**

SIPVNEALAAAGLVGVLAGLAFQEMENFLGPVAVGGILMMLVSVAGRVDGLELKKLGEVSWEEEAEISGSSARYDVALSEQGEFKLLSEEKVPWDQVVMTSLALVGAAIHPFALLLVLAGWLLHVKGARR

**Proteina ns3 (não estrutural) 10 proteinas**

>case_#1|VIPR_ALG4_256274852_4571_6439

SGDVLWDIPTPKIIEECEHLEDGIYGIFQSTFLGASQRGVGVAQGGVFHTMWHVTRGAFLVRNGKKLIPSWASVKEDLVAYGGSWKLEGRWDGEEEVQLIAAVPGKNVVNVQTKPSLFKVRNGGEIGAVALDYPSGTSGSPIVNRNGEVIGLYGNGILVGDNSFVSAISQTEVKEEGKEELREIPTMLKKGMTTILDFHPGAGKTRRFLPQILAECARRRLRTLVLAPTRVVLSEMKEAFHGLDVKFHTQAFSAHGSGREVIDAMCHATLTYRMLEPTRVVNWEVIIMDEAHFLDPASIAARGWAAHRARANESATILMTATPPGTSDEFPHSNGEIEDVQTDIPSEPWNTGHDWILADKRPTAWFLPSIRAANVMAASLRKAGKSVVVLNRKTFEREYPTIKQKKPDFILATDIAEMGANLCVERVLDCRTAFKPVLVDEGRKVAIKGPLRISASSAAQRRGRIGRNPNRDGDSYYYSEPTSENNAHHVCWLEASMLLDNMEVRGGMVAPLYGVEGTKTPVSPGEMRLRDDQRKVFRELVRNCDLPVWLSWQVAKAGLKTNDRKWCFEGPEEHEILNDSGETVKCRAPGGAKKPLRPRWCDERVSSDQSALSEFIKFAEGRR

>BeH622205|VIPR_ALG4_383464642_4571_6439

SGDVLWDIPTPKIIEECEYLEDGIYGIFQSTFLGASQRGVGVAQGGVFHTMWHVTRGAFLVRNGKKLVPSWASVKEDLVAYGGSWKLEGRWDGEEEVQLIAAAPGKNVVNVQTKPSLFKVRNGGEIGAVALDYPSGTSGSPIVNRNGEVIGLYGNGILVGDNSFVSAISQTEVKEEGKEELQEIPTMLKKGMTTILDFHPGAGKTRRFLPQILAECTRRRLRTLVLAPTRVVLSEMKEAFHGLDVKFHTQAFSAHGSGKEVIDAMCHATLTYRMLEPTRVVNWEVIIMDEAHFLDPASIAARGWAAHRARANESATILMTATPPGTSDEFPHSNGEIEDVQTDIPSEPWNTGHDWILADKRPTAWFLPSIRAANVMAASLRKAGKSVVVLNRKTFEKEYPTIKQKKPDFILATDIAEMGANLCVERVLDCRTAFKPVLVDEGRKVAIKGPLRISASSAAQRRGRIGRNPNRDGDSYYYSEPTSEDNAHHVCWLEASMLLDNMEVRGGMVAPLYGIEGTKTPVSPGEMRLRDDQRRVFRELVRNCDLPVWLSWQVAKAGLKTNDRKWCFEGPEEHEILNDSGETVKCRAPGGAKKPLRPRWCDERVSSDQSALADFIKFAEGRR

>BeH423602|VIPR_ALG4_383464634_4571_6439

SGDVLWDIPTPKIIEECEYLEDGIYGIFQSTFLGASQRGVGVAQGGVFHTMWHVTRGAFLVRNGKKLVPSWASVKEDLVAYGGSWKLEGRWDGEEEVQLIAAAPGKNVVNVQTKPSLFKVRNGGEIGAVALDYPSGTSGSPIVNRNGEVIGLYGNGILVGDNSFVSAISQTEVKEEGKEELQEIPTMLKKGMTTILDFHPGAGKTRRFLPQILAECARRRLRTLVLAPTRVVLSEMKEAFHGLDVKFHTQAFSAHGSGKEVIDAMCHATLTYRMLEPTRVVNWEVIIMDEAHFLDPASIAARGWAAHRARANESATILMTATPPGTSDEFPHSNGEIEDVQTDIPSEPWNTGHDWILADKRPTAWFLPSIRAANVMAASLRKAGKSVVVLNRKTFEKEYPTIKQKKPDFILATDIAEMGANLCVERVLDCRTAFKPVLVDEGRKVAIKGPLRISASSAAQRRGRIGRNPNRDGDSYYYSEPTSEDNAHHVCWLEASMLLDNMEVRGGMVAPLYGIEGTKTPVSPGEMRLRDDQRRVFRELVRNCDLPVWLSWQVAKAGLKTNDRKWCFEGPEEHEILNDSGETVKCRAPGGAKKPLRPRWCDERVSSDQSALADFIKFAEGRR

>BeH655417|VIPR_ALG4_383464648_4571_6439

SGDVLWDIPTPKIIEECEYLEDGIYGIFQSTFLGASQRGVGVAQGGVFHTMWHVTRGAFLVRNGKKLVPSWASVKEDLVAYGGSWKLEGRWDGEEEVQLIAAAPGKNVVNVQTKPSLFKVRNGGEIGAVALDYPSGTSGSPIVNRNGEVIGLYGNGILVGDNSFVSAISQTEVKEEGKEELQEIPTMLKKGMTTILDFHPGAGKTRRFLPQILAECARRRLRTLVLAPTRVVLSEMKEAFHGLDVKFHTQAFSAHGSGKEVIDAMCHATLTYRMLEPTRVVNWEVIIMDEAHFLDPASIAARGWAAHRARANESATILMTATPPGTNDEFPHSNGEIEDVQTDIPSEPWNTGHDWILADKRPTAWFLPSIRAANVMAASLRKAGKSVVVLNRKTFEKEYPTIKQKKPDFILATDIAEMGANLCVERVLDCRTAFKPVLVDEGRKVAIKGPLRISASSAAQRRGRIGRNPNRDGDSYYYSEPTSEDNAHHVCWLEASMLLDNMEVRGGMVAPLYGIEGTKTPVSPGEMRLRDDQRRVFRELVRNCDLPVWLSWQVAKAGLKTNDRKWCFEGPEEHEILNDSGETVKCRAPGGAKKPLRPRWCDERVSSDQSALADFIKFAEGRR

>BeH422973|VIPR_ALG4_383464632_4571_6439

SGDVLWDIPTPKIIEECEYLEDGIYGIFQSTFLGASQRGVGVAQGGVFHTMWHVTRGAFLVRNGKRLVPSWASVKEDLVAYGGSWKLEGRWDGEEEVQLIAAAPGKNVVNVQTKPSLFKVRNGGEIGAVALDYPSGTSGSPIVNRNGEVIGLYGNGILVGDNSFVSAISQTEVKEEGKEELQEIPTMLKKGMTTILDFHPGAGKTRRFLPQILAECARRRLRTLVLAPTRVVLSEMKEAFHGLDVKFHTQAFSAHGSGKEVIDAMCHATLTYRMLEPTRVVNWEVIIMDEAHFLDPASIAARGWAAHRARANESATILMTATPPGTSDEFPHSNGEIEDVQTDIPSEPWNTGHDWILADKRPTAWFLPSIRAANVMAASLRKAGKSVVVLNRKTFEKEYPTIKQKKPDFILATDIAEMGANLCVERVLDCRTAFKPVLVDEGRKVAIKGPLRISASSAAQRRGRIGRNPNRDGDSYYYSEPTSEDNAHHVCWLEASMLLDNMEVRGGMVAPLYGIEGTKTPVSPGEMRLRDDQRRVFRELVRNCDLPVWLSWQVAKAGLKTNDRKWCFEGPEEHEILNDSGETVKCRAPGGAKKPLRPRWCDERVSSDQSALADFIKFAEGRR

>BeH413820|VIPR_ALG4_383464630_4571_6439

SGDVLWDIPAPTIVEECEHLEDGIYGIFQSTFLGASQRGVGVAQGGVFHTMWHVTRGAFLVRNGKKLVPTWASVKEDLVAYGGSWKLEGRWDGEEEVQLIAAAPGKNVVNVQTKPSLFKVRNGGEIGAVALDYPSGTSGSPIVNRNGEVIGLYGNGILVGDNSFVSAISQTEAKEEGKEELQEIPTMLKKGMTTILDFHPGAGKTRRFLPQILAECARRRLRTLVLAPTRVVLSEMKEAFHGLDVKFHTQAFSAHGSGKEVIDAMCHATLTYRMLEPTRVVNWEVIIMDEAHFLDPASIAARGWAAHRARANESATILMTATPPGTSDEFPHSNGEIEDVQTDIPSEPWNTGHDWILADKRPTAWFLPSIRAANVMAASLRKAGKSVVVLNRKTFEKEYPTIKQKKPDFILATDIAEMGANLCVERVLDCRTAFKPVLVDEGRKVAIKGPLRISASSAAQRRGRIGRNPNRDGDSYYYSEPTSEDNAHHVCWLEASMLLDNMEVRGGMVAPLYGIEGTKTPVSPGEMRLRDDQRRVFRELVRNCDLPVWLSWQVAKAGLKTNDRKWCFEGPEEHEILNDSGETVKCRAPGGAKRPLRPRWCDERVSSDQSALAEFIKFAEGRR

>BeH394880|VIPR_ALG4_383464628_4571_6439

SGDVLWDIPTPKIIEECEYLEDGIYGIFQSTFLGASQRGVGVAQGGVFHTMWHVTRGAFLVRNGKKLVPSWASVKEDLVAYGGSWKLEGRWDGEEEVQLIAAAPGKNVVNVQTKPSLFKVRNGGEIGAVALDYPSGTSGSPIVNRNGEVIGLYGNGILVGDNSFVSAISQTEVKEEGKEELQEIPTMLKKGMTTILDFHPGAGKTRRFLPQILAECARRRLRTLVLAPTRVVLSEMKEAFHGLDVKFHTQAFSAHGSGKEVIDAMCHATLTYRMLEPTRVVNWEVIIMDEAHFLDPASIAARGWAAHRARANESATILMTATPPGTSDEFPHSNGEIEDVQTDIPSEPWNTGHDWILADKRPTAWFLPSIRAANVMAASLRKAGKSVVVLNRKTFEKEYPTIKQKKPDFILATDIAEMGANLCVERVLDCRTSFKPVLVDEGRKVAIKGPLRISASSAAQRRGRIGRNPNRDGDSYYYSEPTSEDNAHHVCWLEASMLLDNMEVRGGMVAPLYGIEGTKTPVSPGEMRLRDDQRRVFRELVRNCDLPVWLSWQVAKAGLKTNDRKWCFEGPEEHEILNDSGETVKCRAPGGAKKPLRPRWCDERVSSDQSALADFIKFAEGRR

>BeH526722|VIPR_ALG4_383464640_4571_6439

SGDVLWDIPTPKIIEECEYLEDGIYGIFQSTFLGASQRGVGVAQGGVFHTMWHVTRGAFLVRNGKKLVPSWASVKEDLVAYGGSWKLEGRWDGEEEVQLIAAAPGKNVVNVQTKPSLFKVRNGGEIGAVALDYPSGTSGSPIVNRNGEVIGLYGNGILVGDNSFVSAISQTEVKEEGKEELQEIPTMLKKGMTTILDFHPGAGKTRRFLPQILAECARRRLRTLVLAPTRVVLSEMKEAFHGLDVKFHTQAFSAHGSGKEVIDAMCHATLTYRMLEPTRVVNWEVIIMDEAHFLDPASIAARGWAAHRARANESATILMTATPPGTSDEFPHSNGEIEDVQTDIPSEPWNTGHDWILADKRPTAWFLPSIRAANVMAASLRKAGKSVVVLNRKTFEKEYPTIKQKKPDFILATDIAEMGANLCVERVLDCRTAFKPVLVDEGRKVAIKGPLRISASSAAQRRGRIGRNPNRDGDSYYYSEPTSEDNAHHVCWLEASMLLDNMEVRGGMVAPLYGIEGTKTPASPGEMRLRDDQRRVFRELVRNCDLPVWLSWQVAKAGLKTNDRKWCFEGPEEHEILNDSGETVKCRAPGGAKKPLRPRWCDERVSSDQSALADFIKFAEGRR

>88/1999|VIPR_ALG4_586947712_4571_6439

SGDVLWDIPAPTIVEECEHLEDGIYGIFQSTFLGASQRGVGVAQGGVFHTMWHVTRGAFLVRNGKKLVPTWASVKEDLVAYGGSWKLEGRWDGEEEVQLIAAAPGKNVVNVQTKPSLFKVRNGGEIGAVALDYPSGTSGSPIVNRNGEVIGLYGNGILVGDNSFVSAISQTEAKEEGREELQEIPTMLKKGMTTILDFHPGAGKTRRYLPQILAECARRRLRTLVLAPTRVVLSEMKEAFHGLDVKFHTQAFSAHGSGKEVIDAMCHATLTYRMLEPTRVVNWEVIIMDEAHFLDPASIAARGWAAHRARANESATILMTATPPGTGDEFPHSNGEIEDVQTDIPSEPWNTGHDWILADKRPTAWFLPSIRAANVMAASLRKAGKSVVVLNRKTFEKEYPTIKQKKPDFILATDIAEMGANLCVERVLDCRTAFKPVLVDEGRKVAIKGPLRISASSAAQRRGRIGRNPNRDGDSYYYSEPTSEDNAHHVCWLEASMLLDNMEVRGGMVAPLYGIEGTKTPVSTGEMRLRDDQRRVFRELVRNCDLPVWLSWQVAKAGLKTNDRKWCFEGPEEHEILNDSGETVKCRAPGGAKRPLRPRWCDERVSSDQSALAEFIKFAEGRR

>H190|VIPR_ALG4_AVQ94369_1_4571_6439

SGDVLWDIPTPKIIEECEYLEDGIYGIFQSTFLGASQRGVGVAQGGVFHTMWHVTRGAFLVRNGKKLVPSWASVKEDLVAYGGSWKLDGRWDGEEEVQLIAAAPGKNVVNVQTKPSLFKVKNGGEIGAVALDYPSGTSGSPIVNRNGEVIGLYGNGILVGDNSFVSAISQTEVKEEGKEELQEIPTMLKKGMTTILDFHPGAGKTRRFLPQILAECARRRLRTLVLAPTRVVLSEMKEAFHGLDVKFHTQAFSAHGSGKEVIDAMCHATLTYRMLEPTRVVNWEVIIMDEAHFLDPASIAARGWAAHRARANESATILMTATPPGTSDEFPHSNGEIEDVQTDIPSEPWNTGHDWILADKRPTAWFLPSIRAANVMAASLRKAGKSVVVLNRKTFEKEYPTIKQKKPDFILATDIAEMGANLCVERVLDCRTAFKPVLVDEGRKVAIKGPLRISASSAAQRRGRIGRNPNRDGDSYYYSEPTSEDNAHHVCWLEASMLLDNMEVRGGMVAPLYGIEGTKTPVSPGEMRLRDDQRRVFRELVRNCDLPVWLSWQVAKAGLKTNDRKWCFEGPEEHEILNDSGETVKCRAPGGAKKPLRPRWCDERVSSDQSALADFIKFAEGRR

**>Consenso NS3**

SGDVLWDIPTPKIIEECEYLEDGIYGIFQSTFLGASQRGVGVAQGGVFHTMWHVTRGAFLVRNGKKLVPSWASVKEDLVAYGGSWKLEGRWDGEEEVQLIAAAPGKNVVNVQTKPSLFKVRNGGEIGAVALDYPSGTSGSPIVNRNGEVIGLYGNGILVGDNSFVSAISQTEVKEEGKEELQEIPTMLKKGMTTILDFHPGAGKTRRFLPQILAECARRRLRTLVLAPTRVVLSEMKEAFHGLDVKFHTQAFSAHGSGKEVIDAMCHATLTYRMLEPTRVVNWEVIIMDEAHFLDPASIAARGWAAHRARANESATILMTATPPGTSDEFPHSNGEIEDVQTDIPSEPWNTGHDWILADKRPTAWFLPSIRAANVMAASLRKAGKSVVVLNRKTFEKEYPTIKQKKPDFILATDIAEMGANLCVERVLDCRTAFKPVLVDEGRKVAIKGPLRISASSAAQRRGRIGRNPNRDGDSYYYSEPTSEDNAHHVCWLEASMLLDNMEVRGGMVAPLYGIEGTKTPVSPGEMRLRDDQRRVFRELVRNCDLPVWLSWQVAKAGLKTNDRKWCFEGPEEHEILNDSGETVKCRAPGGAKKPLRPRWCDERVSSDQSALADFIKFAEGRR

**Proteina ns4a (proteina não estrutural) 7 proteinas**

>case_#1|VIPR_ALG4_256274852_6440_6817

GAAEVLVVLSELPDFLAKKGGEAMDTISVFLHSEEGSRAYRNALSMMPEAMTIVMLFILAGLLTSGMVIFFMSPKGISRMSMAMGTMAGCGYLMFLGGVKPTHISYIMLIFFVLMVVVIPEPGQQR

>BeH423602|VIPR_ALG4_383464634_6440_6817

GAAEMLVVLSELPDFLAKKGGEAVDTISVFLHSEEGSRAYRNALSMMPEAMTTVMLFVLAGLLTSGMVIFFMSPKGISRMSMAMGTMAGCGYLMFLGGVKPTHISYIMLIFFVLMVVVVPEPGQQR

>BeH422973|VIPR_ALG4_383464632_6440_6817

GAAEMLVVLSELPDFLAKKGGEAVDTISVLLHSEEGSRAYRNALSMMPEAMTTVMLFVLAGLLTSGMVIFFMSPKGISRMSMAMGTMAGCGYLMFLGGVKPTHISYIMLIFFVLMVVVVPEPGQQR

>BeH413820|VIPR_ALG4_383464630_6440_6817

GAAEVLVVLSELPDFLAKKGGEAMDTISVFLNSEEGSRAYRNALSMMPEAMTIVMLFVLAGLLTSGMVIFFMSPKGISRMSMAMGTMAGCGYLMFLGGVKPTHISYIMLIFFVLMVVVVPEPGQQR

>BeH394880|VIPR_ALG4_383464628_6440_6817

GAAEVLVVLSELPDFLAKKGGEAVDTISVFLHSEEGSRAYRNALSMMPEAMTTVMLFVLAGLLTSGMVIFFMSPKGISRMSMAMGTMAGCGYLMFLGGVKPTHISYIMLIFFVLMVVVVPEPGQQR

>88/1999|VIPR_ALG4_586947712_6440_6817

GAAEVLVVLSELPDFLAKKGGEAMDTISVFLNSEEGSRAYRNALSMMPEAMTIVMLFVLAGLLTSGMVIFFMSPKGISRMSMAMGTMAGSGYLMFLGGVKPTHISYIMLIFFVLMVVVVPEPGQQR

>H191|VIPR_ALG4_AVQ94370_1_6440_6817

GAAEMLVVLSELPDFLAKKGGEAVDTISVLLHSEEGSRAYRNALSMMPEAMTTVMLFVLAGLLTSGMVVFFMSPKGISRMSMAMGTMAGCGYLMFLGGVKPTHISYIMLIFFVLMVVVVPEPGQQR

**>Consenso NS4A**

GAAEVLVVLSELPDFLAKKGGEAVDTISVFLHSEEGSRAYRNALSMMPEAMTTVMLFVLAGLLTSGMVIFFM

SPKGISRMSMAMGTMAGCGYLMFLGGVKPTHISYIMLIFFVLMVVVVPEPGQQR

**Proteina ns4b (proteina não estrutural) 5 proteinas**

>case_#1|VIPR_ALG4_256274852_6887_7636

NELGMLEKTKEDLFGKKNLIPSSASPWSWPDLDLKPGAAWTVYVGIVTMLSPMLHHWIKVEYGNLSLSGIAQSASVLSFMDKGIPFMKMNISVIMLLVSGWNSITVMPLLCGIGCAMLHWSLILPGIKAQQSKLAQRRVFHGVAKNPVVDGNPTVDIEEAPEMPALYEKKLALYLLLALSLASVAMCRTPFSLAEGIVLASAALGPLIEGNTSLLWNGPMAVSMTGVMRGNHYAFVGVMYNLWKMKTGRR

>BeH423602|VIPR_ALG4_383464634_6887_7636

NELGMLEKTKEDLFGKKNLIPSSAAPWSWPDFDLKPGAAWTVYVGIVTMLSPMLHHWIKVEYGNLSLSGIAQSASVLSFMDKGIPFMKMNISVIILLVSGWNSITVMPLLCGIGCAMLHWTLILPGIKAQQSKLAQRRVFHGVAKNPVVDGNPTVDIEEAPEMPALYEKKLALYLLLALSLASVAMCRTPFSLAEGIVLASAALGPLIEGNTSLLWNGPMAVSMTGVMRGNYYAFVGVMYNLWKMNTGRR

>BeH413820|VIPR_ALG4_383464630_6887_7636

NELGMLEKTKEDLFGKKNLIPSNTAPWNWPDLDLKPGAAWTVYVGIVTMLSPMLHHWIKVEYGNLSLSGIAQSASVLSFMDKGIPFMKMNISVIILLISGWNSITVMPLLCGIGCAMLHWTLILPGIKAQQSKLAQRRVFHGVAKNPVVDGNPTVDIEEAPEMPALYEKKLALYLLLALSLASVAMCRTPFSLAEGIVLASAALGPLIEGNTSLLWNGPMAVSMTGVMRGNYYAFVGVMYNLWKMKTGRR

>BeH394880|VIPR_ALG4_383464628_6887_7636

NELGMLEKTKEDLFGKKNLIPSSAAPWSWPDFDLKPGAAWTVYVGIVTMLSPMLHHWIKVEYGNLSLSGIAQSASVLSFMDKGIPFMKMNISVIILLVSGWNSITVMPLLCGIGCAMLHWTLILPGIKAQQSKLAQRRVFHGVAKNPVVDGNPTVDIEEAPEMPALYEKKLALYLLLALSLASVAMCRTPFSLAEGIVLASAALGPLIEGNTSLLWNGPMAVSMTGVMRGNYYAFVGVMYNLWKMKTGRR

>88/1999|VIPR_ALG4_586947712_6887_7636

NELGMLEKTKEDLFGKKNLIPSSTAPWNWPDLDLKPGAAWTVYVGIVTMLSPMLHHWIKVEYGNLSLSGIAQSASVLSFMDKGIPFMKMNISVIILLISGWNSITVMPLLCGIGCAMLHWTLILPGIKAQQSKLAQRRVFHGVAKNPVVDGNPTVDIEEAPEMPALYEKKLALYLLLALSLASVAMCRTPFSLAEGIVLASAALGPLIEGNTSLLWNGPMAVSMTGVMRGNYYAFVGVMYNLWKMNTGRR

**>Consenso NS4B**

NELGMLEKTKEDLFGKKNLIPSSAAPWSWPDLDLKPGAAWTVYVGIVTMLSPMLHHWIKVEYGNLSLSGIAQSASVLSFMDKGIPFMKMNISVIILLVSGWNSITVMPLLCGIGCAMLHWTLILPGIKAQQSKLAQRRVFHGVAKNPVVDGNPTVDIEEAPEMPALYEKKLALYLLLALSLASVAMCRTPFSLAEGIVLASAALGPLIEGNTSLLWNGPMAVSMTGVMRGNYYAFVGVMYNLWKMKTGRR

**Proteina ns5 (não estrutural) 4 proteinas**

>BeH422973|VIPR_ALG4_383464632_7637_10351

GRANGKTLGEVWKRELNLLDKQQFELYKRTDIVEVDRDTARRHLAEGKVDTGVAVSRGTAKLRWFHERGYVKLEGRVTDLGCGRGGWCYYAAAQREVSGVKGFTLGKEGHEKPMNVQSLGWNIITFKDKTDVHRLEPVKCDTLLCDIGESSPSSVTEGERTMRVLDTVEKWLGCGVESFCVKVLAPYMPDVLEKLELLQRRFGGTVIRNPLSRNSTHEMYYVSGARSNIAFTVNQTSRLLMRRMRRPTGKVTLEADVILPIGTRSVETDKGPLDRAAIEERVERIKSEYTATWFHDNDNPYRTWHYCGSYVTRTSGSAASMINGVIKILTYPWDRIEEVTRMAMTDTTPFGQQRVFKEKVDTRAKDPPAGTRKIMKVVNRWLFRHLAREKNPRLCTKEEFIAKVRSHAAIGAFLEEQEQWKTANEAVQDPKFWELVDEERRLHQQGRCRTCVYNMMGKREKKLSEFGKAKGSRAIWYMWLGARYLEFEALGFLNEDHWASRENSGGGVEGIGLQYLGYVIRDLAALEGGGFYADDTAGWDTRITEADLDDEQEILNYMSPHHRKLALAVMEMTYKNKVVKVLRPAPGGKAYMDVISRRDQRGSGQVVTYALNTITNLKVQLIRMAEAEMVIHHQHVQDCDDTVLTKLEAWLAEHGCDRLKRMAVSGDDCVVRPIDDRFGLALSHLNAMSKVRKDISEWQPSKGWDDWENVPFCSHHFHELQLKDGRRIVVPCRDQDELVGRGRVSPGNGWMIKETACLSKAYANMWSLMYFHKRDMRLLSLAVSSAVPTSWVPQGRTTWSVHGKGEWMTTEDMLEVWNRVWITNNPHMQDKTTVKEWRDIPYLTKRQDKLCGSLIGITNRATWASHIHLVIHRIRTLIGKERYTDYLTVMDRYSVDADLQPGELI

>BeH413820|VIPR_ALG4_383464630_7637_10351

GRANGKTLGEVWKRELNLLDKQQFELYKRTDIVEVDRETARRHLAEGKVDTGVAVSRGTAKLRWFHERGYVKLEGRVTDLGCGRGGWCYYAAAQKEVSGVKGFTLGKEGHEKPMNVQSLGWNIITFKDKTDIHRLEPVKCDTLLCDIGESSPSSVTEGERTMRVLDTVEKWLGCGVESFCVKVLAPYMPDVIEKLELLQRRFGGTVIRNPLSRNSTHEMYYVSGARSNITFTVNQTSRLLMRRMRRPTGKVTLEADVILPIGTRSVETDKGPLDRAAIEERVERIKSEYTATWFYDNDNPYRTWHYCGSYVTRTSGSAASMINGVIKILTYPWDRIEEVTRMAMTDTTPFGQQRVFKEKVDTRAKDPPAGTRKIMKVVNRWLFRHLAREKSPRLCTKEEFIAKVRSHAAIGAFLEEQEQWKTANEAVQDPKFWELVDEERRLHQQGRCRTCVYNMMGKREKKLSEFGKAKGSRAIWYMWLGARYLEFEALGFLNEDHWASRENSGGGVEGIGLQHLGYVIRGLAALEGGGFYADDTAGWDTRITEADLDDEQEILNYMSPHHRKLALAVMEMTYKNKVVKVLRPAPGGKAYMDVISRRDQRGSGQVVTYALNTITNLKVQLVRMAEAEMVVHHQHVQDCDDAVLTKLEAWLAEHGCDRLKRMAVSGDDCVVRPIDDRFGLALSHLNAMSKVRKDISEWQPSKGWNDWENVPFCSHHFHELQLKDGRRIVVPCRDQDELIGRGRVSPGNGWMIKETACLSKAYANMWSLMYFHKRDMRLLSLAVSSAVPTSWVPQGRTTWSVHGKGEWMTTEDMLEVWNRVWIVNNPHMQDKTTVKEWRDIPYLTKRQDKLCGSLIGMTNRATWASHIHLVIHRIRTLIGKEKYTDYLTVMDRYSVDADLQPGELI

>BeH394880|VIPR_ALG4_383464628_7637_10351

GRANGKTLGEVWKRELNLLDKQQFELYKRTDIVEVDRDTARRHLAEGKVDTGVAVSRGTAKLRWFHERGYVKLEGRVTDLGCGRGGWCYYAAAQREVSGVKGFTLGKEGHEKPMNVQSLGWNIITFKDKIDVHRLEPVKCDTLLCDIGESSSSSVTEGERTMRVLDTVEKWLGCGVESFCVKVLAPYMPDVLEKLELLQRRFGGTVIRNPLSRNSTHEMYYSGARSNIAFTVNQTSRLLMRRMRRPTGKVTLEADVVLPIGTRSVETDKGPLDRAAIEERVERIKSEYTATWFHDNDNPYRTWHYCGSYVTRTSGSAASMINGVIKILTYPWDRIEEVTRMAMTDTTPFGQQRVFKEKVDTRAKDPPAGTRKIMKVVNRWLFRHLAREKNPRLCTKEEFIAKVRSHAAIGAFLEEQEQWKTANEAVQDPKFWELVDEERRLHQQGRCRTCVYNMMGKREKKLSEFGKAKGSRAIWYMWLGARYLEFEALGFLNEDHWASRENSGGGVEGIGLQYLGYVIRDLAALEGGGFYADDTAGWDTRITEADLDDEQEVLNYMSPHHRKLALAVMEMTYKNKVVKVLRPSPGGKAYMDVISRRDQIGSGQVVTYALNTITNLKVQLIRMAEAEMVIHHQHVQDCDDAVLTKLEAWLAEHGCDRLKRMAVSGDDCVVRPIDDRFGLALSHLNAMSKVRKDISEWQPSKGWDDWENVPFCSHHFHELQLKDGRRIVVPCRDQDELVGRGRVSPGNGWMIKETACLSKAYANMWALMYFHKRDMRLLSLAVSSAVPTSWVPQGRTTWSVHGKGEWMTTEDMLEVWNRVWITNNPHMQDKTTVREWRDIPYLTKRQDKLCGSLIGMTNRATWASHIHLVIHRIRTLIGKERYTDYLTVMDRYSVDADLQPGELI

>88/1999|VIPR_ALG4_586947712_7637_10351

GRANGKTLGEVWKRELNLLDKQQFELYKRTDIVEVDRETARRHLAEGKVDTGVAVSRGTAKLRWFHERGYVRLEGRVTDLGCGRGGWCYYAAAQKEVSGVKGFTLGKEGHEKPMNVQSLGWNIITFKDKTDIHRLEPVKCDTLLCDIGESSPSSVTEGERTMRVLDTVEKWLGCGVESFCVKVLAPYMPDVLEKLELLQRRFGGTVIRNPLSRNSTHEMYYVSGARSNITFTVNQTSRLLMRRMRRPTGKVTLEADVILPIGTRSVETDKGPLDRAAIEERVERIKSEYAATWFHDNDNPYRTWHYCGSYVTRTSGSAASMINGVIKILTYPWDRIEEVTRMAMTDTTPFGQQRVFKEKVDTRAKDPPVGTRKIMKVVNRWLFRHLAREKSPRLCTKEEFIAKVRSHAAIGAFLEEQEQWKTANEAVQDPKFWELVDEERRLHQQGRCRTCVYNMMGKREKKLSEFGKAKGSRAIWYMWLGARYLEFEALGFLNEDHWASRENSGGGVEGIGLQHLGYVIRDLAALEGGGFYADDTAGWDTRITEADLDDEQEILNYMSPHHRKLALAVMEMTYKNKVVKVLRPAPGGKAYMDIISRRDQRGSGQVVTYALNTITNLKVQLIRMAEAEMVVHHQHVQDCDDAVLTKLEAWLAEHGCDRLKRMAVSGDDCVVRPIDDRFGLALSHLNAMSKVRKDISEWQPSKGWNDWENVPFCSHHFHELQLKDGRRIVVPCRDQDELIGRGRVSPGNGWMIKETACLSKAYANMWSLMYFHKRDMRLLSLAVSSAVPTSWVPQGRTTWSVHGKGEWMTTEDMLEVWNRVWIVNNPHMQDKTTVKEWRDIPYLTKRQDKLCGSLIGMTNRATWASHIHLVIHRIRTLIGKEKYTDYLTVMDRYSVDADLQPGELI

**>Consenso NS5**

GRANGKTLGEVWKRELNLLDKQQFELYKRTDIVEVDRDTARRHLAEGKVDTGVAVSRGTAKLRWFHERGYVKLEGRVTDLGCGRGGWCYYAAAQKEVSGVKGFTLGKEGHEKPMNVQSLGWNIITFKDKTDIHRLEPVKCDTLLCDIGESSPSSVTEGERTMRVLDTVEKWLGCGVESFCVKVLAPYMPDVLEKLELLQRRFGGTVIRNPLSRNSTHEMYYVSGARSNIAFTVNQTSRLLMRRMRRPTGKVTLEADVILPIGTRSVETDKGPLDRAAIEERVERIKSEYTATWFHDNDNPYRTWHYCGSYVTRTSGSAASMINGVIKILTYPWDRIEEVTRMAMTDTTPFGQQRVFKEKVDTRAKDPPAGTRKIMKVVNRWLFRHLAREKNPRLCTKEEFIAKVRSHAAIGAFLEEQEQWKTANEAVQDPKFWELVDEERRLHQQGRCRTCVYNMMGKREKKLSEFGKAKGSRAIWYMWLGARYLEFEALGFLNEDHWASRENSGGGVEGIGLQHLGYVIRDLAALEGGGFYADDTAGWDTRITEADLDDEQEILNYMSPHHRKLALAVMEMTYKNKVVKVLRPAPGGKAYMDVISRRDQRGSGQVVTYALNTITNLKVQLIRMAEAEMVIHHQHVQDCDDAVLTKLEAWLAEHGCDRLKRMAVSGDDCVVRPIDDRFGLALSHLNAMSKVRKDISEWQPSKGWDDWENVPFCSHHFHELQLKDGRRIVVPCRDQDELIGRGRVSPGNGWMIKETACLSKAYANMWSLMYFHKRDMRLLSLAVSSAVPTSWVPQGRTTWSVHGKGEWMTTEDMLEVWNRVWITNNPHMQDKTTVKEWRDIPYLTKRQDKLCGSLIGMTNRATWASHIHLVIHRIRTLIGKEKYTDYLTVMDRYSVDADLQPGELI
