# Supplementary material for: Advancing molecular modeling and reverse vaccinology in broad-spectrum yellow fever virus vaccine development
Source: Sci Rep. 2024 May 12;14:10842. doi: 10.1038/s41598-024-60680-9 (PMC11089047; doi:10.1038/s41598-024-60680-9)
Supplement: Supplementary file 1 — Supplementary Information. [file 41598_2024_60680_MOESM1_ESM.zip › Yellow_Fever_data/2_Prediction of T-cell epitopes/propred/propred - e.docx]

##### **Allele No: 1 Name: HLAA1**

LVEFEPPHA

##### **Allele No: 2 Name: HLAA2**

VMAPDKPSL

IKTLKFDAL

TKDTNNSKL

VMVADDLTA

##### **Allele No: 3 Name: HLAA*0201**

VMAPDKPSL

VMVADDLTA

LVGVIMMFL
SLDISLETV

##### **Allele No: 4 Name: HLAA*0205**

VMAPDKPSL

ALGNQEGSL

LVGVIMMFL

##### **Allele No: 5 Name: HLAA*1101**

##### **Allele No: 6 Name: HLAA24**

TNNSKLYKL

RVKLSALTL

IPVMVADDL

FFTSVGKGI

##### **Allele No: 7 Name: HLAA3**

VMAPDKPSL

VMVADDLTA

##### **Allele No: 8 Name: HLAA*3101**

##### **Allele No: 9 Name: HLAA*3302**

##### **Allele No: 10 Name: HLAA68.1**

LVEFEPPHA

LVGVIMMFL

##### **Allele No: 11 Name: HLAA20 Cattle**

DKPSLDISL

IKTLKFDAL

TKDTNNSKL

NKGILVTVN

TKVIMGVVL

##### **Allele No: 12 Name: HLAA2.1**

VMAPDKPSL

CPSTGEAHL

FEPPHAATI

ALGNQEGSL

FFTSVGKGI
SLDISLETV

##### **Allele No: 13 Name: HLAB14**

DKPSLDISL

GHVSCRVKL

TKVIMGVVL

##### **Allele No: 14 Name: HLAB*2702**

VMAPDKPSL

CRIPVMVAD

##### **Allele No: 15 Name: HLAB*2705**

VMAPDKPSL

ALGNQEGSL

RVKLSALTL
QEGSLKTAL

##### **Allele No: 16 Name: HLAB*3501**

CPSTGEAHL

AATIKVLAL

RVKLSALTL

APCRIPVMV
IPVMVADDL

##### **Allele No: 17 Name: HLAB*3701**

LDISLETVA

TDIKTLKFD

FEPPHAATI

QEGSLKTAL

APCRIPVMV

FFTSVGKGI

LVGVIMMFL
IPVMVADDL

##### **Allele No: 18 Name: HLAB*3801**

CPSTGEAHL

NWNTDIKTL

PHAATIKVL

GHVSCRVKL

IPVMVADDL

##### **Allele No: 19 Name: HLAB*3901**

PHAATIKVL

TKDTNNSKL

GHVSCRVKL

##### **Allele No: 20 Name: HLAB*3902**

VMAPDKPSL

CPSTGEAHL

NWNTDIKTL

AATIKVLAL

TKDTNNSKL

GHVSCRVKL

IPVMVADDL

QTMKGAERL

TKVIMGVVL

LVGVIMMFL
DKPSLDISL

IKTLKFDAL

ALGNQEGSL

TNNSKLYKL

##### **Allele No: 21 Name: HLAB40**

LDISLETVA

FEPPHAATI

QEGSLKTAL

IPVMVADDL

QTMKGAERL
AATIKVLAL

##### **Allele No: 22 Name: HLAB*4403**

LDISLETVA

FEPPHAATI

QEGSLKTAL

##### **Allele No: 23 Name: HLAB*5101**

CPSTGEAHL

AATIKVLAL

APCRIPVMV

FFTSVGKGI
IPVMVADDL

##### **Allele No: 24 Name: HLAB*5102**

CPSTGEAHL

AATIKVLAL

APCRIPVMV

FFTSVGKGI
IPVMVADDL

##### **Allele No: 25 Name: HLAB*5103**

CPSTGEAHL

AATIKVLAL

APCRIPVMV
IPVMVADDL

##### **Allele No: 26 Name: HLAB*5201**

APCRIPVMV

##### **Allele No: 27 Name: HLAB*5301**

LDISLETVA

CPSTGEAHL

FEPPHAATI

APCRIPVMV

FFTSVGKGI

LVGVIMMFL
IPVMVADDL
VMVADDLTA

##### **Allele No: 28 Name: HLAB*5401**

CPSTGEAHL

FEPPHAATI

APCRIPVMV

FFTSVGKGI
IPVMVADDL

**Allele No: 29 Name: HLAB*51**

VMAPDKPSL

CPSTGEAHL

IKTLKFDAL

LVEFEPPHA

APCRIPVMV

FFTSVGKGI

LVGVIMMFL
LDISLETVA

FEPPHAATI

IPVMVADDL
VMVADDLTA

##### **Allele No: 30 Name: HLAB*5801**

AATIKVLAL

QTMKGAERL

##### **Allele No: 31 Name: HLAB60**

CPSTGEAHL

FEPPHAATI

QEGSLKTAL

TNNSKLYKL

IPVMVADDL

QTMKGAERL
AATIKVLAL

##### **Allele No: 32 Name: HLAB61**

LDISLETVA

FEPPHAATI

QEGSLKTAL

APCRIPVMV

##### **Allele No: 33 Name: HLAB62**

ALGNQEGSL

##### **Allele No: 34 Name: HLAB7**

VMAPDKPSL

CPSTGEAHL

AATIKVLAL

TNNSKLYKL

RVKLSALTL

APCRIPVMV

QTMKGAERL

LVGVIMMFL
ALGNQEGSL

IPVMVADDL

##### **Allele No: 35 Name: HLAB*0702**

CPSTGEAHL

RVKLSALTL

APCRIPVMV
IPVMVADDL

##### **Allele No: 36 Name: HLAB8**

CPSTGEAHL

AATIKVLAL

TNNSKLYKL

RVKLSALTL

IPVMVADDL

##### **Allele No: 37 Name: HLACw*0301**

IKTLKFDAL

GHVSCRVKL

IPVMVADDL

QTMKGAERL

TKVIMGVVL

##### **Allele No: 38 Name: HLACw*0401**

VMAPDKPSL

CPSTGEAHL

NWNTDIKTL

AATIKVLAL

TNNSKLYKL

APCRIPVMV

FFTSVGKGI
IPVMVADDL

##### **Allele No: 39 Name: HLACw*0602**

DKPSLDISL

AATIKVLAL

QEGSLKTAL

TNNSKLYKL

TKVIMGVVL

LVGVIMMFL

##### **Allele No: 40 Name: HLACw*0702**

IPVMVADDL

LVGVIMMFL

##### **Allele No: 41 Name: MHCDb**

FEPPHAATI

TKDTNNSKL

IPVMVADDL

TKVIMGVVL

##### **Allele No: 42 Name: MHCDb revised**

VMAPDKPSL

RVKLSALTL

LVGVIMMFL

##### **Allele No: 43 Name: MHCDd**

DKPSLDISL

IKTLKFDAL

FEPPHAATI

TNNSKLYKL
AATIKVLAL

##### **Allele No: 44 Name: MHCKb**

IKTLKFDAL

##### **Allele No: 45 Name: MHCKd**

##### **Allele No: 46 Name: MHCKk**

LDISLETVA

FEPPHAATI

QEGSLKTAL

FFTSVGKGI

##### **Allele No: 47 Name: MHCLd**

CPSTGEAHL

APCRIPVMV
IPVMVADDL
