# Supplementary material for: Advancing molecular modeling and reverse vaccinology in broad-spectrum yellow fever virus vaccine development
Source: Sci Rep. 2024 May 12;14:10842. doi: 10.1038/s41598-024-60680-9 (PMC11089047; doi:10.1038/s41598-024-60680-9)
Supplement: Supplementary file 1 — Supplementary Information. [file 41598_2024_60680_MOESM1_ESM.zip › Yellow_Fever_data/2_Prediction of T-cell epitopes/propred/propred ns3.docx]

##### **Allele No: 1 Name: HLAA1**

LAECARRRL

NVMAASLRK

##### **Allele No: 2 Name: HLAA2**

KIIEECEYL

VLAPTRVVL

VLVDEGRKV

##### **Allele No: 3 Name: HLAA*0201**

KIIEECEYL

YGIFQSTFL

VLAPTRVVL

IIMDEAHFL

VLVDEGRKV

RNCDLPVWL

##### **Allele No: 4 Name: HLAA*0205**

KIIEECEYL

VLAPTRVVL

IIMDEAHFL

KQKKPDFIL

VLVDEGRKV

VCWLEASML

RVSSDQSAL

##### **Allele No: 5 Name: HLAA*1101**

LVPSWASVK

NVMAASLRK

##### **Allele No: 6 Name: HLAA24**

KIIEECEYL

IIMDEAHFL

KQKKPDFIL

RNCDLPVWL

EGPEEHEIL

RVSSDQSAL

##### **Allele No: 7 Name: HLAA3**

LVPSWASVK

VLAPTRVVL

NVMAASLRK

##### **Allele No: 8 Name: HLAA*3101**

NVMAASLRK

##### **Allele No: 9 Name: HLAA*3302**

DSGETVKCR

##### **Allele No: 10 Name: HLAA68.1**

LVPSWASVK

NVMAASLRK

DSGETVKCR

##### **Allele No: 11 Name: HLAA20 Cattle**

TKPSLFKVR

DKRPTAWFL

RKVAIKGPL

DSGETVKCR

##### **Allele No: 12 Name: HLAA2.1**

YGIFQSTFL

WHVTRGAFL

VLAPTRVVL

WNTGHDWIL

CRTAFKPVL
VLVDEGRKV

##### **Allele No: 13 Name: HLAB14**

YGIFQSTFL

WHVTRGAFL

NRNGEVIGL

RRRLRTLVL

CRTAFKPVL

RKVAIKGPL
VLAPTRVVL

##### **Allele No: 14 Name: HLAB*2702**

NRNGEVIGL

RRRLRTLVL

HRARANESA

IRAANVMAA

KQKKPDFIL

CRTAFKPVL

VCWLEASML

RNCDLPVWL

PRWCDERVS

##### **Allele No: 15 Name: HLAB*2705**

KIIEECEYL

NRNGEVIGL

RRRLRTLVL

HRARANESA

IRAANVMAA

KQKKPDFIL

CRTAFKPVL

VCWLEASML

RNCDLPVWL

PRWCDERVS
VLAPTRVVL

RVSSDQSAL

##### **Allele No: 16 Name: HLAB*3501**

KIIEECEYL

IIMDEAHFL

AANVMAASL

KQKKPDFIL

RNCDLPVWL

EGPEEHEIL

RVSSDQSAL

##### **Allele No: 17 Name: HLAB*3701**

YGIFQSTFL

WHVTRGAFL

SWASVKEDL

WDGEEEVQL

IIMDEAHFL

DKRPTAWFL

PDFILATDI

RKVAIKGPL

VCWLEASML

##### **Allele No: 18 Name: HLAB*3801**

KIIEECEYL

WHVTRGAFL

SWASVKEDL

GGEIGAVAL

LAECARRRL

IIMDEAHFL

WNTGHDWIL

RNCDLPVWL

EGPEEHEIL
SWQVAKAGL

**Allele No: 19 Name: HLAB*3901**

KIIEECEYL

WHVTRGAFL

GGEIGAVAL

NRNGEVIGL

IIMDEAHFL

CRTAFKPVL

##### **Allele No: 20 Name: HLAB*3902**

KIIEECEYL

YGIFQSTFL

WHVTRGAFL

SWASVKEDL

GGEIGAVAL

NRNGEVIGL

LAECARRRL

VLAPTRVVL

IIMDEAHFL

WNTGHDWIL

DKRPTAWFL

AANVMAASL

KQKKPDFIL

CRTAFKPVL

RKVAIKGPL

VCWLEASML

RNCDLPVWL

EGPEEHEIL

RVSSDQSAL
SWQVAKAGL

##### **Allele No: 21 Name: HLAB40**

YGIFQSTFL

WDGEEEVQL

AANVMAASL

VCWLEASML
EEEVQLIAA

##### **Allele No: 22 Name: HLAB*4403**

EEEVQLIAA

##### **Allele No: 23 Name: HLAB*5101**

YGIFQSTFL

GGEIGAVAL

LAECARRRL

AANVMAASL

EGPEEHEIL

##### **Allele No: 24 Name: HLAB*5102**

YGIFQSTFL

GGEIGAVAL

LAECARRRL

AANVMAASL

VLVDEGRKV

EGPEEHEIL

##### **Allele No: 25 Name: HLAB*5103**

YGIFQSTFL

GGEIGAVAL

LAECARRRL

AANVMAASL

EGPEEHEIL

SGETVKCRA

##### **Allele No: 26 Name: HLAB*5201**

NGGEIGAVA

KQKKPDFIL

EGPEEHEIL

##### **Allele No: 27 Name: HLAB*5301**

YGIFQSTFL

WHVTRGAFL

WDGEEEVQL

VLAPTRVVL

IIMDEAHFL

WNTGHDWIL

IRAANVMAA

VCWLEASML

##### **Allele No: 28 Name: HLAB*5401**

YGIFQSTFL

WHVTRGAFL

WDGEEEVQL

WNTGHDWIL

AWFLPSIRA

PDFILATDI

VCWLEASML

##### **Allele No: 29 Name: HLAB*51**

YGIFQSTFL

WHVTRGAFL

WDGEEEVQL

LAECARRRL

VLAPTRVVL

IIMDEAHFL

WNTGHDWIL

AWFLPSIRA

VLVDEGRKV

VCWLEASML
IRAANVMAA

##### **Allele No: 30 Name: HLAB*5801**

LAECARRRL

AANVMAASL

##### **Allele No: 31 Name: HLAB60**

YGIFQSTFL

WDGEEEVQL

GGEIGAVAL

LAECARRRL

WNTGHDWIL

AANVMAASL

VCWLEASML

RNCDLPVWL

EGPEEHEIL
EEEVQLIAA

##### **Allele No: 32 Name: HLAB61**

WDGEEEVQL
EEEVQLIAA

##### **Allele No: 33 Name: HLAB62**

KQKKPDFIL

VLVDEGRKV

##### **Allele No: 34 Name: HLAB7**

KIIEECEYL

YGIFQSTFL

LAECARRRL

VLAPTRVVL

IIMDEAHFL

WNTGHDWIL

DKRPTAWFL

AANVMAASL

KQKKPDFIL

VCWLEASML

RNCDLPVWL

EGPEEHEIL

RVSSDQSAL
RRRLRTLVL

**Allele No: 35 Name: HLAB*0702**

RRRLRTLVL

HFLDPASIA

HRARANESA

KQKKPDFIL

RKVAIKGPL

RNCDLPVWL

RVSSDQSAL

##### **Allele No: 36 Name: HLAB8**

RRRLRTLVL

DKRPTAWFL

AANVMAASL

KQKKPDFIL

##### **Allele No: 37 Name: HLACw*0301**

KIIEECEYL

YGIFQSTFL

WHVTRGAFL

IIMDEAHFL

RKVAIKGPL

##### **Allele No: 38 Name: HLACw*0401**

SWASVKEDL

VLAPTRVVL

HFLDPASIA

AANVMAASL

SWQVAKAGL

##### **Allele No: 39 Name: HLACw*0602**

AANVMAASL

CRTAFKPVL

RKVAIKGPL

RNCDLPVWL

##### **Allele No: 40 Name: HLACw*0702**

##### **Allele No: 41 Name: MHCDb**

KIIEECEYL

YGIFQSTFL

IRAANVMAA

EGPEEHEIL

##### **Allele No: 42 Name: MHCDb revised**

YGIFQSTFL

AANVMAASL

RVSSDQSAL

##### **Allele No: 43 Name: MHCDd**

YGIFQSTFL

WHVTRGAFL

NGGEIGAVA

RRRLRTLVL

EGPEEHEIL
GGEIGAVAL

##### **Allele No: 44 Name: MHCKb**

IIMDEAHFL

RNCDLPVWL

EGPEEHEIL

##### **Allele No: 45 Name: MHCKd**

KIIEECEYL

YGIFQSTFL

SWASVKEDL

IIMDEAHFL

SWQVAKAGL

##### **Allele No: 46 Name: MHCKk**

WDGEEEVQL

PDFILATDI
EEEVQLIAA

##### **Allele No: 47 Name: MHCLd**
