# Supplementary material for: Advancing molecular modeling and reverse vaccinology in broad-spectrum yellow fever virus vaccine development
Source: Sci Rep. 2024 May 12;14:10842. doi: 10.1038/s41598-024-60680-9 (PMC11089047; doi:10.1038/s41598-024-60680-9)
Supplement: Supplementary file 1 — Supplementary Information. [file 41598_2024_60680_MOESM1_ESM.zip › Yellow_Fever_data/2_Prediction of T-cell epitopes/propred/propred ns5.docx]

##### **Allele No: 1 Name: HLAA1**

KVDTGVAVS

KLEGRVTDL

SVETDKGPL

##### **Allele No: 2 Name: HLAA2**

GKVDTGVAV

KLEGRVTDL

VLDTVEKWL

YMPDVLEKL

VTLEADVIL

RLCTKEEFI

YMWLGARYL

YMSPHHRKL

##### **Allele No: 3 Name: HLAA*0201**

KLEGRVTDL

VLDTVEKWL

YMPDVLEKL

VTLEADVIL

RLCTKEEFI

MMGKREKKL

YMWLGARYL

GLQHLGYVI

YMSPHHRKL

RPIDDRFGL
FIAKVRSHA

**Allele No: 4 Name: HLAA*0205**

KLEGRVTDL

VLDTVEKWL

VESFCVKVL

YMPDVLEKL

VTLEADVIL

SVETDKGPL

MMGKREKKL

YMWLGARYL

HLGYVIRDL

YMSPHHRKL

TITNLKVQL

##### **Allele No: 5 Name: HLAA*1101**

##### **Allele No: 6 Name: HLAA24**

KLEGRVTDL

YMPDVLEKL

VTLEADVIL

RPIDDRFGL
RFGLALSHL

##### **Allele No: 7 Name: HLAA3**

KLEGRVTDL

VLDTVEKWL

YMPDVLEKL

RLCTKEEFI

GLQHLGYVI

##### **Allele No: 8 Name: HLAA*3101**

##### **Allele No: 9 Name: HLAA*3302**

##### **Allele No: 10 Name: HLAA68.1**

##### **Allele No: 11 Name: HLAA20 Cattle**

GKVDTGVAV

YKNKVVKVL

##### **Allele No: 12 Name: HLAA2.1**

KLEGRVTDL

VLDTVEKWL

YMPDVLEKL

RLCTKEEFI

MMGKREKKL

YMWLGARYL

GLQHLGYVI

YMSPHHRKL

IDDRFGLAL
HLGYVIRDL

**Allele No: 13 Name: HLAB14**

DRDTARRHL

KLEGRVTDL

YMPDVLEKL

RRPTGKVTL

MMGKREKKL

ARYLEFEAL

YKNKVVKVL

RPIDDRFGL

KRDMRLLSL
RRHLAEGKV

VTLEADVIL

##### **Allele No: 14 Name: HLAB*2702**

DRDTARRHL

KLEGRVTDL

KEVSGVKGF

YMPDVLEKL

RRPTGKVTL

RLCTKEEFI

YMWLGARYL

AALEGGGFY

KRDMRLLSL
ARRHLAEGK

ARYLEFEAL
RRHLAEGKV

##### **Allele No: 15 Name: HLAB*2705**

DRDTARRHL

KLEGRVTDL

YMPDVLEKL

RRPTGKVTL

RLCTKEEFI

YMWLGARYL

RPIDDRFGL

KRDMRLLSL
ARRHLAEGK

ARYLEFEAL
RRHLAEGKV

##### **Allele No: 16 Name: HLAB*3501**

VSGVKGFTL

YMPDVLEKL

VTLEADVIL

VNRWLFRHL

AALEGGGFY

DTRITEADL

RPIDDRFGL

##### **Allele No: 17 Name: HLAB*3701**

KEVSGVKGF

VLDTVEKWL

VESFCVKVL

YMPDVLEKL

TDKGPLDRA

RLCTKEEFI

HLGYVIRDL

YKNKVVKVL

QDCDDAVLT

IDDRFGLAL

FHKRDMRLL
CDDAVLTKL
DDAVLTKLE

**Allele No: 18 Name: HLAB*3801**

FHERGYVKL

VLDTVEKWL

YMPDVLEKL

VTLEADVIL

SVETDKGPL

RPIDDRFGL

SHHFHELQL

FHKRDMRLL
KLEGRVTDL

RFGLALSHL

##### **Allele No: 19 Name: HLAB*3901**

DRDTARRHL

FHERGYVKL

YMPDVLEKL

RRPTGKVTL

ARYLEFEAL

SHHFHELQL

FHKRDMRLL
KLEGRVTDL

KRDMRLLSL

##### **Allele No: 20 Name: HLAB*3902**

DRDTARRHL

FHERGYVKL

VSGVKGFTL

VLDTVEKWL

YMPDVLEKL

RRPTGKVTL

SVETDKGPL

MMGKREKKL

YMWLGARYL

HLGYVIRDL

YMSPHHRKL

YKNKVVKVL

TITNLKVQL

RPIDDRFGL

SHHFHELQL

KRDMRLLSL
KLEGRVTDL

VTLEADVIL

ARYLEFEAL

RFGLALSHL

##### **Allele No: 21 Name: HLAB40**

KEVSGVKGF

VESFCVKVL

VTLEADVIL

TDKGPLDRA

CDDAVLTKL

RPIDDRFGL
IDDRFGLAL

##### **Allele No: 22 Name: HLAB*4403**

KEVSGVKGF

VESFCVKVL

AALEGGGFY

CDDAVLTKL

##### **Allele No: 23 Name: HLAB*5101**

RPIDDRFGL

##### **Allele No: 24 Name: HLAB*5102**

YMWLGARYL

RPIDDRFGL

##### **Allele No: 25 Name: HLAB*5103**

RPIDDRFGL

##### **Allele No: 26 Name: HLAB*5201**

VTLEADVIL

GLQHLGYVI

##### **Allele No: 27 Name: HLAB*5301**

FHERGYVKL

VSGVKGFTL

VLDTVEKWL

YMPDVLEKL

VTLEADVIL

FIAKVRSHA

YMWLGARYL

YMSPHHRKL

YKNKVVKVL

RPIDDRFGL

FHKRDMRLL
IDDRFGLAL

##### **Allele No: 28 Name: HLAB*5401**

FHERGYVKL

YMPDVLEKL

FIAKVRSHA

YMWLGARYL

YMSPHHRKL

YKNKVVKVL

RPIDDRFGL

FHKRDMRLL
ARYLEFEAL

##### **Allele No: 29 Name: HLAB*51**

FHERGYVKL

VSGVKGFTL

VLDTVEKWL

VESFCVKVL

YMPDVLEKL

VTLEADVIL

VNRWLFRHL

FIAKVRSHA

MMGKREKKL

YMWLGARYL

YMSPHHRKL

YKNKVVKVL

RPIDDRFGL

FHKRDMRLL
IDDRFGLAL

##### **Allele No: 30 Name: HLAB*5801**

KEVSGVKGF

VTLEADVIL

AALEGGGFY
VSGVKGFTL

**Allele No: 31 Name: HLAB60**

VSGVKGFTL

VESFCVKVL

VTLEADVIL

VNRWLFRHL

DTRITEADL

CDDAVLTKL

RPIDDRFGL
IDDRFGLAL

##### **Allele No: 32 Name: HLAB61**

KEVSGVKGF

VESFCVKVL

TDKGPLDRA

CDDAVLTKL

IDDRFGLAL

##### **Allele No: 33 Name: HLAB62**

GLQHLGYVI

AALEGGGFY
HLGYVIRDL

##### **Allele No: 34 Name: HLAB7**

VSGVKGFTL

YMPDVLEKL

VTLEADVIL

SVETDKGPL

VNRWLFRHL

MMGKREKKL

YMWLGARYL

HLGYVIRDL

DTRITEADL

YMSPHHRKL

TITNLKVQL

RPIDDRFGL

##### **Allele No: 35 Name: HLAB*0702**

RRHLAEGKV

KLEGRVTDL

KEVSGVKGF

RRPTGKVTL

SVETDKGPL

VNRWLFRHL

RLCTKEEFI

HLGYVIRDL

RPIDDRFGL

SHHFHELQL

FHKRDMRLL
RPTGKVTLE

RFGLALSHL

KRDMRLLSL

##### **Allele No: 36 Name: HLAB8**

KLEGRVTDL

VSGVKGFTL

VNRWLFRHL

RLCTKEEFI

MMGKREKKL

DTRITEADL

##### **Allele No: 37 Name: HLACw*0301**

FHERGYVKL

VTLEADVIL

ARYLEFEAL

YMSPHHRKL

RPIDDRFGL

##### **Allele No: 38 Name: HLACw*0401**

KLEGRVTDL

VLDTVEKWL

YMPDVLEKL

VTLEADVIL

HLGYVIRDL

DTRITEADL

YMSPHHRKL

RPIDDRFGL
RFGLALSHL

**Allele No: 39 Name: HLACw*0602**

VNRWLFRHL

YKNKVVKVL

TITNLKVQL

IDDRFGLAL

KRDMRLLSL

##### **Allele No: 40 Name: HLACw*0702**

YMPDVLEKL

HLGYVIRDL

CDDAVLTKL
AALEGGGFY

##### **Allele No: 41 Name: MHCDb**

YMPDVLEKL

VTLEADVIL

##### **Allele No: 42 Name: MHCDb revised**

YMPDVLEKL

YMWLGARYL

TITNLKVQL

##### **Allele No: 43 Name: MHCDd**

KLEGRVTDL

VSGVKGFTL

YMPDVLEKL

RRPTGKVTL

MMGKREKKL

KRDMRLLSL

##### **Allele No: 44 Name: MHCKb**

FHERGYVKL

YMPDVLEKL

VNRWLFRHL

ARYLEFEAL

##### **Allele No: 45 Name: MHCKd**

VTLEADVIL

RPIDDRFGL
RFGLALSHL

##### **Allele No: 46 Name: MHCKk**

KEVSGVKGF

VESFCVKVL

RLCTKEEFI

GLQHLGYVI

CDDAVLTKL

##### **Allele No: 47 Name: MHCLd**

KEVSGVKGF

RPIDDRFGL
VSGVKGFTL
