# Supplementary material for: Advancing molecular modeling and reverse vaccinology in broad-spectrum yellow fever virus vaccine development
Source: Sci Rep. 2024 May 12;14:10842. doi: 10.1038/s41598-024-60680-9 (PMC11089047; doi:10.1038/s41598-024-60680-9)
Supplement: Supplementary file 1 — Supplementary Information. [file 41598_2024_60680_MOESM1_ESM.zip › Yellow_Fever_data/2_Prediction of T-cell epitopes/propred/propred ns2a.docx]

##### **Allele No: 1 Name: HLAA1**

ILDLLKLTV

##### **Allele No: 2 Name: HLAA2**

MMIAMEVVL

ILVGGMVLL

AMLVGQVTI

LKLTVAVGL

TLWSPRERL

WKYLNAVSL

PVTMAEVRL
GMVLLGAML

ILDLLKLTV

VLTLGAAMV
MLVGQVTIL

##### **Allele No: 3 Name: HLAA*0201**

MMIAMEVVL

QILVGGMVL

AMLVGQVTI

TLWSPRERL

TLGAAMVEI

MMGGLWKYL

VALTLTSYL
ILVGGMVLL

MLVGQVTIL

VLTLGAAMV
GMVLLGAML

ILDLLKLTV

##### **Allele No: 4 Name: HLAA*0205**

MMIAMEVVL

QILVGGMVL

AMLVGQVTI

AAFSIRPGL

TLWSPRERL

TLGAAMVEI

MMGGLWKYL

TIPLVALTL
ILVGGMVLL

MLVGQVTIL

VLTLGAAMV

VALTLTSYL
GMVLLGAML

GQVTILDLL
VTILDLLKL

##### **Allele No: 5 Name: HLAA*1101**

TINAVASRK

##### **Allele No: 6 Name: HLAA24**

GMVLLGAML

GQVTILDLL

TIPLVALTL
VTILDLLKL

VALTLTSYL

##### **Allele No: 7 Name: HLAA3**

MMIAMEVVL

ILVGGMVLL

AMLVGQVTI

TLWSPRERL

TLGAAMVEI

MMGGLWKYL

TINAVASRK
GMVLLGAML
MLVGQVTIL

##### **Allele No: 8 Name: HLAA*3101**

##### **Allele No: 9 Name: HLAA*3302**

##### **Allele No: 10 Name: HLAA68.1**

##### **Allele No: 11 Name: HLAA20 Cattle**

LKLTVAVGL

WKYLNAVSL

QKTIPLVAL

##### **Allele No: 12 Name: HLAA2.1**

MMIAMEVVL

ILVGGMVLL

AMLVGQVTI

AAFSIRPGL

TLWSPRERL

TLGAAMVEI

MMGGLWKYL

VALTLTSYL
GMVLLGAML

GQVTILDLL

VLTLGAAMV

WKYLNAVSL
MLVGQVTIL
ILDLLKLTV

##### **Allele No: 13 Name: HLAB14**

KRQGPKQIL

MLVGQVTIL

LKLTVAVGL

AAFSIRPGL

TLWSPRERL

WKYLNAVSL

SRKASNVIL

QKTIPLVAL
QILVGGMVL

GQVTILDLL

VALTLTSYL
ILVGGMVLL

VTILDLLKL

##### **Allele No: 14 Name: HLAB*2702**

MMIAMEVVL

KRQGPKQIL

GMVLLGAML

GQVTILDLL

AAFSIRPGL

TLWSPRERL

SRKASNVIL

MQKTIPLVA
AMLVGQVTI

PRERLVLTL

##### **Allele No: 15 Name: HLAB*2705**

MMIAMEVVL

KRQGPKQIL

GMVLLGAML

GQVTILDLL

AAFSIRPGL

TLWSPRERL

SRKASNVIL

AEVRLATML
ILVGGMVLL

MLVGQVTIL

PRERLVLTL

##### **Allele No: 16 Name: HLAB*3501**

AAFSIRPGL

WSPRERLVL

GAAMVEIAL

ASNVILPLM

VALTLTSYL
MALLTPVTM

**Allele No: 17 Name: HLAB*3701**

ILVGGMVLL

GQVTILDLL

AAFSIRPGL

GAAMVEIAL

ASNVILPLM

AEVRLATML

TIPLVALTL
GMVLLGAML

LKLTVAVGL

##### **Allele No: 18 Name: HLAB*3801**

ILVGGMVLL

MLVGQVTIL

MMGGLWKYL

##### **Allele No: 19 Name: HLAB*3901**

KRQGPKQIL

MLVGQVTIL

PRERLVLTL
ILVGGMVLL

GQVTILDLL
VTILDLLKL

##### **Allele No: 20 Name: HLAB*3902**

MMIAMEVVL

KRQGPKQIL

GMVLLGAML

GQVTILDLL

AAFSIRPGL

TLWSPRERL

GAAMVEIAL

MMGGLWKYL

QKTIPLVAL
QILVGGMVL

MLVGQVTIL

LKLTVAVGL

WSPRERLVL

WKYLNAVSL

TIPLVALTL
ILVGGMVLL

VTILDLLKL

LNAVSLCIL

VALTLTSYL

##### **Allele No: 21 Name: HLAB40**

GQVTILDLL

AAFSIRPGL

GAAMVEIAL

LNAVSLCIL

AEVRLATML

VALTLTSYL
VTILDLLKL

##### **Allele No: 22 Name: HLAB*4403**

AEVRLATML

##### **Allele No: 23 Name: HLAB*5101**

AMLVGQVTI

AAFSIRPGL

GAAMVEIAL

MALLTPVTM

VALTLTSYL

##### **Allele No: 24 Name: HLAB*5102**

AMLVGQVTI

AAFSIRPGL

TLWSPRERL

GAAMVEIAL

MALLTPVTM

VALTLTSYL

##### **Allele No: 25 Name: HLAB*5103**

AMLVGQVTI

AAFSIRPGL

VLTLGAAMV

MALLTPVTM

VALTLTSYL
ILDLLKLTV

TLGAAMVEI
GAAMVEIAL

##### **Allele No: 26 Name: HLAB*5201**

MMIAMEVVL

QILVGGMVL

ILDLLKLTV

VLTLGAAMV

MQKTIPLVA

##### **Allele No: 27 Name: HLAB*5301**

MMIAMEVVL

ILVGGMVLL

MLVGQVTIL

LKLTVAVGL

WSPRERLVL

MMGGLWKYL

MALLTPVTM

MQKTIPLVA
VTILDLLKL

VLTLGAAMV

WKYLNAVSL

VALTLTSYL
@ILDLLKLTV

##### **Allele No: 28 Name: HLAB*5401**

LKLTVAVGL

AAFSIRPGL

TLWSPRERL

WKYLNAVSL

VALTLTSYL
WSPRERLVL

##### **Allele No: 29 Name: HLAB*51**

MMIAMEVVL

ILVGGMVLL

MLVGQVTIL

LKLTVAVGL

WSPRERLVL

MMGGLWKYL

LCILTINAV

MALLTPVTM

MQKTIPLVA
VTILDLLKL

VLTLGAAMV

WKYLNAVSL

VALTLTSYL
ILDLLKLTV

LNAVSLCIL

##### **Allele No: 30 Name: HLAB*5801**

VTILDLLKL

AAFSIRPGL

WSPRERLVL

GAAMVEIAL

ASNVILPLM

VALTLTSYL
MALLTPVTM

##### **Allele No: 31 Name: HLAB60**

GQVTILDLL

AAFSIRPGL

WSPRERLVL

GAAMVEIAL

LNAVSLCIL

AEVRLATML

VALTLTSYL
VTILDLLKL

##### **Allele No: 32 Name: HLAB61**

LCILTINAV

AEVRLATML

##### **Allele No: 33 Name: HLAB62**

ILVGGMVLL

MLVGQVTIL

VLTLGAAMV

MQKTIPLVA
GQVTILDLL

TLGAAMVEI

##### **Allele No: 34 Name: HLAB7**

MMIAMEVVL

QILVGGMVL

MLVGQVTIL

AAFSIRPGL

TLWSPRERL

GAAMVEIAL

MMGGLWKYL

TIPLVALTL
ILVGGMVLL

GQVTILDLL

WSPRERLVL

LNAVSLCIL

VALTLTSYL
GMVLLGAML

VTILDLLKL

##### **Allele No: 35 Name: HLAB*0702**

KRQGPKQIL

##### **Allele No: 36 Name: HLAB8**

AAFSIRPGL

GAAMVEIAL

VALTLTSYL

##### **Allele No: 37 Name: HLACw*0301**

MMIAMEVVL

QILVGGMVL

MLVGQVTIL

LKLTVAVGL

WKYLNAVSL

MALLTPVTM

VALTLTSYL
ILVGGMVLL

GQVTILDLL

AEVRLATML
GMVLLGAML

VTILDLLKL

##### **Allele No: 38 Name: HLACw*0401**

MLVGQVTIL

LNAVSLCIL
GQVTILDLL
VTILDLLKL

##### **Allele No: 39 Name: HLACw*0602**

MMIAMEVVL

GMVLLGAML

GQVTILDLL

AAFSIRPGL

MMGGLWKYL

AEVRLATML

VALTLTSYL

##### **Allele No: 40 Name: HLACw*0702**

##### **Allele No: 41 Name: MHCDb**

MMIAMEVVL

QILVGGMVL

AMLVGQVTI

WSPRERLVL

WKYLNAVSL

MALLTPVTM

TIPLVALTL
ILVGGMVLL

GQVTILDLL

AEVRLATML

VALTLTSYL
GMVLLGAML

VTILDLLKL

##### **Allele No: 42 Name: MHCDb revised**

MMIAMEVVL

ILVGGMVLL

AMLVGQVTI

AAFSIRPGL

TLWSPRERL

GAAMVEIAL

MMGGLWKYL

ASNVILPLM

VALTLTSYL
GMVLLGAML

GQVTILDLL

WSPRERLVL

WKYLNAVSL

MALLTPVTM
MLVGQVTIL

##### **Allele No: 43 Name: MHCDd**

WSPRERLVL

TIPLVALTL

##### **Allele No: 44 Name: MHCKb**

WKYLNAVSL

TIPLVALTL
VALTLTSYL

##### **Allele No: 45 Name: MHCKd**

MMIAMEVVL

QILVGGMVL

AMLVGQVTI

VALTLTSYL
ILVGGMVLL

MLVGQVTIL
GMVLLGAML

GQVTILDLL
VTILDLLKL

##### **Allele No: 46 Name: MHCKk**

AMLVGQVTI

TLGAAMVEI

AEVRLATML

##### **Allele No: 47 Name: MHCLd**

WSPRERLVL

ASNVILPLM
