# Supplementary material for: Advancing molecular modeling and reverse vaccinology in broad-spectrum yellow fever virus vaccine development
Source: Sci Rep. 2024 May 12;14:10842. doi: 10.1038/s41598-024-60680-9 (PMC11089047; doi:10.1038/s41598-024-60680-9)
Supplement: Supplementary file 1 — Supplementary Information. [file 41598_2024_60680_MOESM1_ESM.zip › Yellow_Fever_data/2_Prediction of T-cell epitopes/propred/propred ns4a.docx]

##### **Allele No: 1 Name: HLAA1**

AVDTISVFL

##### **Allele No: 2 Name: HLAA2**

VLSELPDFL

TTVMLFVLA

YIMLIFFVL
MLFVLAGLL

##### **Allele No: 3 Name: HLAA*0201**

VLSELPDFL

AVDTISVFL

MLFVLAGLL

YIMLIFFVL

##### **Allele No: 4 Name: HLAA*0205**

GAAEVLVVL

AVDTISVFL

MTTVMLFVL

YIMLIFFVL
EVLVVLSEL

MLFVLAGLL
VLSELPDFL

##### **Allele No: 5 Name: HLAA*1101**

##### **Allele No: 6 Name: HLAA24**

GAAEVLVVL

YIMLIFFVL
EVLVVLSEL

##### **Allele No: 7 Name: HLAA3**

VLSELPDFL

MLFVLAGLL

YIMLIFFVL

##### **Allele No: 8 Name: HLAA*3101**

YIMLIFFVL

##### **Allele No: 9 Name: HLAA*3302**

EVLVVLSEL

##### **Allele No: 10 Name: HLAA68.1**

EVLVVLSEL

##### **Allele No: 11 Name: HLAA20 Cattle**

##### **Allele No: 12 Name: HLAA2.1**

VLSELPDFL

MLFVLAGLL

YIMLIFFVL

##### **Allele No: 13 Name: HLAB14**

EVLVVLSEL

MLFVLAGLL

##### **Allele No: 14 Name: HLAB*2702**

MLFVLAGLL

##### **Allele No: 15 Name: HLAB*2705**

VLSELPDFL

MLFVLAGLL

##### **Allele No: 16 Name: HLAB*3501**

GAAEVLVVL
VLSELPDFL

##### **Allele No: 17 Name: HLAB*3701**

GAAEVLVVL

AVDTISVFL

PEAMTTVML

YIMLIFFVL
EVLVVLSEL

MLFVLAGLL
VLSELPDFL

##### **Allele No: 18 Name: HLAB*3801**

GAAEVLVVL

AVDTISVFL
VLSELPDFL

##### **Allele No: 19 Name: HLAB*3901**

GAAEVLVVL

AVDTISVFL
EVLVVLSEL

##### **Allele No: 20 Name: HLAB*3902**

GAAEVLVVL

AVDTISVFL

MTTVMLFVL

YIMLIFFVL
EVLVVLSEL

MLFVLAGLL
VLSELPDFL

##### **Allele No: 21 Name: HLAB40**

GAAEVLVVL

PEAMTTVML
TTVMLFVLA

##### **Allele No: 22 Name: HLAB*4403**

##### **Allele No: 23 Name: HLAB*5101**

GAAEVLVVL

##### **Allele No: 24 Name: HLAB*5102**

GAAEVLVVL

##### **Allele No: 25 Name: HLAB*5103**

GAAEVLVVL

##### **Allele No: 26 Name: HLAB*5201**

##### **Allele No: 27 Name: HLAB*5301**

VLSELPDFL

MTTVMLFVL

YIMLIFFVL
MLFVLAGLL

**Allele No: 28 Name: HLAB*5401**

MLFVLAGLL

YIMLIFFVL

##### **Allele No: 29 Name: HLAB*51**

VLSELPDFL

MTTVMLFVL

YIMLIFFVL
MLFVLAGLL

##### **Allele No: 30 Name: HLAB*5801**

GAAEVLVVL

MTTVMLFVL
TTVMLFVLA

##### **Allele No: 31 Name: HLAB60**

GAAEVLVVL

PEAMTTVML
MTTVMLFVL

##### **Allele No: 32 Name: HLAB61**

PEAMTTVML
TTVMLFVLA

##### **Allele No: 33 Name: HLAB62**

VLSELPDFL

MLFVLAGLL

##### **Allele No: 34 Name: HLAB7**

GAAEVLVVL

AVDTISVFL

MTTVMLFVL

YIMLIFFVL
EVLVVLSEL

MLFVLAGLL
VLSELPDFL

##### **Allele No: 35 Name: HLAB*0702**

##### **Allele No: 36 Name: HLAB8**

GAAEVLVVL
VLSELPDFL

EVLVVLSEL

YIMLIFFVL

##### **Allele No: 38 Name: HLACw*0401**

GAAEVLVVL

AVDTISVFL

MTTVMLFVL
EVLVVLSEL

##### **Allele No: 39 Name: HLACw*0602**

VLSELPDFL

AVDTISVFL

MTTVMLFVL

YIMLIFFVL
MLFVLAGLL

##### **Allele No: 40 Name: HLACw*0702**

GAAEVLVVL

##### **Allele No: 41 Name: MHCDb**

EVLVVLSEL

##### **Allele No: 42 Name: MHCDb revised**

GAAEVLVVL

##### **Allele No: 43 Name: MHCDd**

##### **Allele No: 44 Name: MHCKb**

MLFVLAGLL

YIMLIFFVL

##### **Allele No: 45 Name: MHCKd**

EVLVVLSEL

YIMLIFFVL

##### **Allele No: 46 Name: MHCKk**

##### **Allele No: 47 Name: MHCLd**
