# Supplementary material for: Advancing molecular modeling and reverse vaccinology in broad-spectrum yellow fever virus vaccine development
Source: Sci Rep. 2024 May 12;14:10842. doi: 10.1038/s41598-024-60680-9 (PMC11089047; doi:10.1038/s41598-024-60680-9)
Supplement: Supplementary file 1 — Supplementary Information. [file 41598_2024_60680_MOESM1_ESM.zip › Yellow_Fever_data/2_Prediction of T-cell epitopes/propred/propred ns4b.docx]

##### **Allele No: 1 Name: HLAA1**

LAEGIVLAS

##### **Allele No: 2 Name: HLAA2**

ALYEKKLAL

AMCRTPFSL
KKLALYLLL

##### **Allele No: 3 Name: HLAA*0201**

ALYEKKLAL

AMCRTPFSL
LALYLLLAL

##### **Allele No: 4 Name: HLAA*0205**

ALYEKKLAL

AMCRTPFSL

GIVLASAAL
LALYLLLAL

FSLAEGIVL
LASAALGPL

##### **Allele No: 5 Name: HLAA*1101**

##### **Allele No: 6 Name: HLAA24**

LALYLLLAL
LYLLLALSL

##### **Allele No: 7 Name: HLAA3**

ELGMLEKTK

ALYEKKLAL

AMCRTPFSL

##### **Allele No: 8 Name: HLAA*3101**

##### **Allele No: 9 Name: HLAA*3302**

##### **Allele No: 10 Name: HLAA68.1**

ELGMLEKTK

##### **Allele No: 11 Name: HLAA20 Cattle**

ELGMLEKTK

EKKLALYLL
KKLALYLLL

##### **Allele No: 12 Name: HLAA2.1**

MPALYEKKL

AMCRTPFSL
ALYEKKLAL

FSLAEGIVL

##### **Allele No: 13 Name: HLAB14**

ALYEKKLAL
EKKLALYLL
KKLALYLLL
LALYLLLAL

##### **Allele No: 14 Name: HLAB*2702**

ALYEKKLAL

AMCRTPFSL
CRTPFSLAE
TPFSLAEGI

##### **Allele No: 15 Name: HLAB*2705**

ALYEKKLAL

AMCRTPFSL
YEKKLALYL

##### **Allele No: 16 Name: HLAB*3501**

GIKAQQSKL

MPALYEKKL

TPFSLAEGI

LASAALGPL
ALYEKKLAL

FSLAEGIVL
LALYLLLAL

##### **Allele No: 17 Name: HLAB*3701**

EDLFGKKNL

YEKKLALYL

AEGIVLASA
EKKLALYLL
KKLALYLLL

##### **Allele No: 18 Name: HLAB*3801**

MPALYEKKL

LYLLLALSL
ALYEKKLAL

**Allele No: 19 Name: HLAB*3901**

LALYLLLAL

##### **Allele No: 20 Name: HLAB*3902**

MPALYEKKL

LYLLLALSL

AMCRTPFSL

GIVLASAAL
ALYEKKLAL

FSLAEGIVL
EKKLALYLL

LASAALGPL
KKLALYLLL
LALYLLLAL

**Allele No: 21 Name: HLAB40**

EDLFGKKNL

MPALYEKKL

FSLAEGIVL
YEKKLALYL

AEGIVLASA
LALYLLLAL

##### **Allele No: 22 Name: HLAB*4403**

EDLFGKKNL

YEKKLALYL

AEGIVLASA

##### **Allele No: 23 Name: HLAB*5101**

MPALYEKKL

TPFSLAEGI

LASAALGPL
LALYLLLAL

##### **Allele No: 24 Name: HLAB*5102**

MPALYEKKL

TPFSLAEGI

LASAALGPL
LALYLLLAL

##### **Allele No: 25 Name: HLAB*5103**

MPALYEKKL

TPFSLAEGI

LASAALGPL
LALYLLLAL

LAEGIVLAS

##### **Allele No: 26 Name: HLAB*5201**

TPFSLAEGI

##### **Allele No: 27 Name: HLAB*5301**

MPALYEKKL

LYLLLALSL

TPFSLAEGI
YEKKLALYL

FSLAEGIVL
LALYLLLAL

##### **Allele No: 28 Name: HLAB*5401**

MPALYEKKL

LYLLLALSL

TPFSLAEGI
ALYEKKLAL]

FSLAEGIVL
YEKKLALYL
LALYLLLAL

##### **Allele No: 29 Name: HLAB*51**

MPALYEKKL

LYLLLALSL

TPFSLAEGI

LASAALGPL
YEKKLALYL

FSLAEGIVL
LALYLLLAL

##### **Allele No: 30 Name: HLAB*5801**

PSSAAPWSW

LALYLLLAL

FSLAEGIVL
LASAALGPL

##### **Allele No: 31 Name: HLAB60**

EDLFGKKNL

MPALYEKKL

FSLAEGIVL
YEKKLALYL

AEGIVLASA
LALYLLLAL

LASAALGPL

##### **Allele No: 32 Name: HLAB61**

EDLFGKKNL

YEKKLALYL

AEGIVLASA

##### **Allele No: 33 Name: HLAB62**

##### **Allele No: 34 Name: HLAB7**

GIKAQQSKL

MPALYEKKL

AMCRTPFSL

GIVLASAAL
ALYEKKLAL

TPFSLAEGI

LASAALGPL
LALYLLLAL

FSLAEGIVL

##### **Allele No: 35 Name: HLAB*0702**

MPALYEKKL

TPFSLAEGI
YEKKLALYL
KKLALYLLL

##### **Allele No: 36 Name: HLAB8**

GIKAQQSKL

MPALYEKKL

LASAALGPL
ALYEKKLAL
EKKLALYLL
LALYLLLAL

##### **Allele No: 37 Name: HLACw*0301**

EDLFGKKNL

ALYEKKLAL

FSLAEGIVL
KKLALYLLL

GIVLASAAL
LALYLLLAL
LYLLLALSL

##### **Allele No: 38 Name: HLACw*0401**

MPALYEKKL

LYLLLALSL

AMCRTPFSL

GIVLASAAL
LALYLLLAL

TPFSLAEGI

LASAALGPL

##### **Allele No: 39 Name: HLACw*0602**

YEKKLALYL
KKLALYLLL
LALYLLLAL
LYLLLALSL

##### **Allele No: 40 Name: HLACw*0702**

MPALYEKKL

LYLLLALSL

**Allele No: 41 Name: MHCDb**

LALYLLLAL

FSLAEGIVL

##### **Allele No: 42 Name: MHCDb revised**

LALYLLLAL

AMCRTPFSL

LASAALGPL
FSLAEGIVL

##### **Allele No: 43 Name: MHCDd**

##### **Allele No: 44 Name: MHCKb**

KKLALYLLL

##### **Allele No: 45 Name: MHCKd**

LALYLLLAL

FSLAEGIVL
LYLLLALSL

GIVLASAAL

##### **Allele No: 46 Name: MHCKk**

EDLFGKKNL

YEKKLALYL

TPFSLAEGI
AEGIVLASA

##### **Allele No: 47 Name: MHCLd**

EDLFGKKNL

MPALYEKKL

TPFSLAEGI
FSLAEGIVL
