# Supplementary material for: Advancing molecular modeling and reverse vaccinology in broad-spectrum yellow fever virus vaccine development
Source: Sci Rep. 2024 May 12;14:10842. doi: 10.1038/s41598-024-60680-9 (PMC11089047; doi:10.1038/s41598-024-60680-9)
Supplement: Supplementary file 1 — Supplementary Information. [file 41598_2024_60680_MOESM1_ESM.zip › Yellow_Fever_data/2_Prediction of T-cell epitopes/MHC CLASS II/NETMHCII C.docx]

**Proteína C**

**Allele: DRB1_0101. Number of high binders 6.**

47 FIFFFLFNVLTGKKI

48 IFFFLFNVLTGKKIT

49 FFFLFNVLTGKKITA

50 FFLFNVLTGKKITAH

51 FLFNVLTGKKITAHL

52 LFNVLTGKKITAHLK

**Allele: DRB1_0301. Number of high binders 9**

7 QGKTLGVNMVRRGVR

8 GKTLGVNMVRRGVRS

9 KTLGVNMVRRGVRSL

10 TLGVNMVRRGVRSLS

11 LGVNMVRRGVRSLSS

12 GVNMVRRGVRSLSSK

13 VNMVRRGVRSLSSKI

75 RQGLAVLKKVKRVVA

76 QGLAVLKKVKRVVAS

**Allele: DRB1_0401. Number of high binders 0.**

**Allele: DRB1_0405. Number of high binders 3.**

63 AHLKKLWRMLDPRQG

64 HLKKLWRMLDPRQGL

65 LKKLWRMLDPRQGLA

**Allele: DRB1_0701. Number of high binders 4**

47 FIFFFLFNVLTGKKI

81 LKKVKRVVASLMRGL

78 LAVLKKVKRVVASLM

79 AVLKKVKRVVASLMR

**Allele: DRB1_0802. Number of high binders 7**.

63 AHLKKLWRMLDPRQG

64 HLKKLWRMLDPRQGL

76 QGLAVLKKVKRVVAS

77 GLAVLKKVKRVVASL

78 LAVLKKVKRVVASLM

79 AVLKKVKRVVASLMR

80 VLKKVKRVVASLMRG

**Allele: DRB1_0901. Number of high binders 2**

81 LKKVKRVVASLMRGL

82 KKVKRVVASLMRGLS

**Allele: DRB1_1101. Number of high binders 38.**

8 GKTLGVNMVRRGVRS

9 KTLGVNMVRRGVRSL

10 TLGVNMVRRGVRSLS

11 LGVNMVRRGVRSLSS

12 GVNMVRRGVRSLSSK

13 VNMVRRGVRSLSSKI

14 NMVRRGVRSLSSKIK

15 MVRRGVRSLSSKIKQ

16 VRRGVRSLSSKIKQK

55 VLTGKKITAHLKKLW

56 LTGKKITAHLKKLWR

57 TGKKITAHLKKLWRM

58 GKKITAHLKKLWRML

59 KKITAHLKKLWRMLD

60 KITAHLKKLWRMLDP

61 ITAHLKKLWRMLDPR

62 TAHLKKLWRMLDPRQ

63 AHLKKLWRMLDPRQG

64 HLKKLWRMLDPRQGL

65 LKKLWRMLDPRQGLA

66 KKLWRMLDPRQGLAV

67 KLWRMLDPRQGLAVL

72 LDPRQGLAVLKKVKR

73 DPRQGLAVLKKVKRV

74 PRQGLAVLKKVKRVV

75 RQGLAVLKKVKRVVA

76 QGLAVLKKVKRVVAS

77 GLAVLKKVKRVVASL

78 LAVLKKVKRVVASLM

79 AVLKKVKRVVASLMR

80 VLKKVKRVVASLMRG

81 LKKVKRVVASLMRGL

82 KKVKRVVASLMRGLS

83 KVKRVVASLMRGLSS

84 VKRVVASLMRGLSSR

85 KRVVASLMRGLSSRK

86 RVVASLMRGLSSRKR

87 VVASLMRGLSSRKRR

**Allele: DRB1_1201. Number of high binders 10**

58 GKKITAHLKKLWRML

59 KKITAHLKKLWRMLD

60 KITAHLKKLWRMLDP

61 ITAHLKKLWRMLDPR

74 PRQGLAVLKKVKRVV

75 RQGLAVLKKVKRVVA

76 QGLAVLKKVKRVVAS

77 GLAVLKKVKRVVASL

78 LAVLKKVKRVVASLM

79 AVLKKVKRVVASLMR

**Allele: DRB1_1302. Number of high binders 0**

**Allele: DRB1_1501. Number of high binders 0.**

**Allele: DRB3_0101. Number of high binders 0**

**Allele: DRB3_0202. Number of high binders 4.**

6 AQGKTLGVNMVRRGV

7 QGKTLGVNMVRRGVR

8 GKTLGVNMVRRGVRS

9 KTLGVNMVRRGVRSL

**Allele: DRB4_0101. Number of high binders 0.**

**Allele: DRB5_0101. Number of high binders 48**

7 QGKTLGVNMVRRGVR

8 GKTLGVNMVRRGVRS

9 KTLGVNMVRRGVRSL

10 TLGVNMVRRGVRSLS

11 LGVNMVRRGVRSLSS

12 GVNMVRRGVRSLSSK

13 VNMVRRGVRSLSSKI

14 NMVRRGVRSLSSKIK

15 MVRRGVRSLSSKIKQ

16 VRRGVRSLSSKIKQK

17 RRGVRSLSSKIKQKT

18 RGVRSLSSKIKQKTK

19 GVRSLSSKIKQKTKQ

20 VRSLSSKIKQKTKQI

47 FIFFFLFNVLTGKKI

48 IFFFLFNVLTGKKIT

49 FFFLFNVLTGKKITA

50 FFLFNVLTGKKITAH

51 FLFNVLTGKKITAHL

52 LFNVLTGKKITAHLK

53 FNVLTGKKITAHLKK

54 NVLTGKKITAHLKKL

55 VLTGKKITAHLKKLW

56 LTGKKITAHLKKLWR

57 TGKKITAHLKKLWRM

58 GKKITAHLKKLWRML

64 HLKKLWRMLDPRQGL

65 LKKLWRMLDPRQGLA

66 KKLWRMLDPRQGLAV

67 KLWRMLDPRQGLAVL

69 WRMLDPRQGLAVLKK

71 MLDPRQGLAVLKKVK

72 LDPRQGLAVLKKVKR

73 DPRQGLAVLKKVKRV

74 PRQGLAVLKKVKRVV

75 RQGLAVLKKVKRVVA

76 QGLAVLKKVKRVVAS

77 GLAVLKKVKRVVASL

78 LAVLKKVKRVVASLM

79 AVLKKVKRVVASLMR

80 VLKKVKRVVASLMRG

81 LKKVKRVVASLMRGL

82 KKVKRVVASLMRGLS

83 KVKRVVASLMRGLSS

84 VKRVVASLMRGLSSR

85 KRVVASLMRGLSSRK

86 RVVASLMRGLSSRKR

87 VVASLMRGLSSRKRR

**Allele: HLA-DQA10501-DQB10201. Number of high binders 0.**

**Allele: HLA-DQA10501-DQB10301. Number of high binders 0.**

**Allele: HLA-DQA10301-DQB10302. Number of high binders 0.**

**Allele: HLA-DQA10401-DQB10402. Number of high binders 0.**

**Allele: HLA-DQA10101-DQB10501. Number of high binders 0.**

**Allele: HLA-DQA10102-DQB10602. Number of high binders 3.**

83 KVKRVVASLMRGLSS

84 VKRVVASLMRGLSSR

85 KRVVASLMRGLSSRK

**Allele: HLA-DPA10201-DPB10101. Number of high binders 0.**

**Allele: HLA-DPA10103-DPB10201. Number of high binders 8.**

39 GPSRGVQGFIFFFLF

40 PSRGVQGFIFFFLFN

41 SRGVQGFIFFFLFNV

42 RGVQGFIFFFLFNVL

43 GVQGFIFFFLFNVLT

44 VQGFIFFFLFNVLTG

45 QGFIFFFLFNVLTGK

46 GFIFFFLFNVLTGKK

**Allele: HLA-DPA10103-DPB10401. Number of high binders 0.**

**Allele: HLA-DPA10301-DPB10402. Number of high binders 0.**

**Allele: HLA-DPA10201-DPB10501. Number of high binders 13.**

54 NVLTGKKITAHLKKL

55 VLTGKKITAHLKKLW

56 LTGKKITAHLKKLWR

57 TGKKITAHLKKLWRM

58 GKKITAHLKKLWRML

74 PRQGLAVLKKVKRVV

75 RQGLAVLKKVKRVVA

79 AVLKKVKRVVASLMR

80 VLKKVKRVVASLMRG

81 LKKVKRVVASLMRGL

82 KKVKRVVASLMRGLS

83 KVKRVVASLMRGLSS

84 VKRVVASLMRGLSSR

**Allele: HLA-DPA10201-DPB11401. Number of high binders 11.**

78 LAVLKKVKRVVASLM

56 LTGKKITAHLKKLWR

57 TGKKITAHLKKLWRM

58 GKKITAHLKKLWRML

79 AVLKKVKRVVASLMR

80 VLKKVKRVVASLMRG

81 LKKVKRVVASLMRGL

82 KKVKRVVASLMRGLS

83 KVKRVVASLMRGLSS

84 VKRVVASLMRGLSSR

85 KRVVASLMRGLSSRK
