# Supplementary material for: Advancing molecular modeling and reverse vaccinology in broad-spectrum yellow fever virus vaccine development
Source: Sci Rep. 2024 May 12;14:10842. doi: 10.1038/s41598-024-60680-9 (PMC11089047; doi:10.1038/s41598-024-60680-9)
Supplement: Supplementary file 1 — Supplementary Information. [file 41598_2024_60680_MOESM1_ESM.zip › Yellow_Fever_data/2_Prediction of T-cell epitopes/MHC CLASS II/NETMHCII E.docx]

**Proteína E**

**Allele: DRB1_0101. Number of high binders 0.**

**Allele: DRB1_0301. Number of high binders 20.**

45 LETVAIDGPAEARKV

123 KSMSLFEVDQTKIQY

124 SMSLFEVDQTKIQYV

125 MSLFEVDQTKIQYVI

126 SLFEVDQTKIQYVIR

127 LFEVDQTKIQYVIRA

129 EVDQTKIQYVIRAQL

130 VDQTKIQYVIRAQLH

131 DQTKIQYVIRAQLHV

132 QTKIQYVIRAQLHVG

133 TKIQYVIRAQLHVGA

134 KIQYVIRAQLHVGAK

148 KQENWNTDIKTLKFD

149 QENWNTDIKTLKFDA

150 ENWNTDIKTLKFDAL

374 SYIIVGTGDSRLTYQ

375 YIIVGTGDSRLTYQW

376 IIVGTGDSRLTYQWH

466 INTRNMTMSMSMILV

468 TRNMTMSMSMILVGV

**Allele: DRB1_0401. Number of high binders 5.**

344 VNKGILVTVNPIAST

345 NKGILVTVNPIASTN

346 KGILVTVNPIASTND

385 LTYQWHKEGSSIGKL

386 TYQWHKEGSSIGKLF

**Allele: DRB1_0405. Number of high binders 0.**

**Allele: DRB1_0701. Number of high binders 17.**

113 IVACAKFTCAKSMSL

114 VACAKFTCAKSMSLF

115 ACAKFTCAKSMSLFE

116 CAKFTCAKSMSLFEV

117 AKFTCAKSMSLFEVD

420 FGSAGGFFTSVGKGI

421 GSAGGFFTSVGKGIH

422 SAGGFFTSVGKGIHT

423 AGGFFTSVGKGIHTV

424 GGFFTSVGKGIHTVF

425 GFFTSVGKGIHTVFG

463 WVGINTRNMTMSMSM

464 VGINTRNMTMSMSM

465 GINTRNMTMSMSMIL

466 INTRNMTMSMSMILV

467 NTRNMTMSMSMILVG

468 TRNMTMSMSMILVGV

**Allele: DRB1_0802. Number of high binders 10.**

342 AAVNKGILVTVNPIA

343 AVNKGILVTVNPIAS

344 VNKGILVTVNPIAST

345 NKGILVTVNPIASTN

346 KGILVTVNPIASTND

347 GILVTVNPIASTNDD

348 ILVTVNPIASTNDDE

360 DDEVLIEVNPPFGDS

361 DEVLIEVNPPFGDSY

362 EVLIEVNPPFGDSYI

**Allele: DRB1_0901. Number of high binders 9.**

420 FGSAGGFFTSVGKGI

421 GSAGGFFTSVGKGIH

422 SAGGFFTSVGKGIHT

423 AGGFFTSVGKGIHTV

424 GGFFTSVGKGIHTVF

464 VGINTRNMTMSMSMI

465 GINTRNMTMSMSMIL

466 INTRNMTMSMSMILV

467 NTRNMTMSMSMILVG

**Allele: DRB1_1101. Number of high binders 5.**

421 GSAGGFFTSVGKGIH

422 SAGGFFTSVGKGIHT

423 AGGFFTSVGKGIHTV

424 GGFFTSVGKGIHTVF

425 GFFTSVGKGIHTVFG

**Allele: DRB1_1201. Number of high binders 3.**

283 CRVKLSALTLKGTSY

284 RVKLSALTLKGTSYK

285 VKLSALTLKGTSYKM

**Allele: DRB1_1302. Number of high binders 4**

336 VADDLTAAVNKGILV

337 ADDLTAAVNKGILVT

338 DDLTAAVNKGILVTV

339 DLTAAVNKGILVTVN

**Allele: DRB1_1501. Number of high binders 0.**

**Allele: DRB3_0101. Number of high binders 25.**

115 ACAKFTCAKSMSLFE

116 CAKFTCAKSMSLFEV

117 AKFTCAKSMSLFEVD

122 AKSMSLFEVDQTKIQ

123 KSMSLFEVDQTKIQY

124 SMSLFEVDQTKIQYV

125 MSLFEVDQTKIQYVI

126 SLFEVDQTKIQYVIR

127 LFEVDQTKIQYVIRA

198 MEKESWIVDKQWAQD

199 EKESWIVDKQWAQDL

200 KESWIVDKQWAQDLT

203 WIVDKQWAQDLTLPW

204 IVDKQWAQDLTLPWQ

205 VDKQWAQDLTLPWQS

206 DKQWAQDLTLPWQSG

207 KQWAQDLTLPWQSGS

208 QWAQDLTLPWQSGSG

330 CRIPVMVADDLTAAV

331 RIPVMVADDLTAAVN

332 IPVMVADDLTAAVNK

406 GAERLAVMGDAAWDF

408 ERLAVMGDAAWDFGS

409 RLAVMGDAAWDFGSA

**Allele: DRB4_0101.Number of high binders 28**

121 CAKSMSLFEVDQTKI

122 AKSMSLFEVDQTKIQ

123 KSMSLFEVDQTKIQY

129 EVDQTKIQYVIRAQL

130 VDQTKIQYVIRAQLH

131 DQTKIQYVIRAQLHV

132 QTKIQYVIRAQLHVG

133 TKIQYVIRAQLHVGA

134 KIQYVIRAQLHVGAK

135 IQYVIRAQLHVGAKQ

136 QYVIRAQLHVGAKQE

137 YVIRAQLHVGAKQEN

151 NWNTDIKTLKFDALS

152 WNTDIKTLKFDALSG

153 NTDIKTLKFDALSGS

154 TDIKTLKFDALSGSQ

235 EPPHAATIKVLALGN

236 PPHAATIKVLALGNQ

237 PHAATIKVLALGNQE

379 GTGDSRLTYQWHKEG

380 TGDSRLTYQWHKEGS

381 GDSRLTYQWHKEGSS

382 DSRLTYQWHKEGSSI

465 GINTRNMTMSMSMIL

466 INTRNMTMSMSMILV

467 NTRNMTMSMSMILVG

468 TRNMTMSMSMILVGV

469 RNMTMSMSMILVGVI

**Allele: DRB5_0101. Number of high binders 4.**

57 RKVCYSAVLTNVKIN

58 KVCYSAVLTNVKIND

135 IQYVIRAQLHVGAKQ

252 GSLKTALTGAMRVTK

**Allele: DRB3_0202. Number of high binders 9.**

301 TDKMSFVKNPTDTGH

302 DKMSFVKNPTDTGHGGT

303 KMSFVKNPTDTGHGT

304 MSFVKNPTDTGHGTA

338 DDLTAAVNKGILVTV

339 DLTAAVNKGILVTVN

340 LTAAVNKGILVTVNP

466 INTRNMTMSMSMILV

467 NTRNMTMSMSMILVG

**Allele: HLA-DQA10501-DQB10201. Number of high binders 3.**

406 GAERLAVMGDAAWDF

407 AERLAVMGDAAWDFG

408 ERLAVMGDAAWDFGS

**Allele: HLA-DQA10501-DQB10301. Number of high binders 15.**

10 DFIEGVHGGTWVSAT

11 FIEGVHGGTWVSATL

12 IEGVHGGTWVSATLE

13 EGVHGGTWVSATLEQ

14 GVHGGTWVSATLEQD

103 NGCGLFGKGSIVACA

104 GCGLFGKGSIVACAK

105 CGLFGKGSIVACAKF

106 GLFGKGSIVACAKFT

107 LFGKGSIVACAKFTC

108 FGKGSIVACAKFTCA

416 AAWDFGSAGGFFTSV

417 AWDFGSAGGFFTSVG

418 WDFGSAGGFFTSVGK

419 DFGSAGGFFTSVGKG

**Allele: HLA-DQA10301-DQB10302. Number of high binders 14.**

158 TLKFDALSGSQEAEF

159 LKFDALSGSQEAEFT

185 QTAVDFSNSYIAEME

186 TAVDFSNSYIAEMEK

187 AVDFSNSYIAEMEKE

188 VDFSNSYIAEMEKES

189 DFSNSYIAEMEKESW

190 FSNSYIAEMEKESWI

353 NPIASTNDDEVLIEV

354 PIASTNDDEVLIEVN

355 IASTNDDEVLIEVNP

356 ASTNDDEVLIEVNPP

357 STNDDEVLIEVNPPF

358 TNDDEVLIEVNPPFG

**Allele: HLA-DQA10401-DQB10402. Number of high binders 14.**

14 GVHGGTWVSATLEQD

15 VHGGTWVSATLEQDK

16 HGGTWVSATLEQDKC

17 GGTWVSATLEQDKCV

18 GTWVSATLEQDKCVT

157 KTLKFDALSGSQEAE

158 TLKFDALSGSQEAEF

159 LKFDALSGSQEAEFT

184 VQTAVDFSNSYIAEM

185 QTAVDFSNSYIAEME

186 TAVDFSNSYIAEMEK

187 AVDFSNSYIAEMEKE

188 VDFSNSYIAEMEKES

189 DFSNSYIAEMEKESW

**Allele: HLA-DQA10101-DQB10501. Number of high binders 0.**

**Allele: HLA-DQA10102-DQB10602. Number of high binders 7.**

105 CGLFGKGSIVACAKF

106 GLFGKGSIVACAKFT

309 NPTDTGHGTAVMQVK

310 PTDTGHGTAVMQVKV

311 TDTGHGTAVMQVKVP

312 DTGHGTAVMQVKVPK

313 TGHGTAVMQVKVPKG

**Allele: HLA-DPA10201-DPB10101. Number of high binders 0.**

**Allele: HLA-DPA10103-DPB10201. Number of high binders 0.**

**Allele: HLA-DPA10103-DPB10401. Number of high binders 4.**

431 GKGIHTVFGSAFQGL

432 KGIHTVFGSAFQGLF

433 GIHTVFGSAFQGLFG

434 IHTVFGSAFQGLFGG

**Allele: HLA-DPA10103-DPB10402. Number of high binders 8.**

124 SMSLFEVDQTKIQYV

125 MSLFEVDQTKIQYVI

217 WQSGSGGVWREMHHL

218 QSGSGGVWREMHHLV

219 SGSGGVWREMHHLVE

220 GSGGVWREMHHLVEF

221 SGGVWREMHHLVEFE

222 GGVWREMHHLVEFEP

**Allele: HLA-DPA10201-DPB10501. Number of high binders 4.**

281 VSCRVKLSALTLKGT

282 SCRVKLSALTLKGTS

283 CRVKLSALTLKGTSY

284 RVKLSALTLKGTSYK

**Allele: HLA-DPA10201-DPB11401. Number of high binders 6.**

279 GHVSCRVKLSALTLK

280 HVSCRVKLSALTLKG

281 VSCRVKLSALTLKGT

282 SCRVKLSALTLKGTS

283 CRVKLSALTLKGTSY

284 RVKLSALTLKGTSYK
