# Supplementary material for: Advancing molecular modeling and reverse vaccinology in broad-spectrum yellow fever virus vaccine development
Source: Sci Rep. 2024 May 12;14:10842. doi: 10.1038/s41598-024-60680-9 (PMC11089047; doi:10.1038/s41598-024-60680-9)
Supplement: Supplementary file 1 — Supplementary Information. [file 41598_2024_60680_MOESM1_ESM.zip › Yellow_Fever_data/2_Prediction of T-cell epitopes/MHC CLASS II/NETMHCII M.docx]

**Proteína M**

Allele: DRB1_0101. Number of high binders 1.

38 - RNPFFAVTALAIAYL

Allele: DRB1_0301. Number of high binders 9

24 MGERQLQKIERWLVR

25 GERQLQKIERWLVRN

26 ERQLQKIERWLVRNP

27 RQLQKIERWLVRNPF

47 LAIAYLVGSNMTQRV

48 AIAYLVGSNMTQRVV

49 IAYLVGSNMTQRVVI

50 AYLVGSNMTQRVVIA

51 YLVGSNMTQRVVIAL

Allele: DRB1_0401. Number of high binders 3

47 LAIAYLVGSNMTQRV

48 AIAYLVGSNMTQRVV

49 IAYLVGSNMTQRVVI

Allele: DRB1_0405. Number of high binders 0.

NENHUM

Allele: DRB1_0701. Number of high binders 5.

35 WLVRNPFFAVTALAI

36 LVRNPFFAVTALAIA

37 VRNPFFAVTALAIAY

38 RNPFFAVTALAIAYL

39 NPFFAVTALAIAYLV

Allele: DRB1_0802. Number of high binders 2

60 RVVIALLVLAVGPAY

61 VVIALLVLAVGPAYS

Allele: DRB1_0901. Number of high binders 13.

35 WLVRNPFFAVTALAI

36 LVRNPFFAVTALAIA

37 VRNPFFAVTALAIAY

38 RNPFFAVTALAIAYL

39 NPFFAVTALAIAYLV

40 PFFAVTALAIAYLVG

43 AVTALAIAYLVGSNM

44 VTALAIAYLVGSNMT

45 TALAIAYLVGSNMTQ

46 ALAIAYLVGSNMTQR

47 LAIAYLVGSNMTQRV

48 AIAYLVGSNMTQRVV

61 VVIALLVLAVGPAYS

Allele: RB1_1101D. Number of high binders 5.

24 MGERQLQKIERWLVR

25 GERQLQKIERWLVRN

26 ERQLQKIERWLVRNP

27 RQLQKIERWLVRNPF

28 QLQKIERWLVRNPFF

Allele: DRB1_1201. Number of high binders 0.

NENHUM

Allele: DRB1_1301. Number of high binders 4

48 AIAYLVGSNMTQRVV

49 IAYLVGSNMTQRVVI

50 AYLVGSNMTQRVVIA

51 YLVGSNMTQRVVIAL

Allele: DRB1_1501. Number of high binders 0.

NENHUM

Allele: DRB3_0101. Number of high binders 4

35 WLVRNPFFAVTALAI

36 LVRNPFFAVTALAIA

37 VRNPFFAVTALAIAY

38 RNPFFAVTALAIAYL

Allele: DRB3_0202. Number of high binders 14.

31 KIERWLVRNPFFAVT

32 IERWLVRNPFFAVTA

33 ERWLVRNPFFAVTAL

34 RWLVRNPFFAVTALA

35 WLVRNPFFAVTALAI

36 LVRNPFFAVTALAIA

37 VRNPFFAVTALAIAY

38 RNPFFAVTALAIAYL

39 NPFFAVTALAIAYLV

47 LAIAYLVGSNMTQRV

48 AIAYLVGSNMTQRVV

49 IAYLVGSNMTQRVVI

50 AYLVGSNMTQRVVIA

51 YLVGSNMTQRVVIAL

Allele: DRB4_0101. Number of high binders 0.

NENHUM

Allele: DRB5_0101. Number of high binders 0.

NENHUM

Allele: HLA-DQA10501-DQB10201. Number of high binders 0

NENHUM

Allele: HLA-DQA10501-DQB10301. Number of high binders 0.

NENHUM

Allele: HLA-DQA10301-DQB10302. Number of high binders 0.

NENHUM

Allele: HLA-DQA10401-DQB10402. Number of high binders 0.

NENHUM

Allele: HLA-DQA10101-DQB10501. Number of high binders 0.

NENHUM

Allele: HLA-DQA10102-DQB10602. Number of high binders 0.

NENHUM

Allele: HLA-DPA10201-DPB10101. Number of high binders 0.

NENHUM

Allele: HLA-DPA10103-DPB10201. Number of high binders 0.

NENHUM

Allele: HLA-DPA10103-DPB10401. Number of high binders 7.

30 QKIERWLVRNPFFAV

31 KIERWLVRNPFFAVT

32 IERWLVRNPFFAVTA

33 ERWLVRNPFFAVTAL

34 RWLVRNPFFAVTALA

35 WLVRNPFFAVTALAI

36 LVRNPFFAVTALAIA

Allele: HLA-DPA10103-DPB10402. Number of high binders 0.

NENHUM

Allele: HLA-DPA10201-DPB10501. Number of high binders 0.

NENHUM

Allele: HLA-DPA10201-DPB11401. Number of high binders 7.

35 WLVRNPFFAVTALAI

36 LVRNPFFAVTALAIA

37 VRNPFFAVTALAIAY

38 RNPFFAVTALAIAYL

39 NPFFAVTALAIAYLV

40 PFFAVTALAIAYLVG

41 FFAVTALAIAYLVGS
