# Supplementary material for: Advancing molecular modeling and reverse vaccinology in broad-spectrum yellow fever virus vaccine development
Source: Sci Rep. 2024 May 12;14:10842. doi: 10.1038/s41598-024-60680-9 (PMC11089047; doi:10.1038/s41598-024-60680-9)
Supplement: Supplementary file 1 — Supplementary Information. [file 41598_2024_60680_MOESM1_ESM.zip › Yellow_Fever_data/2_Prediction of T-cell epitopes/MHC CLASS II/ALELOS MHC2.docx]

DRB1_0101,DRB1_0301,DRB1_0401,DRB1_0405,DRB1_0701,DRB1_0802,DRB1_0901,DRB1_1101,DRB1_1201,DRB1_1302,DRB1_1501

DRB3_0101,DRB3_0202,

DRB4_0101,

DRB5_0101

HLA-DQA10501-DQB10201,HLA-DQA10501-DQB10301,HLA-DQA10301-DQB10302,HLA-DQA10401-DQB10402,HLA-DQA10101-DQB10501,HLA-DQA10102-DQB10602

HLA-DPA10201-DPB10101,HLA-DPA10103-DPB10201,HLA-DPA10103-DPB10401,HLA-DPA10301-DPB10402,HLA-DPA10201-DPB10501,HLA-DPA10201-DPB11401
