# Supplementary material for: Advancing molecular modeling and reverse vaccinology in broad-spectrum yellow fever virus vaccine development
Source: Sci Rep. 2024 May 12;14:10842. doi: 10.1038/s41598-024-60680-9 (PMC11089047; doi:10.1038/s41598-024-60680-9)
Supplement: Supplementary file 1 — Supplementary Information. [file 41598_2024_60680_MOESM1_ESM.zip › Yellow_Fever_data/2_Prediction of T-cell epitopes/MHC CLASS II/NETMHCII NS5.docx]

**Proteína NS5**

**Allele: DRB1_0101. Number of high binders 8.**

66 HERGYVKLEGRVTDL

67 ERGYVKLEGRVTDLG

68 RGYVKLEGRVTDLGC

575 KNKVVKVLRPAPGGK

576 NKVVKVLRPAPGGKA

577 KVVKVLRPAPGGKAY

578 VVKVLRPAPGGKAYM

619 VQLIRMAEAEMVIHH

**Allele: DRB1_0301. Number of high binders 54.**

28 KRTDIVEVDRDTARR

29 RTDIVEVDRDTARRH

30 TDIVEVDRDTARRHL

31 DIVEVDRDTARRHLA

32 IVEVDRDTARRHLAE

51 TGVAVSRGTAKLRWF

52 GVAVSRGTAKLRWFH

226 RSNIAFTVNQTSRLL

227 SNIAFTVNQTSRLLM

228 NIAFTVNQTSRLLMR

229 IAFTVNQTSRLLMRR

230 AFTVNQTSRLLMRRM

231 FTVNQTSRLLMRRMR

232 TVNQTSRLLMRRMRR

247 PTGKVTLEADVILPI

248 TGKVTLEADVILPIG

249 GKVTLEADVILPIGT

250 KVTLEADVILPIGTR

251 VTLEADVILPIGTRS

323 NGVIKILTYPWDRIE

324 GVIKILTYPWDRIEE

331 YPWDRIEEVTRMAMT

332 PWDRIEEVTRMAMTD

333 WDRIEEVTRMAMTDT

334 DRIEEVTRMAMTDTT

371 TRKIMKVVNRWLFRH

372 RKIMKVVNRWLFRHL

489 ALGFLNEDHWASREN

490 LGFLNEDHWASRENS

513 LQHLGYVIRDLAALE

514 QHLGYVIRDLAALEG

515 HLGYVIRDLAALEGG

516 LGYVIRDLAALEGGG

566 ALAVMEMTYKNKVVK

567 LAVMEMTYKNKVVKV

568 AVMEMTYKNKVVKVL

569 VMEMTYKNKVVKVLR

585 APGGKAYMDVISRRD

586 PGGKAYMDVISRRDQ

587 GGKAYMDVISRRDQR

588 GKAYMDVISRRDQRG

589 KAYMDVISRRDQRGS

619 VQLIRMAEAEMVIHH

766 WSLMYFHKRDMRLLS

767 SLMYFHKRDMRLLSL

768 LMYFHKRDMRLLSLA

769 MYFHKRDMRLLSLAV

770 YFHKRDMRLLSLAVS

771 FHKRDMRLLSLAVSS

863 WASHIHLVIHRIRTL

864 ASHIHLVIHRIRTLI

865 SHIHLVIHRIRTLIG

866 HIHLVIHRIRTLIGK

867 IHLVIHRIRTLIGKE

**Allele: DRB1_0401. Number of high binders 21**

225 ARSNIAFTVNQTSRL

226 RSNIAFTVNQTSRLL

227 SNIAFTVNQTSRLLM

339 VTRMAMTDTTPFGQQ

340 TRMAMTDTTPFGQQR

525 ALEGGGFYADDTAGW

526 LEGGGFYADDTAGWD

527 EGGGFYADDTAGWDT

528 GGGFYADDTAGWDTR

529 GGFYADDTAGWDTRI

530 GFYADDTAGWDTRIT

586 PGGKAYMDVISRRDQ

587 GGKAYMDVISRRDQR

588 GKAYMDVISRRDQRG

589 KAYMDVISRRDQRGS

674 IDDRFGLALSHLNAM

675 DDRFGLALSHLNAMS

676 DRFGLALSHLNAMSK

677 RFGLALSHLNAMSKV

867 IHLVIHRIRTLIGKE

868 HLVIHRIRTLIGKEK

**Allele: DRB1_0405. Number of high binders 4**

654 HGCDRLKRMAVSGDD

655 GCDRLKRMAVSGDDC

656 CDRLKRMAVSGDDCV

657 DRLKRMAVSGDDCVV

**Allele: DRB1_0701. Number of high binders 34**

110 HEKPMNVQSLGWNII

111 EKPMNVQSLGWNIIT

112 KPMNVQSLGWNIITF

113 PMNVQSLGWNIITFK

225 ARSNIAFTVNQTSRL

226 RSNIAFTVNQTSRLL

227 SNIAFTVNQTSRLLM

228 NIAFTVNQTSRLLMR

229 IAFTVNQTSRLLMRR

368 PAGTRKIMKVVNRWL

369 AGTRKIMKVVNRWLF

370 GTRKIMKVVNRWLFR

396 TKEEFIAKVRSHAAI

397 KEEFIAKVRSHAAIG

398 EEFIAKVRSHAAIGA

399 EFIAKVRSHAAIGAF

400 FIAKVRSHAAIGAFL

401 IAKVRSHAAIGAFLE

471 GSRAIWYMWLGARYL

605 QVVTYALNTITNLKV

606 VVTYALNTITNLKVQ

607 VTYALNTITNLKVQL

608 TYALNTITNLKVQLI

609 YALNTITNLKVQLIR

610 ALNTITNLKVQLIRM

674 IDDRFGLALSHLNAM

855 IGMTNRATWASHIHL

856 GMTNRATWASHIHLV

857 MTNRATWASHIHLVI

858 TNRATWASHIHLVIH

863 WASHIHLVIHRIRTL

864 ASHIHLVIHRIRTLI

865 SHIHLVIHRIRTLIG

866 HIHLVIHRIRTLIGK

**Allele:DRB1_0802. Number of high binders 28**

370 GTRKIMKVVNRWLFR

371 TRKIMKVVNRWLFRH

372 RKIMKVVNRWLFRHL

373 KIMKVVNRWLFRHLA

394 LCTKEEFIAKVRSHA

395 CTKEEFIAKVRSHAA

396 TKEEFIAKVRSHAAI

397 KEEFIAKVRSHAAIG

398 EEFIAKVRSHAAIGA

399 EFIAKVRSHAAIGAF

400 FIAKVRSHAAIGAFL

573 TYKNKVVKVLRPAPG

574 YKNKVVKVLRPAPGG

575 KNKVVKVLRPAPGGK

576 NKVVKVLRPAPGGKA

577 KVVKVLRPAPGGKAY

613 TITNLKVQLIRMAEA

615 TNLKVQLIRMAEAEM

774 RDMRLLSLAVSSAVP

775 DMRLLSLAVSSAVPT

776 MRLLSLAVSSAVPTS

777 RLLSLAVSSAVPTSW

865 SHIHLVIHRIRTLIG

866 HIHLVIHRIRTLIGK

867 IHLVIHRIRTLIGKE

868 HLVIHRIRTLIGKEK

869 LVIHRIRTLIGKEKY

870 VIHRIRTLIGKEKYT

**Allele: DRB1_0901. Number of high binders 52**

82 CGRGGWCYYAAAQKE

83 GRGGWCYYAAAQKEV

84 RGGWCYYAAAQKEVS

85 GGWCYYAAAQKEVSG

86 GWCYYAAAQKEVSGV

87 WCYYAAAQKEVSGVK

111 EKPMNVQSLGWNIIT

112 KPMNVQSLGWNIITF

113 PMNVQSLGWNIITFK

174 CGVESFCVKVLAPYM

175 GVESFCVKVLAPYMP

176 VESFCVKVLAPYMPD

178 SFCVKVLAPYMPDVL

179 FCVKVLAPYMPDVLE

215 STHEMYYVSGARSNI

216 THEMYYVSGARSNIA

217 HEMYYVSGARSNIAF

218 EMYYVSGARSNIAFT

398 EEFIAKVRSHAAIGA

399 EFIAKVRSHAAIGAF

400 FIAKVRSHAAIGAFL

401 IAKVRSHAAIGAFLE

402 AKVRSHAAIGAFLEE

471 GSRAIWYMWLGARYL

472 SRAIWYMWLGARYLE

473 RAIWYMWLGARYLEF

474 AIWYMWLGARYLEFE

475 IWYMWLGARYLEFEA

616 NLKVQLIRMAEAEMV

617 LKVQLIRMAEAEMVI

618 KVQLIRMAEAEMVIH

619 VQLIRMAEAEMVIHH

620 QLIRMAEAEMVIHHQ

672 RPIDDRFGLALSHLN

673 PIDDRFGLALSHLNA

674 IDDRFGLALSHLNAM

675 DDRFGLALSHLNAMS

676 DRFGLALSHLNAMSK

677 RFGLALSHLNAMSKV

755 TACLSKAYANMWSLM

756 ACLSKAYANMWSLMY

757 CLSKAYANMWSLMYF

773 KRDMRLLSLAVSSAV

774 RDMRLLSLAVSSAVP

775 DMRLLSLAVSSAVPT

776 MRLLSLAVSSAVPTS

777 RLLSLAVSSAVPTSW

778 LLSLAVSSAVPTSWV

779 LSLAVSSAVPTSWVP

856 GMTNRATWASHIHLV

857 MTNRATWASHIHLVI

858 TNRATWASHIHLVIH

**Allele: DRB1_1101. Number of high binders 71**

85 GGWCYYAAAQKEVSG

86 GWCYYAAAQKEVSGV

87 WCYYAAAQKEVSGVK

88 CYYAAAQKEVSGVKG

189 PDVLEKLELLQRRFG

190 DVLEKLELLQRRFGG

191 VLEKLELLQRRFGGT

192 LEKLELLQRRFGGTV

193 EKLELLQRRFGGTVI

194 KLELLQRRFGGTVIR

195 LELLQRRFGGTVIRN

217 HEMYYVSGARSNIAF

229 IAFTVNQTSRLLMRR

230 AFTVNQTSRLLMRRM

231 FTVNQTSRLLMRRMR

232 TVNQTSRLLMRRMRR

233 VNQTSRLLMRRMRRP

234 NQTSRLLMRRMRRPT

235 QTSRLLMRRMRRPTG

236 TSRLLMRRMRRPTGK

237 SRLLMRRMRRPTGKV

238 RLLMRRMRRPTGKVT

239 LLMRRMRRPTGKVTL

240 LMRRMRRPTGKVTLE

301 YRTWHYCGSYVTRTS

375 MKVVNRWLFRHLARE

376 KVVNRWLFRHLAREK

377 VVNRWLFRHLAREKN

378 VNRWLFRHLAREKNP

379 NRWLFRHLAREKNPR

380 RWLFRHLAREKNPRL

381 WLFRHLAREKNPRLC

382 LFRHLAREKNPRLCT

394 LCTKEEFIAKVRSHA

395 CTKEEFIAKVRSHAA

396 TKEEFIAKVRSHAAI

397 KEEFIAKVRSHAAIG

398 EEFIAKVRSHAAIGA

399 EFIAKVRSHAAIGAF

447 RCRTCVYNMMGKREK

448 CRTCVYNMMGKREKK

449 RTCVYNMMGKREKKL

450 TCVYNMMGKREKKLS

451 CVYNMMGKREKKLSE

452 VYNMMGKREKKLSEF

552 QEILNYMSPHHRKLA

553 EILNYMSPHHRKLAL

554 ILNYMSPHHRKLALA

555 LNYMSPHHRKLALAV

575 KNKVVKVLRPAPGGK

576 NKVVKVLRPAPGGKA

577 KVVKVLRPAPGGKAY

676 DRFGLALSHLNAMSK

680 LALSHLNAMSKVRKD

682 LSHLNAMSKVRKDIS

759 SKAYANMWSLMYFHK

768 LMYFHKRDMRLLSLA

769 MYFHKRDMRLLSLAV

770 YFHKRDMRLLSLAVS

771 FHKRDMRLLSLAVSS

772 HKRDMRLLSLAVSSA

773 KRDMRLLSLAVSSAV

774 RDMRLLSLAVSSAVP

775 DMRLLSLAVSSAVPT

850 LCGSLIGMTNRATWA

851 CGSLIGMTNRATWAS

852 GSLIGMTNRATWASH

868 HLVIHRIRTLIGKEK

869 LVIHRIRTLIGKEKY

870 VIHRIRTLIGKEKYT

871 IHRIRTLIGKEKYTD

**Allele: DRB1_1201. Number of high binders 12.**

188 MPDVLEKLELLQRRF

189 PDVLEKLELLQRRFG

319 ASMINGVIKILTYPW

320 SMINGVIKILTYPWD

321 MINGVIKILTYPWDR

322 INGVIKILTYPWDRI

864 ASHIHLVIHRIRTLI

865 SHIHLVIHRIRTLIG

866 HIHLVIHRIRTLIGK

867 IHLVIHRIRTLIGKE

868 HLVIHRIRTLIGKEK

869 LVIHRIRTLIGKEKY

**Allele: DRB1_1302. Number of high binders 5.**

567 LAVMEMTYKNKVVKV

568 AVMEMTYKNKVVKVL

569 VMEMTYKNKVVKVLR

570 MEMTYKNKVVKVLRP

571 EMTYKNKVVKVLRPA

**Allele: DRB1_1501. Number of high binders 13**

370 GTRKIMKVVNRWLFR

371 TRKIMKVVNRWLFRH

372 RKIMKVVNRWLFRHL

373 KIMKVVNRWLFRHLA

398 EEFIAKVRSHAAIGA

399 EFIAKVRSHAAIGAF

548 LDDEQEILNYMSPHH

549 DDEQEILNYMSPHHR

550 DEQEILNYMSPHHRK

551 EQEILNYMSPHHRKL

552 QEILNYMSPHHRKLA

553 EILNYMSPHHRKLAL

554 ILNYMSPHHRKLALA

**Allele: DRB3_0101. Number of high binders 21**

181 VKVLAPYMPDVLEKL

182 KVLAPYMPDVLEKLE

183 VLAPYMPDVLEKLEL

184 LAPYMPDVLEKLELL

185 APYMPDVLEKLELLQ

227 SNIAFTVNQTSRLLM

247 PTGKVTLEADVILPI

248 TGKVTLEADVILPIG

249 GKVTLEADVILPIGT

250 KVTLEADVILPIGTR

251 VTLEADVILPIGTRS

479 WLGARYLEFEALGFL

480 LGARYLEFEALGFLN

481 GARYLEFEALGFLNE

526 LEGGGFYADDTAGWD

527 EGGGFYADDTAGWDT

528 GGGFYADDTAGWDTR

529 GGFYADDTAGWDTRI

530 GFYADDTAGWDTRIT

758 LSKAYANMWSLMYFH

759 SKAYANMWSLMYFHK

**Allele: DRB3_0202. Number of high binders 51**

200 RRFGGTVIRNPLSRN

201 RFGGTVIRNPLSRNS

202 FGGTVIRNPLSRNST

203 GGTVIRNPLSRNSTH

204 GTVIRNPLSRNSTHE

215 STHEMYYVSGARSNI

216 THEMYYVSGARSNIA

217 HEMYYVSGARSNIAF

218 EMYYVSGARSNIAFT

225 ARSNIAFTVNQTSRL

226 RSNIAFTVNQTSRLL

227 SNIAFTVNQTSRLLM

228 NIAFTVNQTSRLLMR

229 IAFTVNQTSRLLMRR

230 AFTVNQTSRLLMRRM

231 FTVNQTSRLLMRRMR

291 ATWFHDNDNPYRTWH

445 QGRCRTCVYNMMGKR

446 GRCRTCVYNMMGKRE

447 RCRTCVYNMMGKREK

448 CRTCVYNMMGKREKK

569 VMEMTYKNKVVKVLR

570 MEMTYKNKVVKVLRP

601 RGSGQVVTYALNTIT

602 GSGQVVTYALNTITN

603 SGQVVTYALNTITNL

604 GQVVTYALNTITNLK

605 QVVTYALNTITNLKV

606 VVTYALNTITNLKVQ

607 VTYALNTITNLKVQL

608 TYALNTITNLKVQLI

619 VQLIRMAEAEMVIHH

674 IDDRFGLALSHLNAM

675 DDRFGLALSHLNAMS

676 DRFGLALSHLNAMSK

759 SKAYANMWSLMYFHK

766 WSLMYFHKRDMRLLS

767 SLMYFHKRDMRLLSL

768 LMYFHKRDMRLLSLA

769 MYFHKRDMRLLSLAV

775 DMRLLSLAVSSAVPT

776 MRLLSLAVSSAVPTS

777 RLLSLAVSSAVPTSW

778 LLSLAVSSAVPTSWV

779 LSLAVSSAVPTSWVP

815 EVWNRVWITNNPHMQ

816 VWNRVWITNNPHMQD

817 WNRVWITNNPHMQDK

818 NRVWITNNPHMQDKT

819 RVWITNNPHMQDKTT

820 VWITNNPHMQDKTTV

**Allele: DRB4_0101. Number of high binders 18**

552 QEILNYMSPHHRKLA

611 LNTITNLKVQLIRMA

612 NTITNLKVQLIRMAE

613 TITNLKVQLIRMAEA

614 ITNLKVQLIRMAEAE

615 TNLKVQLIRMAEAEM

616 NLKVQLIRMAEAEMV

617 LKVQLIRMAEAEMVI

618 KVQLIRMAEAEMVIH

769 MYFHKRDMRLLSLAV

770 YFHKRDMRLLSLAVS

771 FHKRDMRLLSLAVSS

772 HKRDMRLLSLAVSSA

773 KRDMRLLSLAVSSAV

774 RDMRLLSLAVSSAVP

865 SHIHLVIHRIRTLIG

866 HIHLVIHRIRTLIGK

867 IHLVIHRIRTLIGKE

**Allele: DRB5_0101. Number of high binders 86**

49 VDTGVAVSRGTAKLR

50 DTGVAVSRGTAKLRW

51 TGVAVSRGTAKLRWF

52 GVAVSRGTAKLRWFH

53 VAVSRGTAKLRWFHE

54 AVSRGTAKLRWFHER

59 TAKLRWFHERGYVKL

60 AKLRWFHERGYVKLE

61 KLRWFHERGYVKLEG

62 LRWFHERGYVKLEGR

63 RWFHERGYVKLEGRV

82 CGRGGWCYYAAAQKE

83 GRGGWCYYAAAQKEV

84 RGGWCYYAAAQKEVS

85 GGWCYYAAAQKEVSG

86 GWCYYAAAQKEVSGV

215 STHEMYYVSGARSNI

216 THEMYYVSGARSNIA

217 HEMYYVSGARSNIAF

218 EMYYVSGARSNIAFT

231 FTVNQTSRLLMRRMR

232 TVNQTSRLLMRRMRR

233 VNQTSRLLMRRMRRP

234 NQTSRLLMRRMRRPT

236 TSRLLMRRMRRPTGK

237 SRLLMRRMRRPTGKV

238 RLLMRRMRRPTGKVT

239 LLMRRMRRPTGKVTL

240 LMRRMRRPTGKVTLE

316 GSAASMINGVIKILT

377 VVNRWLFRHLAREKN

378 VNRWLFRHLAREKNP

379 NRWLFRHLAREKNPR

380 RWLFRHLAREKNPRL

381 WLFRHLAREKNPRLC

382 LFRHLAREKNPRLCT

396 TKEEFIAKVRSHAAI

447 RCRTCVYNMMGKREK

448 CRTCVYNMMGKREKK

449 RTCVYNMMGKREKKL

450 TCVYNMMGKREKKLS

451 CVYNMMGKREKKLSE

452 VYNMMGKREKKLSEF

549 DDEQEILNYMSPHHR

550 DEQEILNYMSPHHRK

551 EQEILNYMSPHHRKL

552 QEILNYMSPHHRKLA

553 EILNYMSPHHRKLAL

554 ILNYMSPHHRKLALA

555 LNYMSPHHRKLALAV

566 ALAVMEMTYKNKVVK

575 KNKVVKVLRPAPGGK

576 NKVVKVLRPAPGGKA

577 KVVKVLRPAPGGKAY

578 VVKVLRPAPGGKAYM

579 VKVLRPAPGGKAYMD

580 KVLRPAPGGKAYMDV

674 IDDRFGLALSHLNAM

675 DDRFGLALSHLNAMS

676 DRFGLALSHLNAMSK

677 RFGLALSHLNAMSKV

678 FGLALSHLNAMSKVR

679 GLALSHLNAMSKVRK

680 LALSHLNAMSKVRKD

681 ALSHLNAMSKVRKDI

682 LSHLNAMSKVRKDIS

683 SHLNAMSKVRKDISE

757 CLSKAYANMWSLMYF

758 LSKAYANMWSLMYFH

759 SKAYANMWSLMYFHK

760 KAYANMWSLMYFHKR

761 AYANMWSLMYFHKRD

762 YANMWSLMYFHKRDM

763 ANMWSLMYFHKRDMR

764 NMWSLMYFHKRDMRL

765 MWSLMYFHKRDMRLL

766 WSLMYFHKRDMRLLS

832 TTVKEWRDIPYLTKR

833 TVKEWRDIPYLTKRQ

834 VKEWRDIPYLTKRQD

835 KEWRDIPYLTKRQDK

868 HLVIHRIRTLIGKEK

869 LVIHRIRTLIGKEKY

870 VIHRIRTLIGKEKYT

871 IHRIRTLIGKEKYTD

872 HRIRTLIGKEKYTDY

**Allele: HLA-DQA10501-DQB10201. Number of high binders 30.**

246 RPTGKVTLEADVILP

247 PTGKVTLEADVILPI

248 TGKVTLEADVILPIG

249 GKVTLEADVILPIGT

404 VRSHAAIGAFLEEQE

405 RSHAAIGAFLEEQEQ

406 SHAAIGAFLEEQEQW

407 HAAIGAFLEEQEQWK

426 AVQDPKFWELVDEER

427 VQDPKFWELVDEERR

428 QDPKFWELVDEERRL

476 WYMWLGARYLEFEAL

477 YMWLGARYLEFEALG

478 MWLGARYLEFEALGF

479 WLGARYLEFEALGFL

539 WDTRITEADLDDEQE

540 DTRITEADLDDEQEI

616 NLKVQLIRMAEAEMV

617 LKVQLIRMAEAEMVI

618 KVQLIRMAEAEMVIH

801 VHGKGEWMTTEDMLE

802 HGKGEWMTTEDMLEV

803 GKGEWMTTEDMLEVW

804 KGEWMTTEDMLEVWN

805 GEWMTTEDMLEVWNR

806 EWMTTEDMLEVWNRV

887 LTVMDRYSVDADLQP

888 TVMDRYSVDADLQPG

889 VMDRYSVDADLQPGE

890 MDRYSVDADLQPGEL

**Allele: HLA-DQA10501-DQB10301. Number of high binders 12**

216 THEMYYVSGARSNIA

217 HEMYYVSGARSNIAF

218 EMYYVSGARSNIAFT

219 MYYVSGARSNIAFTV

220 YYVSGARSNIAFTVN

221 YVSGARSNIAFTVNQ

222 VSGARSNIAFTVNQT

777 RLLSLAVSSAVPTSW

778 LLSLAVSSAVPTSWV

779 LSLAVSSAVPTSWVP

780 SLAVSSAVPTSWVPQ

781 LAVSSAVPTSWVPQG

**Allele: HLA-DQA10301-DQB10302. Number of high binders 8.**

401 IAKVRSHAAIGAFLE

402 AKVRSHAAIGAFLEE

403 KVRSHAAIGAFLEEQ

404 VRSHAAIGAFLEEQE

405 RSHAAIGAFLEEQEQ

406 SHAAIGAFLEEQEQW

407 HAAIGAFLEEQEQWK

408 AAIGAFLEEQEQWKT

**Allele: HLA-DQA10401-DQB10402. Number of high binders 9**

402 AKVRSHAAIGAFLEE

403 KVRSHAAIGAFLEEQ

404 VRSHAAIGAFLEEQE

405 RSHAAIGAFLEEQEQ

406 SHAAIGAFLEEQEQW

407 HAAIGAFLEEQEQWK

408 AAIGAFLEEQEQWKT

482 ARYLEFEALGFLNED

483 RYLEFEALGFLNEDH

**Allele: HLA-DQA10101-DQB10501. Number of high binders 4**

829 QDKTTVKEWRDIPYL

830 DKTTVKEWRDIPYLT

831 KTTVKEWRDIPYLTK

832 TTVKEWRDIPYLTKR

**Allele: HLA-DQA10102-DQB10602. Number of high binders 17.**

219 MYYVSGARSNIAFTV

220 YYVSGARSNIAFTVN

221 YVSGARSNIAFTVNQ

311 VTRTSGSAASMINGV

312 TRTSGSAASMINGVI

313 RTSGSAASMINGVIK

314 TSGSAASMINGVIKI

315 SGSAASMINGVIKIL

316 GSAASMINGVIKILT

404 VRSHAAIGAFLEEQE

405 RSHAAIGAFLEEQEQ

613 TITNLKVQLIRMAEA

614 ITNLKVQLIRMAEAE

615 TNLKVQLIRMAEAEM

616 NLKVQLIRMAEAEMV

617 LKVQLIRMAEAEMVI

618 KVQLIRMAEAEMVIH

**Allele: HLA-DPA10201-DPB10101. Number of high binders 4**

476 WYMWLGARYLEFEAL

481 GARYLEFEALGFLNE

482 ARYLEFEALGFLNED

483 RYLEFEALGFLNEDH

**Allele: HLA-DPA10103-DPB10201. Number of high binders 11**

473 RAIWYMWLGARYLEF

474 AIWYMWLGARYLEFE

475 IWYMWLGARYLEFEA

476 WYMWLGARYLEFEAL

477 YMWLGARYLEFEALG

478 MWLGARYLEFEALGF

479 WLGARYLEFEALGFL

480 LGARYLEFEALGFLN

481 GARYLEFEALGFLNE

482 ARYLEFEALGFLNED

483 RYLEFEALGFLNEDH

**Allele: HLA-DPA10103-DPB10401. Number of high binders 15**

473 RAIWYMWLGARYLEF

474 AIWYMWLGARYLEFE

475 IWYMWLGARYLEFEA

476 WYMWLGARYLEFEAL

477 YMWLGARYLEFEALG

478 MWLGARYLEFEALGF

479 WLGARYLEFEALGFL

480 LGARYLEFEALGFLN

481 GARYLEFEALGFLNE

482 ARYLEFEALGFLNED

483 RYLEFEALGFLNEDH

484 YLEFEALGFLNEDHW

485 LEFEALGFLNEDHWA

760 KAYANMWSLMYFHKR

762 YANMWSLMYFHKRDM

**Allele: HLA-DPA10301-DPB10402. Number of high binders 11.**

184 LAPYMPDVLEKLELL

185 APYMPDVLEKLELLQ

186 PYMPDVLEKLELLQR

187 YMPDVLEKLELLQRR

188 MPDVLEKLELLQRRF

481 GARYLEFEALGFLNE

482 ARYLEFEALGFLNED

483 RYLEFEALGFLNEDH

484 YLEFEALGFLNEDHW

676 DRFGLALSHLNAMSK

677 RFGLALSHLNAMSKV

**Allele: HLA-DPA10201-DPB10501. Number of high binders 0.**

**Allele: HLA-DPA10201-DPB11401. Number of high binders 17**

400 FIAKVRSHAAIGAFL

401 IAKVRSHAAIGAFLE

402 AKVRSHAAIGAFLEE

557 YMSPHHRKLALAVME

558 MSPHHRKLALAVMEM

559 SPHHRKLALAVMEMT

560 PHHRKLALAVMEMTY

561 HHRKLALAVMEMTYK

562 HRKLALAVMEMTYKN

563 RKLALAVMEMTYKNK

617 LKVQLIRMAEAEMVI

618 KVQLIRMAEAEMVIH

619 VQLIRMAEAEMVIHH

620 QLIRMAEAEMVIHHQ

621 LIRMAEAEMVIHHQH

770 YFHKRDMRLLSLAVS

771 FHKRDMRLLSLAVSS
