# Supplementary material for: Advancing molecular modeling and reverse vaccinology in broad-spectrum yellow fever virus vaccine development
Source: Sci Rep. 2024 May 12;14:10842. doi: 10.1038/s41598-024-60680-9 (PMC11089047; doi:10.1038/s41598-024-60680-9)
Supplement: Supplementary file 1 — Supplementary Information. [file 41598_2024_60680_MOESM1_ESM.zip › Yellow_Fever_data/2_Prediction of T-cell epitopes/MHC CLASS II/NETMHCII NS3.docx]

**Allele: DRB1_0101. Number of high binders 11.**

95 EEVQLIAAAPGKNVV

96 EVQLIAAAPGKNVVN

97 VQLIAAAPGKNVVNV

145 RNGEVIGLYGNGILV

146 NGEVIGLYGNGILVG

147 GEVIGLYGNGILVGD

267 HATLTYRMLEPTRVV

268 ATLTYRMLEPTRVVN

269 TLTYRMLEPTRVVNW

270 LTYRMLEPTRVVNWE

271 TYRMLEPTRVVNWEV

**Allele: DRB1_0301. Number of high binders 85.**

1 SGDVLWDIPTPKIIE

2 GDVLWDIPTPKIIEE

48 FHTMWHVTRGAFLVR

49 HTMWHVTRGAFLVRN

56 RGAFLVRNGKKLVPS

57 GAFLVRNGKKLVPSW

58 AFLVRNGKKLVPSWA

152 LYGNGILVGDNSFVS

153 YGNGILVGDNSFVSA

154 GNGILVGDNSFVSAI

155 NGILVGDNSFVSAIS

156 GILVGDNSFVSAISQ

157 ILVGDNSFVSAISQT

206 RRFLPQILAECARRR

207 RFLPQILAECARRRL

208 FLPQILAECARRRLR

209 LPQILAECARRRLRT

210 PQILAECARRRLRTL

211 QILAECARRRLRTLV

220 RLRTLVLAPTRVVLS

221 LRTLVLAPTRVVLSE

222 RTLVLAPTRVVLSEM

223 TLVLAPTRVVLSEMK

226 LAPTRVVLSEMKEAF

227 APTRVVLSEMKEAFH

228 PTRVVLSEMKEAFHG

229 TRVVLSEMKEAFHGL

230 RVVLSEMKEAFHGLD

262 IDAMCHATLTYRMLE

280 VVNWEVIIMDEAHFL

281 VNWEVIIMDEAHFLD

282 NWEVIIMDEAHFLDP

283 WEVIIMDEAHFLDPA

284 EVIIMDEAHFLDPAS

285 VIIMDEAHFLDPASI

349 WNTGHDWILADKRPT

350 NTGHDWILADKRPTA

351 TGHDWILADKRPTAW

352 GHDWILADKRPTAWF

353 HDWILADKRPTAWFL

354 DWILADKRPTAWFLP

355 WILADKRPTAWFLPS

360 KRPTAWFLPSIRAAN

361 RPTAWFLPSIRAANV

362 PTAWFLPSIRAANVM

363 TAWFLPSIRAANVMA

364 AWFLPSIRAANVMAA

365 WFLPSIRAANVMAAS

366 FLPSIRAANVMAASL

367 LPSIRAANVMAASLR

368 PSIRAANVMAASLRK

369 SIRAANVMAASLRKA

371 RAANVMAASLRKAGK

372 AANVMAASLRKAGKS

373 ANVMAASLRKAGKSV

374 NVMAASLRKAGKSVV

383 AGKSVVVLNRKTFEK

384 GKSVVVLNRKTFEKE

385 KSVVVLNRKTFEKEY

406 KPDFILATDIAEMGA

407 PDFILATDIAEMGAN

408 DFILATDIAEMGANL

409 FILATDIAEMGANLC

432 TAFKPVLVDEGRKVA

433 AFKPVLVDEGRKVAI

434 FKPVLVDEGRKVAIK

435 KPVLVDEGRKVAIKG

447 IKGPLRISASSAAQR

448 KGPLRISASSAAQRR

449 GPLRISASSAAQRRG

450 PLRISASSAAQRRGR

451 LRISASSAAQRRGRI

452 RISASSAAQRRGRIG

492 WLEASMLLDNMEVRG

493 LEASMLLDNMEVRGG

494 EASMLLDNMEVRGGM

495 ASMLLDNMEVRGGMV

496 SMLLDNMEVRGGMVA

497 MLLDNMEVRGGMVAP

523 SPGEMRLRDDQRRVF

524 PGEMRLRDDQRRVFR

525 GEMRLRDDQRRVFRE

526 EMRLRDDQRRVFREL

527 MRLRDDQRRVFRELV

528 RLRDDQRRVFRELVR

**Allele: DRB1_0401. Number of high binders 15**

158 LVGDNSFVSAISQTE

159 VGDNSFVSAISQTEV

160 GDNSFVSAISQTEVK

161 DNSFVSAISQTEVKE

162 NSFVSAISQTEVKEE

163 SFVSAISQTEVKEEG

312 NESATILMTATPPGT

313 ESATILMTATPPGTS

314 SATILMTATPPGTSD

315 ATILMTATPPGTSDE

316 TILMTATPPGTSDEF

406 KPDFILATDIAEMGA

407 PDFILATDIAEMGAN

448 KGPLRISASSAAQRR

449 GPLRISASSAAQRRG

**Allele: DRB1_0405. Number of high binders 11**

158 LVGDNSFVSAISQTE

159 VGDNSFVSAISQTEV

160 GDNSFVSAISQTEVK

161 DNSFVSAISQTEVKE

162 NSFVSAISQTEVKEE

163 SFVSAISQTEVKEEG

314 SATILMTATPPGTSD

404 QKKPDFILATDIAEM

405 KKPDFILATDIAEMG

406 KPDFILATDIAEMGA

407 PDFILATDIAEMGAN

**Allele: DRB1_0701. Number of high binders 29.**

46 GVFHTMWHVTRGAFL

47 VFHTMWHVTRGAFLV

48 FHTMWHVTRGAFLVR

49 HTMWHVTRGAFLVRN

50 TMWHVTRGAFLVRNG

51 MWHVTRGAFLVRNGK

53 HVTRGAFLVRNGKKL

54 VTRGAFLVRNGKKLV

55 TRGAFLVRNGKKLVP

73 SVKEDLVAYGGSWKL

74 VKEDLVAYGGSWKLE

75 KEDLVAYGGSWKLEG

218 RRRLRTLVLAPTRVV

219 RRLRTLVLAPTRVVL

220 RLRTLVLAPTRVVLS

221 LRTLVLAPTRVVLSE

222 RTLVLAPTRVVLSEM

260 EVIDAMCHATLTYRM

261 VIDAMCHATLTYRML

262 IDAMCHATLTYRMLE

263 DAMCHATLTYRMLEP

264 AMCHATLTYRMLEPT

266 CHATLTYRMLEPTRV

267 HATLTYRMLEPTRVV

268 ATLTYRMLEPTRVVN

269 TLTYRMLEPTRVVNW

270 LTYRMLEPTRVVNWE

271 TYRMLEPTRVVNWEV

272 YRMLEPTRVVNWEVI

**Allele: DRB1_0802. Number of high binders 20.**

96 EVQLIAAAPGKNVVN

217 ARRRLRTLVLAPTRV

218 RRRLRTLVLAPTRVV

219 RRLRTLVLAPTRVVL

220 RLRTLVLAPTRVVLS

221 LRTLVLAPTRVVLSE

222 RTLVLAPTRVVLSEM

223 TLVLAPTRVVLSEMK

311 ANESATILMTATPPG

312 NESATILMTATPPGT

313 ESATILMTATPPGTS

314 SATILMTATPPGTSD

315 ATILMTATPPGTSDE

316 TILMTATPPGTSDEF

317 ILMTATPPGTSDEFP

364 AWFLPSIRAANVMAA

365 WFLPSIRAANVMAAS

447 IKGPLRISASSAAQR

448 KGPLRISASSAAQRR

449 GPLRISASSAAQRRG

**Allele: DRB1_0901. Number of high binders 41.**

21 EDGIYGIFQSTFLGA

22 DGIYGIFQSTFLGAS

23 GIYGIFQSTFLGASQ

46 GVFHTMWHVTRGAFL

47 VFHTMWHVTRGAFLV

48 FHTMWHVTRGAFLVR

49 HTMWHVTRGAFLVRN

50 TMWHVTRGAFLVRNG

62 RNGKKLVPSWASVKE

63 NGKKLVPSWASVKED

64 GKKLVPSWASVKEDL

74 VKEDLVAYGGSWKLE

75 KEDLVAYGGSWKLEG

218 RRRLRTLVLAPTRVV

219 RRLRTLVLAPTRVVL

220 RLRTLVLAPTRVVLS

221 LRTLVLAPTRVVLSE

222 RTLVLAPTRVVLSEM

223 TLVLAPTRVVLSEMK

261 VIDAMCHATLTYRML

262 IDAMCHATLTYRMLE

267 HATLTYRMLEPTRVV

268 ATLTYRMLEPTRVVN

269 TLTYRMLEPTRVVNW

270 LTYRMLEPTRVVNWE

271 TYRMLEPTRVVNWEV

305 AAHRARANESATILM

306 AHRARANESATILMT

307 HRARANESATILMTA

308 RARANESATILMTAT

360 KRPTAWFLPSIRAAN

361 RPTAWFLPSIRAANV

362 PTAWFLPSIRAANVM

363 TAWFLPSIRAANVMA

364 AWFLPSIRAANVMAA

365 WFLPSIRAANVMAAS

445 VAIKGPLRISASSAA

446 AIKGPLRISASSAAQ

447 IKGPLRISASSAAQR

448 KGPLRISASSAAQRR

449 GPLRISASSAAQRRG

**Allele: DRB1_1101. Number of high binders 45.**

26 GIFQSTFLGASQRGV

27 IFQSTFLGASQRGVG

28 FQSTFLGASQRGVGV

29 QSTFLGASQRGVGVA

30 STFLGASQRGVGVAQ

43 AQGGVFHTMWHVTRG

44 QGGVFHTMWHVTRGA

45 GGVFHTMWHVTRGAF

46 GVFHTMWHVTRGAFL

47 VFHTMWHVTRGAFLV

48 FHTMWHVTRGAFLVR

49 HTMWHVTRGAFLVRN

54 VTRGAFLVRNGKKLV

55 TRGAFLVRNGKKLVP

56 RGAFLVRNGKKLVPS

57 GAFLVRNGKKLVPSW

58 AFLVRNGKKLVPSWA

216 CARRRLRTLVLAPTR

217 ARRRLRTLVLAPTRV

218 RRRLRTLVLAPTRVV

219 RRLRTLVLAPTRVVL

220 RLRTLVLAPTRVVLS

221 LRTLVLAPTRVVLSE

350 NTGHDWILADKRPTA

351 TGHDWILADKRPTAW

352 GHDWILADKRPTAWF

353 HDWILADKRPTAWFL

354 DWILADKRPTAWFLP

359 DKRPTAWFLPSIRAA

360 KRPTAWFLPSIRAAN

361 RPTAWFLPSIRAANV

362 PTAWFLPSIRAANVM

363 TAWFLPSIRAANVMA

364 AWFLPSIRAANVMAA

365 WFLPSIRAANVMAAS

371 RAANVMAASLRKAGK

372 AANVMAASLRKAGKS

373 ANVMAASLRKAGKSV

374 NVMAASLRKAGKSVV

375 VMAASLRKAGKSVVV

381 RKAGKSVVVLNRKTF

382 KAGKSVVVLNRKTFE

383 AGKSVVVLNRKTFEK

384 GKSVVVLNRKTFEKE

385 KSVVVLNRKTFEKEY

**Allele: DRB1_1201. Number of high binders 0.**

**Allele: DRB1_1302. Number of high binders 7.**

219 RRLRTLVLAPTRVVL

220 RLRTLVLAPTRVVLS

366 FLPSIRAANVMAASL

535 RVFRELVRNCDLPVW

536 VFRELVRNCDLPVWL

537 FRELVRNCDLPVWLS

538 RELVRNCDLPVWLSW

**Allele: DRB1_1501. Number of high binders 6.**

144 NRNGEVIGLYGNGIL

145 RNGEVIGLYGNGILV

146 NGEVIGLYGNGILVG

147 GEVIGLYGNGILVGD

148 EVIGLYGNGILVGDN

149 VIGLYGNGILVGDNS

**Allele: DRB3_0101. Number of high binders 21**

153 YGNGILVGDNSFVSA

154 GNGILVGDNSFVSAI

155 NGILVGDNSFVSAIS

267 HATLTYRMLEPTRVV

268 ATLTYRMLEPTRVVN

269 TLTYRMLEPTRVVNW

280 VVNWEVIIMDEAHFL

281 VNWEVIIMDEAHFLD

282 NWEVIIMDEAHFLDP

283 WEVIIMDEAHFLDPA

284 EVIIMDEAHFLDPAS

285 VIIMDEAHFLDPASI

431 RTAFKPVLVDEGRKV

432 TAFKPVLVDEGRKVA

433 AFKPVLVDEGRKVAI

434 FKPVLVDEGRKVAIK

435 KPVLVDEGRKVAIKG

486 NAHHVCWLEASMLLD

487 AHHVCWLEASMLLDN

488 HHVCWLEASMLLDNM

489 HVCWLEASMLLDNME

**Allele: DRB3_0202. Number of high binders 37**

47 VFHTMWHVTRGAFLV

48 FHTMWHVTRGAFLVR

49 HTMWHVTRGAFLVRN

50 TMWHVTRGAFLVRNG

54 VTRGAFLVRNGKKLV

55 TRGAFLVRNGKKLVP

56 RGAFLVRNGKKLVPS

57 GAFLVRNGKKLVPSW

58 AFLVRNGKKLVPSWA

62 RNGKKLVPSWASVKE

63 NGKKLVPSWASVKED

64 GKKLVPSWASVKEDL

221 LRTLVLAPTRVVLSE

267 HATLTYRMLEPTRVV

268 ATLTYRMLEPTRVVN

269 TLTYRMLEPTRVVNW

270 LTYRMLEPTRVVNWE

271 TYRMLEPTRVVNWEV

305 AAHRARANESATILM

306 AHRARANESATILMT

351 TGHDWILADKRPTAW

352 GHDWILADKRPTAWF

353 HDWILADKRPTAWFL

354 DWILADKRPTAWFLP

360 KRPTAWFLPSIRAAN

361 RPTAWFLPSIRAANV

362 PTAWFLPSIRAANVM

363 TAWFLPSIRAANVMA

364 AWFLPSIRAANVMAA

365 WFLPSIRAANVMAAS

446 AIKGPLRISASSAAQ

447 IKGPLRISASSAAQR

448 KGPLRISASSAAQRR

449 GPLRISASSAAQRRG

450 PLRISASSAAQRRGR

536 VFRELVRNCDLPVWL

537 FRELVRNCDLPVWLS

**Allele: DRB4_0101. Number of high binders 17.**

121 RNGGEIGAVALDYPS

122 NGGEIGAVALDYPSG

123 GGEIGAVALDYPSGT

124 GEIGAVALDYPSGTS

202 AGKTRRFLPQILAEC

203 GKTRRFLPQILAECA

204 KTRRFLPQILAECAR

205 TRRFLPQILAECARR

214 AECARRRLRTLVLAP

215 ECARRRLRTLVLAPT

216 CARRRLRTLVLAPTR

217 ARRRLRTLVLAPTRV

218 RRRLRTLVLAPTRVV

219 RRLRTLVLAPTRVVL

447 IKGPLRISASSAAQR

448 KGPLRISASSAAQRR

449 GPLRISASSAAQRRG

**Allele: DRB5_0101. Number of high binders 81.**

42 VAQGGVFHTMWHVTR

43 AQGGVFHTMWHVTRG

44 QGGVFHTMWHVTRGA

45 GGVFHTMWHVTRGAF

46 GVFHTMWHVTRGAFL

47 VFHTMWHVTRGAFLV

48 FHTMWHVTRGAFLVR

49 HTMWHVTRGAFLVRN

50 TMWHVTRGAFLVRNG

51 MWHVTRGAFLVRNGK

52 WHVTRGAFLVRNGKK

53 HVTRGAFLVRNGKKL

54 VTRGAFLVRNGKKLV

55 TRGAFLVRNGKKLVP

56 RGAFLVRNGKKLVPS

57 GAFLVRNGKKLVPSW

58 AFLVRNGKKLVPSWA

62 RNGKKLVPSWASVKE

63 NGKKLVPSWASVKED

64 GKKLVPSWASVKEDL

65 KKLVPSWASVKEDLV

95 EEVQLIAAAPGKNVV

96 EVQLIAAAPGKNVVN

106 KNVVNVQTKPSLFKV

107 NVVNVQTKPSLFKVR

108 VVNVQTKPSLFKVRN

109 VNVQTKPSLFKVRNG

176 EGKEELQEIPTMLKK

177 GKEELQEIPTMLKKG

178 KEELQEIPTMLKKGM

179 EELQEIPTMLKKGMT

180 ELQEIPTMLKKGMTT

181 LQEIPTMLKKGMTTI

193 TTILDFHPGAGKTRR

194 TILDFHPGAGKTRRF

195 ILDFHPGAGKTRRFL

196 LDFHPGAGKTRRFLP

206 RRFLPQILAECARRR

207 RFLPQILAECARRRL

208 FLPQILAECARRRLR

209 LPQILAECARRRLRT

210 PQILAECARRRLRTL

211 QILAECARRRLRTLV

217 ARRRLRTLVLAPTRV

218 RRRLRTLVLAPTRVV

219 RRLRTLVLAPTRVVL

220 RLRTLVLAPTRVVLS

221 LRTLVLAPTRVVLSE

267 HATLTYRMLEPTRVV

268 ATLTYRMLEPTRVVN

269 TLTYRMLEPTRVVNW

270 LTYRMLEPTRVVNWE

295 DPASIAARGWAAHRA

296 PASIAARGWAAHRAR

297 ASIAARGWAAHRARA

298 SIAARGWAAHRARAN

299 IAARGWAAHRARANE

300 AARGWAAHRARANES

368 PSIRAANVMAASLRK

369 SIRAANVMAASLRKA

370 IRAANVMAASLRKAG

371 RAANVMAASLRKAGK

372 AANVMAASLRKAGKS

373 ANVMAASLRKAGKSV

374 NVMAASLRKAGKSVV

392 RKTFEKEYPTIKQKK

441 EGRKVAIKGPLRISA

442 GRKVAIKGPLRISAS

448 KGPLRISASSAAQRR

449 GPLRISASSAAQRRG

450 PLRISASSAAQRRGR

451 LRISASSAAQRRGRI

452 RISASSAAQRRGRIG

545 DLPVWLSWQVAKAGL

546 LPVWLSWQVAKAGLK

547 PVWLSWQVAKAGLKT

548 VWLSWQVAKAGLKTN

549 WLSWQVAKAGLKTND

550 LSWQVAKAGLKTNDR

551 SWQVAKAGLKTNDRK

552 WQVAKAGLKTNDRKW

**Allele: HLA-DQA10501-DQB10201. Number of high binders 11.**

120 VRNGGEIGAVALDYP

121 RNGGEIGAVALDYPS

122 NGGEIGAVALDYPSG

123 GGEIGAVALDYPSGT

124 GEIGAVALDYPSGTS

277 PTRVVNWEVIIMDEA

278 TRVVNWEVIIMDEAH

279 RVVNWEVIIMDEAHF

280 VVNWEVIIMDEAHFL

281 VNWEVIIMDEAHFLD

282 NWEVIIMDEAHFLDP

**Allele: HLA-DQA10501-DQB10301. Number of high binders 22**

36 SQRGVGVAQGGVFHT

37 QRGVGVAQGGVFHTM

38 RGVGVAQGGVFHTMW

39 GVGVAQGGVFHTMWH

40 VGVAQGGVFHTMWHV

41 GVAQGGVFHTMWHVT

308 RARANESATILMTAT

309 ARANESATILMTATP

313 ESATILMTATPPGTS

314 SATILMTATPPGTSD

315 ATILMTATPPGTSDE

316 TILMTATPPGTSDEF

365 WFLPSIRAANVMAAS

366 FLPSIRAANVMAASL

367 LPSIRAANVMAASLR

368 PSIRAANVMAASLRK

369 SIRAANVMAASLRKA

370 IRAANVMAASLRKAG

371 RAANVMAASLRKAGK

499 LDNMEVRGGMVAPLY

500 DNMEVRGGMVAPLYG

501 NMEVRGGMVAPLYGI

**Allele: HLA-DQA10301-DQB10302. Number of high binders 7**

158 LVGDNSFVSAISQTE

159 VGDNSFVSAISQTEV

160 GDNSFVSAISQTEVK

161 DNSFVSAISQTEVKE

276 EPTRVVNWEVIIMDE

277 PTRVVNWEVIIMDEA

278 TRVVNWEVIIMDEAH

**Allele: HLA-DQA10401-DQB10402. Number of high binders 9.**

277 PTRVVNWEVIIMDEA

278 TRVVNWEVIIMDEAH

279 RVVNWEVIIMDEAHF

280 VVNWEVIIMDEAHFL

404 QKKPDFILATDIAEM

405 KKPDFILATDIAEMG

406 KPDFILATDIAEMGA

407 PDFILATDIAEMGAN

490 VCWLEASMLLDNMEV

**Allele: HLA-DQA10101-DQB10501. Number of high binders 5**

284 EVIIMDEAHFLDPAS

285 VIIMDEAHFLDPASI

286 IIMDEAHFLDPASIA

287 IMDEAHFLDPASIAA

359 DKRPTAWFLPSIRAA

**Allele: HLA-DQA10102-DQB10602. Number of high binders 20**

307 HRARANESATILMTA

308 RARANESATILMTAT

309 ARANESATILMTATP

310 RANESATILMTATPP

311 ANESATILMTATPPG

312 NESATILMTATPPGT

364 AWFLPSIRAANVMAA

365 WFLPSIRAANVMAAS

366 FLPSIRAANVMAASL

367 LPSIRAANVMAASLR

368 PSIRAANVMAASLRK

369 SIRAANVMAASLRKA

370 IRAANVMAASLRKAG

371 RAANVMAASLRKAGK

372 AANVMAASLRKAGKS

373 ANVMAASLRKAGKSV

488 HHVCWLEASMLLDNM

489 HVCWLEASMLLDNME

490 VCWLEASMLLDNMEV

491 CWLEASMLLDNMEVR

**Allele: HLA-DPA10201-DPB10101. Number of high binders 5**

21 EDGIYGIFQSTFLGA

22 DGIYGIFQSTFLGAS

23 GIYGIFQSTFLGASQ

24 IYGIFQSTFLGASQR

25 YGIFQSTFLGASQRG

**Allele: HLA-DPA10103-DPB10201. Number of high binders 0.**

**Allele: HLA-DPA10103-DPB10401. Number of high binders 6**

21 EDGIYGIFQSTFLGA

22 DGIYGIFQSTFLGAS

23 GIYGIFQSTFLGASQ

24 IYGIFQSTFLGASQR

25 YGIFQSTFLGASQRG

26 GIFQSTFLGASQRGV

**Allele: HLA-DPA10301-DPB10402. Number of high binders 1.**

24 IYGIFQSTFLGASQR

**Allele: HLA-DPA10201-DPB10501. Number of high binders 2.**

608 DQSALADFIKFAEGR

609 QSALADFIKFAEGRR

**Allele: HLA-DPA10201-DPB11401. Number of high binders 9**

214 AECARRRLRTLVLAP

215 ECARRRLRTLVLAPT

216 CARRRLRTLVLAPTR

217 ARRRLRTLVLAPTRV

218 RRRLRTLVLAPTRVV

219 RRLRTLVLAPTRVVL

220 RLRTLVLAPTRVVLS

307 HRARANESATILMTA

308 RARANESATILMTAT
