# Supplementary material for: Advancing molecular modeling and reverse vaccinology in broad-spectrum yellow fever virus vaccine development
Source: Sci Rep. 2024 May 12;14:10842. doi: 10.1038/s41598-024-60680-9 (PMC11089047; doi:10.1038/s41598-024-60680-9)
Supplement: Supplementary file 1 — Supplementary Information. [file 41598_2024_60680_MOESM1_ESM.zip › Yellow_Fever_data/2_Prediction of T-cell epitopes/MHC CLASS II/NETMHC11 NSP1.docx]

**PROTEÍNA NSP1**

**Allele: DRB1_0101. Number of high binders 0.**

**Allele: DRB1_0301. Number of high binders 17.**

84 VDISVVVQDSKNIYQ

85 DISVVVQDSKNIYQR

86 ISVVVQDSKNIYQRG

87 SVVVQDSKNIYQRGT

88 VVVQDSKNIYQRGTH

127 GRKNGSFIIDGKSRK

128 RKNGSFIIDGKSRKE

129 KNGSFIIDGKSRKEC

130 NGSFIIDGKSRKECP

131 GSFIIDGKSRKECPF

132 SFIIDGKSRKECPFS

162 VFTTRVYMDAVFEYT

163 FTTRVYMDAVFEYTM

164 TTRVYMDAVFEYTMD

165 TRVYMDAVFEYTMDC

166 RVYMDAVFEYTMDCD

336 RPKKTHDSHLVRSWV

**Allele: DRB1_0401. Number of high binders 0.**

**Allele: DRB1_0405. Number of high binders 0.**

**Allele: DRB1_0701. Number of high binders 1.**

222 KECEWPLTHTIGTSV

**Allele: DRB1_0802. Number of high binders 3.**

36 PEDPVKLASIVKASF

37 EDPVKLASIVKASFE

38 DPVKLASIVKASFEE

**Allele: DRB1_0901. Number of high binders 8.**

93 SKNIYQRGTHPFSRI

94 KNIYQRGTHPFSRIR

317 TMPPVSFHGSDGCWY

318 MPPVSFHGSDGCWYP

319 PPVSFHGSDGCWYPM

320 PVSFHGSDGCWYPME

321 VSFHGSDGCWYPMEI

322 SFHGSDGCWYPMEIR

**Allele: DRB1_1101. Number of high binders 11.**

93 SKNIYQRGTHPFSRI

94 KNIYQRGTHPFSRIR

95 NIYQRGTHPFSRIRD

115 WKTWGKNLVFSPGRK

110 GLQYGWKTWGKNLVF

111 LQYGWKTWGKNLVFS

112 QYGWKTWGKNLVFSP

113 YGWKTWGKNLVFSPG

118 WGKNLVFSPGRKNGS

119 GKNLVFSPGRKNGSF

120 KNLVFSPGRKNGSFI

**Allele: DRB1_1201. Number of high binders 2.**

208 NGTWMIHTLETLDYK

209 GTWMIHTLETLDYKE

**Allele: DRB1_1302. Number of high binders 0.**

**Allele: DRB1_1501.**

**Allele: DRB3_0101. Number of high binders 15**

128 RKNGSFIIDGKSRKE

129 KNGSFIIDGKSRKEC

130 NGSFIIDGKSRKECP

161 GVFTTRVYMDAVFEY

162 VFTTRVYMDAVFEYT

163 FTTRVYMDAVFEYTM

164 TTRVYMDAVFEYTMD

165 TRVYMDAVFEYTMDC

166 RVYMDAVFEYTMDCD

169 MDAVFEYTMDCDGSI

171 AVFEYTMDCDGSILG

172 VFEYTMDCDGSILGA

173 FEYTMDCDGSILGAA

174 EYTMDCDGSILGAAV

**Allele: DRB3_0202. Number of high binders 10.**

127 GRKNGSFIIDGKSRK

128 RKNGSFIIDGKSRKE

129 KNGSFIIDGKSRKEC

130 NGSFIIDGKSRKECP

131 GSFIIDGKSRKECPF

91 QDSKNIYQRGTHPFS

92 DSKNIYQRGTHPFSR

93 SKNIYQRGTHPFSRI

94 KNIYQRGTHPFSRIR

95 NIYQRGTHPFSRIRD

**Allele: DRB4_0101. Number of high binders 0.**

**Allele:DRB5_0101. Number of high binders 23.**

93 SKNIYQRGTHPFSRI

94 KNIYQRGTHPFSRIR

95 NIYQRGTHPFSRIRD

96 IYQRGTHPFSRIRDG

115 WKTWGKNLVFSPGRK

116 KTWGKNLVFSPGRKN

117 TWGKNLVFSPGRKNG

118 WGKNLVFSPGRKNGS

119 GKNLVFSPGRKNGSF

120 KNLVFSPGRKNGSFI

128 RKNGSFIIDGKSRKE

129 KNGSFIIDGKSRKEC

130 NGSFIIDGKSRKECP

178 DCDGSILGAAVNGKK

179 CDGSILGAAVNGKKS

180 DGSILGAAVNGKKSA

181 GSILGAAVNGKKSAH

182 SILGAAVNGKKSAHG

183 ILGAAVNGKKSAHGS

326 SDGCWYPMEIRPKKT

327 DGCWYPMEIRPKKTH

328 GCWYPMEIRPKKTHD

329 CWYPMEIRPKKTHDS

**Allele: HLA-DQA10501-DQB10201. Number of high binders 16.**

70 SRADEINAILEENEV

71 RADEINAILEENEVD

72 ADEINAILEENEVDI

73 DEINAILEENEVDIS

144 PFSNRVWNSFQIEEF

145 FSNRVWNSFQIEEFG

146 SNRVWNSFQIEEFGT

147 NRVWNSFQIEEFGTG

160 TGVFTTRVYMDAVFE

161 GVFTTRVYMDAVFEY

162 VFTTRVYMDAVFEYT

163 FTTRVYMDAVFEYTM

164 TTRVYMDAVFEYTMD

165 TRVYMDAVFEYTMDC

166 RVYMDAVFEYTMDCD

207 VNGTWMIHTLETLDY

**Allele: HLA-DQA10501-DQB10301. Number of high binders 4.**

177 MDCDGSILGAAVNGK

178 DCDGSILGAAVNGKK

179 CDGSILGAAVNGKKS

180 DGSILGAAVNGKKSA

**Allele: HLA-DQA10301-DQB10302. Number of high binders 17.**

67 MWRSRADEINAILEE

68 WRSRADEINAILEEN

69 RSRADEINAILEENE

70 SRADEINAILEENEV

71 RADEINAILEENEVD

72 ADEINAILEENEVDI

73 DEINAILEENEVDIS

77 AILEENEVDISVVVQ

78 ILEENEVDISVVVQD

79 LEENEVDISVVVQDS

80 EENEVDISVVVQDSK

81 ENEVDISVVVQDSKN

82 NEVDISVVVQDSKNI

83 EVDISVVVQDSKNIY

144 PFSNRVWNSFQIEEF

145 FSNRVWNSFQIEEFG

146 SNRVWNSFQIEEFGT

**Allele: HLA-DQA10401-DQB10402. Number of high binders 16.**

67 MWRSRADEINAILEE

68 WRSRADEINAILEEN

69 RSRADEINAILEENE

70 SRADEINAILEENEV

71 RADEINAILEENEVD

72 ADEINAILEENEVDI

73 DEINAILEENEVDIS

78 ILEENEVDISVVVQD

79 LEENEVDISVVVQDS

80 EENEVDISVVVQDSK

81 ENEVDISVVVQDSKN

144 PFSNRVWNSFQIEEF

225 EWPLTHTIGTSVEES

226 WPLTHTIGTSVEESD

227 PLTHTIGTSVEESDM

228 LTHTIGTSVEESDMF

**Allele: HLA-DQA10101-DQB10501. Number of high binders 7.**

144 PFSNRVWNSFQIEEF

145 FSNRVWNSFQIEEFG

146 SNRVWNSFQIEEFGT

147 NRVWNSFQIEEFGTG

148 RVWNSFQIEEFGTGV

162 VFTTRVYMDAVFEYT

163 FTTRVYMDAVFEYTM

**Allele: HLA-DQA10102-DQB10602. Number of high binders 0.**

**Allele: HLA-DPA10201-DPB10101. Number of high binders 5.**

146 SNRVWNSFQIEEFGT

147 NRVWNSFQIEEFGTG

148 RVWNSFQIEEFGTGV

149 VWNSFQIEEFGTGVF

150 WNSFQIEEFGTGVFT

**Allele: HLA-DPA10103-DPB10201. Number of high binders 0.**

**Allele: HLA-DPA10103-DPB10401. Number of high binders 0.**

**Allele: HLA-DPA10301-DPB10402. Number of high binders 0.**

**Allele: HLA-DPA10201-DPB10501. Number of high binders 2.**

38 DPVKLASIVKASFEE

39 PVKLASIVKASFEEG

**Allele: HLA-DPA10201-DPB11401. Number of high binders 0.**
