# Supplementary material for: Advancing molecular modeling and reverse vaccinology in broad-spectrum yellow fever virus vaccine development
Source: Sci Rep. 2024 May 12;14:10842. doi: 10.1038/s41598-024-60680-9 (PMC11089047; doi:10.1038/s41598-024-60680-9)
Supplement: Supplementary file 1 — Supplementary Information. [file 41598_2024_60680_MOESM1_ESM.zip › Yellow_Fever_data/2_Prediction of T-cell epitopes/MHC CLASS II/NETMHCII NS2B.docx]

**Proteína NS2B**

**Allele: DRB1_0101. Number of high binders 0.**

**Allele: DRB1_0301. Number of high binders 1.**

42 VSVAGRVDGLELKKL

**Allele: DRB1_0401. Number of high binders 4**

17 LAGLAFQEMENFLGP

18 AGLAFQEMENFLGPV

20 LAFQEMENFLGPVAV

**Allele: DRB1_0405. Number of high binders 0.**

**Allele: DRB1_0701. Number of high binders 8.**

34 VGGILMMLVSVAGRV

35 GGILMMLVSVAGRVD

36 GILMMLVSVAGRVDG

37 ILMMLVSVAGRVDGL

38 LMMLVSVAGRVDGLE

39 MMLVSVAGRVDGLEL

100 TSLALVGAAIHPFAL

101 SLALVGAAIHPFALL

**Allele: DRB1_0802. Number of high binders 0.**

**Allele: DRB1_0901. Number of high binders 13.**

34 VGGILMMLVSVAGRV

35 GGILMMLVSVAGRVD

36 GILMMLVSVAGRVDG

37 ILMMLVSVAGRVDGL

38 LMMLVSVAGRVDGLE

39 MMLVSVAGRVDGLEL

98 VMTSLALVGAAIHPF

99 MTSLALVGAAIHPFA

100 TSLALVGAAIHPFAL

101 SLALVGAAIHPFALL

102 LALVGAAIHPFALLL

103 ALVGAAIHPFALLLV

104 LVGAAIHPFALLLVL

**Allele: DRB1_1101. Number of high binders 0.**

**Allele: DRB1_1201. Number of high binders 0.**

**Allele: DRB1_1302. Number of high binders 0.**

**Allele: DRB1_1501. Number of high binders 0.**

**Allele: DRB3_0101. Number of high binders 0**

**Allele: DRB3_0202. Number of high binders 0.**

**Allele: DRB4_0101. Number of high binders 0**

**Allele: DRB5_0101. Number of high binders 5.**

34 VGGILMMLVSVAGRV

35 GGILMMLVSVAGRVD

36 GILMMLVSVAGRVDG

37 ILMMLVSVAGRVDGL

116 LVLAGWLLHVKGARR

**Allele: HLA-DQA10501-DQB10201. Number of high binders 2.**

15 GVLAGLAFQEMENFL

53 LKKLGEVSWEEEAEI

**Allele: HLA-DQA10501-DQB10301. Number of high binders 2.**

4 VNEALAAAGLVGVLA

100 TSLALVGAAIHPFAL

**Allele: HLA-DQA10301-DQB10302. Number of high binders 3.**

12 GLVGVLAGLAFQEME

14 VGVLAGLAFQEMENF

70 SSARYDVALSEQGEF

**Allele: HLA-DQA10401-DQB10402. Number of high binders 7**

10 AAGLVGVLAGLAFQE

11 AGLVGVLAGLAFQEM

12 GLVGVLAGLAFQEME

13 LVGVLAGLAFQEMEN

14 VGVLAGLAFQEMENF

15 GVLAGLAFQEMENFL

16 VLAGLAFQEMENFLG

**Allele: HLA-DQA10101-DQB10501. Number of high binders 0.**

**Allele: HLA-DQA10102-DQB10602. Number of high binders 4.**

95 DQVVMTSLALVGAAI

96 QVVMTSLALVGAAIH

97 VVMTSLALVGAAIHP

98 VMTSLALVGAAIHPF

**Allele: HLA-DPA10201-DPB10101. Number of high binders 0**

**Allele: HLA-DPA10103-DPB10201. Number of high binders 0**

**Allele: HLA-DPA10103-DPB10401. Number of high binders 0**

**Allele: HLA-DPA10301-DPB10402. Number of high binders 0**

**Allele: HLA-DPA10201-DPB10501. Number of high binders 0.**

**Allele: HLA-DPA10201-DPB11401. Number of high binders 0.**
