# Supplementary material for: Advancing molecular modeling and reverse vaccinology in broad-spectrum yellow fever virus vaccine development
Source: Sci Rep. 2024 May 12;14:10842. doi: 10.1038/s41598-024-60680-9 (PMC11089047; doi:10.1038/s41598-024-60680-9)
Supplement: Supplementary file 1 — Supplementary Information. [file 41598_2024_60680_MOESM1_ESM.zip › Yellow_Fever_data/2_Prediction of T-cell epitopes/MHC CLASS II/NETMHCII NS4A.docx]

**Proteína NS4A**

**Allele: DRB1_0101. Number of high binders 0.**

**Allele: DRB1_0301. Number of high binders 0.**

**Allele: DRB1_0401. Number of high binders 5**

35 EGSRAYRNALSMMPE

36 GSRAYRNALSMMPEA

37 SRAYRNALSMMPEAM

38 RAYRNALSMMPEAMT

39 AYRNALSMMPEAMTT

**Allele: DRB1_0405. Number of high binders 4**

35 EGSRAYRNALSMMPE

36 GSRAYRNALSMMPEA

37 SRAYRNALSMMPEAM

38 RAYRNALSMMPEAMT

**Allele: DRB1_0701. Number of high binders 9**

90 CGYLMFLGGVKPTHI

91 GYLMFLGGVKPTHIS

92 YLMFLGGVKPTHISY

93 LMFLGGVKPTHISYI

94 MFLGGVKPTHISYIM

95 FLGGVKPTHISYIML

96 LGGVKPTHISYIMLI

97 GGVKPTHISYIMLIF

98 GVKPTHISYIMLIFF

**Allele: DRB1_0802. Number of high binders 1.**

74 PKGISRMSMAMGTMA

**Allele: DRB1_0901. Number of high binders 24**

35 EGSRAYRNALSMMPE

36 GSRAYRNALSMMPEA

37 SRAYRNALSMMPEAM

38 RAYRNALSMMPEAMT

39 AYRNALSMMPEAMTT

40 YRNALSMMPEAMTTV

41 RNALSMMPEAMTTVM

64 TSGMVIFFMSPKGIS

65 SGMVIFFMSPKGISR

66 GMVIFFMSPKGISRM

67 MVIFFMSPKGISRMS

74 PKGISRMSMAMGTMA

75 KGISRMSMAMGTMAG

76 GISRMSMAMGTMAGC

77 ISRMSMAMGTMAGCG

78 SRMSMAMGTMAGCGY

79 RMSMAMGTMAGCGYL

80 MSMAMGTMAGCGYLM

93 LMFLGGVKPTHISYI

94 MFLGGVKPTHISYIM

95 FLGGVKPTHISYIML

96 LGGVKPTHISYIMLI

97 GGVKPTHISYIMLIF

98 GVKPTHISYIMLIFF

**Allele: DRB1_1101. Number of high binders 7**

64 TSGMVIFFMSPKGIS

65 SGMVIFFMSPKGISR

66 GMVIFFMSPKGISRM

67 MVIFFMSPKGISRMS

68 VIFFMSPKGISRMSM

69 IFFMSPKGISRMSMA

70 FFMSPKGISRMSMAM

**Allele: DRB1_1201. Number of high binders 0.**

**Allele: DRB1_1302. Number of high binders 0.**

**Allele: DRB1_1501. Number of high binders 6**

63 LTSGMVIFFMSPKGI

64 TSGMVIFFMSPKGIS

65 SGMVIFFMSPKGISR

66 GMVIFFMSPKGISRM

67 MVIFFMSPKGISRMS

68 VIFFMSPKGISRMSM

**Allele: DRB3_0101. Number of high binders 0.**

**Allele: DRB3_0202. Number of high binders 0**

**Allele: DRB4_0101. Number of high binders 5**

71 FMSPKGISRMSMAMG

72 MSPKGISRMSMAMGT

73 SPKGISRMSMAMGTM

74 PKGISRMSMAMGTMA

75 KGISRMSMAMGTMAG

**Allele: DRB5_0101. Number of high binders 13**

65 SGMVIFFMSPKGISR

66 GMVIFFMSPKGISRM

67 MVIFFMSPKGISRMS

68 VIFFMSPKGISRMSM

69 IFFMSPKGISRMSMA

70 FFMSPKGISRMSMAM

86 TMAGCGYLMFLGGVK

87 MAGCGYLMFLGGVKP

88 AGCGYLMFLGGVKPT

89 GCGYLMFLGGVKPTH

90 CGYLMFLGGVKPTHI

91 GYLMFLGGVKPTHIS

92 YLMFLGGVKPTHISY

**Allele: HLA-DQA10501-DQB10201. Number of high binders 7**

1 GAAEVLVVLSELPDF

2 AAEVLVVLSELPDFL

3 AEVLVVLSELPDFLA

4 EVLVVLSELPDFLAK

5 VLVVLSELPDFLAKK

6 LVVLSELPDFLAKKG

7 VVLSELPDFLAKKGG

**Allele: HLA-DQA10501-DQB10301. Number of high binders 0**

**Allele: HLA-DQA10301-DQB10302. Number of high binders 2.**

21 GEAVDTISVFLHSEE

22 EAVDTISVFLHSEEG

**Allele: HLA-DQA10401-DQB10402. Number of high binders 5**

1 GAAEVLVVLSELPDF

2 AAEVLVVLSELPDFL

21 GEAVDTISVFLHSEE

22 EAVDTISVFLHSEEG

23 AVDTISVFLHSEEGS

**Allele: HLA-DQA10101-DQB10501. Number of high binders 0**

**Allele: HLA-DQA10102-DQB10602. Number of high binders 18**

1 GAAEVLVVLSELPDF

36 GSRAYRNALSMMPEA

37 SRAYRNALSMMPEAM

38 RAYRNALSMMPEAMT

39 AYRNALSMMPEAMTT

40 YRNALSMMPEAMTTV

41 RNALSMMPEAMTTVM

59 LAGLLTSGMVIFFMS

60 AGLLTSGMVIFFMSP

61 GLLTSGMVIFFMSPK

62 LLTSGMVIFFMSPKG

72 MSPKGISRMSMAMGT

73 SPKGISRMSMAMGTM

74 PKGISRMSMAMGTMA

75 KGISRMSMAMGTMAG

76 GISRMSMAMGTMAGC

77 ISRMSMAMGTMAGCG

78 SRMSMAMGTMAGCGY

79 RMSMAMGTMAGCGYL

**Allele: HLA-DPA10201-DPB10101. Number of high binders 0.**

**Allele: HLA-DPA10103-DPB10201. Number of high binders 0**

**Allele: HLA-DPA10103-DPB10401. Number of high binders 0.**

**Allele: HLA-DPA10301-DPB10402. Number of high binders 0.**

**Allele: HLA-DPA10201-DPB10501. Number of high binders 0**

**Allele: HLA-DPA10201-DPB11401. Number of high binders 3.**

36 GSRAYRNALSMMPEA

37 SRAYRNALSMMPEAM

38 RAYRNALSMMPEAMT
