# Supplementary material for: Advancing molecular modeling and reverse vaccinology in broad-spectrum yellow fever virus vaccine development
Source: Sci Rep. 2024 May 12;14:10842. doi: 10.1038/s41598-024-60680-9 (PMC11089047; doi:10.1038/s41598-024-60680-9)
Supplement: Supplementary file 1 — Supplementary Information. [file 41598_2024_60680_MOESM1_ESM.zip › Yellow_Fever_data/2_Prediction of T-cell epitopes/MHC CLASS II/NETMHCII NS4B.docx]

**Proteína NS2B**

**Allele: DRB1_0101. Number of high binders 1**

124 LPGIKAQQSKLAQRR

**Allele: DRB1_0301. Number of high binders 11.**

119 HWTLILPGIKAQQSK

120 WTLILPGIKAQQSKL

121 TLILPGIKAQQSKLA

175 LLLALSLASVAMCRT

176 LLALSLASVAMCRTP

177 LALSLASVAMCRTPF

216 WNGPMAVSMTGVMRG

217 NGPMAVSMTGVMRGN

218 GPMAVSMTGVMRGNY

219 PMAVSMTGVMRGNYY

220 MAVSMTGVMRGNYYA

**Allele: DRB1_0401. Number of high binders 7**

40 WTVYVGIVTMLSPML

41 TVYVGIVTMLSPMLH

42 VYVGIVTMLSPMLHH

43 YVGIVTMLSPMLHHW

44 VGIVTMLSPMLHHWI

82 KGIPFMKMNISVIIL

124 LPGIKAQQSKLAQRR

**Allele: DRB1_0405. Number of high binders 0.**

**Allele: DRB1_0701. Number of high binders 10**

81 DKGIPFMKMNISVII

82 KGIPFMKMNISVIIL

83 GIPFMKMNISVIILL

84 IPFMKMNISVIILLV

85 PFMKMNISVIILLVS

95 ILLVSGWNSITVMPL

96 LLVSGWNSITVMPLL

135 AQRRVFHGVAKNPVV

136 QRRVFHGVAKNPVVD

185 AMCRTPFSLAEGIVL

**Allele: DRB1_0802. Number of high binders 5**

118 LHWTLILPGIKAQQS

119 HWTLILPGIKAQQSK

120 WTLILPGIKAQQSKL

121 TLILPGIKAQQSKLA

122 LILPGIKAQQSKLAQ

**Allele: DRB1_0901. Number of high binders 43**

13 LFGKKNLIPSSAAPW

14 FGKKNLIPSSAAPWS

15 GKKNLIPSSAAPWSW

16 KKNLIPSSAAPWSWP

17 KNLIPSSAAPWSWPD

18 NLIPSSAAPWSWPDL

31 DLDLKPGAAWTVYVG

32 LDLKPGAAWTVYVGI

41 TVYVGIVTMLSPMLH

81 DKGIPFMKMNISVII

82 KGIPFMKMNISVIIL

83 GIPFMKMNISVIILL

95 ILLVSGWNSITVMPL

96 LLVSGWNSITVMPLL

97 LVSGWNSITVMPLLC

98 VSGWNSITVMPLLCG

99 SGWNSITVMPLLCGI

100 GWNSITVMPLLCGIG

134 LAQRRVFHGVAKNPV

135 AQRRVFHGVAKNPVV

136 QRRVFHGVAKNPVVD

137 RRVFHGVAKNPVVDG

138 RVFHGVAKNPVVDGN

171 LALYLLLALSLASVA

172 ALYLLLALSLASVAM

173 LYLLLALSLASVAMC

174 YLLLALSLASVAMCR

175 LLLALSLASVAMCRT

176 LLALSLASVAMCRTP

185 AMCRTPFSLAEGIVL

186 MCRTPFSLAEGIVLA

187 CRTPFSLAEGIVLAS

188 RTPFSLAEGIVLASA

189 TPFSLAEGIVLASAA

190 PFSLAEGIVLASAAL

191 FSLAEGIVLASAALG

193 LAEGIVLASAALGPL

194 AEGIVLASAALGPLI

195 EGIVLASAALGPLIE

196 GIVLASAALGPLIEG

197 IVLASAALGPLIEGN

198 VLASAALGPLIEGNT

226 GVMRGNYYAFVGVMY

**Allele: DRB1_1101. Number of high binders 7.**

135 AQRRVFHGVAKNPVV

136 QRRVFHGVAKNPVVD

137 RRVFHGVAKNPVVDG

138 RVFHGVAKNPVVDGN

180 SLASVAMCRTPFSLA

181 LASVAMCRTPFSLAE

182 ASVAMCRTPFSLAEG

**Allele: DRB1_1201. Number of high binders 7**

43 YVGIVTMLSPMLHHW

44 VGIVTMLSPMLHHWI

45 GIVTMLSPMLHHWIK

46 IVTMLSPMLHHWIKV

161 PEMPALYEKKLALYL

162 EMPALYEKKLALYLL

163 MPALYEKKLALYLLL

**Allele: DRB1_1302. Number of high binders 12.**

81 DKGIPFMKMNISVII

82 KGIPFMKMNISVIIL

83 GIPFMKMNISVIILL

84 IPFMKMNISVIILLV

85 PFMKMNISVIILLVS

208 IEGNTSLLWNGPMAV

209 EGNTSLLWNGPMAVS

210 GNTSLLWNGPMAVSM

211 NTSLLWNGPMAVSMT

212 TSLLWNGPMAVSMTG

213 SLLWNGPMAVSMTGV

214 LLWNGPMAVSMTGVM

**Allele: DRB1_1501. Number of high binders 7**

41 TVYVGIVTMLSPMLH

42 VYVGIVTMLSPMLHH

43 YVGIVTMLSPMLHHW

44 VGIVTMLSPMLHHWI

45 GIVTMLSPMLHHWIK

46 IVTMLSPMLHHWIKV

93 VIILLVSGWNSITVM

**Allele: DRB3_0101. Number of high binders 6**

83 GIPFMKMNISVIILL

84 IPFMKMNISVIILLV

185 AMCRTPFSLAEGIVL

186 MCRTPFSLAEGIVLA

187 CRTPFSLAEGIVLAS

188 RTPFSLAEGIVLASA

**Allele: DRB3_0202. Number of high binders 9.**

14 FGKKNLIPSSAAPWS

15 GKKNLIPSSAAPWSW

16 KKNLIPSSAAPWSWP

17 KNLIPSSAAPWSWPD

209 EGNTSLLWNGPMAVS

210 GNTSLLWNGPMAVSM

211 NTSLLWNGPMAVSMT

212 TSLLWNGPMAVSMTG

213 SLLWNGPMAVSMTGV

**Allele: DRB4_0101. Number of high binders 6**

79 FMDKGIPFMKMNISV

80 MDKGIPFMKMNISVI

81 DKGIPFMKMNISVII

82 KGIPFMKMNISVIIL

83 GIPFMKMNISVIILL

84 IPFMKMNISVIILLV

**Allele: DRB5_0101. Number of high binders 23**

41 TVYVGIVTMLSPMLH

42 VYVGIVTMLSPMLHH

43 YVGIVTMLSPMLHHW

44 VGIVTMLSPMLHHWI

45 GIVTMLSPMLHHWIK

46 IVTMLSPMLHHWIKV

47 VTMLSPMLHHWIKVE

75 SVLSFMDKGIPFMKM

76 VLSFMDKGIPFMKMN

77 LSFMDKGIPFMKMNI

78 SFMDKGIPFMKMNIS

79 FMDKGIPFMKMNISV

116 AMLHWTLILPGIKAQ

117 MLHWTLILPGIKAQQ

118 LHWTLILPGIKAQQS

119 HWTLILPGIKAQQSK

124 LPGIKAQQSKLAQRR

125 PGIKAQQSKLAQRRV

126 GIKAQQSKLAQRRVF

175 LLLALSLASVAMCRT

176 LLALSLASVAMCRTP

177 LALSLASVAMCRTPF

236 VGVMYNLWKMKTGRR

**Allele: HLA-DQA10501-DQB10201. Number of high binders 0**

**Allele: HLA-DQA10501-DQB10301. Number of high binders 17**

29 WPDLDLKPGAAWTVY

30 PDLDLKPGAAWTVYV

31 DLDLKPGAAWTVYVG

32 LDLKPGAAWTVYVGI

33 DLKPGAAWTVYVGIV

34 LKPGAAWTVYVGIVT

65 LSLSGIAQSASVLSF

66 SLSGIAQSASVLSFM

67 LSGIAQSASVLSFMD

68 SGIAQSASVLSFMDK

188 RTPFSLAEGIVLASA

189 TPFSLAEGIVLASAA

190 PFSLAEGIVLASAAL

191 FSLAEGIVLASAALG

192 SLAEGIVLASAALGP

193 LAEGIVLASAALGPL

194 AEGIVLASAALGPLI

**Allele: HLA-DQA10301-DQB10302. Number of high binders 8.**

145 KNPVVDGNPTVDIEE

146 NPVVDGNPTVDIEEA

147 PVVDGNPTVDIEEAP

148 VVDGNPTVDIEEAPE

149 VDGNPTVDIEEAPEM

150 DGNPTVDIEEAPEMP

153 PTVDIEEAPEMPALY

154 TVDIEEAPEMPALYE

**Allele: HLA-DQA10401-DQB10402. Number of high binders 0**

**Allele: HLA-DQA10101-DQB10501. Number of high binders 0**

**Allele: HLA-DQA10102-DQB10602. Number of high binders 6**

188 RTPFSLAEGIVLASA

189 TPFSLAEGIVLASAA

190 PFSLAEGIVLASAAL

191 FSLAEGIVLASAALG

192 SLAEGIVLASAALGP

193 LAEGIVLASAALGPL

**Allele: HLA-DPA10201-DPB10101. Number of high binders 0**

**Allele: HLA-DPA10103-DPB10201. Number of high binders 0.**

**Allele: HLA-DPA10103-DPB10401. Number of high binders 6**

226 GVMRGNYYAFVGVMY

229 RGNYYAFVGVMYNLW

230 GNYYAFVGVMYNLWK

231 NYYAFVGVMYNLWKM

232 YYAFVGVMYNLWKMK

233 YAFVGVMYNLWKMKT

**Allele: HLA-DPA10301-DPB10402. Number of high binders 0**

**Allele: HLA-DPA10201-DPB10501. Number of high binders 6.**

163 MPALYEKKLALYLLL

164 PALYEKKLALYLLLA

165 ALYEKKLALYLLLAL

166 LYEKKLALYLLLALS

167 YEKKLALYLLLALSL

168 EKKLALYLLLALSLA

**Allele: HLA-DPA10201-DPB11401. Number of high binders 5**

164 PALYEKKLALYLLLA

165 ALYEKKLALYLLLAL

166 LYEKKLALYLLLALS

167 YEKKLALYLLLALSL

168 EKKLALYLLLALSLA
