# Supplementary material for: Advancing molecular modeling and reverse vaccinology in broad-spectrum yellow fever virus vaccine development
Source: Sci Rep. 2024 May 12;14:10842. doi: 10.1038/s41598-024-60680-9 (PMC11089047; doi:10.1038/s41598-024-60680-9)
Supplement: Supplementary file 1 — Supplementary Information. [file 41598_2024_60680_MOESM1_ESM.zip › Yellow_Fever_data/2_Prediction of T-cell epitopes/MHC CLASS II/NTCMHCII NS2A.docx]

**Proteína NS2A**

**Allele: DRB1_0101. Number of high binders 5.**

119 MGGLWKYLNAVSLCI

120 GGLWKYLNAVSLCIL

121 GLWKYLNAVSLCILT

122 LWKYLNAVSLCILTI

123 WKYLNAVSLCILTIN

**Allele: DRB1_0301. Number of high binders 20.**

9 GLVSMMIAMEVVLRK

10 LVSMMIAMEVVLRKR

11 VSMMIAMEVVLRKRQ

12 SMMIAMEVVLRKRQG

13 MMIAMEVVLRKRQGP

14 MIAMEVVLRKRQGPK

15 IAMEVVLRKRQGPKQ

89 GFGLRTLWSPRERLV

90 FGLRTLWSPRERLVL

91 GLRTLWSPRERLVLT

2 LRTLWSPRERLVLTL

129 VSLCILTINAVASRK

130 SLCILTINAVASRKA

131 LCILTINAVASRKAS

32 CILTINAVASRKASN

153 ALLTPVTMAEVRLAT

154 LLTPVTMAEVRLATM

155 LTPVTMAEVRLATML

156 TPVTMAEVRLATMLF

157 PVTMAEVRLATMLFC

**Allele: DRB1_0401. Number of high binders 5.**

119 MGGLWKYLNAVSLCI

120 GGLWKYLNAVSLCIL

121 GLWKYLNAVSLCILT

122 LWKYLNAVSLCILTI

123 WKYLNAVSLCILTIN

**Allele: DRB1_0405. Number of high binders 9.**

67 NGGDAMYMALIAAFS

68 GGDAMYMALIAAFSI

69 GDAMYMALIAAFSIR

70 DAMYMALIAAFSIRP

71 AMYMALIAAFSIRPG

118 MMGGLWKYLNAVSLC

119 MGGLWKYLNAVSLCI

120 GGLWKYLNAVSLCIL

121 GLWKYLNAVSLCILT

**Allele: DRB1_0701. Number of high binders 23**

46 VTILDLLKLTVAVGL

47 TILDLLKLTVAVGLH

48 ILDLLKLTVAVGLHF

49 LDLLKLTVAVGLHFH

50 DLLKLTVAVGLHFHE

51 LLKLTVAVGLHFHEM

52 LKLTVAVGLHFHEMN

74 MALIAAFSIRPGLLI

75 ALIAAFSIRPGLLIG

76 LIAAFSIRPGLLIGF

77 IAAFSIRPGLLIGFG

78 AAFSIRPGLLIGFGL

119 MGGLWKYLNAVSLCI

120 GGLWKYLNAVSLCIL

183 SKDTSMQKTIPLVAL

184 KDTSMQKTIPLVALT

185 DTSMQKTIPLVALTL

186 TSMQKTIPLVALTLT

187 SMQKTIPLVALTLTS

191 TIPLVALTLTSYLGL

206 TQPFLGLCAFMATRI

207 QPFLGLCAFMATRIF

209 FLGLCAFMATRIFGR

**Allele: DRB1_0802. Number of high binders 6**

71 AMYMALIAAFSIRPG

72 MYMALIAAFSIRPGL

73 YMALIAAFSIRPGLL

74 MALIAAFSIRPGLLI

75 ALIAAFSIRPGLLIG

132 CILTINAVASRKASN

**Allele: DRB1_0901. Number of high binders 24**

66 NNGGDAMYMALIAAF

67 NGGDAMYMALIAAFS

68 GGDAMYMALIAAFSI

69 GDAMYMALIAAFSIR

70 DAMYMALIAAFSIRP

71 AMYMALIAAFSIRPG

72 MYMALIAAFSIRPGL

74 MALIAAFSIRPGLLI

75 ALIAAFSIRPGLLIG

76 LIAAFSIRPGLLIGF

77 IAAFSIRPGLLIGFG

78 AAFSIRPGLLIGFGL

79 AFSIRPGLLIGFGLR

96 WSPRERLVLTLGAAM

97 SPRERLVLTLGAAMV

98 PRERLVLTLGAAMVE

99 RERLVLTLGAAMVEI

100 ERLVLTLGAAMVEIA

101 RLVLTLGAAMVEIAL

102 LVLTLGAAMVEIALG

117 GMMGGLWKYLNAVSL

118 MMGGLWKYLNAVSLC

119 MGGLWKYLNAVSLCI

120 GGLWKYLNAVSLCIL

**Allele: DRB1_1101. Number of high binders 8.**

14 MIAMEVVLRKRQGPK

15 IAMEVVLRKRQGPKQ

16 AMEVVLRKRQGPKQI

115 LGGMMGGLWKYLNAV

116 GGMMGGLWKYLNAVS

117 GMMGGLWKYLNAVSL

209 FLGLCAFMATRIFGR

210 LGLCAFMATRIFGRR

**Allele: DRB1_1201. Number of high binders 0.**

**Allele: DRB1_1302. Number of high binders 0.**

**Allele: DRB1_1501. Number of high binders 0.**

**Allele: DRB3_0101. Number of high binders 0.**

**Allele: DRB3_0202. Number of high binders 9.**

119 MGGLWKYLNAVSLCI

120 GGLWKYLNAVSLCIL

121 GLWKYLNAVSLCILT

122 LWKYLNAVSLCILTI

128 AVSLCILTINAVASR

129 VSLCILTINAVASRK

130 SLCILTINAVASRKA

131 LCILTINAVASRKAS

132 CILTINAVASRKASN

**Allele: DRB4_0101. Number of high binders 0.**

**Allele: DRB5_0101. Number of high binders 10.**

89 GFGLRTLWSPRERLV

90 FGLRTLWSPRERLVL

129 VSLCILTINAVASRK

130 SLCILTINAVASRKA

131 LCILTINAVASRKAS

132 CILTINAVASRKASN

133 ILTINAVASRKASNV

134 LTINAVASRKASNVI

209 FLGLCAFMATRIFGR

210 LGLCAFMATRIFGRR

**Allele: HLA-DQA10501-DQB10201. Number of high binders 0.**

**Allele: HLA-DQA10501-DQB10301. Number of high binders 0.**

**Allele: HLA-DQA10301-DQB10302. Number of high binders 0.**

**Allele: HLA-DQA10401-DQB10402. Number of high binders 4.**

99 RERLVLTLGAAMVEI

100 ERLVLTLGAAMVEIA

101 RLVLTLGAAMVEIAL

102 LVLTLGAAMVEIALG

**Allele: HLA-DQA10101-DQB10501. Number of high binders 0.**

**Allele: HLA-DQA10102-DQB10602. Number of high binders 13.**

27 PKQILVGGMVLLGAM

28 KQILVGGMVLLGAML

29 QILVGGMVLLGAMLV

30 ILVGGMVLLGAMLVG

62 FHEMNNGGDAMYMAL

63 HEMNNGGDAMYMALI

64 EMNNGGDAMYMALIA

65 MNNGGDAMYMALIAA

153 ALLTPVTMAEVRLAT

154 LLTPVTMAEVRLATM

155 LTPVTMAEVRLATML

156 TPVTMAEVRLATMLF

157 PVTMAEVRLATMLFC

**Allele: HLA-DPA10201-DPB10101. Number of high binders 0.**

**Allele: HLA-DPA10103-DPB10201. Number of high binders 0.**

**Allele:HLA-DPA10103-DPB10401. Number of high binders 2.**

93 PLVALTLTSYLGLTQ

194 LVALTLTSYLGLTQP

**Allele: HLA-DPA10301-DPB10402. Number of high binders 5.**

42 LVGQVTILDLLKLTV

43 VGQVTILDLLKLTVA

44 GQVTILDLLKLTVAV

194 LVALTLTSYLGLTQP

195 VALTLTSYLGLTQPF

**Allele: HLA-DPA10201-DPB10501. Number of high binders 0.**

**Allele: HLA-DPA10201-DPB11401. Number of high binders 13.**

120 GGLWKYLNAVSLCIL

121 GLWKYLNAVSLCILT

122 LWKYLNAVSLCILTI

123 WKYLNAVSLCILTIN

137 NAVASRKASNVILPL

138 AVASRKASNVILPLM

139 VASRKASNVILPLMA

140 ASRKASNVILPLMAL

141 SRKASNVILPLMALL

142 RKASNVILPLMALLT

185 DTSMQKTIPLVALTL

186 TSMQKTIPLVALTLT

187 SMQKTIPLVALTLTS
