# Supplementary figures and images for: Advancing molecular modeling and reverse vaccinology in broad-spectrum yellow fever virus vaccine development (part 2 of 2)
Source: Sci Rep. 2024 May 12;14:10842. doi: 10.1038/s41598-024-60680-9 (PMC11089047; doi:10.1038/s41598-024-60680-9)

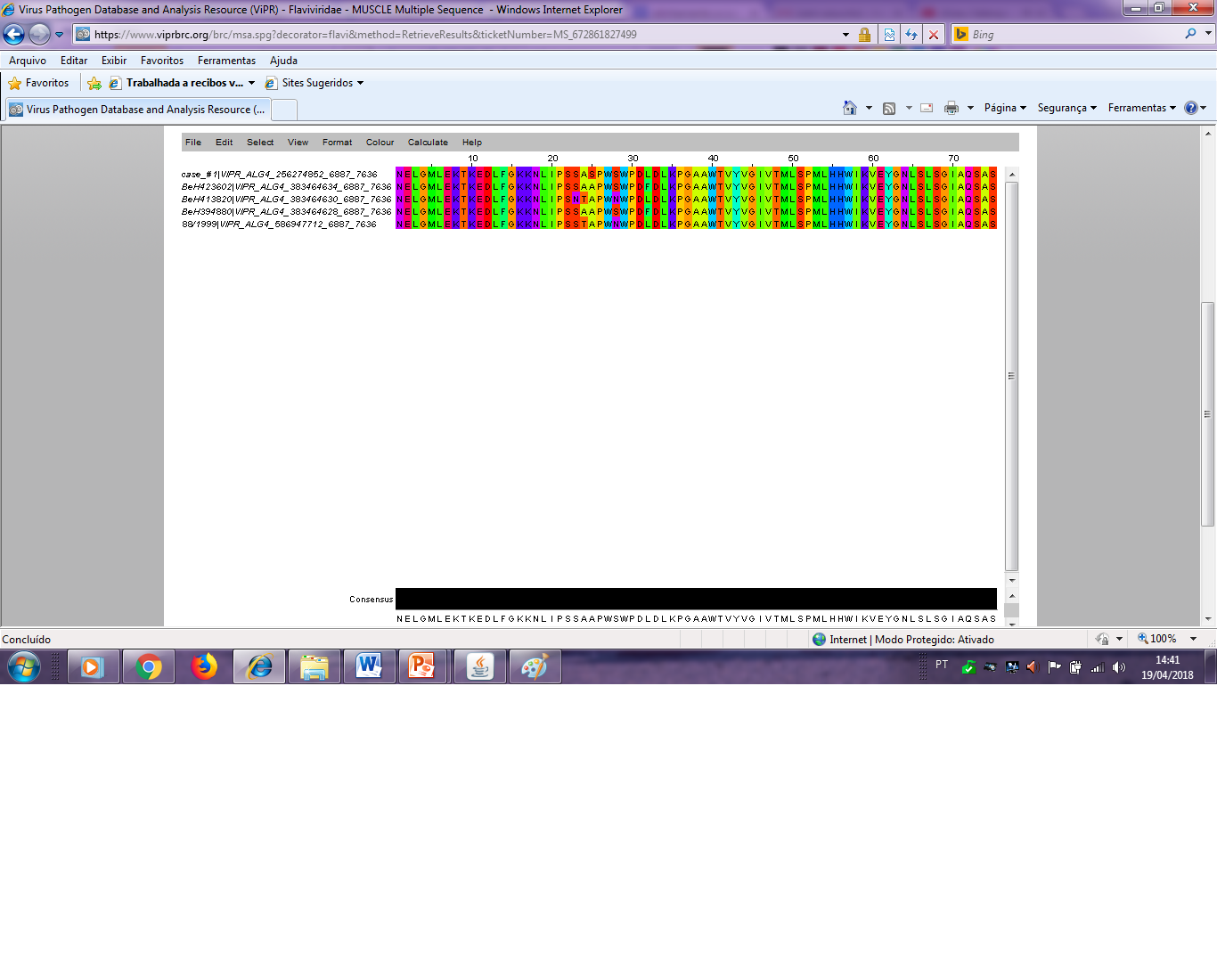

Supplement: Supplementary file 1 — Supplementary Information. [file 41598_2024_60680_MOESM1_ESM.zip › Yellow_Fever_data/1_Acquisition_proteins/Prints VIPR/ns4b/passo 3 ns4b consenso.png]

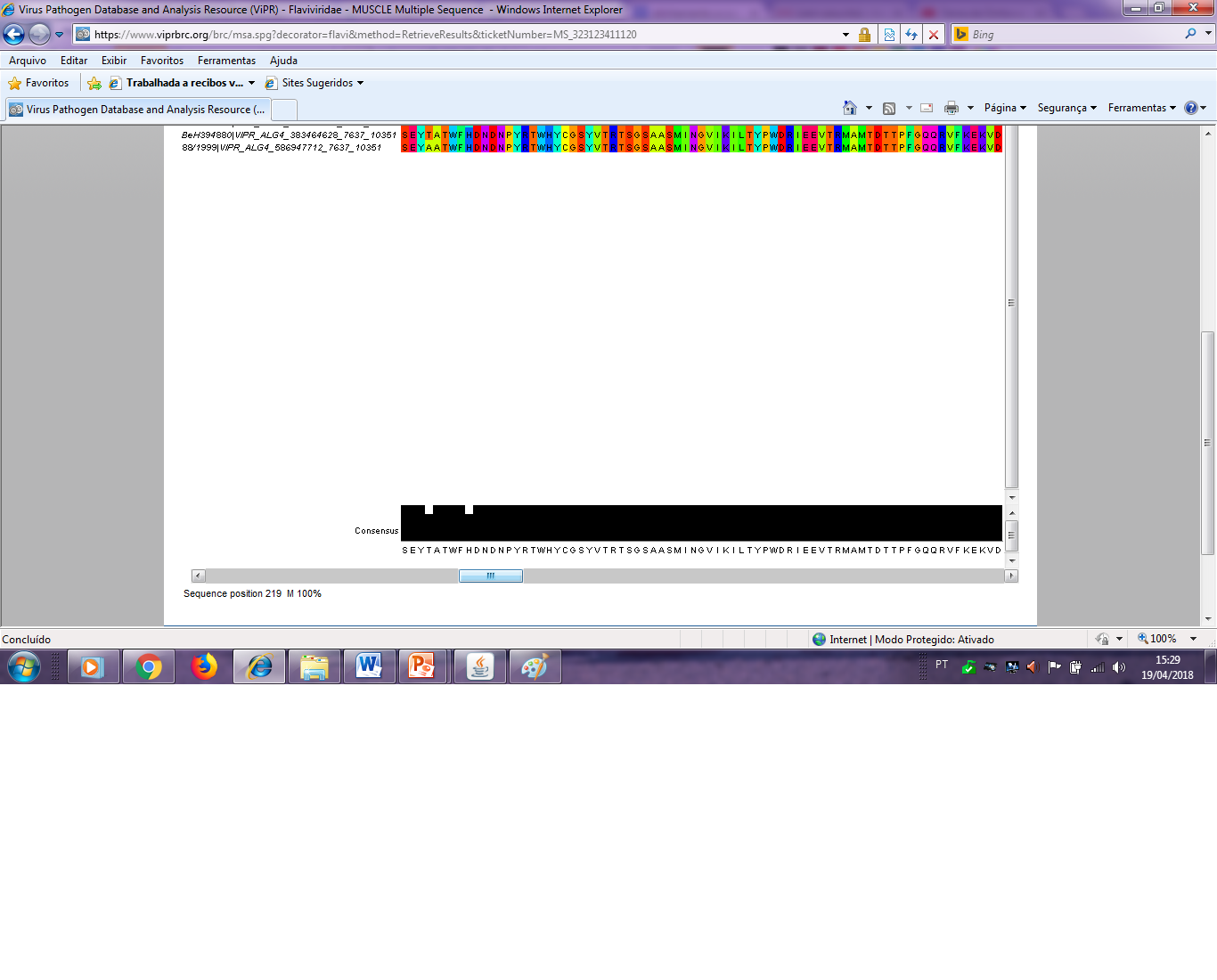

Supplement: Supplementary file 1 — Supplementary Information. [file 41598_2024_60680_MOESM1_ESM.zip › Yellow_Fever_data/1_Acquisition_proteins/Prints VIPR/ns5/passo 3.5 ns5 consenso.png]

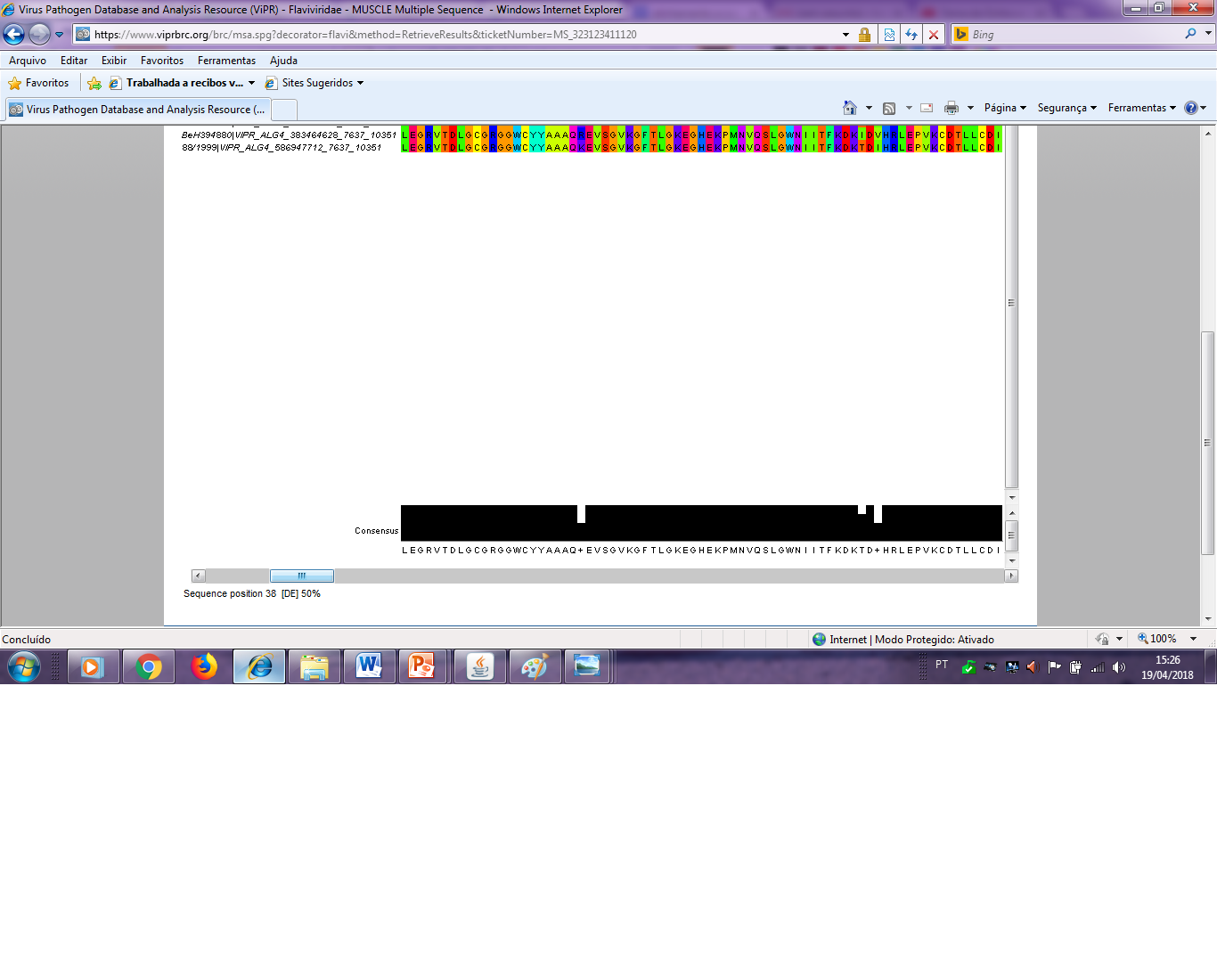

Supplement: Supplementary file 1 — Supplementary Information. [file 41598_2024_60680_MOESM1_ESM.zip › Yellow_Fever_data/1_Acquisition_proteins/Prints VIPR/ns5/passo 3.2 ns5 consenso.png]

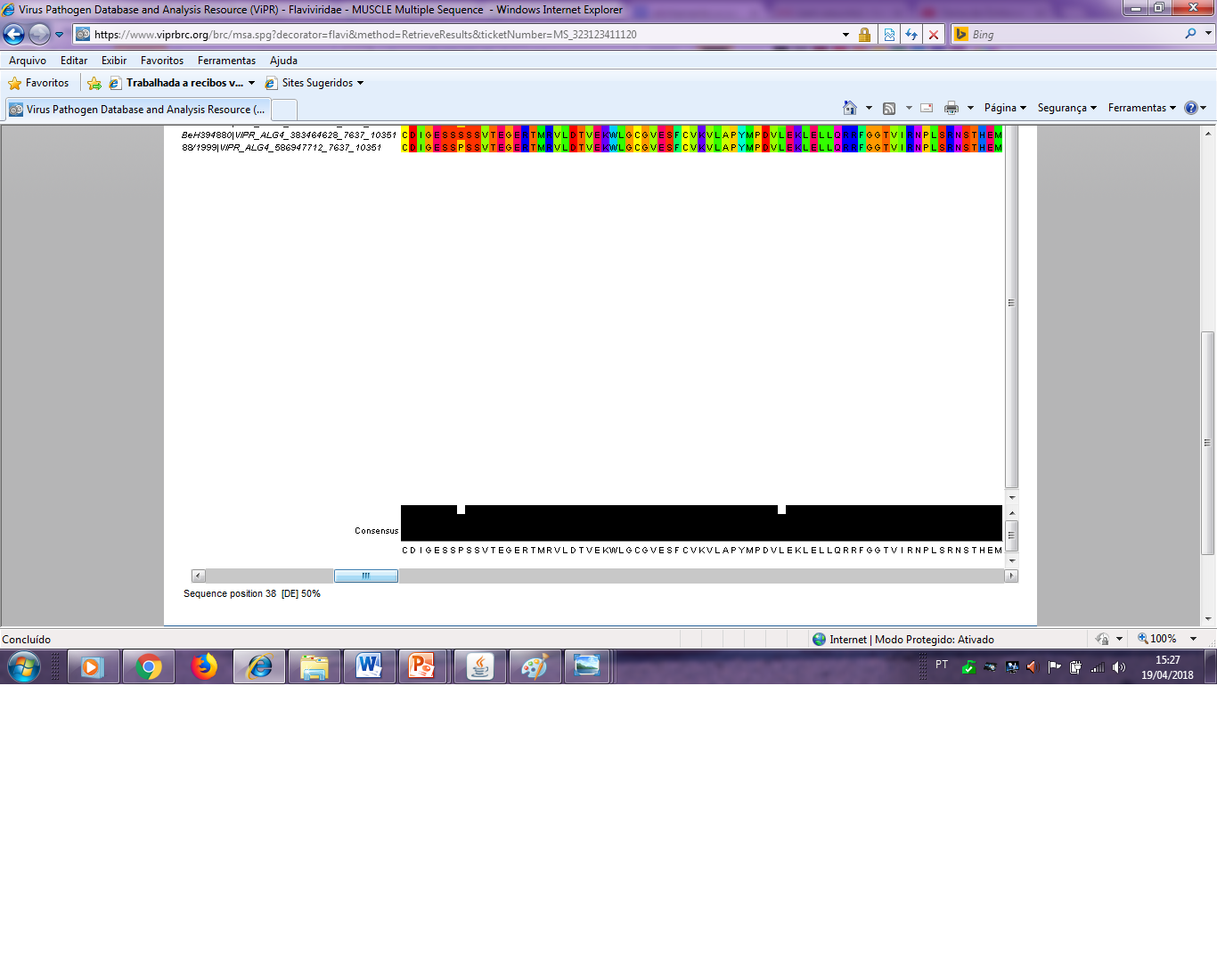

Supplement: Supplementary file 1 — Supplementary Information. [file 41598_2024_60680_MOESM1_ESM.zip › Yellow_Fever_data/1_Acquisition_proteins/Prints VIPR/ns5/passo 3.3 ns5 consenso.png]

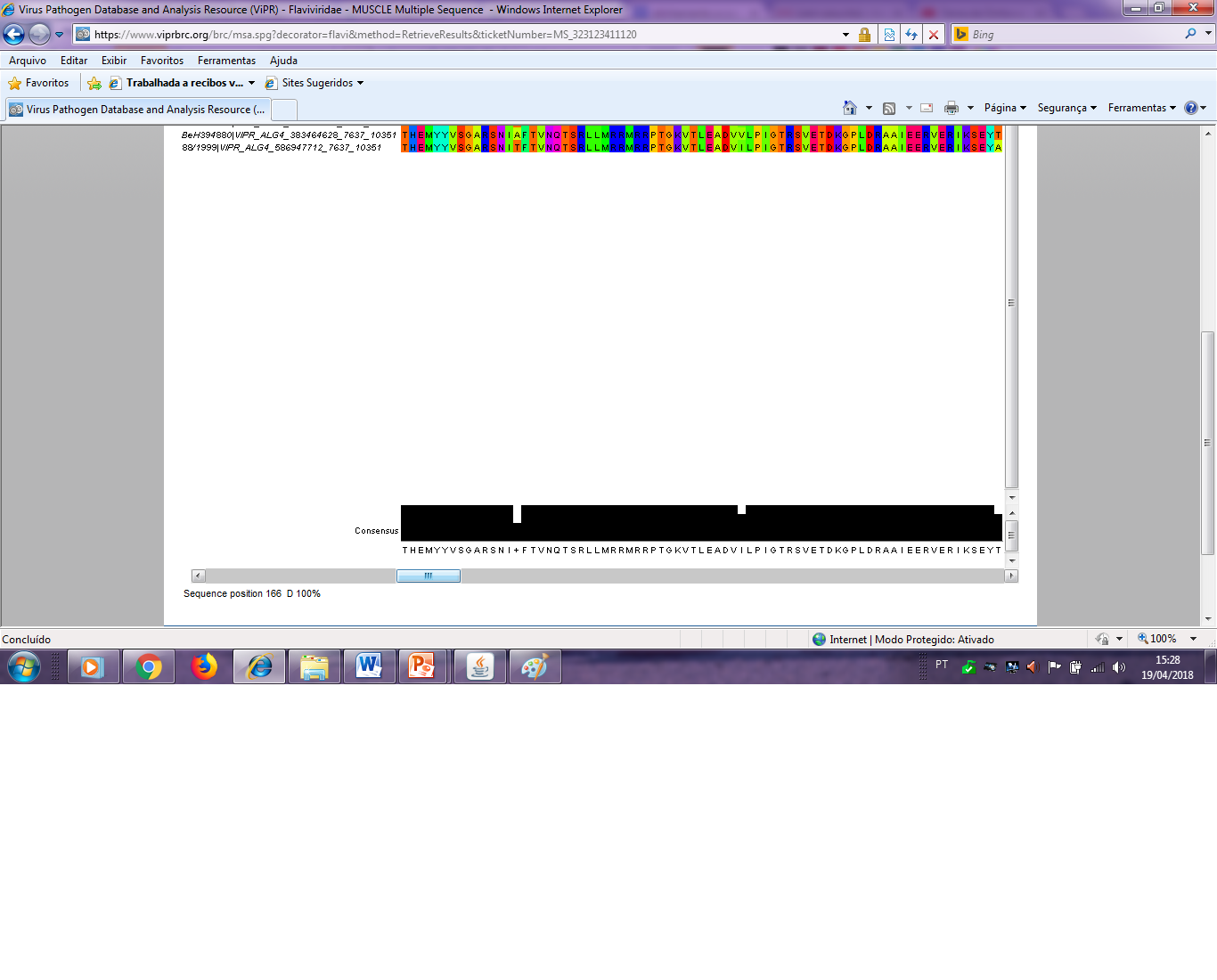

Supplement: Supplementary file 1 — Supplementary Information. [file 41598_2024_60680_MOESM1_ESM.zip › Yellow_Fever_data/1_Acquisition_proteins/Prints VIPR/ns5/passo 3.4 ns5 consenso.png]

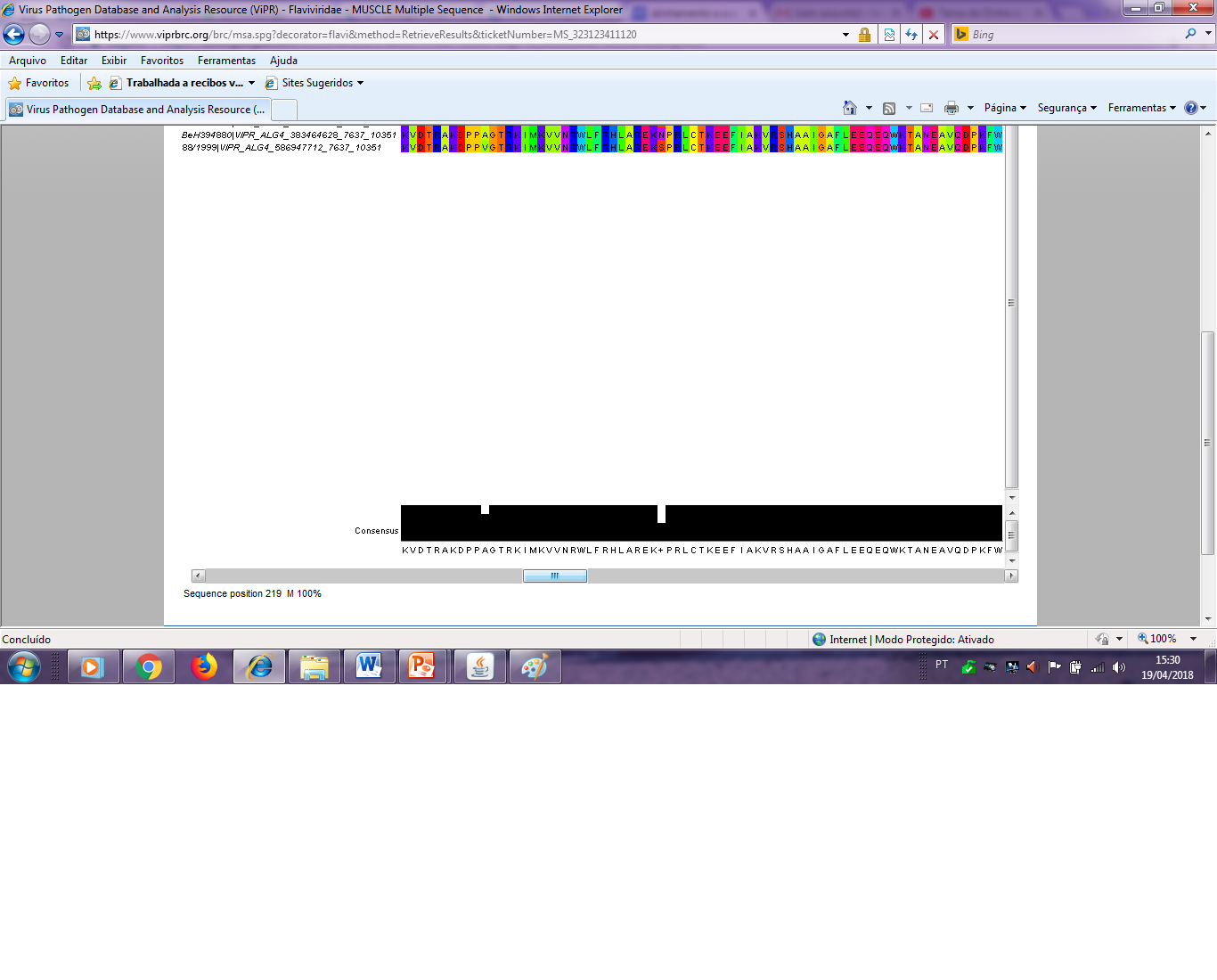

Supplement: Supplementary file 1 — Supplementary Information. [file 41598_2024_60680_MOESM1_ESM.zip › Yellow_Fever_data/1_Acquisition_proteins/Prints VIPR/ns5/passo 3.6 ns5 consenso.png]

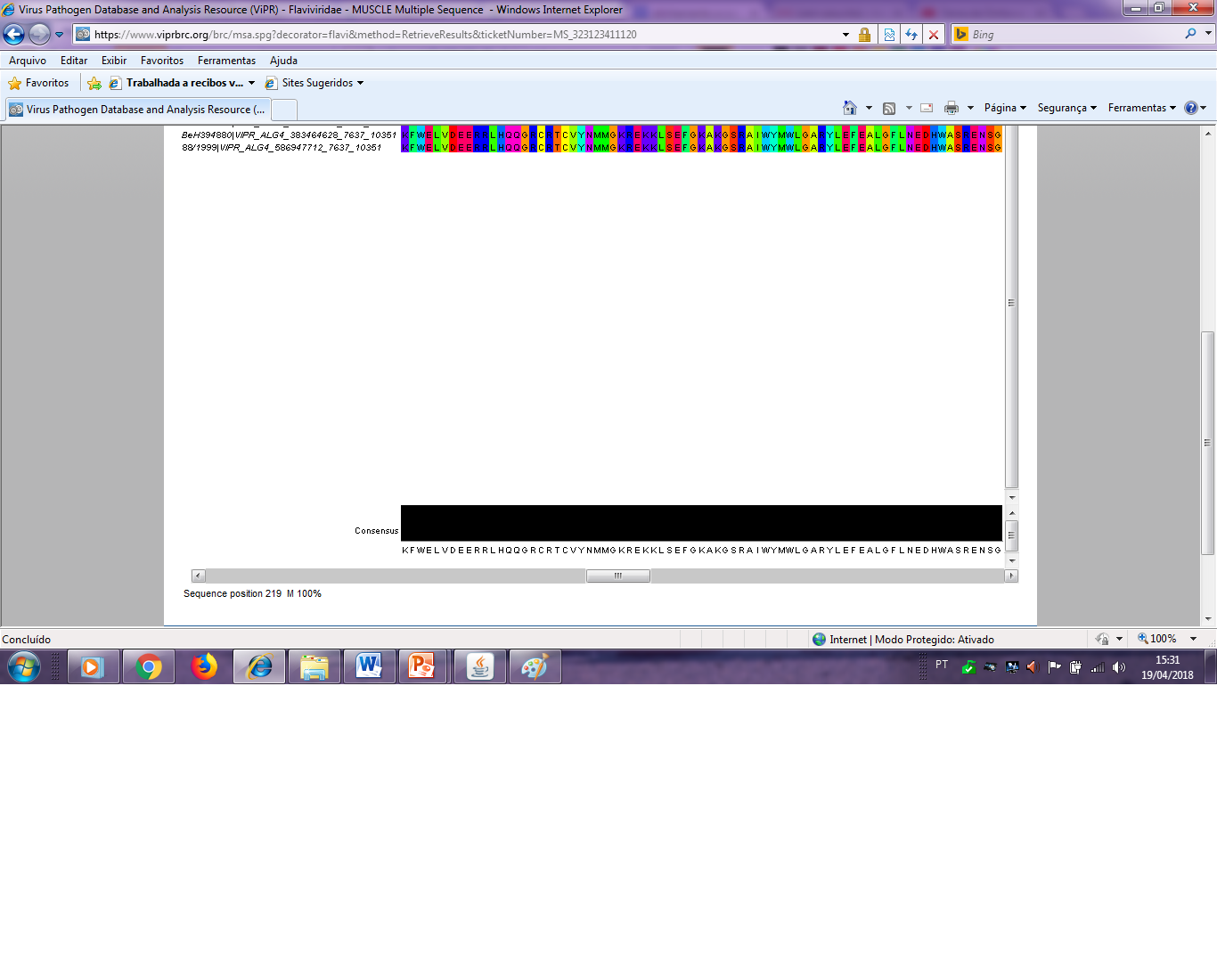

Supplement: Supplementary file 1 — Supplementary Information. [file 41598_2024_60680_MOESM1_ESM.zip › Yellow_Fever_data/1_Acquisition_proteins/Prints VIPR/ns5/passo 3.7 ns5 consenso.png]

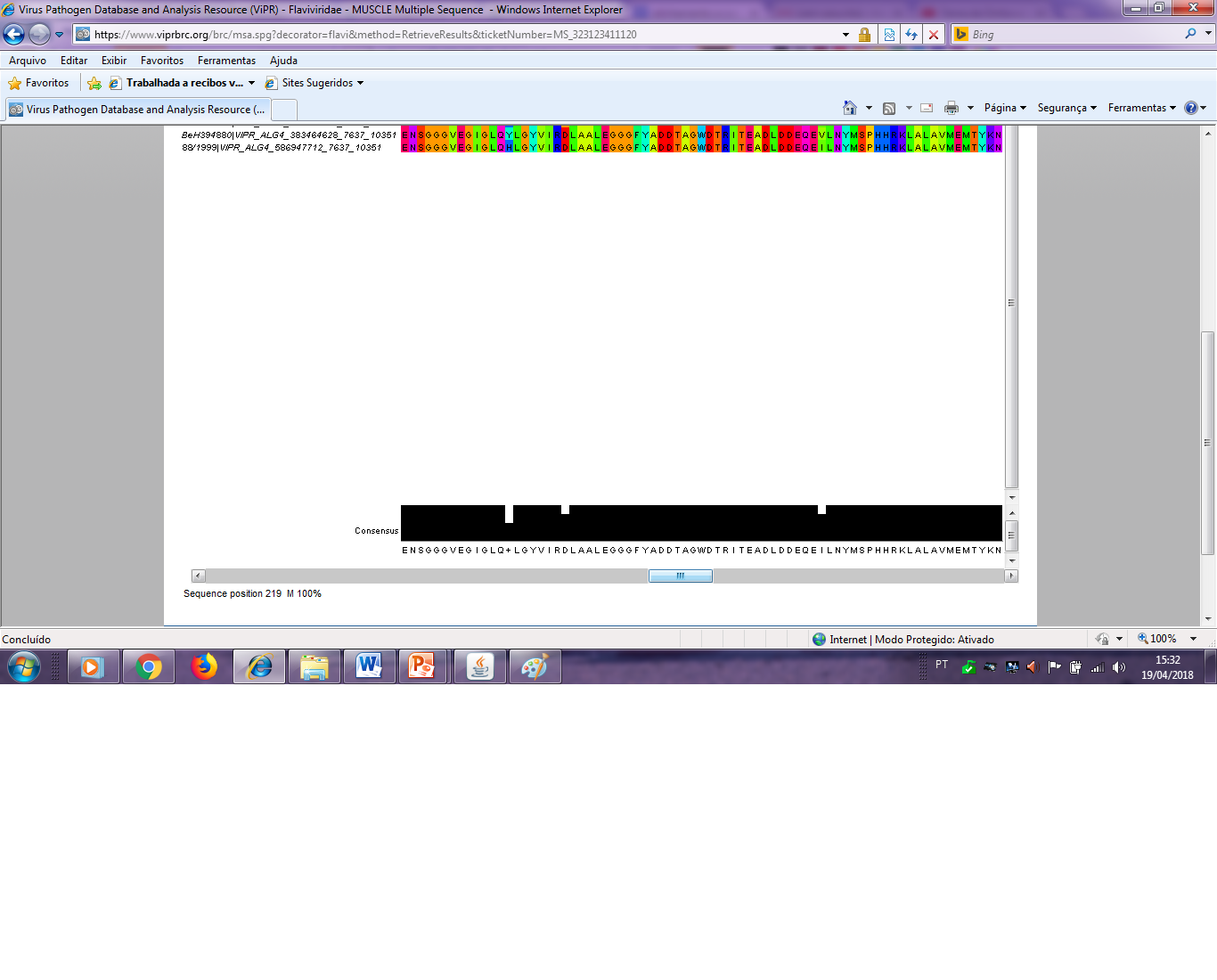

Supplement: Supplementary file 1 — Supplementary Information. [file 41598_2024_60680_MOESM1_ESM.zip › Yellow_Fever_data/1_Acquisition_proteins/Prints VIPR/ns5/passo 3.8 ns5 consenso.png]

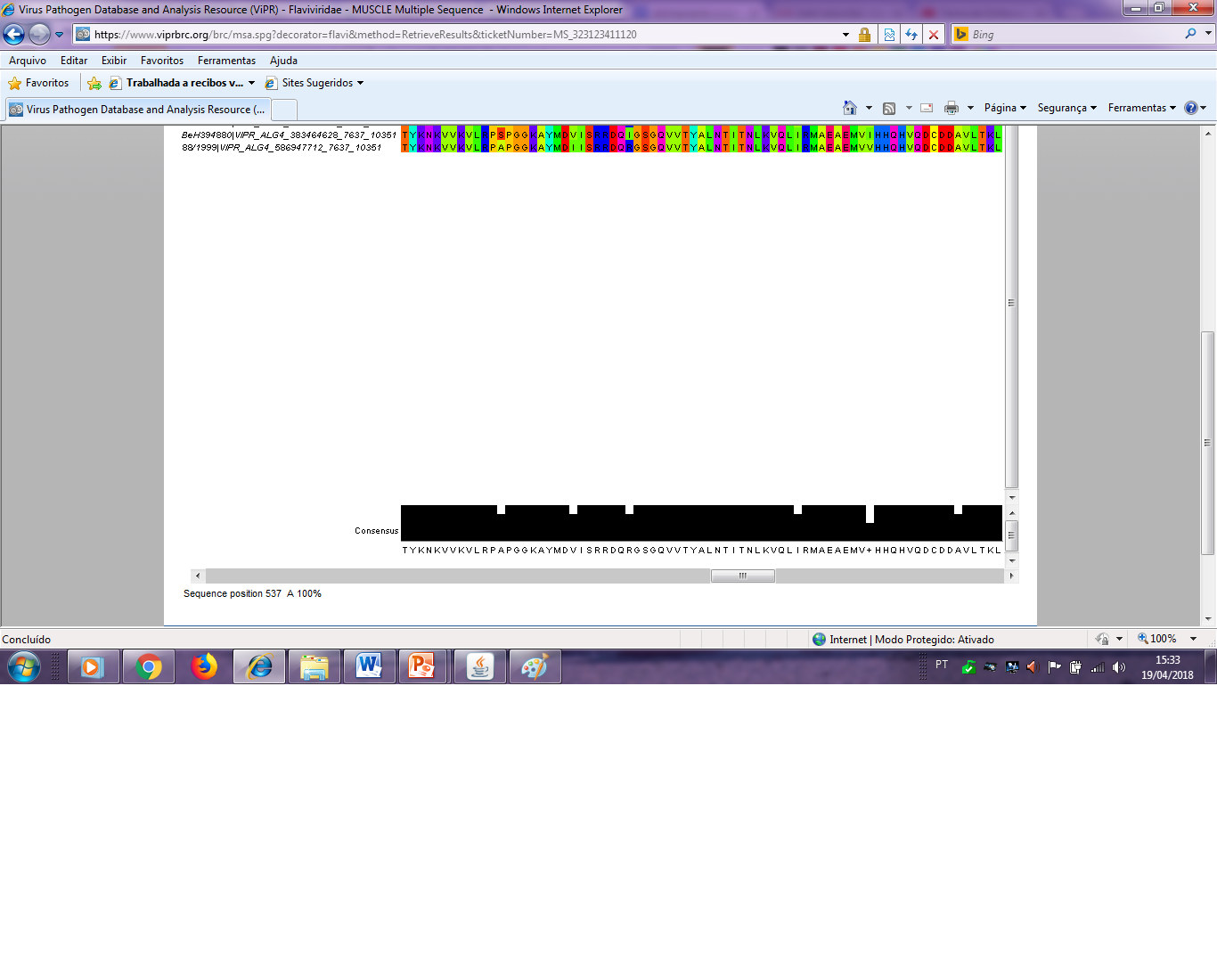

Supplement: Supplementary file 1 — Supplementary Information. [file 41598_2024_60680_MOESM1_ESM.zip › Yellow_Fever_data/1_Acquisition_proteins/Prints VIPR/ns5/passo 3.9 ns5 consenso.png]

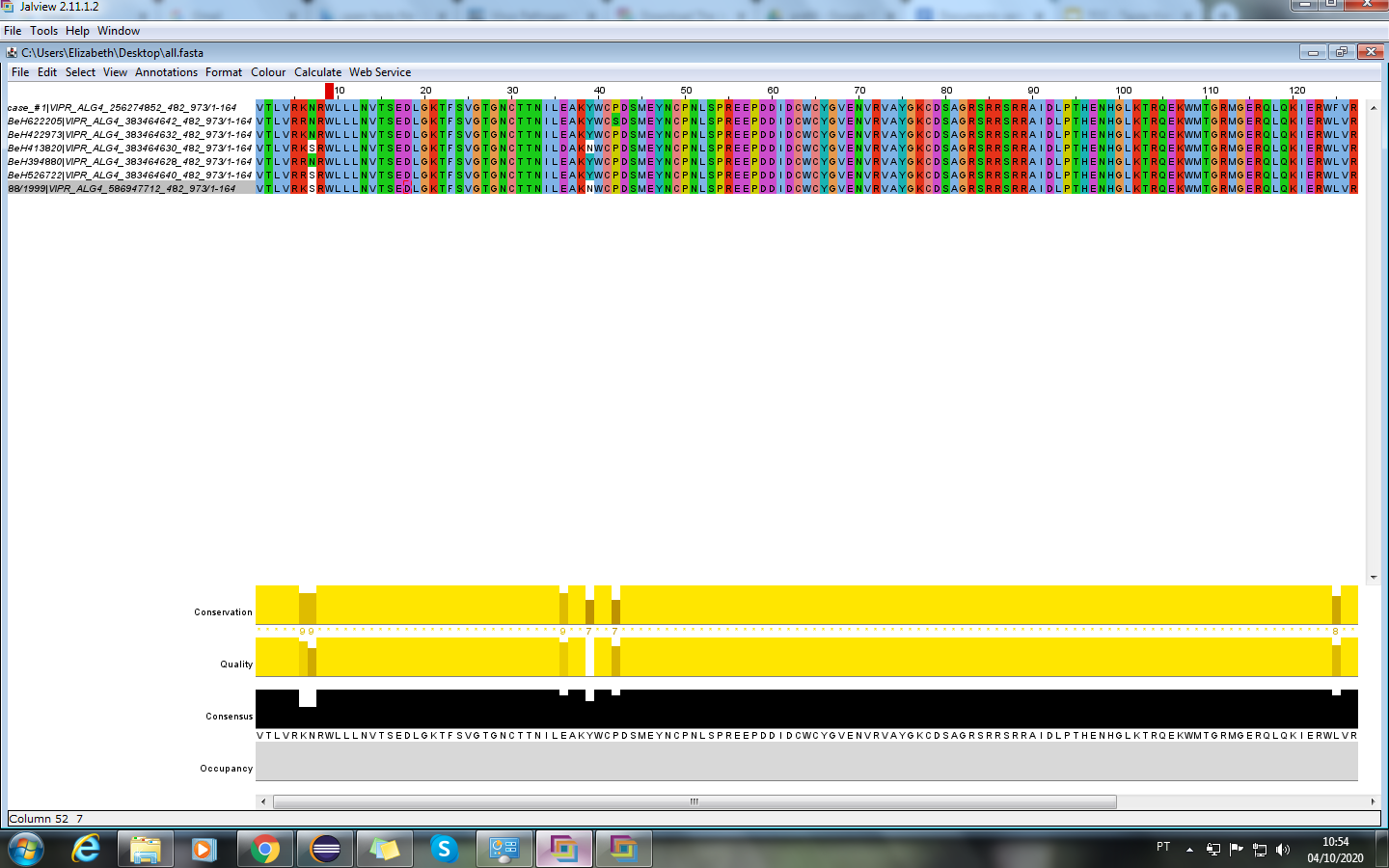


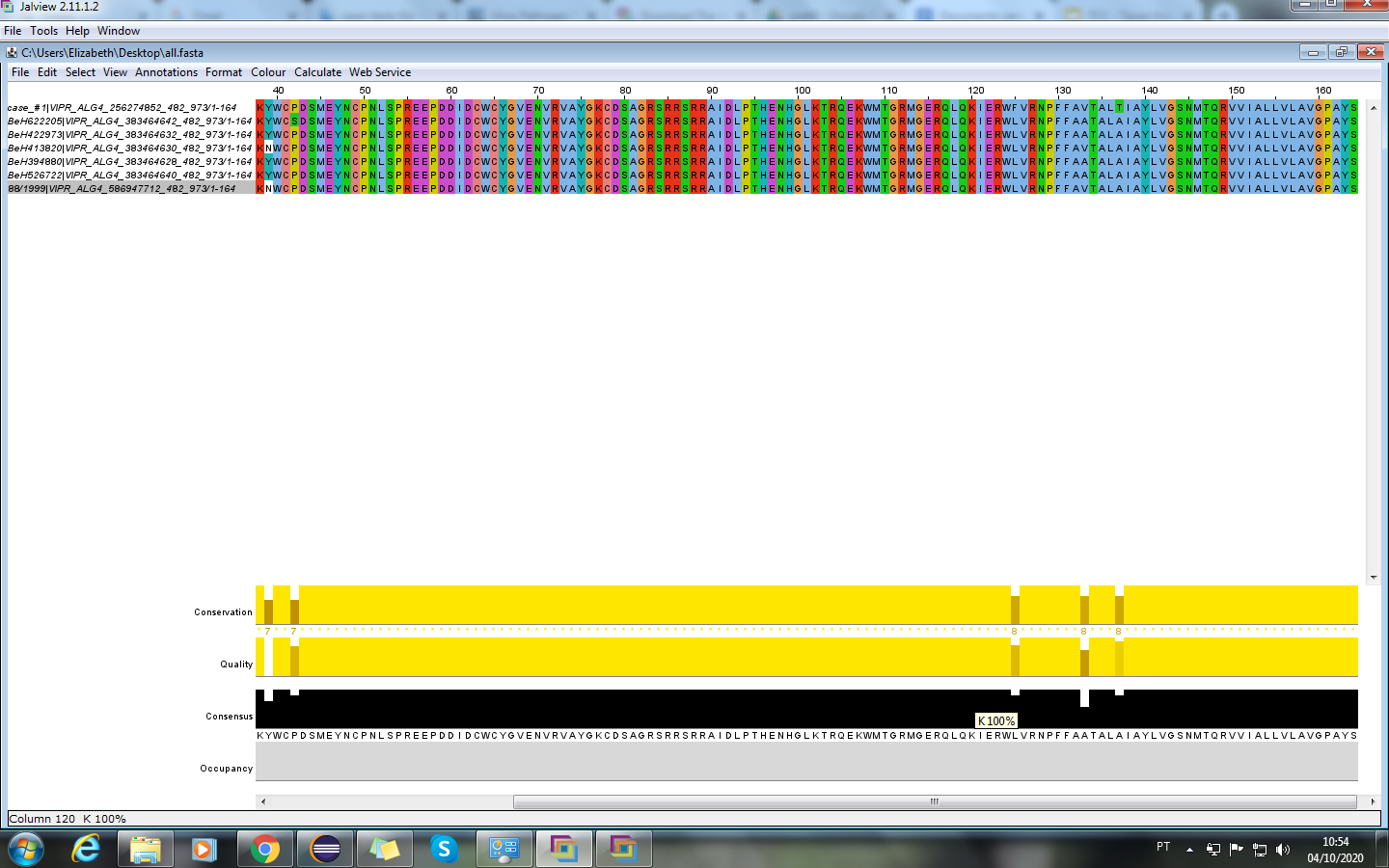

Supplement: Supplementary file 1 — Supplementary Information. [file 41598_2024_60680_MOESM1_ESM.zip › Yellow_Fever_data/1_Acquisition_proteins/Prints VIPR/preM/Documento sem título.docx]

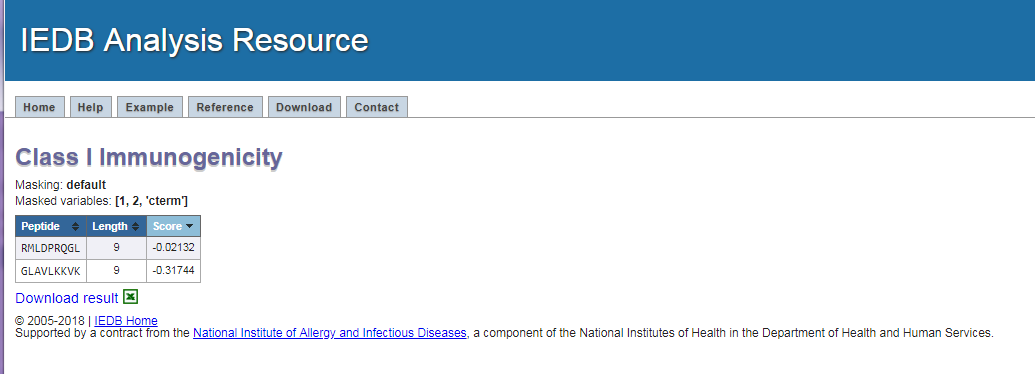

Supplement: Supplementary file 1 — Supplementary Information. [file 41598_2024_60680_MOESM1_ESM.zip › Yellow_Fever_data/2_Prediction of T-cell epitopes/IEDB_Immunogenicity/PROTEINA C.png]

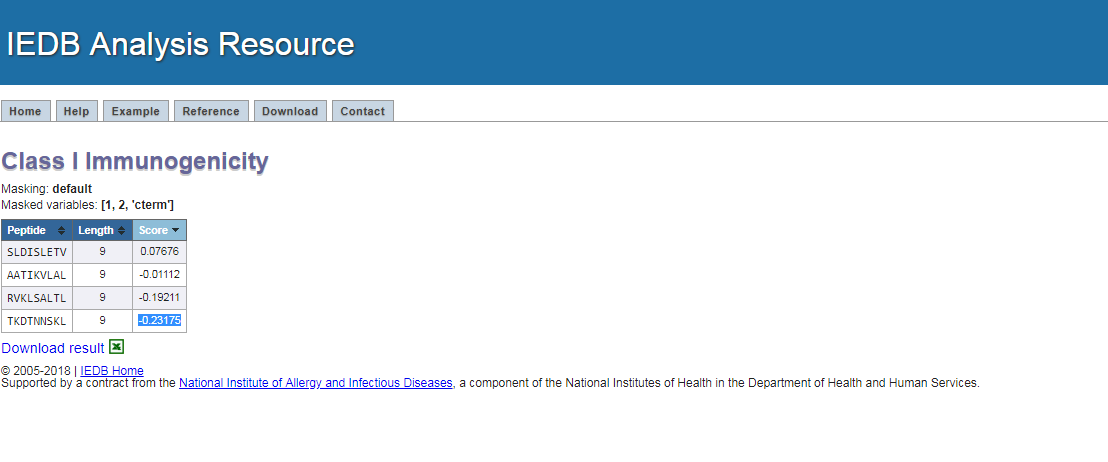

Supplement: Supplementary file 1 — Supplementary Information. [file 41598_2024_60680_MOESM1_ESM.zip › Yellow_Fever_data/2_Prediction of T-cell epitopes/IEDB_Immunogenicity/PROTEINA E.png]
