# Supplementary figures and images for: Advancing molecular modeling and reverse vaccinology in broad-spectrum yellow fever virus vaccine development (part 1 of 2)
Source: Sci Rep. 2024 May 12;14:10842. doi: 10.1038/s41598-024-60680-9 (PMC11089047; doi:10.1038/s41598-024-60680-9)

(A)

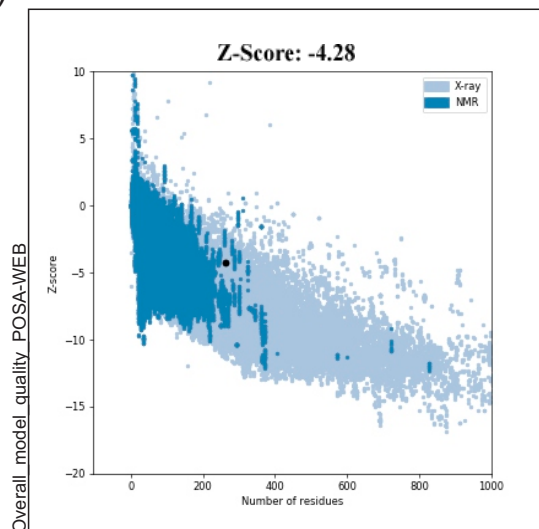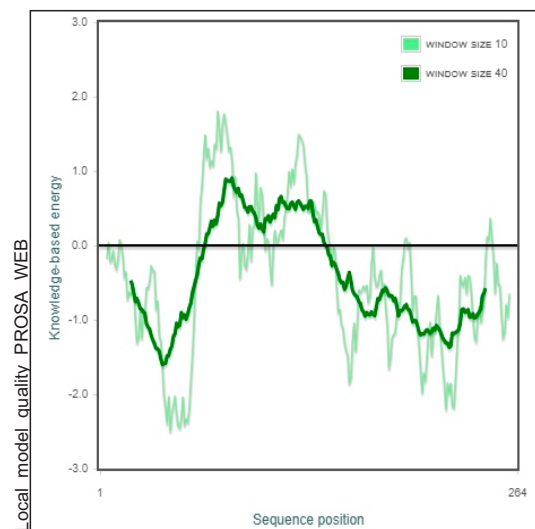

(B)

Quality\_Factor A 87.2038\_ERRAT

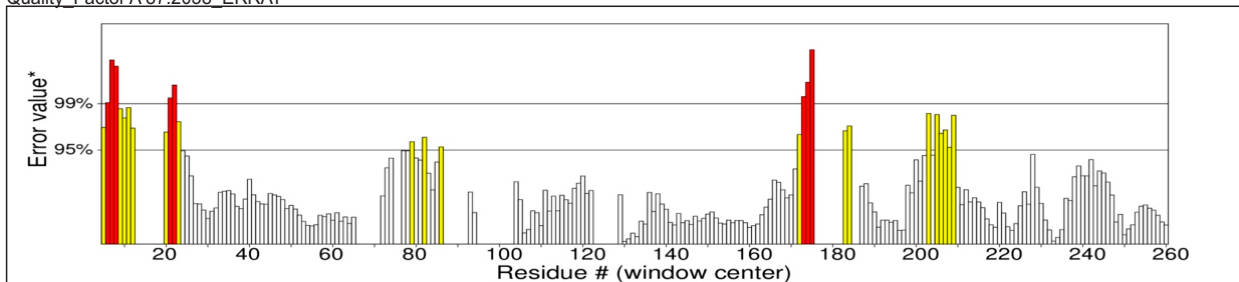

(C)

Score 89.02% Verify3D

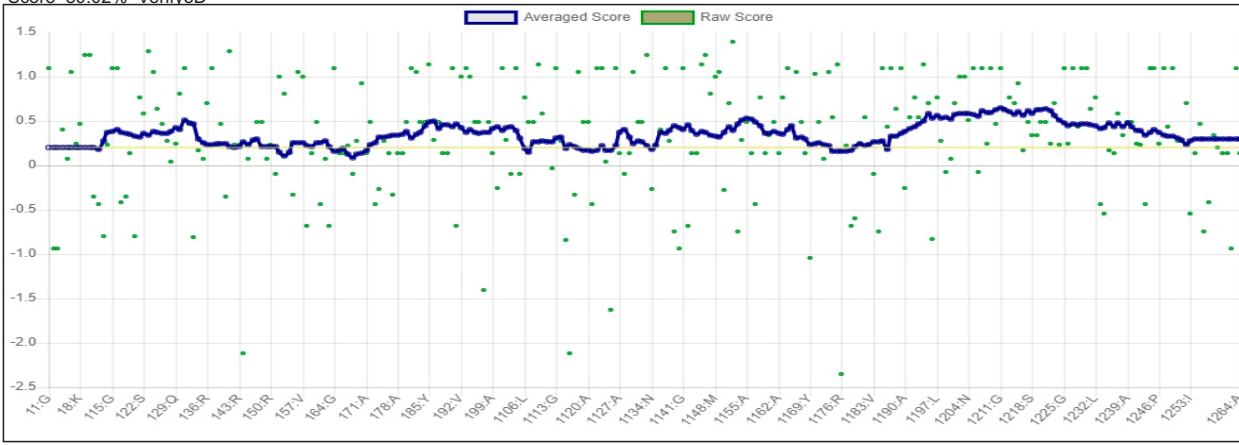

Supplement: Supplementary file 1 — Supplementary Information. [file 41598_2024_60680_MOESM1_ESM.zip › Yellow_Fever_data/Figures_pdf/Figure_3.pdf]

(A)

H-Bonds

Donor

Acceptor

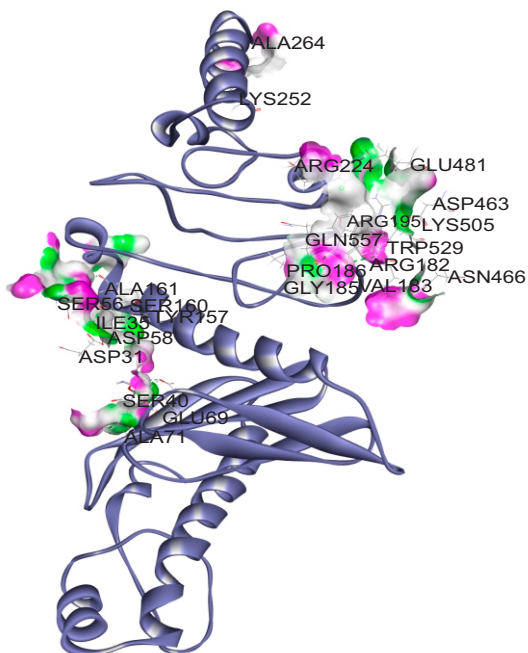

(B)

Interpolated Charge

0,100  
0,067  
0,033  
0,000  
-0,033  
-0,067  
-0,100

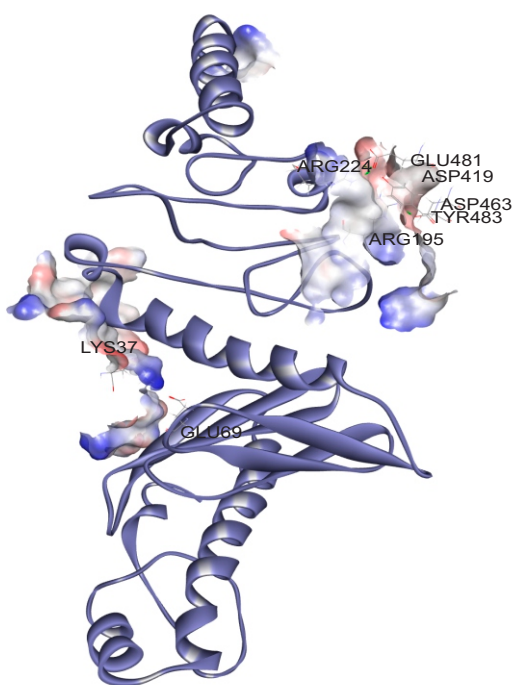

(C)

Hydrophobicity

3,00  
2,00  
1,00  
0,00  
-1,00  
-2,00  
-3,00

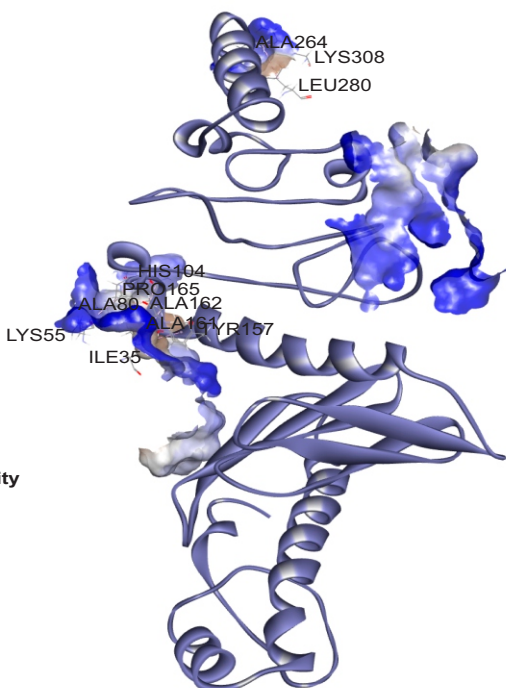

Supplement: Supplementary file 1 — Supplementary Information. [file 41598_2024_60680_MOESM1_ESM.zip › Yellow_Fever_data/Figures_pdf/Figure_5.pdf]

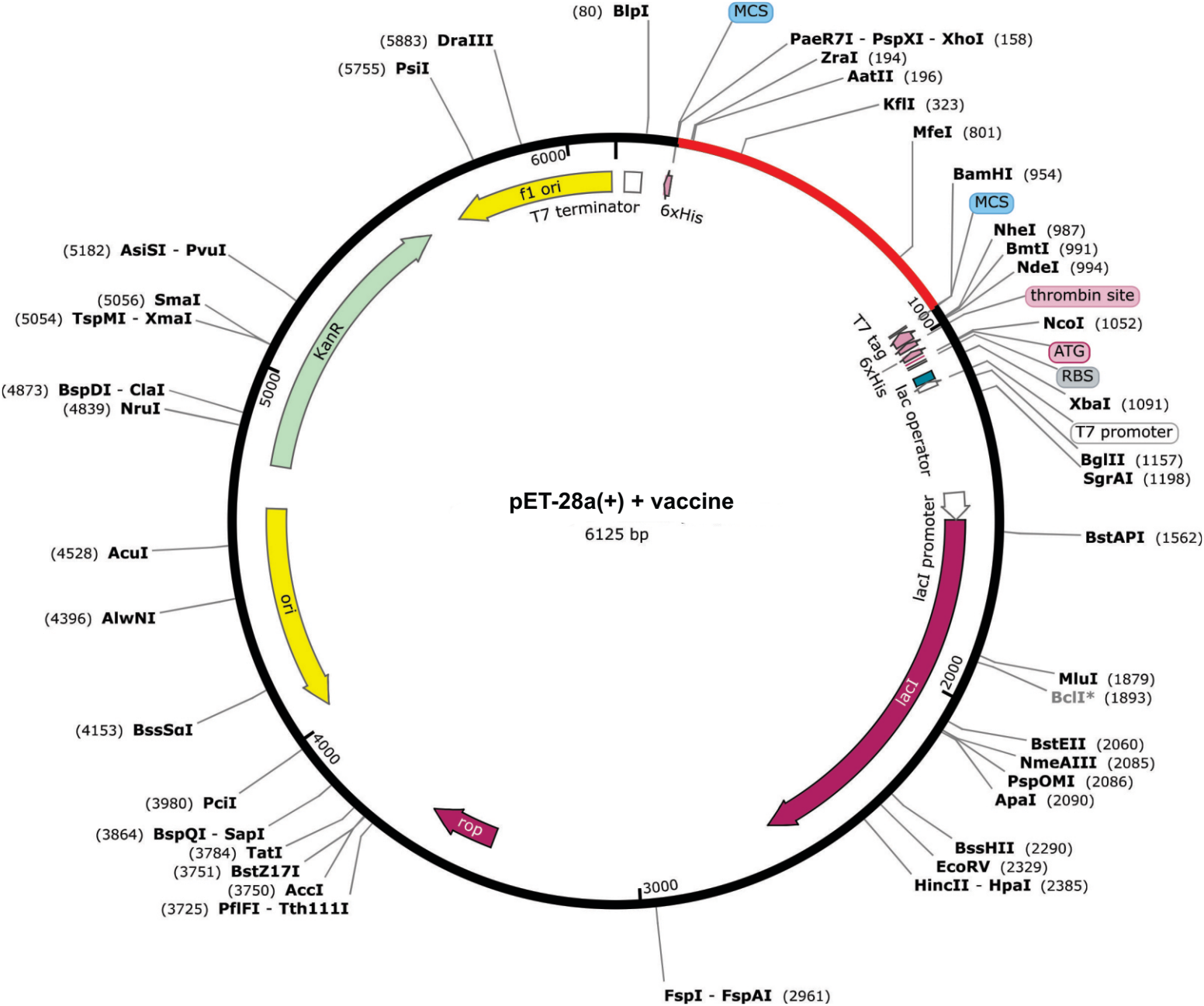

Supplement: Supplementary file 1 — Supplementary Information. [file 41598_2024_60680_MOESM1_ESM.zip › Yellow_Fever_data/Figures_pdf/Figure_6.pdf]

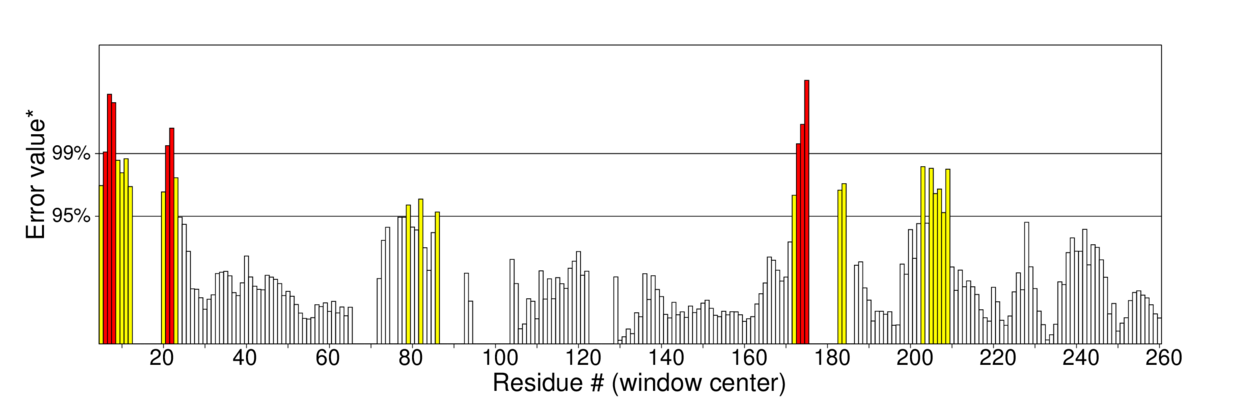

Supplement: Supplementary file 1 — Supplementary Information. [file 41598_2024_60680_MOESM1_ESM.zip › Yellow_Fever_data/4_Vaccine_validation/errat.bmp]

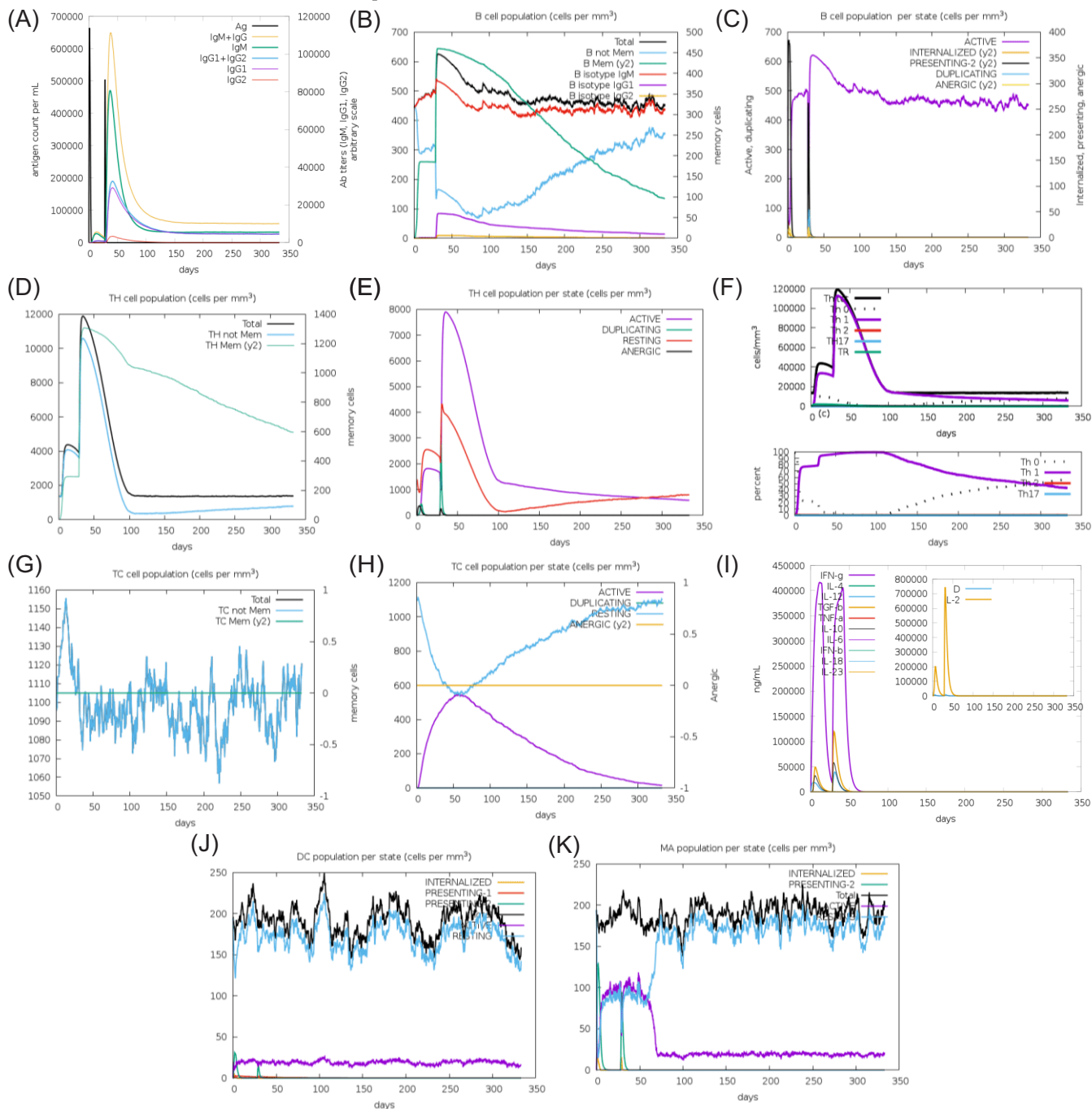

Supplement: Supplementary file 1 — Supplementary Information. [file 41598_2024_60680_MOESM1_ESM.zip › Yellow_Fever_data/8_Immune_response/Figure_7.pdf]

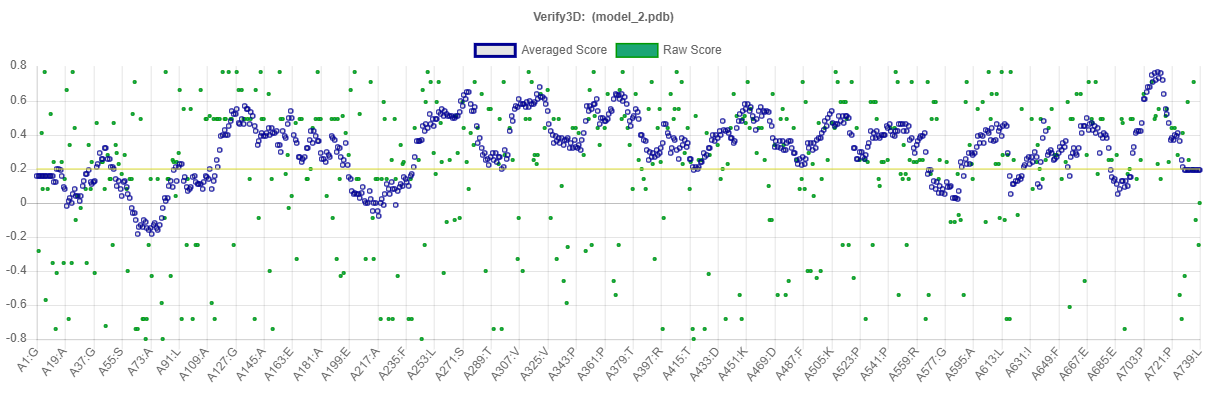

Supplement: Supplementary file 1 — Supplementary Information. [file 41598_2024_60680_MOESM1_ESM.zip › Yellow_Fever_data/4_Vaccine_validation/verify3d.png]

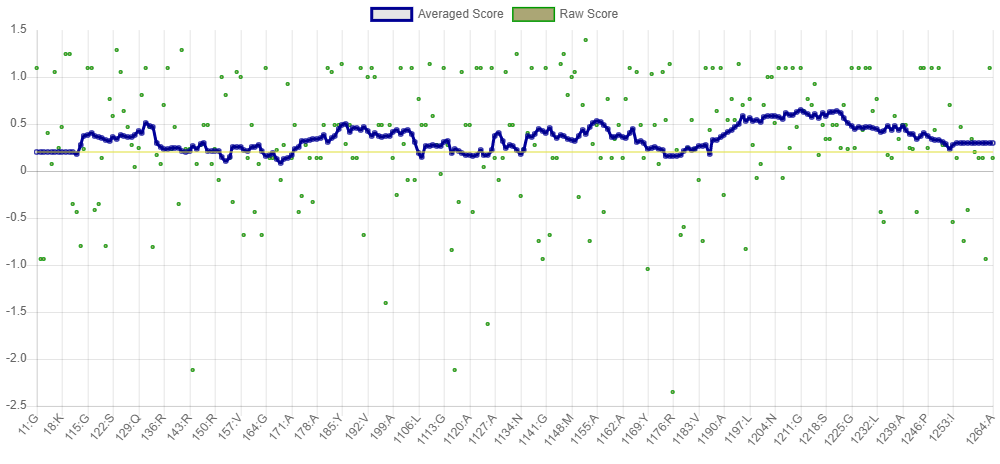

Supplement: Supplementary file 1 — Supplementary Information. [file 41598_2024_60680_MOESM1_ESM.zip › Yellow_Fever_data/4_Vaccine_validation/plot_vf11.bmp]

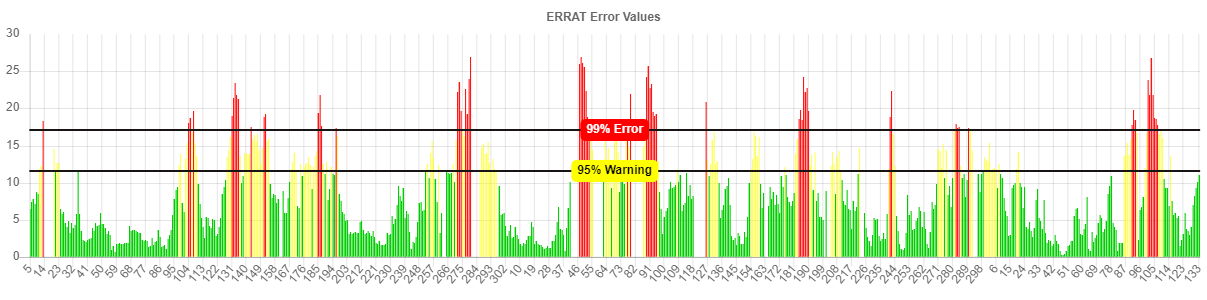

Supplement: Supplementary file 1 — Supplementary Information. [file 41598_2024_60680_MOESM1_ESM.zip › Yellow_Fever_data/4_Vaccine_validation/erratPlot1.png]

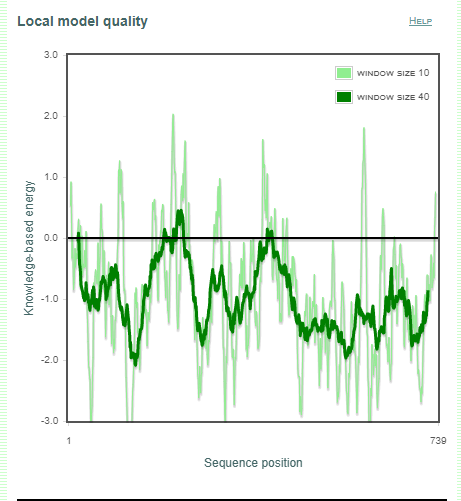

Supplement: Supplementary file 1 — Supplementary Information. [file 41598_2024_60680_MOESM1_ESM.zip › Yellow_Fever_data/4_Vaccine_validation/Sem título.png]

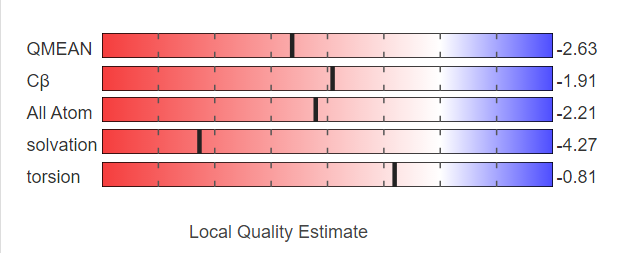

Supplement: Supplementary file 1 — Supplementary Information. [file 41598_2024_60680_MOESM1_ESM.zip › Yellow_Fever_data/4_Vaccine_validation/Sem título.bmp]

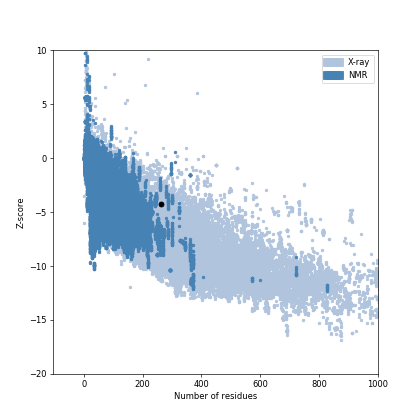

Supplement: Supplementary file 1 — Supplementary Information. [file 41598_2024_60680_MOESM1_ESM.zip › Yellow_Fever_data/4_Vaccine_validation/zplot_94lPtG.png]

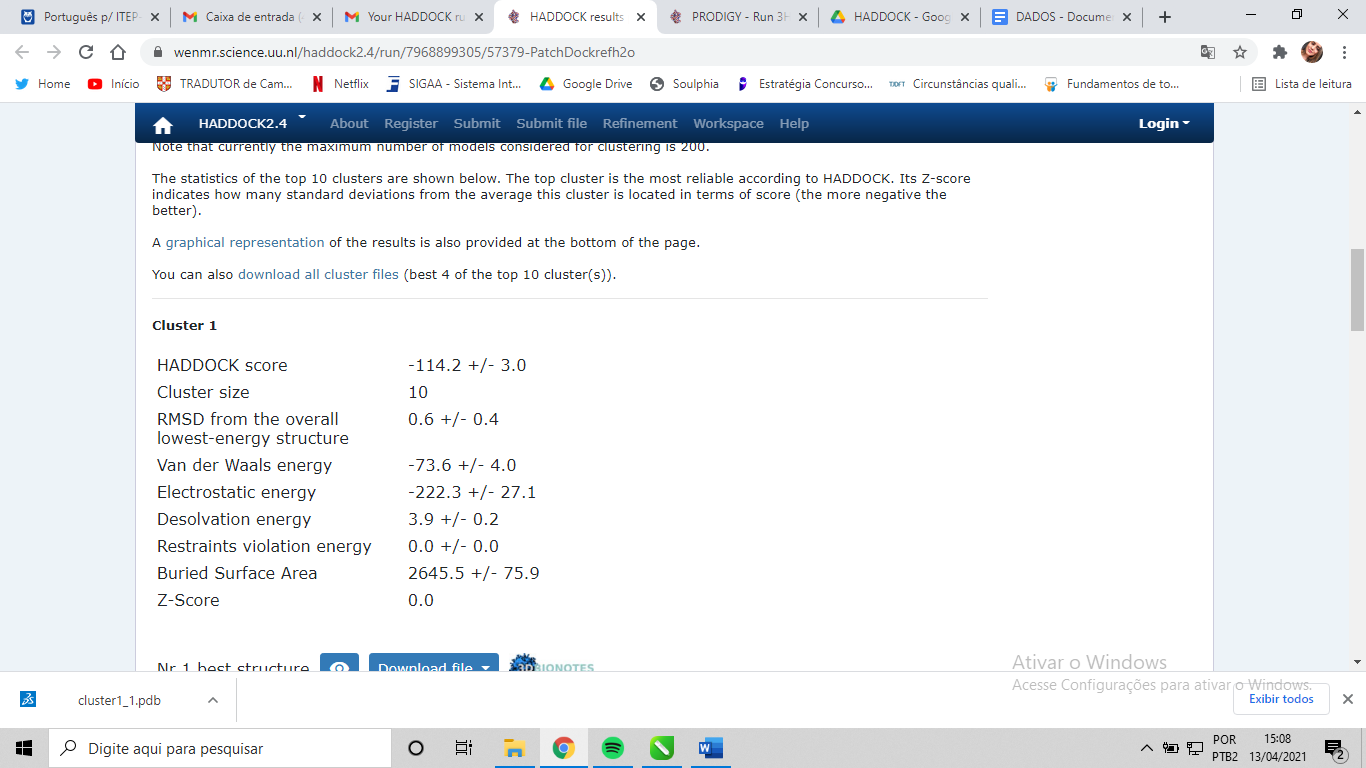


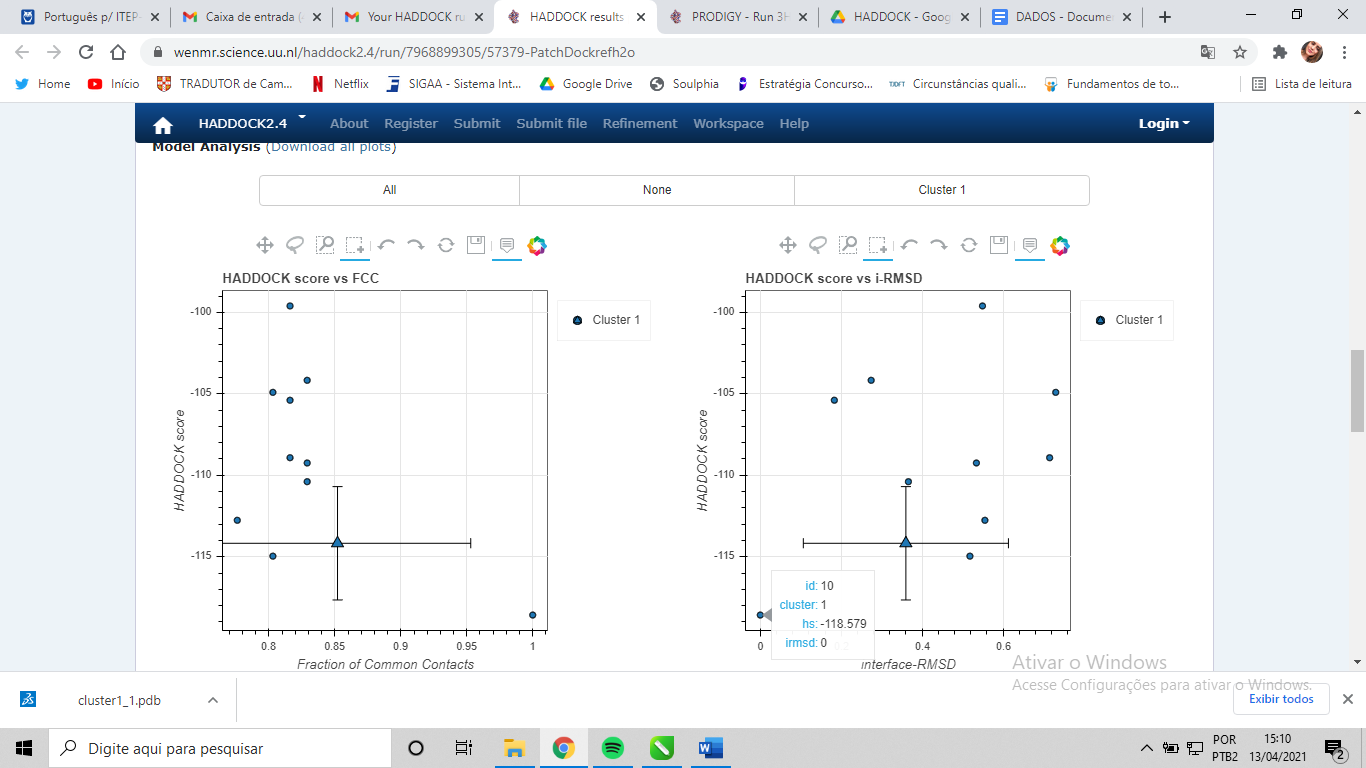


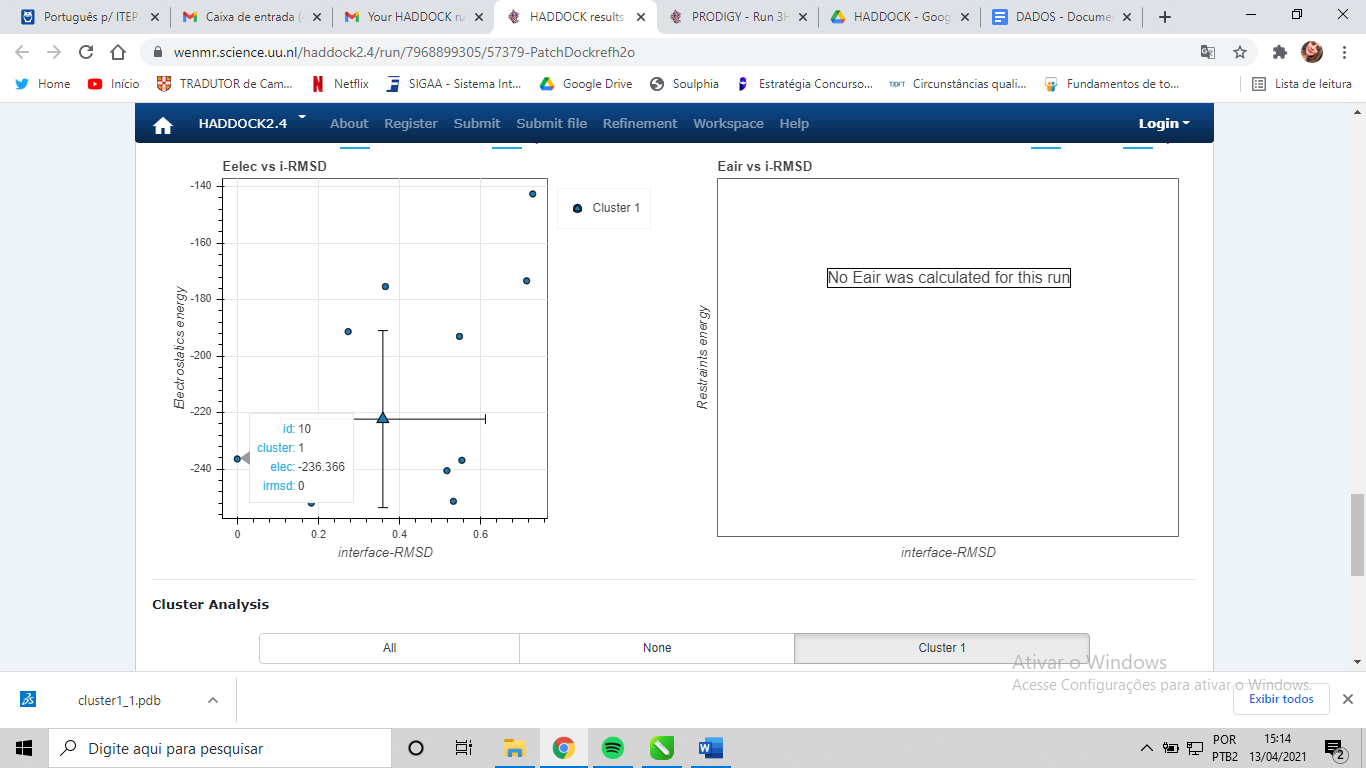

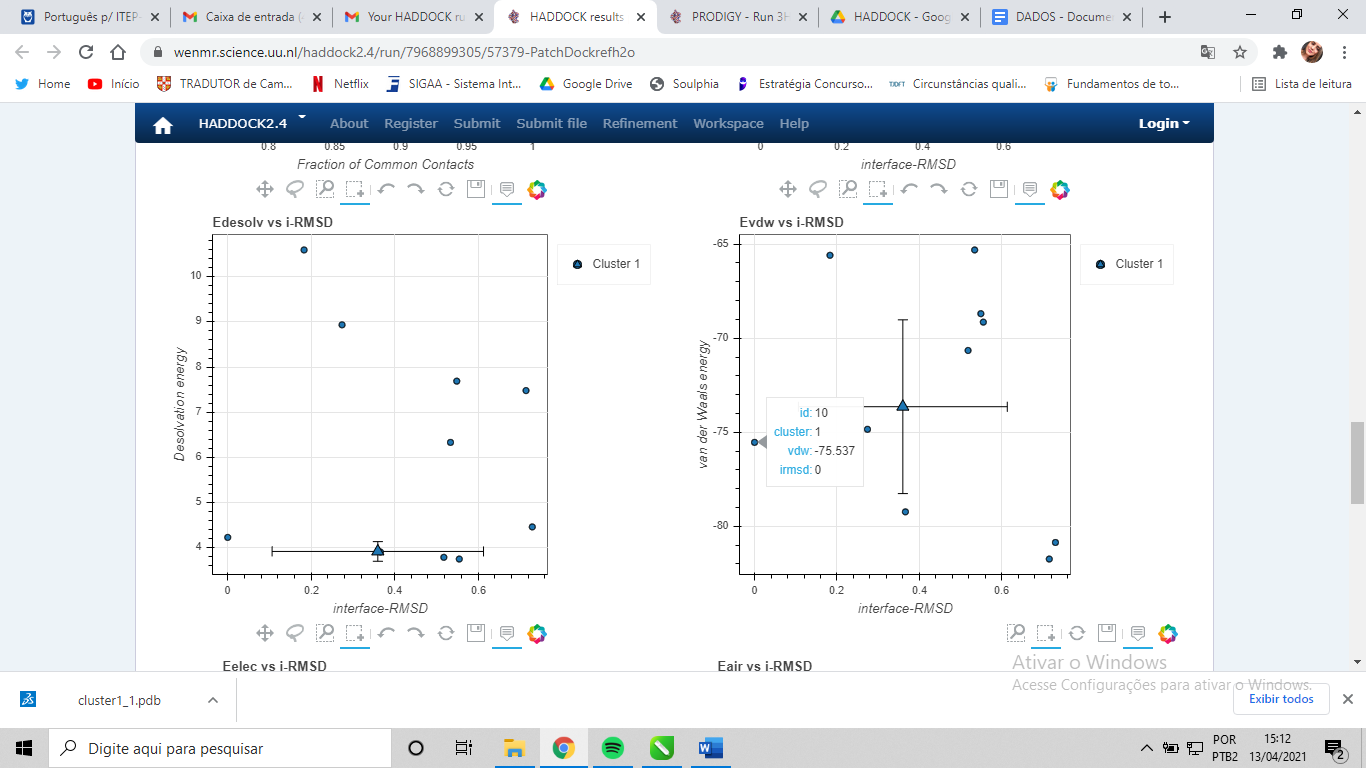

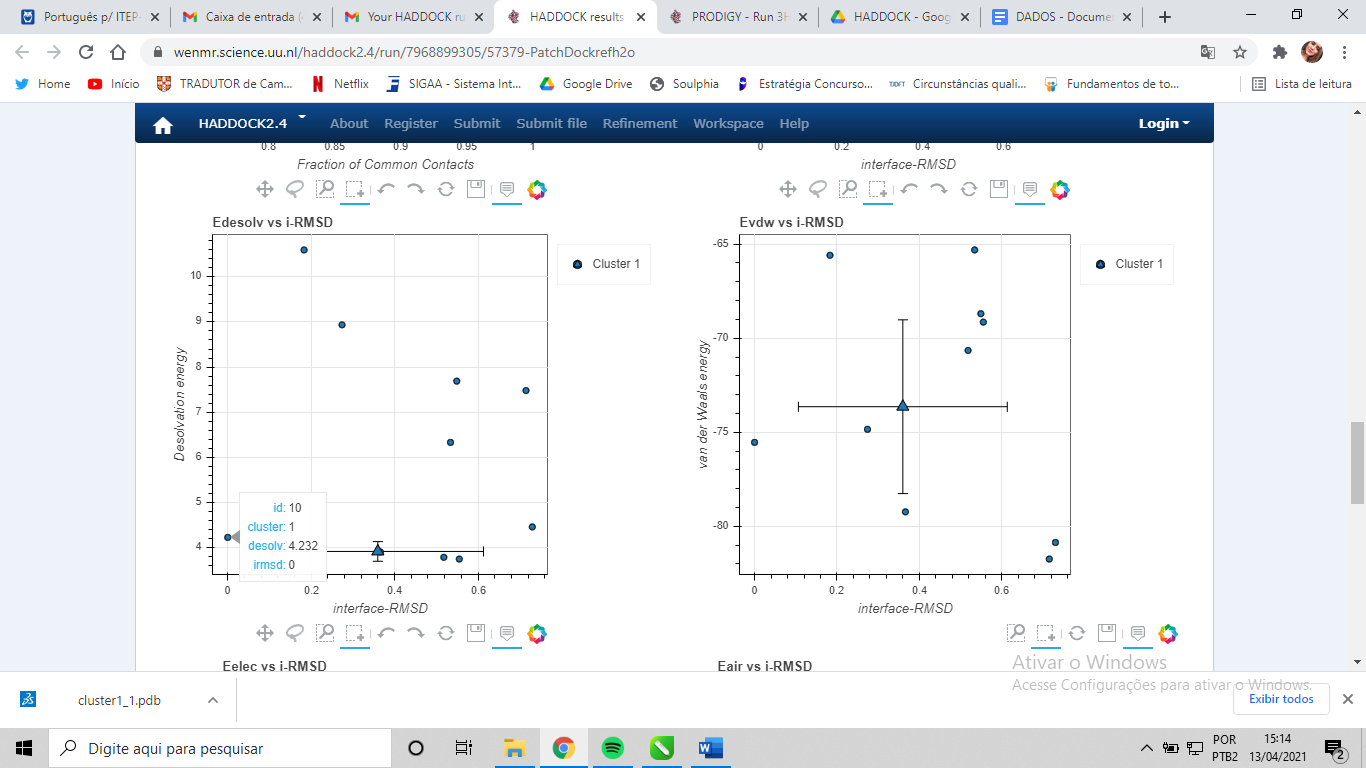

Supplement: Supplementary file 1 — Supplementary Information. [file 41598_2024_60680_MOESM1_ESM.zip › Yellow_Fever_data/5_Molecular docking/HADDOCK/Data.docx]

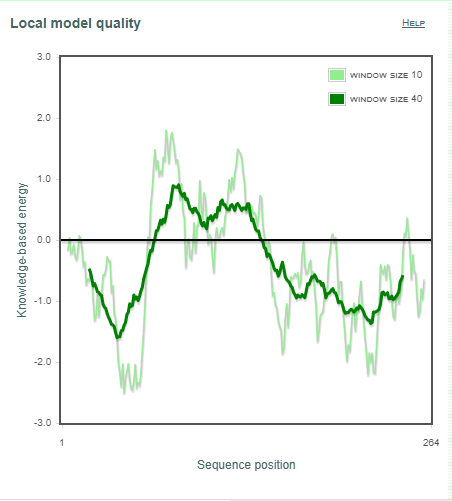

Supplement: Supplementary file 1 — Supplementary Information. [file 41598_2024_60680_MOESM1_ESM.zip › Yellow_Fever_data/4_Vaccine_validation/model quality.bmp]

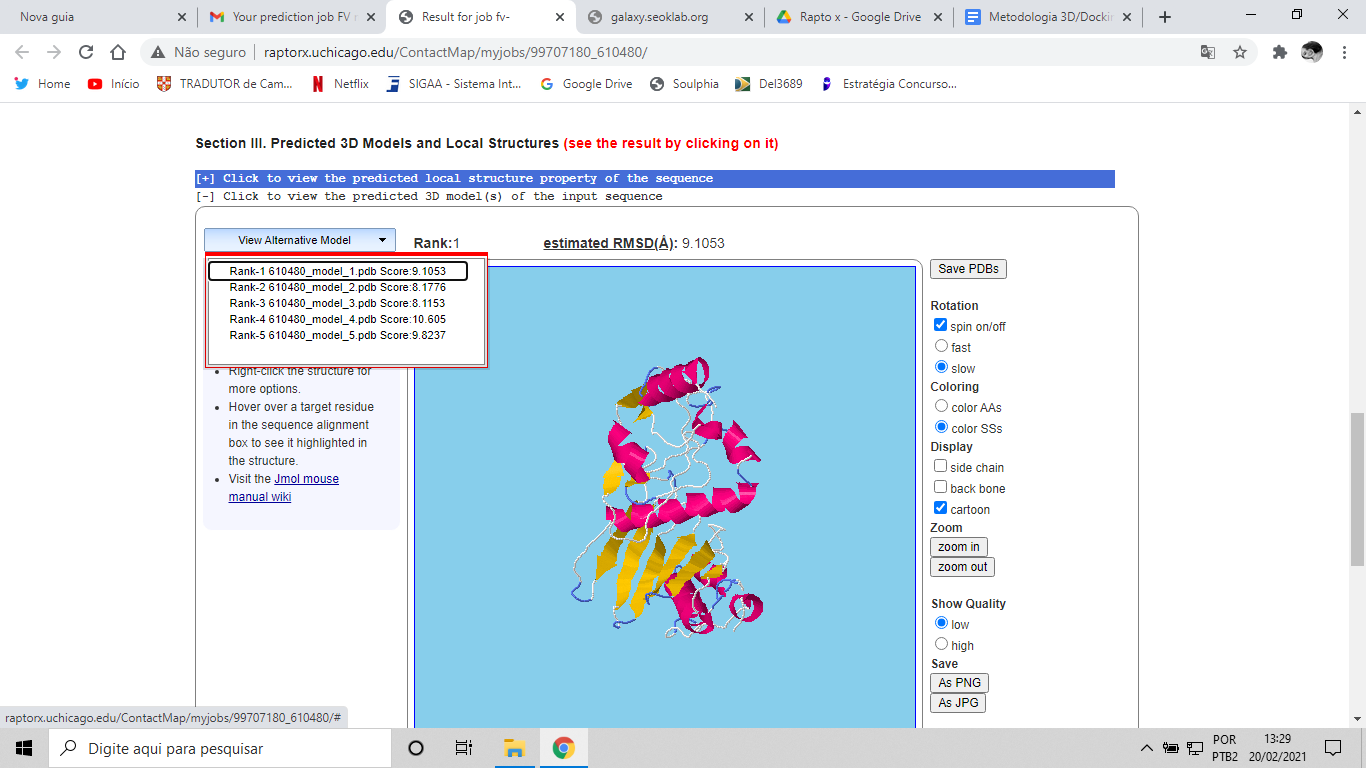

Supplement: Supplementary file 1 — Supplementary Information. [file 41598_2024_60680_MOESM1_ESM.zip › Yellow_Fever_data/5_Molecular docking/Raptor x/Rank.docx]

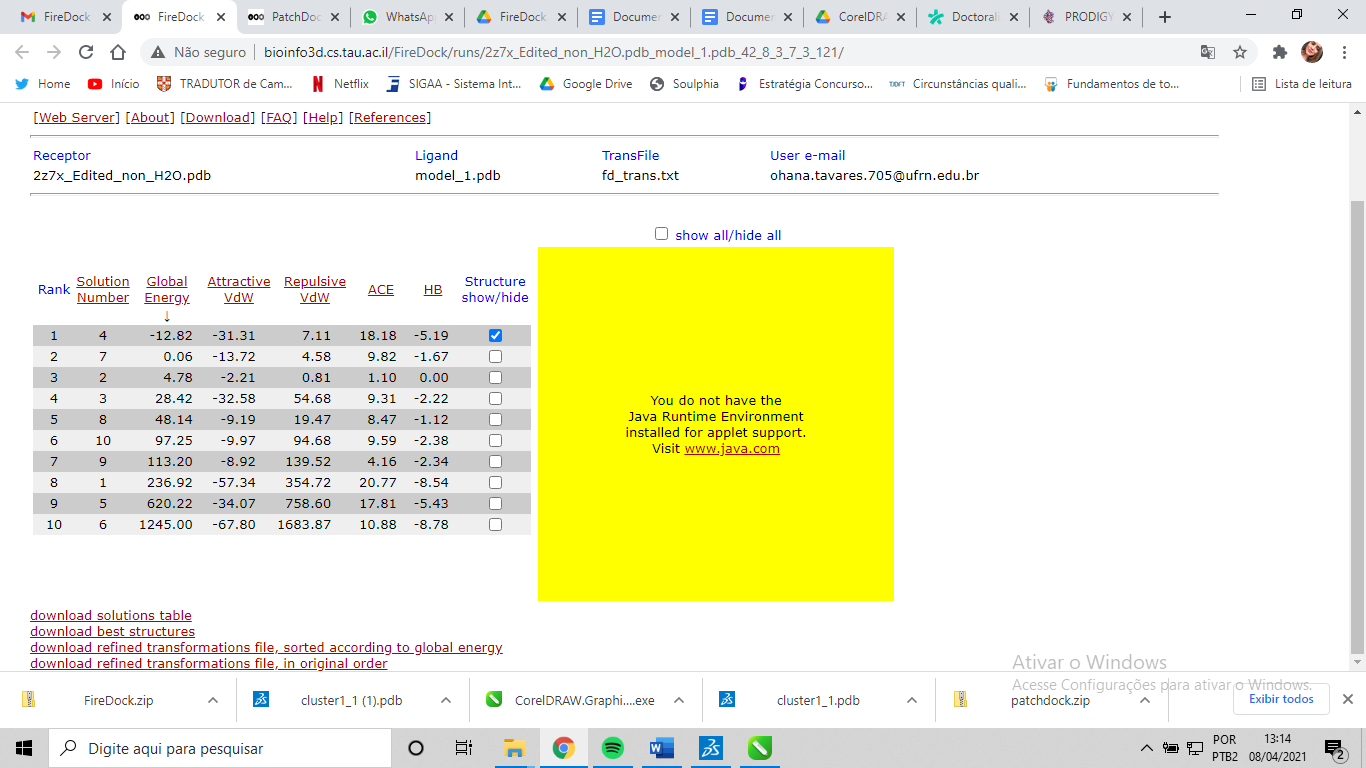

Supplement: Supplementary file 1 — Supplementary Information. [file 41598_2024_60680_MOESM1_ESM.zip › Yellow_Fever_data/5_Molecular docking/Firedock/Print.docx]

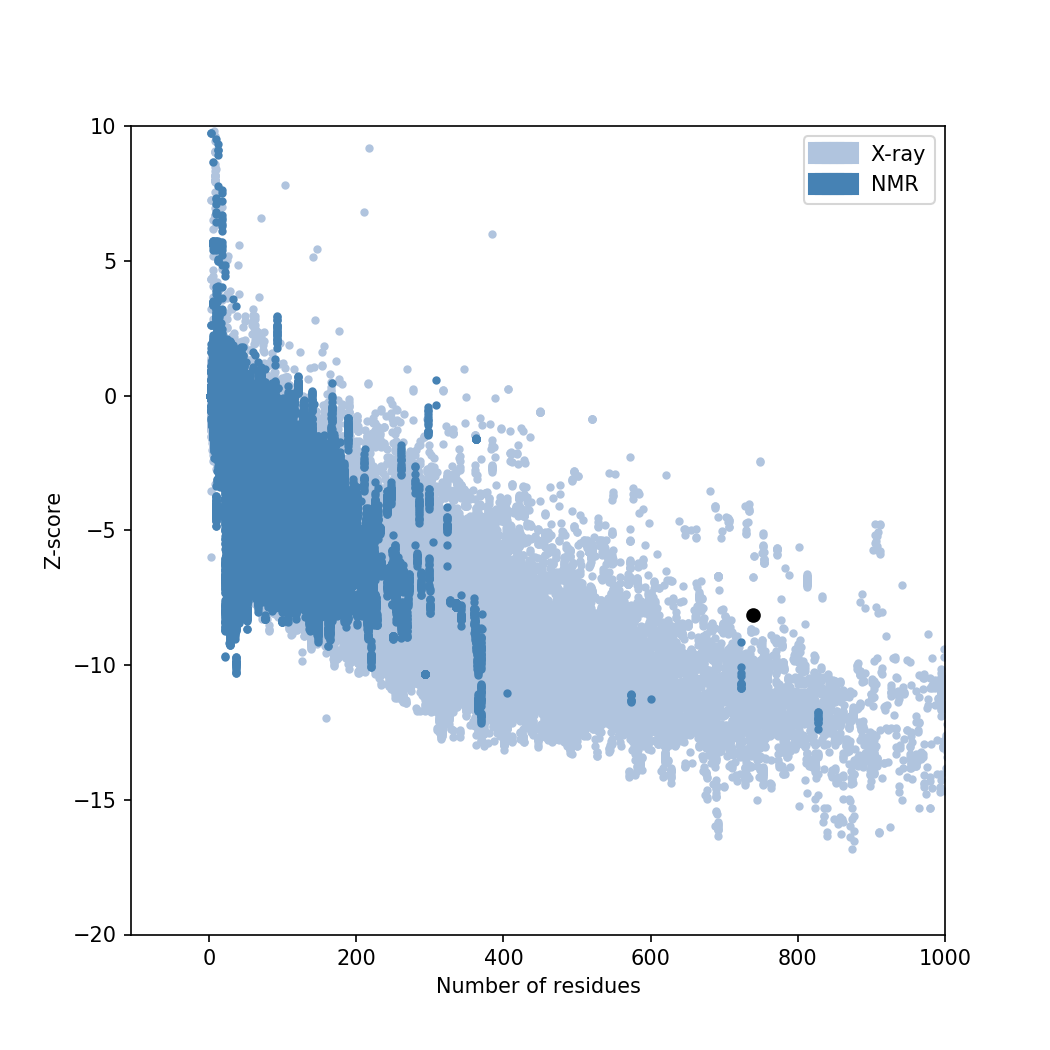

Supplement: Supplementary file 1 — Supplementary Information. [file 41598_2024_60680_MOESM1_ESM.zip › Yellow_Fever_data/4_Vaccine_validation/hr_zplot_8MGZLR.png]

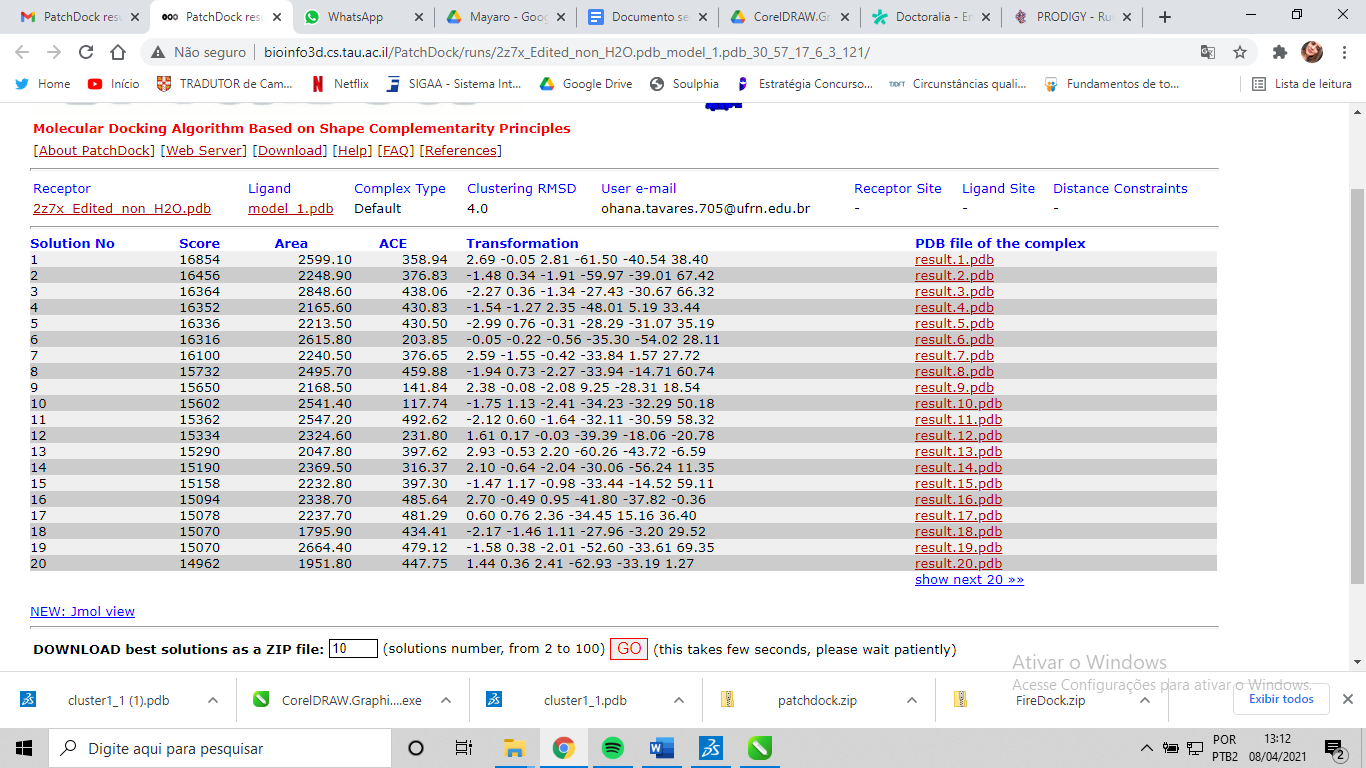

Supplement: Supplementary file 1 — Supplementary Information. [file 41598_2024_60680_MOESM1_ESM.zip › Yellow_Fever_data/5_Molecular docking/PatchDock/Table.docx]

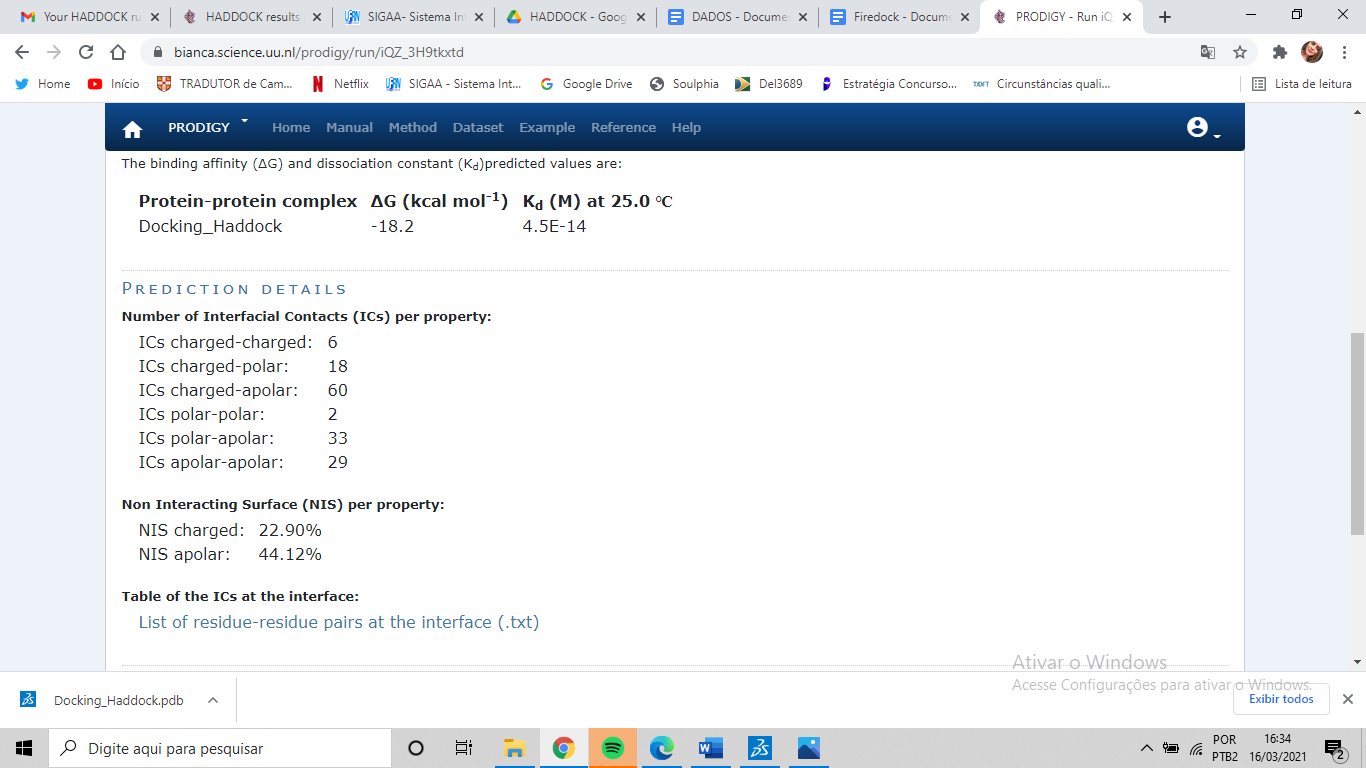

Supplement: Supplementary file 1 — Supplementary Information. [file 41598_2024_60680_MOESM1_ESM.zip › Yellow_Fever_data/5_Molecular docking/Prodigy/Haddock.docx]

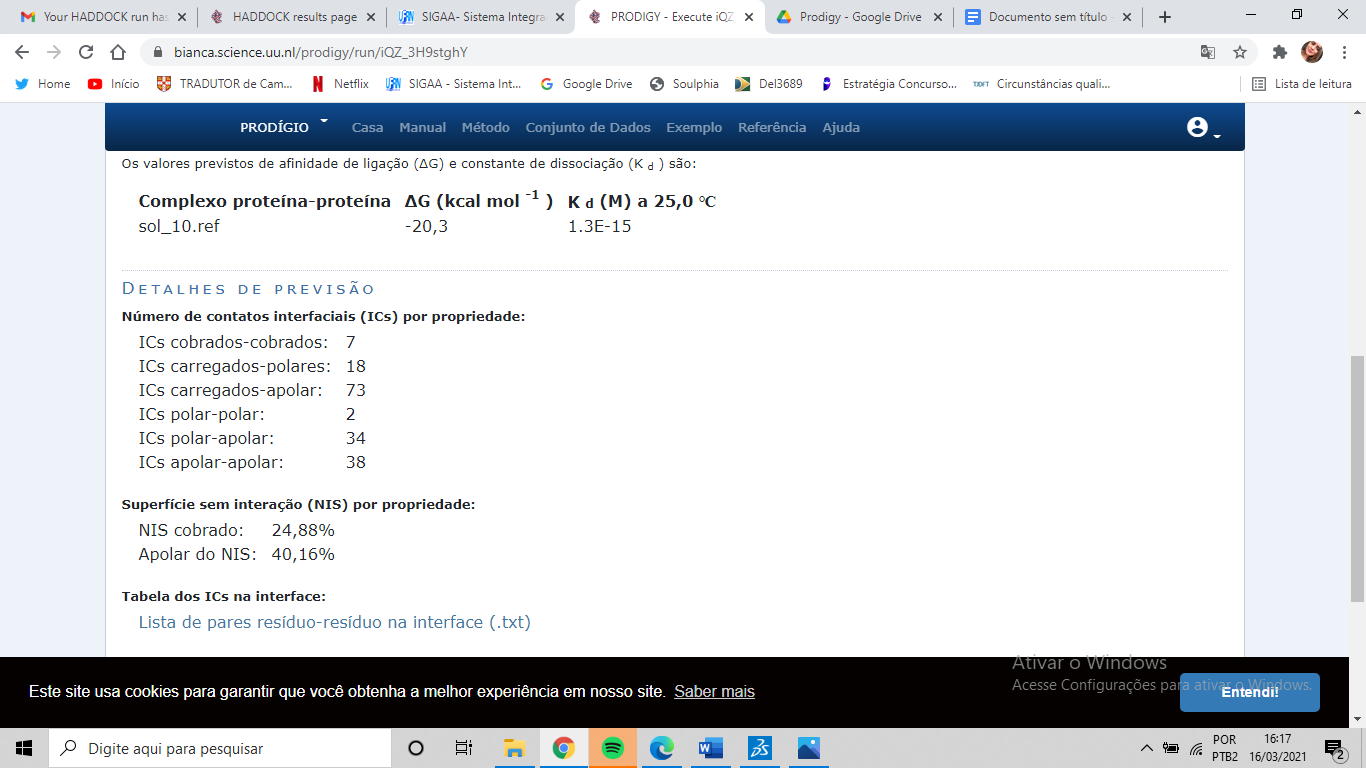

Supplement: Supplementary file 1 — Supplementary Information. [file 41598_2024_60680_MOESM1_ESM.zip › Yellow_Fever_data/5_Molecular docking/Prodigy/Firedock.docx]

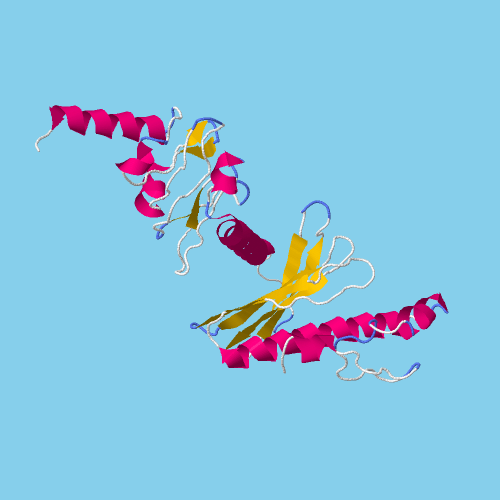

Supplement: Supplementary file 1 — Supplementary Information. [file 41598_2024_60680_MOESM1_ESM.zip › Yellow_Fever_data/5_Molecular docking/Raptor x/610480-R.png]

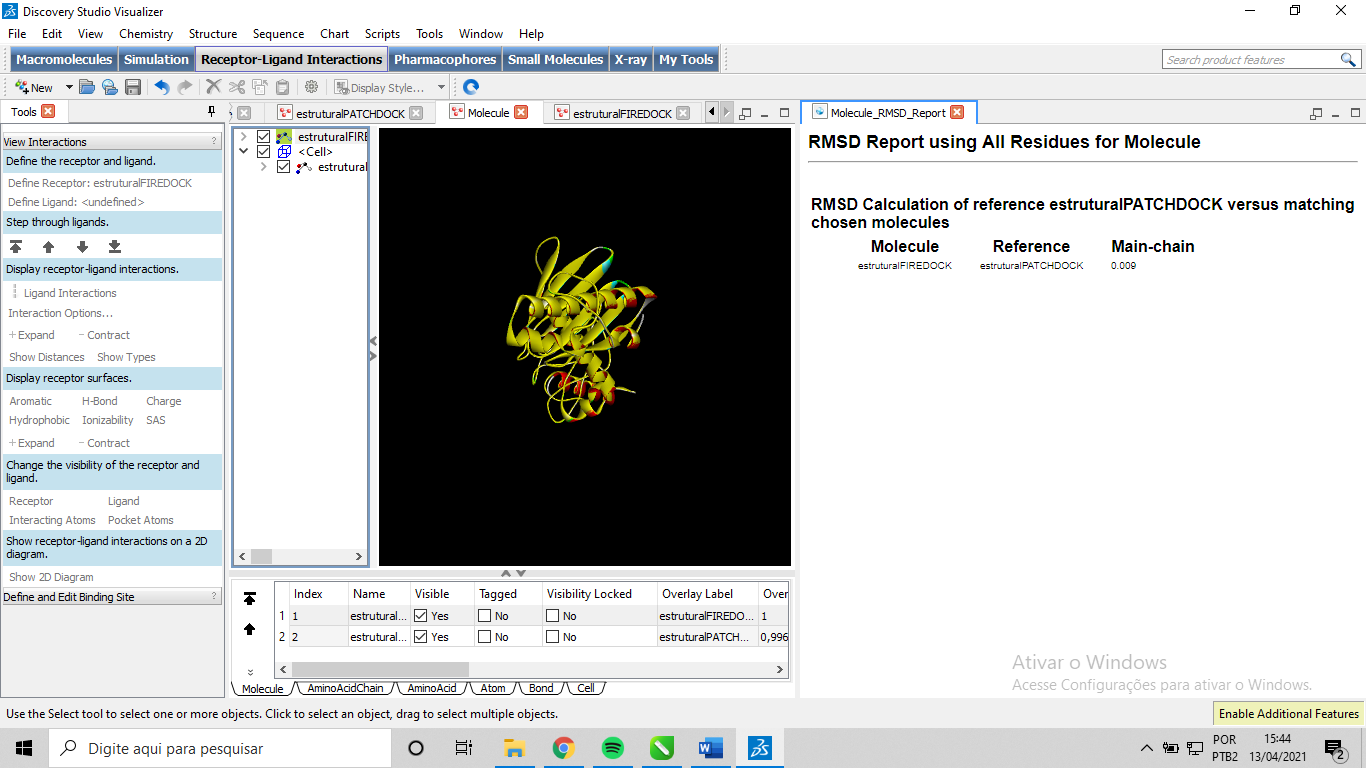


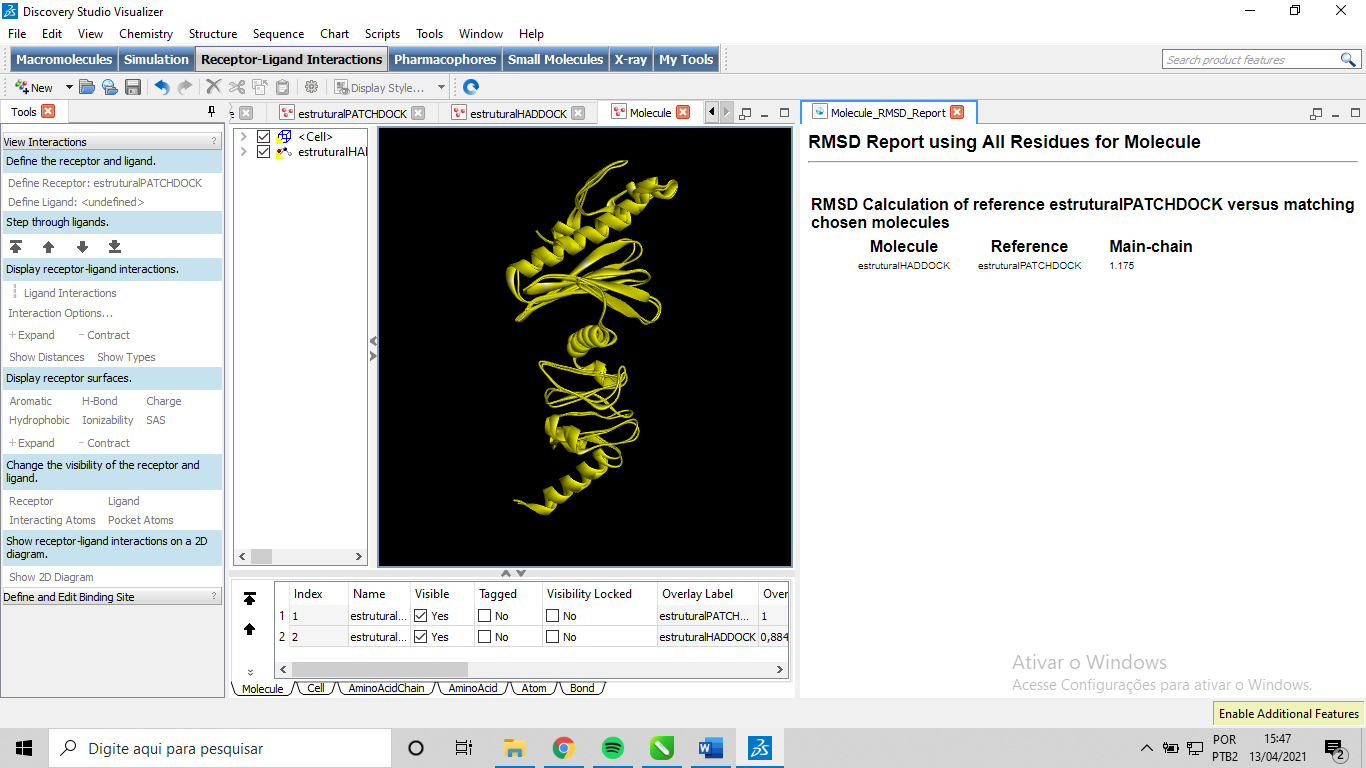

Supplement: Supplementary file 1 — Supplementary Information. [file 41598_2024_60680_MOESM1_ESM.zip › Yellow_Fever_data/5_Molecular docking/RMSD/Docking 2.0.docx]

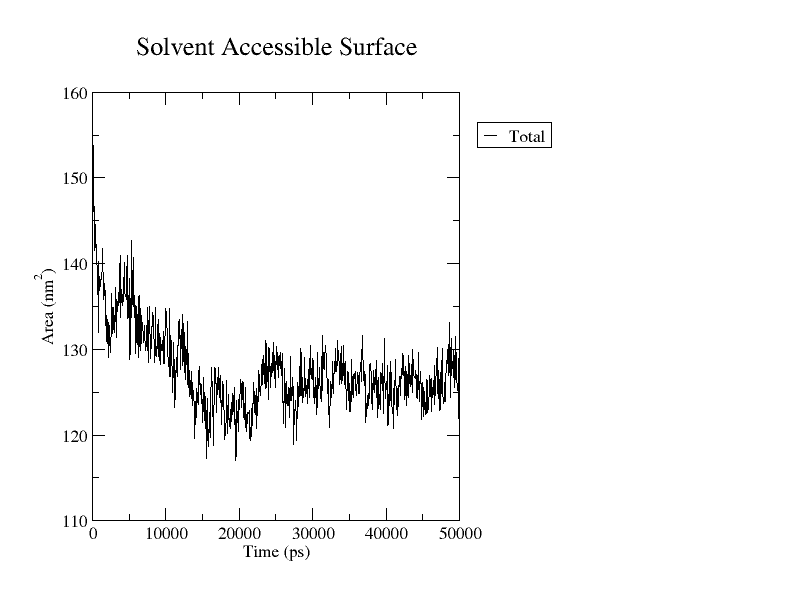

Supplement: Supplementary file 1 — Supplementary Information. [file 41598_2024_60680_MOESM1_ESM.zip › Yellow_Fever_data/7_Molecular Dynamics/SAS-clean-protein.png]

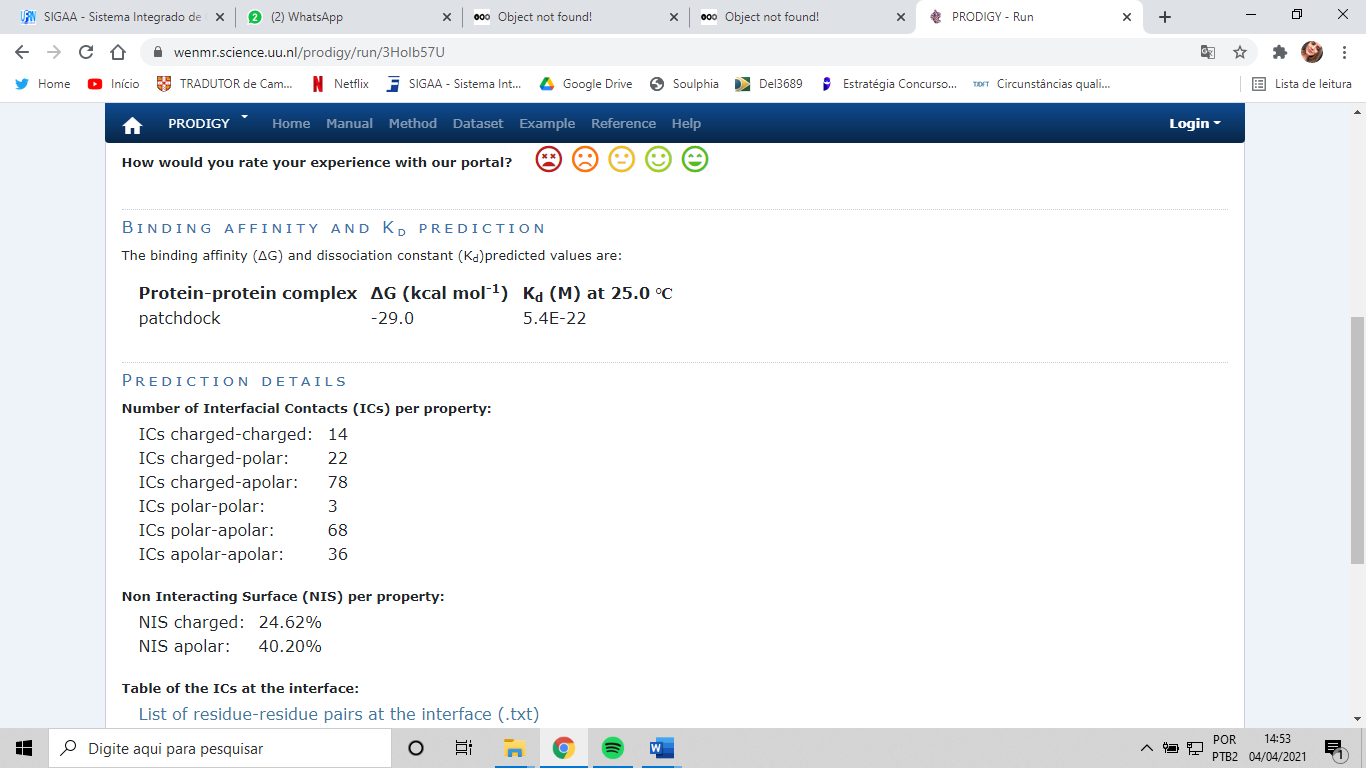

Supplement: Supplementary file 1 — Supplementary Information. [file 41598_2024_60680_MOESM1_ESM.zip › Yellow_Fever_data/5_Molecular docking/Prodigy/Patchdock.docx]

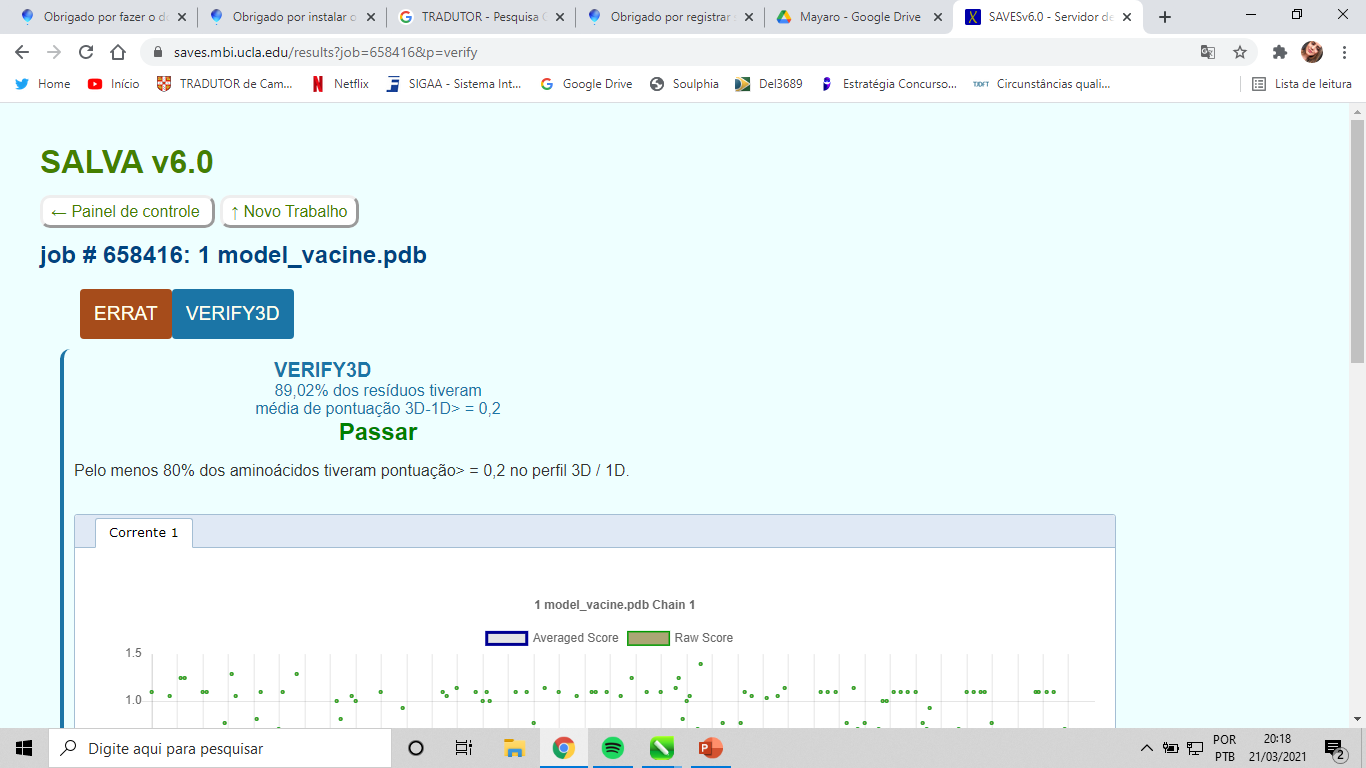


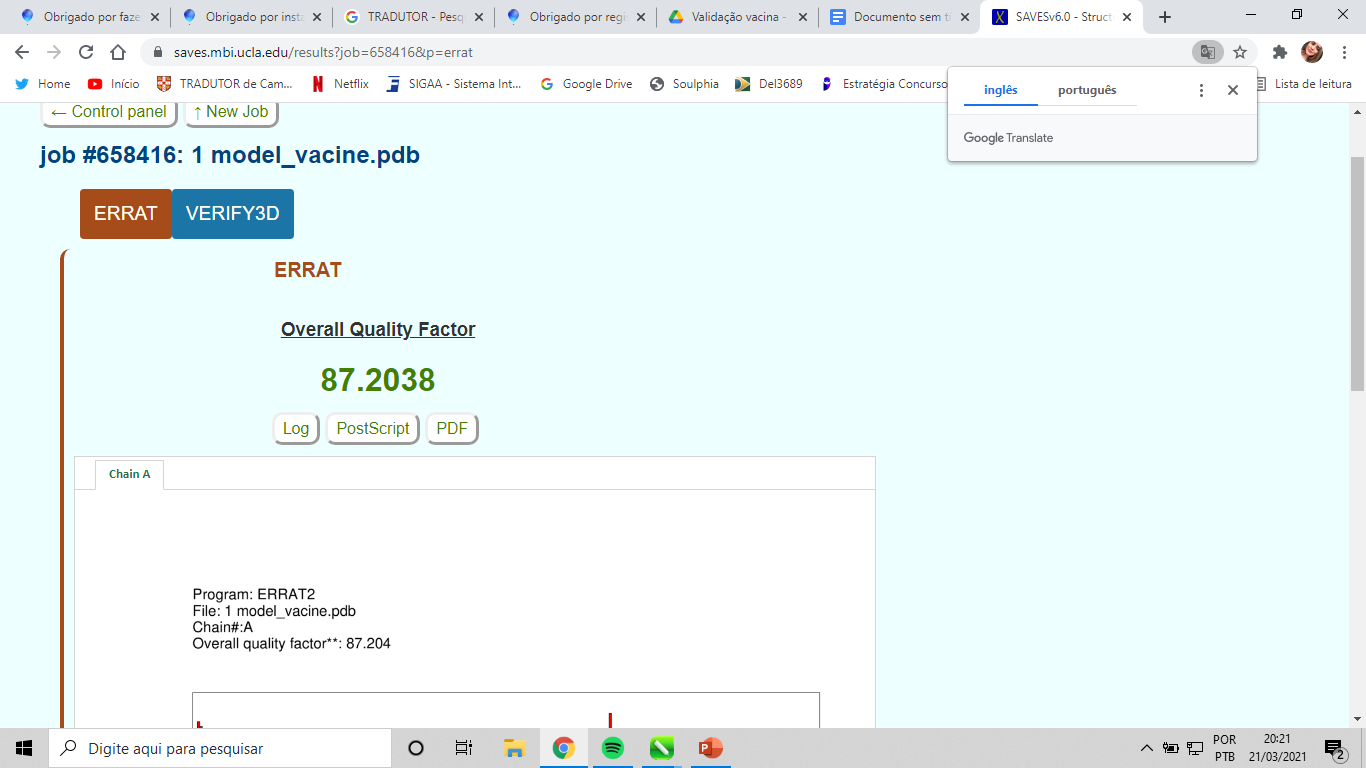

Supplement: Supplementary file 1 — Supplementary Information. [file 41598_2024_60680_MOESM1_ESM.zip › Yellow_Fever_data/4_Vaccine_validation/ERRAT e VERIFYC3D.docx]

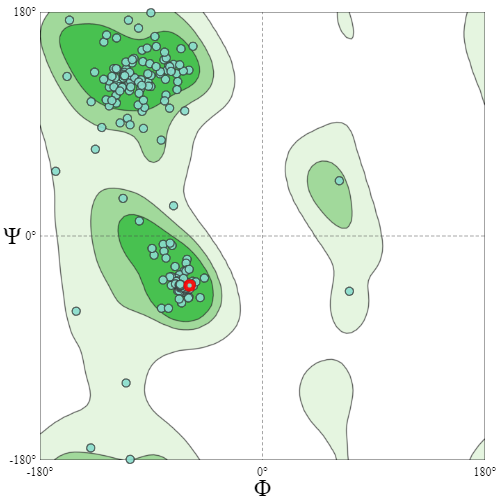

Supplement: Supplementary file 1 — Supplementary Information. [file 41598_2024_60680_MOESM1_ESM.zip › Yellow_Fever_data/4_Vaccine_validation/model_01_general_A.png]

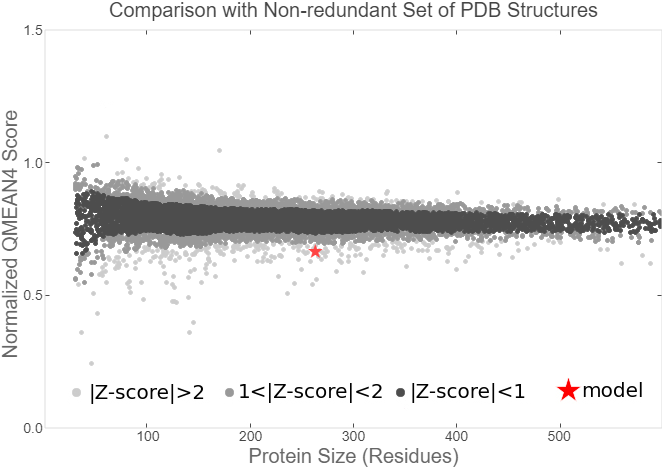

Supplement: Supplementary file 1 — Supplementary Information. [file 41598_2024_60680_MOESM1_ESM.zip › Yellow_Fever_data/4_Vaccine_validation/Quality_comparison.bmp]

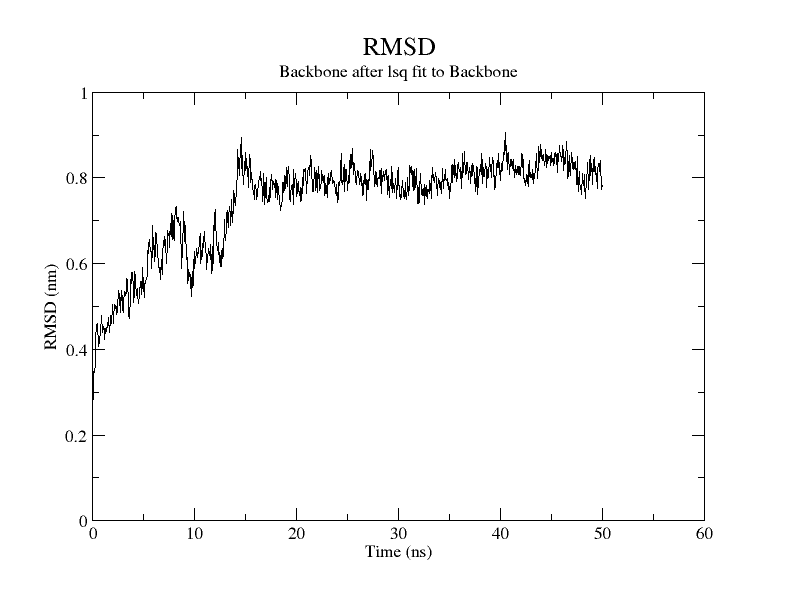

Supplement: Supplementary file 1 — Supplementary Information. [file 41598_2024_60680_MOESM1_ESM.zip › Yellow_Fever_data/7_Molecular Dynamics/rmsd-clean-protein.png]

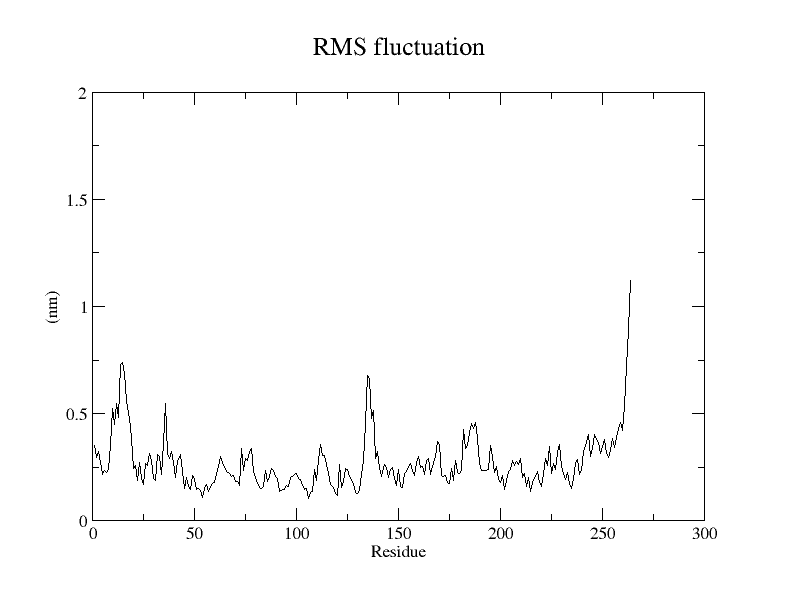

Supplement: Supplementary file 1 — Supplementary Information. [file 41598_2024_60680_MOESM1_ESM.zip › Yellow_Fever_data/7_Molecular Dynamics/rmsf-clean-protein.png]

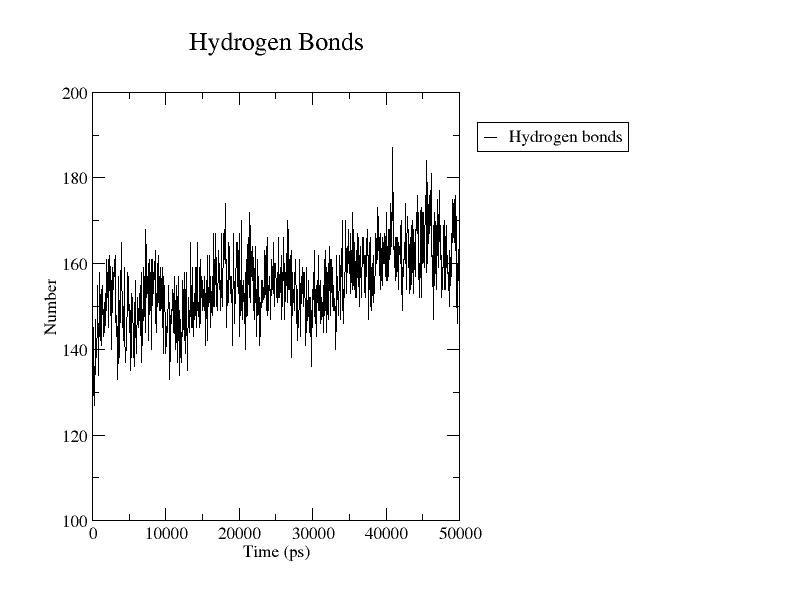

Supplement: Supplementary file 1 — Supplementary Information. [file 41598_2024_60680_MOESM1_ESM.zip › Yellow_Fever_data/7_Molecular Dynamics/hbond-clean-protein.png]

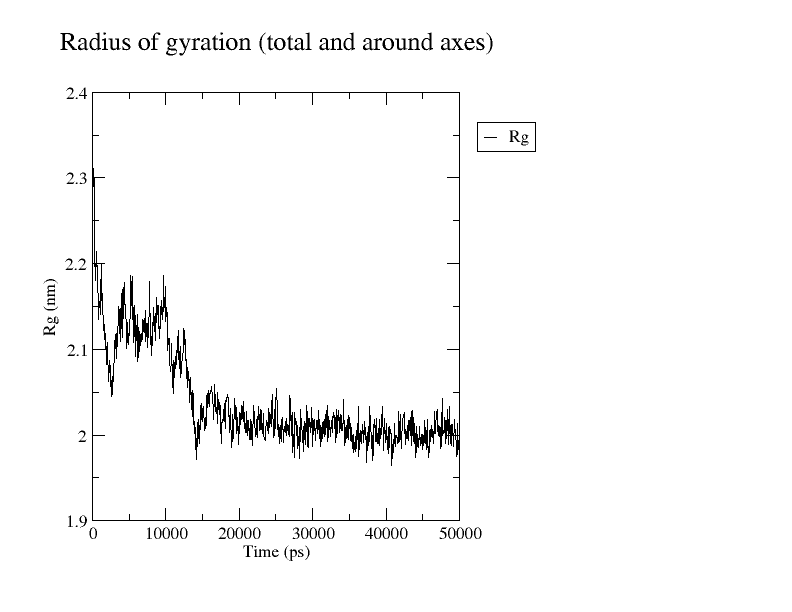

Supplement: Supplementary file 1 — Supplementary Information. [file 41598_2024_60680_MOESM1_ESM.zip › Yellow_Fever_data/7_Molecular Dynamics/gyrate-clean-protein.png]

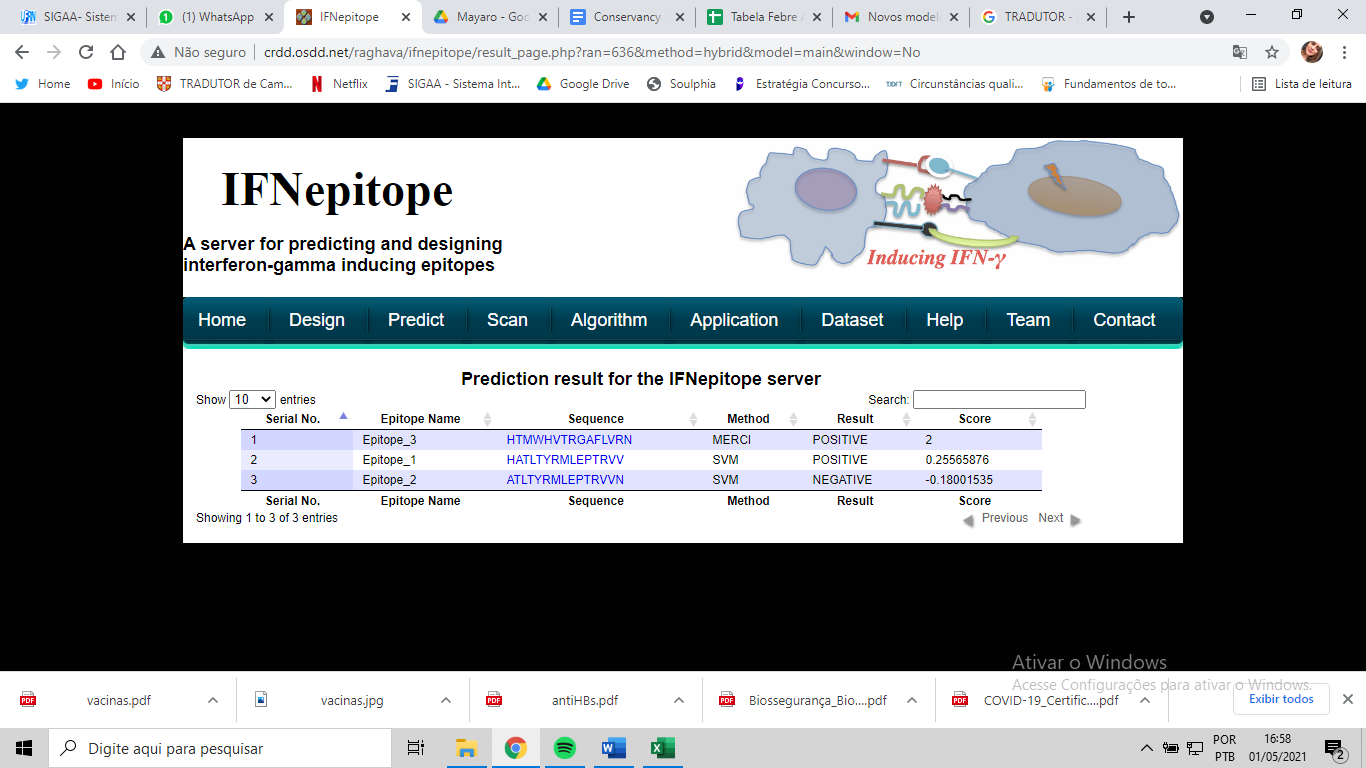


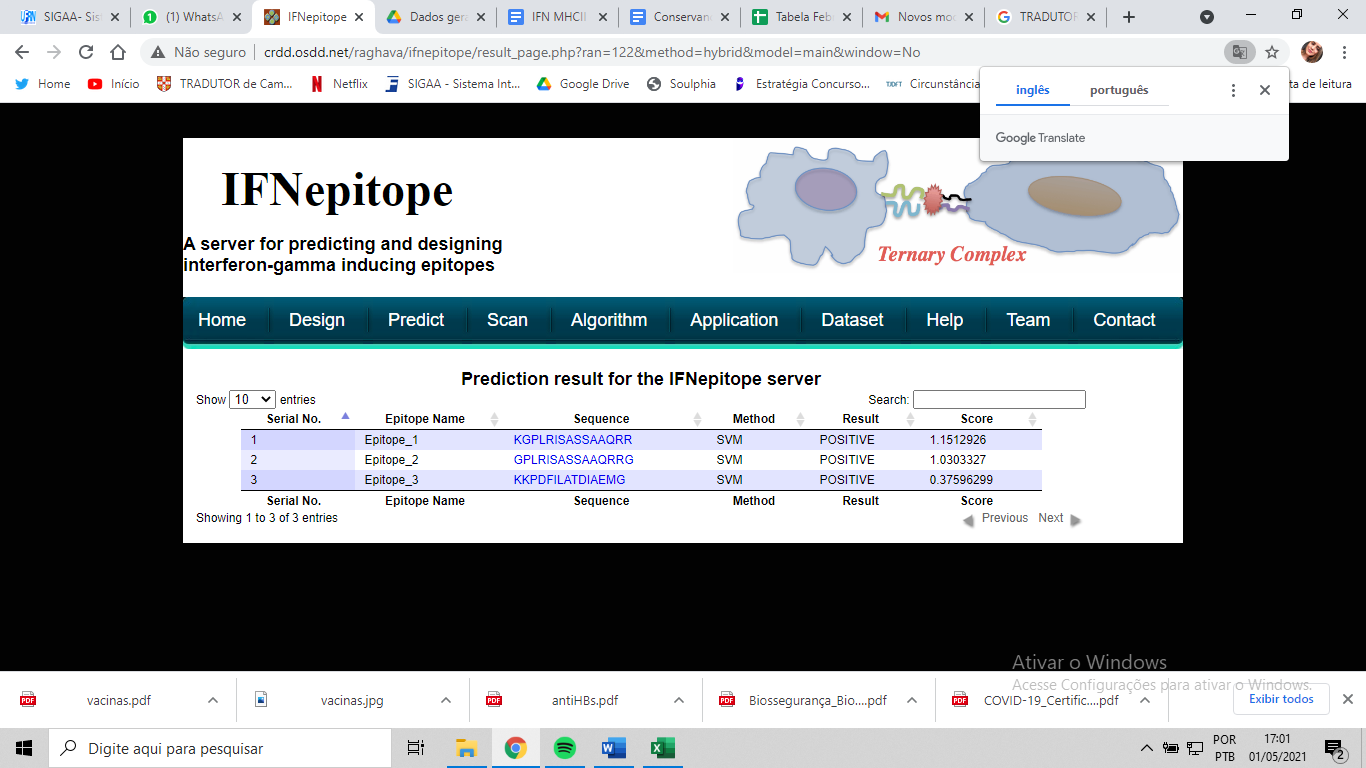


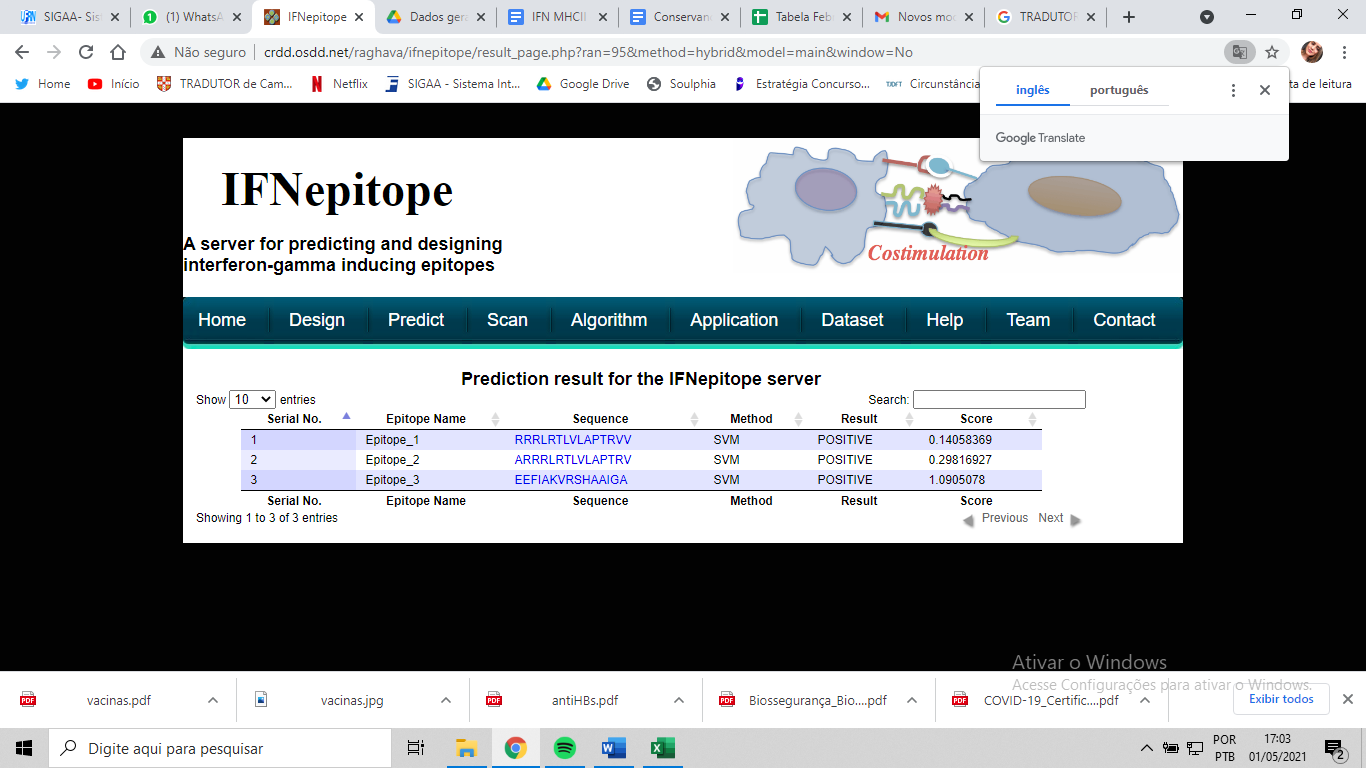

Supplement: Supplementary file 1 — Supplementary Information. [file 41598_2024_60680_MOESM1_ESM.zip › Yellow_Fever_data/2_Prediction of T-cell epitopes/IFN MHCII.docx]

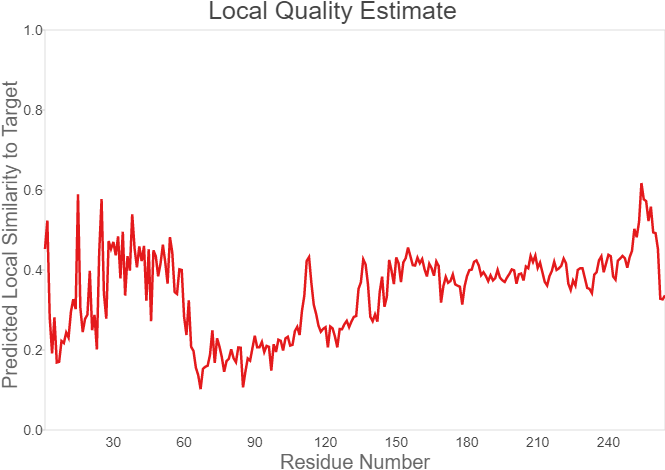

Supplement: Supplementary file 1 — Supplementary Information. [file 41598_2024_60680_MOESM1_ESM.zip › Yellow_Fever_data/4_Vaccine_validation/Local_quality_estimate.bmp]

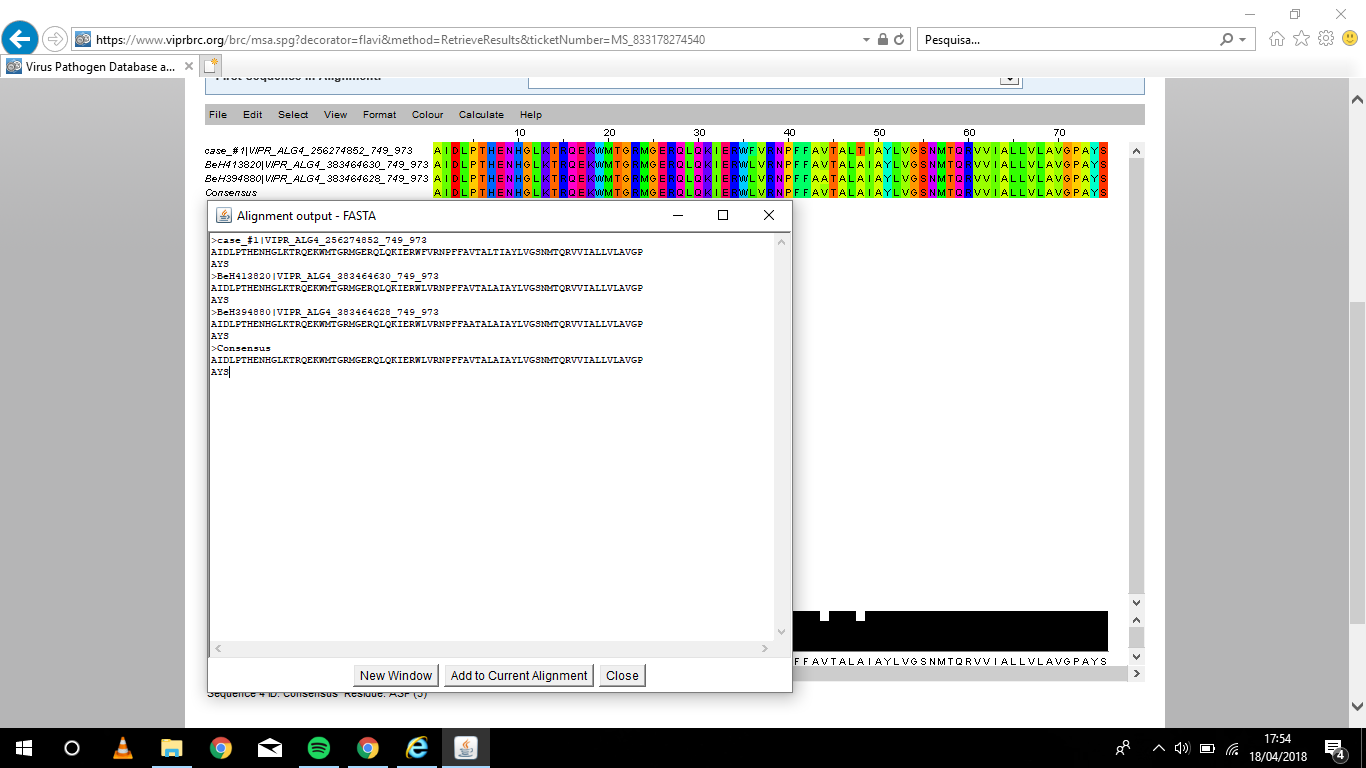

Supplement: Supplementary file 1 — Supplementary Information. [file 41598_2024_60680_MOESM1_ESM.zip › Yellow_Fever_data/1_Acquisition_proteins/Prints VIPR/M/passo 9.png]

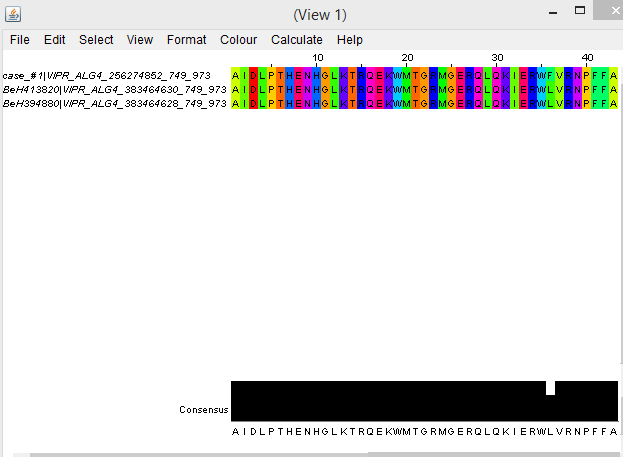

Supplement: Supplementary file 1 — Supplementary Information. [file 41598_2024_60680_MOESM1_ESM.zip › Yellow_Fever_data/1_Acquisition_proteins/Prints VIPR/M/passo 8.png]

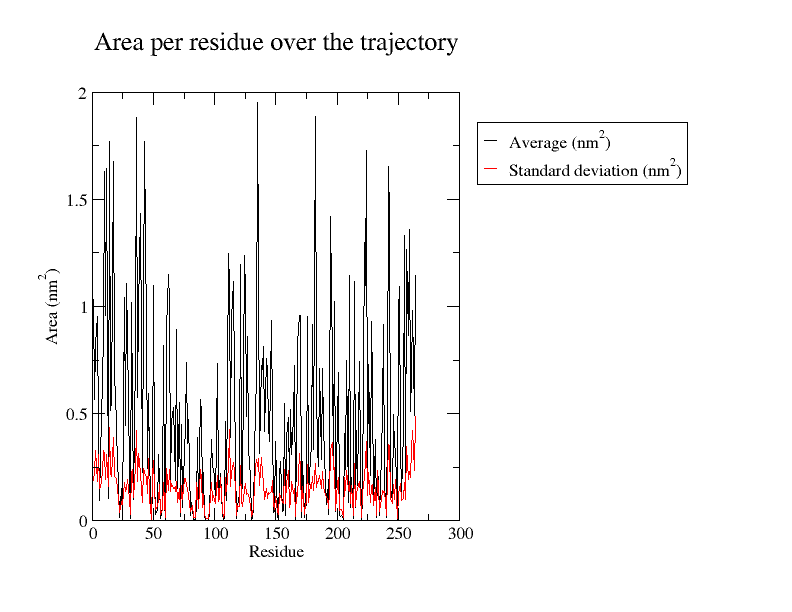

Supplement: Supplementary file 1 — Supplementary Information. [file 41598_2024_60680_MOESM1_ESM.zip › Yellow_Fever_data/7_Molecular Dynamics/SAS_resarea-clean-protein.png]

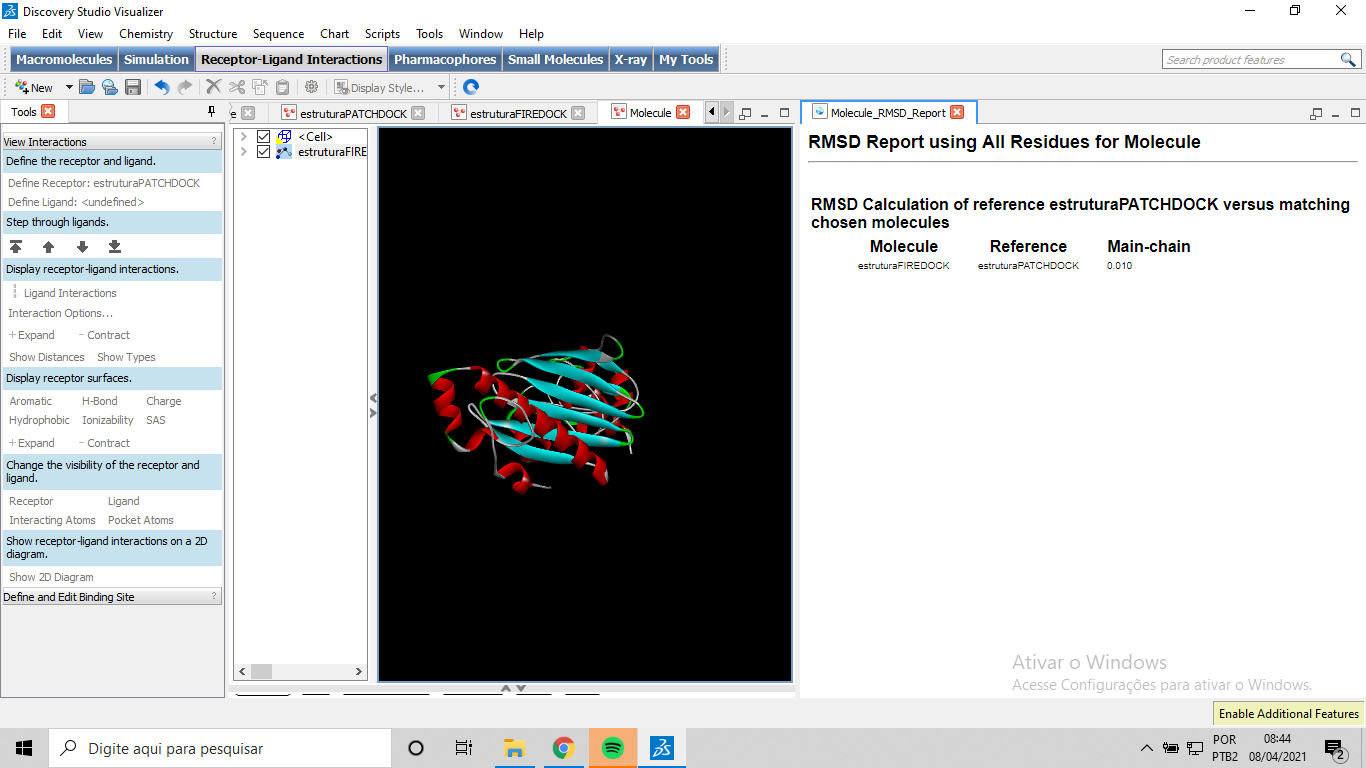


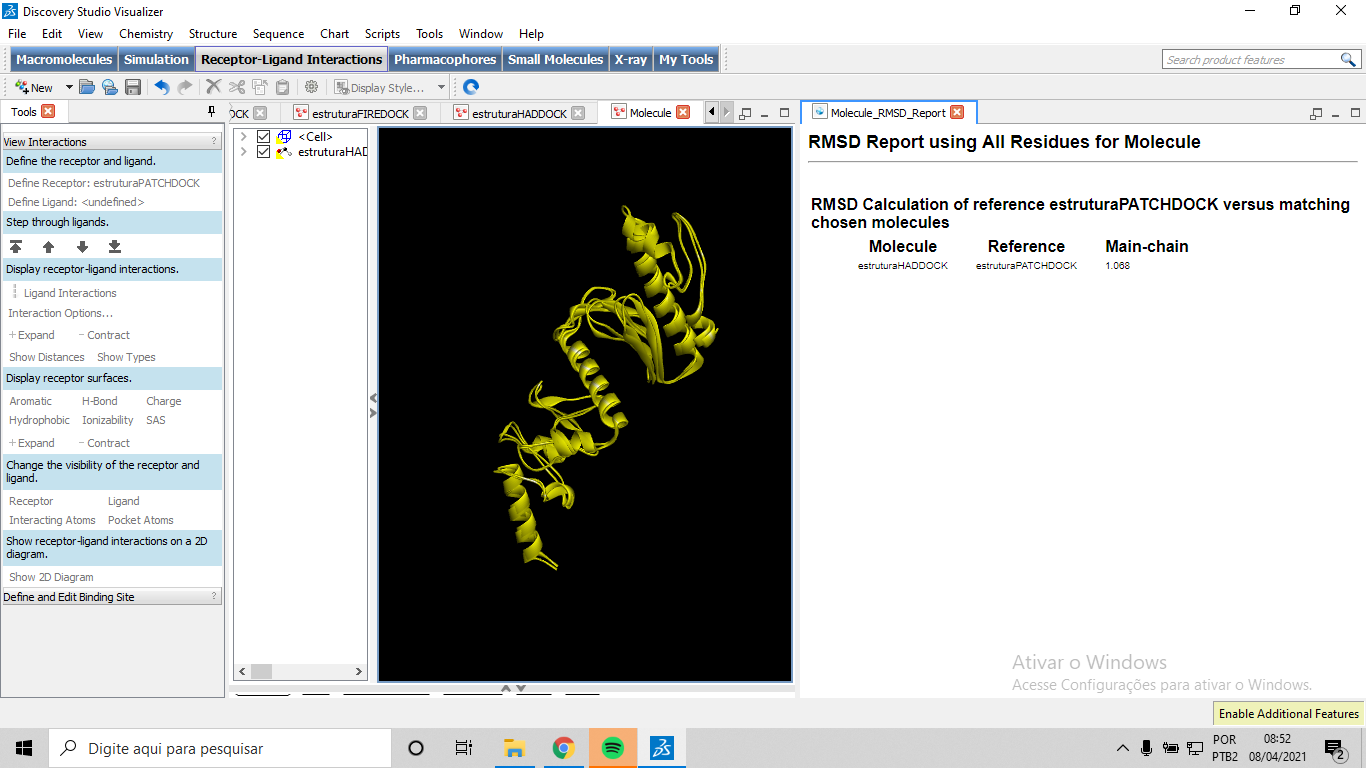

Supplement: Supplementary file 1 — Supplementary Information. [file 41598_2024_60680_MOESM1_ESM.zip › Yellow_Fever_data/5_Molecular docking/RMSD/Documento sem título.docx]

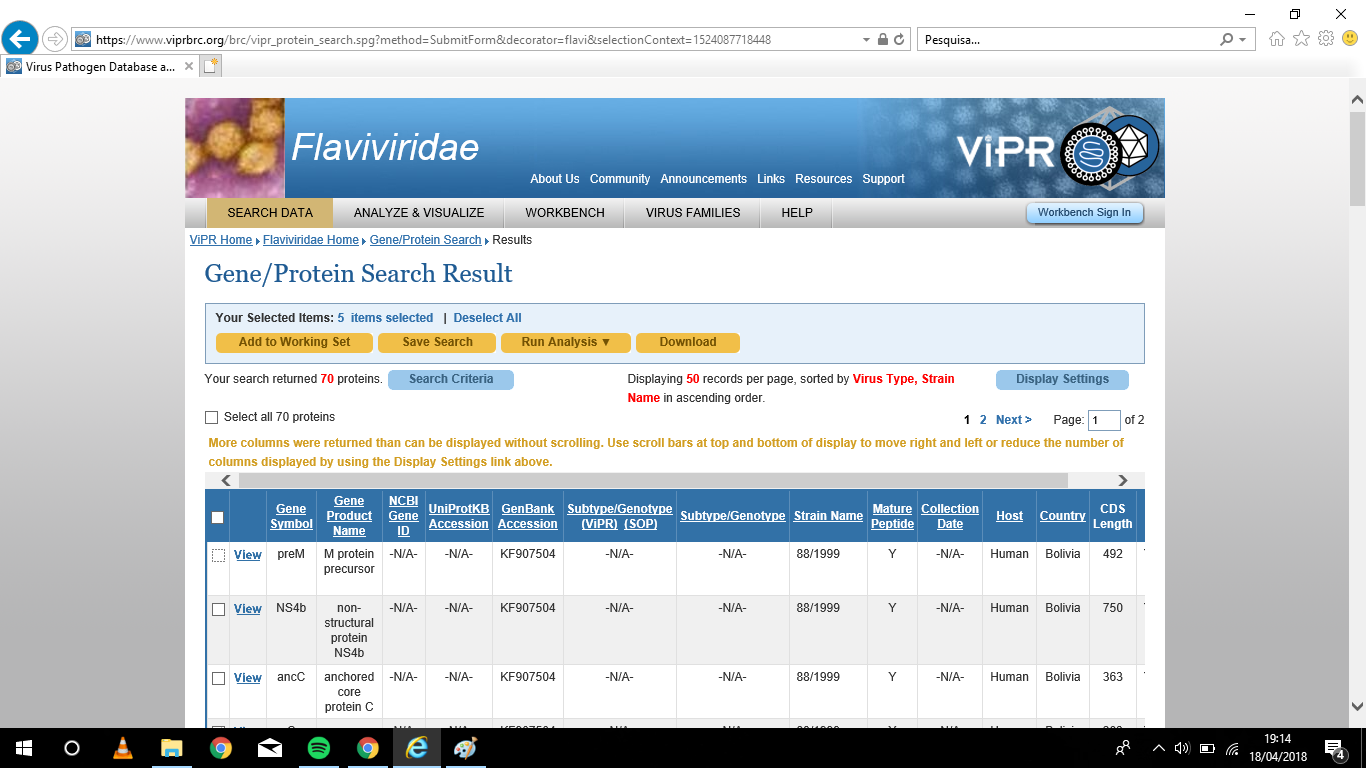

Supplement: Supplementary file 1 — Supplementary Information. [file 41598_2024_60680_MOESM1_ESM.zip › Yellow_Fever_data/1_Acquisition_proteins/Prints VIPR/C/passo 1 c.png]

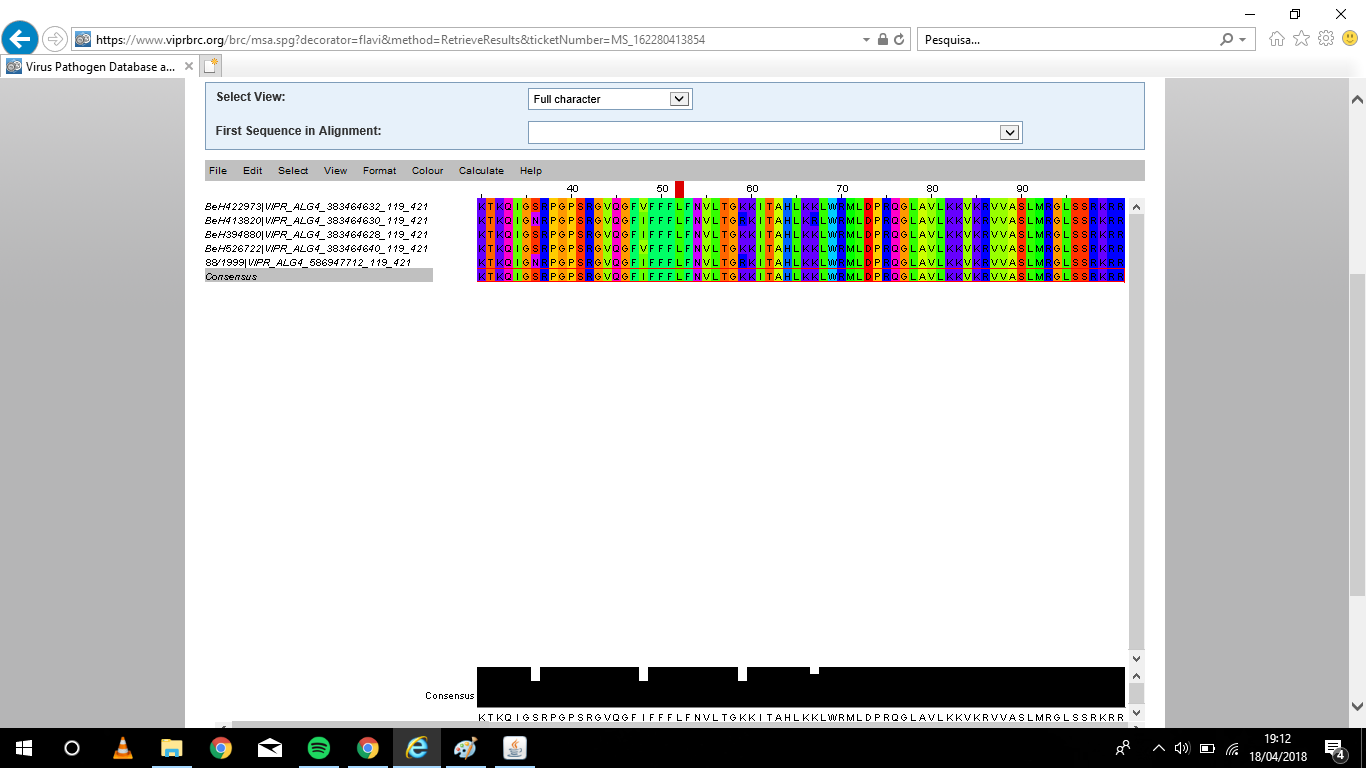

Supplement: Supplementary file 1 — Supplementary Information. [file 41598_2024_60680_MOESM1_ESM.zip › Yellow_Fever_data/1_Acquisition_proteins/Prints VIPR/C/passo 2 c.png]

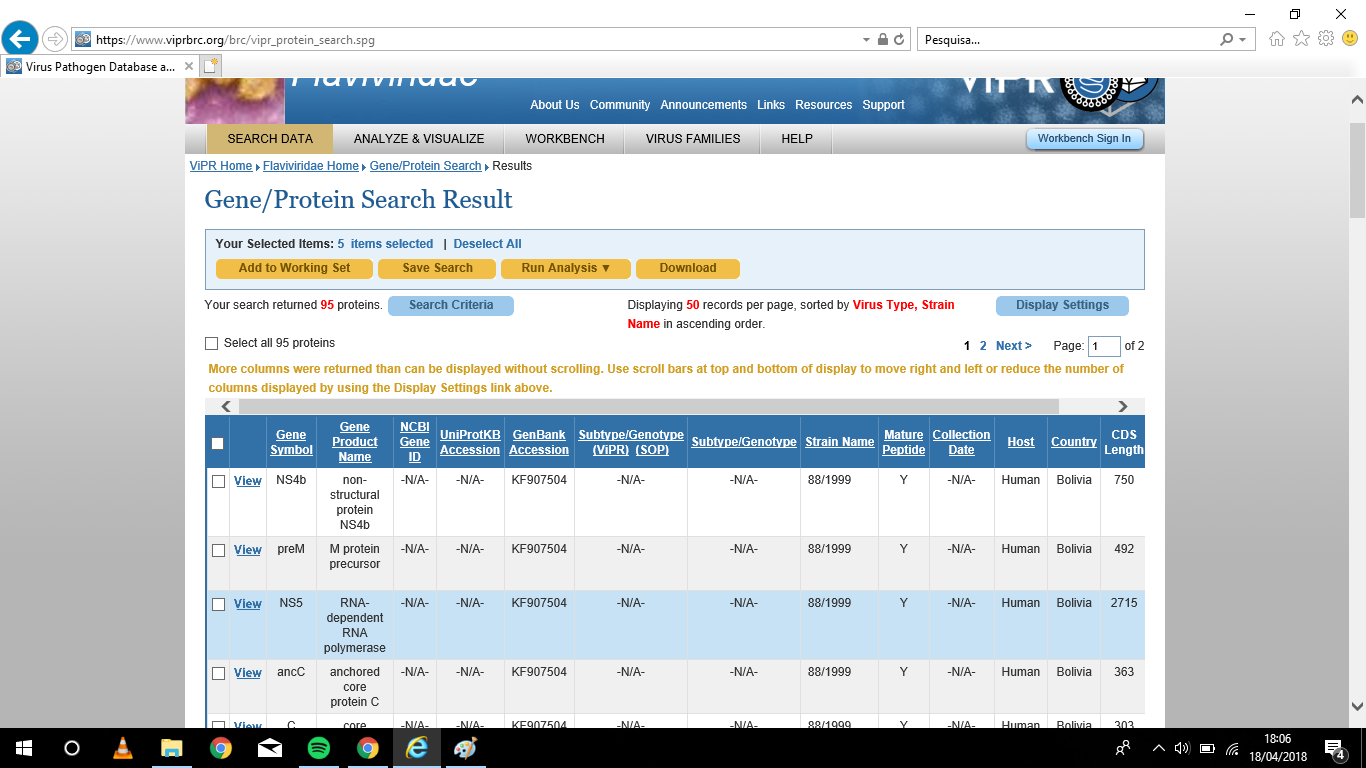

Supplement: Supplementary file 1 — Supplementary Information. [file 41598_2024_60680_MOESM1_ESM.zip › Yellow_Fever_data/1_Acquisition_proteins/Prints VIPR/E/paaso 1 e.png]

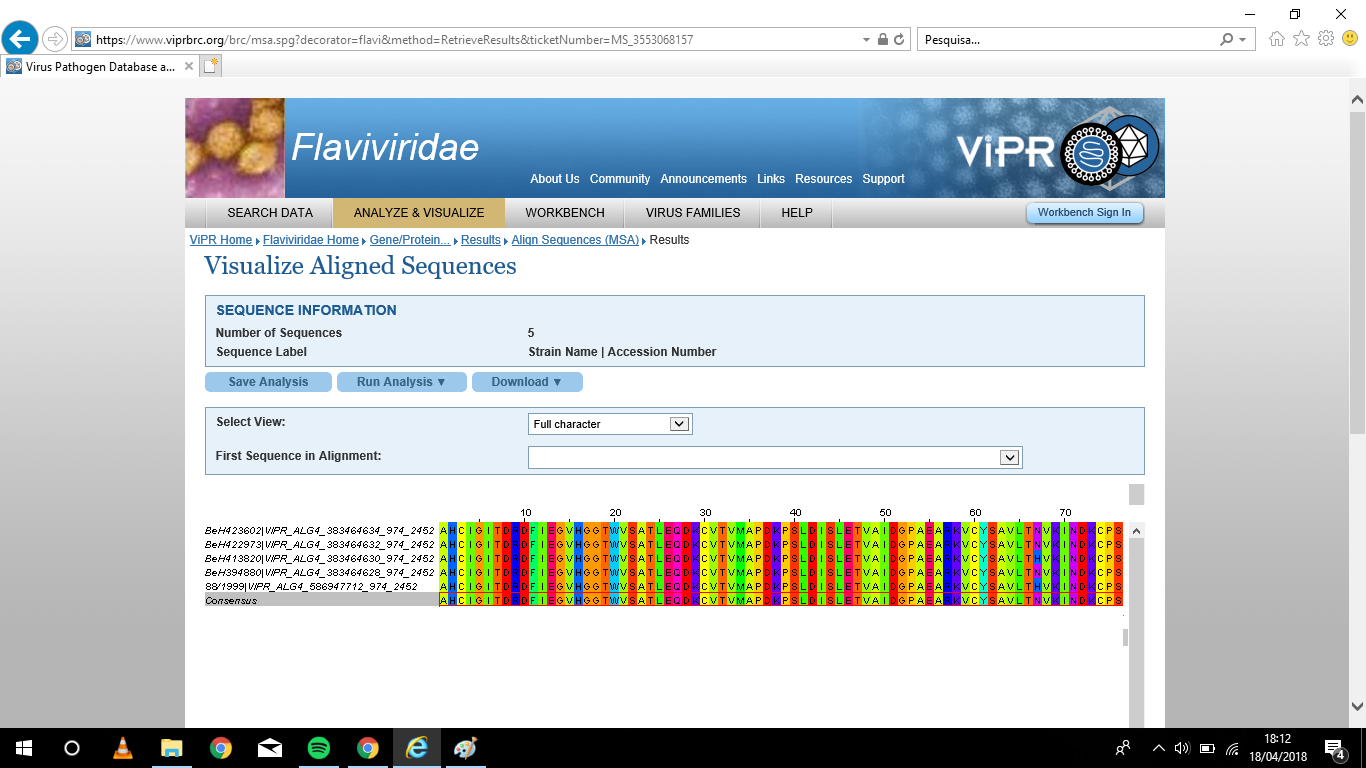

Supplement: Supplementary file 1 — Supplementary Information. [file 41598_2024_60680_MOESM1_ESM.zip › Yellow_Fever_data/1_Acquisition_proteins/Prints VIPR/E/passo 2 e.png]

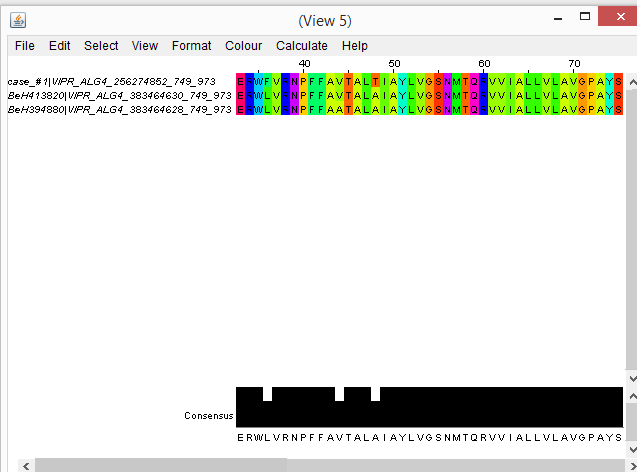

Supplement: Supplementary file 1 — Supplementary Information. [file 41598_2024_60680_MOESM1_ESM.zip › Yellow_Fever_data/1_Acquisition_proteins/Prints VIPR/M/passo 8.2.png]

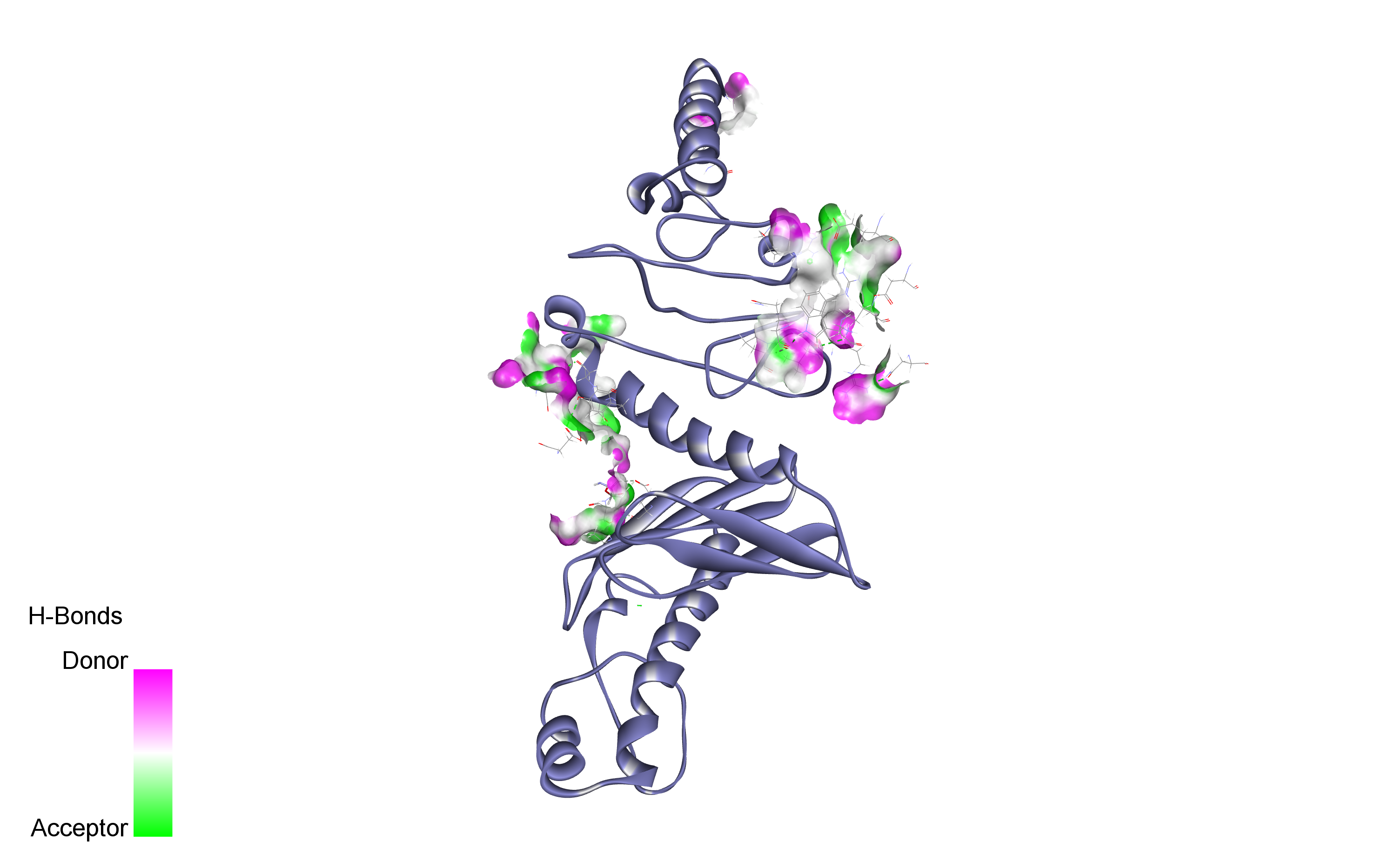

Supplement: Supplementary file 1 — Supplementary Information. [file 41598_2024_60680_MOESM1_ESM.zip › Yellow_Fever_data/6_Intermolecular_interactions/estruturalHADDOCK_YV.bmp]

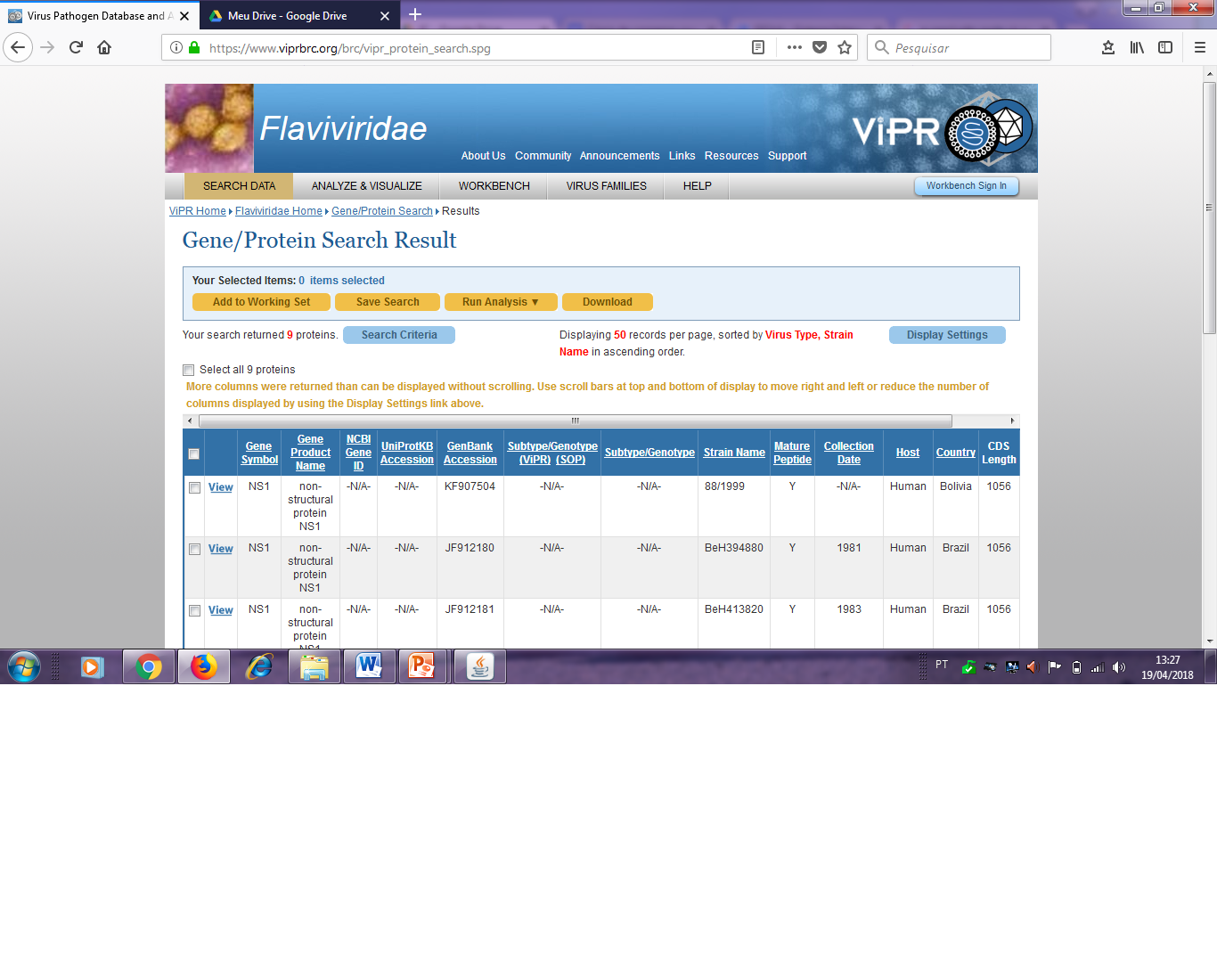

Supplement: Supplementary file 1 — Supplementary Information. [file 41598_2024_60680_MOESM1_ESM.zip › Yellow_Fever_data/1_Acquisition_proteins/Prints VIPR/ns1/passo 1 ns1.png]

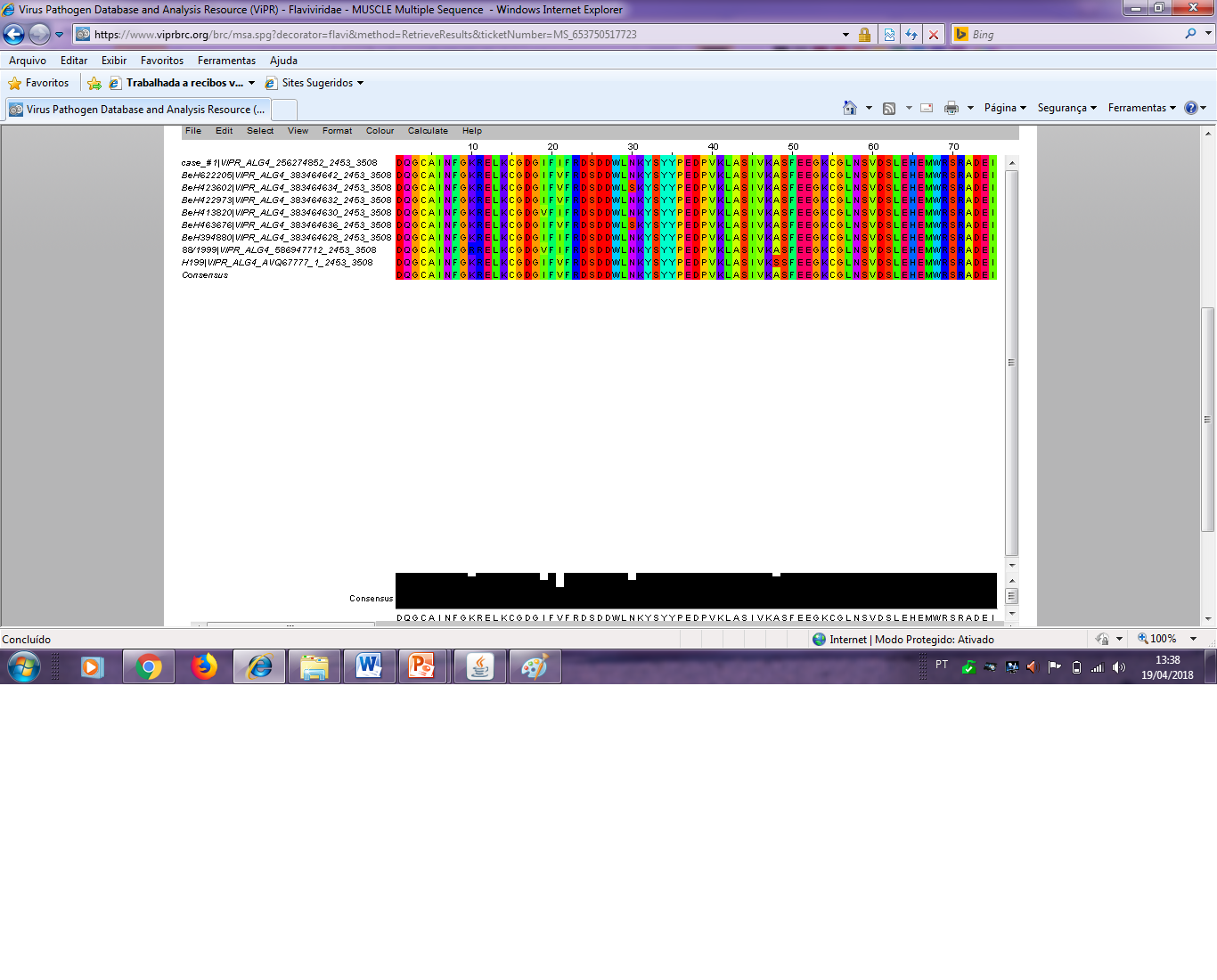

Supplement: Supplementary file 1 — Supplementary Information. [file 41598_2024_60680_MOESM1_ESM.zip › Yellow_Fever_data/1_Acquisition_proteins/Prints VIPR/ns1/passo 2 ns1.png]

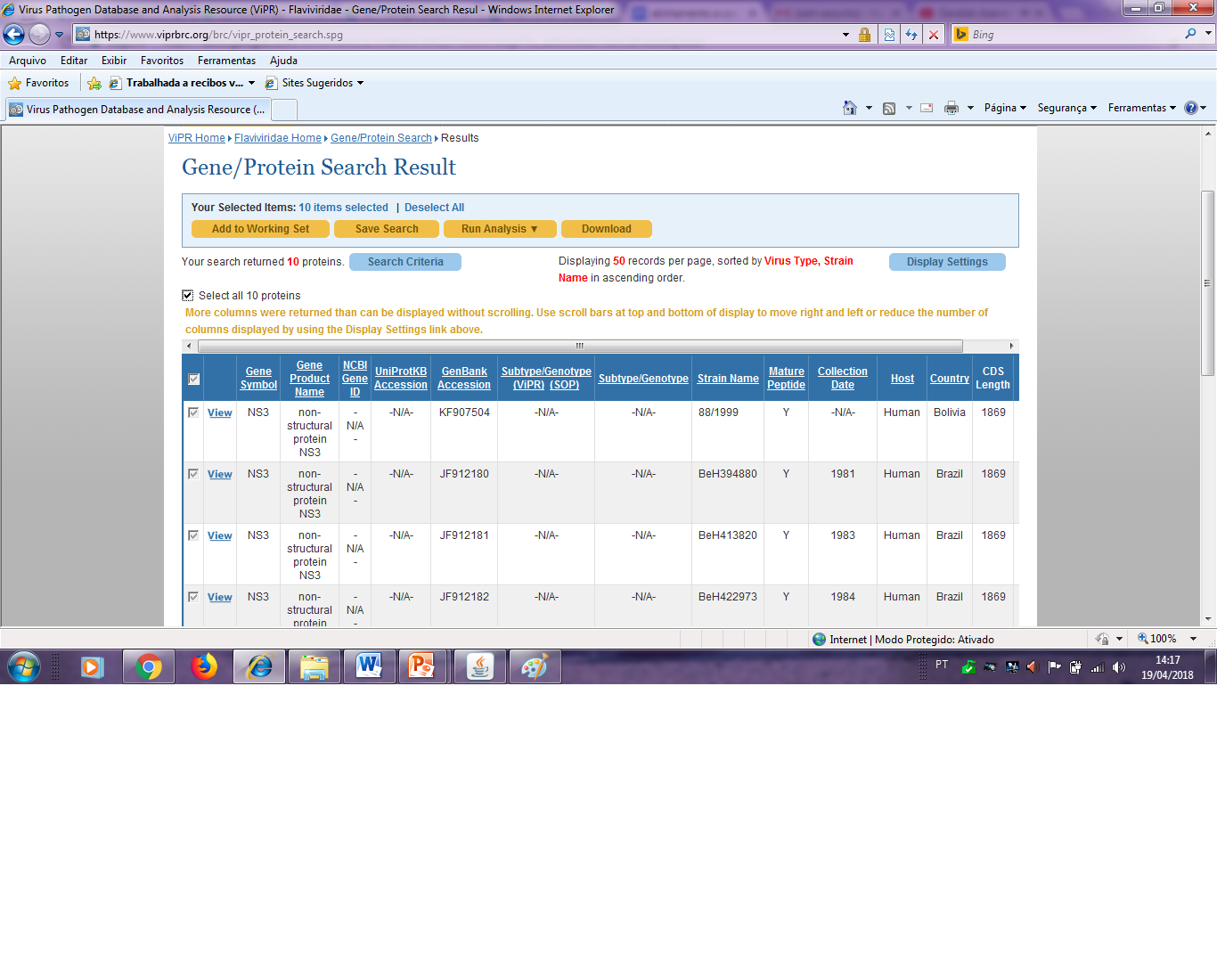

Supplement: Supplementary file 1 — Supplementary Information. [file 41598_2024_60680_MOESM1_ESM.zip › Yellow_Fever_data/1_Acquisition_proteins/Prints VIPR/ns3/passo 1 ns3.png]

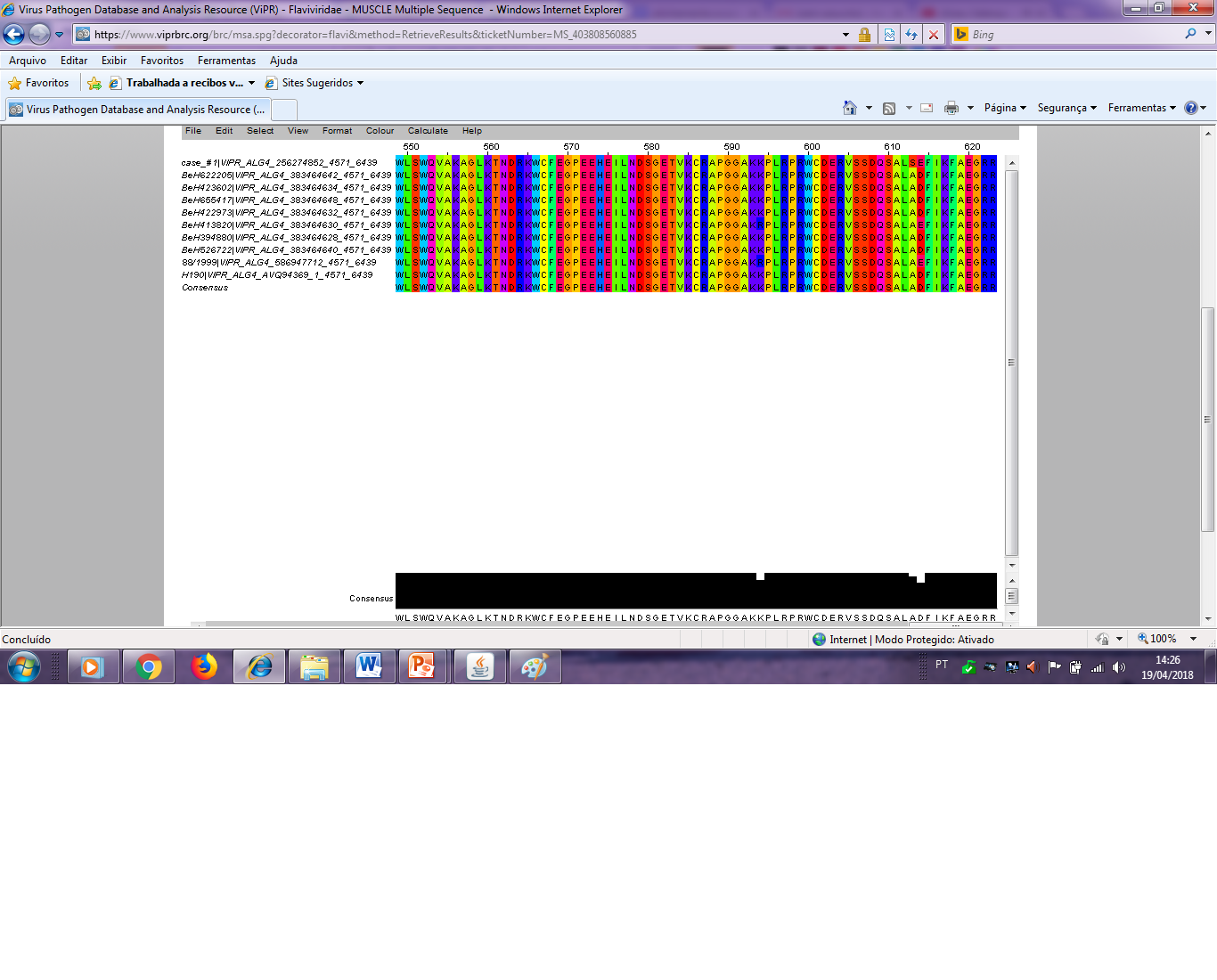

Supplement: Supplementary file 1 — Supplementary Information. [file 41598_2024_60680_MOESM1_ESM.zip › Yellow_Fever_data/1_Acquisition_proteins/Prints VIPR/ns3/passo 2 ns3.png]

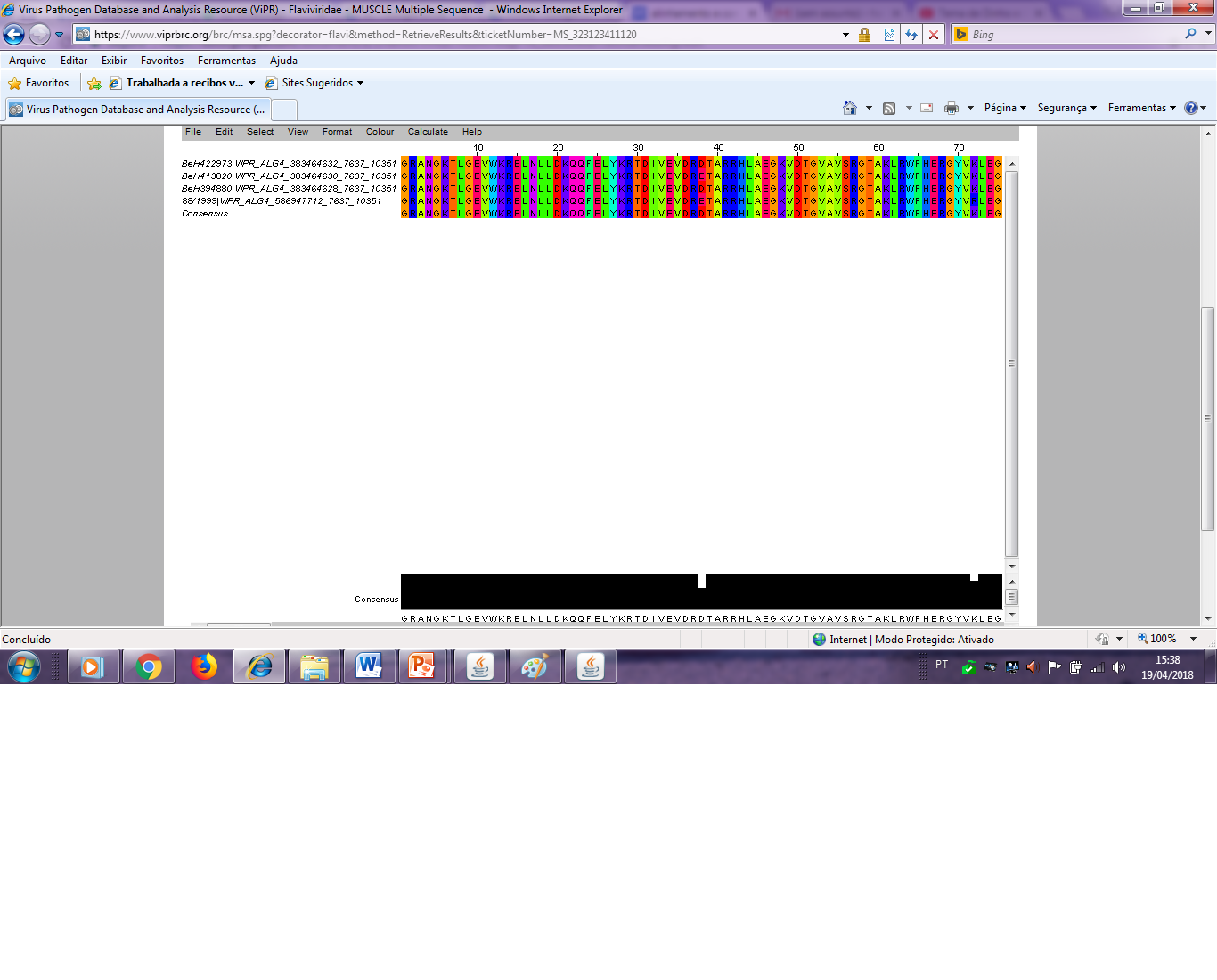

Supplement: Supplementary file 1 — Supplementary Information. [file 41598_2024_60680_MOESM1_ESM.zip › Yellow_Fever_data/1_Acquisition_proteins/Prints VIPR/ns5/passo 2 ns5.png]

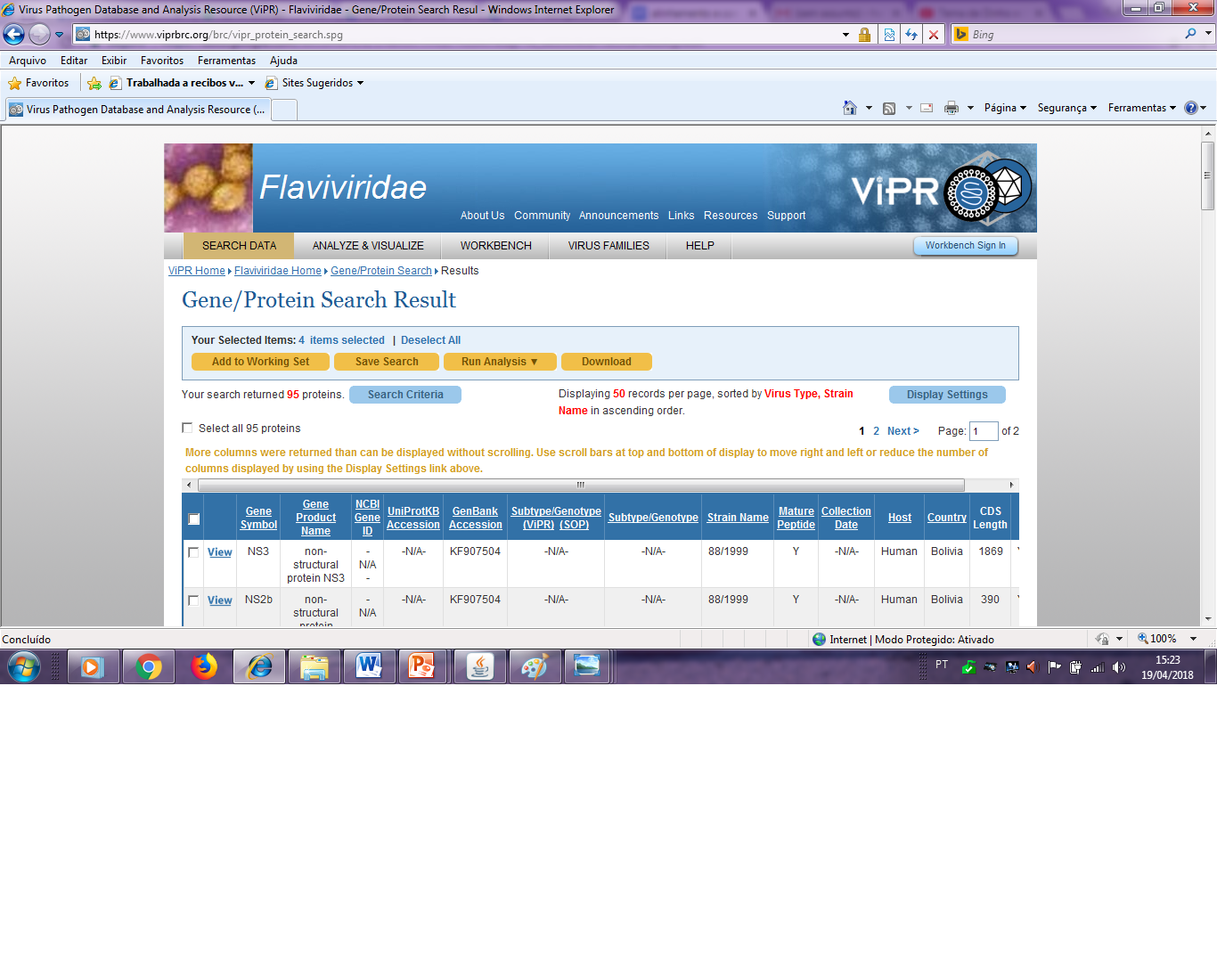

Supplement: Supplementary file 1 — Supplementary Information. [file 41598_2024_60680_MOESM1_ESM.zip › Yellow_Fever_data/1_Acquisition_proteins/Prints VIPR/ns5/passo 1 ns5.png]

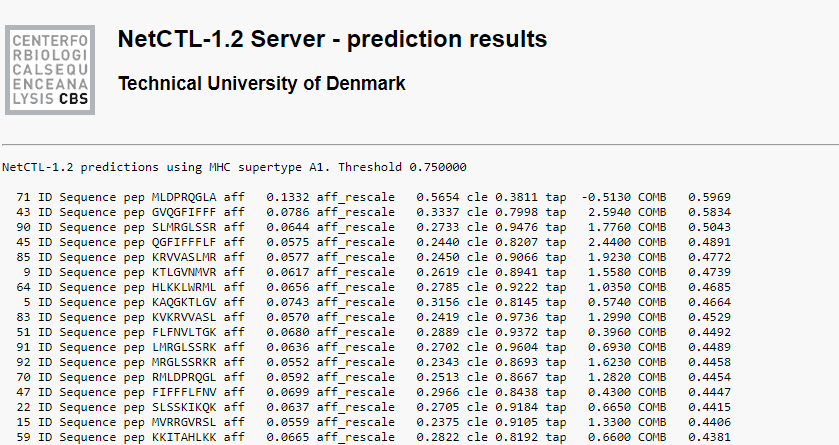

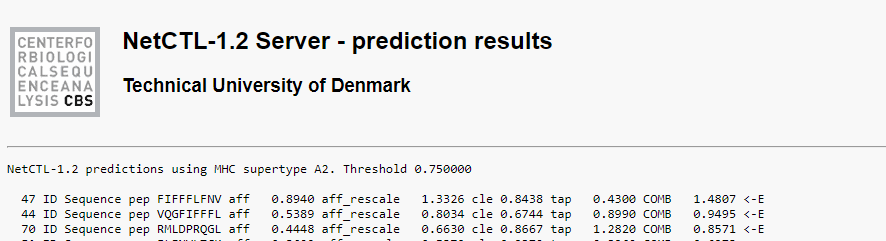

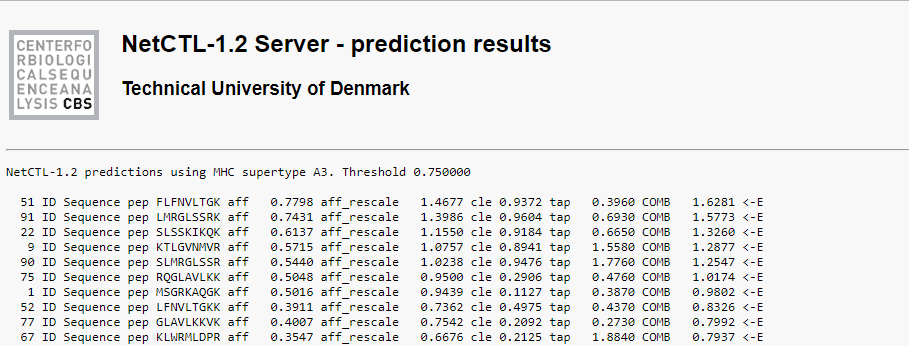

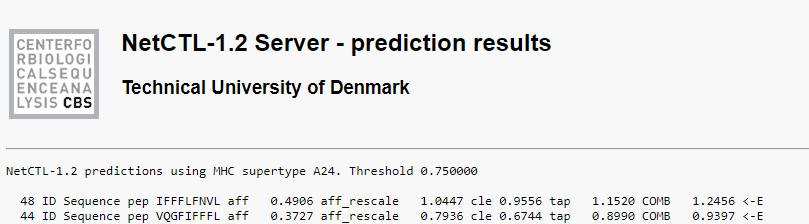

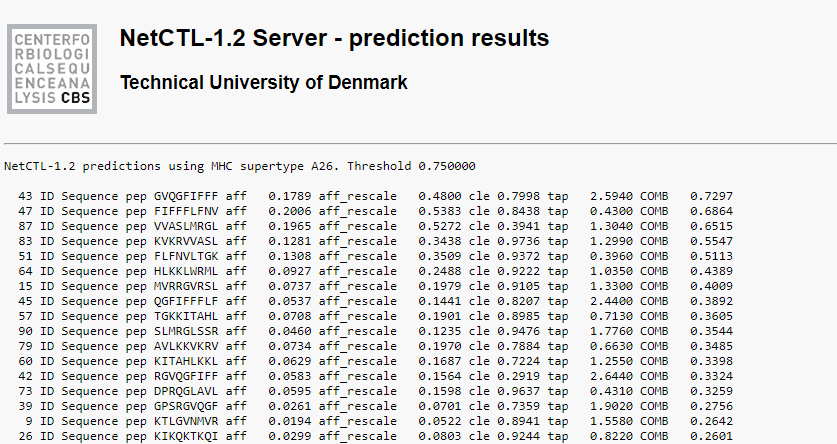

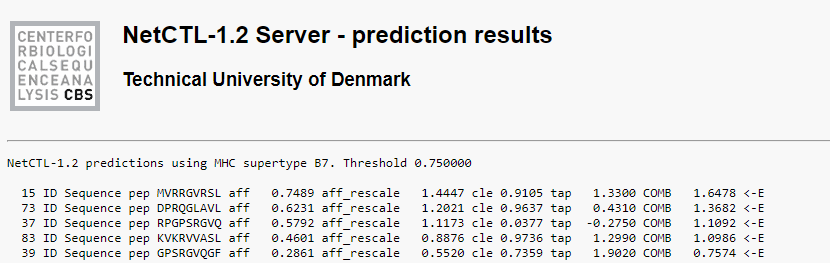

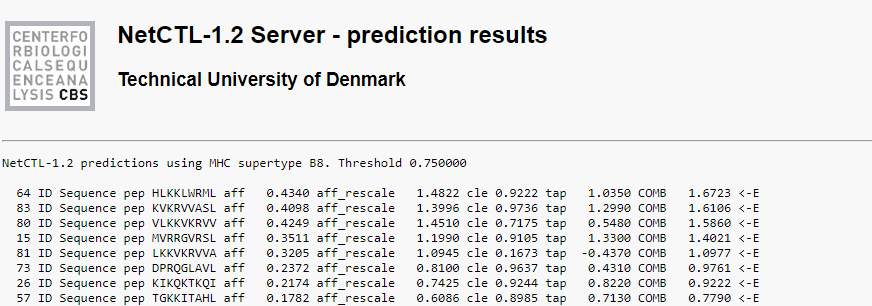

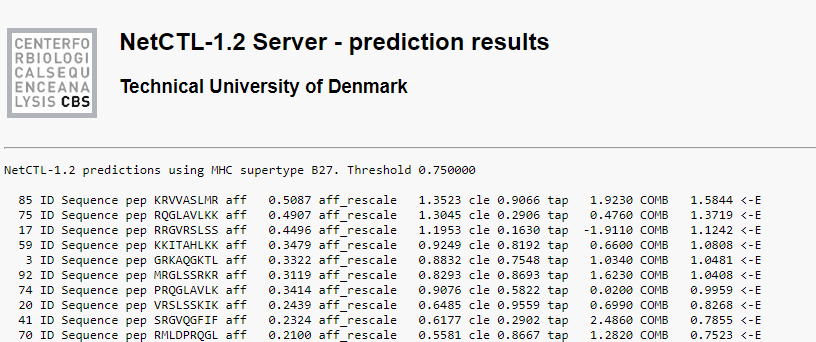

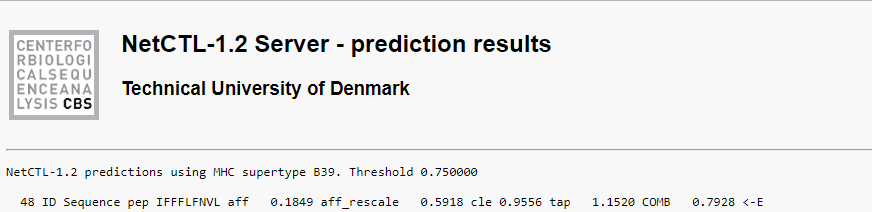

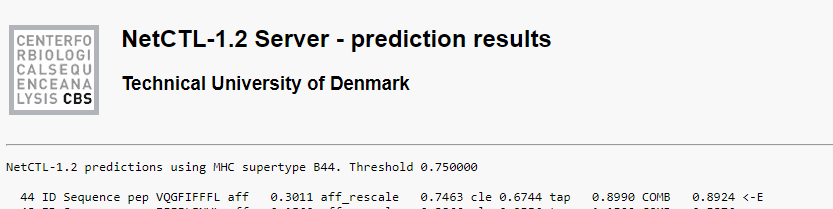

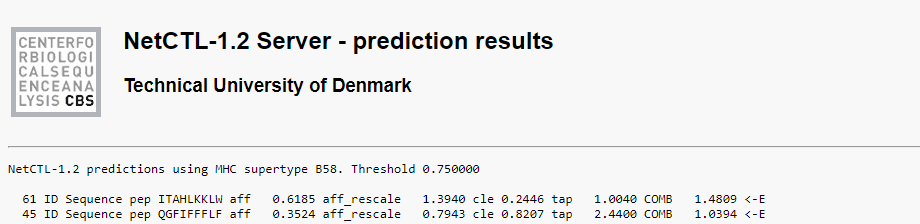

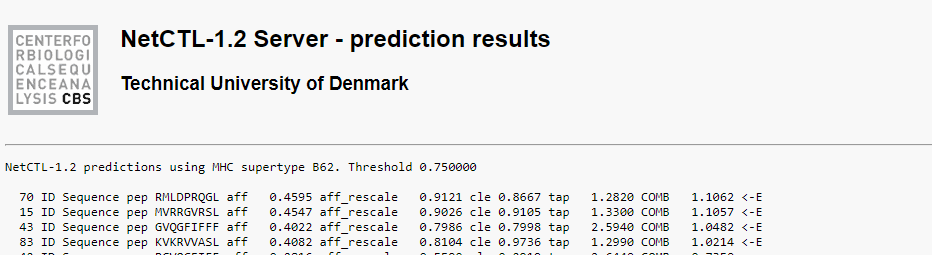

Supplement: Supplementary file 1 — Supplementary Information. [file 41598_2024_60680_MOESM1_ESM.zip › Yellow_Fever_data/2_Prediction of T-cell epitopes/NetCTL/C/NetCTL-C.docx]

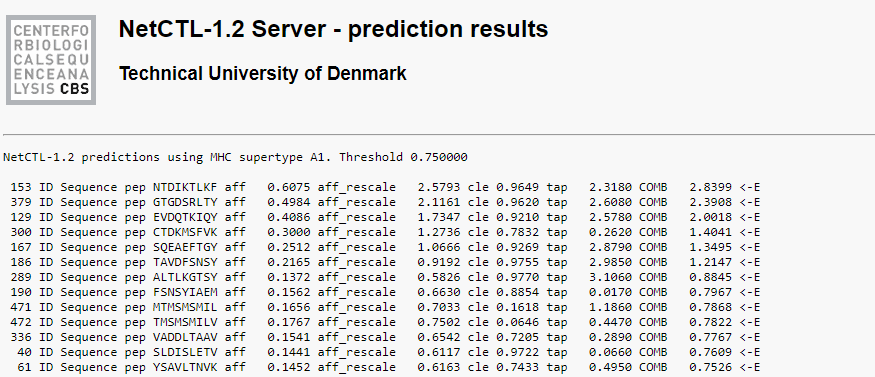

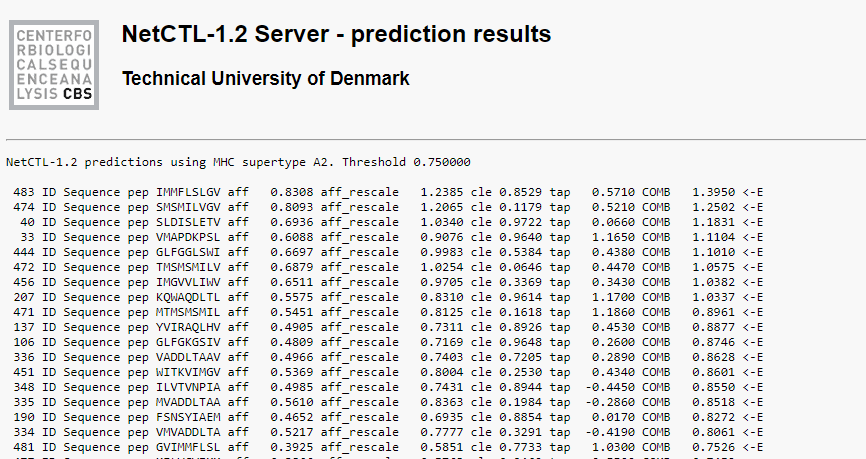

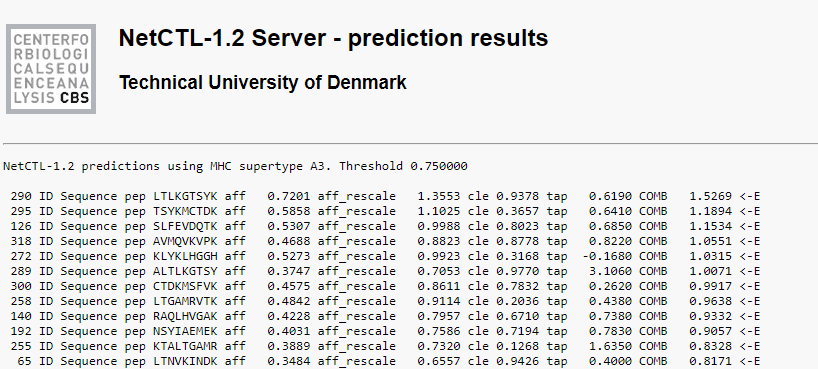

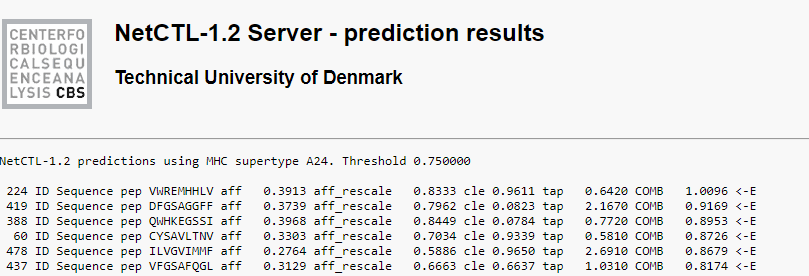

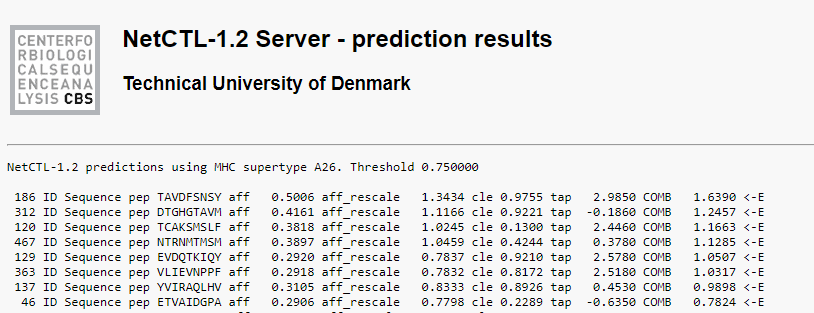

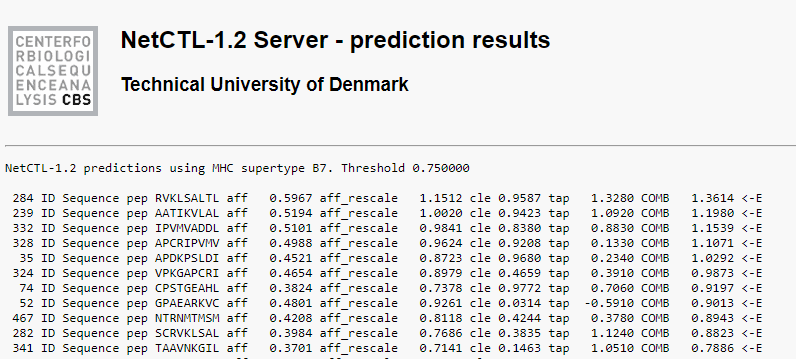

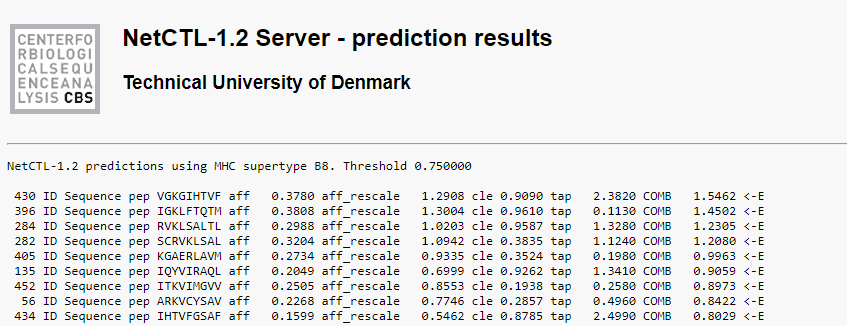

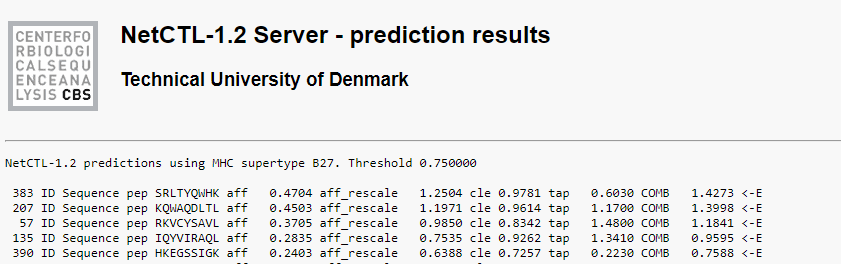

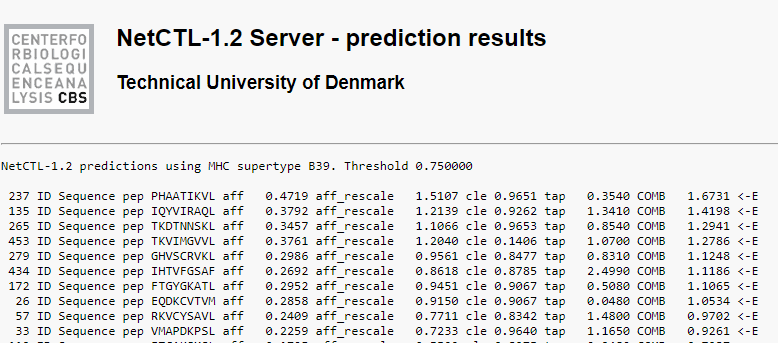

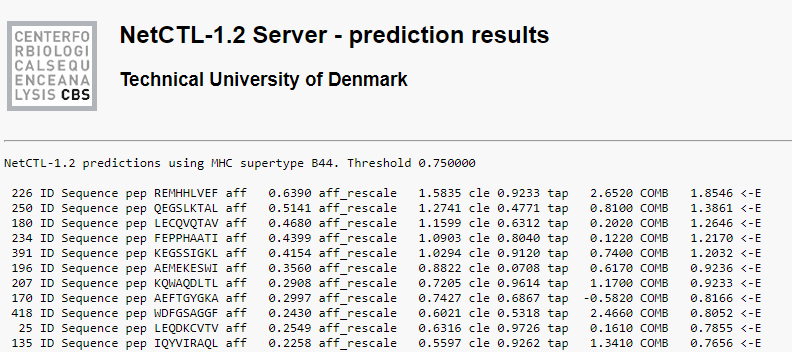

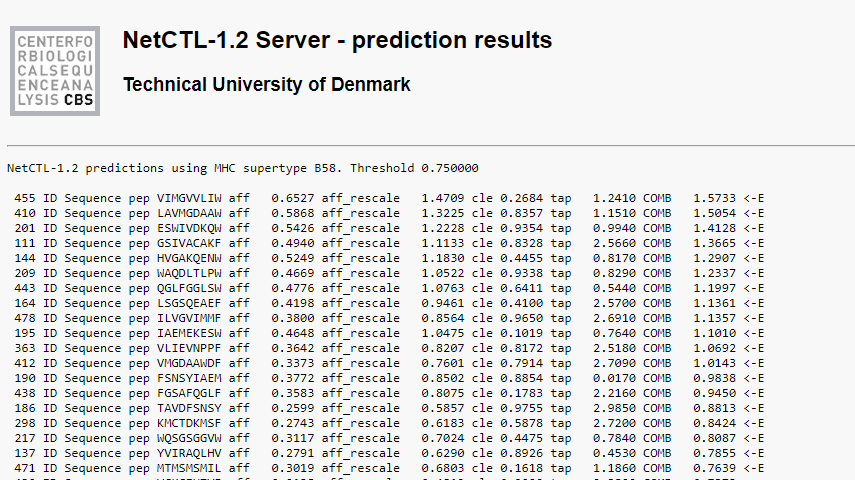

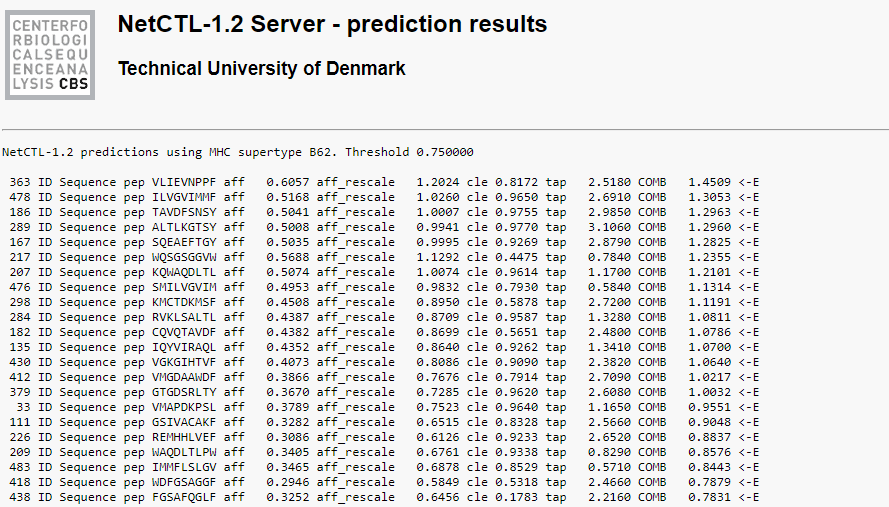

Supplement: Supplementary file 1 — Supplementary Information. [file 41598_2024_60680_MOESM1_ESM.zip › Yellow_Fever_data/2_Prediction of T-cell epitopes/NetCTL/E/NetCTL- E.docx]

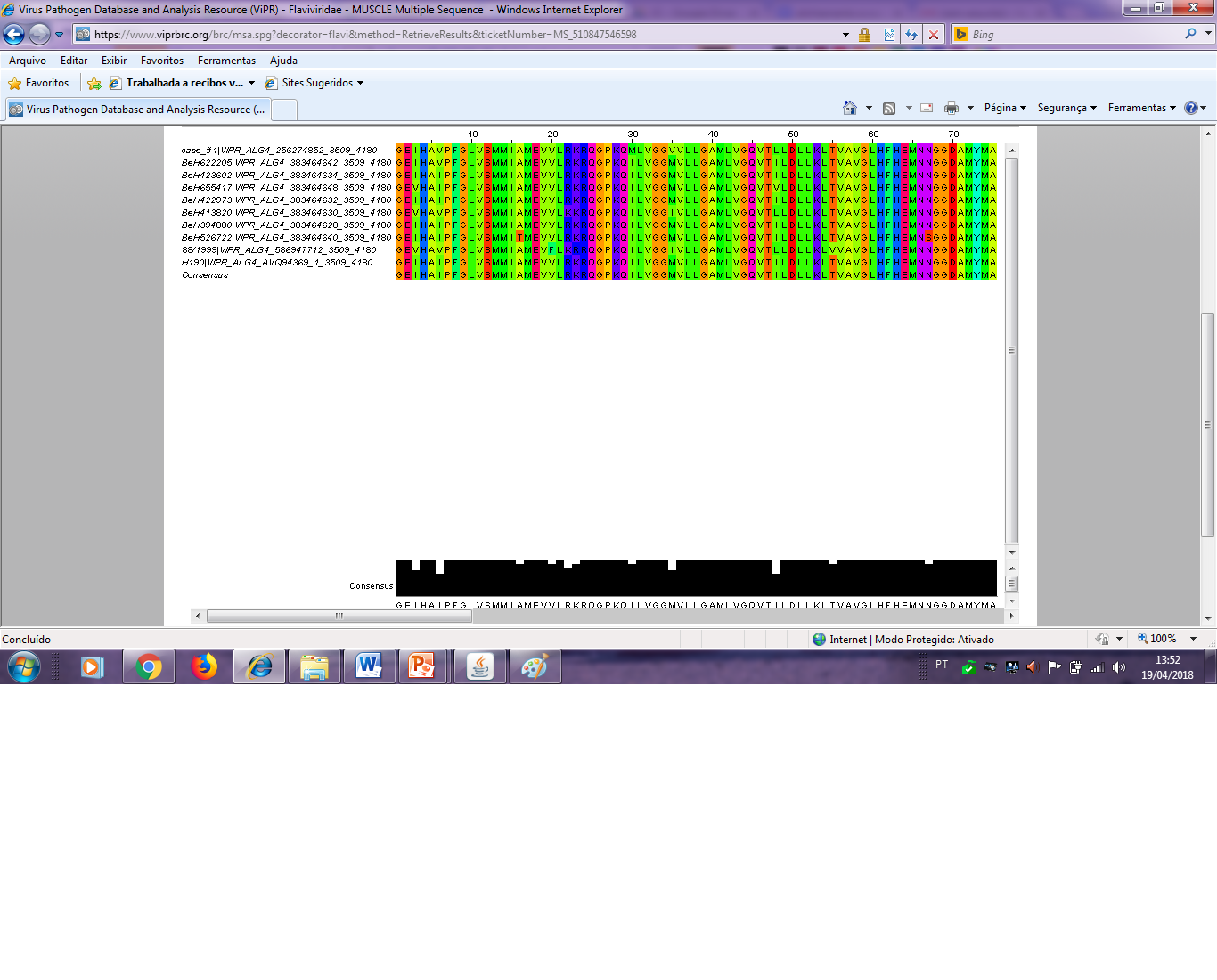

Supplement: Supplementary file 1 — Supplementary Information. [file 41598_2024_60680_MOESM1_ESM.zip › Yellow_Fever_data/1_Acquisition_proteins/Prints VIPR/ns2a/passo 2 ns2a.png]

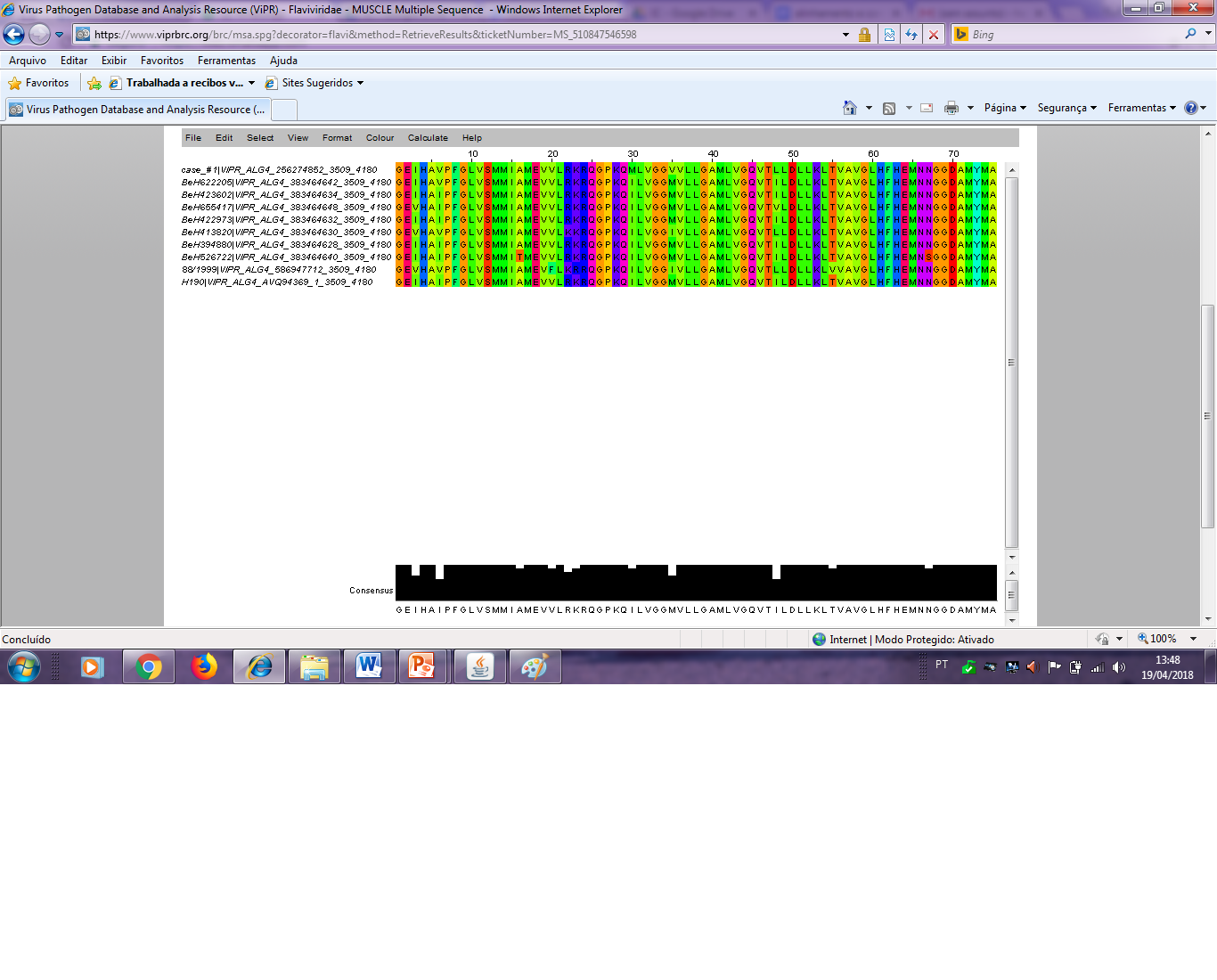

Supplement: Supplementary file 1 — Supplementary Information. [file 41598_2024_60680_MOESM1_ESM.zip › Yellow_Fever_data/1_Acquisition_proteins/Prints VIPR/ns2a/passo 3 ns2a.png]

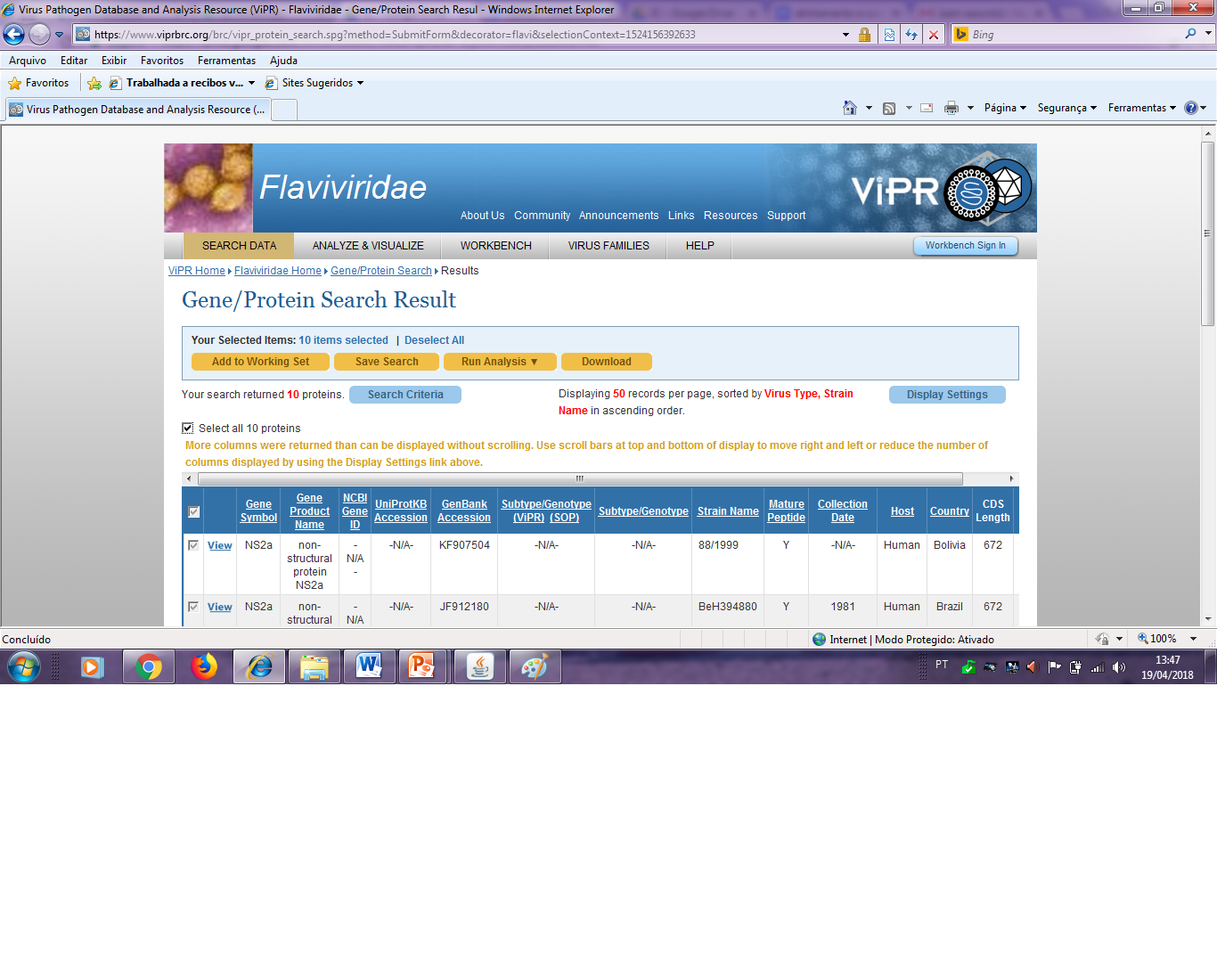

Supplement: Supplementary file 1 — Supplementary Information. [file 41598_2024_60680_MOESM1_ESM.zip › Yellow_Fever_data/1_Acquisition_proteins/Prints VIPR/ns2a/passo 1 ns2a.png]

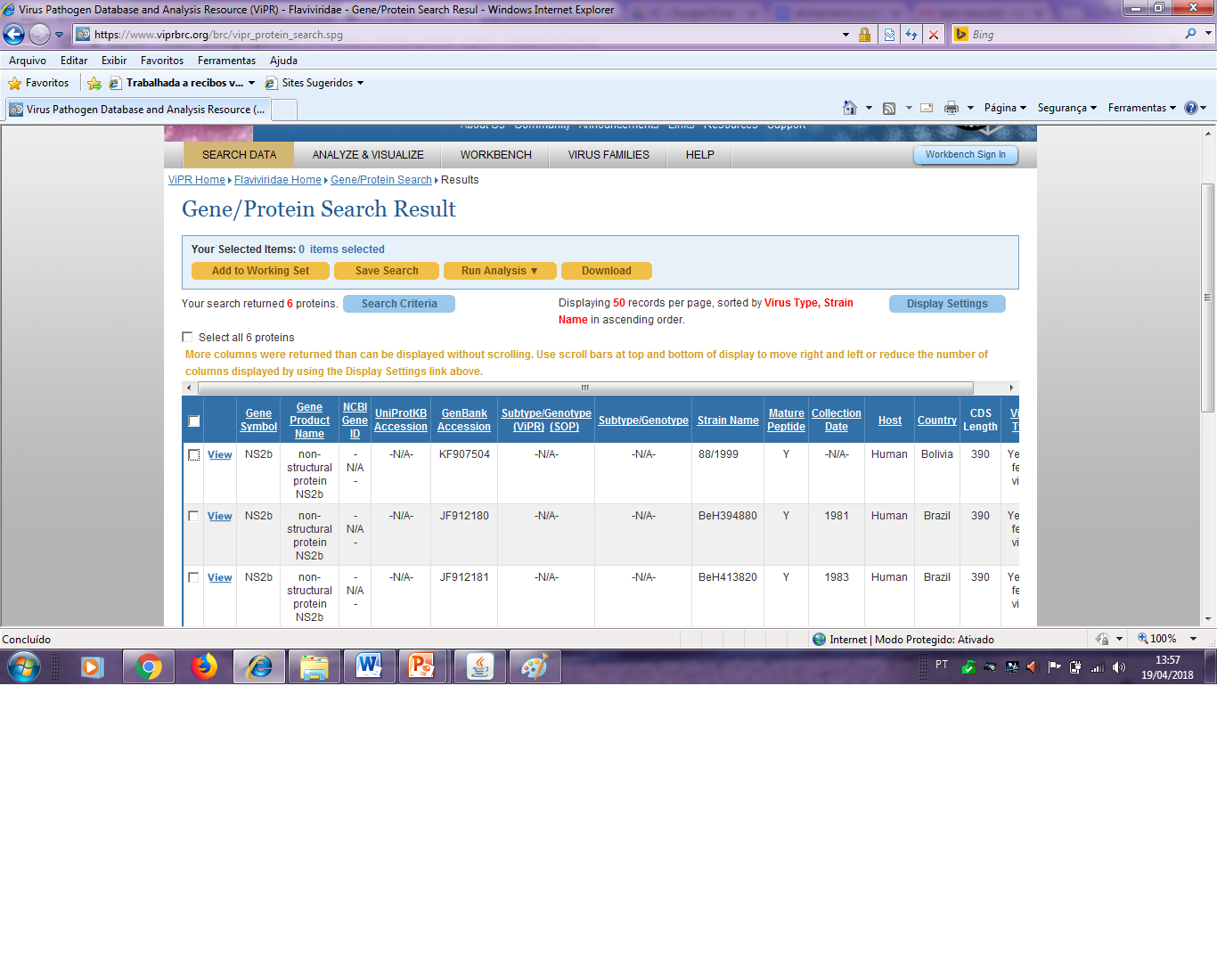

Supplement: Supplementary file 1 — Supplementary Information. [file 41598_2024_60680_MOESM1_ESM.zip › Yellow_Fever_data/1_Acquisition_proteins/Prints VIPR/ns2b/passo 1 ns2b.png]

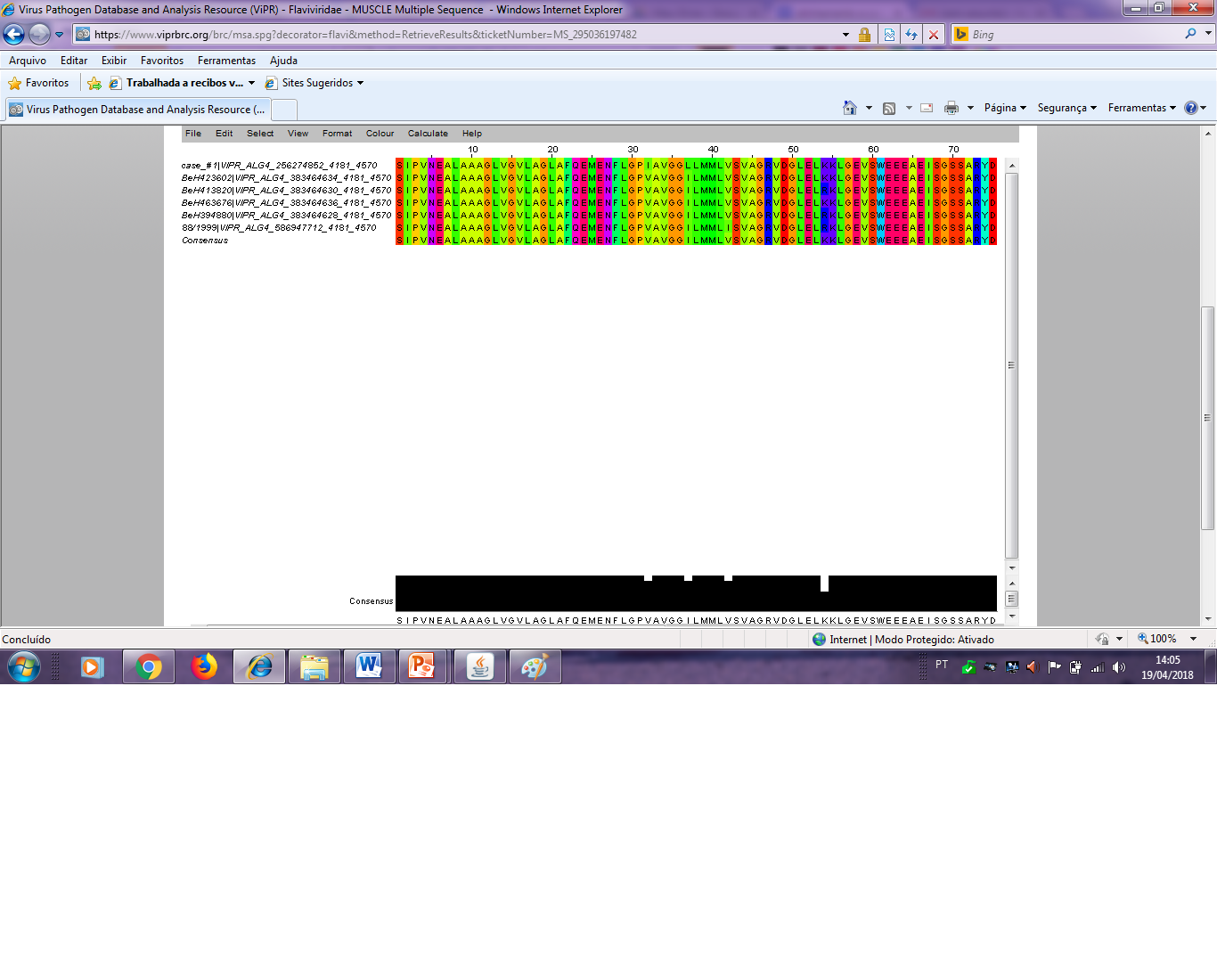

Supplement: Supplementary file 1 — Supplementary Information. [file 41598_2024_60680_MOESM1_ESM.zip › Yellow_Fever_data/1_Acquisition_proteins/Prints VIPR/ns2b/passo 2 ns2b.png]

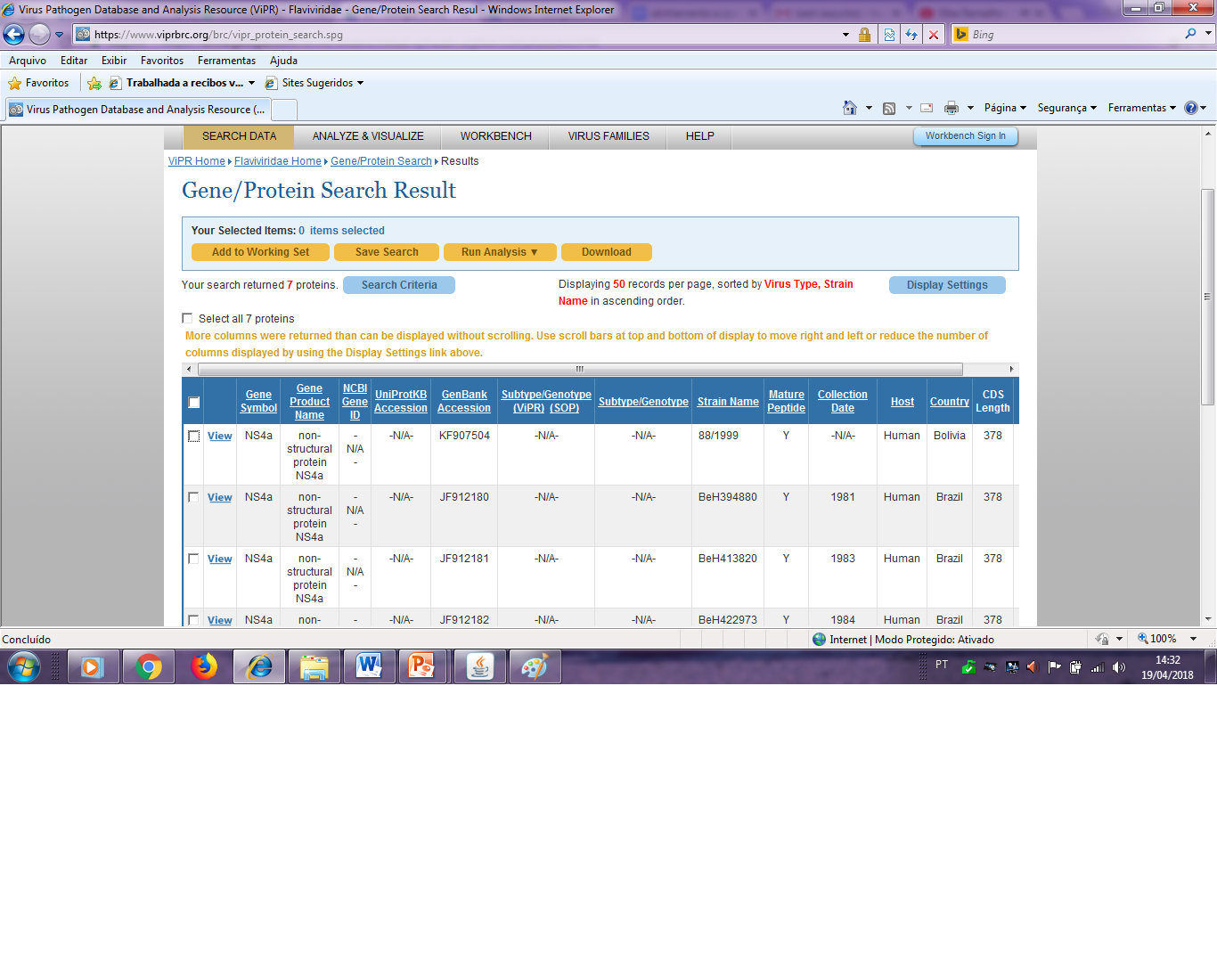

Supplement: Supplementary file 1 — Supplementary Information. [file 41598_2024_60680_MOESM1_ESM.zip › Yellow_Fever_data/1_Acquisition_proteins/Prints VIPR/ns4a/passo 1 ns4a.png]

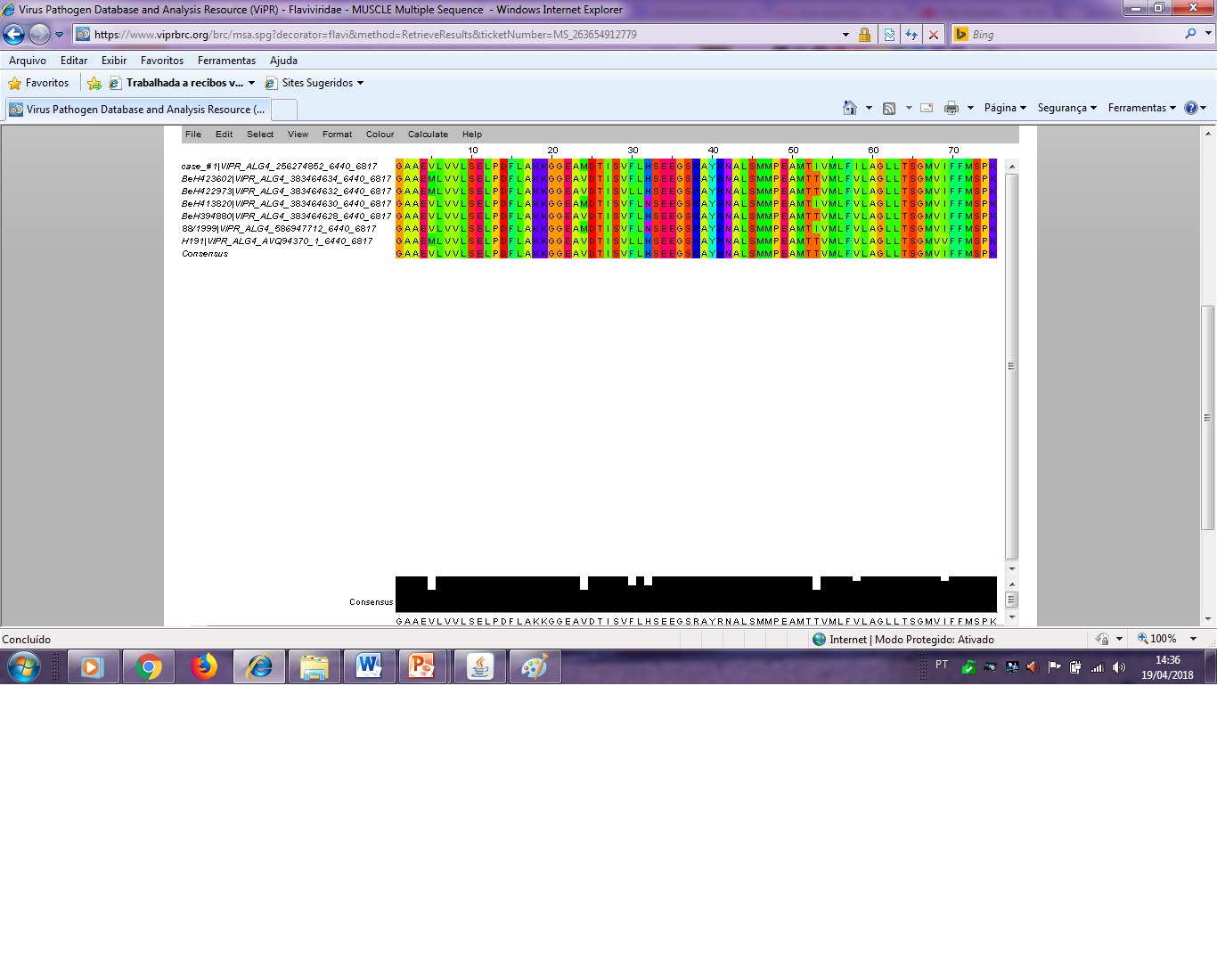

Supplement: Supplementary file 1 — Supplementary Information. [file 41598_2024_60680_MOESM1_ESM.zip › Yellow_Fever_data/1_Acquisition_proteins/Prints VIPR/ns4a/passo 2 ns4a.png]

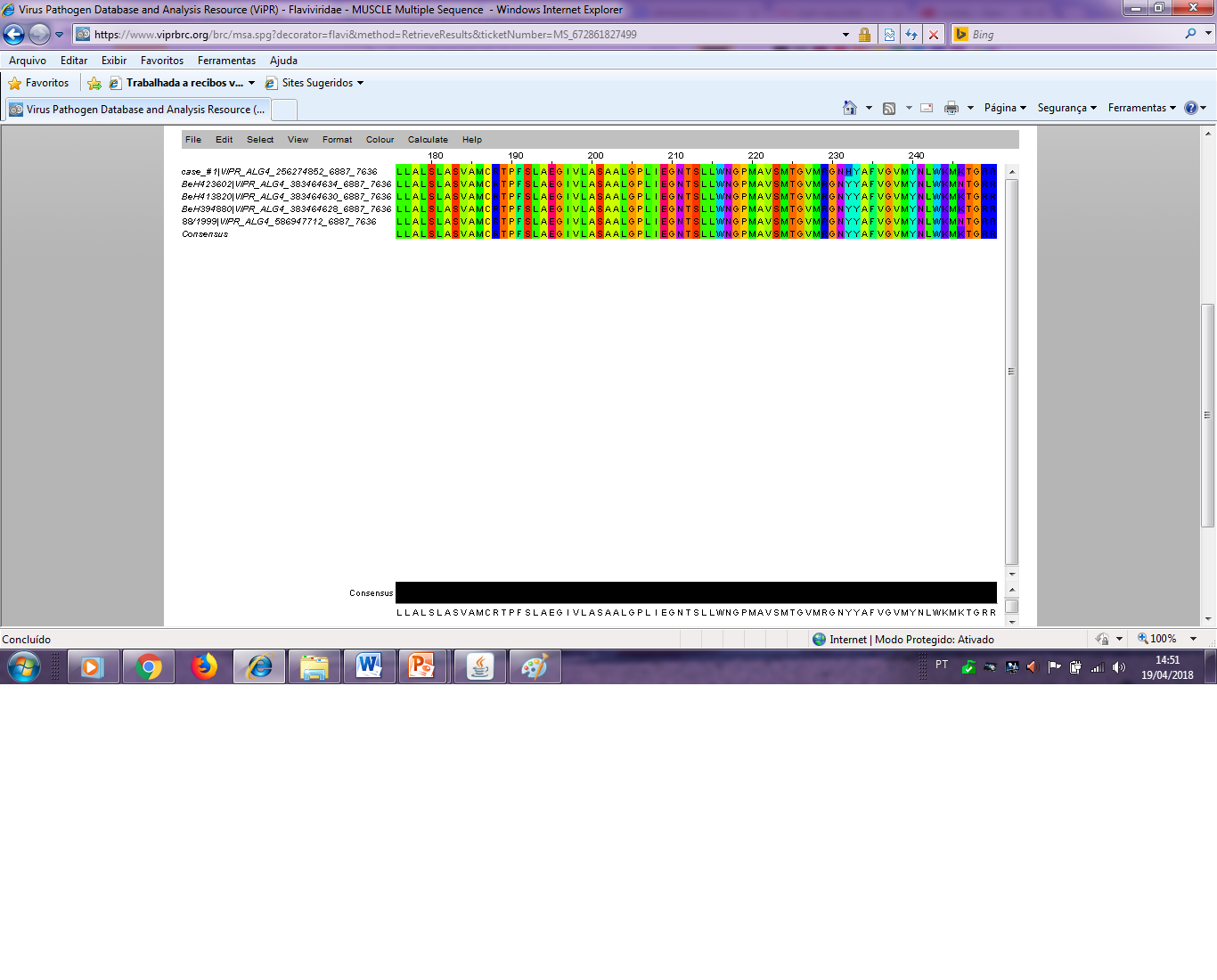

Supplement: Supplementary file 1 — Supplementary Information. [file 41598_2024_60680_MOESM1_ESM.zip › Yellow_Fever_data/1_Acquisition_proteins/Prints VIPR/ns4b/passo 2 ns4b.png]

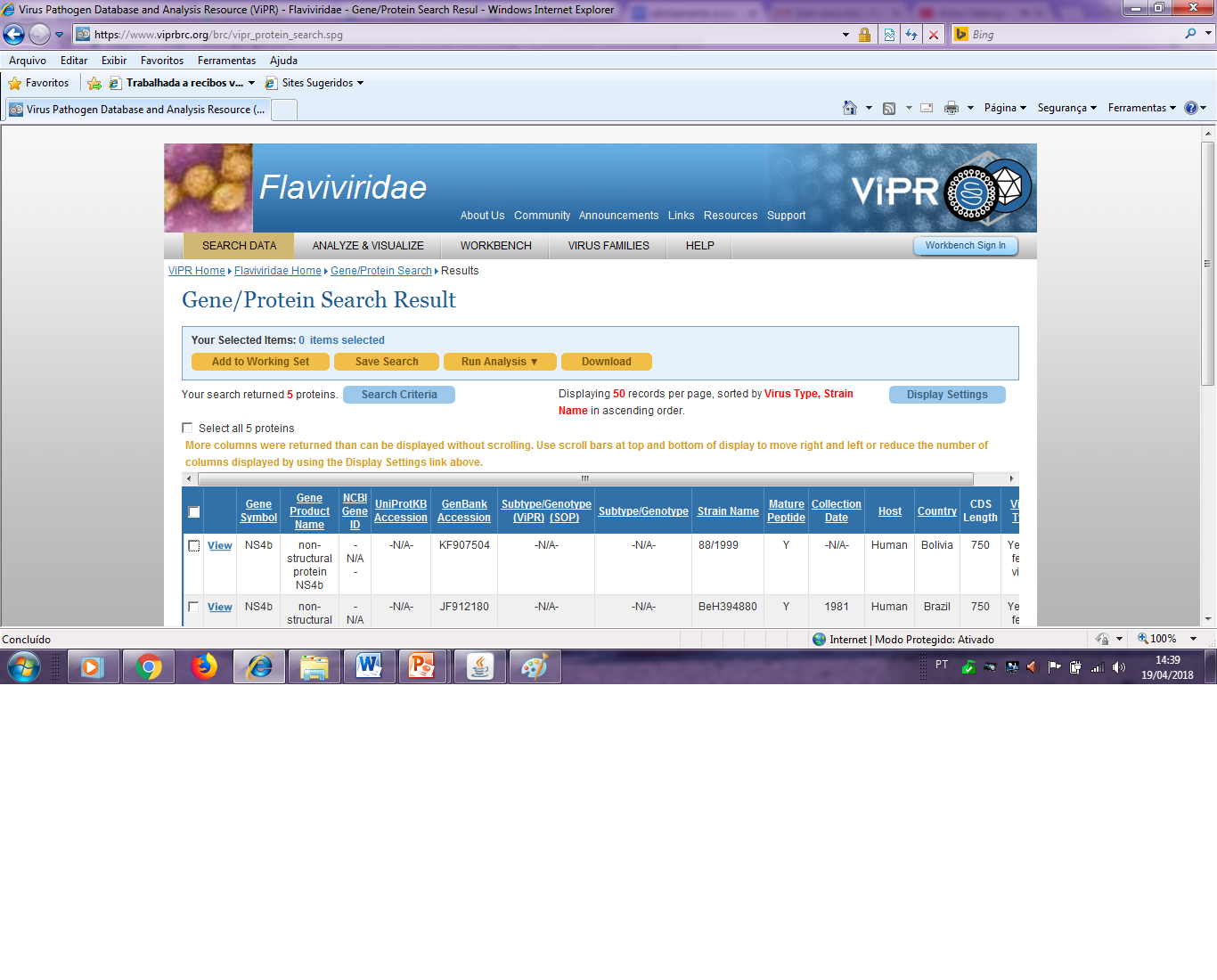

Supplement: Supplementary file 1 — Supplementary Information. [file 41598_2024_60680_MOESM1_ESM.zip › Yellow_Fever_data/1_Acquisition_proteins/Prints VIPR/ns4b/passo 1 ns4b.png]

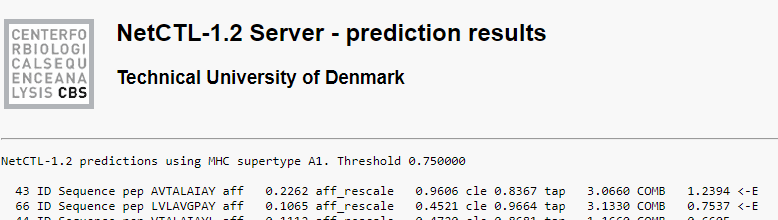

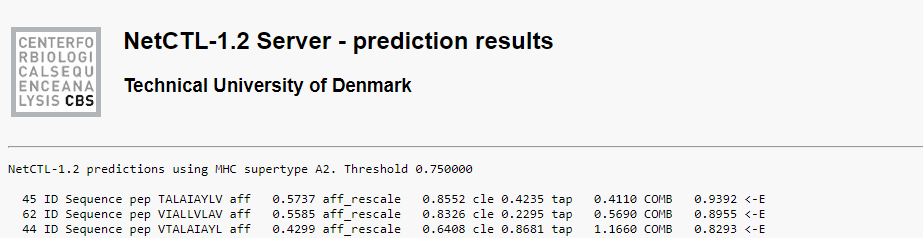

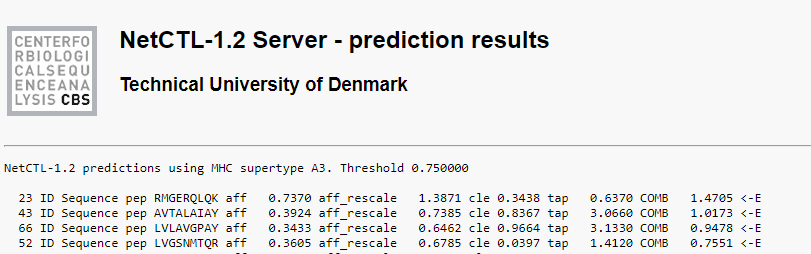

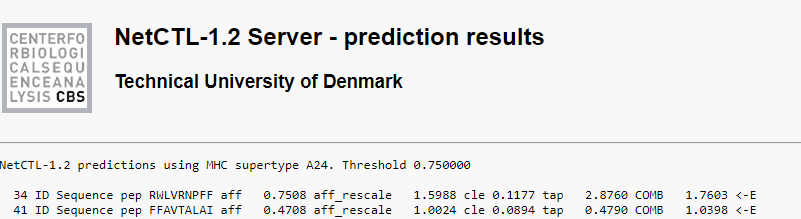

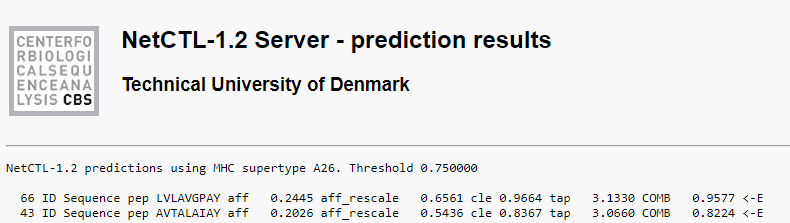

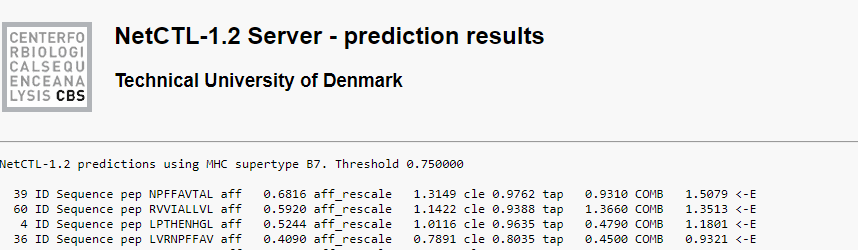

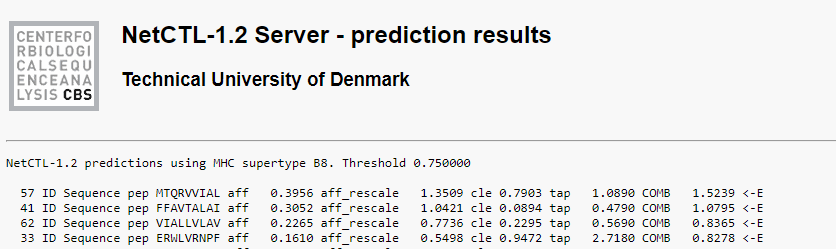

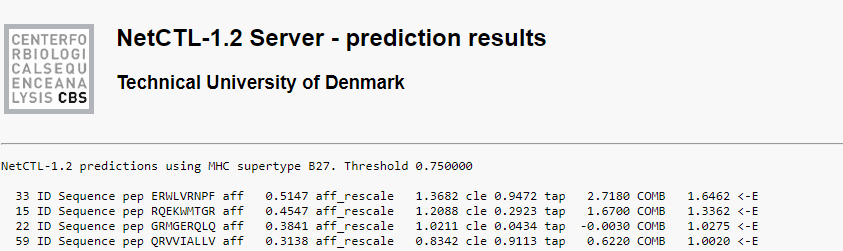

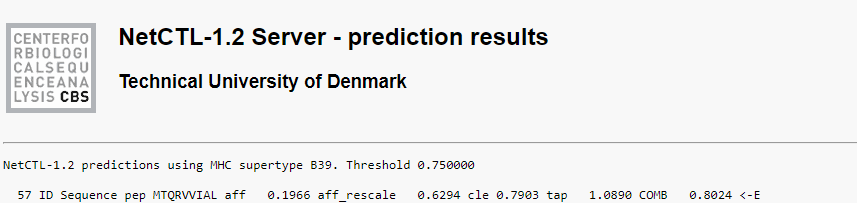

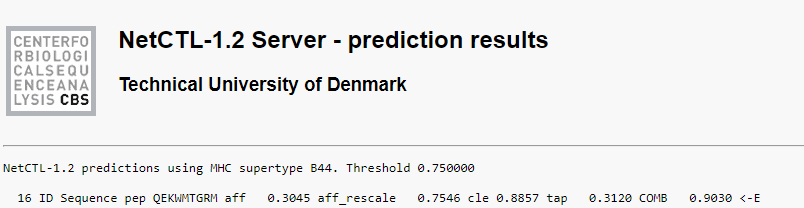

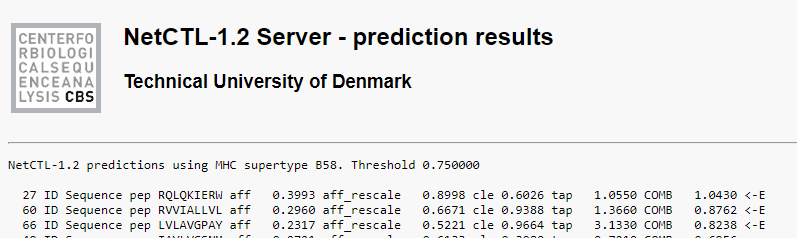

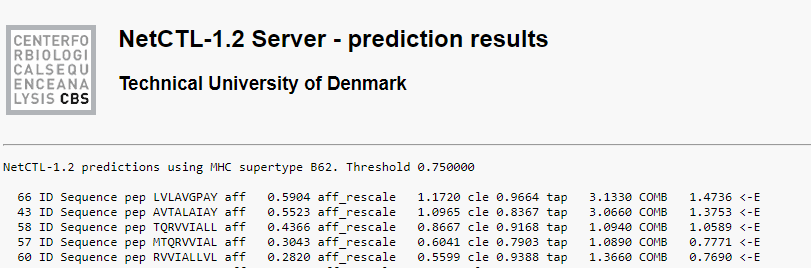

Supplement: Supplementary file 1 — Supplementary Information. [file 41598_2024_60680_MOESM1_ESM.zip › Yellow_Fever_data/2_Prediction of T-cell epitopes/NetCTL/M/NetCTL - M.docx]

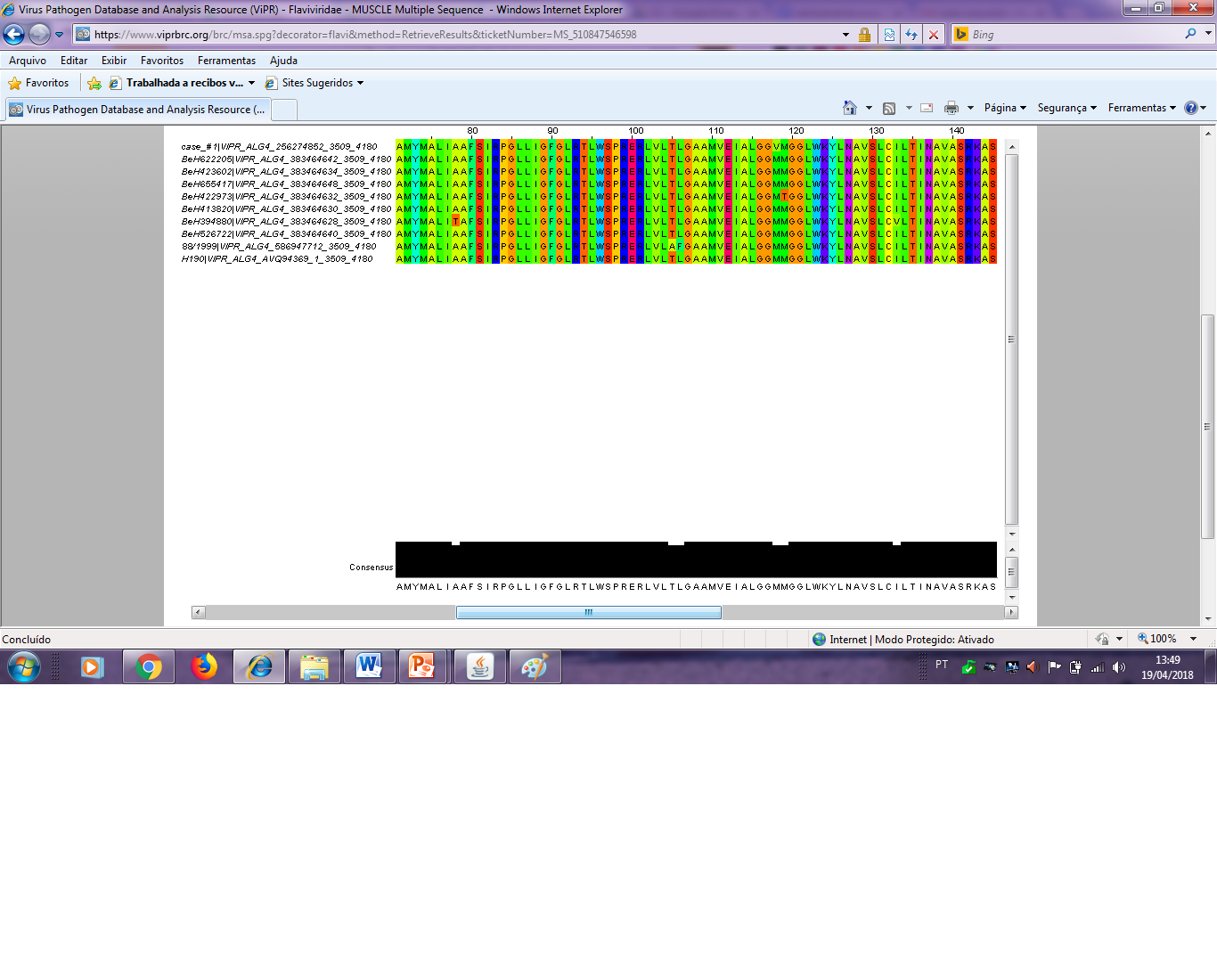

Supplement: Supplementary file 1 — Supplementary Information. [file 41598_2024_60680_MOESM1_ESM.zip › Yellow_Fever_data/1_Acquisition_proteins/Prints VIPR/ns2a/passo 3.2 ns2a.png]

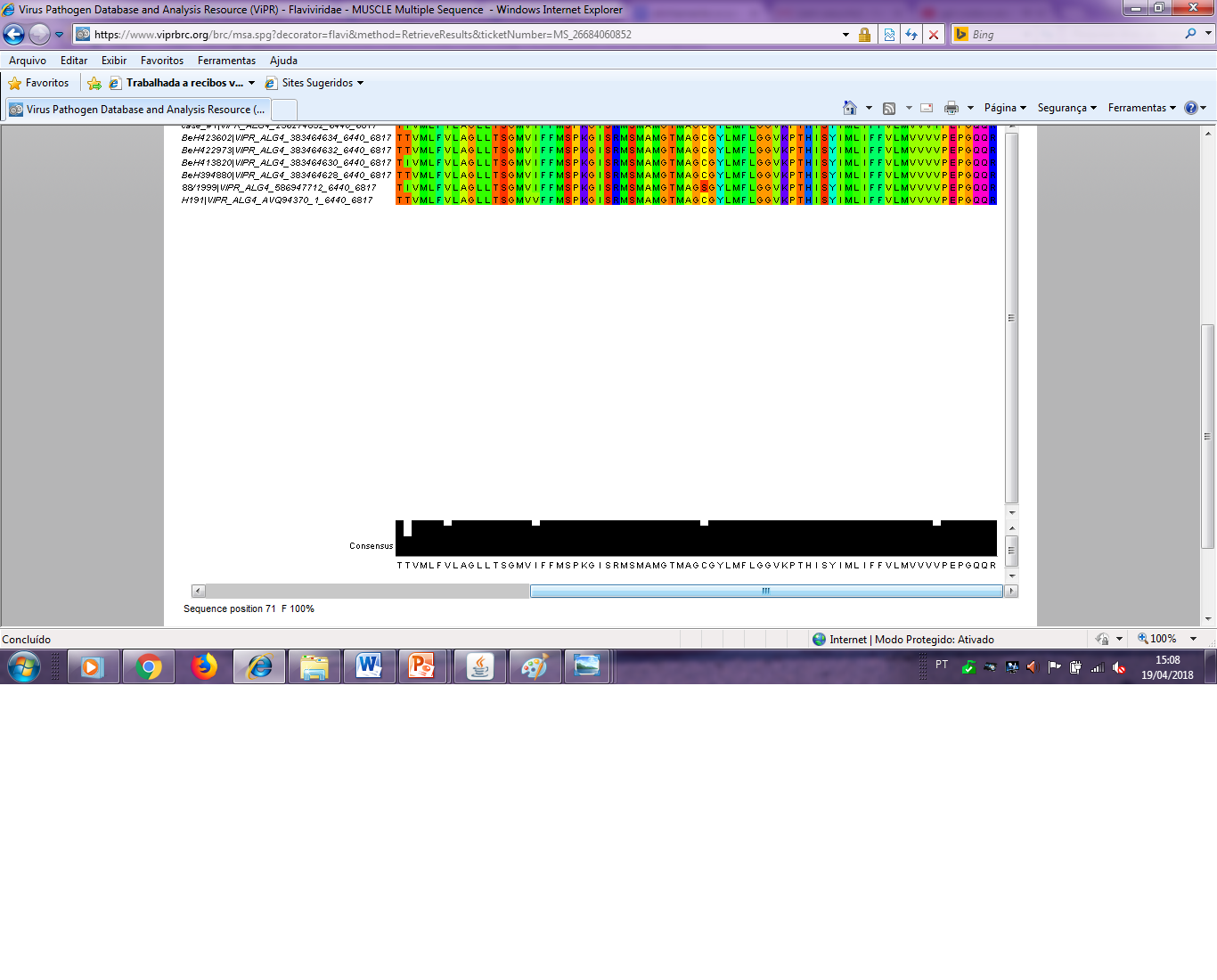

Supplement: Supplementary file 1 — Supplementary Information. [file 41598_2024_60680_MOESM1_ESM.zip › Yellow_Fever_data/1_Acquisition_proteins/Prints VIPR/ns4a/passo 3.2 ns4a.png]

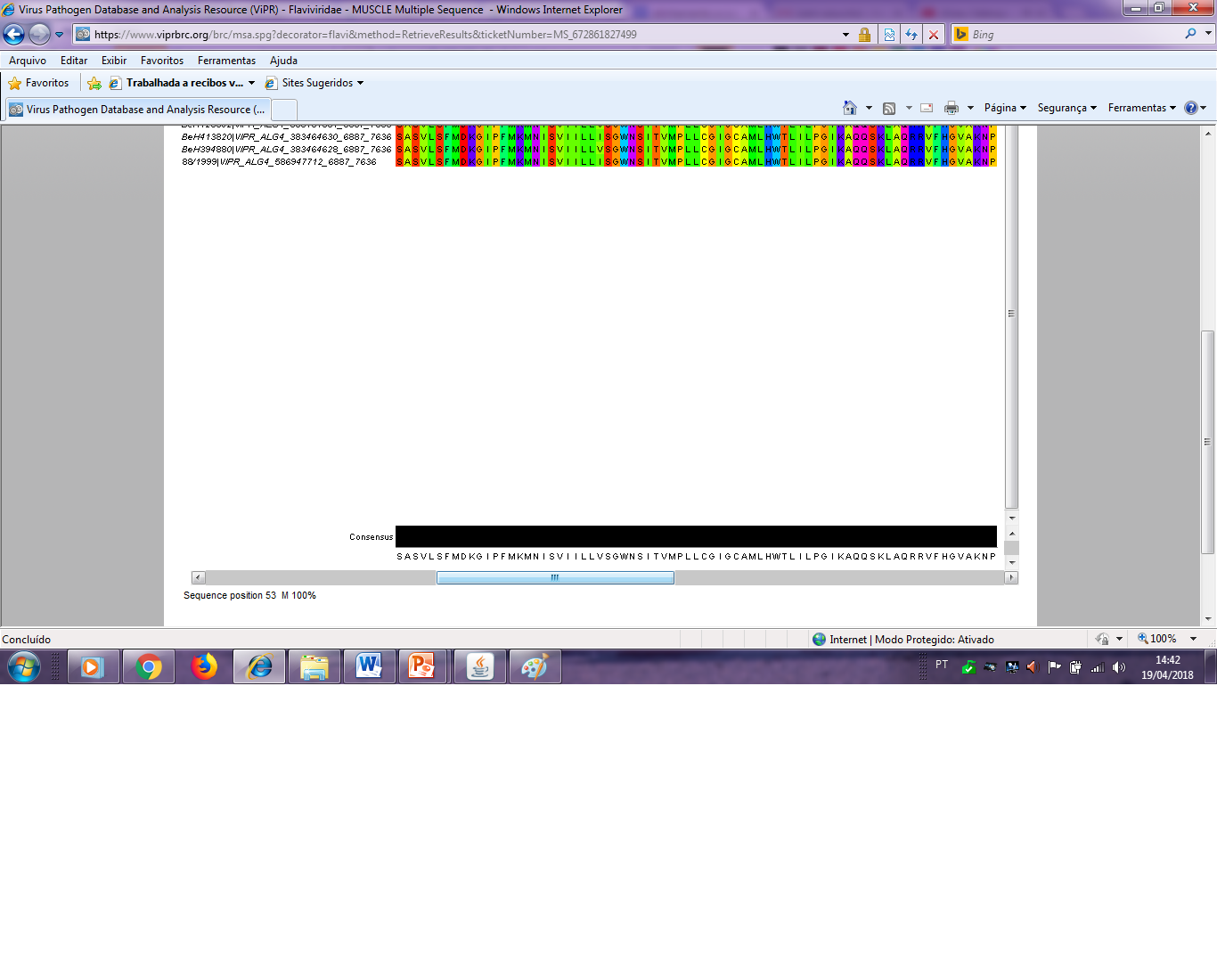

Supplement: Supplementary file 1 — Supplementary Information. [file 41598_2024_60680_MOESM1_ESM.zip › Yellow_Fever_data/1_Acquisition_proteins/Prints VIPR/ns4b/passo 3.2 ns4b.png]

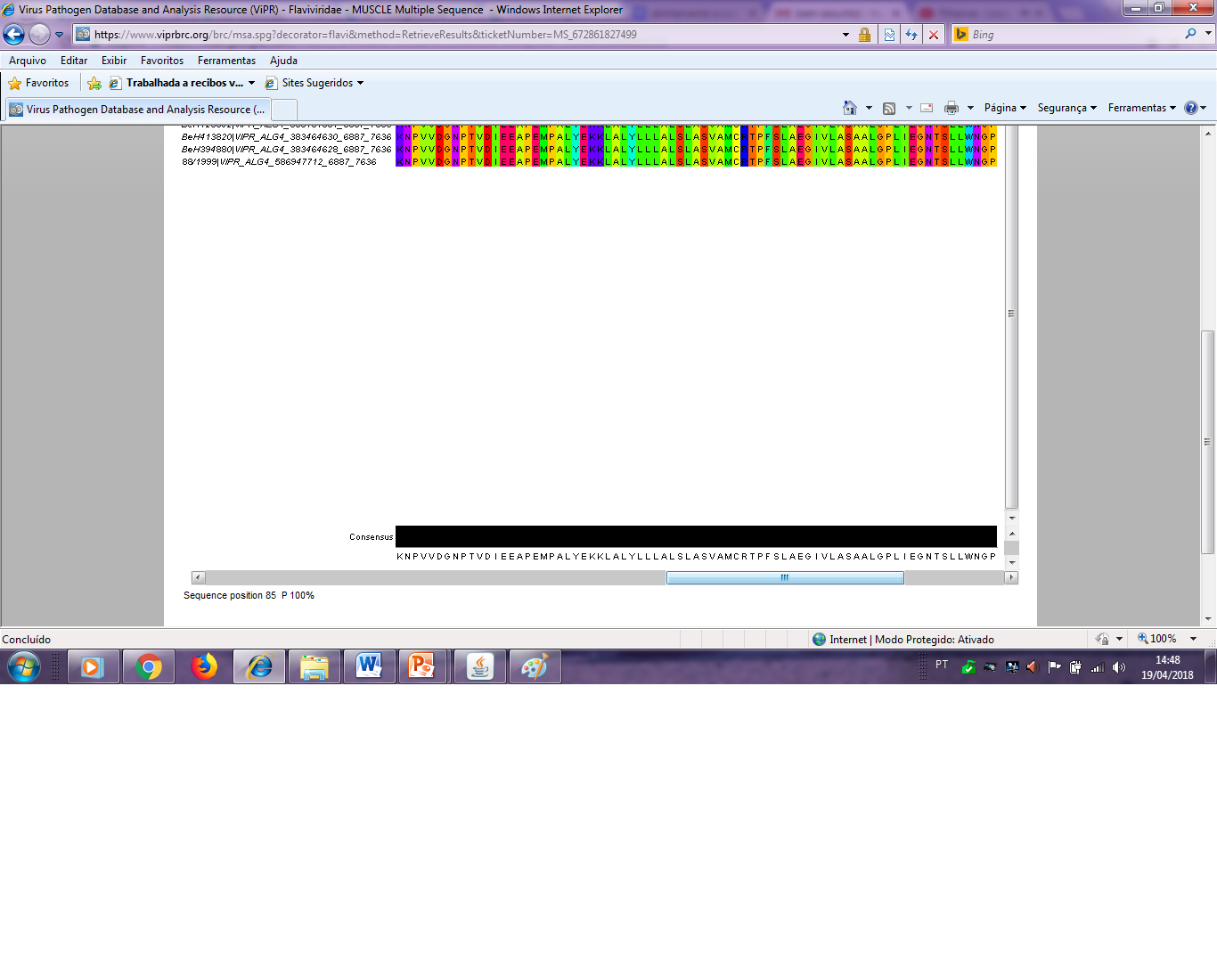

Supplement: Supplementary file 1 — Supplementary Information. [file 41598_2024_60680_MOESM1_ESM.zip › Yellow_Fever_data/1_Acquisition_proteins/Prints VIPR/ns4b/passo 3.3 ns4b.png]

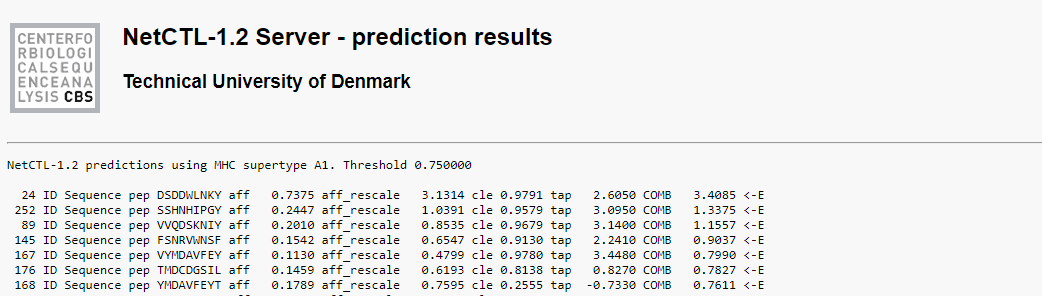

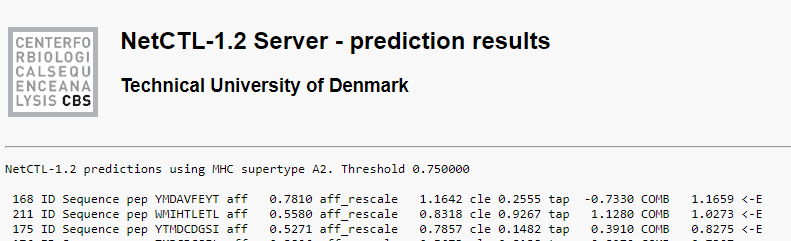

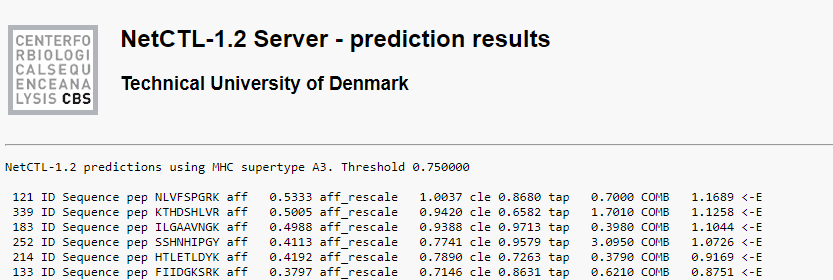

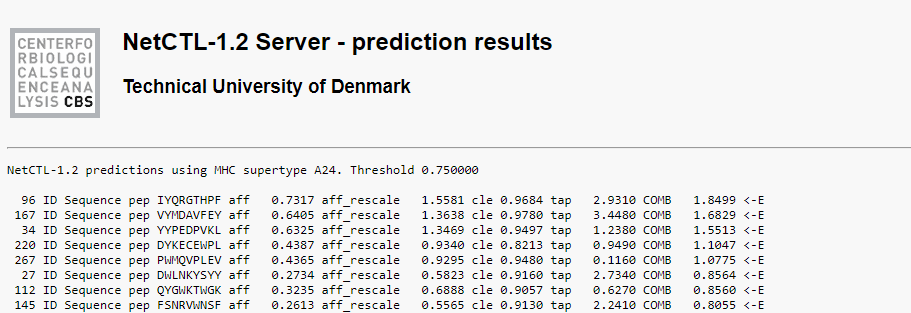

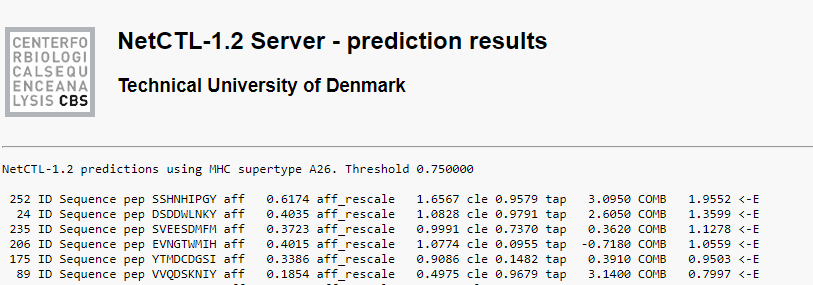

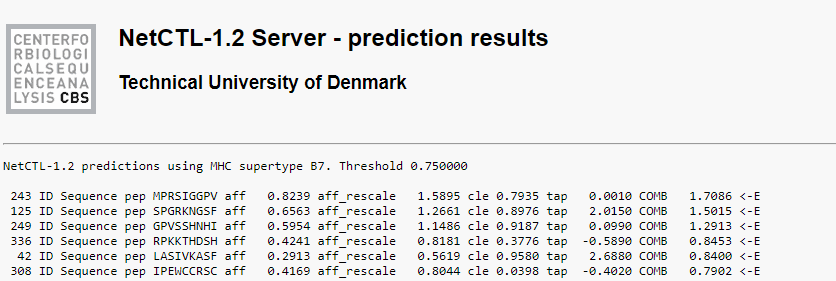

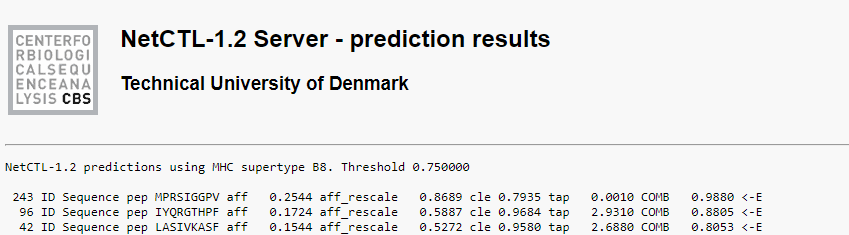

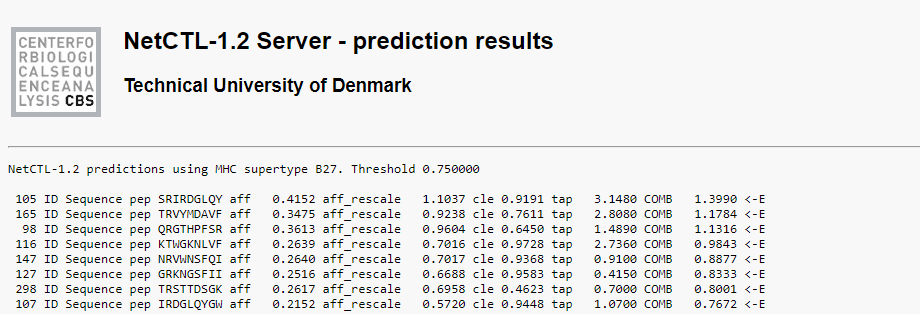

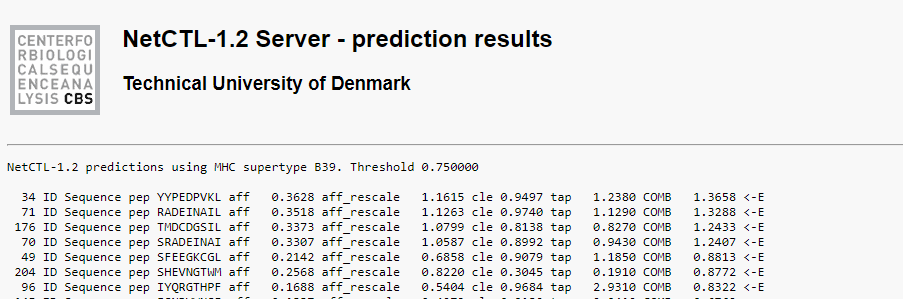

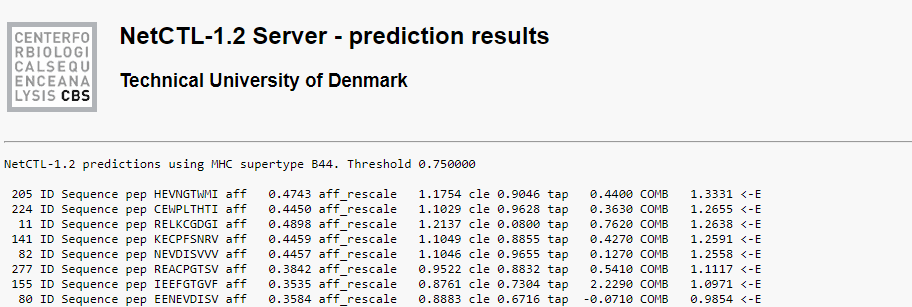

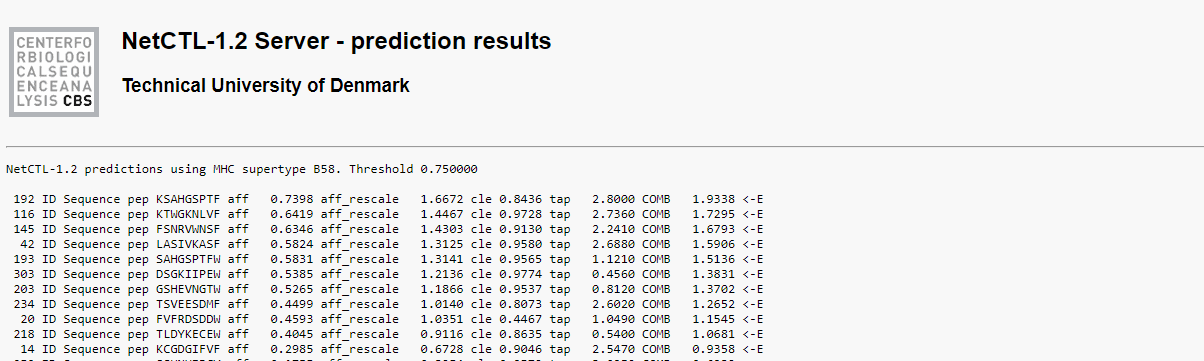

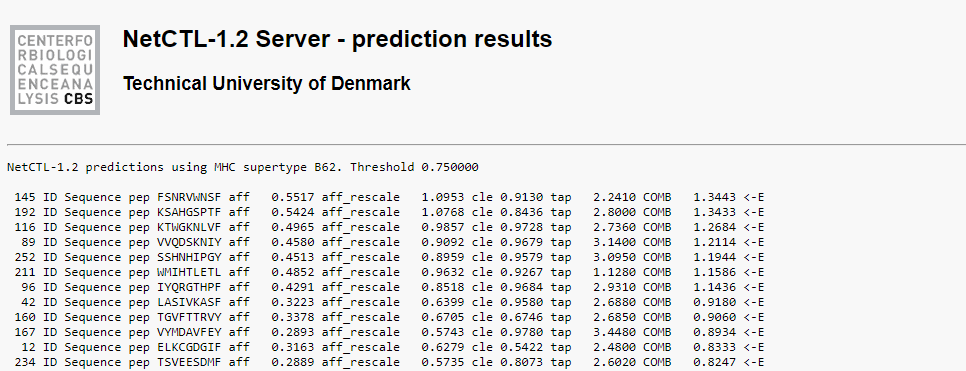

Supplement: Supplementary file 1 — Supplementary Information. [file 41598_2024_60680_MOESM1_ESM.zip › Yellow_Fever_data/2_Prediction of T-cell epitopes/NetCTL/NS1/NetCTL-NS1.docx]

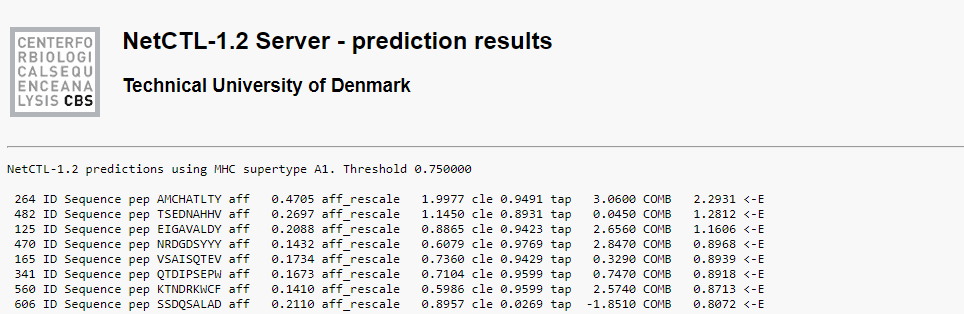

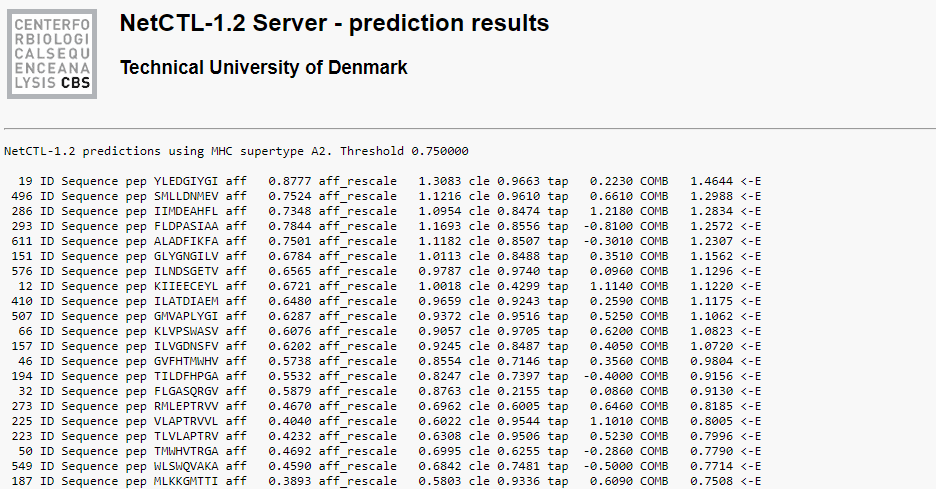

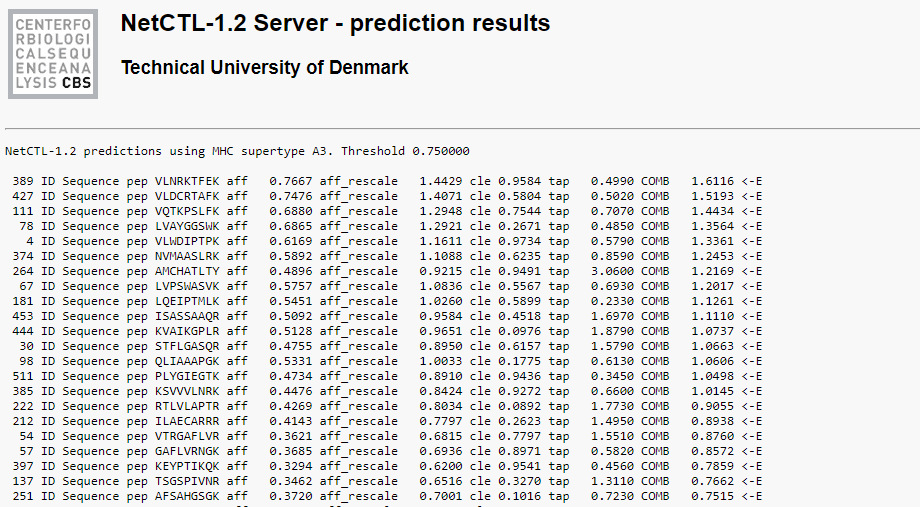

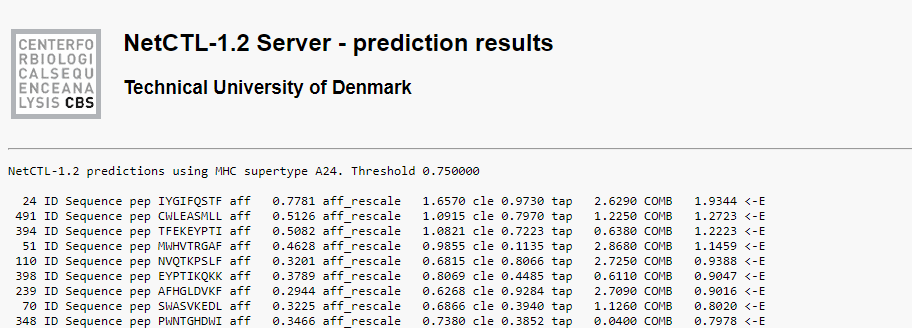

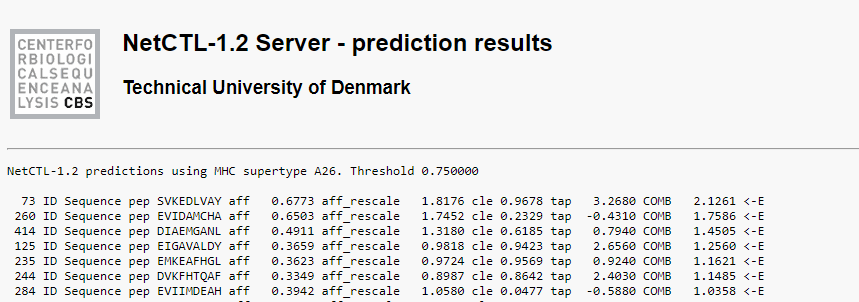

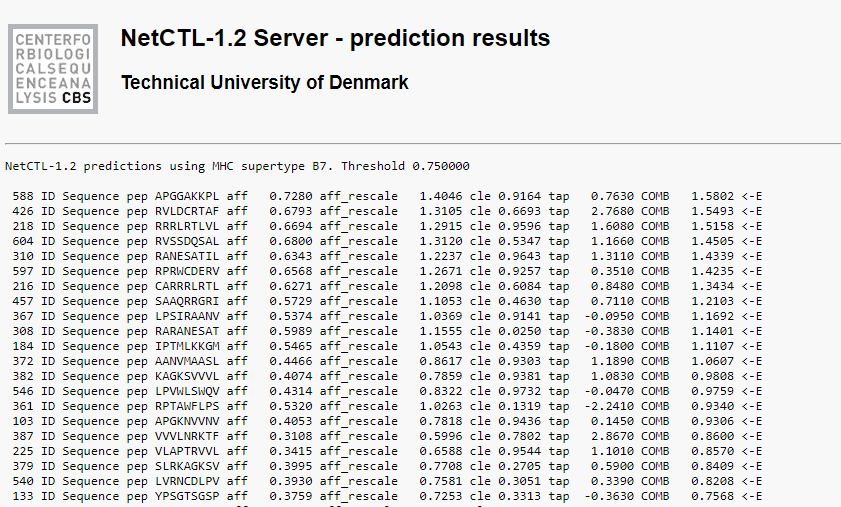

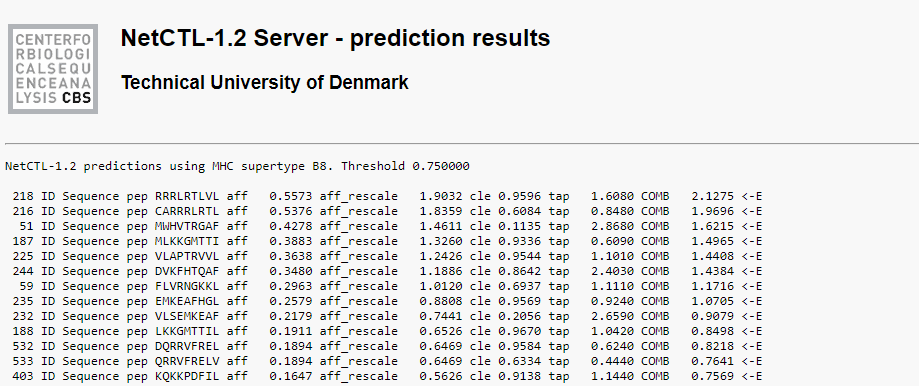

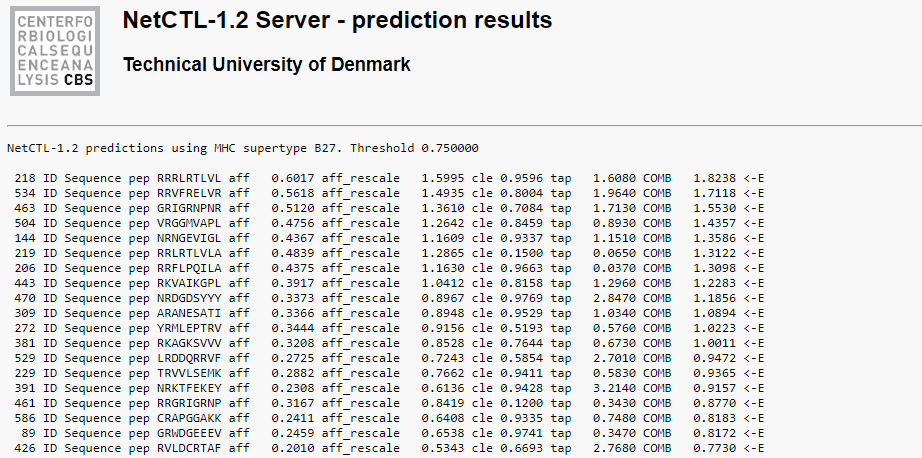

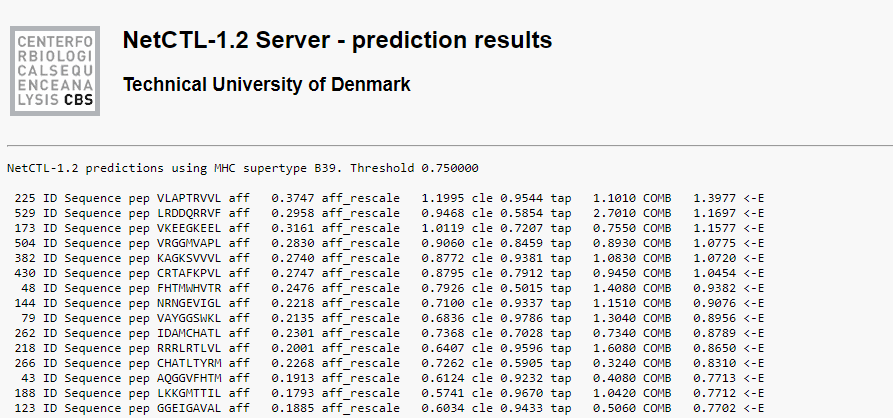

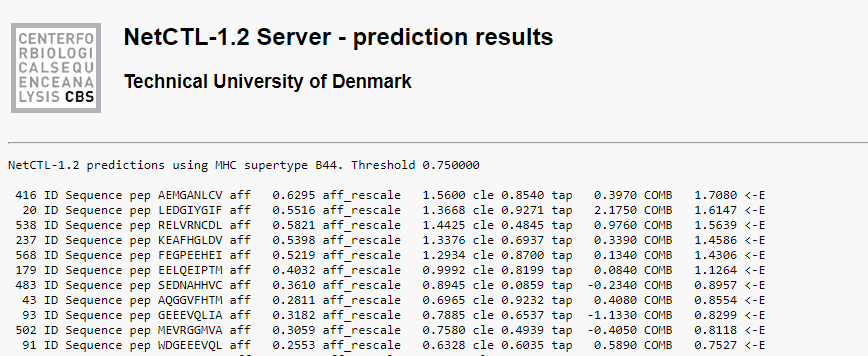

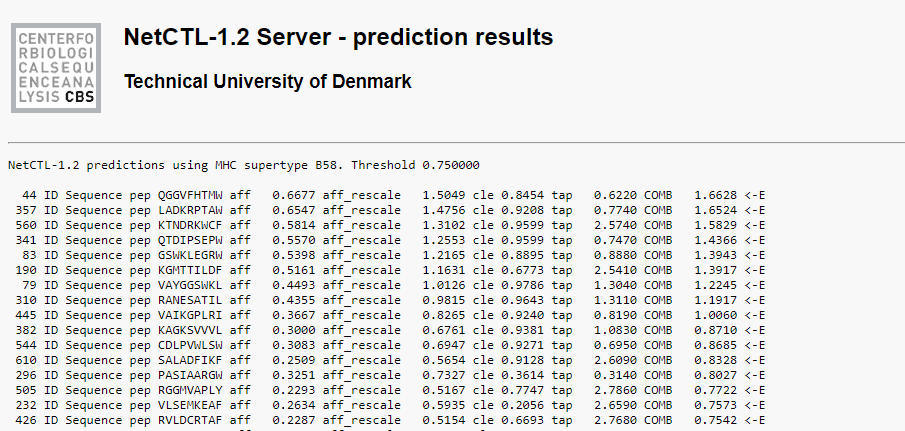

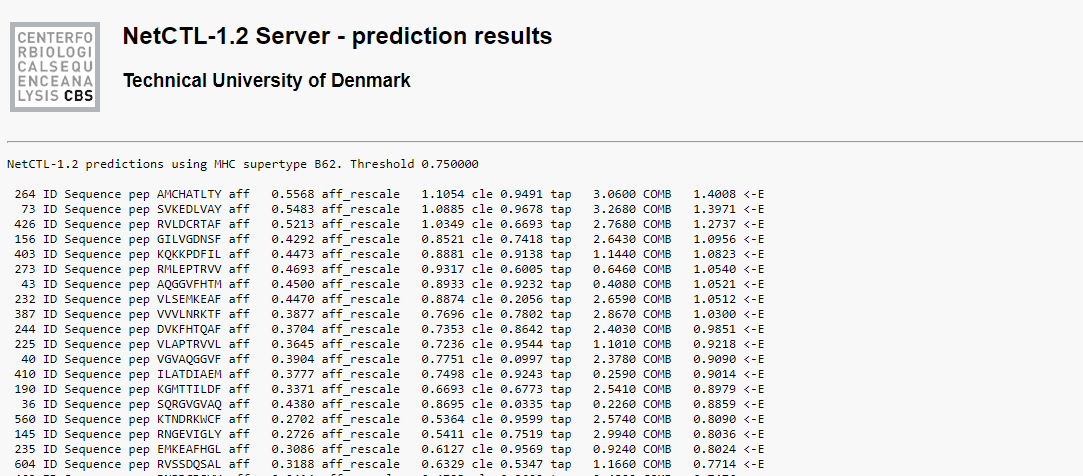

Supplement: Supplementary file 1 — Supplementary Information. [file 41598_2024_60680_MOESM1_ESM.zip › Yellow_Fever_data/2_Prediction of T-cell epitopes/NetCTL/NS3/NetCTL-NS3.docx]

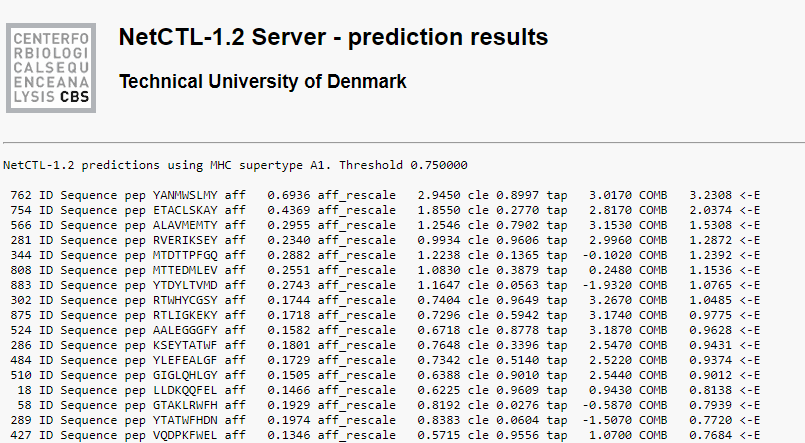

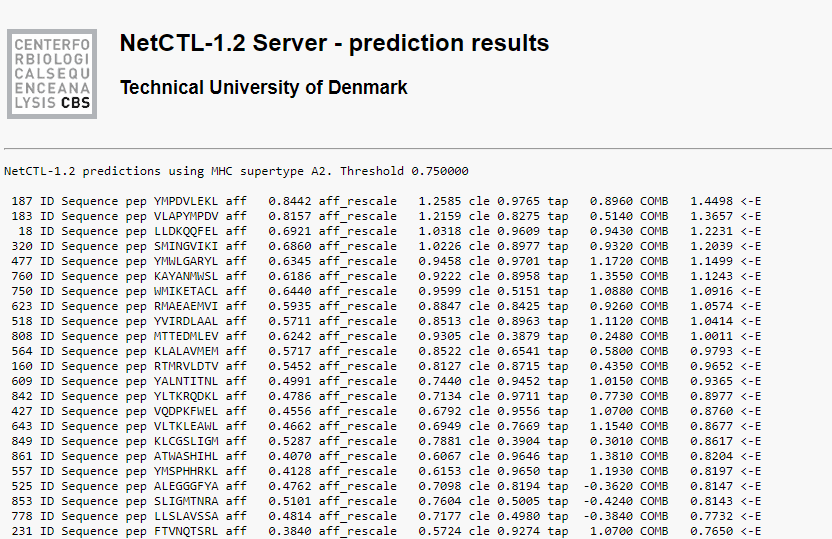

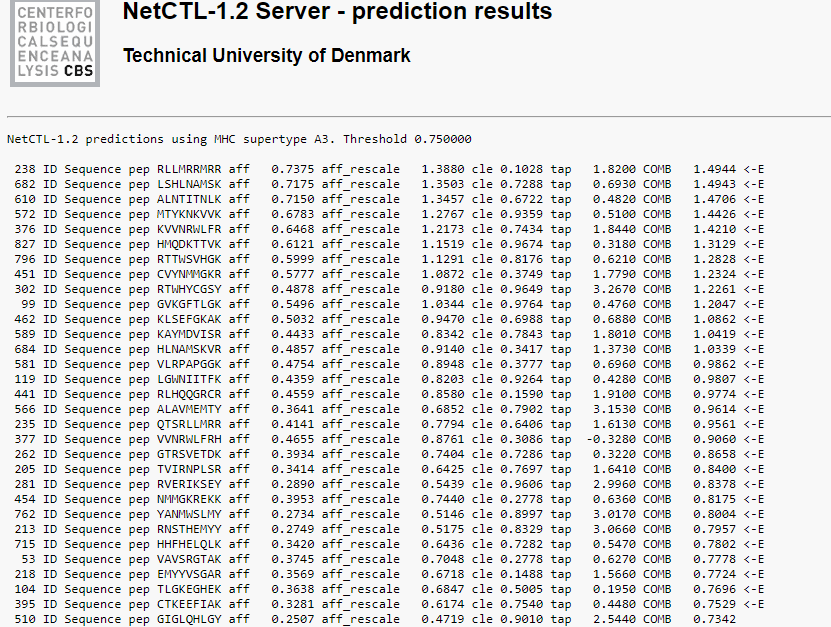

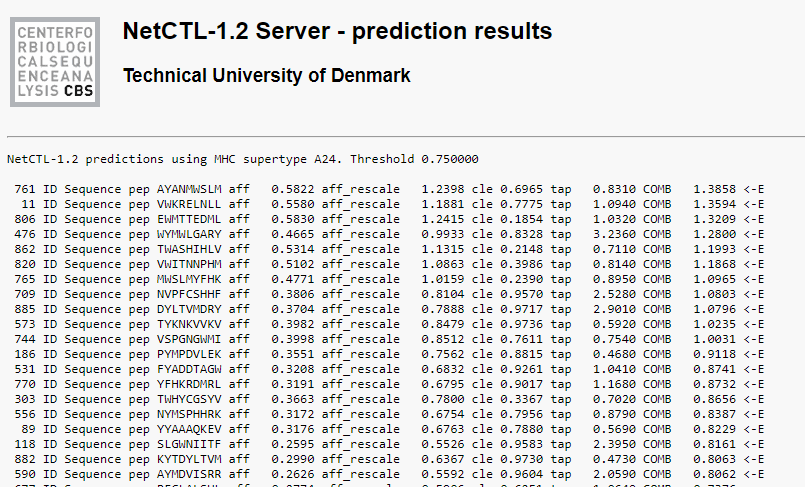

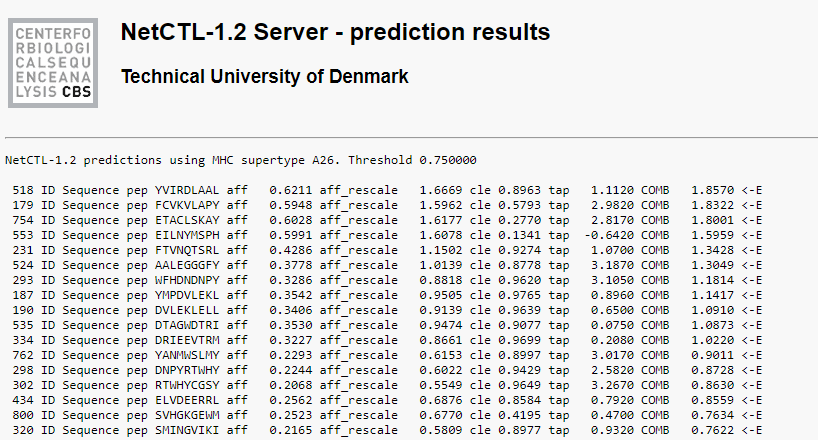

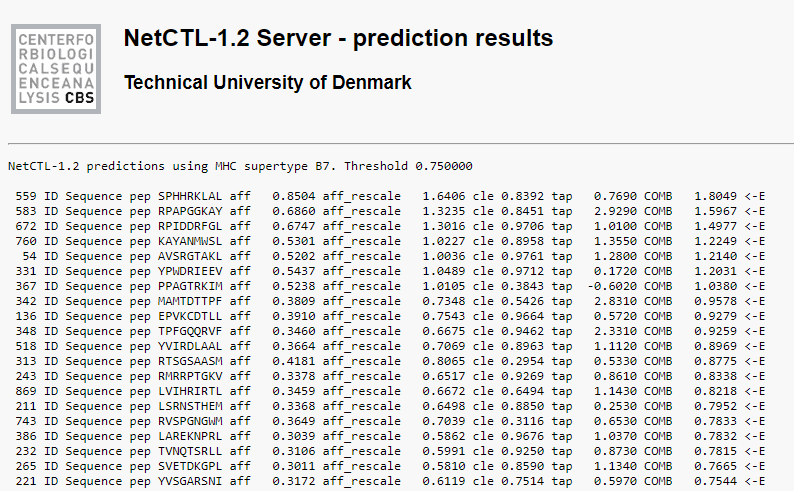

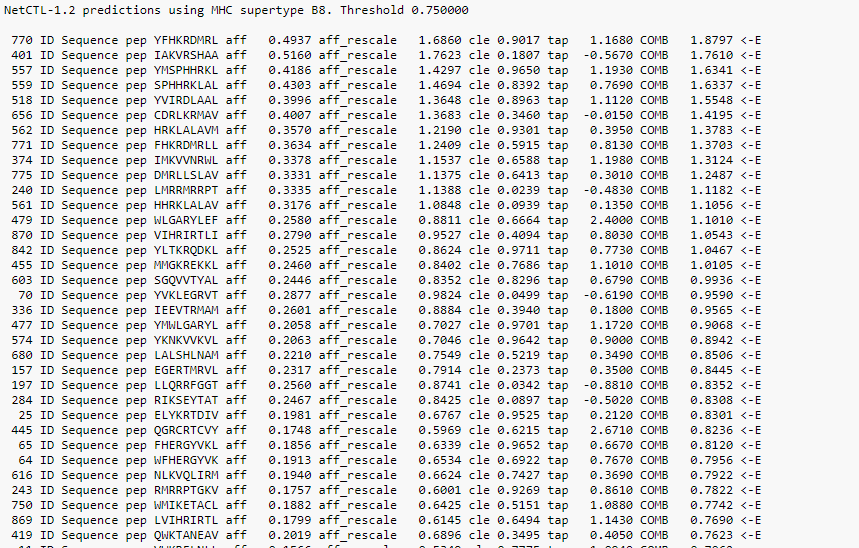

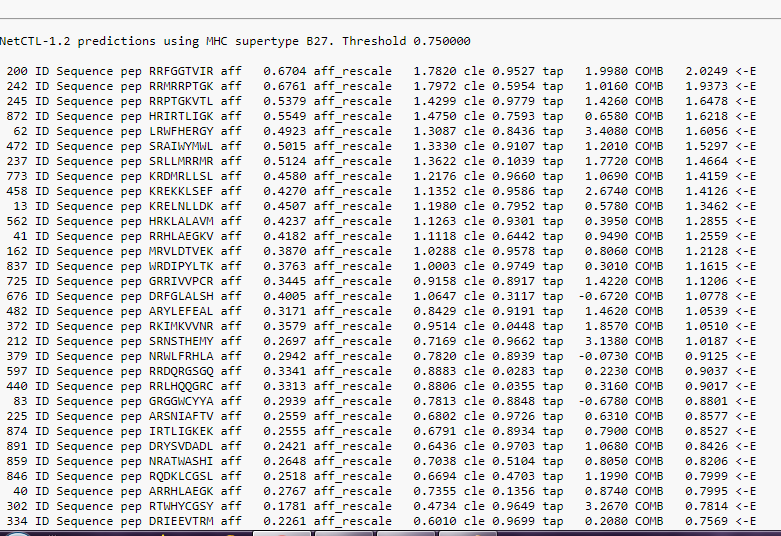

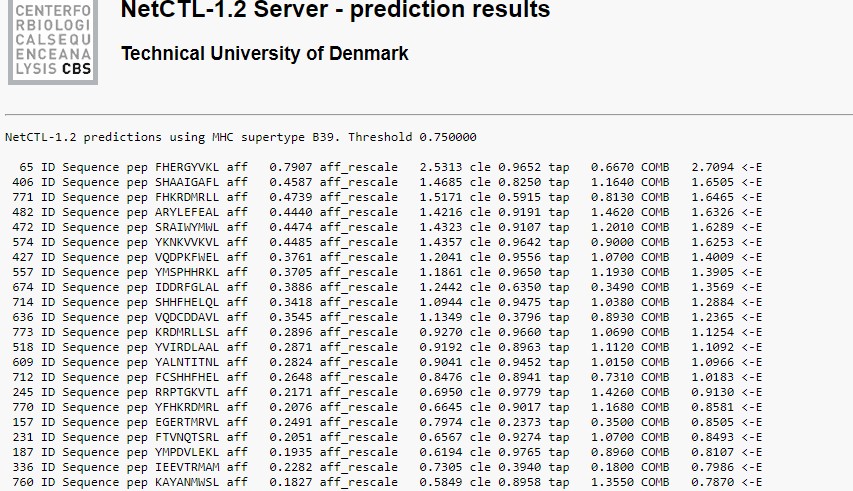

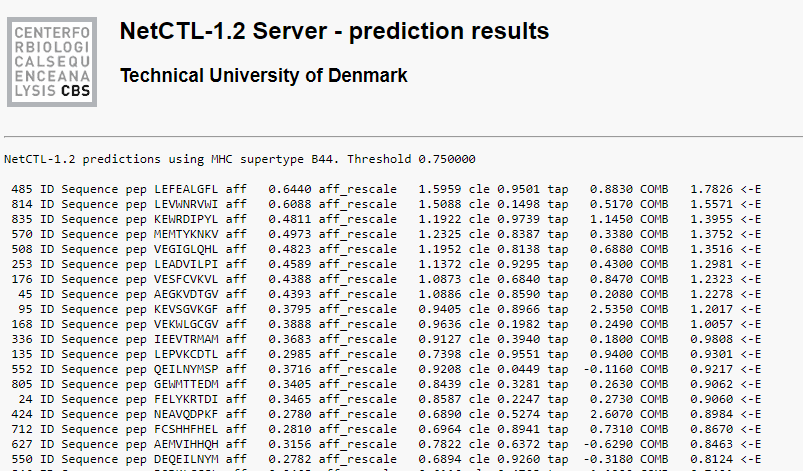

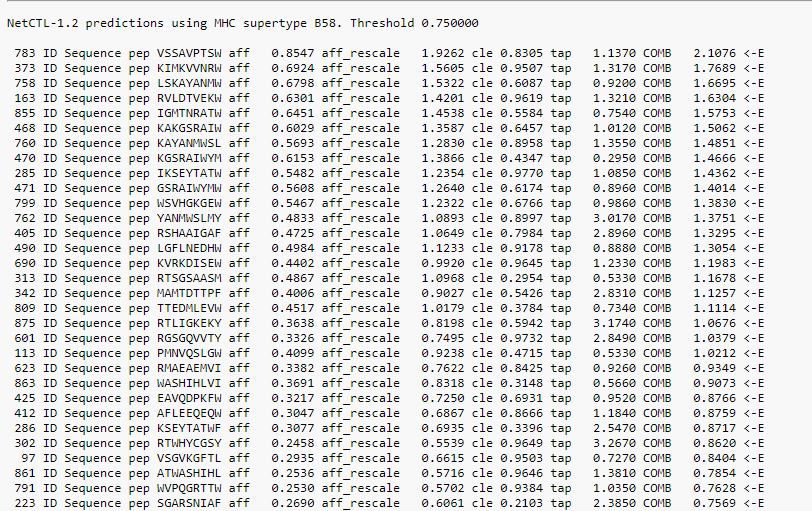

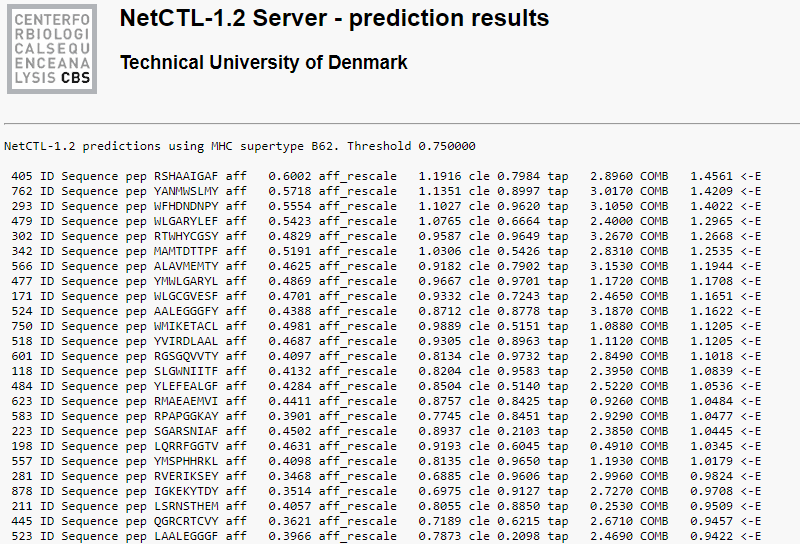

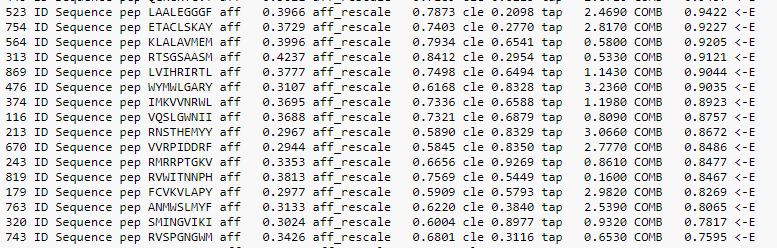

Supplement: Supplementary file 1 — Supplementary Information. [file 41598_2024_60680_MOESM1_ESM.zip › Yellow_Fever_data/2_Prediction of T-cell epitopes/NetCTL/NS5/NetCTL-NS5.docx]

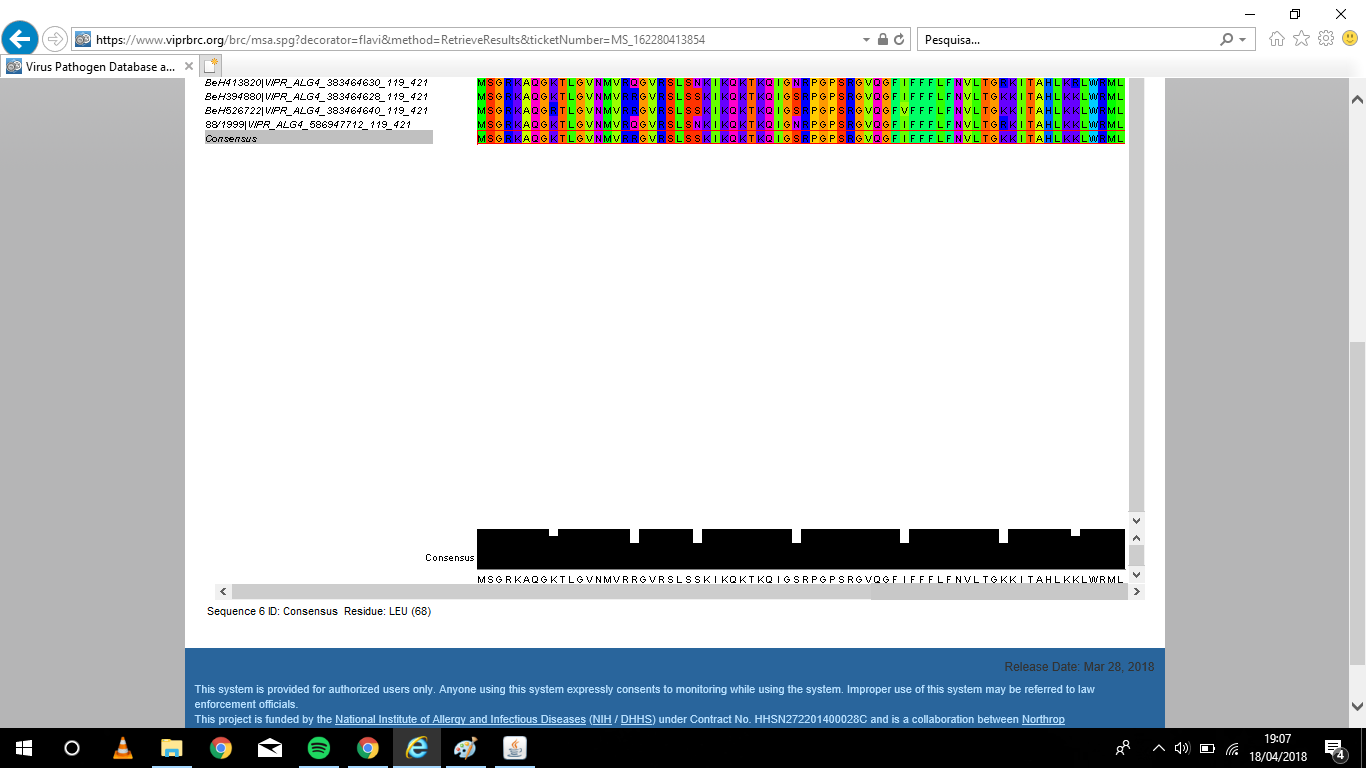

Supplement: Supplementary file 1 — Supplementary Information. [file 41598_2024_60680_MOESM1_ESM.zip › Yellow_Fever_data/1_Acquisition_proteins/Prints VIPR/C/passo 3 c consenso.png]

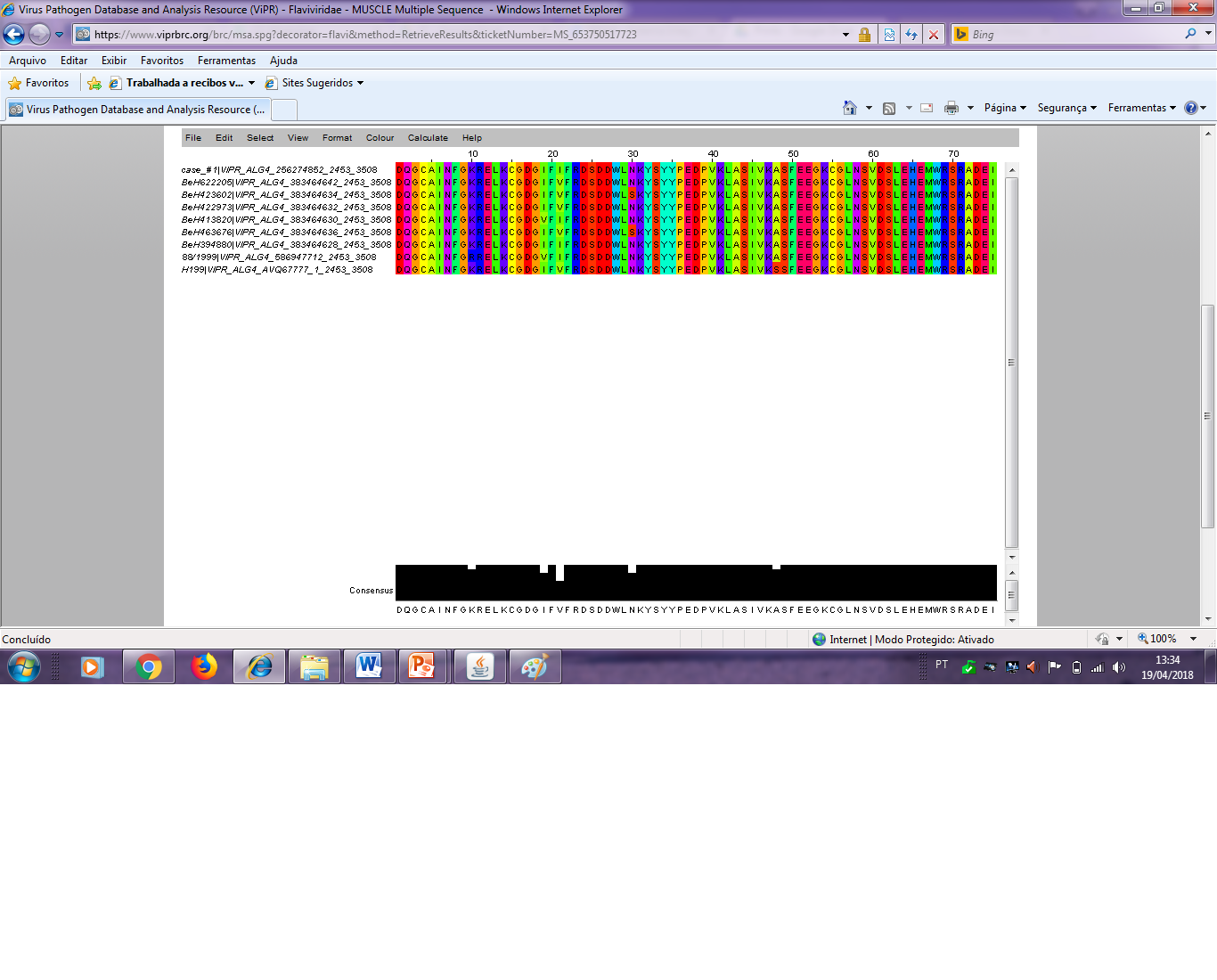

Supplement: Supplementary file 1 — Supplementary Information. [file 41598_2024_60680_MOESM1_ESM.zip › Yellow_Fever_data/1_Acquisition_proteins/Prints VIPR/ns1/passo 3 consenso.png]

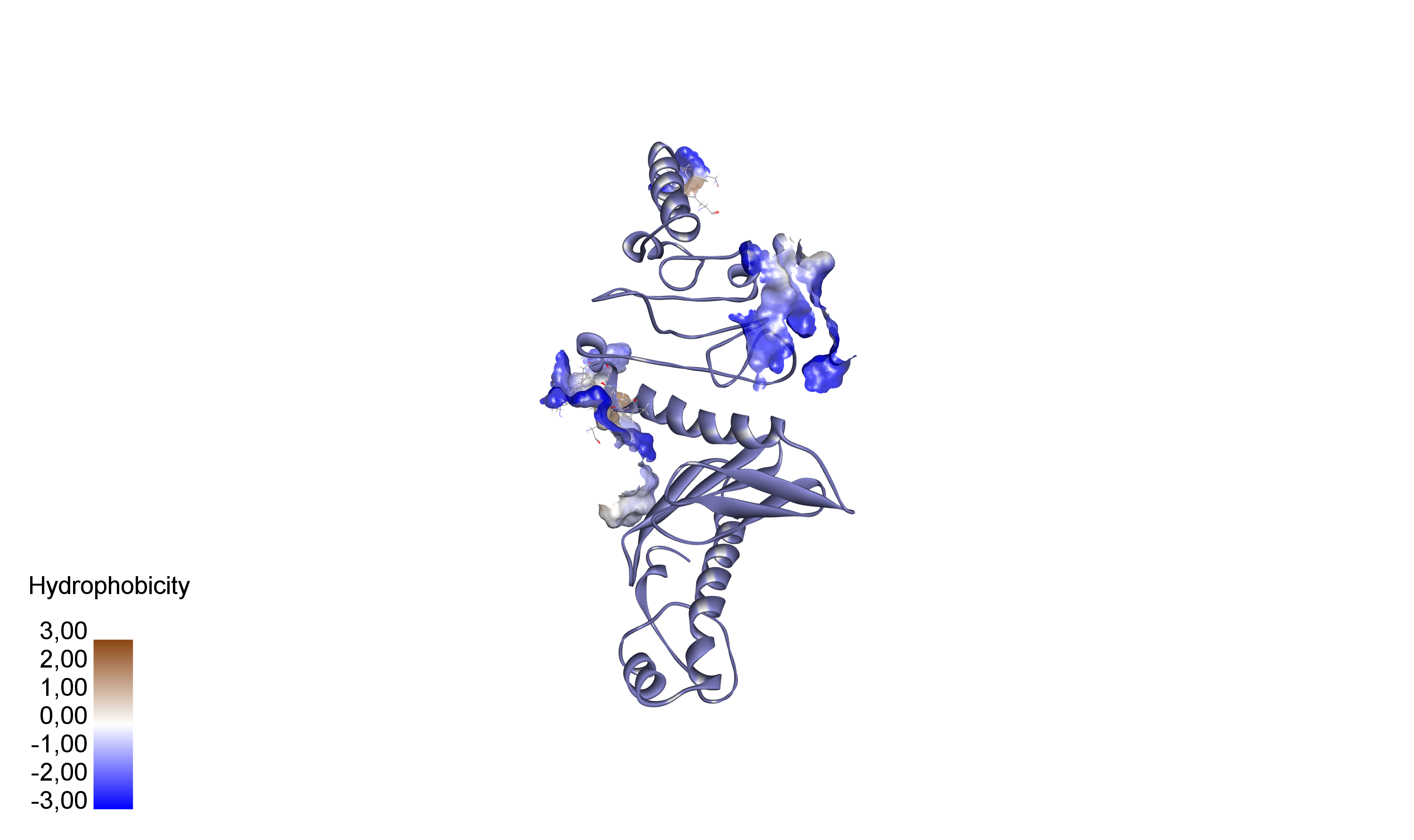

Supplement: Supplementary file 1 — Supplementary Information. [file 41598_2024_60680_MOESM1_ESM.zip › Yellow_Fever_data/6_Intermolecular_interactions/estruturalHADDOCK_YV_hidro.bmp]

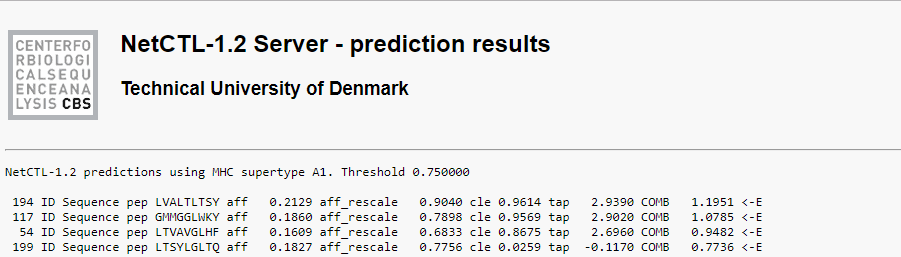

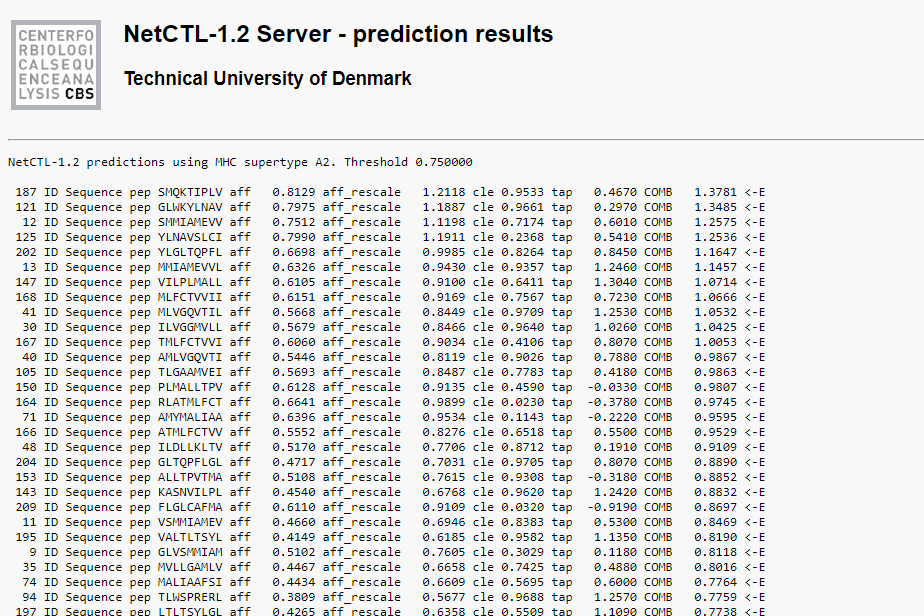

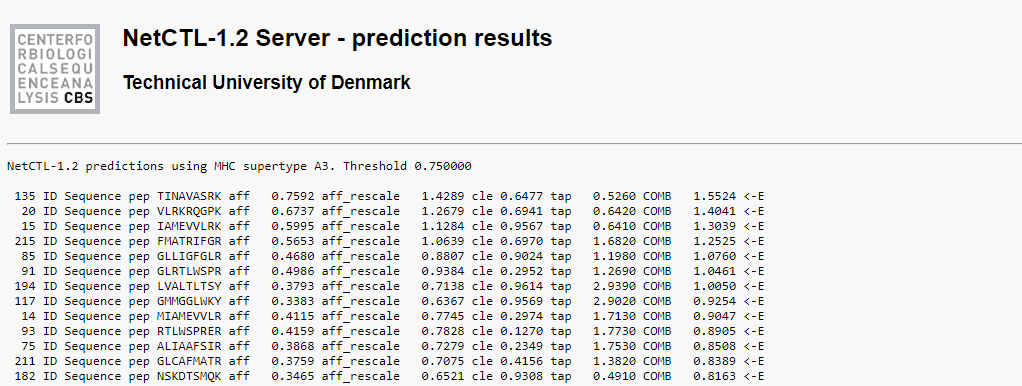

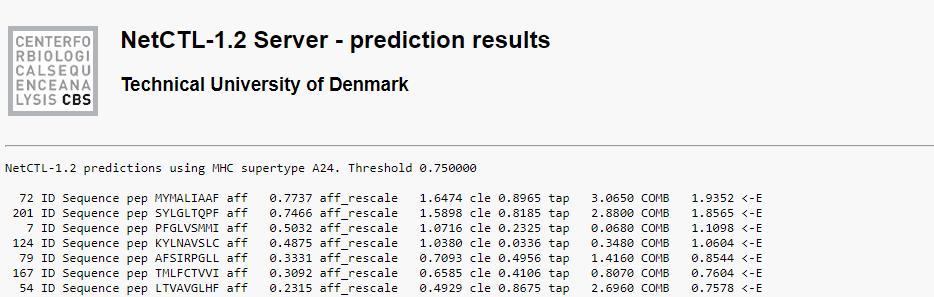

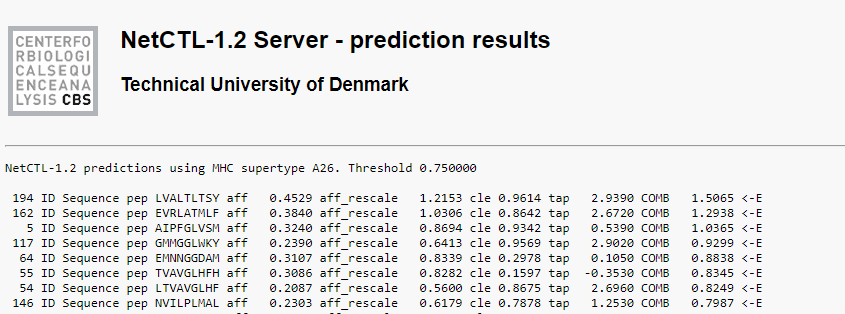

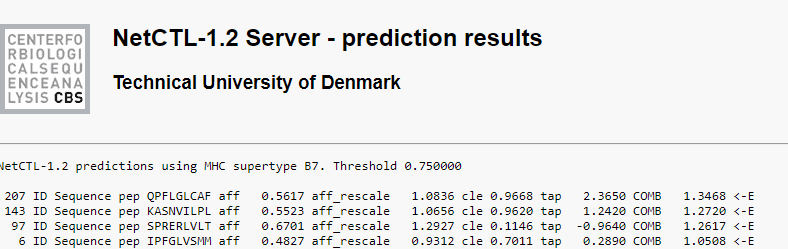

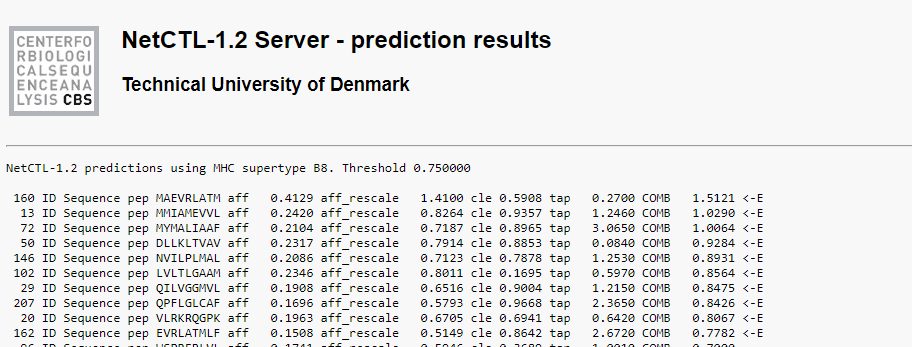

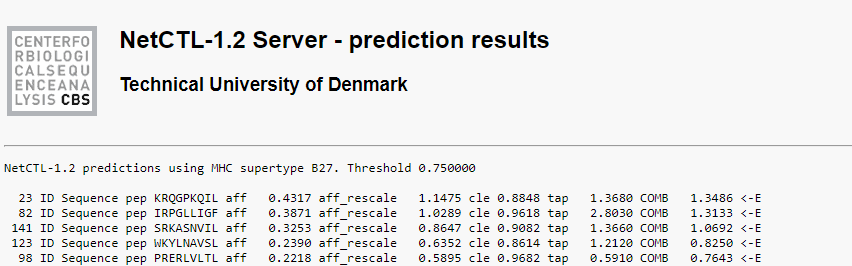

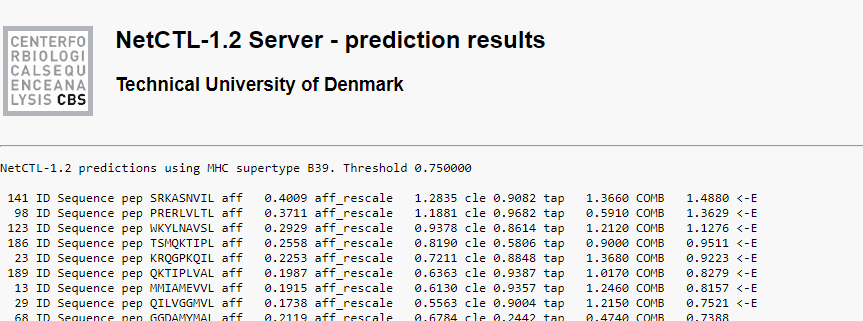

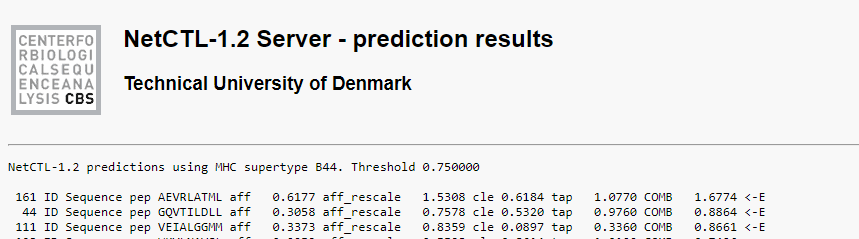

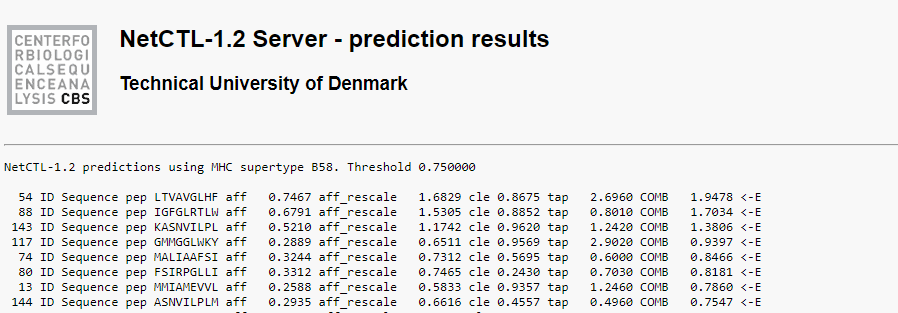

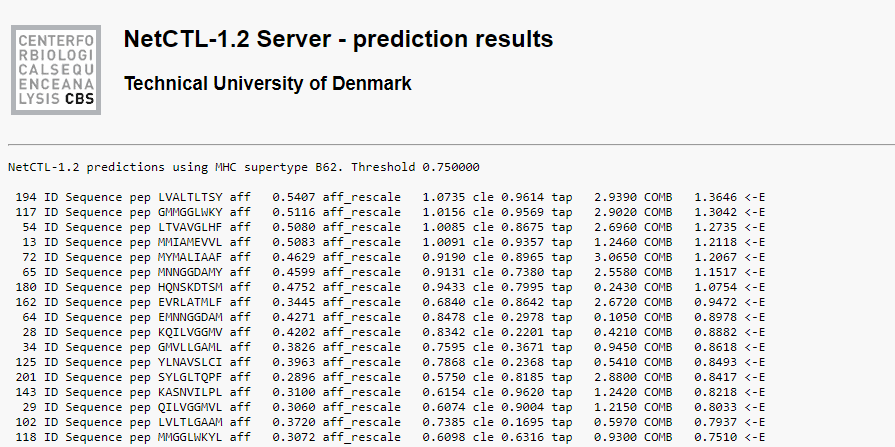

Supplement: Supplementary file 1 — Supplementary Information. [file 41598_2024_60680_MOESM1_ESM.zip › Yellow_Fever_data/2_Prediction of T-cell epitopes/NetCTL/NS2A/NetCTL-NS2A.docx]

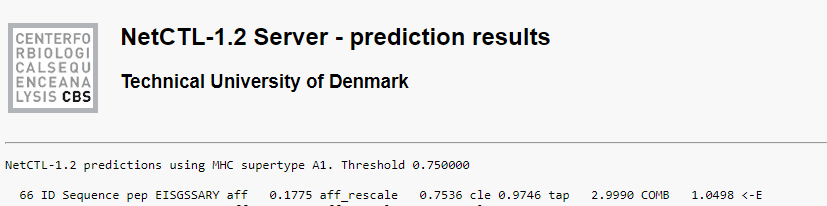

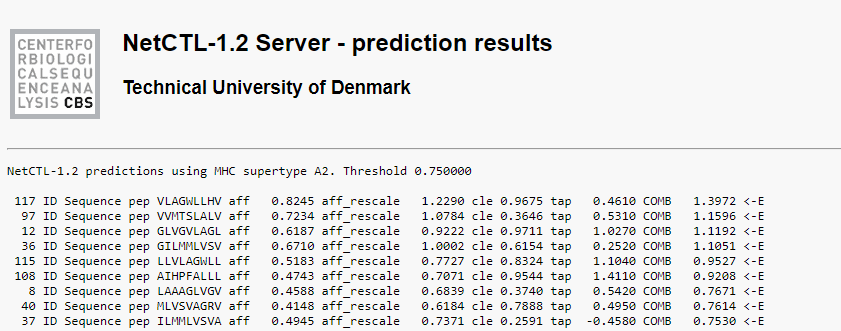

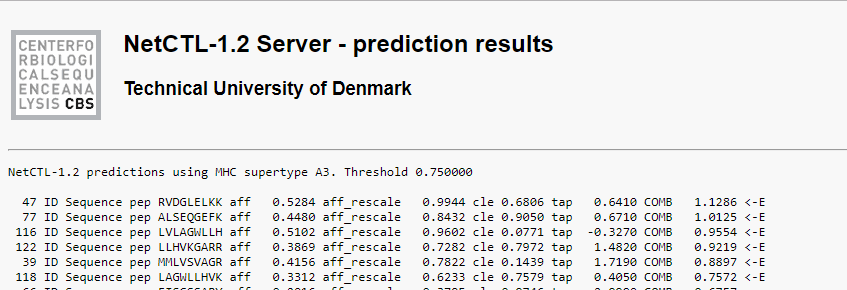

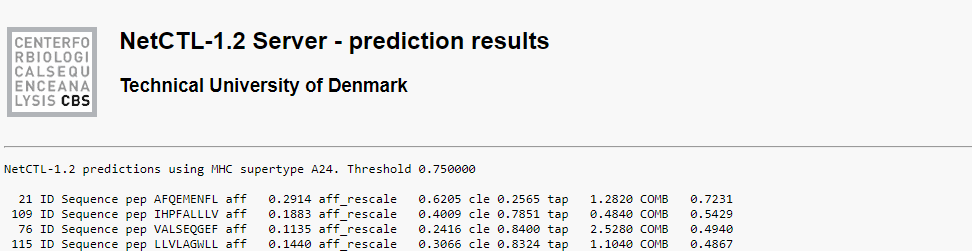

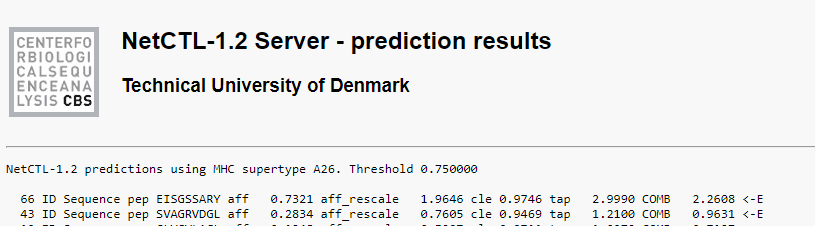

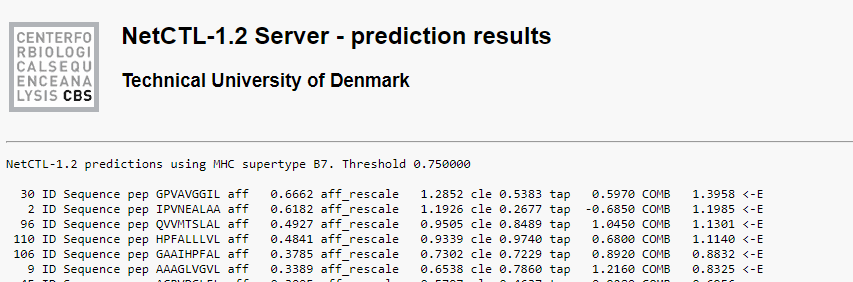

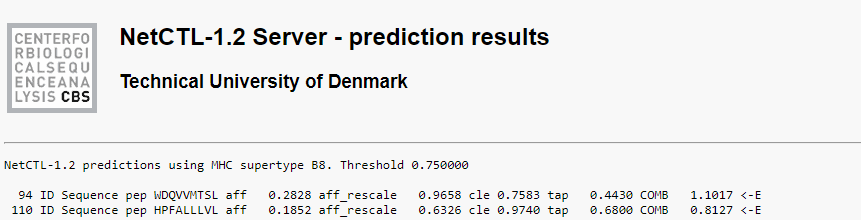

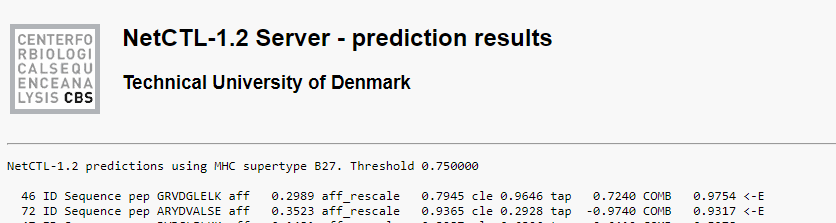

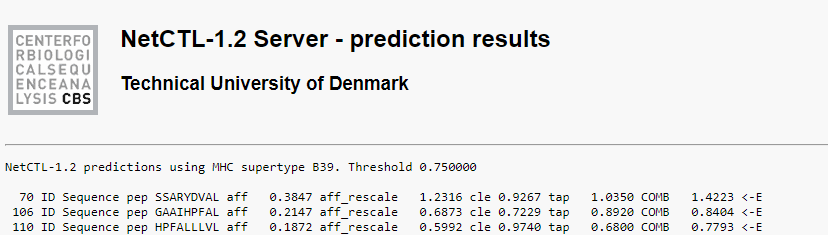

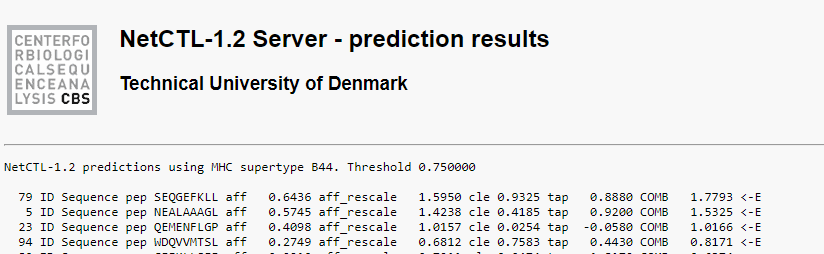

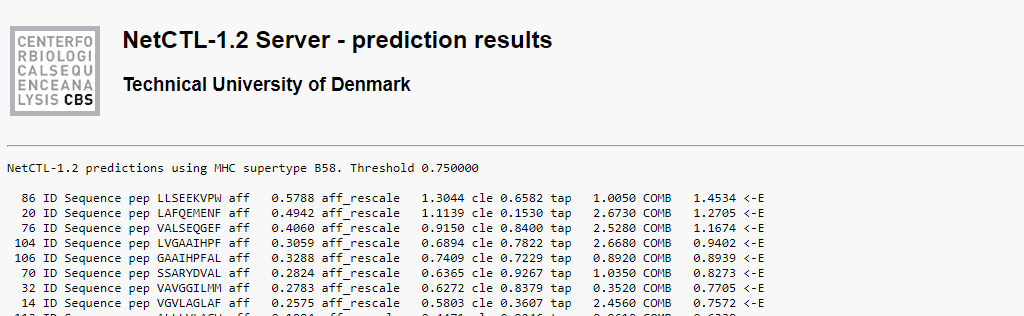

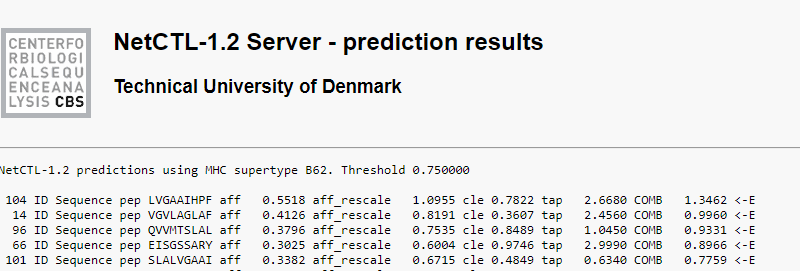

Supplement: Supplementary file 1 — Supplementary Information. [file 41598_2024_60680_MOESM1_ESM.zip › Yellow_Fever_data/2_Prediction of T-cell epitopes/NetCTL/NS2B/NetCTL-NS2B.docx]

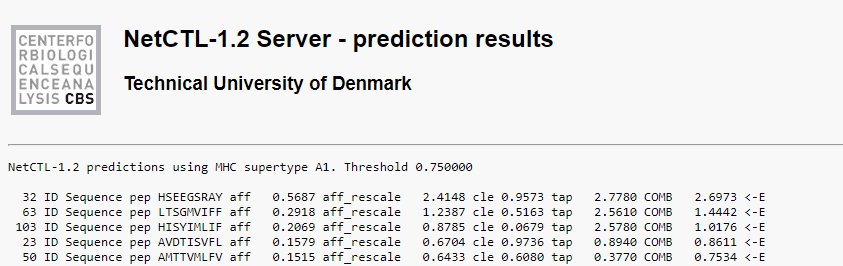

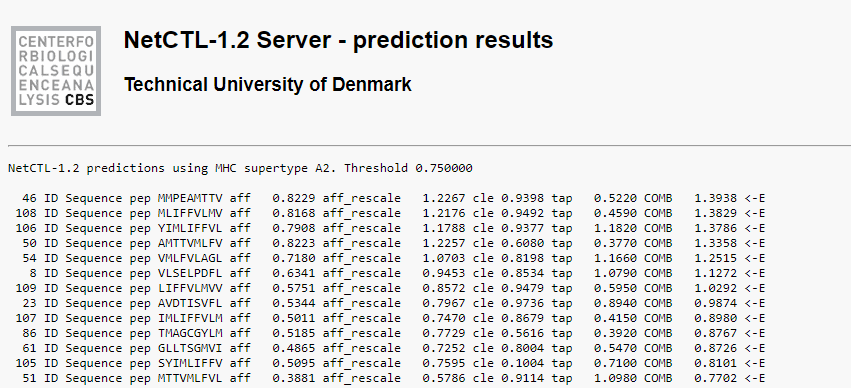

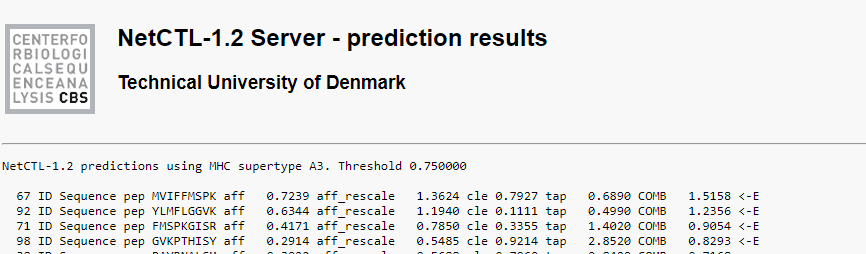

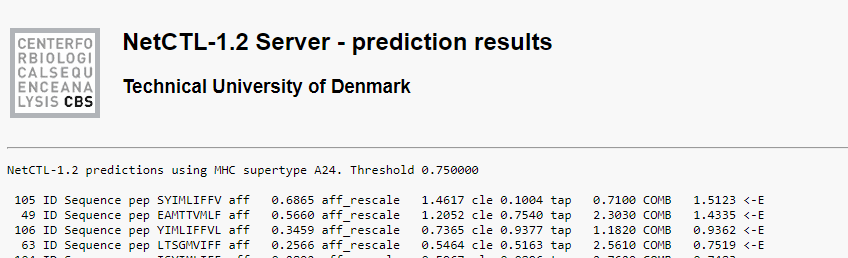

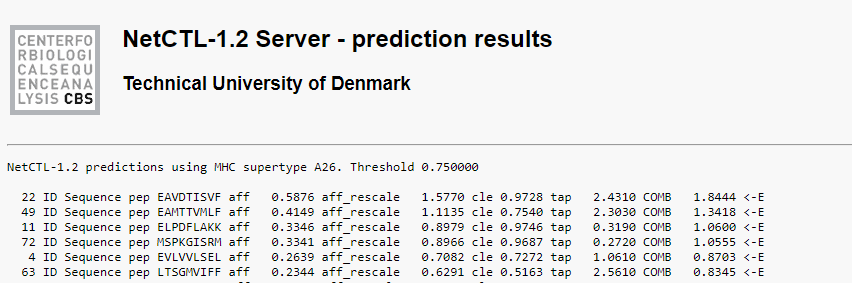

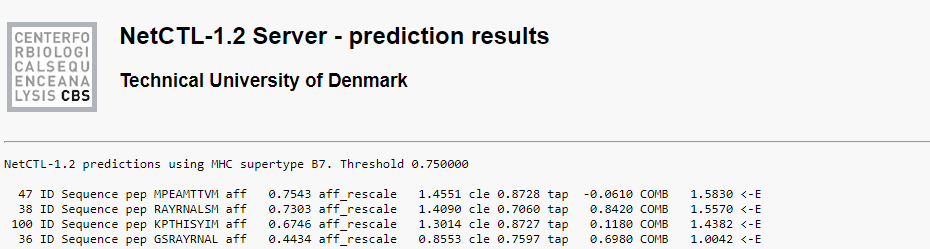

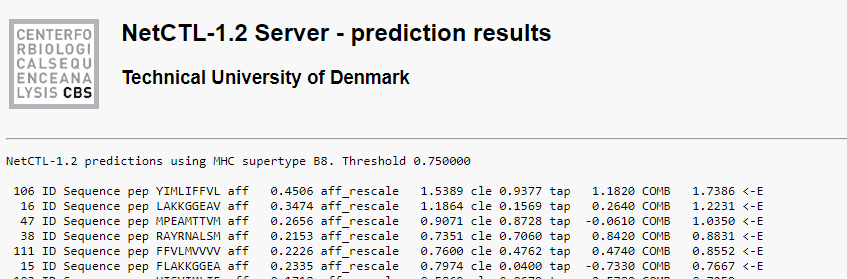

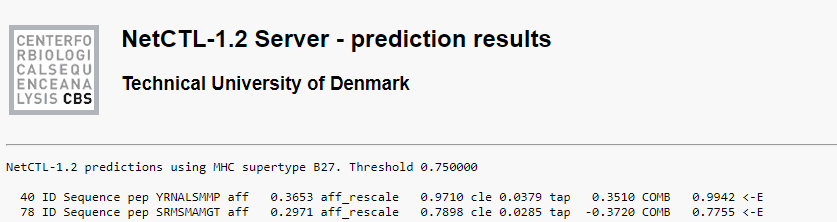

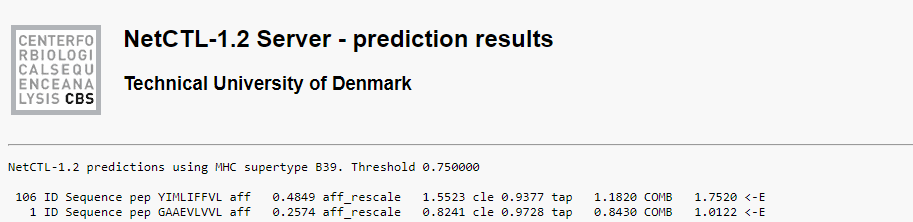

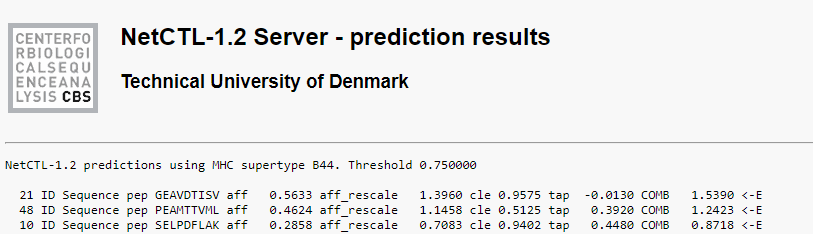

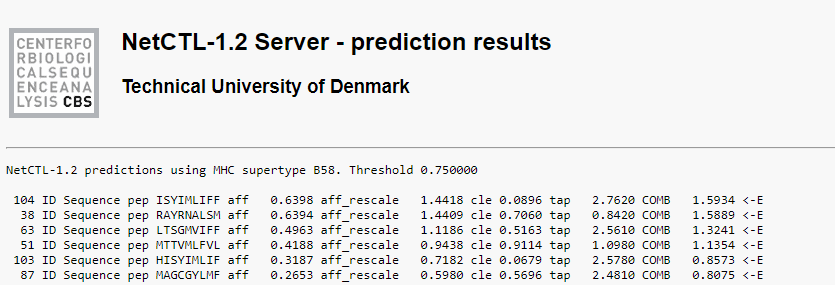

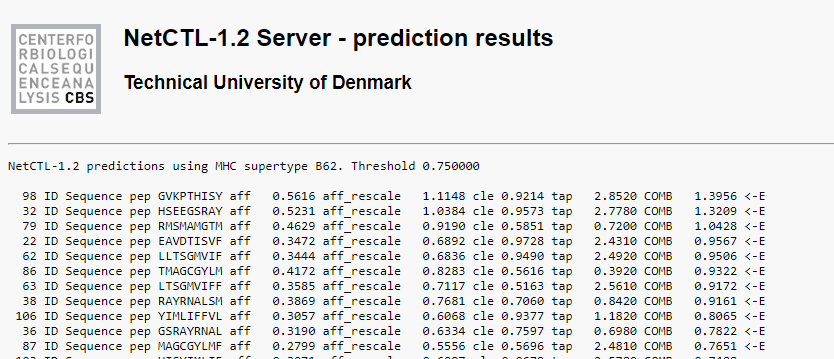

Supplement: Supplementary file 1 — Supplementary Information. [file 41598_2024_60680_MOESM1_ESM.zip › Yellow_Fever_data/2_Prediction of T-cell epitopes/NetCTL/NS4A/NetCTL-NS4A.docx]

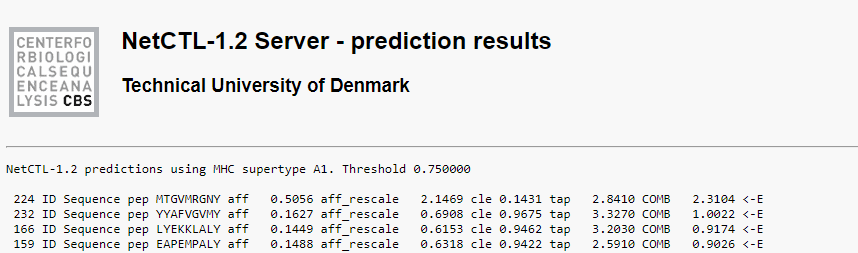

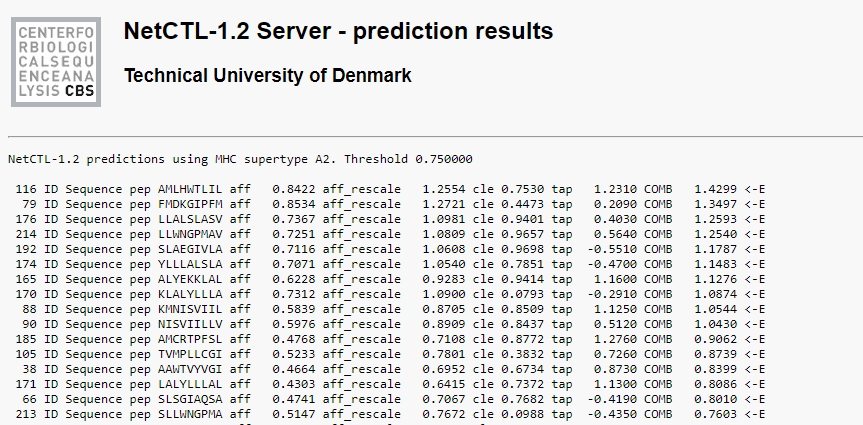

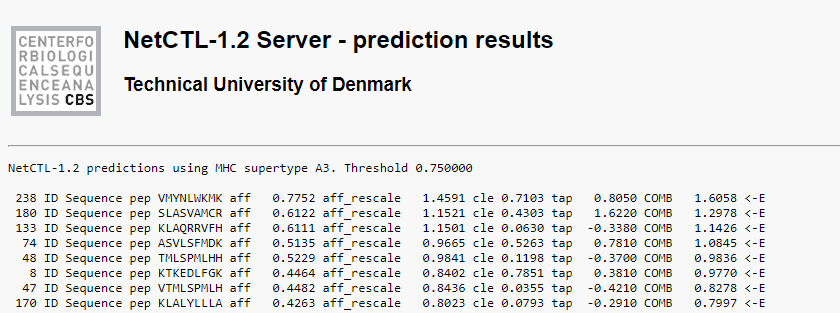

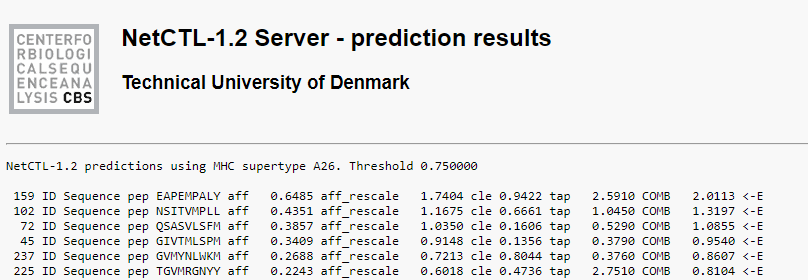

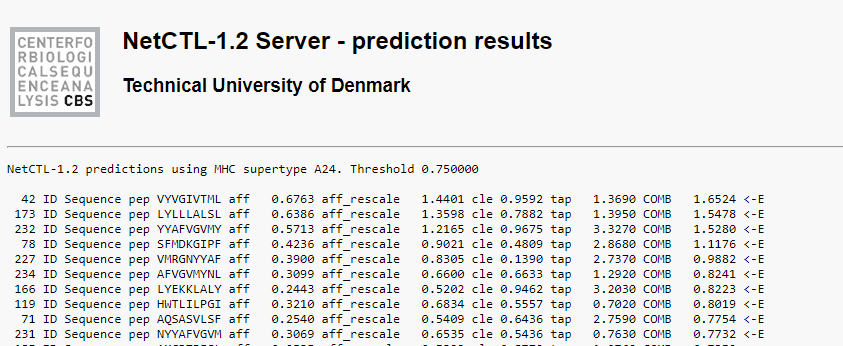

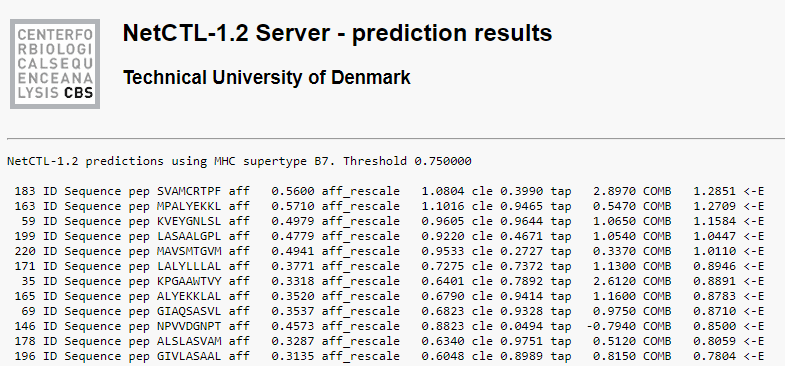

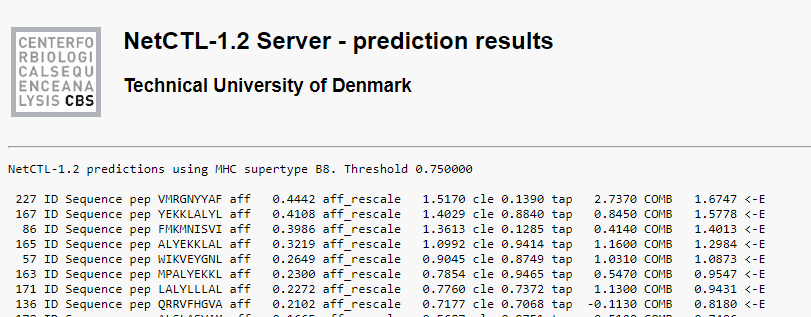

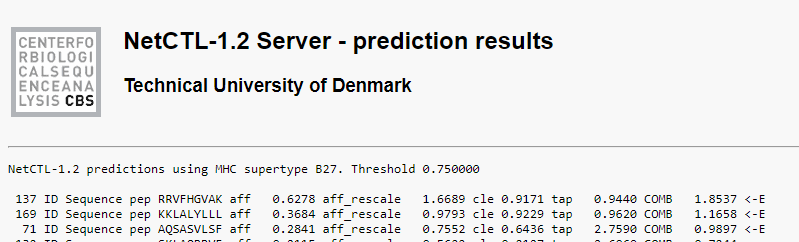

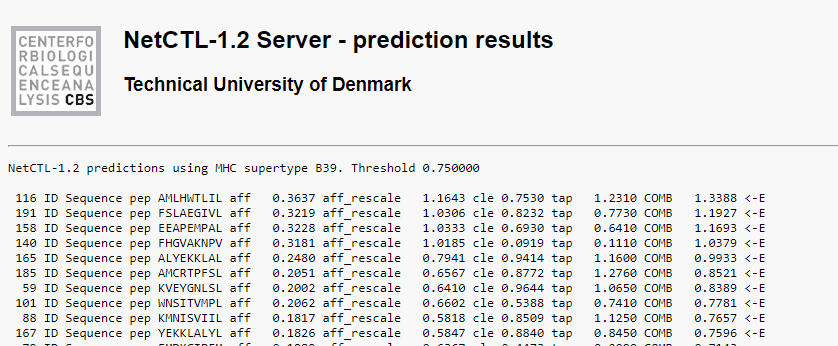

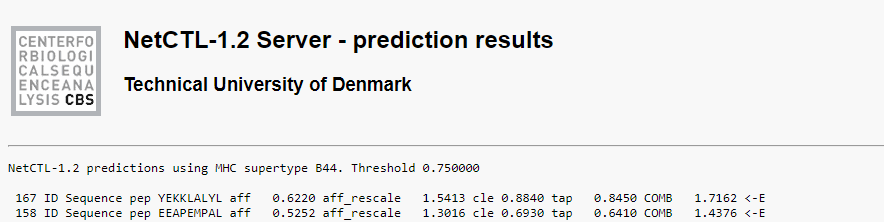

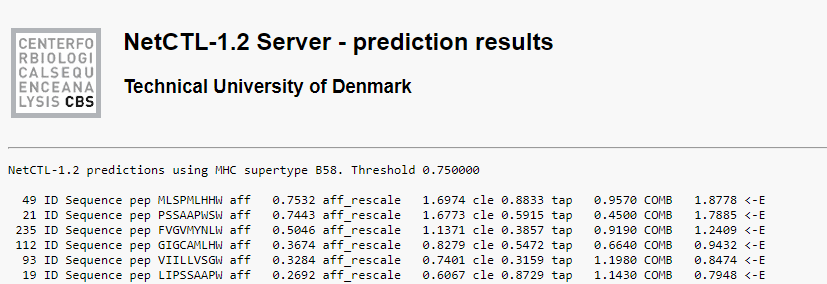

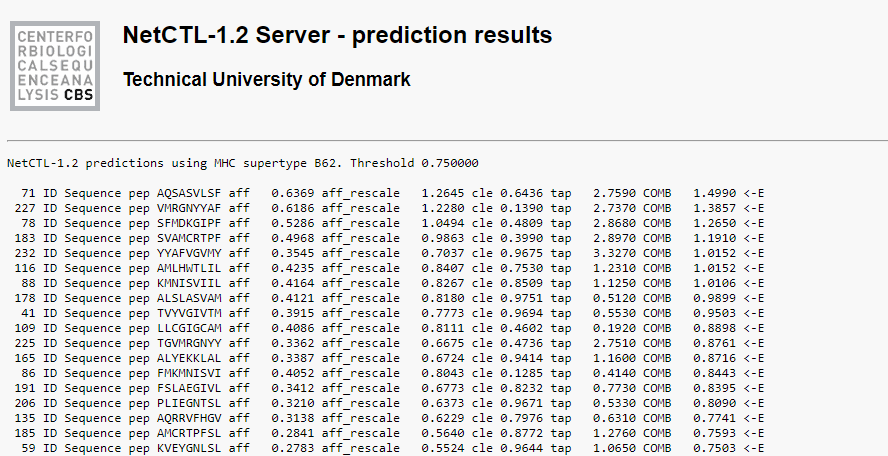

Supplement: Supplementary file 1 — Supplementary Information. [file 41598_2024_60680_MOESM1_ESM.zip › Yellow_Fever_data/2_Prediction of T-cell epitopes/NetCTL/NS4B/NetCTL-NS4B.docx]

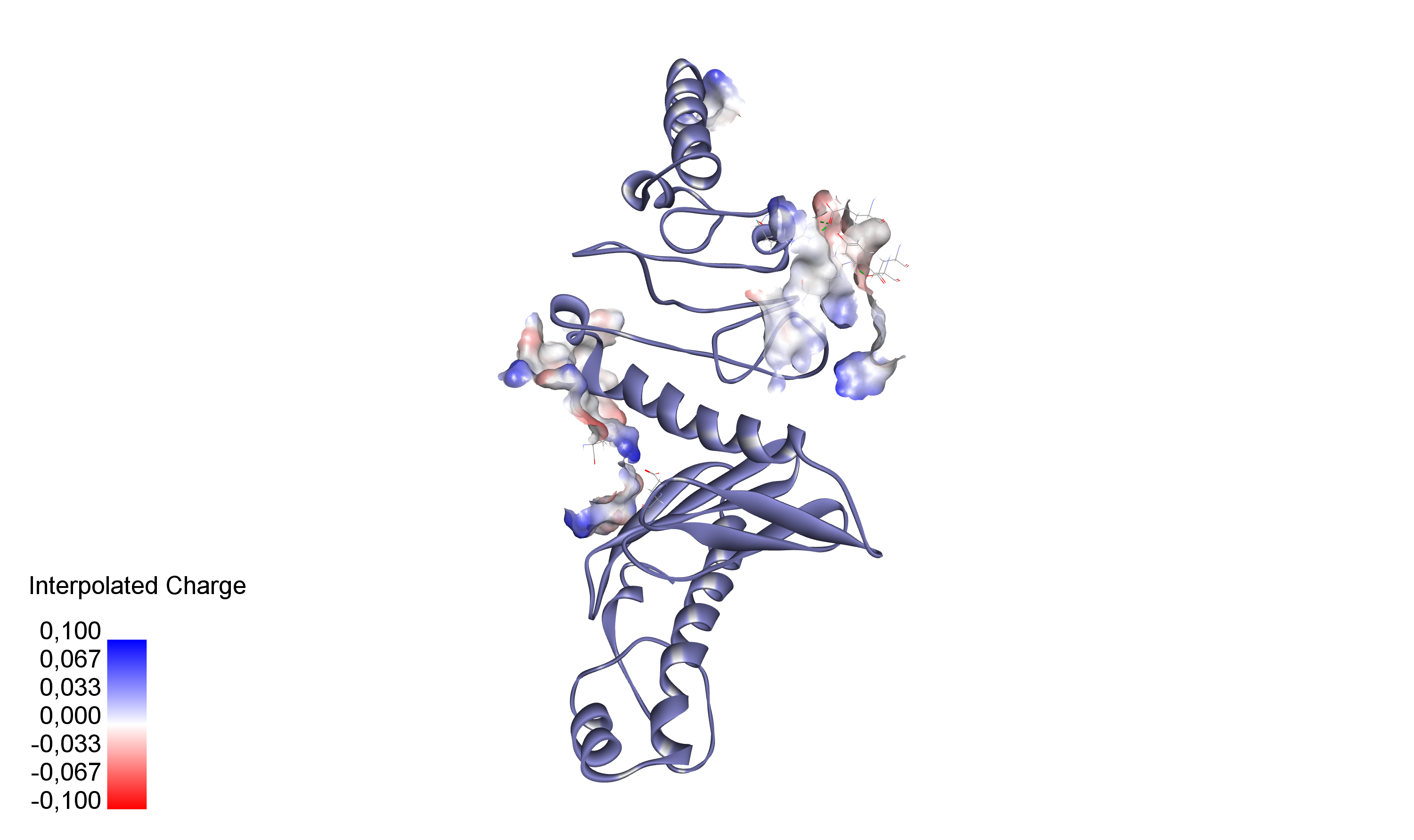

Supplement: Supplementary file 1 — Supplementary Information. [file 41598_2024_60680_MOESM1_ESM.zip › Yellow_Fever_data/6_Intermolecular_interactions/estruturalHADDOCK_YV_charge.bmp]

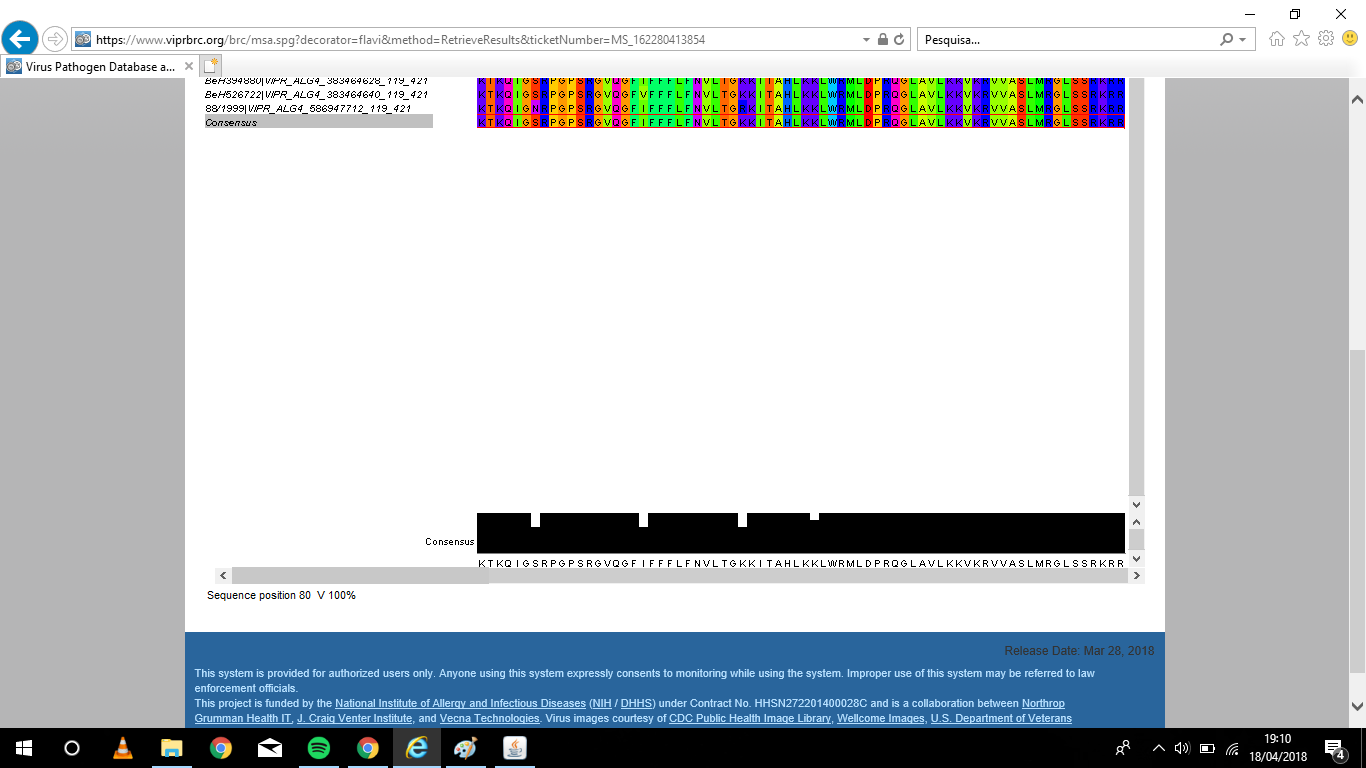

Supplement: Supplementary file 1 — Supplementary Information. [file 41598_2024_60680_MOESM1_ESM.zip › Yellow_Fever_data/1_Acquisition_proteins/Prints VIPR/C/passo 3.2 c consenso.png]

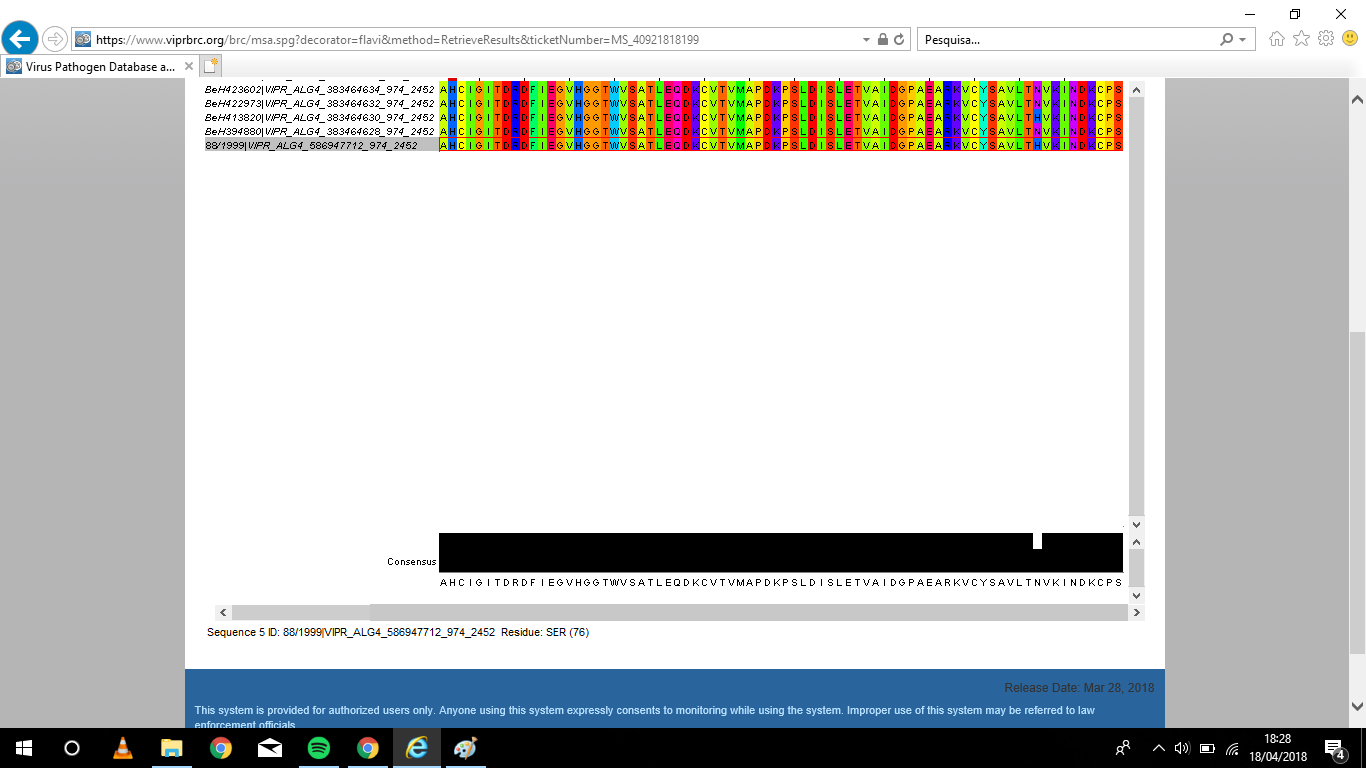

Supplement: Supplementary file 1 — Supplementary Information. [file 41598_2024_60680_MOESM1_ESM.zip › Yellow_Fever_data/1_Acquisition_proteins/Prints VIPR/E/passo 3.1 e consenso.png]

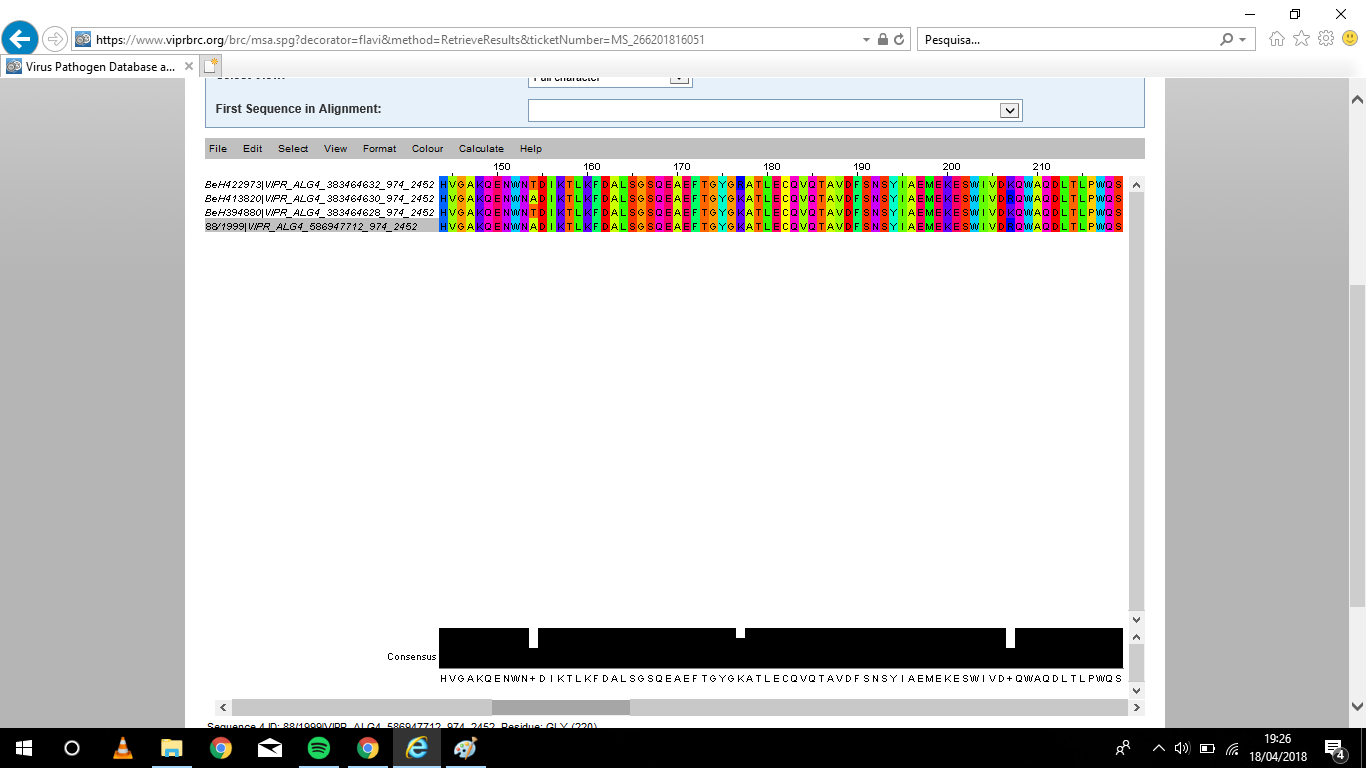

Supplement: Supplementary file 1 — Supplementary Information. [file 41598_2024_60680_MOESM1_ESM.zip › Yellow_Fever_data/1_Acquisition_proteins/Prints VIPR/E/passo 3.3 e consenso.png]

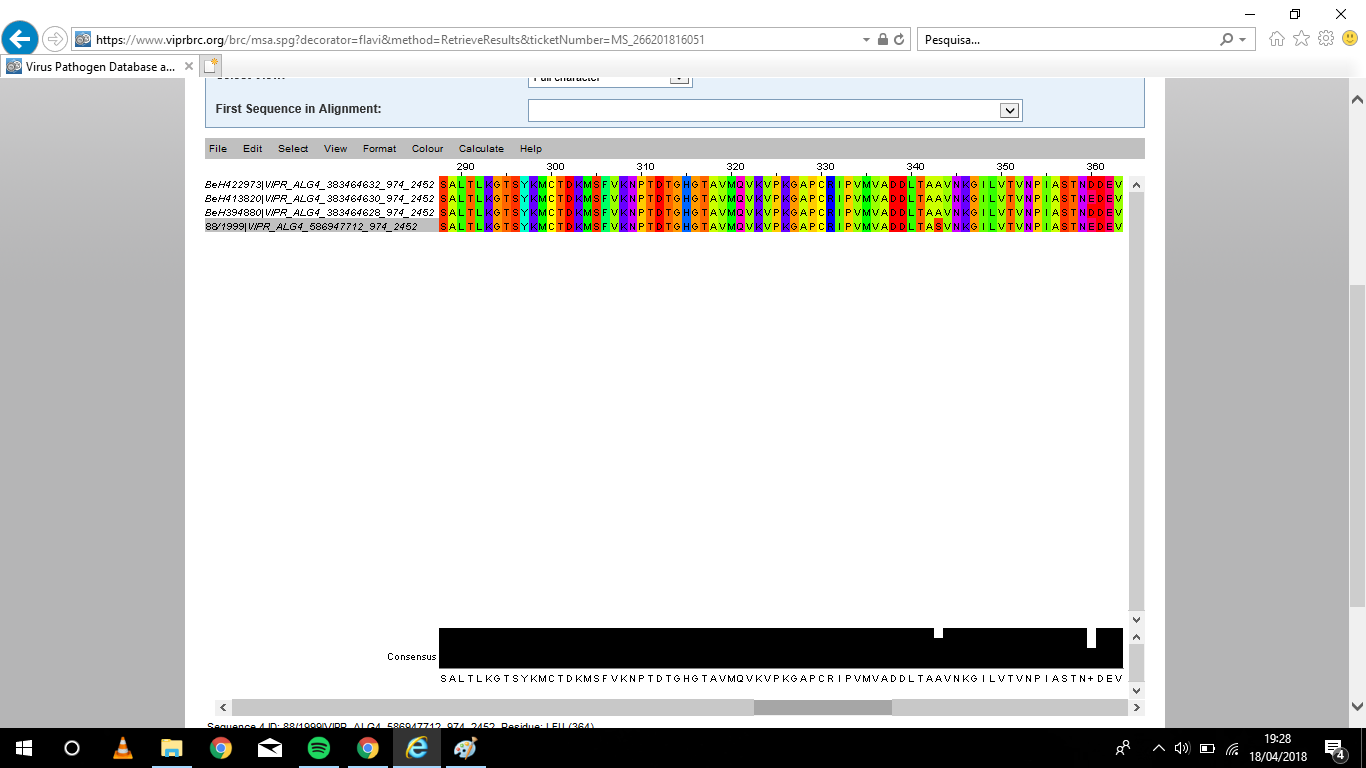

Supplement: Supplementary file 1 — Supplementary Information. [file 41598_2024_60680_MOESM1_ESM.zip › Yellow_Fever_data/1_Acquisition_proteins/Prints VIPR/E/passo 3.5 e consenso.png]

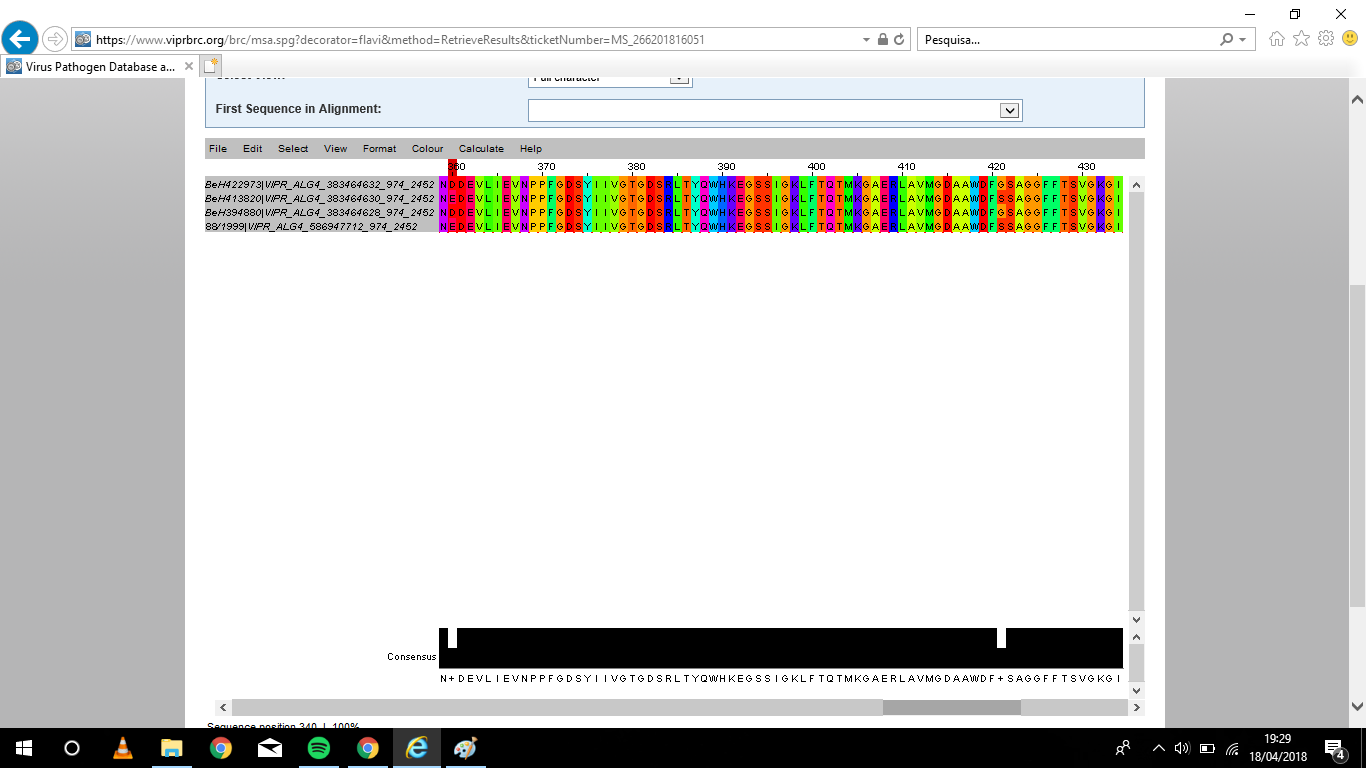

Supplement: Supplementary file 1 — Supplementary Information. [file 41598_2024_60680_MOESM1_ESM.zip › Yellow_Fever_data/1_Acquisition_proteins/Prints VIPR/E/passo 3.6 e consenso.png]

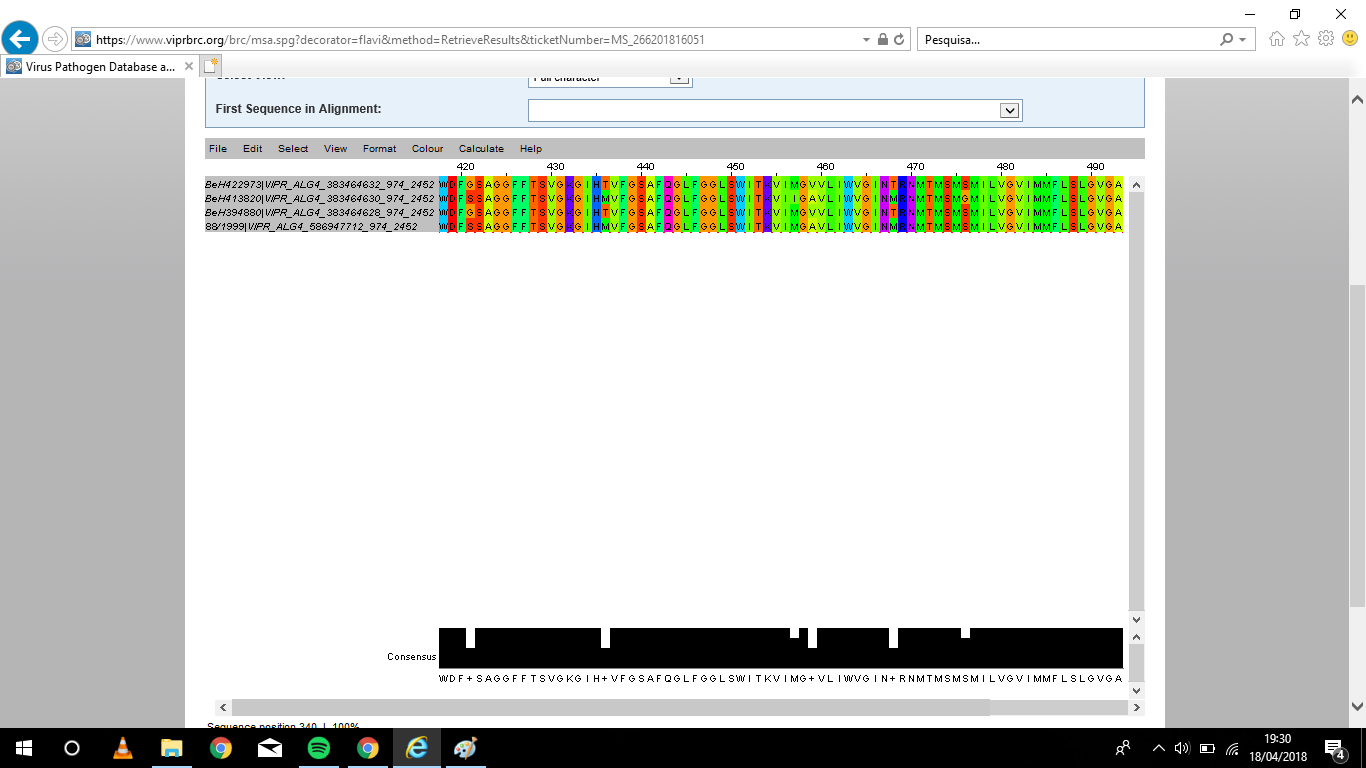

Supplement: Supplementary file 1 — Supplementary Information. [file 41598_2024_60680_MOESM1_ESM.zip › Yellow_Fever_data/1_Acquisition_proteins/Prints VIPR/E/passo 3.7 e consenso.png]

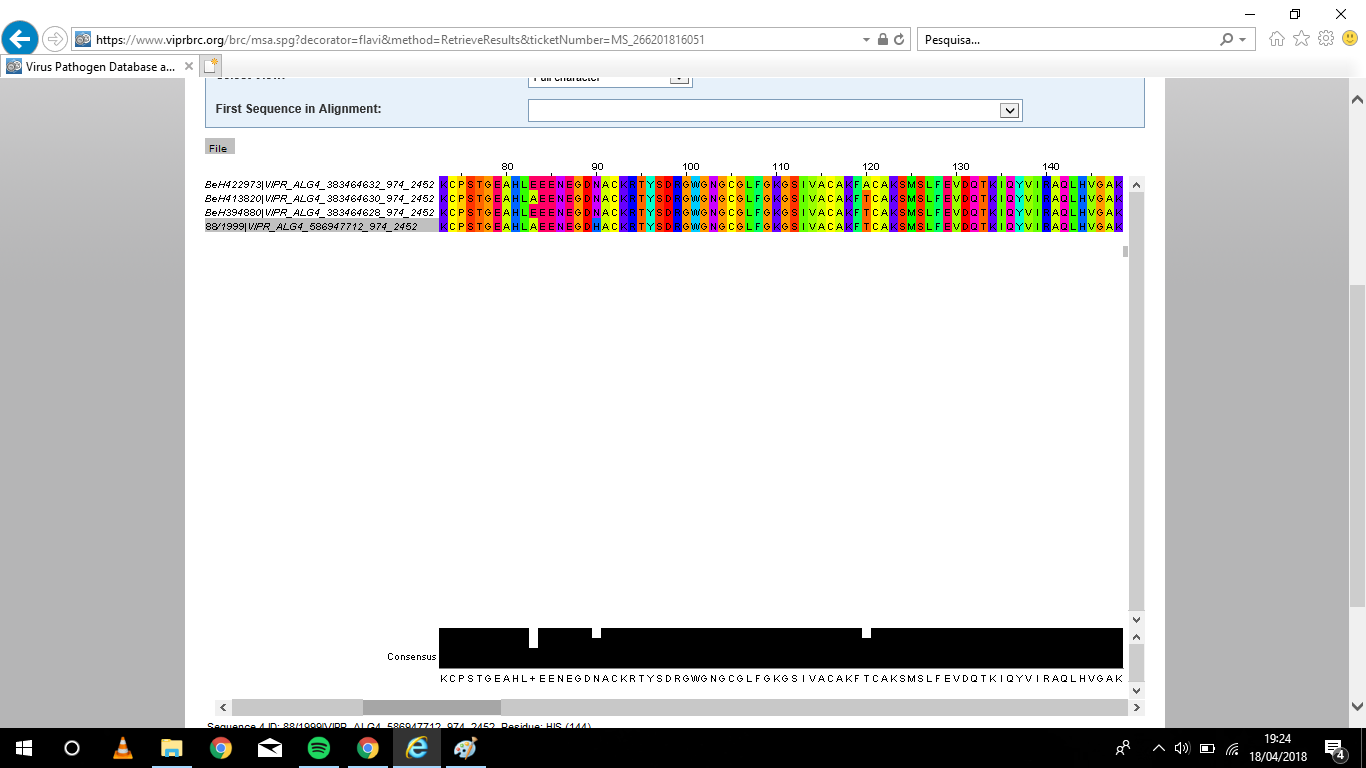

Supplement: Supplementary file 1 — Supplementary Information. [file 41598_2024_60680_MOESM1_ESM.zip › Yellow_Fever_data/1_Acquisition_proteins/Prints VIPR/E/passo 3.2 e consenso.png]

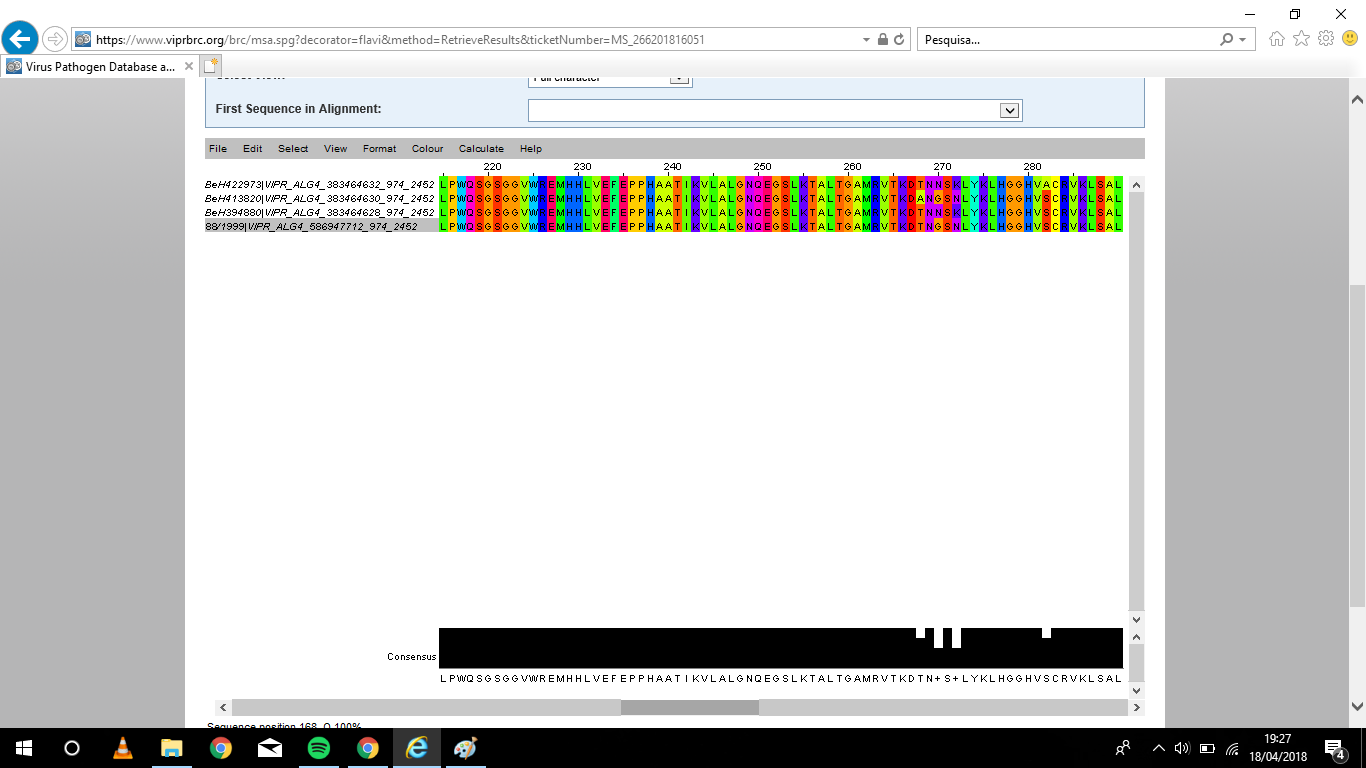

Supplement: Supplementary file 1 — Supplementary Information. [file 41598_2024_60680_MOESM1_ESM.zip › Yellow_Fever_data/1_Acquisition_proteins/Prints VIPR/E/passo 3.4 e consenso.png]

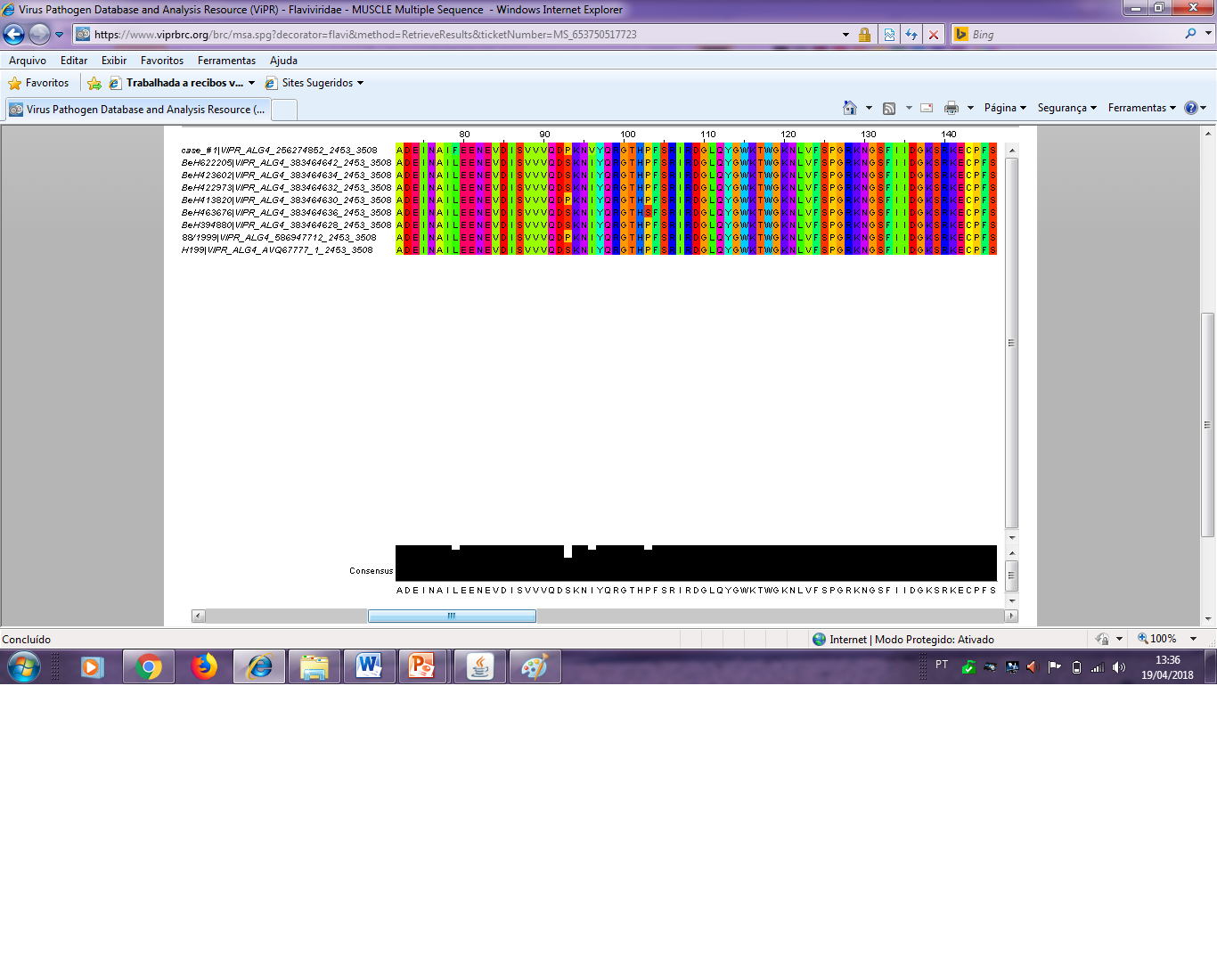

Supplement: Supplementary file 1 — Supplementary Information. [file 41598_2024_60680_MOESM1_ESM.zip › Yellow_Fever_data/1_Acquisition_proteins/Prints VIPR/ns1/passo 3.2 consenso.png]

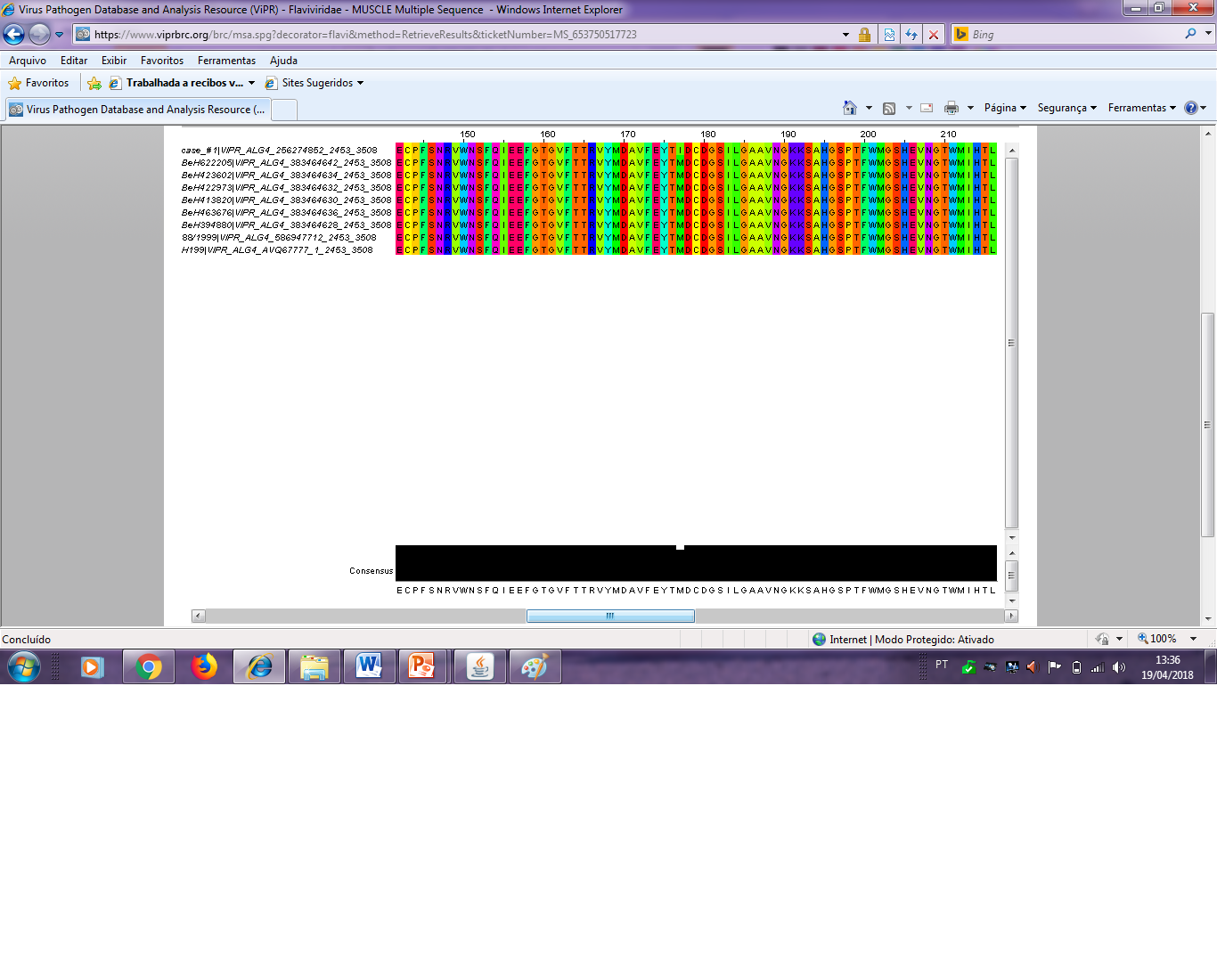

Supplement: Supplementary file 1 — Supplementary Information. [file 41598_2024_60680_MOESM1_ESM.zip › Yellow_Fever_data/1_Acquisition_proteins/Prints VIPR/ns1/passo 3.3 consenso.png]

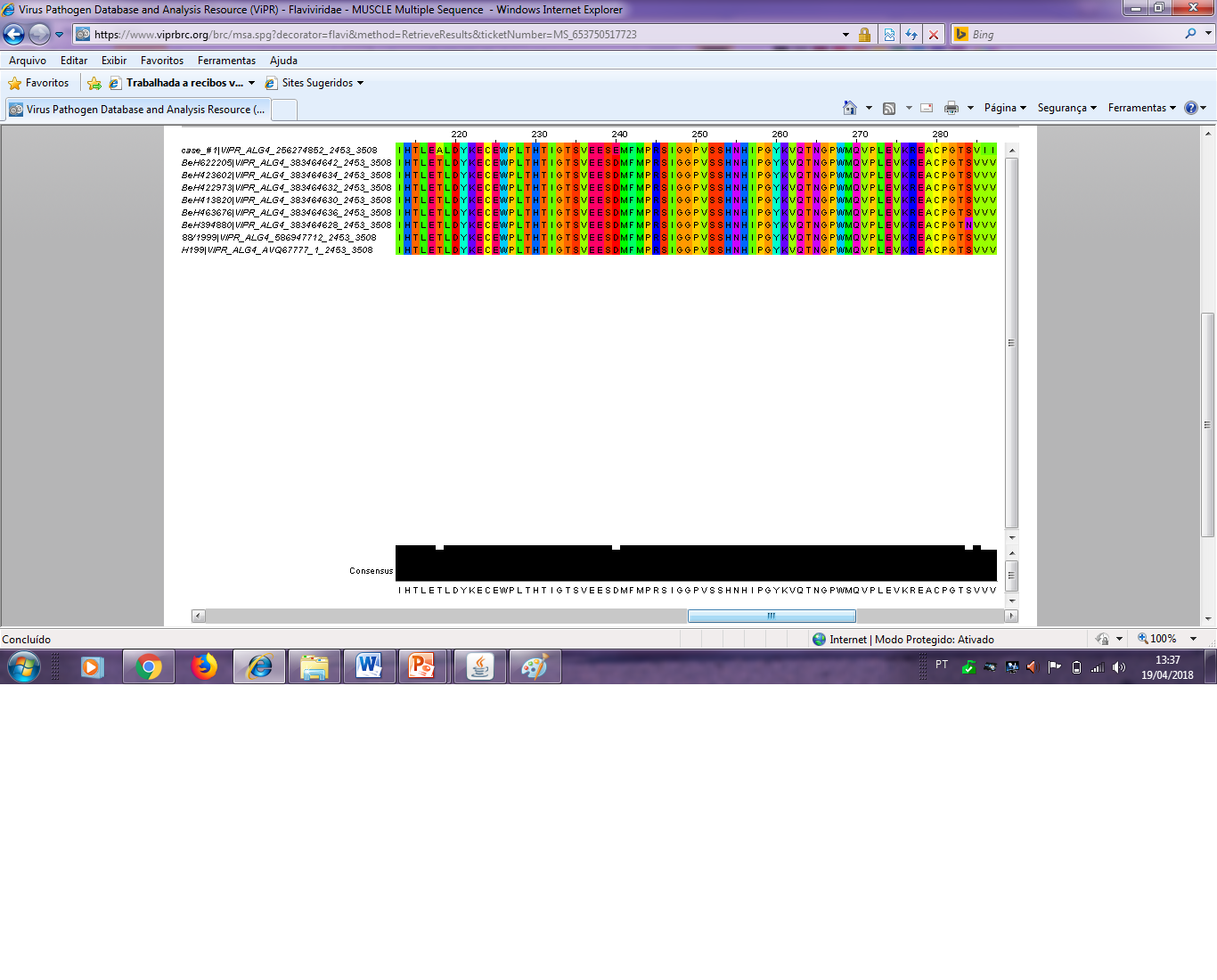

Supplement: Supplementary file 1 — Supplementary Information. [file 41598_2024_60680_MOESM1_ESM.zip › Yellow_Fever_data/1_Acquisition_proteins/Prints VIPR/ns1/passo 3.4 consenso.png]

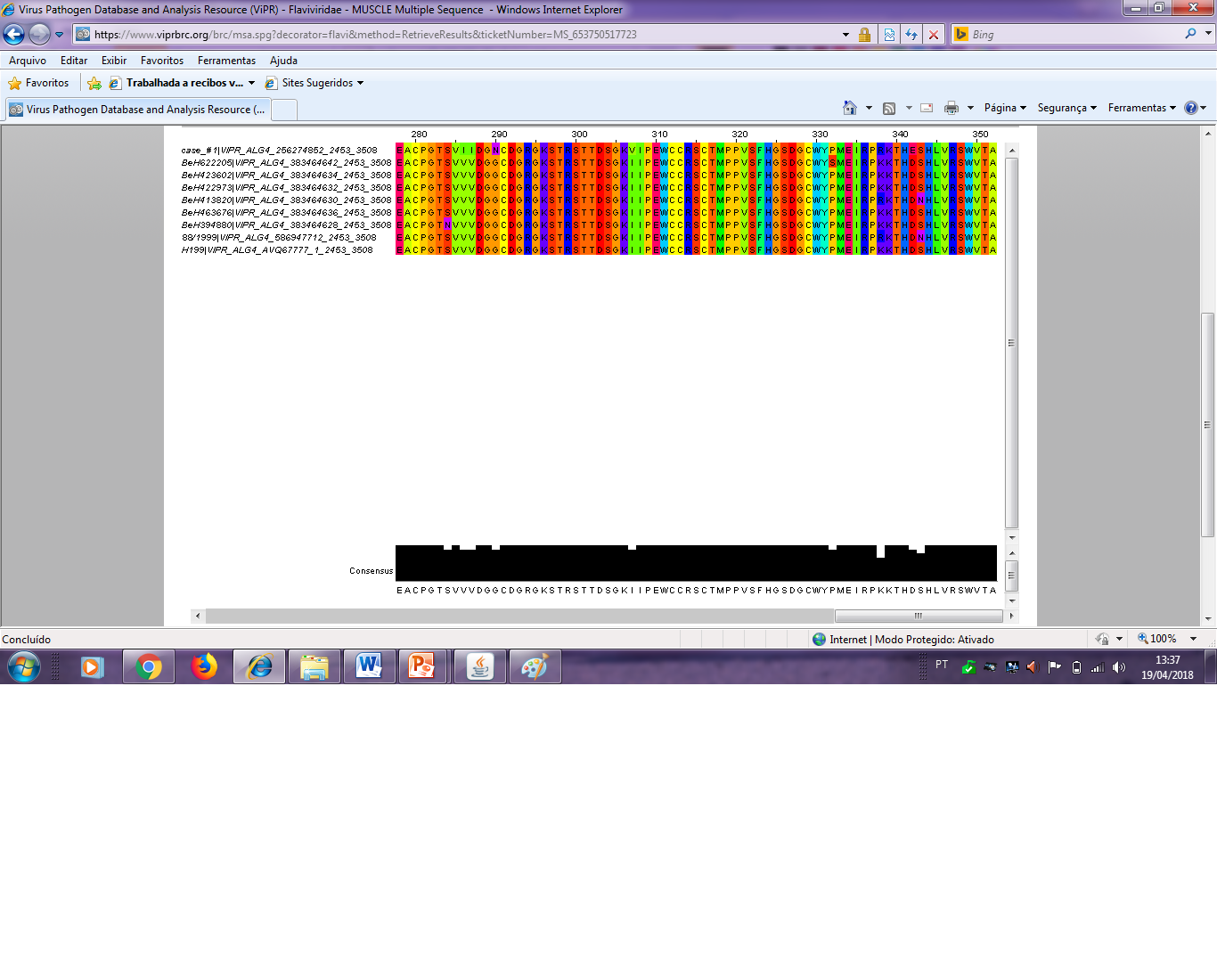

Supplement: Supplementary file 1 — Supplementary Information. [file 41598_2024_60680_MOESM1_ESM.zip › Yellow_Fever_data/1_Acquisition_proteins/Prints VIPR/ns1/passo 3.5 consenso.png]

Proteina M


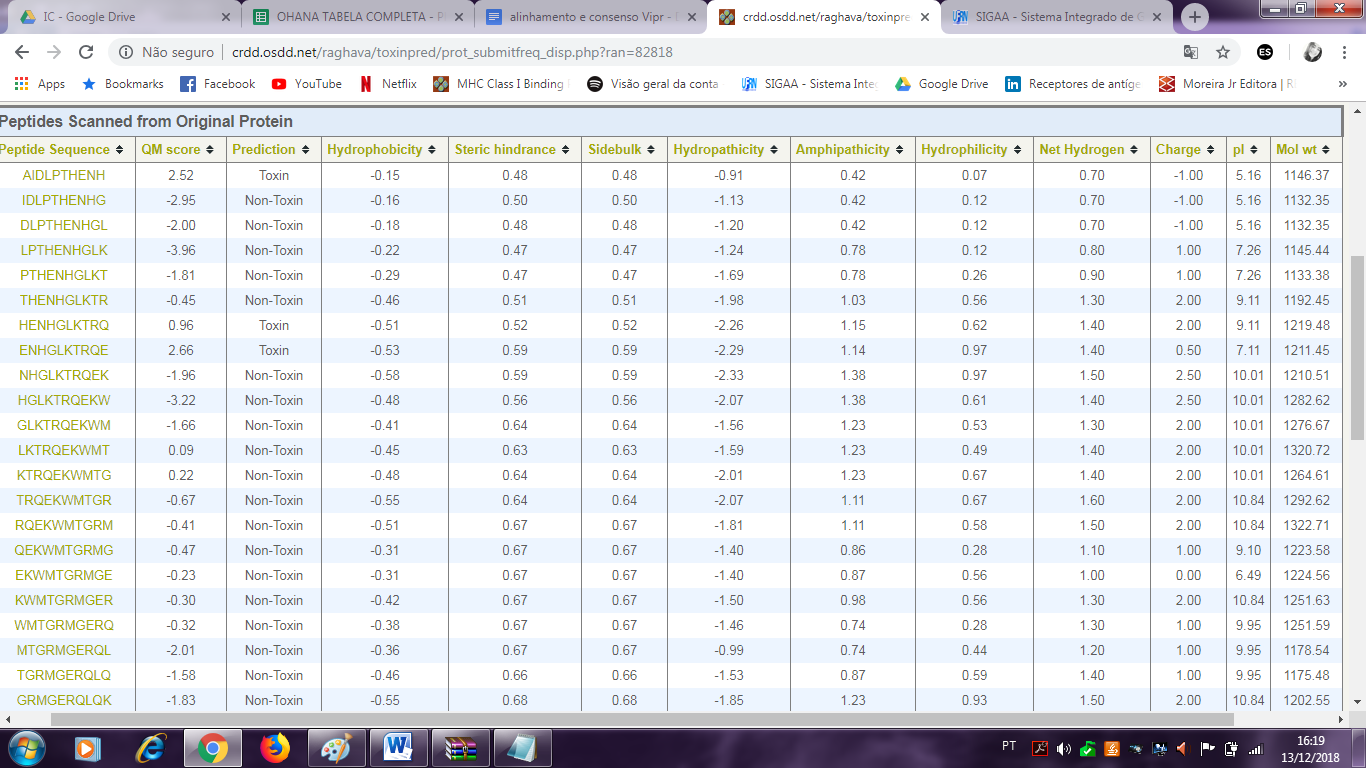


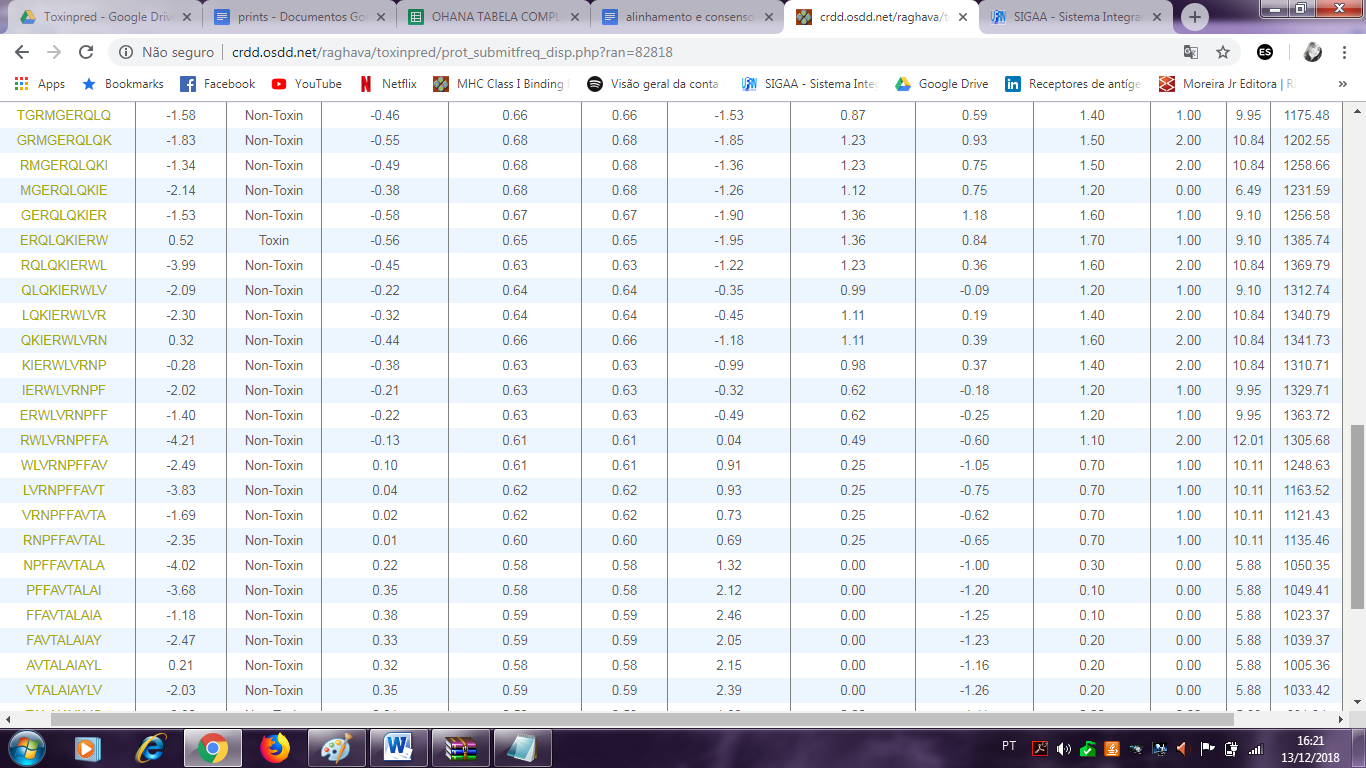


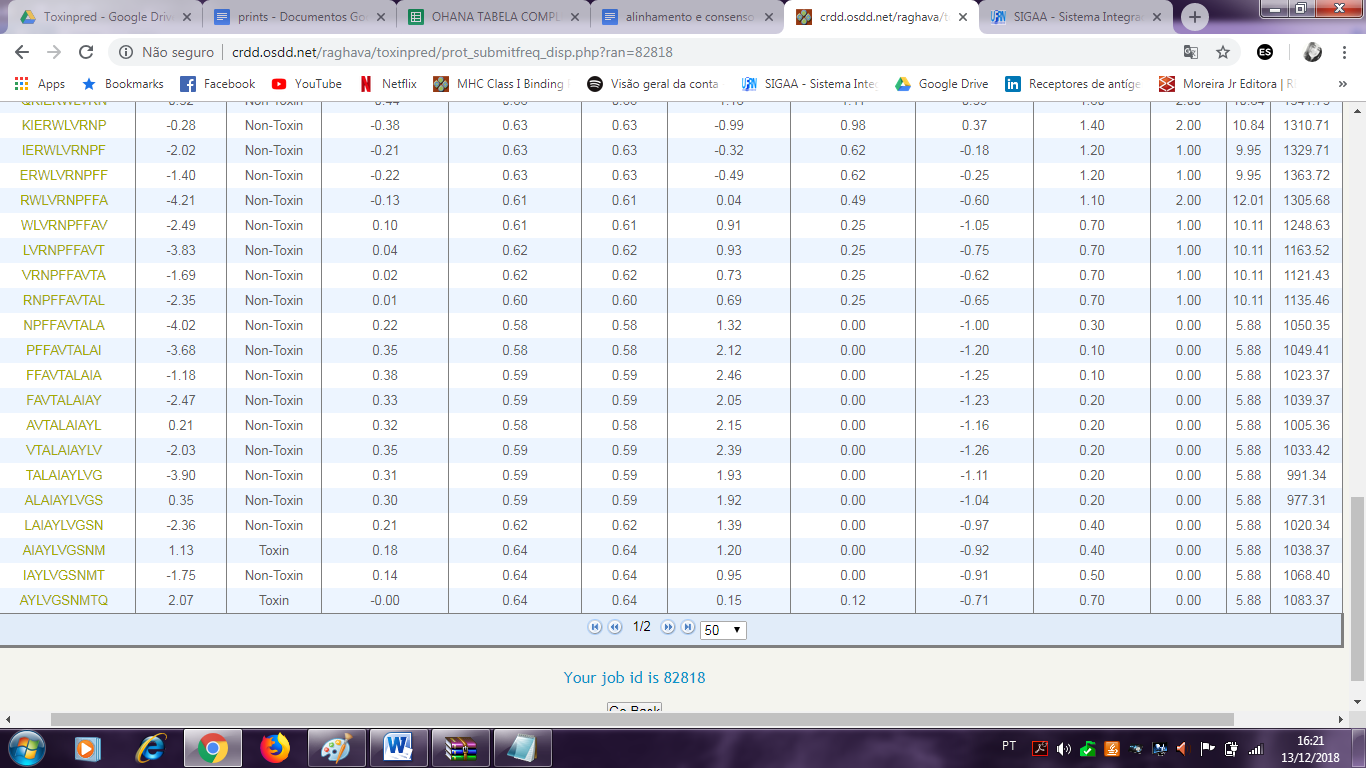


E


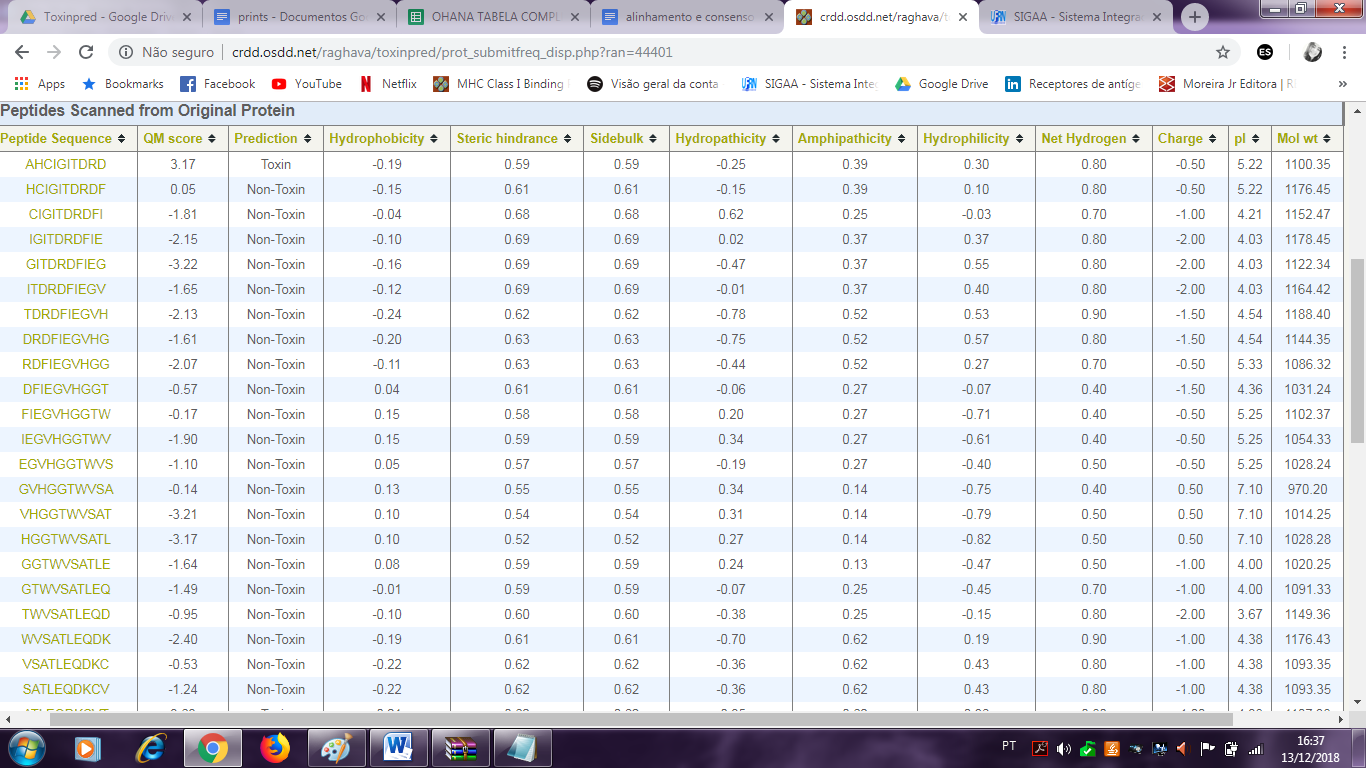


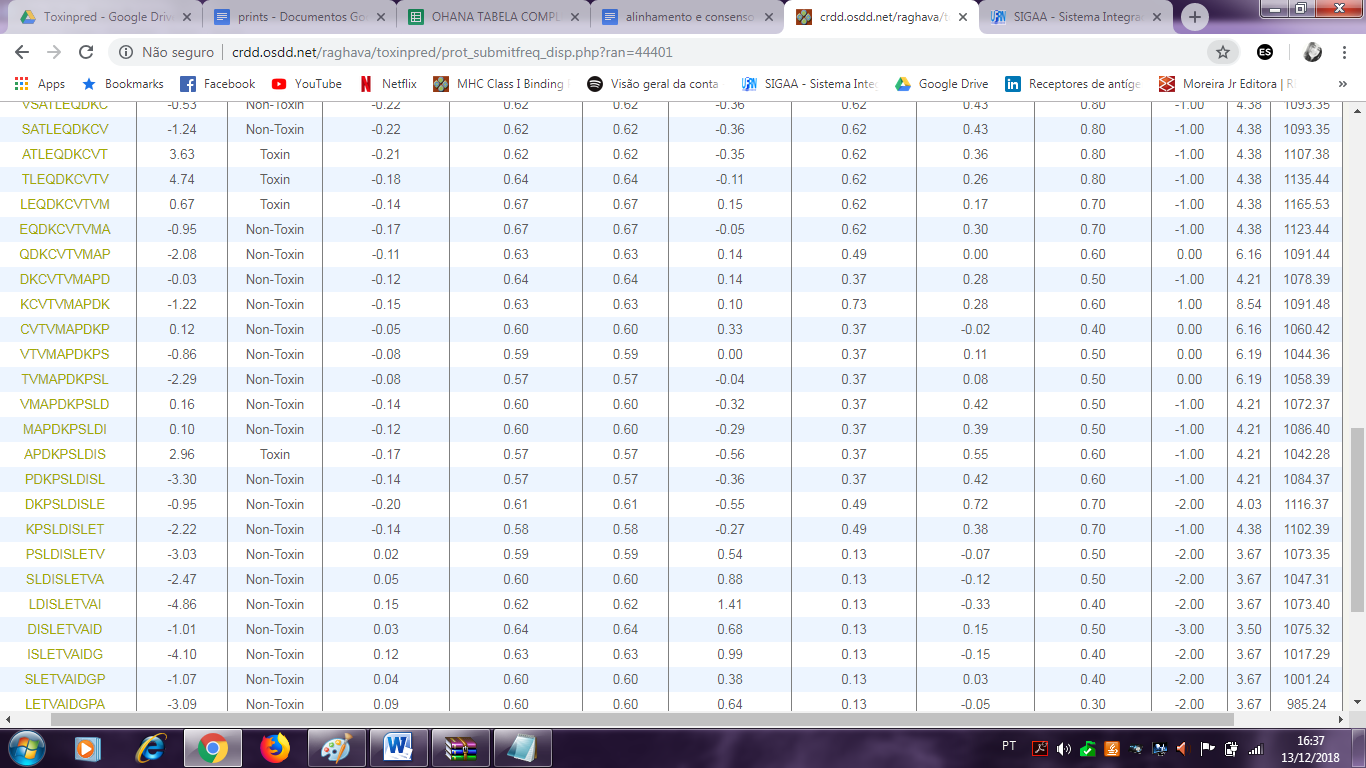


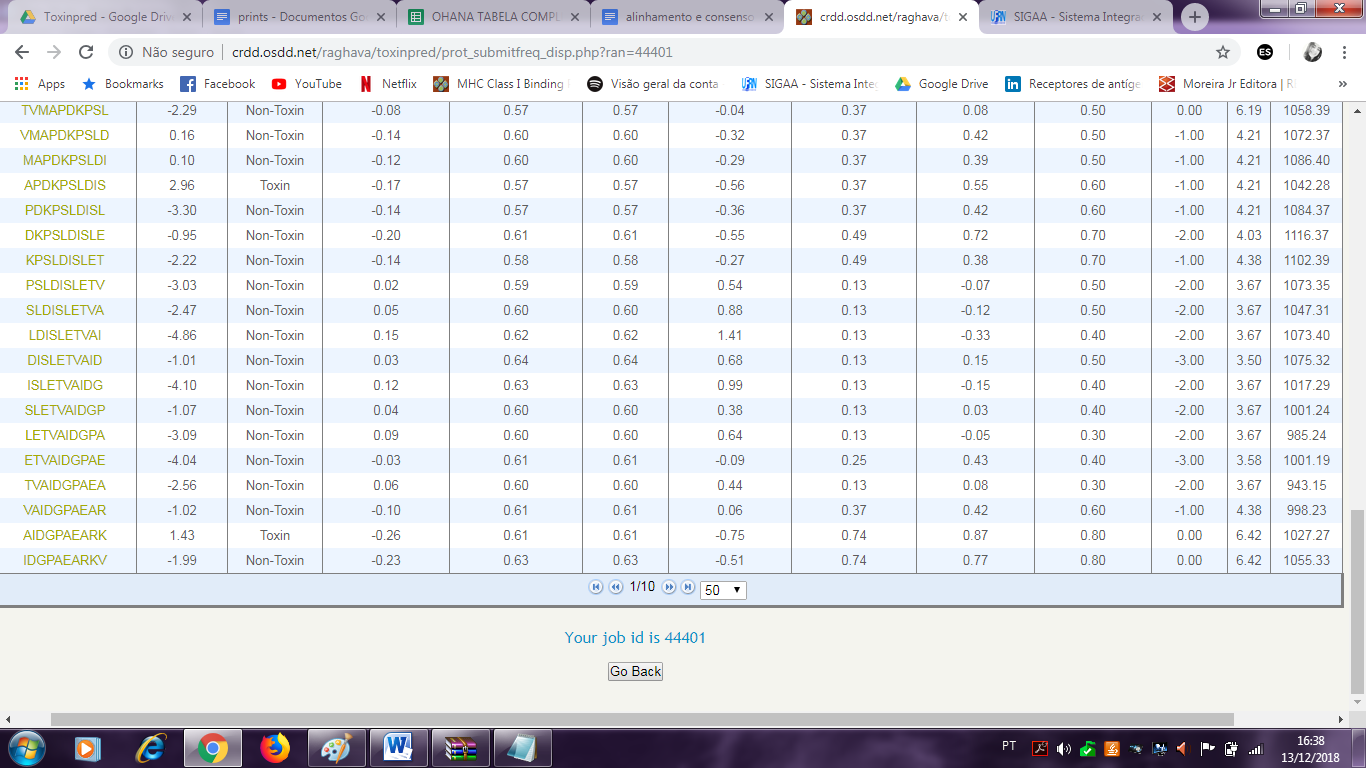


NS2A


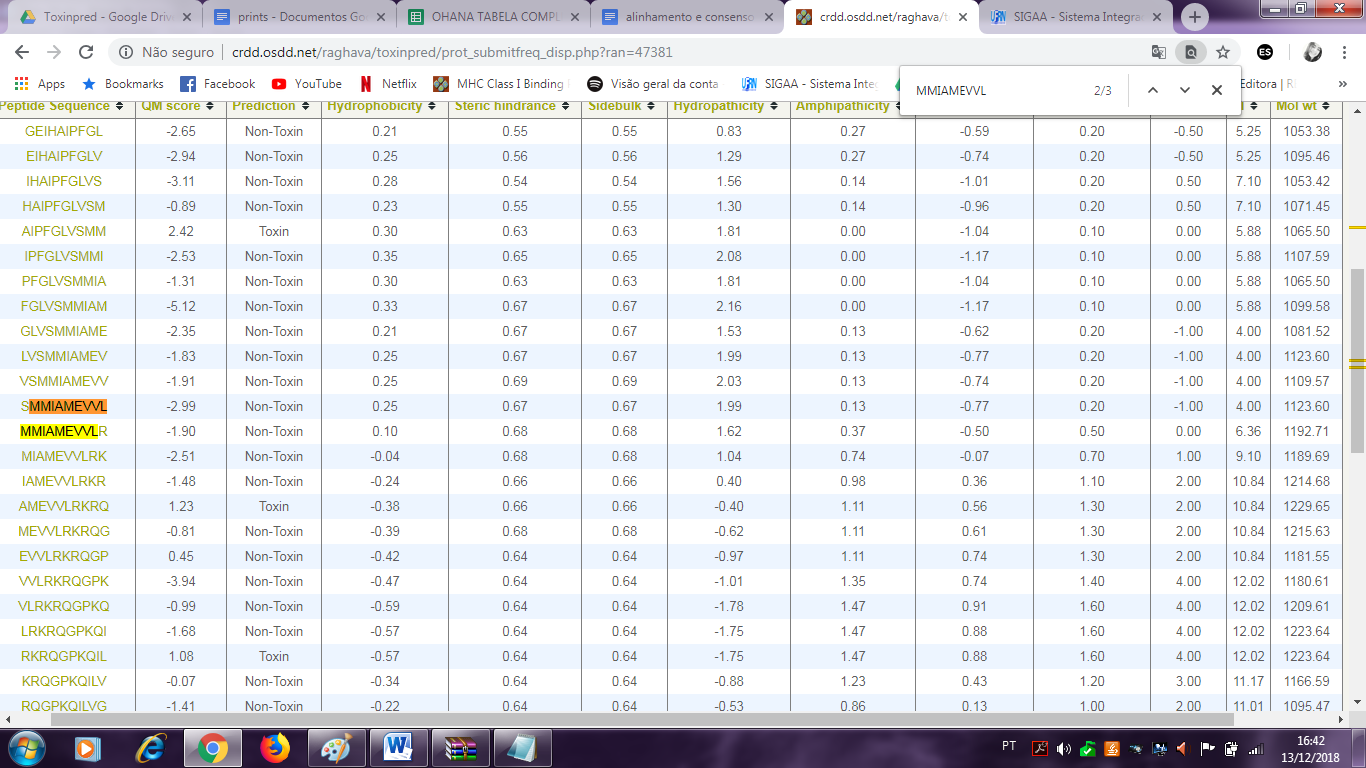


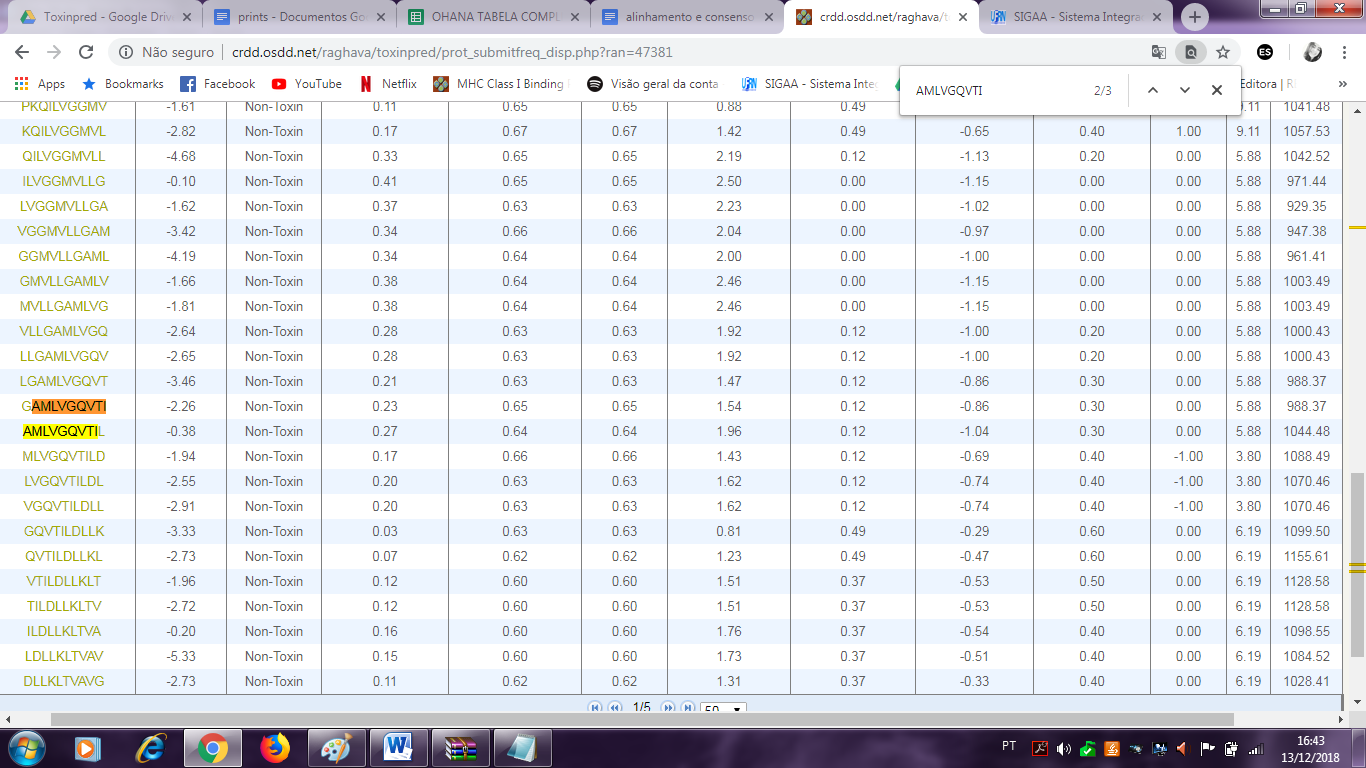


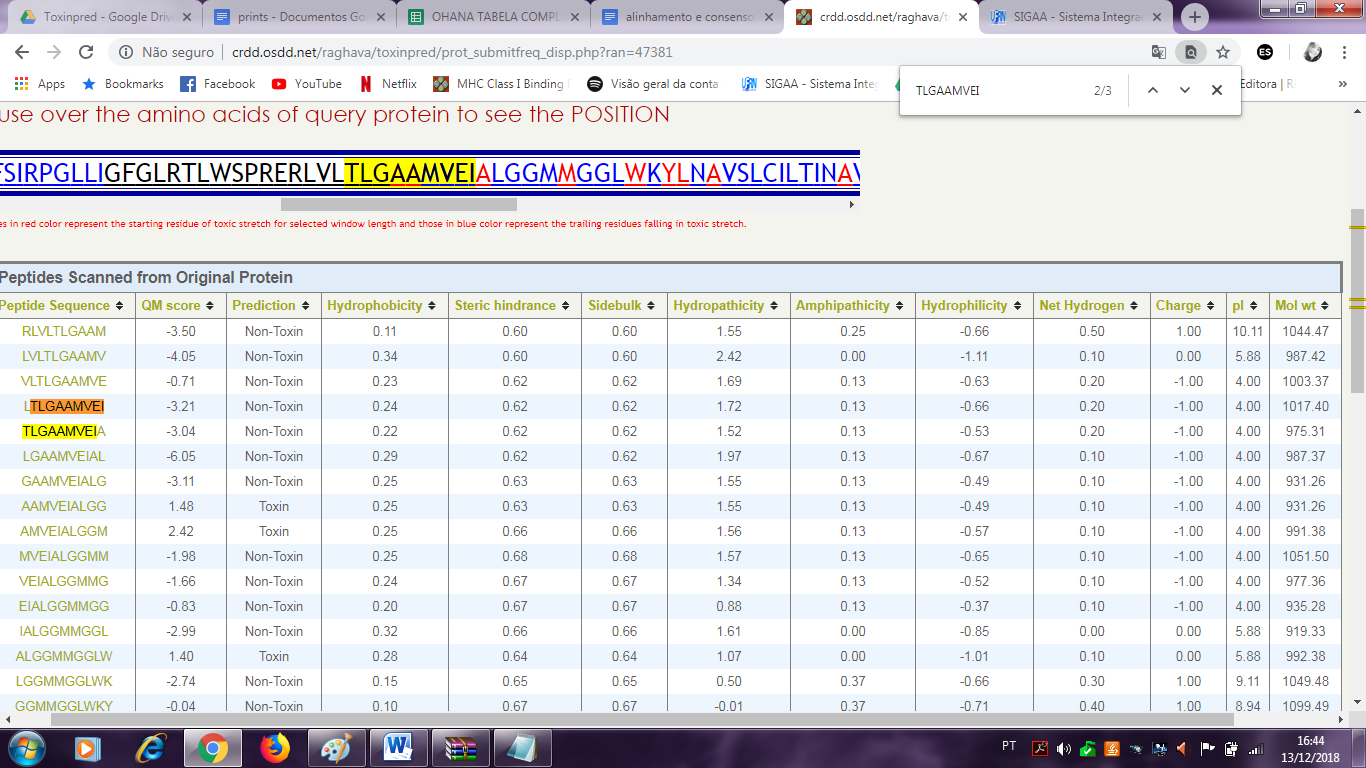


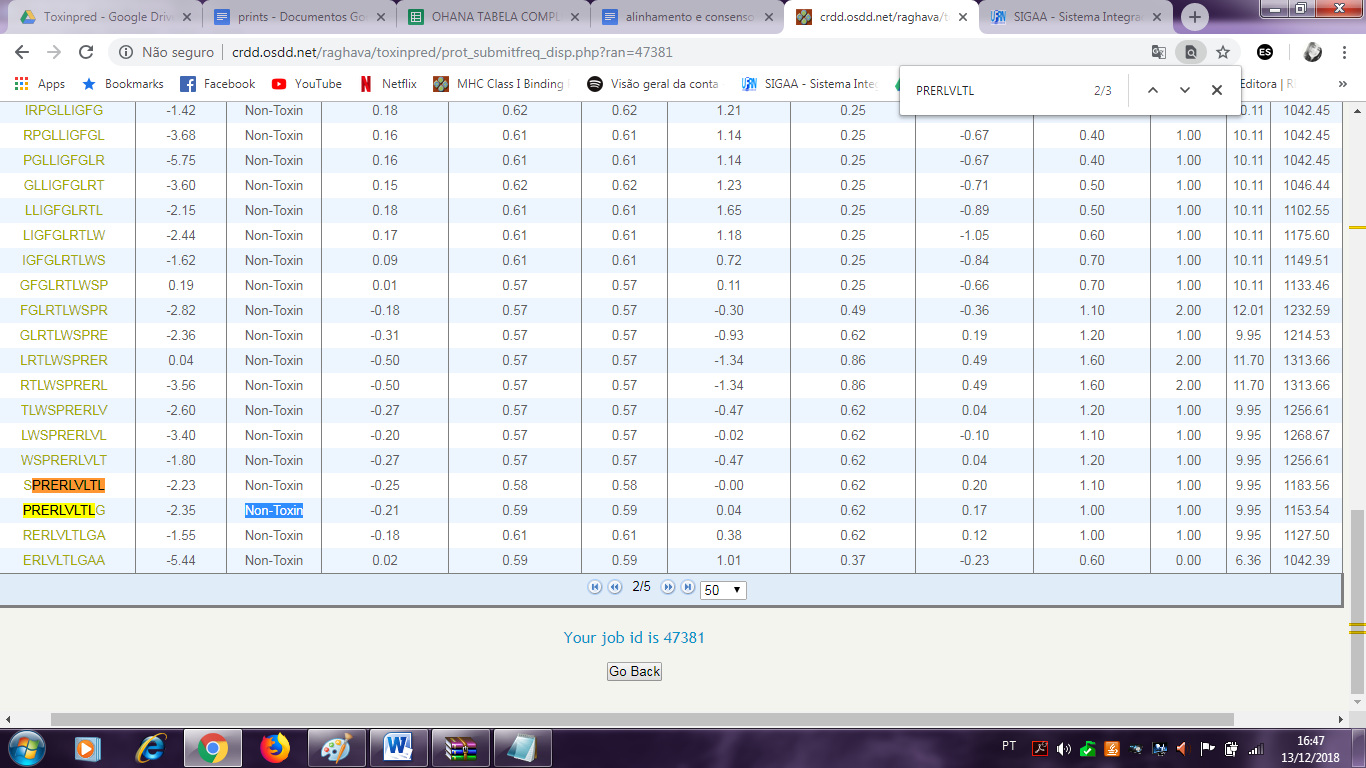


NS2B


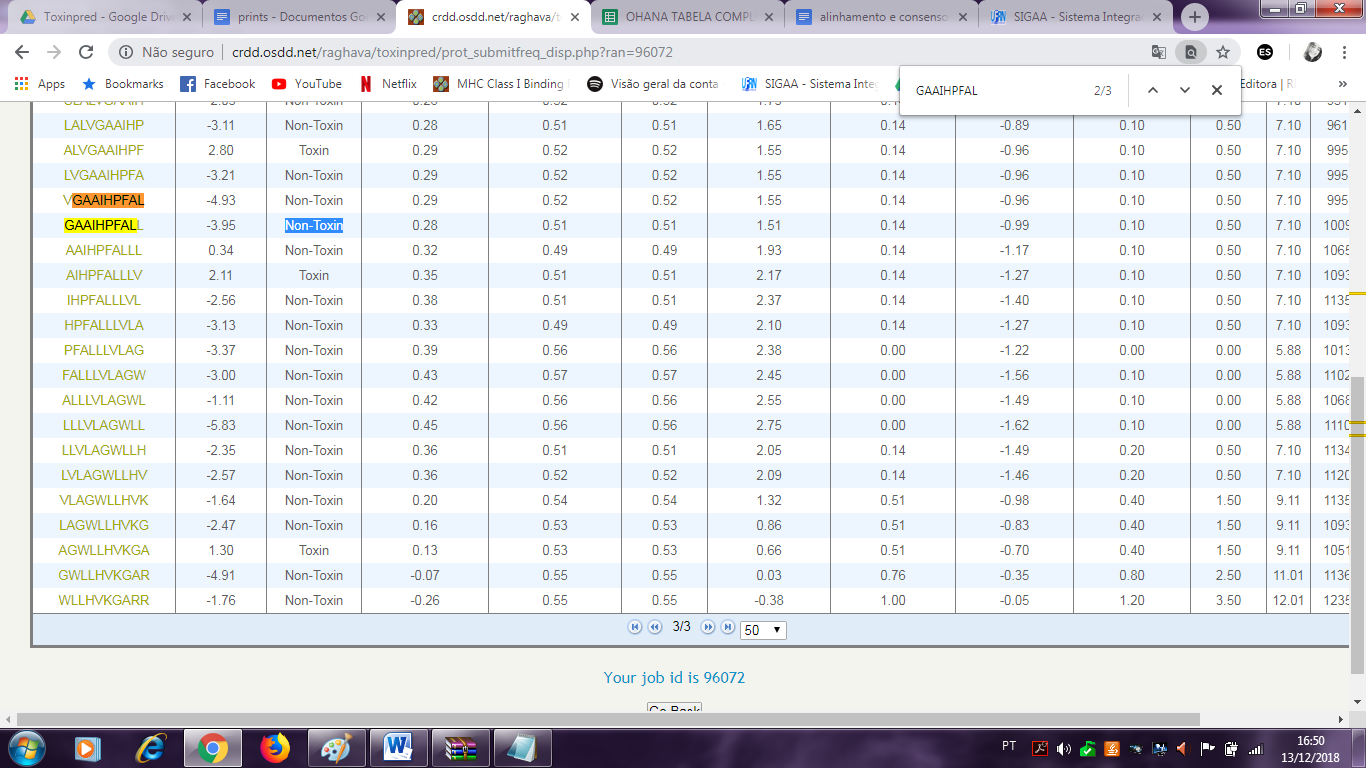


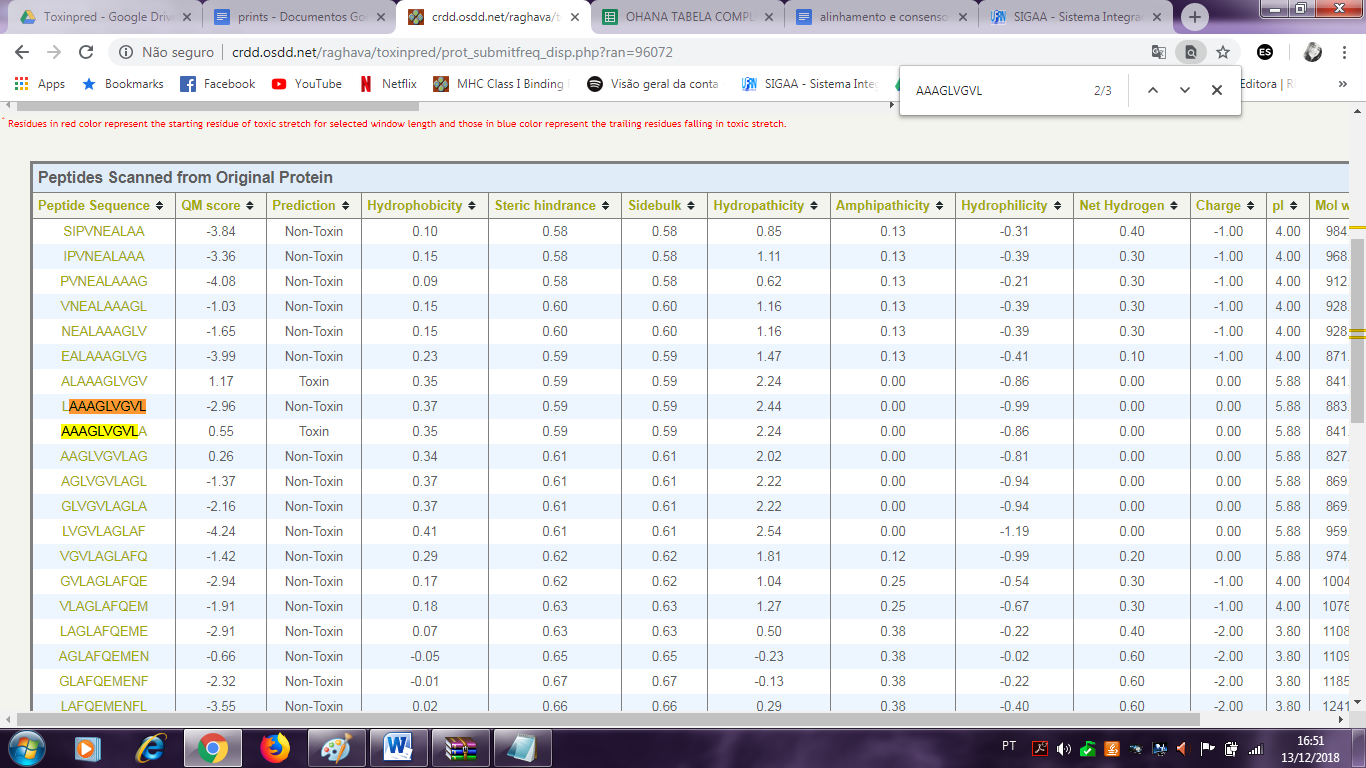


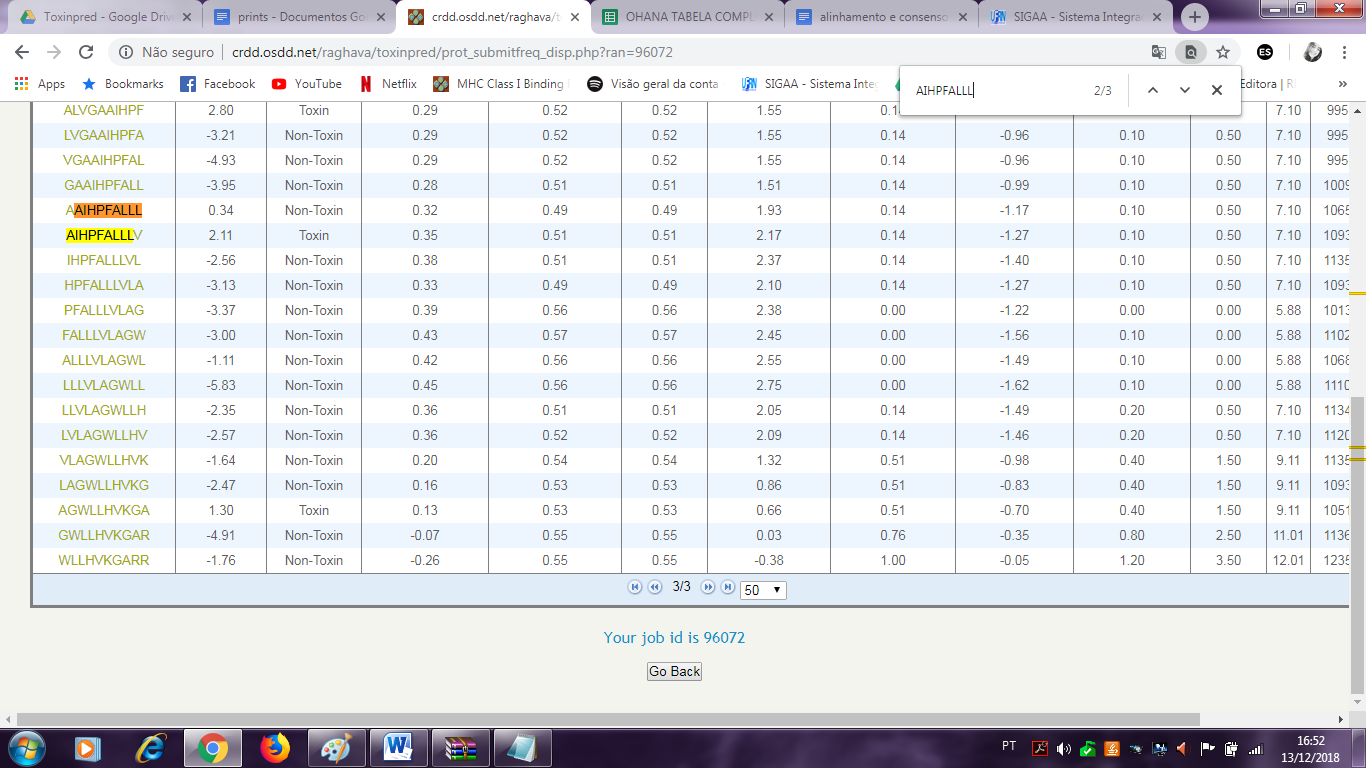


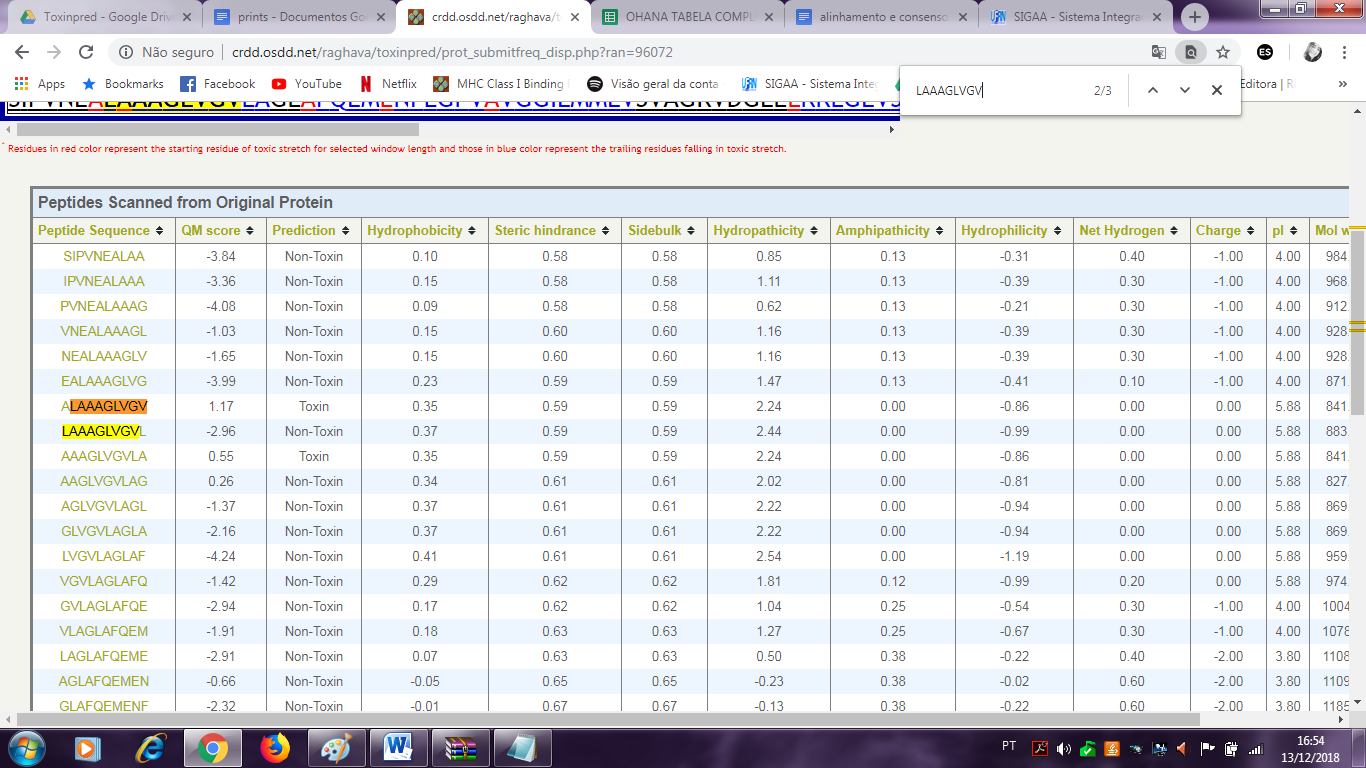


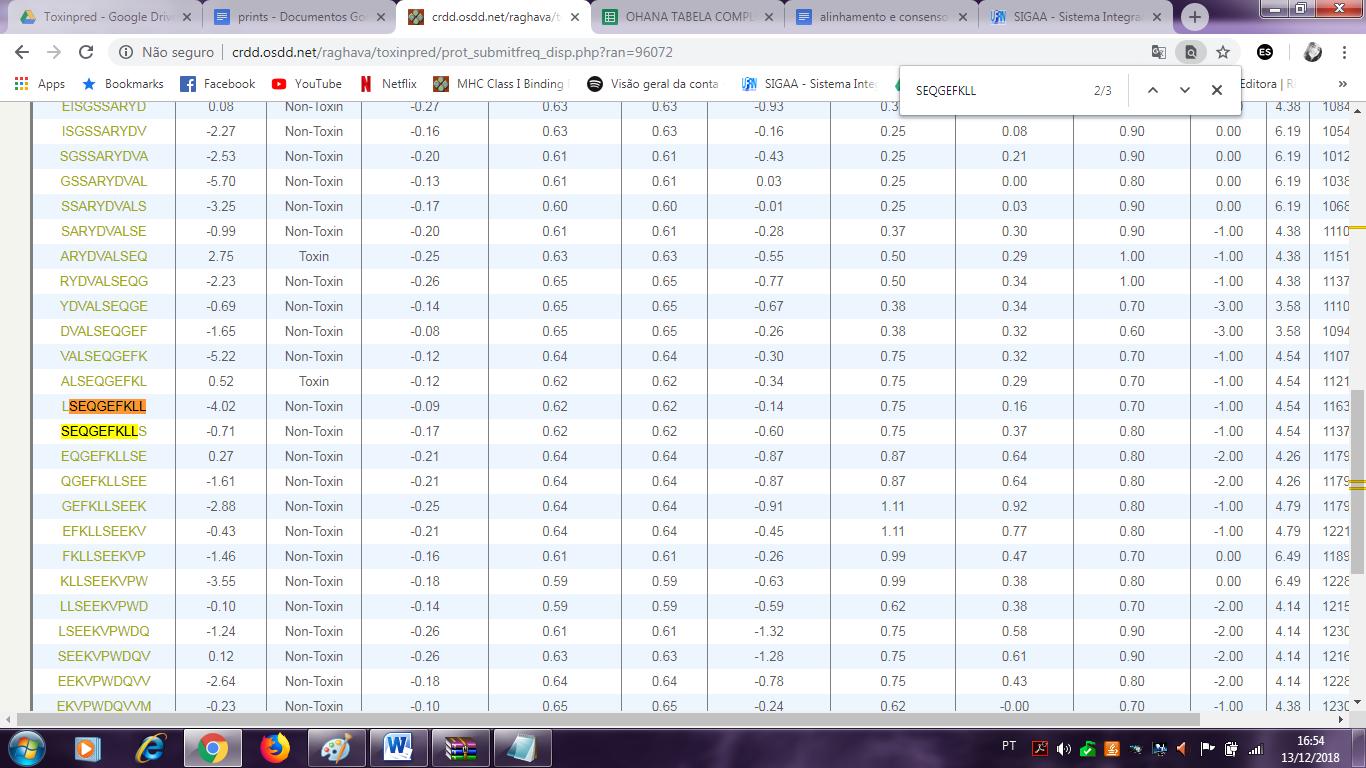


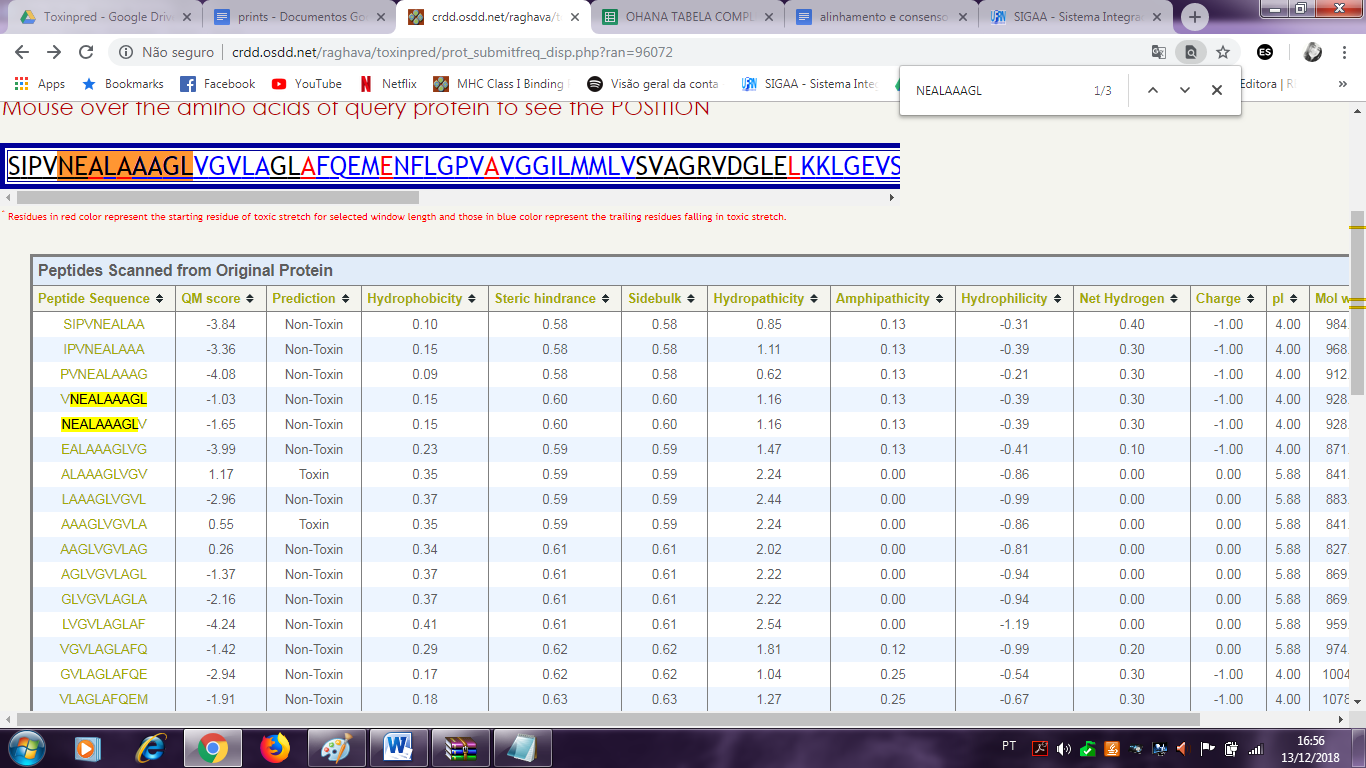


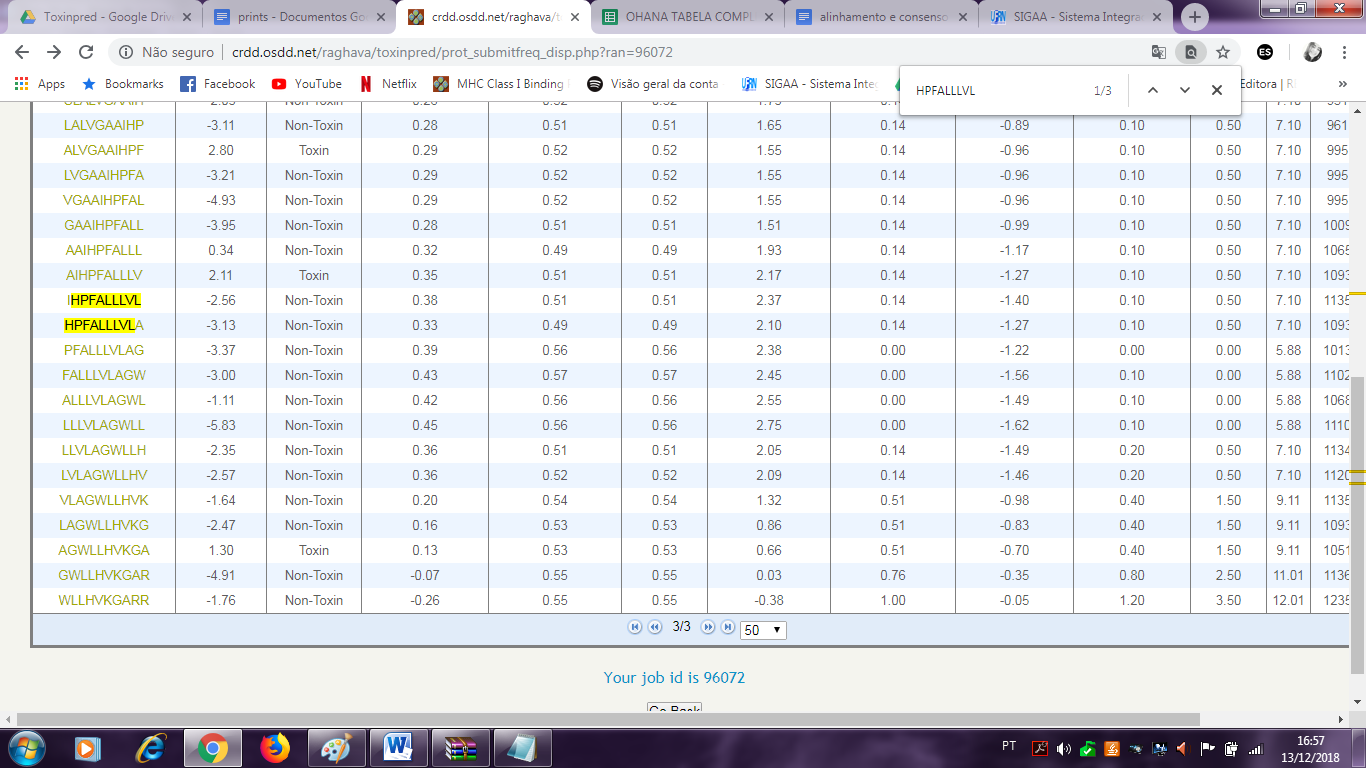


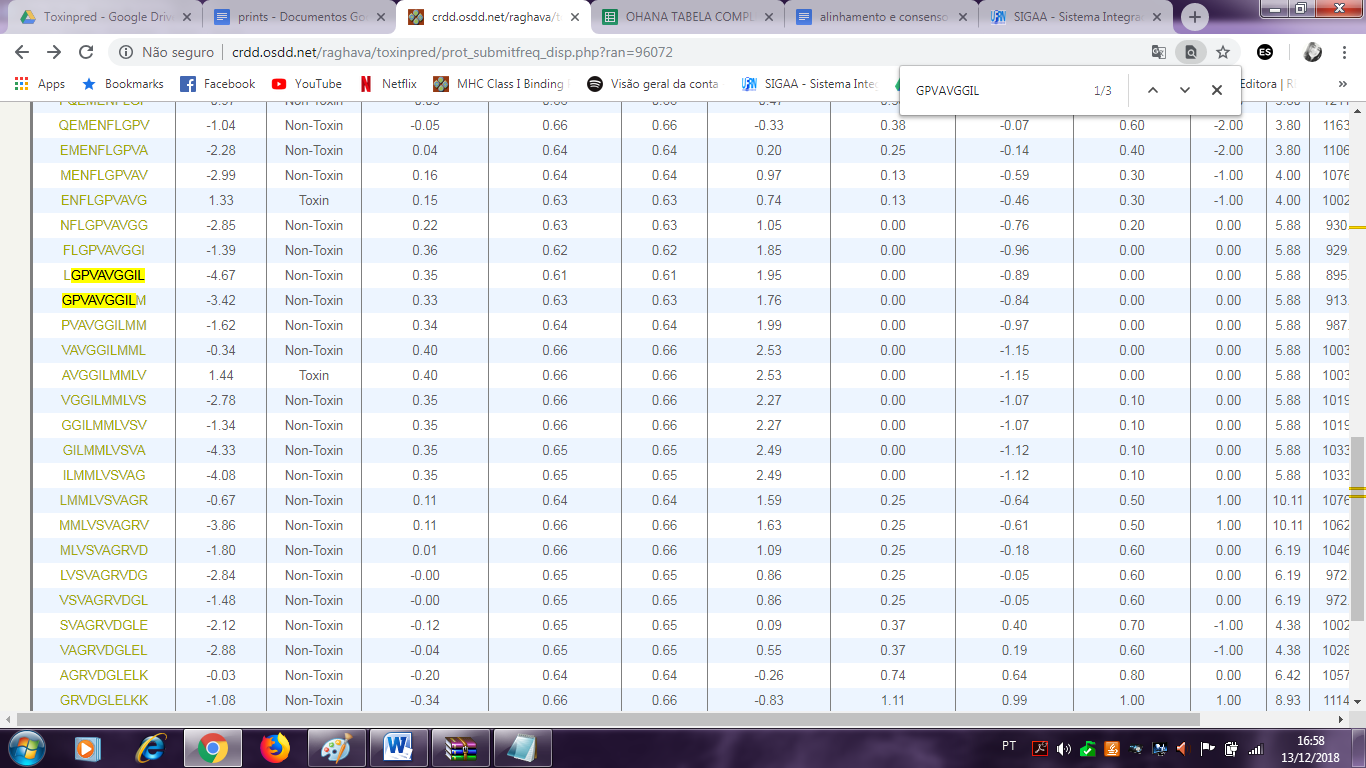


NS3


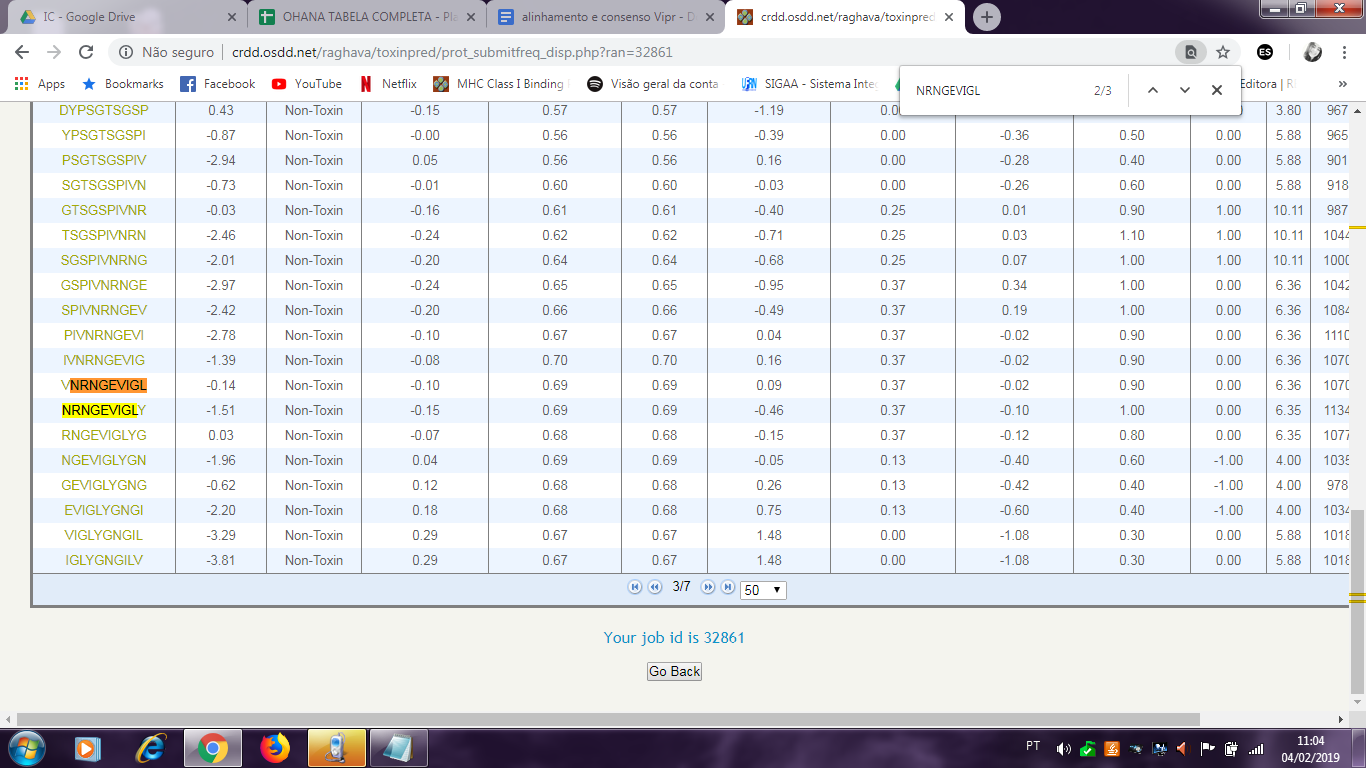


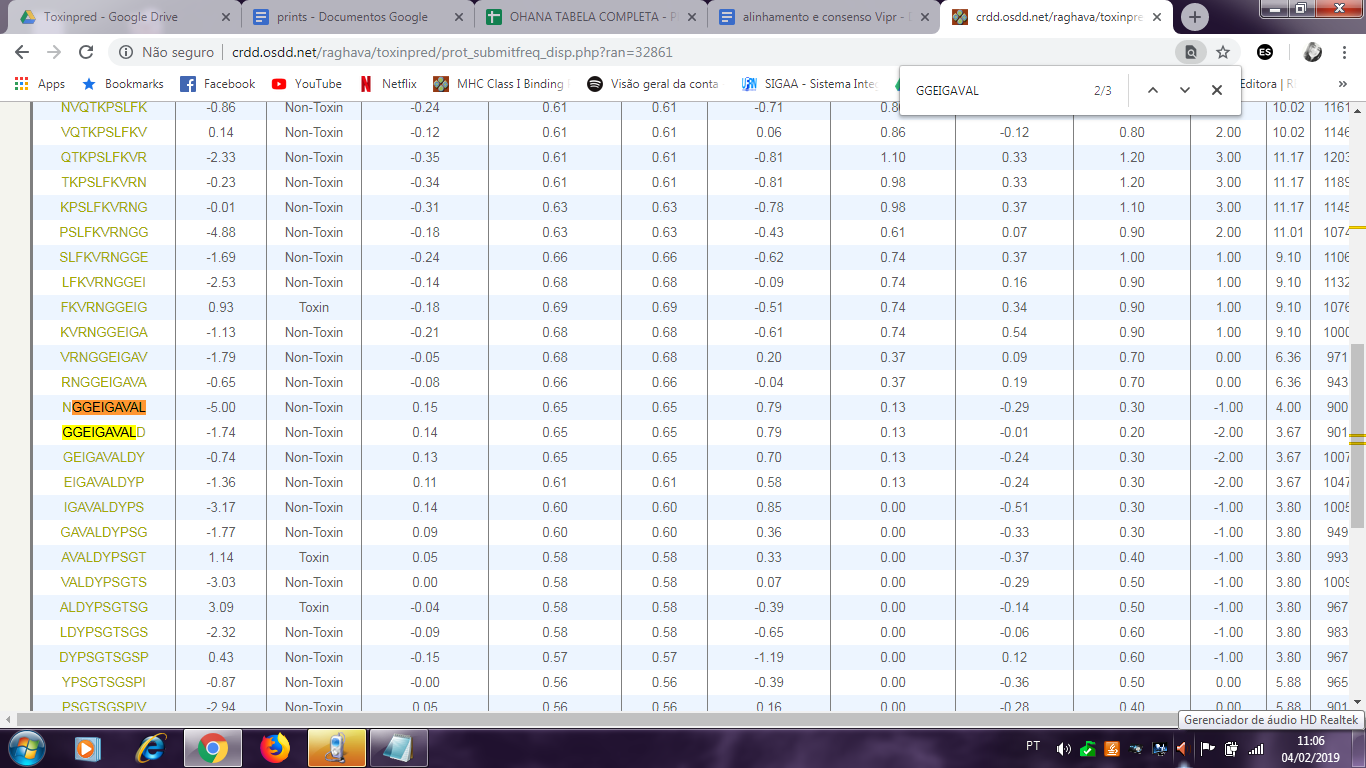


NS4A


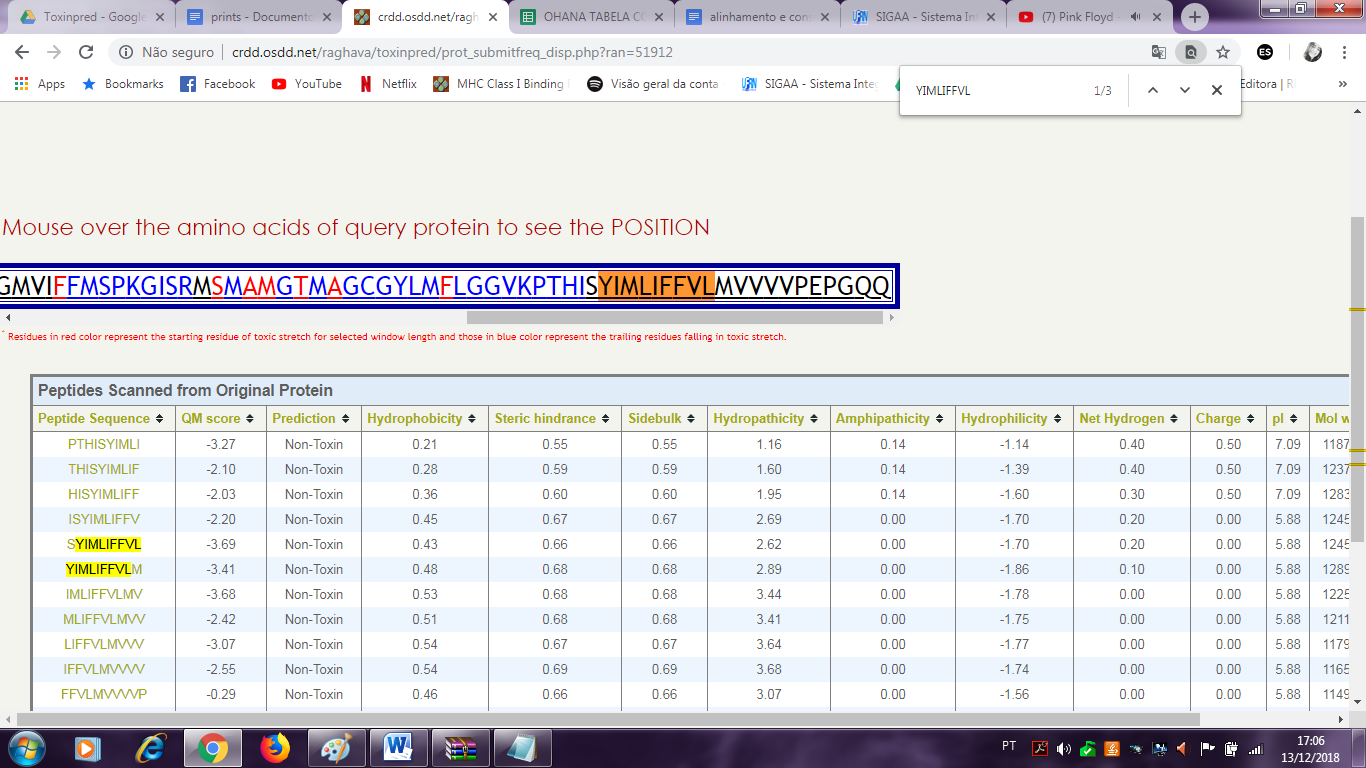


NS4B


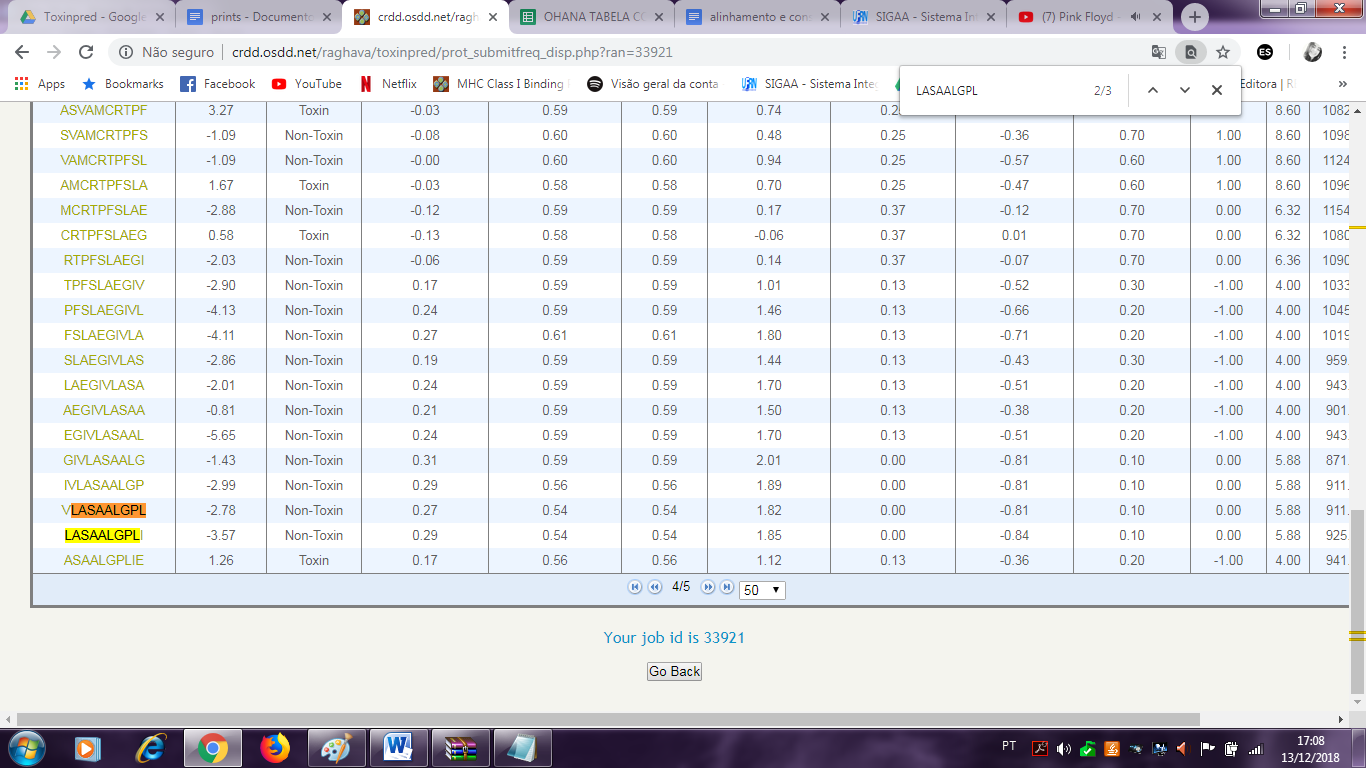


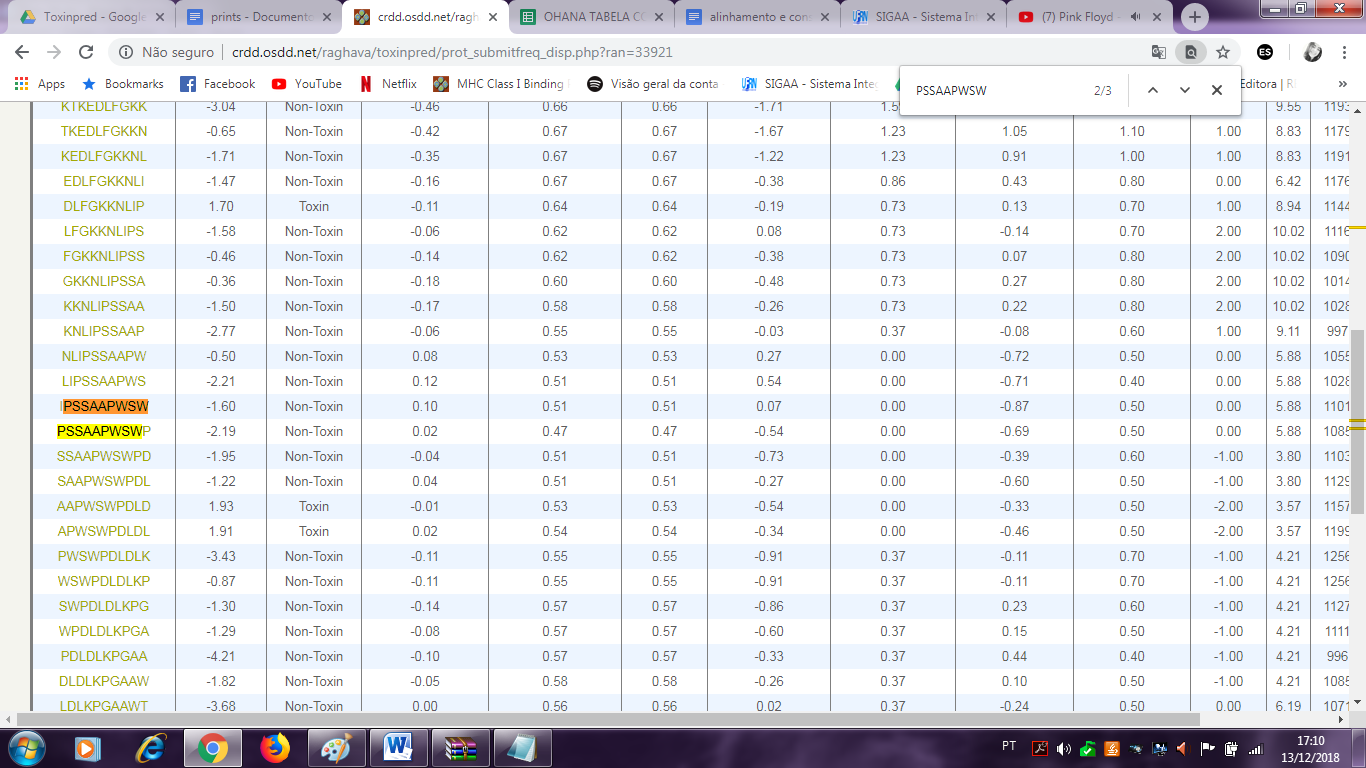


NS5


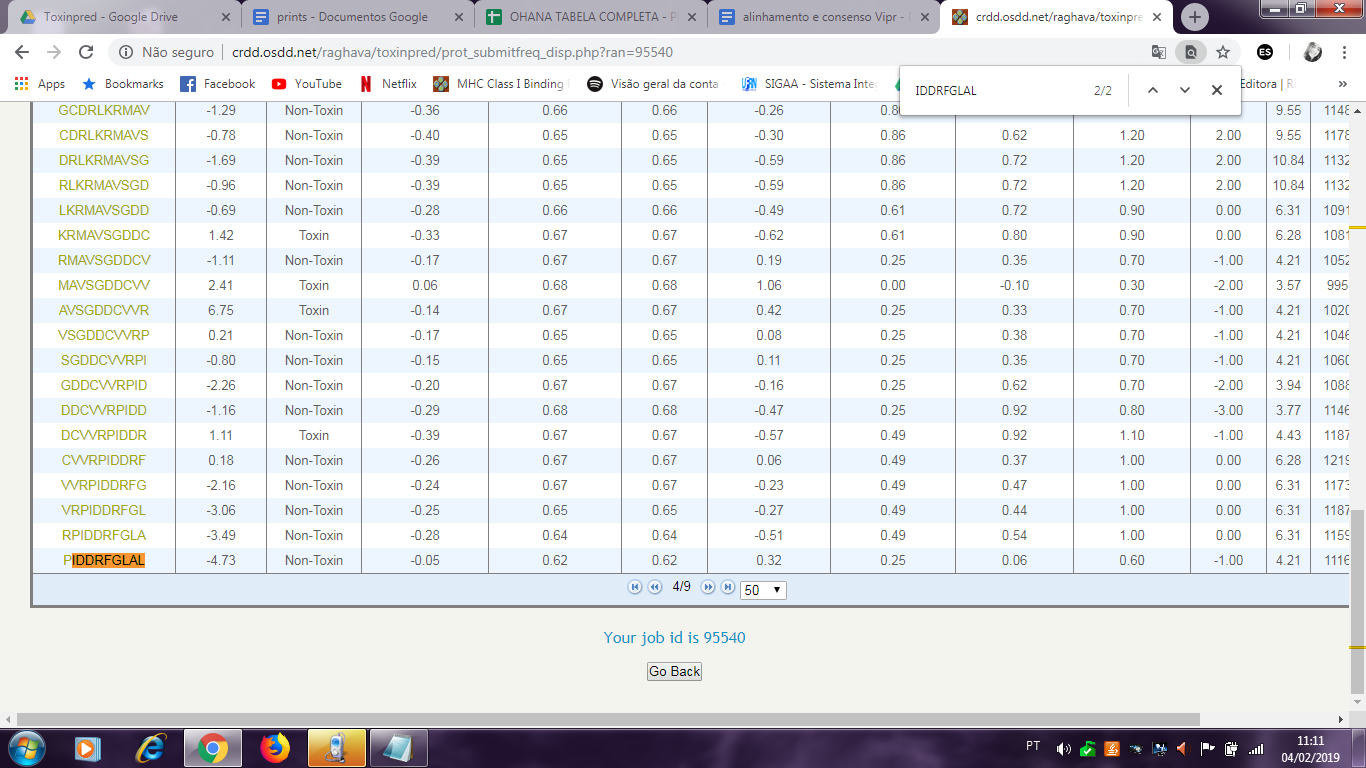

Supplement: Supplementary file 1 — Supplementary Information. [file 41598_2024_60680_MOESM1_ESM.zip › Yellow_Fever_data/2_Prediction of T-cell epitopes/Toxinpred_Toxicity/prints.docx]

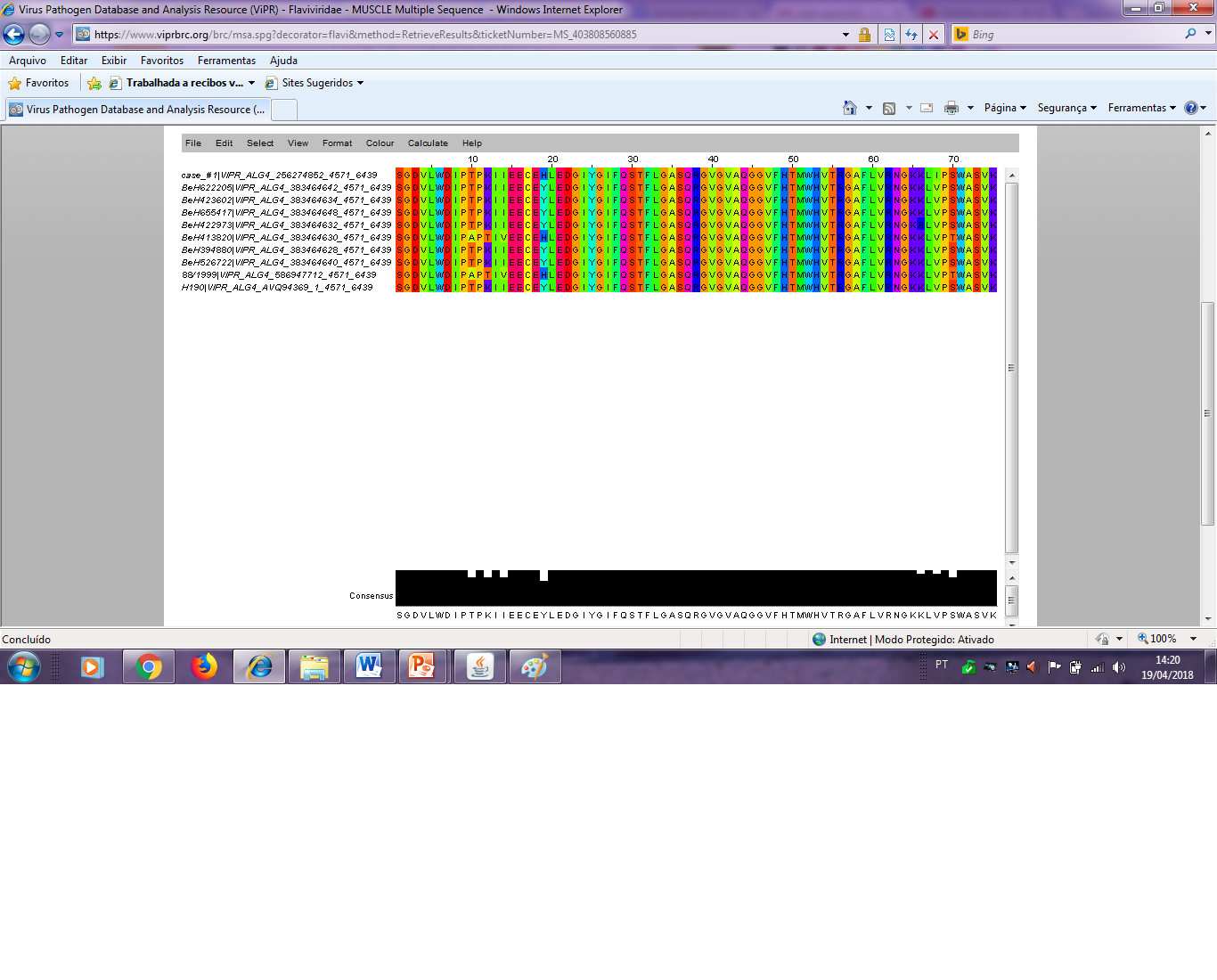

Supplement: Supplementary file 1 — Supplementary Information. [file 41598_2024_60680_MOESM1_ESM.zip › Yellow_Fever_data/1_Acquisition_proteins/Prints VIPR/ns3/passo 3 ns3 consenso.png]

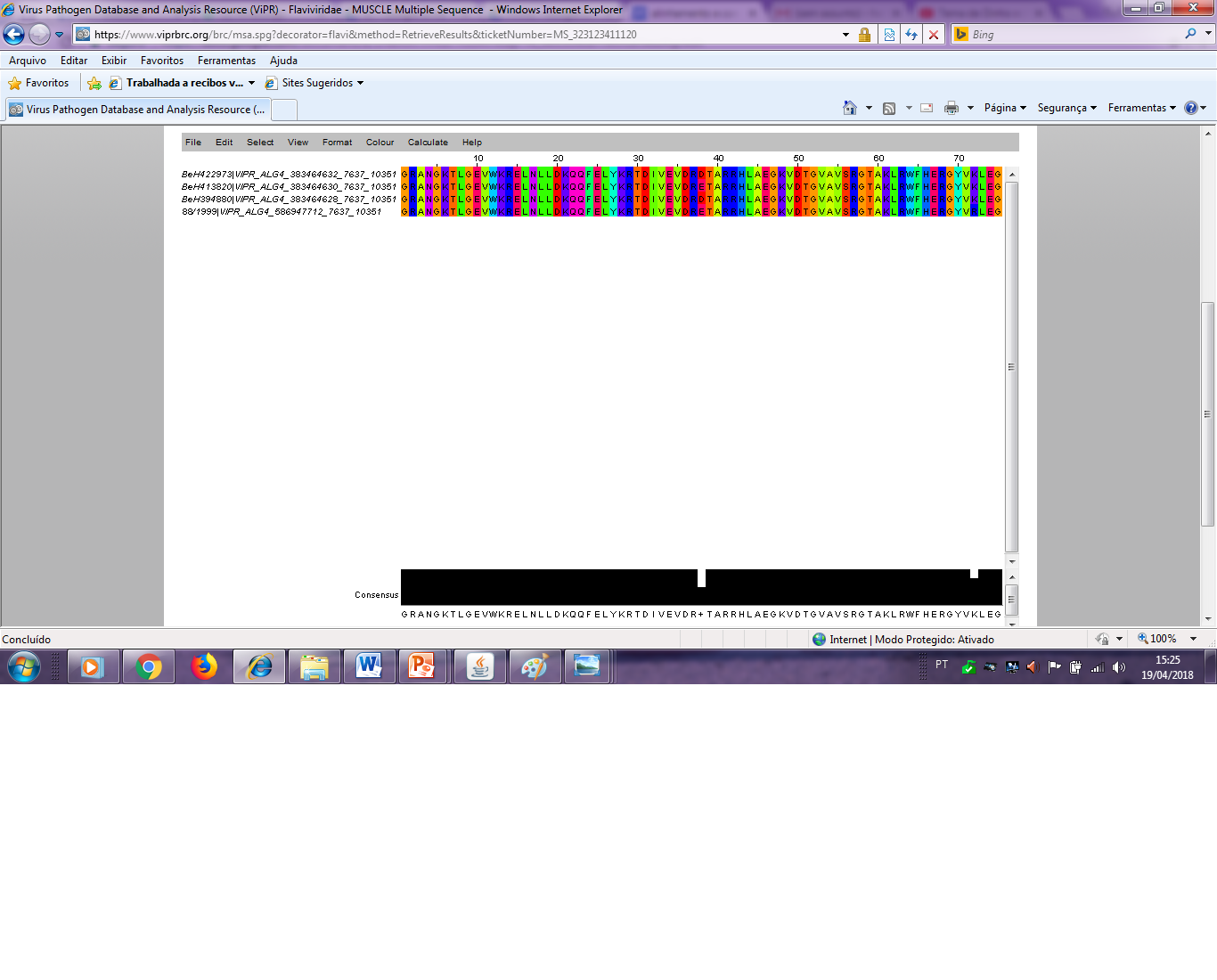

Supplement: Supplementary file 1 — Supplementary Information. [file 41598_2024_60680_MOESM1_ESM.zip › Yellow_Fever_data/1_Acquisition_proteins/Prints VIPR/ns5/passo 3 ns5 consenso.png]

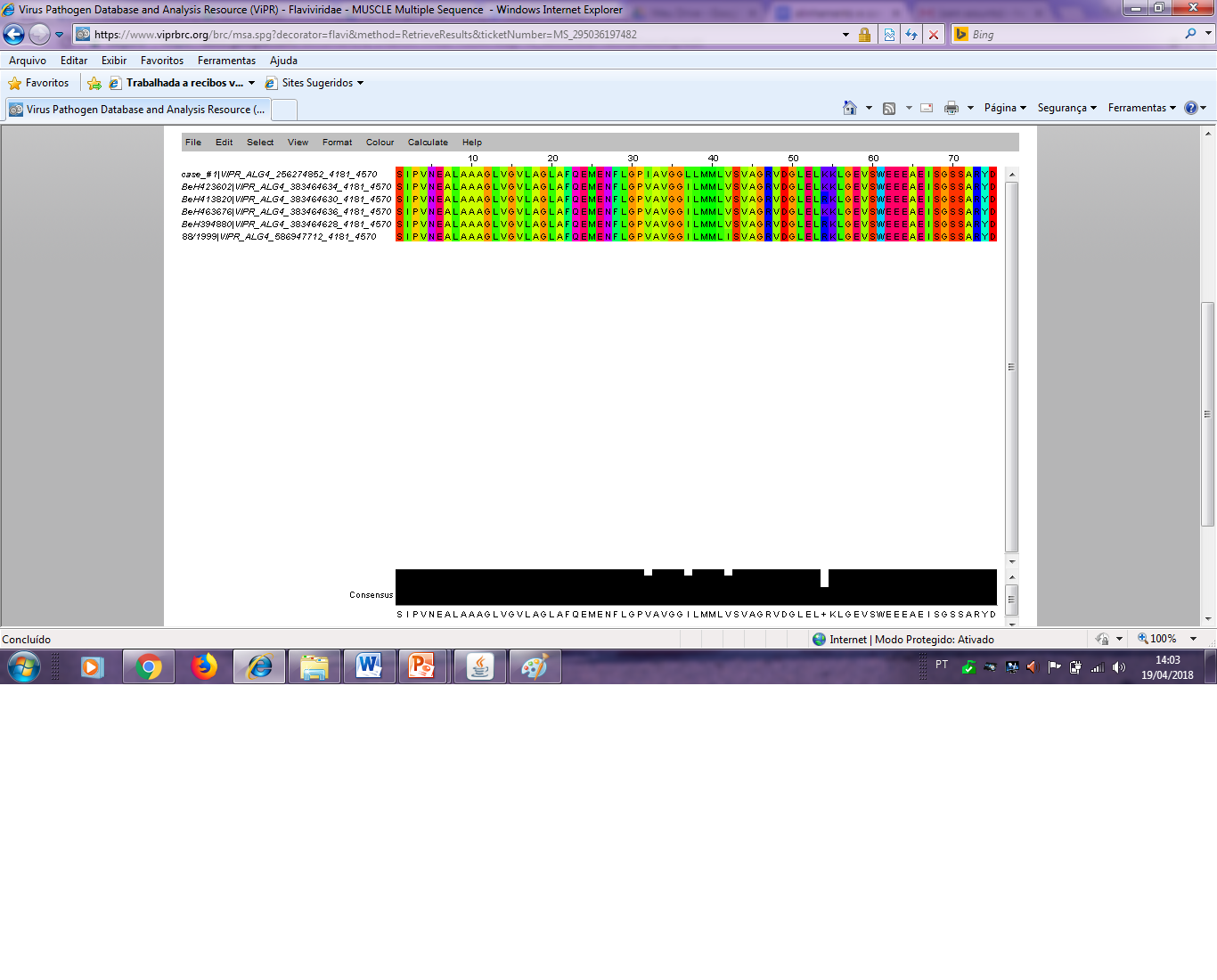

Supplement: Supplementary file 1 — Supplementary Information. [file 41598_2024_60680_MOESM1_ESM.zip › Yellow_Fever_data/1_Acquisition_proteins/Prints VIPR/ns2b/passo 3 ns2b consenso.png]

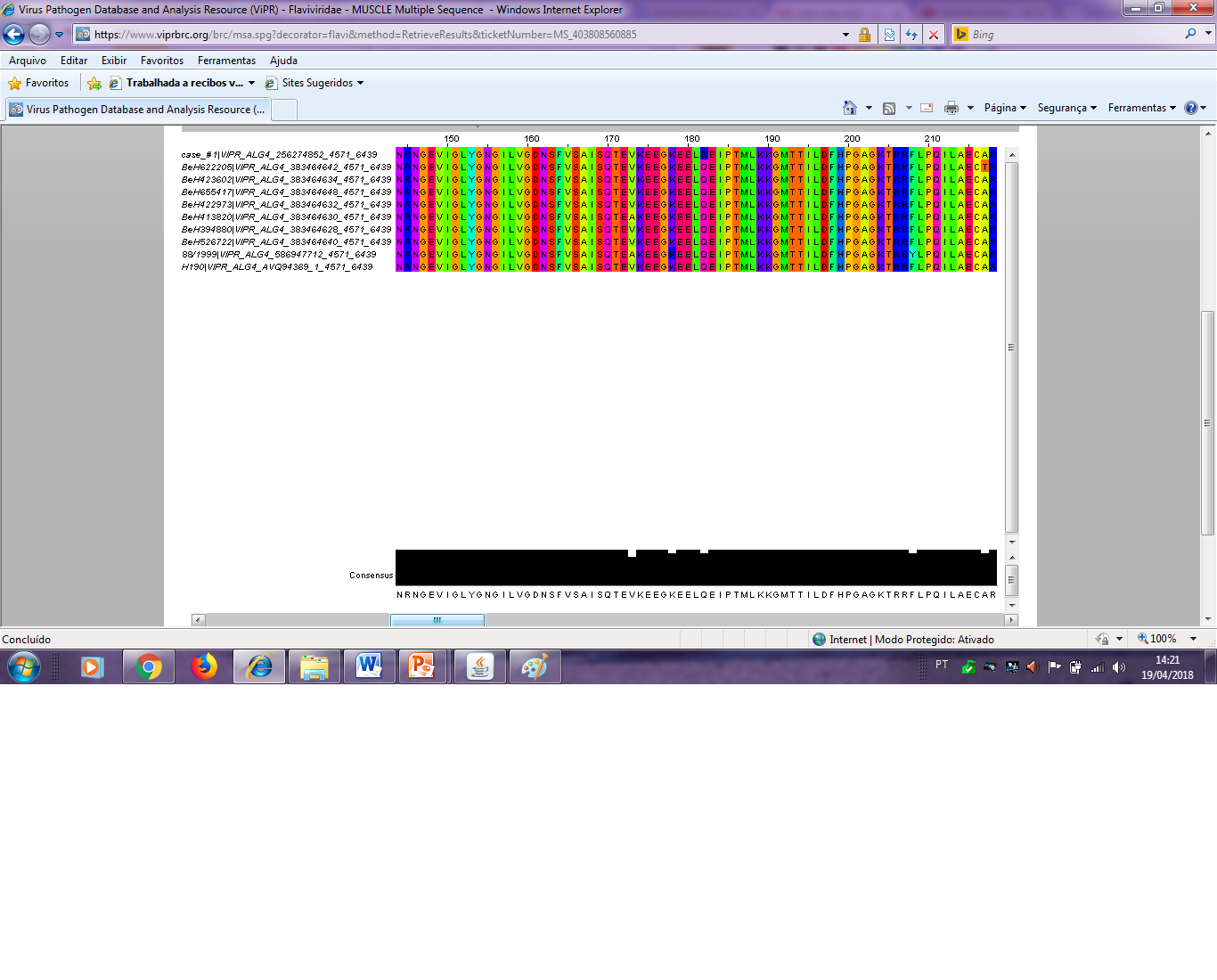

Supplement: Supplementary file 1 — Supplementary Information. [file 41598_2024_60680_MOESM1_ESM.zip › Yellow_Fever_data/1_Acquisition_proteins/Prints VIPR/ns3/passo 3.3 ns3 consenso.png]

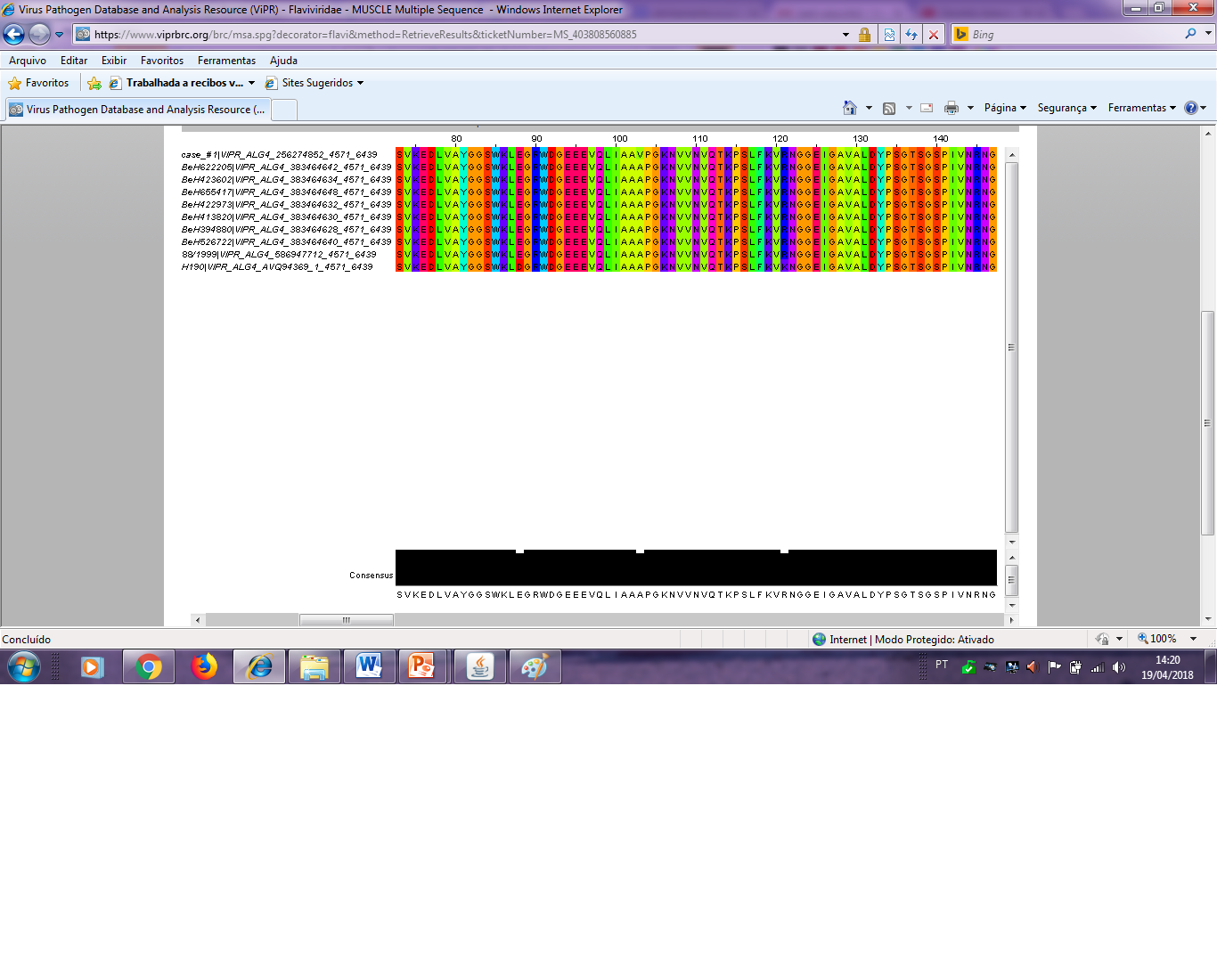

Supplement: Supplementary file 1 — Supplementary Information. [file 41598_2024_60680_MOESM1_ESM.zip › Yellow_Fever_data/1_Acquisition_proteins/Prints VIPR/ns3/passo 3.2 ns3 consenso.png]

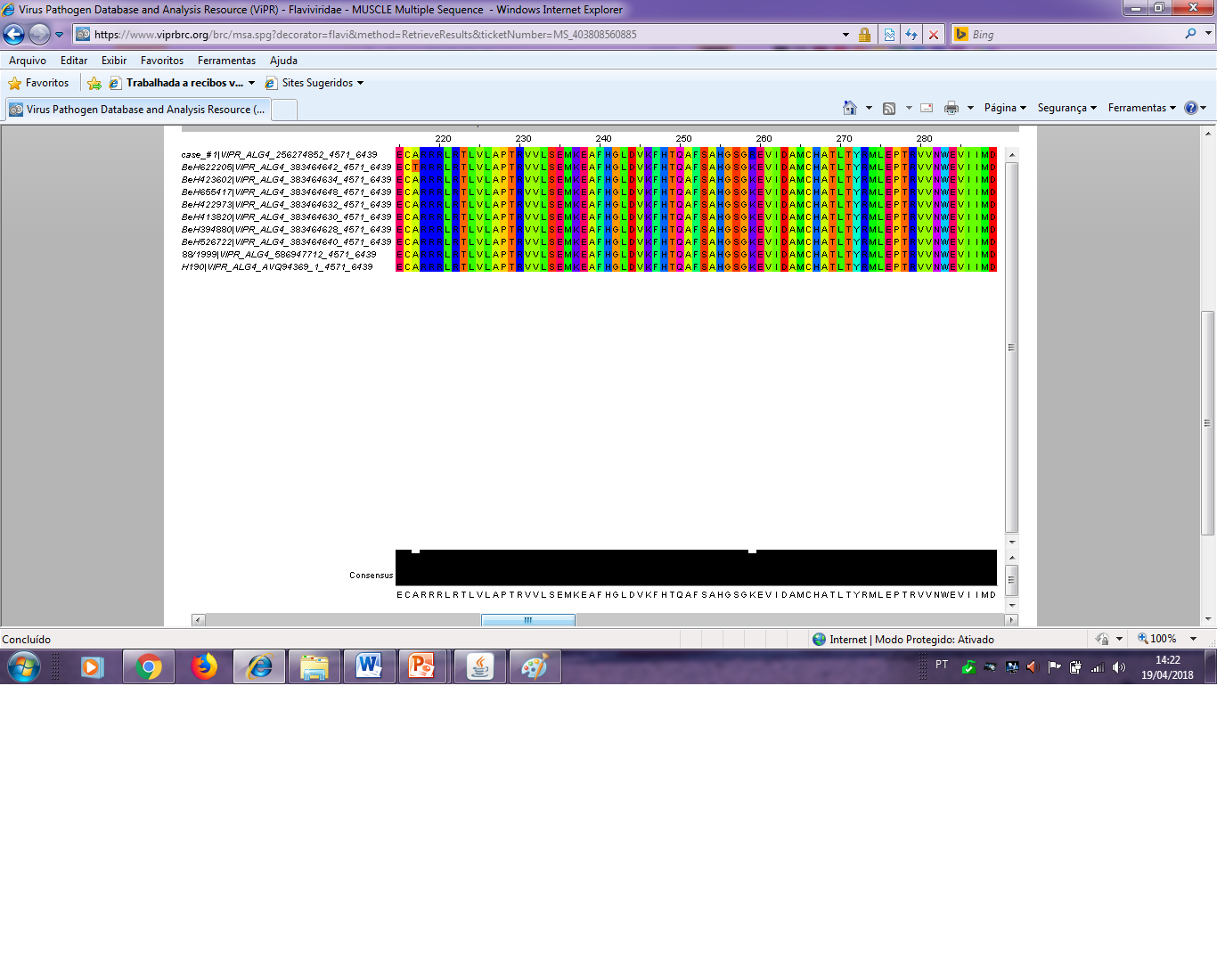

Supplement: Supplementary file 1 — Supplementary Information. [file 41598_2024_60680_MOESM1_ESM.zip › Yellow_Fever_data/1_Acquisition_proteins/Prints VIPR/ns3/passo 3.4 ns3 consenso.png]

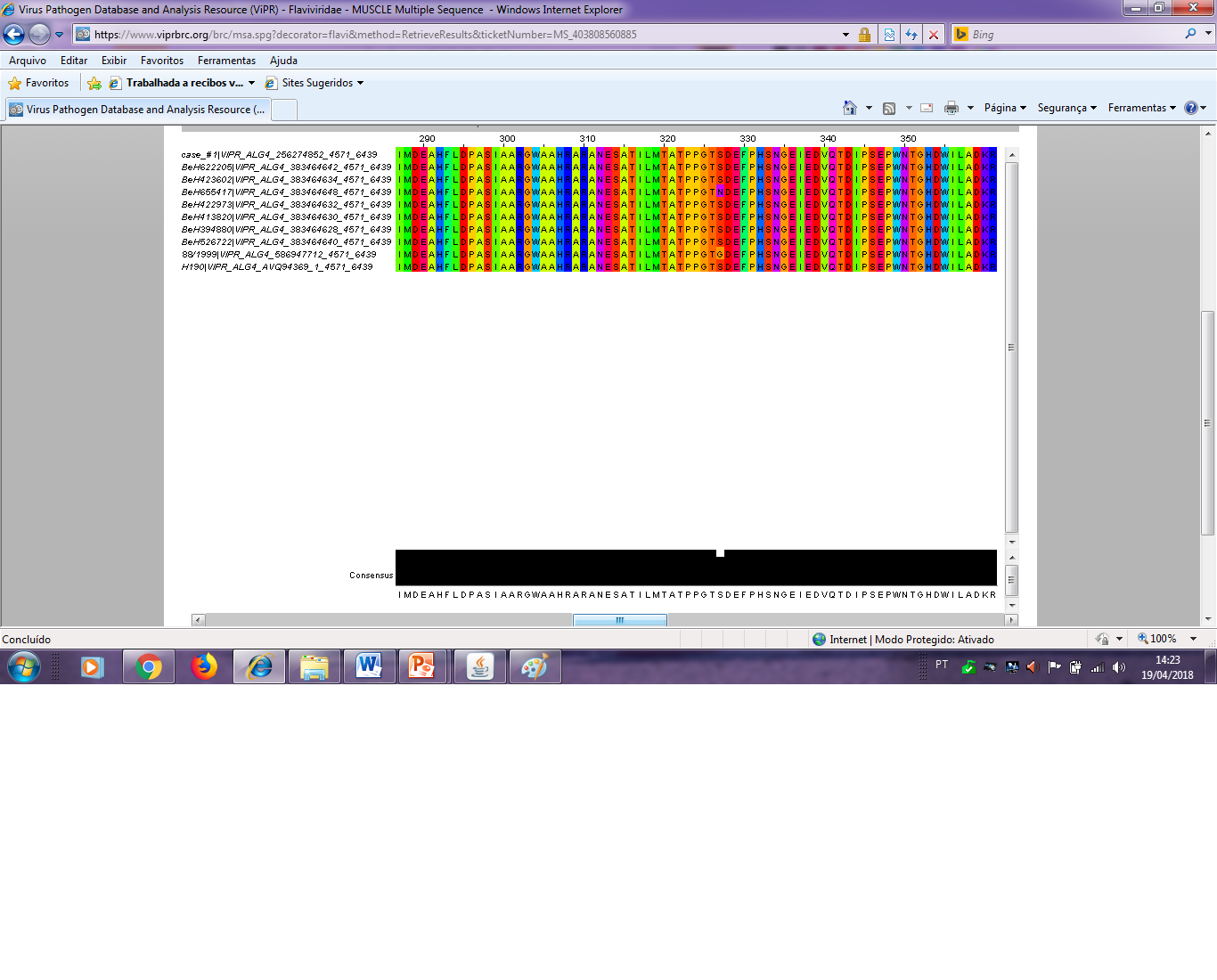

Supplement: Supplementary file 1 — Supplementary Information. [file 41598_2024_60680_MOESM1_ESM.zip › Yellow_Fever_data/1_Acquisition_proteins/Prints VIPR/ns3/passo 3.5 ns3 consenso.png]

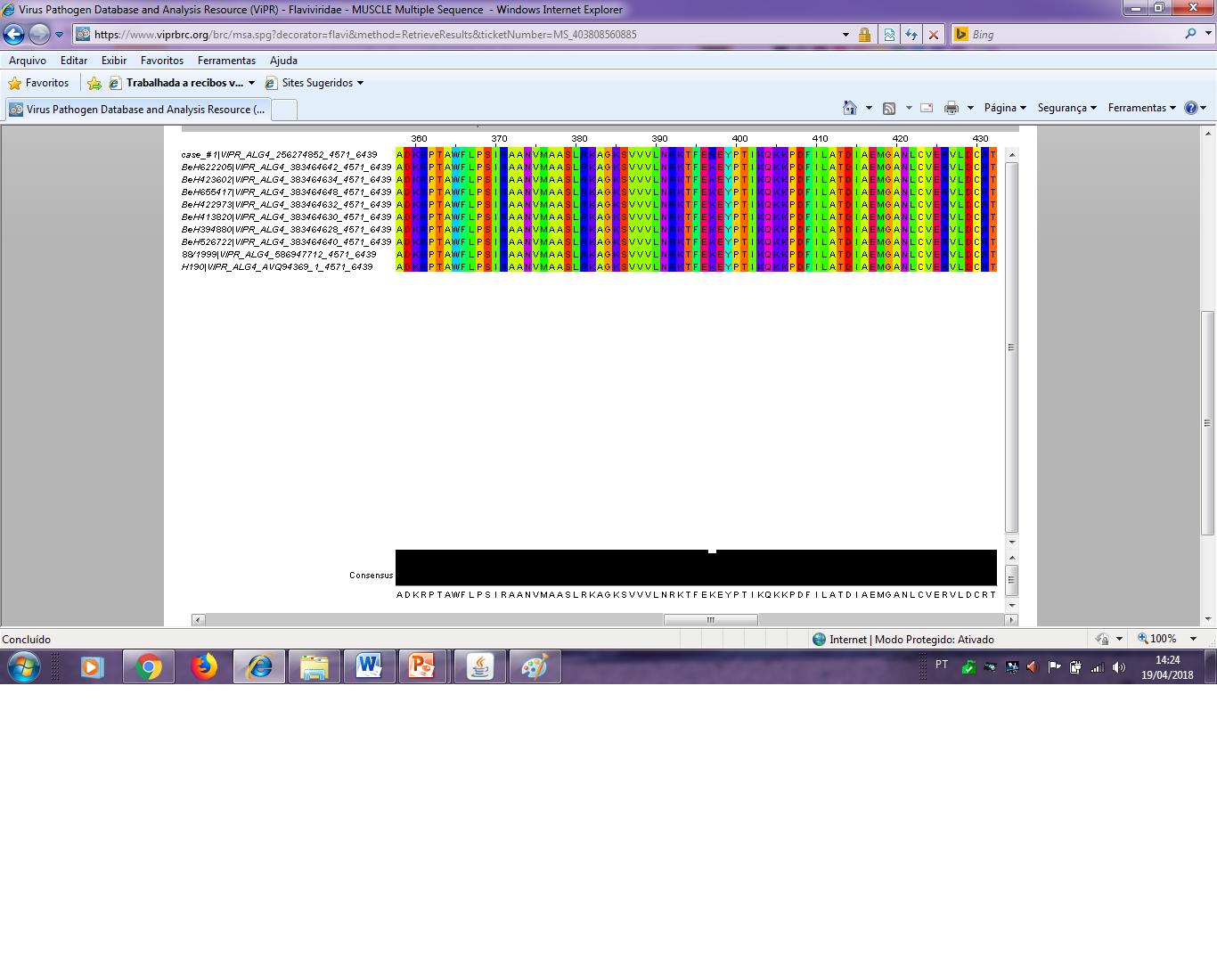

Supplement: Supplementary file 1 — Supplementary Information. [file 41598_2024_60680_MOESM1_ESM.zip › Yellow_Fever_data/1_Acquisition_proteins/Prints VIPR/ns3/passo 3.6 ns3 consenso.png]

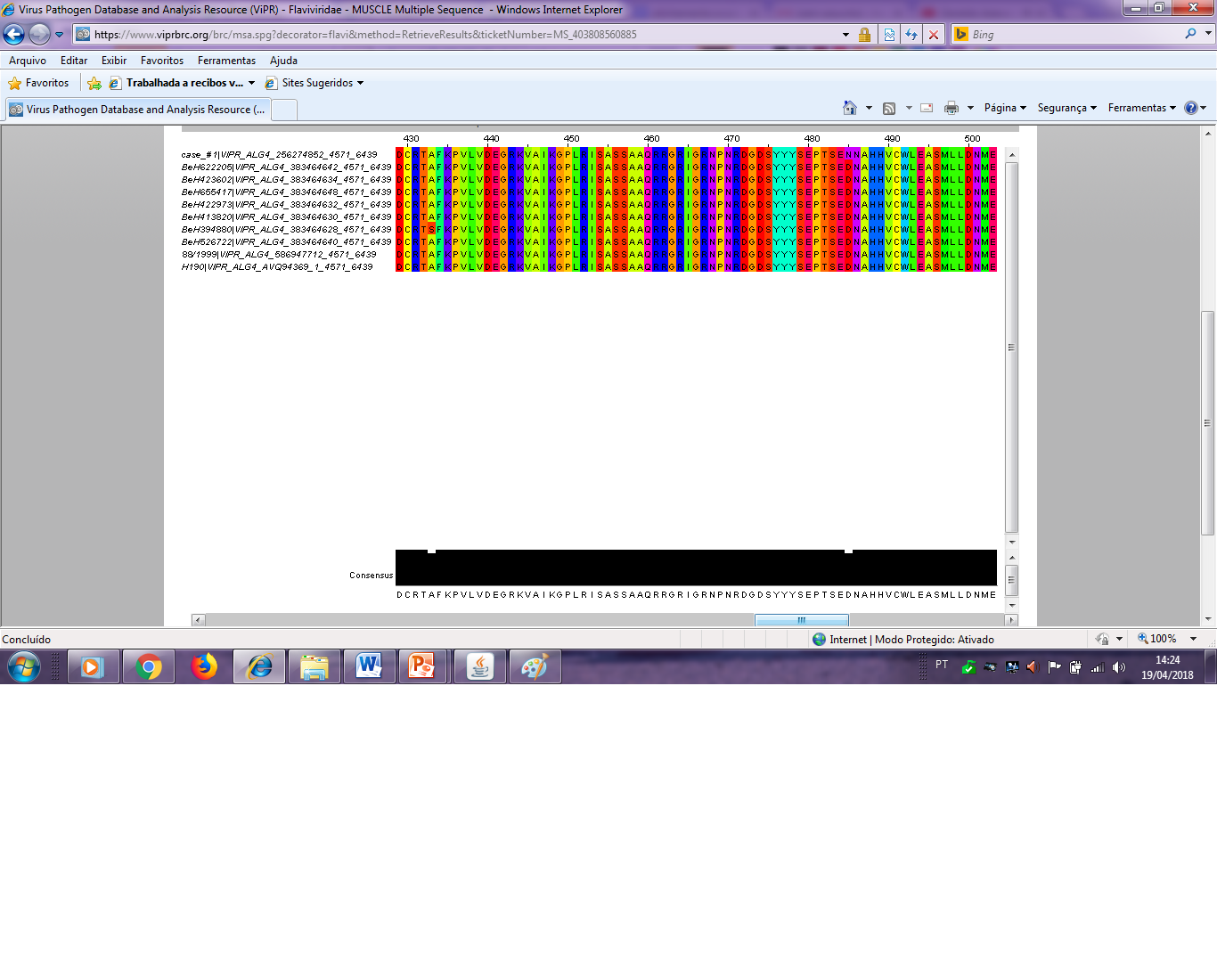

Supplement: Supplementary file 1 — Supplementary Information. [file 41598_2024_60680_MOESM1_ESM.zip › Yellow_Fever_data/1_Acquisition_proteins/Prints VIPR/ns3/passo 3.7 ns3 consenso.png]

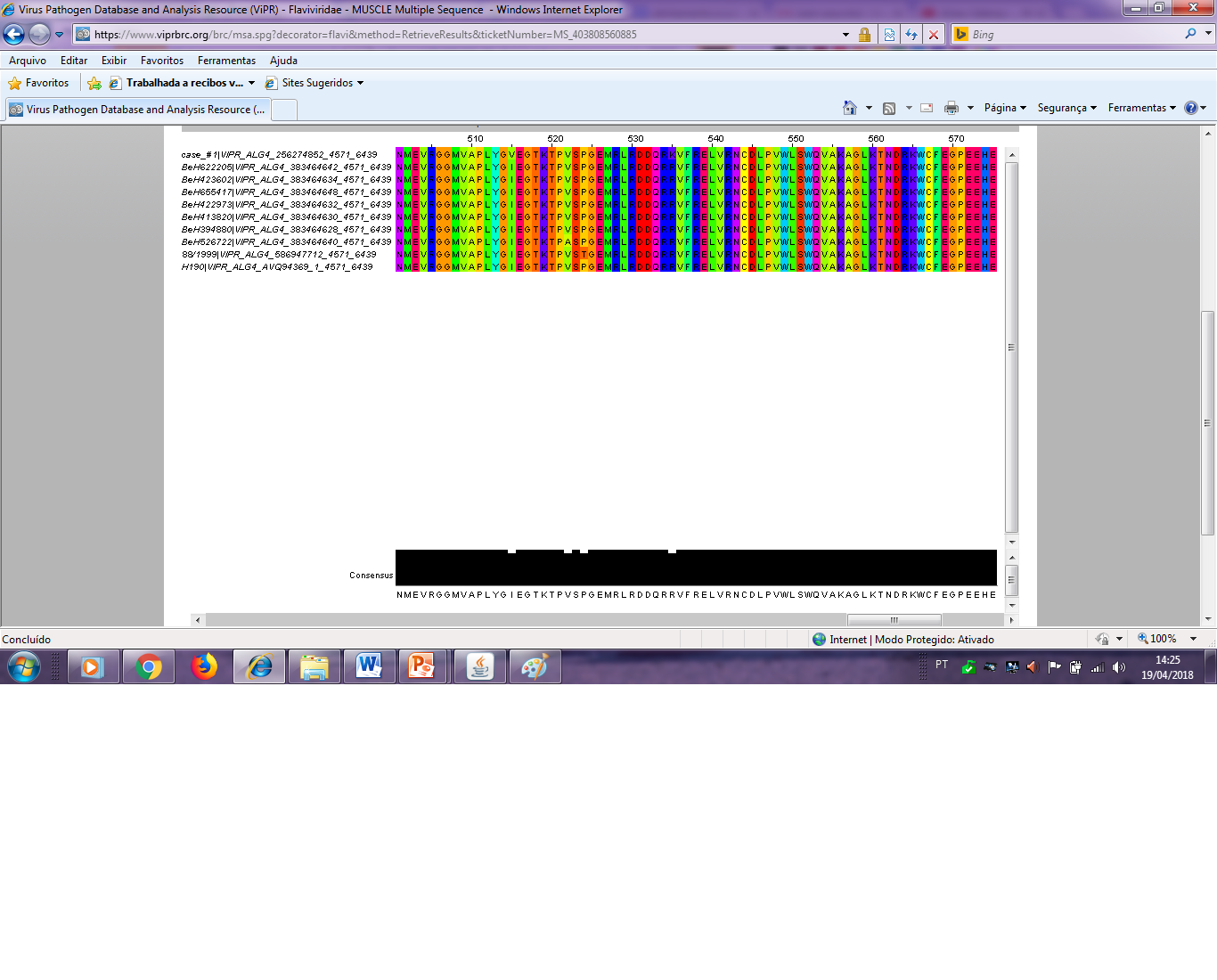

Supplement: Supplementary file 1 — Supplementary Information. [file 41598_2024_60680_MOESM1_ESM.zip › Yellow_Fever_data/1_Acquisition_proteins/Prints VIPR/ns3/passo 3.8 ns3 consenso.png]

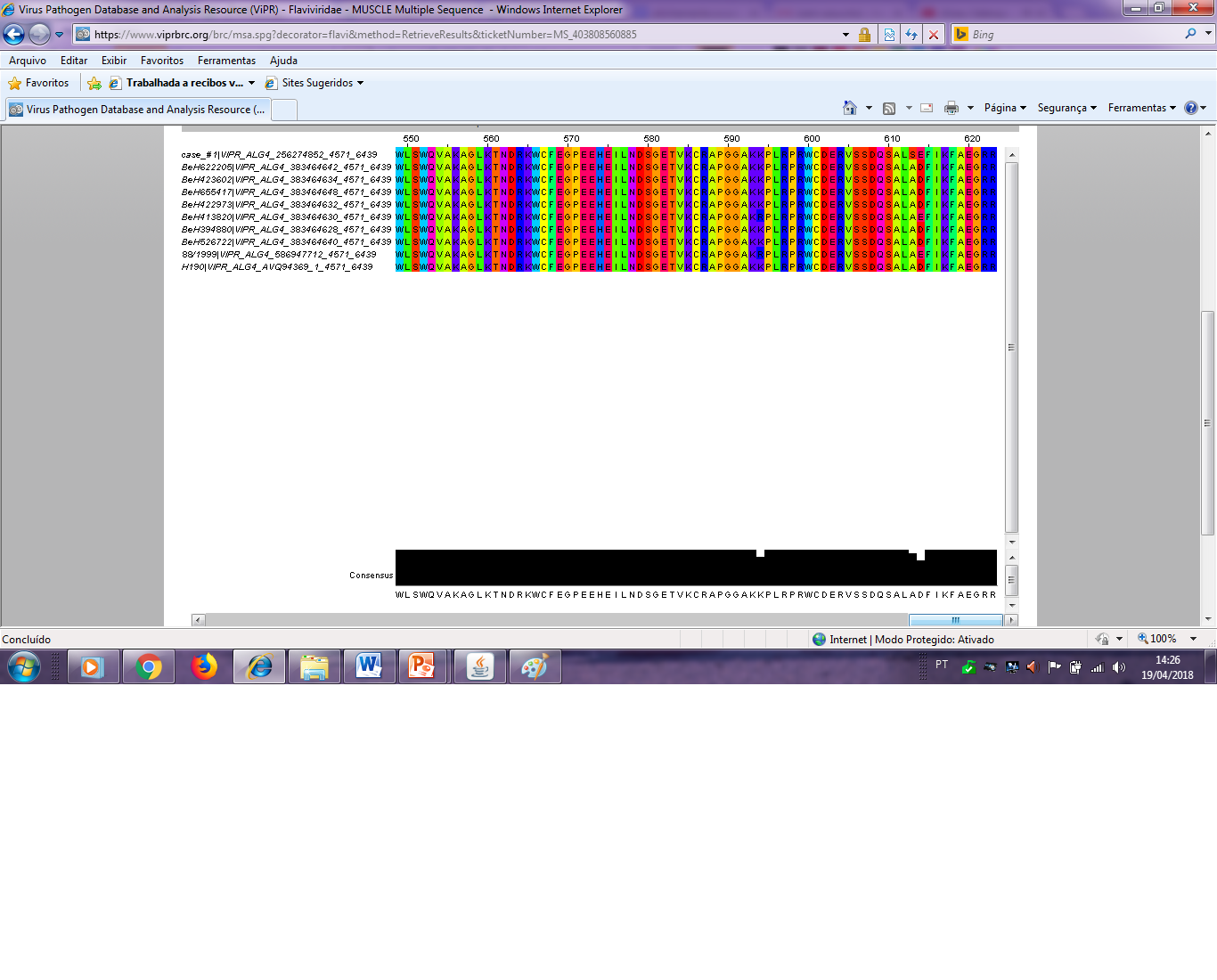

Supplement: Supplementary file 1 — Supplementary Information. [file 41598_2024_60680_MOESM1_ESM.zip › Yellow_Fever_data/1_Acquisition_proteins/Prints VIPR/ns3/passo 3.9 ns3 consenso.png]

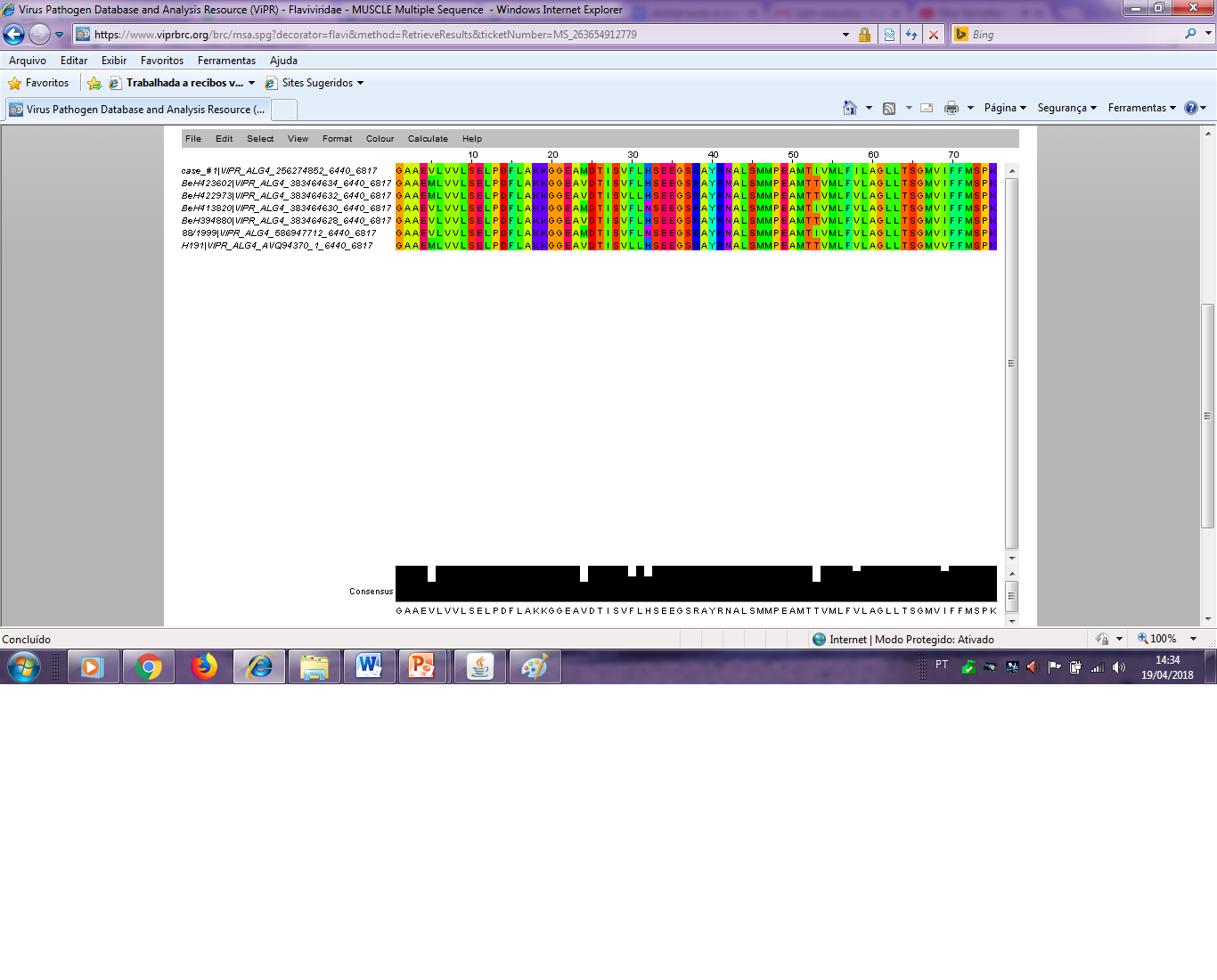

Supplement: Supplementary file 1 — Supplementary Information. [file 41598_2024_60680_MOESM1_ESM.zip › Yellow_Fever_data/1_Acquisition_proteins/Prints VIPR/ns4a/passo 3 ns4a consenso.png]
